# Base-Promoted C→N Carbonyl Shift Enables One-Step Access to aza-β-Lactams from α‑Heteroaryl-α-amino Esters

**DOI:** 10.1021/acs.orglett.6c01451

**Published:** 2026-05-11

**Authors:** Jozef Kristek, Nikola Št’astná, Jiří Pospíšil

**Affiliations:** a Department of Organic Chemistry, Faculty of Science, Palacký University, tř. 17. listopadu 1192/12, Olomouc CZ-771 46, Czech Republic; b Department of Chemical Biology, Faculty of Science, Palacký University, Šlechtitelů 27, Olomouc CZ-78371, Czech Republic; c Laboratory of Growth Regulators, Institute of Experimental Botany of the Czech Academy of Sciences, and Faculty of Science, Palacký University, Šlechtitelů 27, Olomouc CZ-78371, Czech Republic

## Abstract

Aza-β-lactams arise directly from α-heteroaryl-α-amino esters through an *i*PrMgCl-promoted C→N carbonyl shift, hydroxylamine capture, and [1,2]-heteroaryl migration. The reaction proceeds in one step, scales to grams, tolerates diverse Cα-alkyl groups and hydroxylamines, and reveals a predictable divergence to five-membered ureas under strongly basic conditions or with electron-poor *N*-substituents. Isolation and productive re-entry of a carbamate intermediate support a reversible manifold preceding migration and rationalize racemic product formation.

## Graphical abstract

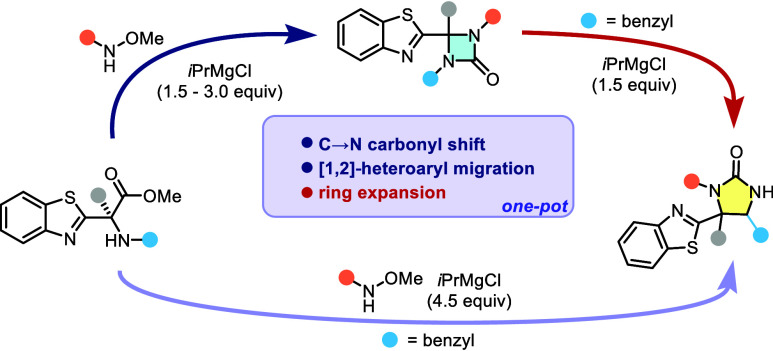

Four-membered nitrogen heterocycles are attractive scaffolds for covalent inhibitor design, as their inherent ring strain and carbonyl electrophilicity enable selective acylation of serine hydrolases (Figure 1A).^1–3^ Classical β-lactams exemplify this reactivity; however, their clinical utility is increasingly compromised by the global spread of β-lactamase-mediated resistance, including carbapenemases and metallo-β-lactamases, for which no broad-spectrum inhibitors are currently available.^4–7^ These challenges motivate the development of alternative strained lactam frameworks that preserve strain-enabled reactivity while circumventing established resistance mechanisms. In this context, replacement of the carbonyl-adjacent methylene unit with nitrogen affords 1,3-diazetidin-2-ones (aza-β-lactams) with distinct electronic properties.^8^ Despite this potential, access to these motifs remains limited since prevailing approaches, such as [2 + 2] imine–isocyanate cycloadditions and photochemical rearrangements, often require specialized conditions and display restricted scope (Figure 1B).^9–11^

**Figure 1:**
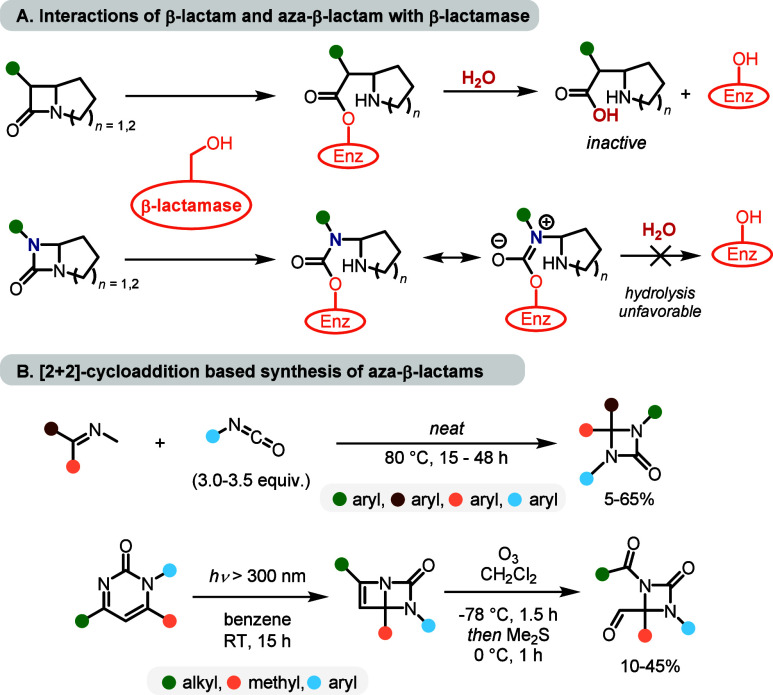
(A) Simplified mode of action of β-lactam antibiotics (experimental) and aza-β-lactams (*in silico* predicted) with β-lactamase inhibitor. (B) Previously employed synthetic approaches to aza-β-lactams, including [2 + 2] imine–isocyanate cycloadditions and photochemical pyrimidinone rearrangements.

While investigating α-heteroaryl-α-amino esters (HAAs) as precursors to 1,2-diazetidin-3-ones, we uncovered an unexpected reactivity manifold involving a base-promoted intramolecular C→N carbonyl shift followed by [1,2]-heteroaryl migration, which directly delivered aza-β-lactams (Figure 2A). This finding prompted development of a single-operation conversion from readily available HAA esters and hydroxylamines.^12^ A systematic base screen identified *i*PrMgCl as uniquely effective (Figure 2B): organolithium reagents and LiHMDS favored carbonyl transfer or carbamate formation, tertiary amines were unreactive, and initiation at −40 °C with controlled warming to 0 °C maximized aza-β-lactam formation while suppressing the carbamate pathway (**6a**). Under optimized conditions, **3a** was obtained in 84% NMR yield (76% isolated). In contrast, chloride additives promoted carbamate formation, and ≥4.5 equiv of *i*PrMgCl induced a clean divergence to five-membered ureas (Table S1).

**Figure 2:**
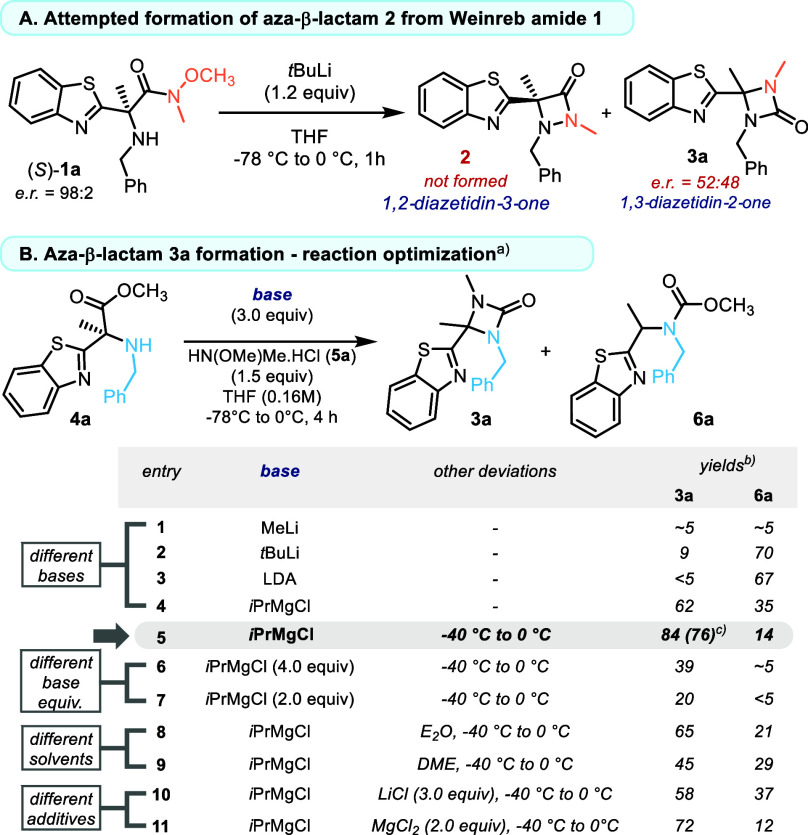
Optimization table for the base-promoted transformation of HAA **4a** into aza-β-lactam **3a**. Selected reaction parameters and their influence on product distribution; see Table S1 for full optimization data. ^a)^Reactions performed on a 0.07 mmol scale of **4a**. ^b)^NMR yields determined using trichloroethylene (TCE) as internal standard. ^c)^Isolated yield.

With this operating window, we established a broad yet bounded scope (Figure 3). Benzo­[*d*]­thiazole-derived HAAs **4** bearing diverse Cα-alkyl substituents furnished aza-β-lactams **3** in moderate to good yields, and the reaction tolerated numerous *O-* and *N-*substituted hydroxylamines **5**, including cyclic variants: isoxazolidine and oxazinane salts delivered hydroxyl-functionalized products **3w** and **3x** in 68 and 70% isolated yield and proved amenable to further derivatization. In contrast, additional ester substitution on the HAA (glutamate-like motifs) compromised outcomes, proline-derived HAAs failed to undergo the carbonyl shift, and Boc-protected or *N*-free HAAs returned the corresponding Weinreb amides **1p** and **1q** rather than rearranged products **3p** and **3q**. Together these trends define the practical envelope: the method is most general for benzo­[*d*]­thiazole HAAs and a wide set of hydroxylamines, with explicit limitations for sterically encumbered ester motifs, proline systems, and Boc protection. For all reactions furnishing aza-β-lactams **3**, the products shown represent the dominant reaction outcomes. Residual material is predominantly unreacted starting HAA **4**, while carbamate side products analogous to **6a** were observed only in isolated cases.

**Figure 3:**
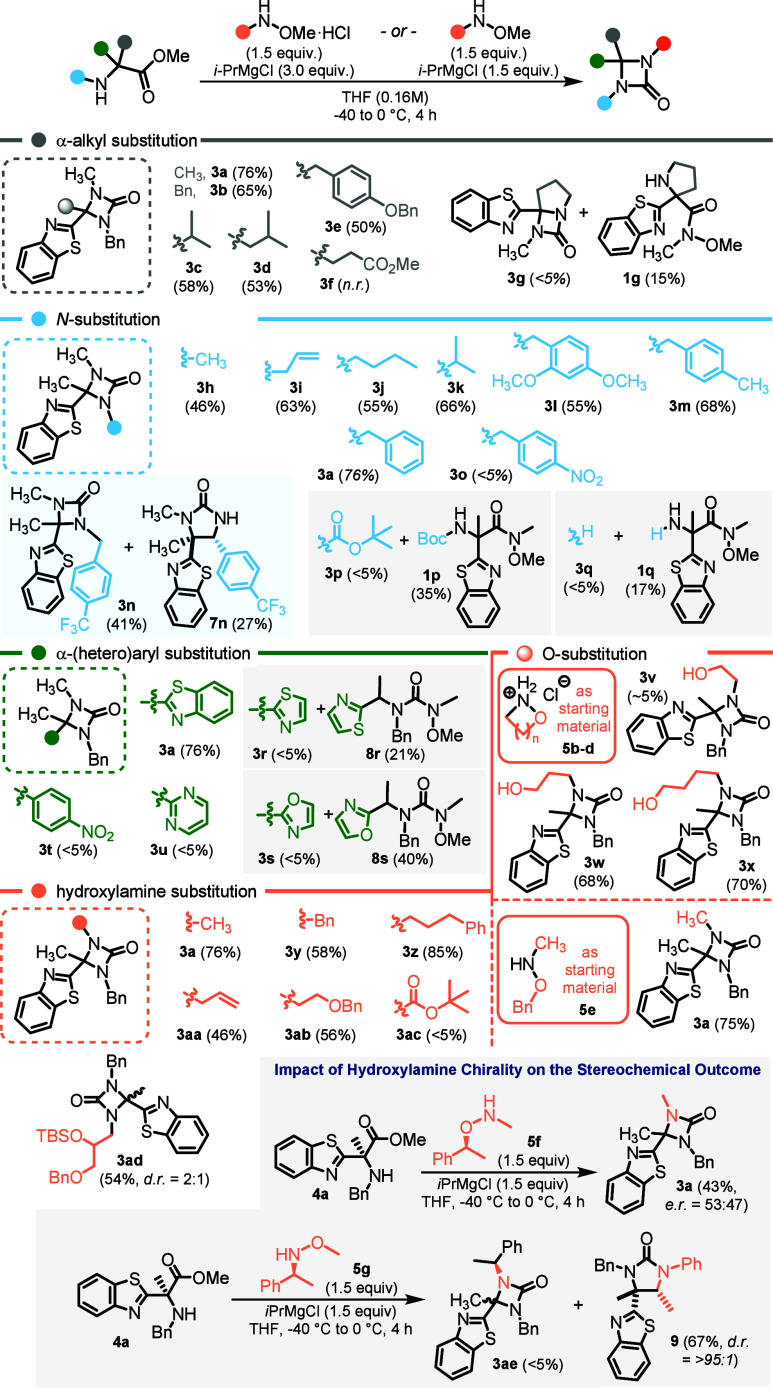
Scope and limitations of the transformation of HAAs **4** into aza-β-lactams **3**. Reactions were performed on a 0.20 mmol scale. *d.r.* values were determined by ^1^H NMR analysis of the crude reaction mixture; yields refer to isolated products. Hydroxylamines **5** were used either as free bases or as their corresponding hydrochloride salts.

As a consequence of the mechanistic features described above, electronic effects on the HAA *N*-substituent modulate chemoselectivity. Simple alkyl and benzyl groups work well, but electron-poor benzylic substituents (e.g., *p*-CF_3_) attenuate formation of the aza-β-lactam and promote the five-membered urea pathway; in a representative case, the *p*-CF_3_ substrate furnished a 41% yield of aza-β-lactam **3n** alongside 27% of the corresponding urea **7n**. This tunable divergence can be exploited deliberately – either by driving ring expansion from isolated aza-β-lactams under strong base or by accessing ureas directly from HAAs in one pot by increasing the *i*PrMgCl loading (Figure 4C and 4D).

**Figure 4:**
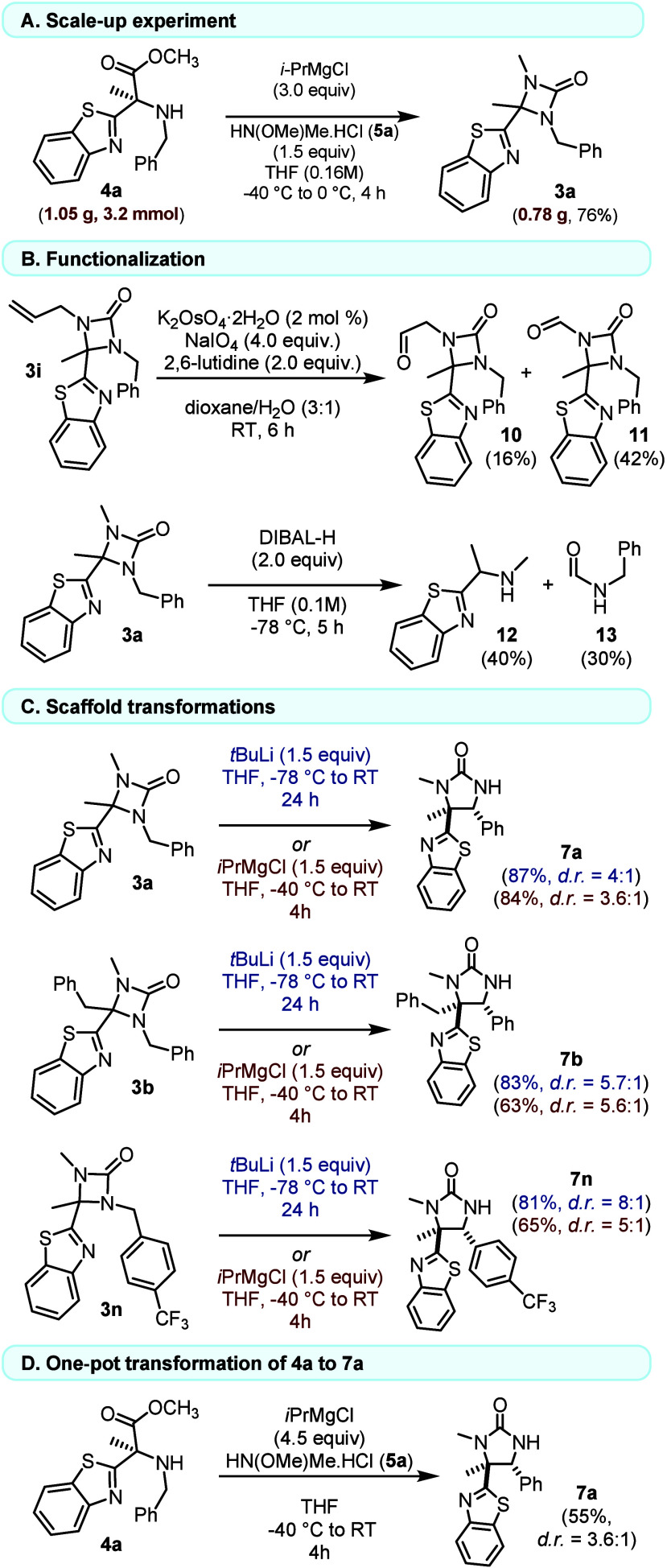
Downstream transformations. (A) Scale-up experiment. (B) Side-chain functionalization of **3**. (C) Base-induced ring expansion of **3** to five-membered ureas **7**. (D) One-pot conversion of HAA **4a** to **7a**. Isolated yields *d.r.* determined by ^1^H NMR analysis of the crude reaction mixture.

From an operational standpoint the method is straightforward and scales to gram quantities without erosion of yield (Figure 4A). The representative product **3a** is robust to hydrolytic conditions and to Brønsted/Lewis acids (Figure S2), enabling practical handling and storage, and it serves as a versatile platform for late-stage diversification: oxidative cleavage of a side-chain olefin delivers useful aldehydes **10** and **11** (Figure 4B); DIBAL-H induces ring opening to an amine **12**/formamide **13** pair (Figure 4B); and base-induced ring expansion provides access to five-membered ureas, which can alternatively be obtained directly from HAAs by increasing the *i*PrMgCl equivalents, creating a predictable divergence within a single operation (Figure 4C and 4D).

Stereochemical probes indicate that the stereochemistry-defining event occurs after equilibration of upstream intermediates. Regardless of the enantiopurity of the HAAs, aza-β-lactams are formed racemic. Chiral hydroxylamines behave similarly regardless of whether the stereocenter is adjacent to oxygen or nitrogen. When located next to oxygen, the reaction affords **3a** in 53:47 *e.r.* In contrast, an *N*-adjacent stereocenter diverts the reaction toward the five-membered urea **9**, obtained as a single diastereomer (>99:1 *d.r.*) yet racemic at the carbon centers. The modest sensitivity to hydroxylamine *O*-substituent bulk indicates steric control over formation of the aziridinone-like intermediate. This interpretation is supported by suppressed formation of **3a** and increased carbamate **6a** formation from *tert*-butyl ester substrates. These observations suggest that C→N migration, rather than external capture, is rate-determining and are consistent with the observed racemization (Figure 5A).

**Figure 5:**
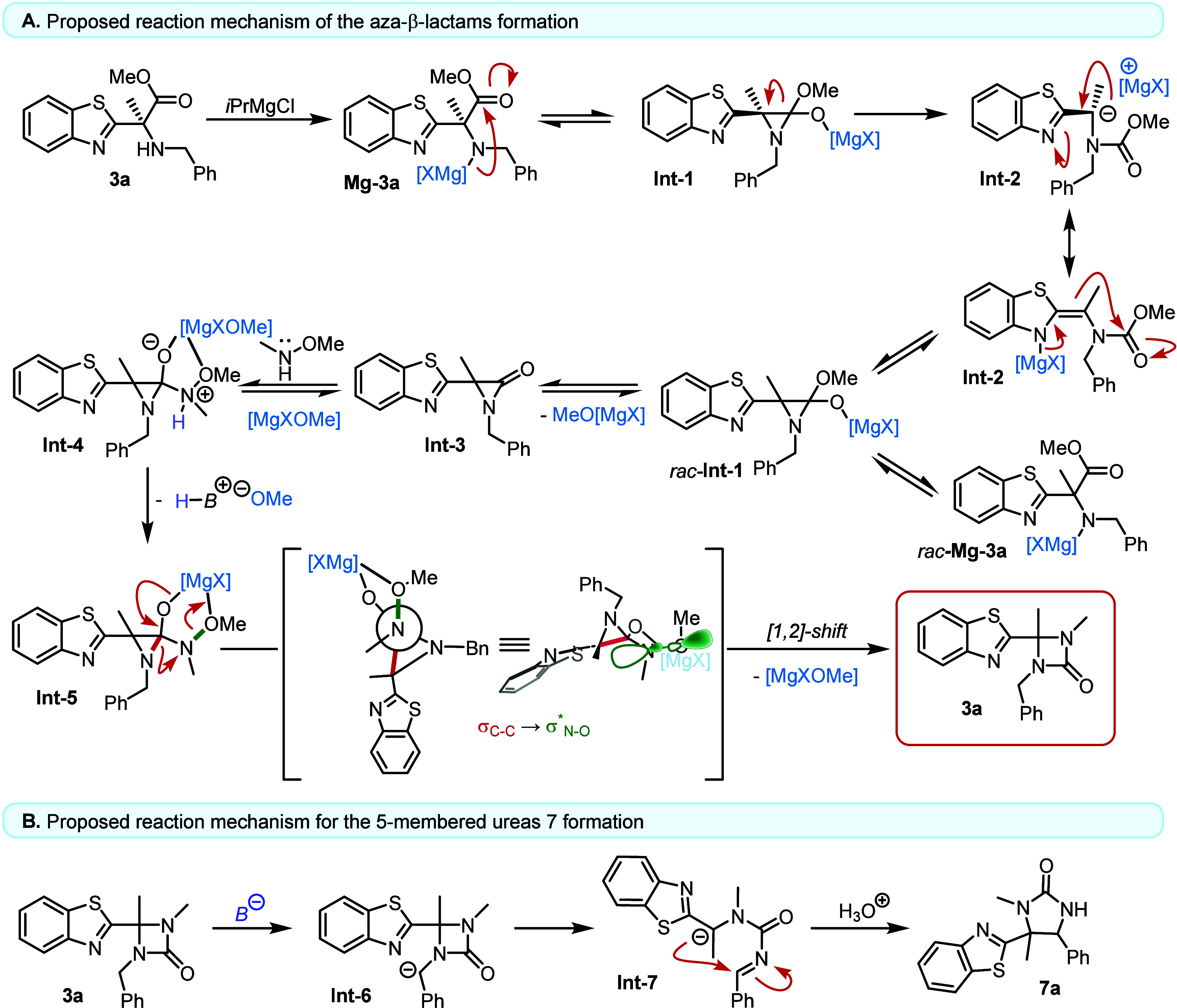
(A) Proposed reaction mechanism leading to the formation of aza-β-lactam **3a**. (B) Proposed pathway for the base-induced formation of five-membered urea heterocycles **7a**.

Mechanistic experiments support the proposed deprotonation (**Mg-3a**)→ intramolecular addition → reversible carbanion/carbamate manifold → aziridinone-like intermediate (**Int-3**) → hydroxylamine capture → [1,2]-heteroaryl migration sequence. Most notably, a carbamate-type intermediate (**6a**) was isolated and shown to re-enter the productive pathway to give **3a** under the standard conditions, directly supporting a reversible stage that precedes the migration/cyclization event and explains the observed racemization from enantioenriched HAAs (Figure S3). The divergence to five-membered ureas is rationalized by base-induced ring expansion of *N*-benzylic aza-β-lactams and manifests cleanly under stronger basic conditions, aligning the optimization data with downstream selectivity (Figure 5B).

Additional mechanistic considerations further clarify the origin of the carbamate byproduct **6a** and the uniquely privileged role of the benzo­[*d*]­thiazole (BT) heterocycle. We attribute the formation of **6a** to the increased thermodynamic stability of intermediate **Int-2** under the reaction conditions. Protonation of this stabilized intermediate leads to irreversible trapping as carbamate **6a**, thereby diminishing the population of the key aziridinone-like intermediate **Int-3**, which is required for productive interception by hydroxylamine to form aza-β-lactam **3a**.

Consistent with this interpretation, a decisive factor for successful progression along the productive pathway appears to be the nature of the heteroaryl substituent. Among all heterocycles examined (Figure 3), only the BT group enables the transformation of **Int-3** into **Int-5**, an intermediate competent for the subsequent [1,2]-heteroaryl migration. Substrates bearing alternative heterocycles either fail to react or diverge toward the formation of five-membered ureas **8r** and **8s**, with no detectable formation of the corresponding aza-β-lactams. This divergence is attributed either to excessive stabilization of **Int-1**, which prevents formation of **Int-2**. In oxazole- and thiazole-derived substrates, the reaction instead progresses to **Int-3**, followed by interception with **5a** and collapse of **Int-4** into thermodynamically favored ureas **8**.

Deuterium-labeling experiments further support the stability of upstream intermediates prior to the migration step. When the reaction was conducted in THF-*d*_6_ and quenched with MeOD followed by D_2_O, no deuterium incorporation was detected in either aza-β-lactam **3a** or carbamate **6a**. These results indicate that protonation events associated with carbamate formation occur prior to the quench and that no reversible incorporation of deuterium takes place under the reaction conditions.

In summary, a tandem C→N carbonyl shift and [1,2]-heteroaryl migration converts HAAs and hydroxylamines to aza-β-lactams in a single, scalable operation orchestrated by *i*PrMgCl at low temperature, delivering strained *N*,*O-*urea frameworks that are both stable and readily diversified. The transformation tolerates diverse Cα-alkyl substitution and a wide range of *O*/*N-*substituted hydroxylamines and encodes a controllable divergence to five-membered ureas under strongly basic conditions or with electron-poor *N-*substituents. Isolation and productive re-entry of a carbamate intermediate and stereochemical probes together support a reversible carbanion/carbamate stage upstream of migration, rationalizing racemic outcomes and guiding future designs. The present generality is highest with benzo­[*d*]­thiazole-derived HAAs; ongoing efforts are directed toward expanding the heteroaryl component and translating the mechanistic picture into asymmetric variants. Together, these results establish a concise and mechanistically defined entry to aza-β-lactams that should be broadly useful for strained heterocycle design and further method development.

## Supplementary Material



## Data Availability

The data underlying this study are available in the published article, in its Supporting Information, and openly available in Zenodo at 10.5281/zenodo.18695962.

## Notes

An expanded discussion of project motivation, full reaction optimization data, product stability studies, control experiments relevant to the mechanistic proposal, detailed experimental procedures, complete compound characterization, single-crystal X-ray diffraction data, copies of all ^1^H, ^13^C­{^1^H, ^19^F} and ^19^F spectra, and HPLC chromatograms (PDF)

## References

[1] Vitaku E., Smith D. T., Njardarson J. T. (2014). Analysis of the Structural Diversity,
Substitution Patterns, and Frequency of Nitrogen Heterocycles among
U.S. FDA Approved Pharmaceuticals: Miniperspective. J. Med. Chem..

[2] Heravi M. M., Zadsirjan V. (2020). Prescribed
Drugs Containing Nitrogen Heterocycles:
An Overview. RSC Adv..

[3] Marshall C. M., Federice J. G., Bell C. N., Cox P. B., Njardarson J. T. (2024). An Update
on the Nitrogen Heterocycle Compositions and Properties of U.S. FDA-Approved
Pharmaceuticals (2013–2023). J. Med.
Chem..

[4] Murray C. J. L., Ikuta K. S., Sharara F., Swetschinski L., Robles Aguilar G., Gray A., Han C., Bisignano C., Rao P., Wool E., Johnson S. C., Browne A. J., Chipeta M. G., Fell F., Hackett S., Haines-Woodhouse G., Kashef Hamadani B. H., Kumaran E. A. P., McManigal B., Achalapong S., Agarwal R., Akech S., Albertson S., Amuasi J., Andrews J., Aravkin A., Ashley E., Babin F.-X., Bailey F., Baker S., Basnyat B., Bekker A., Bender R., Berkley J. A., Bethou A., Bielicki J., Boonkasidecha S., Bukosia J., Carvalheiro C., Castañeda-Orjuela C., Chansamouth V., Chaurasia S., Chiurchiù S., Chowdhury F., Clotaire Donatien R., Cook A. J., Cooper B., Cressey T. R., Criollo-Mora E., Cunningham M., Darboe S., Day N. P. J., De Luca M., Dokova K., Dramowski A., Dunachie S. J., Duong Bich T., Eckmanns T., Eibach D., Emami A., Feasey N., Fisher-Pearson N., Forrest K., Garcia C., Garrett D., Gastmeier P., Giref A. Z., Greer R. C., Gupta V., Haller S., Haselbeck A., Hay S. I., Holm M., Hopkins S., Hsia Y., Iregbu K. C., Jacobs J., Jarovsky D., Javanmardi F., Jenney A. W. J., Khorana M., Khusuwan S., Kissoon N., Kobeissi E., Kostyanev T., Krapp F., Krumkamp R., Kumar A., Kyu H. H., Lim C., Lim K., Limmathurotsakul D., Loftus M. J., Lunn M., Ma J., Manoharan A., Marks F., May J., Mayxay M., Mturi N., Munera-Huertas T., Musicha P., Musila L. A., Mussi-Pinhata M. M., Naidu R. N., Nakamura T., Nanavati R., Nangia S., Newton P., Ngoun C., Novotney A., Nwakanma D., Obiero C. W., Ochoa T. J., Olivas-Martinez A., Olliaro P., Ooko E., Ortiz-Brizuela E., Ounchanum P., Pak G. D., Paredes J. L., Peleg A. Y., Perrone C., Phe T., Phommasone K., Plakkal N., Ponce-de-Leon A., Raad M., Ramdin T., Rattanavong S., Riddell A., Roberts T., Robotham J. V., Roca A., Rosenthal V. D., Rudd K. E., Russell N., Sader H. S., Saengchan W., Schnall J., Scott J. A. G., Seekaew S., Sharland M., Shivamallappa M., Sifuentes-Osornio J., Simpson A. J., Steenkeste N., Stewardson A. J., Stoeva T., Tasak N., Thaiprakong A., Thwaites G., Tigoi C., Turner C., Turner P., Van Doorn H. R., Velaphi S., Vongpradith A., Vongsouvath M., Vu H., Walsh T., Walson J. L., Waner S., Wangrangsimakul T., Wannapinij P., Wozniak T., Young Sharma T. E. M.
W., Yu K. C., Zheng P., Sartorius B., Lopez A. D., Stergachis A., Moore C., Dolecek C., Naghavi M. (2022). Global Burden of Bacterial
Antimicrobial Resistance in 2019: A Systematic Analysis. Lancet.

[5] Tooke C. L., Hinchliffe P., Bragginton E. C., Colenso C. K., Hirvonen V. H. A., Takebayashi Y., Spencer J. (2019). *β*-Lactamases
and *β*-Lactamase Inhibitors in the 21st Century. J. Mol. Biol..

[6] Hammoudi
Halat D., Ayoub Moubareck C. (2020). The Current Burden of Carbapenemases:
Review of Significant Properties and Dissemination among Gram-Negative
Bacteria. Antibiotics.

[7] Bahr G., González L. J., Vila A. J. (2021). Metallo-*β*-Lactamases
in the Age of Multidrug Resistance: From Structure and Mechanism to
Evolution, Dissemination, and Inhibitor Design. Chem. Rev..

[8] Nangia A. (1991). AM1 Calculations on a Series of Bicyclic
Azetidin-2-Ones
and Bicyclic 1,3-Diazetidin-2-Ones: Investigations towards Putative *β*-Lactamase Inactivators. J.
Mol. Struct. THEOCHEM.

[9] Richter R. (1969). Additionen von Arylisocyanaten an Benzaldehyd- und
Benzophenon-anile. Chem. Ber..

[10] Nishio T., Kato A., Kashima C., Omote Y. (1980). Photochemical Electrocyclization of 1,4,6-Trisubstituted Pyrimidin-2-Ones
to 2-Oxo-1,3-Diazabicyclo[2.2.0]­Hex-5-Enes. J. Chem. Soc. Perkin 1.

[11] Kristek J., Pospíšil J. (2025). 3-Diazetidin-2-Ones:
Synthetic Approaches
and Therapeutic Promise within the Aza-*β*-Lactam
Family. Eur. J. Org. Chem..

[12] Kristek J., Chrenko D., Desmedt E., Zálešák F., Št’astná N., De Vleeschouwer F., Pospíšil J. (2026). Stereodivergent Truce-Smiles
Rearrangement
Enables Enantioselective Access to α-Heteroaryl Quaternary Amino
Acid Derivatives. ChemRxiv.

