# Supplementary material for: Base-Promoted C→N Carbonyl Shift Enables One-Step Access to aza-β-Lactams from α‑Heteroaryl-α-amino Esters
Source: Org Lett. 2026 May 11;28(20):6318–23. doi: 10.1021/acs.orglett.6c01451 (PMC13200243; doi:10.1021/acs.orglett.6c01451)

Supporting Information

Base-Promoted C→N Carbonyl Shift Enables One-Step Access to Aza-β-Lactams from *α*-Heteroaryl-*α*-Amino Esters

Jozef Kristek,[a] Nikola Šťastná,[a] and Jiří Pospíšil*[a,b,c]

[a)] Department of Organic Chemistry, Faculty of Science, Palacky University, tř. 17. listopadu 1192/12, Olomouc CZ-771 46, Czech Republic.

[b)] Department of Chemical Biology, Faculty of Science, Palacky University, Šlechtitelů 27, Olomouc CZ-78371, Czech Republic.

[c)] Laboratory of Growth Regulators, Institute of Experimental Botany of the Czech Academy of Sciences, and Faculty of Science, Palacky University, Šlechtitelů 27, Olomouc CZ-78371, Czech Republic.

Email: j.pospisil@upol.cz

Table of content

1 Motivation of the project 3

2 Optimization Tables 4

3 Structure Determination 7

4 Stability Experiments 9

5 Reaction Mechanism Determination – Control Experiments 10

5.1 Additional mechanistic considerations 12

6 Unsuccessful substrates 14

7 General Information 15

7.1 Starting Material Preparation 17

7.1.1 Synthesis of α-heteroaryl-α-amino esters and amides 17

7.1.2 Synthesis of Hydroxylamines **5** 32

7.2 Substrate Scope of Synthesis of 1,3-diazetidin-2-one (aza-β-lactam) 49

7.2.1 Characterization data 50

7.3 Additional Experiments and Post-modifications 65

7.3.1 Stereocenter adjacent to oxygen 65

7.3.2 Stereocenter adjacent to nitrogen 65

7.4 Control and Mechanistic Experiments 71

7.4.1 Reaction with **S1** under standard reaction conditions 71

7.4.2 Preparing and isolating the carbamate **6a** 71

7.4.3 Reaction of carbamate **6a** under standard conditions 72

7.4.4 Transformation of carbamate **6a** to amine **S3** 72

7.4.5 Basic hydrolysis of **4a** to amine **S3** 73

7.4.6 Control reaction with Weinreb amide **1a** 73

7.4.7 Reaction of **4a** with *N*-methyl-*O*-(methylsulfonyl)hydroxylamine **5n** 74

7.4.8 Reaction of **4a** with *O*-(*tert*-butyl)-*N*-methylhydroxylamine hydrochloride **5o** 74

8 References 75

9 Copy of ^{1}H, $^{13}C\{^{1}H\}$NMR, and ^{19}F NMR Spectra 78

1 Motivation of the project

Nitrogen-containing heterocycles underpin a significant fraction of modern pharmaceuticals, with β-lactams occupying a uniquely influential position due to their combination of ring strain, electrophilicity, and highly selective covalent reactivity with serine hydrolases.[1,2] Despite the erosion of antibiotic efficacy driven by the widespread emergence of β-lactamases, β-lactam frameworks continue to inspire new generations of mechanism-based inhibitors designed to intercept these resistance enzymes.[3,4] Re-engineering the four-membered β-lactam architecture by replacing the carbonyl-adjacent methylene with a nitrogen affords 1,3-diazetidin-2-ones (aza-β-lactams) – a class of compact N,O-urea heterocycles with distinct electronic, structural, and biochemical behavior (Fig.1A).[5–8]

Computational analyses suggest that aza-β-lactams diverge substantially from classical β-lactams in their electronic structure and predicted biochemical behavior. In silico models indicate that incorporation of a second ring nitrogen redistributes strain and lowers carbonyl electrophilicity, consistent with enhanced hydrolytic stability relative to β-lactams.[5,6,8,9] Simulations further predict that aza-β-lactams preferentially form carbamate-type acyl adducts with serine nucleophiles, leading to slower deacylation and extended residence times, features commonly associated with β-lactamase inhibitor scaffolds. Finally, electronic mapping highlights orthogonal vectors for substitution and identifies unique reaction pathways – including selective ring expansion and nucleophilic openings – that are not accessible to β-lactams.[5,6,8] Although experimental validation remains limited, these *in silico* insights collectively underscore the potential of aza-β-lactams as distinct and versatile small-ring heterocycles.[5]

Despite these advantages and their demonstrated potential in enzyme inhibition, covalent probe development, and diversity-oriented synthesis, the exploration of aza-β-lactams has been limited primarily by the lack of general and operationally simple synthetic routes (Fig.1B). Existing approaches rely predominantly on [2+2] cycloadditions of imines with isocyanates or photochemical rearrangements of pyrimidinones, methodologies that often suffer from restricted substrate scope, competing side reactions, specialized equipment, or limited tolerance toward polar or electronically diverse functional groups.[5,7,10–12] Many of these routes also generate products with modest stability or require multistep precursor synthesis, diminishing their practicality in discovery-driven contexts.[5,7,12] Consequently, there is a persistent need for concise, modular, and functionally tolerant strategies that transform readily accessible building blocks into structurally diverse aza-β-lactam frameworks.

α-Heteroaryl-α-amino esters (HAAs)[13] represent an attractive platform in this regard: they are easily accessed, structurally diverse, and exhibit rich reactivity under basic conditions. In the course of investigating their behavior, we observed that HAA-derived Weinreb amides undergo spontaneous C→N carbonyl migration, forming aza-β-lactams rather than the anticipated 1,2-diazetidin-3-ones.(Fig.2A) This serendipitous transformation suggested the possibility of a direct, one-step synthesis from the amino ester itself, provided that the interplay of base strength, nucleophile identity, and temperature could be controlled. Building on this insight, we sought to develop a tandem C→N carbonyl shift–amidation–cyclization that would deliver aza-β-lactams broadly and predictably from simple HAA precursors.

2 Optimization Tables

The optimization data in **Table S1** reveal a clear relationship between base identity, reaction temperature, and product distribution in the transformation of HAA ester **4a** to the desired aza-β-lactam **3a**. Strong organolithium bases such as MeLi, *s*BuLi, and *t*BuLi (entries 1, 3–4), as well as LiHMDS (entry 2), gave only traces of the target **3a** and instead funneled material predominantly into the carbamate-type intermediate **6a**. The formation of **6a** in 45–70% yield under these conditions indicates that highly basic and non-chelating reagents favor deprotonation and carbonyl transfer, populating the carbanion/carbamate manifold rather than enabling the intramolecular addition–migration sequence required to form **3a**. LDA behaves similarly (entry 5), promoting **6a** (67%) with almost no productive rearrangement. In contrast, tertiary amine bases such as DABCO and DBU (entries 6–7) fail to initiate the carbonyl shift entirely and instead return the ester **4a** in near-quantitative yield, establishing that nucleophilic metalation, not simple deprotonation, is necessary to trigger the rearrangement.

A qualitative shift occurs with Grignard reagents: *i*PrMgCl reliably engages the desired pathway, and its reactivity is strongly temperature-dependent. At very low initiation temperatures (−78 °C, entry 8) **3a** is obtained in only modest yield (62%), accompanied by substantial amounts of **6a**. As the starting temperature is raised progressively to −60, −50, and −40 °C (entries 9–11), the yield of **3a** steadily increases while **6a** diminishes, culminating in 84% **3a** (76% isolated) and only 14% **6a** at **−40 °C → 0 °C.** This identifies controlled warm-up from −40 °C as optimal for channeling the system through the intramolecular addition and [1,2]-heteroaryl migration steps rather than into the carbamate trap. Starting the reaction too warm (−30 °C, entry 12) compromises selectivity and results in lower **3a** (53%), indicating that a sufficiently low activation barrier must be crossed under controlled conditions to avoid unproductive pathways.

The nature of the Grignard reagent is also important. Although *n*BuMgCl (entry 13) delivers **3a** in good yield (75%), it remains inferior to *i*PrMgCl at −40 °C, suggesting that reagent aggregation or steric/electronic matching influences the balance between reversible intermediate formation and productive migration. Solvent effects reinforce this interpretation: switching from THF to Et_2O or DME (entries 14–15) reduces the yield of **3a** and increases **6a**, consistent with weaker coordination and altered Grignard aggregation state disfavoring the rearrangement.

Stoichiometry further modulates pathway selection. Reducing *i*PrMgCl to 2.2 equiv. (entry 17) dramatically increases **6a** (62%) at the expense of **3a** (30%), showing that insufficient metalation leaves the reaction trapped in the carbamate manifold. Increasing the base slightly (entry 16) restores some formation of **3a**, but not to the level achieved under standard conditions, highlighting that **both** stoichiometry and temperature control are critical. The addition of DIPEA (entry 18) has minimal influence relative to the matched conditions (entry 19), confirming that external amine buffering does not alter the reaction trajectory once the Grignard reagent controls the metalation equilibrium.

Chloride additives exert strong and generally deleterious effects. LiCl or $MgCl_2$ (entries 20–24) heavily bias the reaction toward carbamate formation, with **6a** reaching 65–77% at low additive loadings. At higher additive concentrations this effect becomes partially moderated, but overall, the data indicate that halide salts perturb Grignard aggregation and suppress formation of the reactive intermediate needed for the rearrangement. These observations support the conclusion that chloride perturbs the metalated HAA equilibrium in favor of the carbanion/carbamate pair at the expense of the rearrangement-prone species.

Finally, increased loading of *i*PrMgCl induces a distinct mechanistic divergence. At 4.0 equiv. (entry 25), 3a formation drops and **6a** nearly disappears, signaling onset of over-activation. At 4.5 equiv. (entry 26), the pathway bypasses the four-membered ring entirely and furnishes the urea **7a** in **89%** NMR yield (84% isolated)**.** This establishes a controllable and predictable switch between the four-membered aza-β-lactam and the five-membered urea product simply by adjusting base loading. The $AlMe_3$ experiment (entry 27) was carried out to evaluate the possibility of the Lewis acid-catalyzed mechanism: in CH_2Cl_2 the reaction provides only ~5% **3a** along with substantial amounts of **6a** and **1a**, confirming that Lewis-acidic, non-coordinating conditions fail to support the rearrangement sequence.

Taken together, these data show that *i*PrMgCl in THF at −40 °C → 0 °C with 3.0 equiv. of base (1.5 equiv. of *i*PrMgCl is used to transform the hydroxylamine **5a** to a free base) is uniquely suited to balance metalation, intramolecular attack, reversible intermediate equilibration, and migration, enabling efficient formation of aza-β-lactam **3a** while minimizing the carbamate intermediate **6a**. Lower temperatures, halide additives, or sub-stoichiometric base shift the system toward **6a**, whereas strongly basic conditions or elevated Grignard loading redirect the pathway toward **urea 7a**, providing a tunable and mechanistically coherent map of the reaction landscape.

Table S1: Optimization of the HAA ester **4a** transformation to aza-β-lactam **3a**

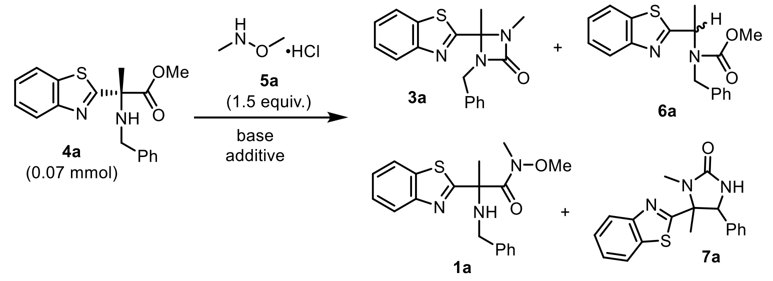

Entry	*Base (equiv.)*	*Additive (equiv.)*	*Conditions*	***3a** (%)[a]*	***6a** (%)[a]*	***1a** (%)[a]*	***7a** (%)[a]*
1	MeLi (3.0 equiv.)	–	THF, -78°C to 0°C, 4h	~5	~5	–	–
2	LiHMDS (3.0 equiv.)	–	THF, -78°C to 0°C, 4h	<5	45	–	–
3	*s*BuLi (3.0 equiv.)	–	THF, -78°C to 0°C, 4h	5	63	–	–
4	*t*BuLi (3.0 equiv.)	–	THF, -78°C to 0°C, 4h	9	70	–	–
5	LDA (3.0 equiv.)	–	THF, -78°C to 0°C, 4h	–	67	<5	–
6[c]	DABCO (3.0 equiv.)	–	THF, -78°C to 0°C, 4h	–	–	–	–
7[d]	DBU (3.0 equiv.)	–	THF, -78°C to 0°C, 4h	–	–	–	–
8	*i*PrMgCl (3.0 equiv.)	–	THF, -78°C to 0°C, 4h	62	35	–	–
9	*i*PrMgCl (3.0 equiv.)	–	THF, -60°C to 0°C, 4h	68	30	–	–
10	*i*PrMgCl (3.0 equiv.)	–	THF, -50°C to 0°C, 4h	71	25	–	–
11	*i*PrMgCl (3.0 equiv.)	–	THF, -40°C to 0°C, 4h	84 (76)[b]	14 (10)[b]	–	–

Entry	*Base (equiv.)*	*Additive (equiv.)*	*Conditions*	***3a*** *(%)*[a)]	***6a*** *(%)*[a)]	***1a*** *(%)*[a)]	***7a*** *(%)*[a)]
12	*i*PrMgCl (3.0 equiv.)	–	THF, -30°C to 0°C, 4h	53	<5	–	–
13	*n*BuMgCl (3.0 equiv.)	–	THF, -40°C to 0°C, 4h	75	12	–	–
14	*i*PrMgCl (3.0 equiv.)	–	Et_2O, -40°C to 0°C, 4h	65	21	–	–
15	*i*PrMgCl (3.0 equiv.)	–	DME, -40°C to 0°C, 4h	45	29	–	–
16	*i*PrMgCl (3.2 equiv.)	–	**5a** (1.1 equiv.), THF, -40°C to 0°C, 4h	52	11	–	–
17	*i*PrMgCl (2.2 equiv.)	–	**5a** (1.1 equiv.), THF, -40°C to 0°C, 4h	30	62	–	–
18	*i*PrMgCl (2.2 equiv.)	DIPEA (2.0 equiv.)	**5a** (1.1 equiv.), THF, -40°C to 0°C, 4h	62	3	–	–
19	*i*PrMgCl (2.2 equiv.)	–	**5a** (1.1 equiv.), THF, -40°C to 0°C, 4h	62	3	–	–
20	*i*PrMgCl (3.0 equiv.)	LiCl (1.5 equiv.)	THF, -40°C to 0°C, 4h	35	65	–	–
21	*i*PrMgCl (3.0 equiv.)	LiCl (3.0 equiv.)	THF, -40°C to 0°C, 4h	62	3	–	–
22	*i*PrMgCl (4.5 equiv.)	LiCl (3.0 equiv.)	THF, -40°C to 0°C, 4h	–	–	–	–
23	*i*PrMgCl (3.0 equiv.)	$MgCl_2$ (1.5 equiv.)	THF, -40°C to 0°C, 4h	11	77	–	–
24	*i*PrMgCl (3.0 equiv.)	$MgCl_2$ (3.0 equiv.)	THF, -40°C to 0°C, 4h	58	37	–	–
25	*i*PrMgCl (4.0 equiv.)	–	THF, -40°C to 0°C, 4h	41	<5	–	–
26	*i*PrMgCl (4.5 equiv.)	–	THF, -40°C to 0°C, 4h	–	–	–	89 (84)[b)]
27	–	$AlMe_3$ (3.0 equiv.)	CH_2Cl_2, -78°C to 0°C, 4h	~5	35	25	–

[a)] The yield was determined by ^{1}H NMR analysis of the crude reaction mixture with trichloroethylene (TCE, 0.05 mmol) as internal standard. [b)] Isolated yield. [c)] Starting material **4a** recuperated in 87% yield. [d)] Starting material **4a** recuperated in 99% yield.

3 Structure Determination

The structure determination of compounds **3**, **7**, and **9** proved challenging. Only limited literature precedent exists for these scaffold types, and the molecules contain multiple quaternary carbon stereocenters embedded within highly congested small rings**,** which complicates unambiguous assignment by routine 1D and 2D NMR spectroscopy. Obtaining high-quality single crystals for X-ray diffraction was equally difficult. Despite extensive crystallization attempts, most samples provided only low- to medium-quality crystals**,** insufficient for definitive structural confirmation. The only structurally comparable examples we identified in the literature were those related to compound **7** (see reference[14]).

Ultimately, we succeeded in obtaining high-quality, publication-grade X-ray structures for compounds **3a**, **3b** and **7b**. The enantioenrichment of **3a** could be verified independently by chiral HPLC, giving an *e.r.* = 53:47 (see compound characterization for details).

For compound **9**, structure assignment likewise relied primarily on X-ray analysis. Although no high-quality crystal could be obtained despite substantial effort, the lower-quality dataset (corresponding CIF file is available via Zenodo – doi: 10.5281/zenodo.18695962) combined with comparative NMR analysis supports the proposed structure. In particular, the NMR features of **9** show close correspondence with the only structurally similar compound we found in the literature (compound **37** in reference[15]), further reinforcing the correctness of our assignment.

Crystal Growth

Single crystals suitable for X-ray diffraction analysis were obtained by slow evaporation from a saturated solution of the corresponding compound in a mixed solvent system of chloroform and *n*-heptane at ambient temperature. The solution was placed in a small vial and allowed to evaporate slowly under partially closed conditions to afford colorless crystals over several days.

Data

The X-ray diffraction data for **3a, 3b**, **7b** and **9** were collected on a Rigaku XtaLAB Synergy-I diffractometer with a HyPix3000 hybrid pixel array detector and micro-focused PhotonJet-I X-ray source (Cu Kα). The analytical absorption corrections were applied using the program CrysAlisPro 1.171.40.82a [a]. The crystal structure was solved using SHELXT program [b] and refined by the full matrix least-squares procedure using Shelxl software (ver. 2018/3) [c] in OLEX2 (version 1.3).[d] All the non-hydrogen atoms were refined anisotropically. The hydrogen atoms were located from the difference maps (hydrogen atom of the secondary amine group) or placed to calculated positions and they were included in the riding model approximation with U_{iso} = 1.2Ueq(C) or 1.5U_{eq} (CH_3). The resulting crystal structure was deposited in the Cambridge Structural Database.

[a] CrysAlisPro 1.171.40.82a, Rigaku Oxford Diffraction, 2020
[b] G. M. Sheldrick, Acta Crystallogr., Sect. A: Found. Adv., 2015, 71, 3.
[c] G. M. Sheldrick, Acta Crystallogr., Sect. A: Found. Adv., 2015,71, 3.
[d] O. Dolomanov, L. J. Bourhis, R. Gildea, J. A. Howard andH. Puschmann, J. Appl. Crystallogr., 2009, 42, 339.

Table S2. . Crystal data and structure refinement for **3a**, **3b** and **7b**.

	**3a**	**3b**	**7b**
Formula	$C_{18}H_{17}N_3OS$	$C_{24}H_{21}N_3OS$	$C_{25}H_{22}Cl_3N_3OS$
Formula weight	323.42	399.50	518.86
Crystal system	monoclinic	monoclinic	triclinic
Space group	$P2_1/c$	$P2_1/c$	*P-1*
T/K	298(2)	298(2)	100.0(1)
Cell parameters			
a/Å	16.1201(3)	12.62692(17)	6.71600(10)
b/Å	6.1642(1)	14.50337(19)	12.1154(2)
c/Å	17.6475(4)	11.44949(15)	15.8930(3)
α/deg	90	90	99.771(2)
β/deg	109.308(2)	97.8518(13)	99.0070(10)
γ/deg	90	90	102.2900(10)
V/Å^3	1654.96(6)	2077.12(5)	1220.02(4)
Z	4	4	2
Density, Dc/g cm^{-3}	1.298	1.278	1.412
Abs. coefficient/mm^{-1}	1.794	1.534	4.389
Reflection collected	8207	12414	11463
R_{int}	0.0216	0.0220	0.0177
Data/restraints/param	3086/0/210	3763/0/264	4538/16/325
$R1^a$, wR_2^b (all data)	0.0537/0.1484	0.0354/0.0930	0.0307/0.0739
R_1^a, wR_2^b [$I > 2\sigma(I)$]	0.0494/0.1463	0.0319/0.0903	0.0292/0.0729
Goodnes of fit	1.071	1.073	1.021
CSD number			

^{a} $R_1 = \sum(|F_o| - |F_c|)/\sum|F_o|$, ^{b} $wR^2 = \{\sum[w(F^2_o - F^2_c)^2]/\sum[w(F^2_o)^2]\}^{1/2}$

Figure S1 Perspective ORTEP-style representations (30% probability ellipsoids) of the molecular structures of compounds **3a**, **3b**, and **7b**. Color code: carbon (light brown), hydrogen (white), nitrogen (light blue), oxygen (red) and sulfur (yellow).

3a **3b** **7b**

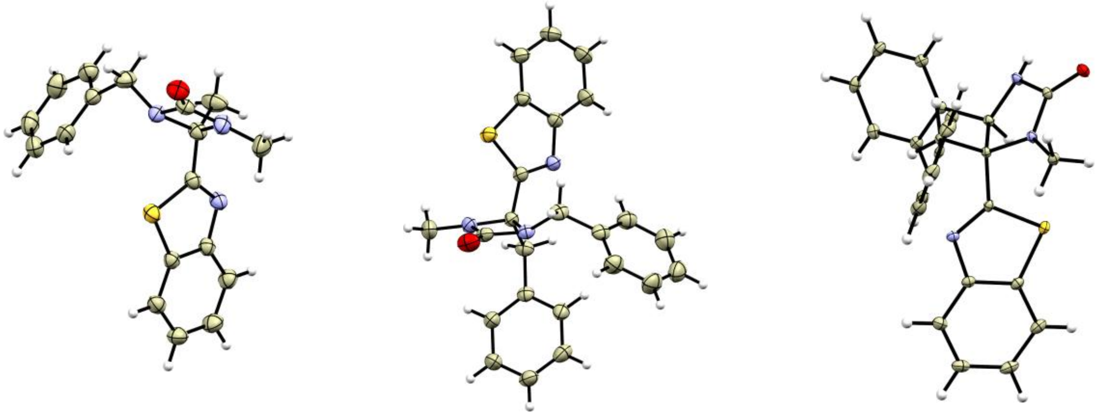

4 Stability Experiments

Given that densely substituted aza-b-lactams **3** represent a relatively new and only sparsely characterized structural class, we evaluated the stability of **3a** under a range of hydrolytic conditions as well as in the presence of selected Lewis and Brønsted acids. (Stability under strongly basic conditions was not examined, as such conditions are inherently part of the reaction protocol.) As shown in Fig. S2, **3a** remained unchanged even after prolonged exposure to all tested environments, demonstrating its notable robustness.

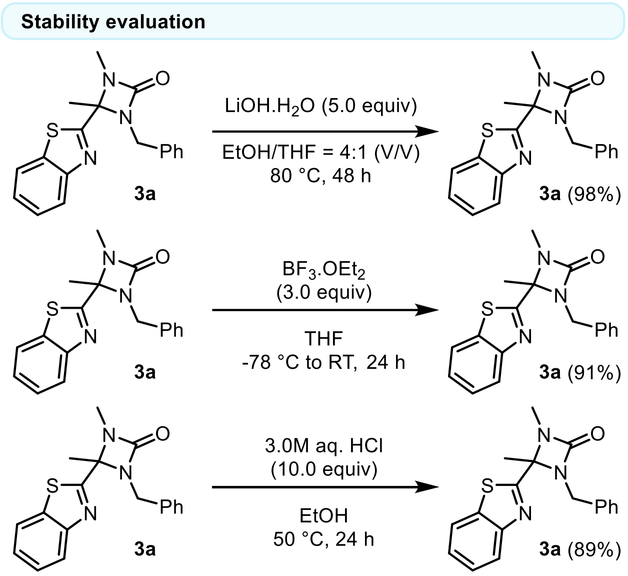

Figure S2. Evaluation of the stability of the aza-β-lactam **3a** under hydrolytic conditions and in the presence of Bronsted and Lewis acids.

5 Reaction Mechanism Determination – Control Experiments

The reaction mechanism proposed in **Fig. 5A** is supported by several key control experiments. Together with the observations described in the main manuscript, the following points provide a coherent picture of the intermediates involved and their relative stabilities.

Indirect evidence for the formation of **Int-3** was obtained using an HAA bearing a *tert*-butyl ester (substrate **S1**, Fig. S3A). Under standard conditions this substrate afforded only **7% of 3a**, whereas the corresponding carbamoyl intermediate **S2a** was isolated in 54% yield. Resubjecting **S2a** to the reaction conditions led to slow formation of **3a**, again accompanied by unreacted **S2a**. These observations are consistent with steric inhibition of **Int-3** formation, shifting the equilibrium toward the carbamate intermediate.

When **4a** is exposed to the standard reaction conditions in the absence of hydroxylamine **5a**, the major isolable product is the carbamoyl intermediate **6a**, which arises from hydrolysis of intermediates **Int-1** and **Int-2** (Fig. S3B). The concurrent formation of **6a**, together with the disappearance of **4a**, indicates that **Int-1** and **Int-2** are in rapid equilibrium and that the α-carbanion formed during the C→N shift is stabilized by the adjacent heteroaryl ring. The two mesomeric forms of **Int-2** (see Fig. S3B) rationalize why the starting material is not recovered: the stabilized carbanion moves the equilibrium away from **4a** and toward downstream intermediates, ultimately producing **6a** upon quenching.

The absence of any detectable aza-β-lactam **3a** under these conditions demonstrates that **Int-3**, the aziridinone-like intermediate, must be highly transient and requires immediate trapping by the amine moiety of hydroxylamine **5a**. Without **5a**, **Int-3** cannot be captured and thus cannot be detected.

To further probe the role of **6a**, we subjected isolated **6a** to the standard reaction conditions in the presence of **5a**, which produced **3a** in 64% yield (Fig. S2C). This re-entry experiment confirms that **6a** lies on the productive pathway and can regenerate the rearrangement sequence under the catalytic conditions. The structure of **6a** was additionally verified by its independent degradation to amine **S3** under Lewis-acid-mediated hydrolysis (Fig. S2C), conditions that selectively hydrolyze the carbamate to the corresponding carbamic acid, followed by decarboxylation. The same product is obtained when HAA ester **4a** is hydrolyzed under basic conditions.

The structure of the hydroxylamine also influences the efficiency of trapping (Fig.S3D). While small changes (e.g., O-Bn) maintain high yields of **3a** (~75%), the sterically demanding O-Ms hydroxylamine **5n** and O-*t*Bu hydroxylamine[16] **5o** fails to produce any **3a**. This strongly suggests that nucleophilic attack of **5a** on **Int-3** is sterically sensitive, and bulky O-substituents hinder productive capture.

Finally, we propose that **Int-5** undergoes **a** [1,2]-heteroaryl migration to form **3a**, rather than proceeding through an alternative pathway involving a second α-carbanion (**Int-S1**, Fig. S4B). Two factors support the migration pathway: (i) Steric considerations – the putative **Int-S1** would require interaction between a trisubstituted carbanion and a sterically congested nitrogen center, making this pathway less favorable. (ii) Stabilization of the carbanion – **Int-S1** would be strongly stabilized by the adjacent heterocycle, yet no protonated analogs or related intermediates are ever observed.

Consistent with this, when thiazole- and oxazole-derived HAAs were evaluated, no traces of the desired product were formed. Instead, only products derived from the **Int-S1-**type intermediate (side-products **8**, Fig. 3) were isolated. We therefore conclude that in these heterocycles the [1,2]-heteroaryl migration is disfavored, likely for geometric reasons and/or because the formation of **Int-S1** is faster than migration, leading exclusively to the corresponding side products.

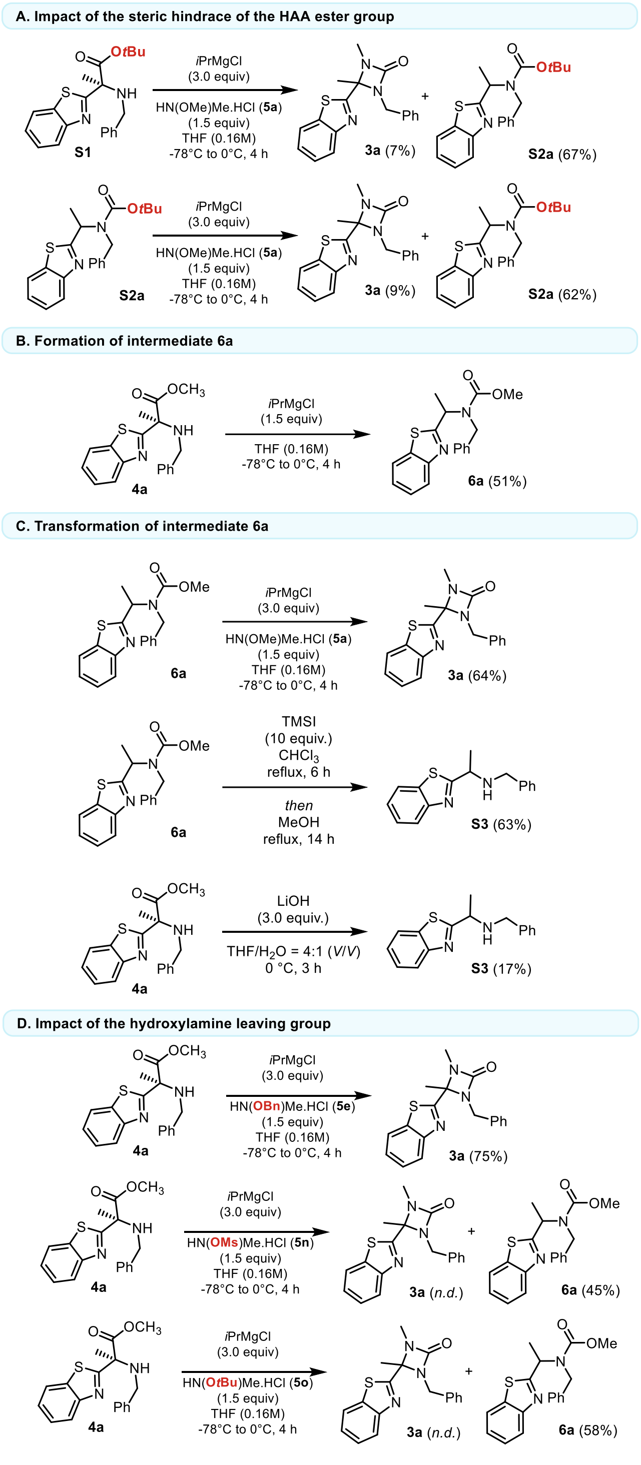

Figure S3. Control experiments supporting the proposed reaction mechanism. (A) *tBu-ester probe (S1):* Standard conditions give **3a** (7%) and accumulate **S2a** (54%); resubmission of **S2a** affords slow**,** partial conversion to **3a**, consistent with steric inhibition of **Int-3**. **(B)** *No hydroxylamine (5a):* **4a** converts exclusively to **6a**, indicating a rapid Int-1 ⇌ Int-2 equilibrium and α-carbanion stabilization that drives hydrolytic capture to **6a**. **(C)** *Re-entry and verification:* Isolated **6a** + **5a** → **3a** (64%) under standard conditions; independent hydrolysis of **6a** → **S3** confirms its structure. **(D)** *Capture sterics:* O-Bn hydroxylamine maintains high yield of **3a** (75%), whereas OMs (**5n**) and O-*t*Bu (**5o**) gives no **3a**, showing steric sensitivity of **Int-3** trapping.

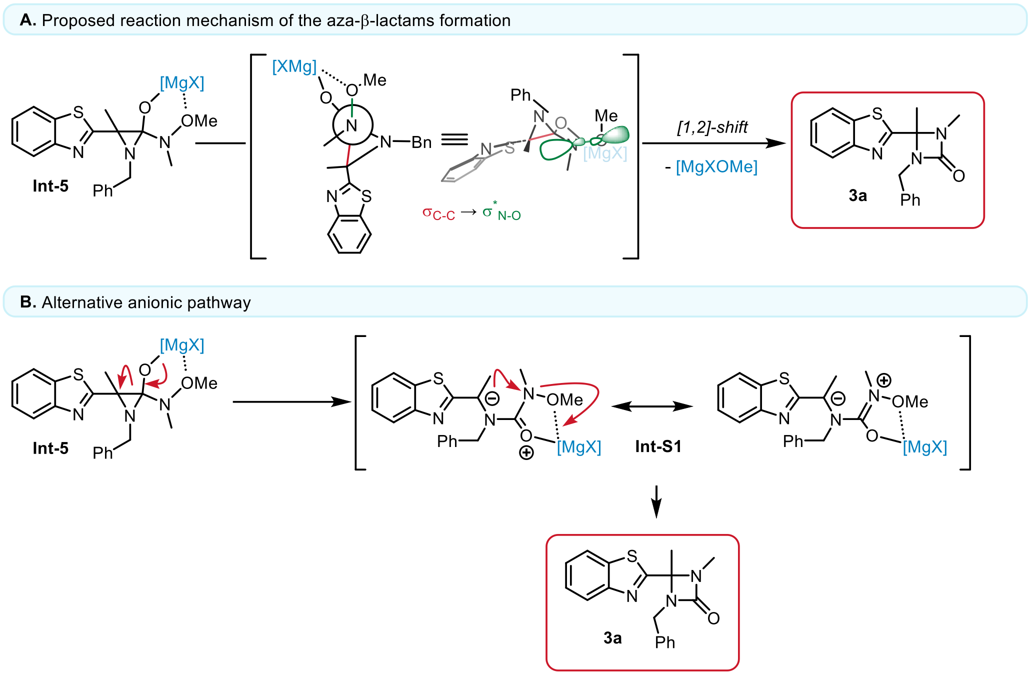

Figure S4. An alternative reaction pathway based on the anionic intermediate **Int-S1** formation.

5.1 Additional mechanistic considerations

Privileged role of the benzo[d]thiazole heterocycle.

A systematic evaluation of alternative heteroaryl substituents (Fig. 3) reveals a uniquely privileged role of the BT moiety. While the precise origin of this effect remains unclear, only the BT heterocycle enables conversion of **Int-3** into **Int-5**, an arrangement geometrically and electronically suited for the subsequent [1,2]-heteroaryl migration. In contrast, substrates bearing other heterocycles either do not enter the rearrangement manifold or diverge toward unproductive pathways. In several cases, excessive stabilization of **Int-1** precludes formation of **Int-2**, as reflected by the absence of racemization of the parent HAA **4**. In oxazole- and thiazole-derived systems, the reaction appears to reach **Int-3**, which can be intercepted by hydroxylamine **5a**; however, the resulting adduct **Int-4** preferentially collapses to the thermodynamically stable, ring-opened ureas **8r** and **8s**.

An alternative scenario involving transient formation of aza-β-lactams **3r** or **3s** followed by rapid ring opening during work-up cannot be excluded. Nevertheless, under all tested reaction and quench conditions, no spectroscopic evidence for **3r** and **3s** was found.

Deuterium-labeling experiments.

The formation of aza-β-lactam **3a** was examined under standard reaction conditions in THF-d_6, followed by quenching with MeOD and subsequent addition of D_2O. Analysis of the crude reaction mixture revealed no detectable incorporation of deuterium in either the aza-β-lactam **3a** or the carbamate side product **6a** (Fig.S5). These observations indicate that protonation steps associated with carbamate formation occur prior to the quench and that no reversible proton–deuterium exchange takes place under the applied conditions.

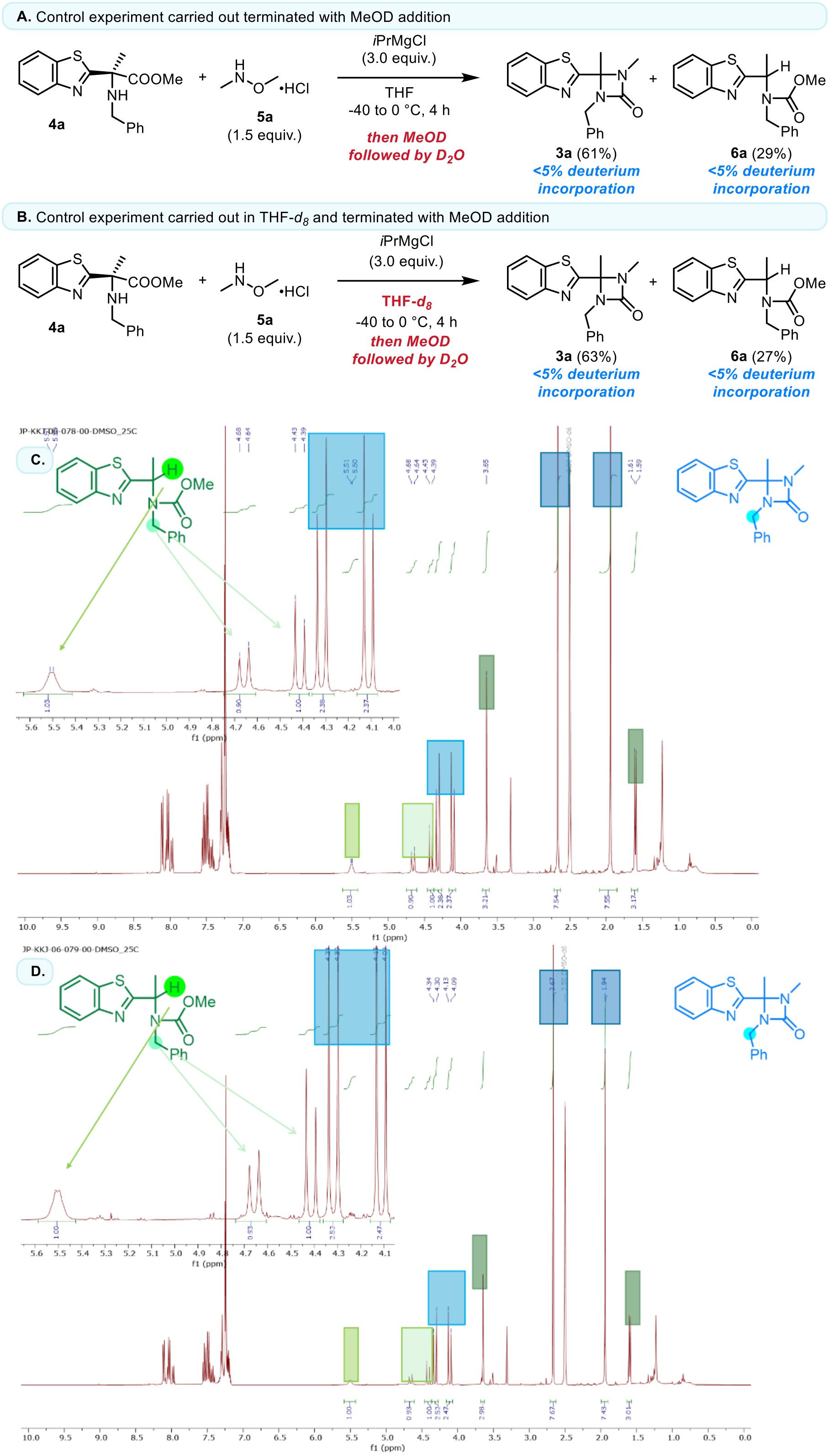

Figure S5. Deuterium-labeling experiments probing proton incorporation during the formation of aza-β-lactam 3a. (A) Reaction scheme carried out under standard conditions in THF, quenched with MeOD and subsequently diluted with D_2O prior to work-up. **(B)** Reaction scheme performed under identical conditions in THF-d_8, followed by quenching with MeOD and dilution with D_2O. **(C)** Representative ^{1}H NMR spectrum of the crude reaction mixture obtained from experiment **A**, showing diagnostic signals corresponding to aza-β-lactam **3a** and carbamate **6a**. **(D)** Representative ^{1}H NMR spectrum of the crude reaction mixture obtained from experiment **B**, showing signals for **3a** and **6a** analogous to those observed in **C**.

Formation of carbamate 6a and stability of Int-2.

Deuterated experiments thus indicate that the carbamate side product **6a** originates from protonation of intermediate **Int-2**, whose stability is enhanced under the standard reaction conditions. The persistence of **Int-2** effectively attenuates the formation of the key aziridinone-like intermediate **Int-3**, thereby limiting productive capture by hydroxylamine **5a** *en route* to aza-β-lactam **3a**. This interpretation is consistent with the increased abundance of **6a** under conditions that favor stabilization of an anionic carbanion/carbamate equilibrium.

6 Unsuccessful substrates

Structures of α-heteroaryl-α-amino Weinreb amide derivatives investigated to probe the influence of alternative α-substituents and anion-stabilizing motifs beyond benzo[d]thiazole. All substrates were recovered unchanged under the standard reaction conditions, and no formation of the corresponding aza-β-lactams was observed (Fig. S6).

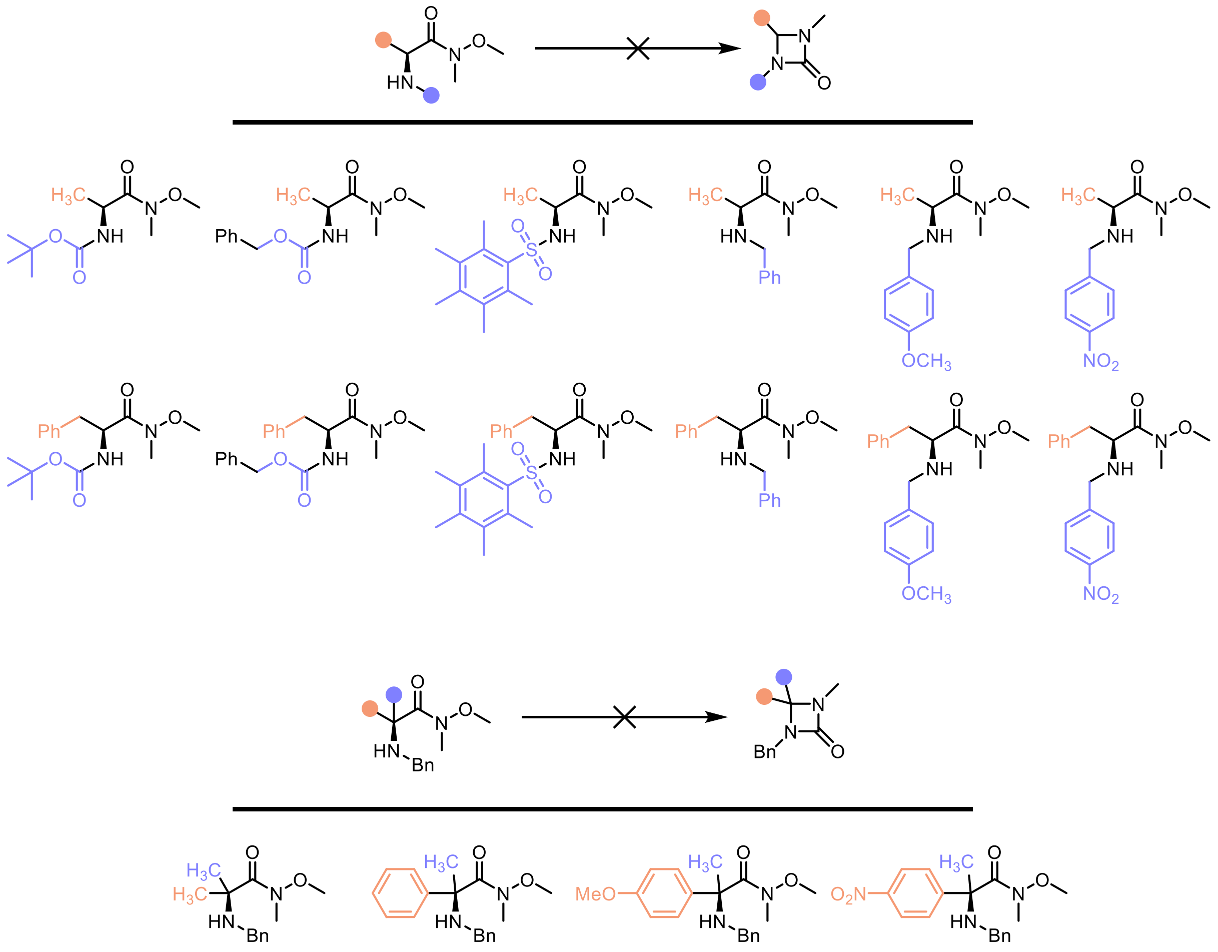

Figure S6. Unsuccessful substrates evaluated under the standard reaction conditions.

7 General Information

All starting materials were purchased from commercial suppliers and used without further purification, unless otherwise stated. All reactions were performed in round-bottom flasks fitted with rubber septa using standard laboratory techniques under positive pressure of argon. Anhydrous solvents were dried according to standard procedures and stored with molecular sieves 3Å. Reactions at low temperatures (-78, -40 °C) were carried out using a cooling bath (dry ice/acetone or N_2/methanol) and indicated temperatures refers to the cooling bath temperature.

Purification of reaction products was carried out by column chromatography using standard grade silica gel (60 Å, 230-400 mesh). Thin layer chromatography (TLC) was performed on precoated silica gel 60 F_{254} plates. Visualization was accomplished with UV light (254 or 366 nm), phosphomolybdic acid, vanillin and potassium permanganate stains, followed by heating.

The determination of melting points was done on a Büchi melting point apparatus. The ^{1}H NMR, ^{13}C NMR and ^{19}F NMR spectra were measured on Jeol ECA400II (400 MHz) or Jeol 500 ECA (500 MHz) in $CDCl_3$, DMSO-d_6, D_2O or CD_3OD. Chemical shifts are reported in ppm, and their calibration was performed (a) in the case of ^{1}H NMR experiments on the residual peak of non-deuterated solvent δ ($CDCl_3$) = 7.26 ppm, δ (DMSO-d_6) = 2.50 ppm, δ (D_2O) = 4.79 ppm or δ (CD_3OD) = 3.31 ppm, (b) in the case of ^{13}C NMR experiments on the middle peak of the ^{13}C signal in deuterated solvent δ ($CDCl_3$) = 77.16 ppm, δ (DMSO-d_6) = 39.52 ppm or δ (CD_3OD) = 49.00 ppm. All ^{13}C NMR spectra were acquired with broadband ^{1}H and ^{19}F decoupling respectively. The proton coupling patterns are represented as a singlet (s), a doublet (d), a doublet of a doublet (dd), a triplet (t), a triplet of a triplet (tt), and a multiplet (m).

High-resolution mass spectrometry (HRMS) was performed on Agilent 6230 high-resolution mass spectrometer with electrospray ionization (ESI) and a time-of-flight analyzer operating in a positive or negative full scan mode in the range of 100 − 1700 m/z. High-performance liquid chromatography (HPLC) was performed using an Agilent 1290 Infinity II system with UV-VIS detector and an Agilent InfinityLab LC/MSD mass detector.

Chiral analysis was performed on Waters Alliance 2695 with autosampler and UV-VIS detector Waters 2996 PDA using chiral columns (CHIRAL ART Amylose-SA 250x4.6 mm, 5 µm; CHIRALCEL Cellulose OD-H, 250x4.6 mm, 5 µm; CHIRALCEL Cellulose OZ-H, 250x4.6 mm, 5 µm). All solvents used were HPLC-grade solvents purchased from Merk. The column employed and the respective solvent mixture are indicated for each experiment.

Specific rotations ($[\alpha]_D^T$) were measured with Perkin Elmer Polarimeter 241 Automatic (Massachusetts, USA) at the indicated temperature. Measurements were performed in a 1 ml cell (50 mm length) with concentrations (g/(100 ml)) reported in corresponding solvent. All microwave irradiation experiments were carried out in a dedicated CEM-Discover mono-mode microwave apparatus. The reactor was used in the standard configuration as delivered, including proprietary software. The reactions were carried out in 10- or 35-mL glass vials that were sealed with silicone/PTFE caps, which can be exposed to a maximum of 250 °C and 20 bar internal pressure. The temperature was measured with an IR sensor on the outer surface of the process vial. After the irradiation period, the reaction vessels were cooled to ambient temperature by gas jet cooling.

Elemental analyses (C, H, N, S) were performed on a FlashSmart CHNS elemental analyzer (Thermo Fisher Scientific), equipped with a single-furnace combustion unit, a thermal conductivity detector (TCD), and a MAS Plus autosampler. Data acquisition and processing were carried out using EagerSmart

data handling software. Samples were analyzed using the standard combustion method under conditions recommended by the manufacturer.

All the NMR yields were determined by ^{1}H NMR analysis of the crude reaction mixture with trichloroethylene (0.05 mmol, signal – 6.46 (s, 1H)) as internal standard. Subsequently, the signal of the standard and product were integrated and NMR yields were computed using the following formula:

$$Y = \frac{I_P}{I_{IS}} \cdot \frac{N_{IS}}{N_P} \cdot \frac{n_{IS}}{n_{SM}} \cdot P_{IS} \,,$$

where

Y = NMR yield
I_P = integral of the product
I_{IS} = integral of the internal standard
N_{IS} = number of internal standard protons
N_P = number of product protons
n_{IS} = moles of the internal standard
n_{SM} = moles of the starting material
P_{IS} = purity of the internal standard

7.1 Starting Material Preparation

7.1.1 Synthesis of α-heteroaryl-α-amino esters and amides

N,N-Disubstituted sulfonamides **S1–S25** were prepared according to previously published procedures.[13,30]

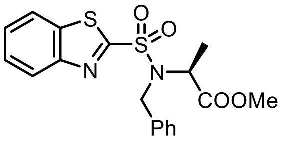

S4

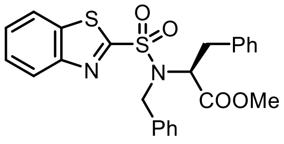

S5

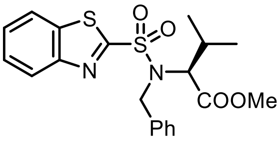

S6

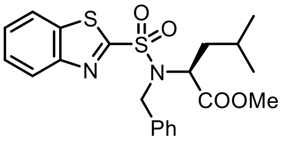

S7

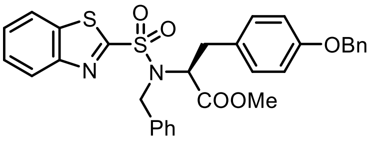

S8

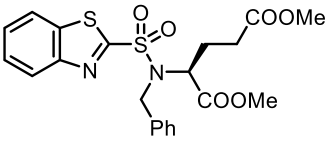

S9

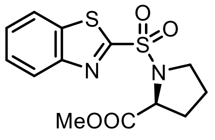

S10

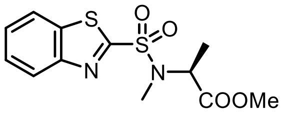

S11

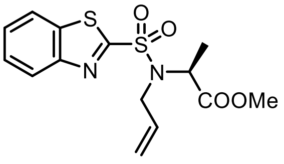

S12

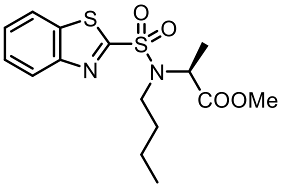

S13

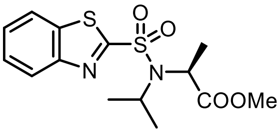

S14

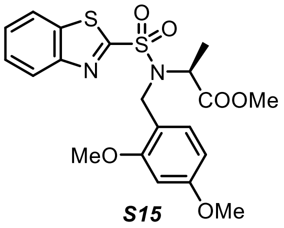

S15

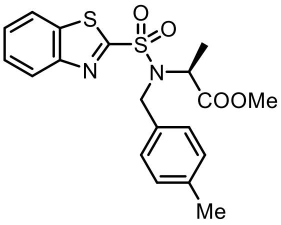

S16

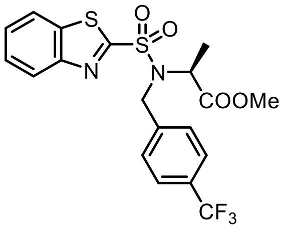

S17

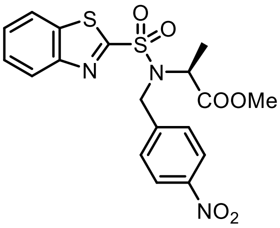

S18

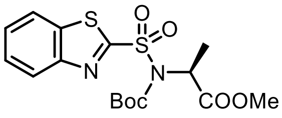

S19

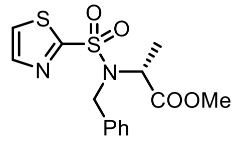

S20

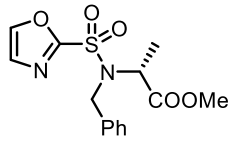

S21

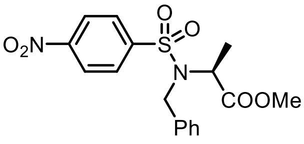

S22

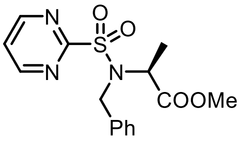

S23

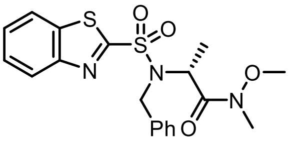

S24

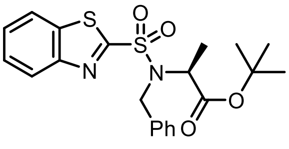

S25

α-Heteroaryl-α-amino esters and amides (**4a–u**, **1a**, and **S1**) were prepared according to previously published protocols.[13]

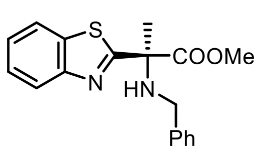

4a

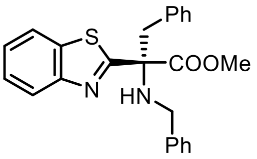

4b

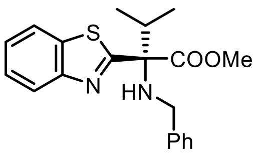

4c

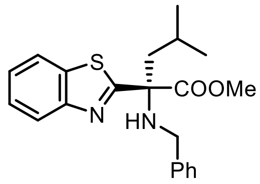

4d

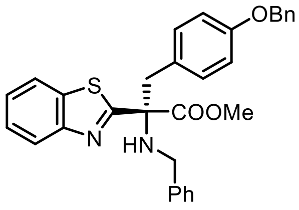

4e

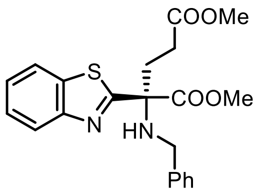

4f

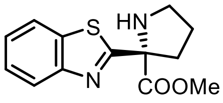

4g

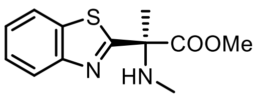

4h

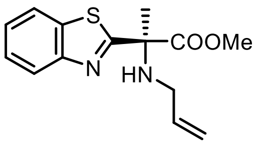

4i

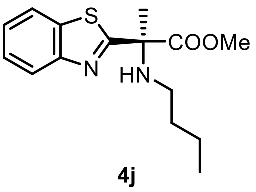

4j

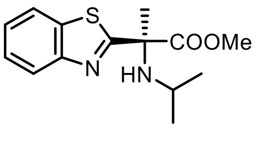

4k

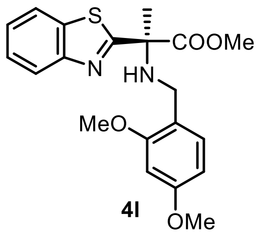

4l

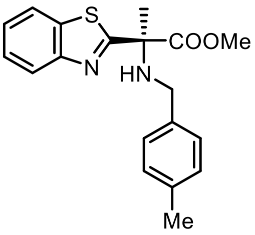

4m

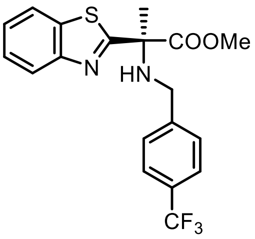

4n

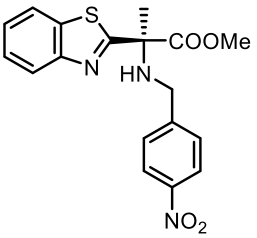

4o

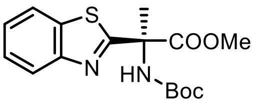

4p

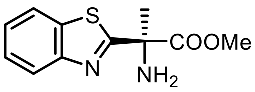

4q

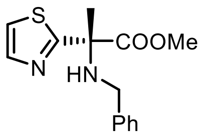

4r

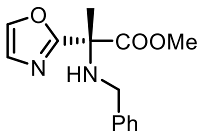

4s

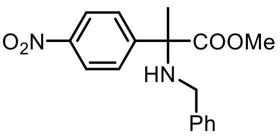

4t

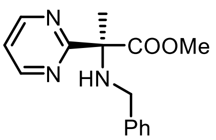

4u

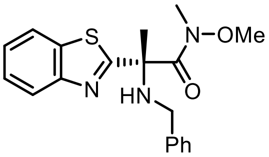

1a

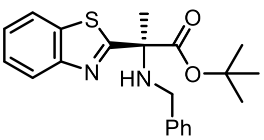

S1

General procedure 1 (GP1):

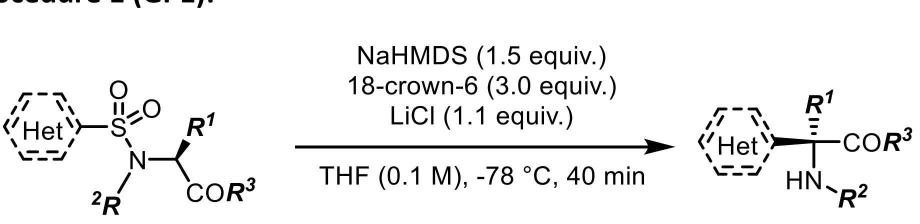

S1-S21 **4a-v, 1a, S1**

Following a reported procedure, to a solution of sulfonamide ***S1-S21*** (1.0 equiv.) in dry THF (0.1 M) was added 18-crown-6 (3.0 equiv.) and LiCl (1.1 equiv.) under inert atmosphere of Ar. The whole reaction mixture was cooled down to -78 °C (dry ice/acetone) and NaHMDS (1 mol/l in THF, 1.5 equiv.) was slowly added. The reaction was mixing at -78°C and after 40 minutes resulting mixture was quenched by aq. sat. solution of NH_4Cl and EtOAc (same amount as THF) was added. Resulting layers were separated and the aqueous layer was extracted 3x EtOAc (3 times of THF). Combined organic layers were washed with brine, dried over Na_2SO_4, solids filtered and the solvents were removed under reduced pressure. The crude product was purified by gradient column chromatography on silica gel.

7.1.1.1 Methyl (R)-2-(benzo[d]thiazol-2-yl)-2-(benzylamino)propanoate (4a)

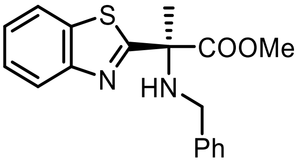

4a

Using sulfonamide ***S4*** according to **GP1**, provided **4a** (551 mg, 1.69 mmol, 84 %, *e.r.* = 96:4) as a colorless oil, after purification by column chromatography (SiO_2, hexane/EtOAc = 15:1 to 4:1).

R_f = 0.27 (hexane/EtOAc = 3:1)

$[\alpha]_D^{24}$ = +11.7° (*c* 1.0, $CHCl_3$).

^{1}H NMR (400 MHz, $CDCl_3$) δ (ppm): 8.04 (dt, *J* = 8.1, 1.0 Hz, 1H), 7.89 (dt, *J* = 8.0, 0.9 Hz, 1H), 7.50 – 7.33 (m, 6H), 7.31 – 7.26 (m, 1H), 3.84 – 3.79 (m, 1H), 3.79 (s, 3H), 2.83 (bs, 1H), 1.96 (s, 3H).

^{13}C {^{1}H} NMR (101 MHz, $CDCl_3$) δ (ppm): 175.2, 172.9, 153.5, 139.7, 135.6, 128.6, 128.4, 127.4, 126.1, 125.3, 123.5, 121.8, 66.5, 53.2, 48.2, 23.7.

HRMS (ESI) *m/z:* $[M+H]^+$ calc. for $C_{18}H_{19}N_2O_2S$, 327.1167; found, 327.1166.

HPLC (SA, Hexane : iPrOH = 90:10, 0.5 mL/min, 298 K, 220 nm): t_{R1} = 11.95 min, t_{R2} = 14.95 min.

Methyl (*R*)-2-(benzo[*d*]thiazol-2-yl)-2-(benzylamino)-3-phenylpropanoate (4b)

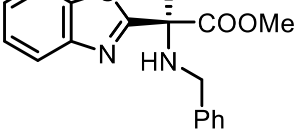

4b

Using sulfonamide ***S5*** according to **GP1**, provided **4b** (500 mg, 1.24 mmol, 96 %, *e.r.* = 9:91) as a colorless oil, after purification by column chromatography (SiO_2, hexane/EtOAc = 15:1 to 5:1).

$\mathbf{R_f}$ = 0.69 (hexane/EtOAc = 2:1)

$[\alpha]_D^{22}$ **=** -17.7° (*c* 1.0, $CHCl_3$).

^{1}H NMR (400 MHz, $CDCl_3$) δ (ppm): 8.10 (ddd, *J* = 8.1, 1.0, 0.5 Hz, 1H), 7.87 (ddd, *J* = 7.9, 1.2, 0.6 Hz, 1H), 7.50 (ddd, *J* = 8.3, 7.2, 1.3 Hz, 1H), 7.44 – 7.37 (m, 3H), 7.37 – 7.30 (m, 2H), 7.30 – 7.21 (m, 5H), 7.16 (tt, *J* = 4.9, 2.3 Hz, 2H), 3.96 (d, *J* = 14.1 Hz, 1H), 3.94 (d, *J* = 12.6 Hz, 1H), 3.81 (d, *J* = 12.6 Hz, 1H), 3.72 (d, *J* = 14.0 Hz, 1H), 3.71 (s, 3H).

^{13}C {^{1}H} NMR (101 MHz, $CDCl_3$) δ (ppm): 173.5, 171.4, 153.2, 139.5, 136.1, 135.5, 130.2, 128.6, 128.3, 127.4, 127.3, 126.1, 125.4, 123.6, 121.9, 70.8, 53.0, 47.7, 41.1.

HRMS (ESI) ***m/z:*** $[M+H]^+$ calculated for $C_{24}H_{22}N_2O_2S$, 403.1475; found, 403.1474.

HPLC (SA, Hexane : iPrOH = 90:10, 0.5 mL/min, 298 K, 220 nm): t_{R1} = 13.55 min, t_{R2} = 14.95 min.

7.1.1.2 Methyl (R)-2-(benzo[d]thiazol-2-yl)-2-(benzylamino)-3-methylbutanoate (4c)

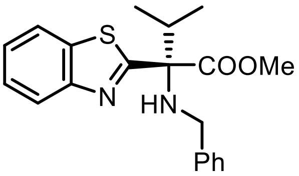

4c

Using sulfonamide ***S6*** according to **GP1**, provided **4c** (165 mg, 0.47 mmol, 93 %, *e.r.* = 99:1) as a colorless oil, after purification by column chromatography (SiO_2, hexane/EtOAc = 15:1 to 5:1).

$\mathbf{R_f}$ = 0.55 (hexane/EtOAc = 3:1)

$[\alpha]_D^{22}$ **=** +31.7° (*c* 0.6, $CHCl_3$).

^{1}H NMR (500 MHz, $CDCl_3$) δ (ppm): 8.09 (dt, *J* = 8.1, 0.8 Hz, 1H), 7.90 (ddd, *J* = 7.9, 1.3, 0.6 Hz, 1H), 7.50 – 7.42 (m, 3H), 7.40 – 7.33 (m, 3H), 7.31 – 7.26 (m, 1H), 3.85 (s, 3H), 3.78 (d, *J* = 12.5 Hz, 1H), 3.69 (d, *J* = 12.5 Hz, 1H), 2.69 (h, *J* = 6.8 Hz, 1H), 2.47 (bs, 1H), 1.12 (d, *J* = 6.7 Hz, 3H), 1.00 (d, *J* = 6.9 Hz, 3H).

^{13}C {^{1}H} NMR (126 MHz, $CDCl_3$) δ (ppm): 172.1, 171.7, 153.3, 139.8, 135.4, 128.6, 128.3, 127.4, 125.8, 125.1, 123.5, 121.5, 73.4, 52.6, 49.1, 36.9, 18.4, 17.7.

HRMS (ESI) ***m/z:*** $[M+H]^+$ calc. for $C_{20}H_{23}N_2O_2S$, 355.1475; found, 355.1471.

HPLC (SA, Hexane : iPrOH = 90:10, 0.5 mL/min, 298 K, 220 nm): t_{R1} = 10.12 min, t_{R2} = 14.18 min.

7.1.1.3 *Methyl (R)-2-(benzo[d]thiazol-2-yl)-2-(benzylamino)-4-methylpentanoate (4d)*

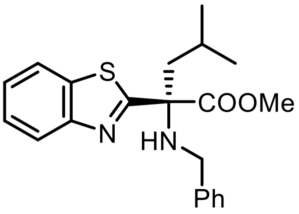

4d

Using sulfonamide ***S7*** according to **GP1**, provided **4d** (158 mg, 0.43 mmol, 86 %, *e.r.* = 95:5) as a colorless oil, after purification by column chromatography (SiO_2, hexane/EtOAc = 15:1 to 5:1).

R_f = 0.75 (hexane/EtOAc = 2:1)

$[\alpha]_D^{24}$ = +12.0° (*c* 1.0, $CHCl_3$).

^1H NMR (500 MHz, $CDCl_3$) δ (ppm): 8.06 (dd, *J* = 8.2, 1.0 Hz, 1H), 7.89 (dd, *J* = 8.0, 1.1 Hz, 1H), 7.48 (ddd, *J* = 8.2, 7.3, 1.3 Hz, 1H), 7.45 – 7.41 (m, 2H), 7.41 – 7.38 (m, 1H), 7.37 – 7.33 (m, 2H), 7.30 – 7.27 (m, 1H), 3.74 (s, 3H), 3.72 – 3.63 (m, 2H), 2.96 (bs, 1H), 2.53 (dd, *J* = 14.6, 7.3 Hz, 1H), 2.43 – 2.34 (m, 1H), 1.87 (hd, *J* = 6.8, 4.8 Hz, 1H), 0.97 (d, *J* = 6.7 Hz, 3H), 0.93 (d, *J* = 6.6 Hz, 3H).

^{13}C {^1H} NMR (126 MHz, $CDCl_3$) δ (ppm): 172.5, 153.4, 135.8, 128.6, 128.4, 127.4, 126.1, 125.3, 123.5, 121.9, 69.1, 53.1, 47.5, 43.3, 24.4, 24.1, 23.2.

HRMS (ESI) *m/z:* $[M+H]^+$ calc. for $C_{21}H_{25}N_2O_2S$, 369.1631; found, 369.1635.

HPLC (SA, Hexane : iPrOH = 80:10, 0.5 mL/min, 298 K, 220 nm): t_{R1} = 10.72 min, t_{R2} = 11.58.

7.1.1.4 *Methyl (R)-2-(benzo[d]thiazol-2-yl)-2-(benzylamino)-3-(4(benzyloxy)phenyl)propanoate (4e)*

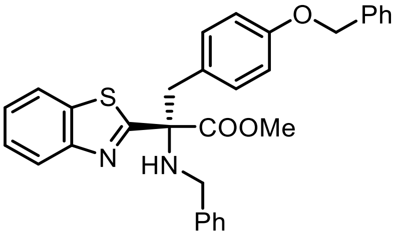

4e

Using sulfonamide ***S8*** according to **GP1**, provided **4e** (158 mg, 0.31 mmol, 78 %, *e.r.* = 85:15) as a colorless oil, after purification by column chromatography (SiO_2, hexane/EtOAc = 15:1 to 5:1).

R_f = 0.65 (hexane/EtOAc = 4:1)

$[\alpha]_D^{21}$ = +18.6° (*c* 1.2, $CHCl_3$).

^1H NMR (500 MHz, $CDCl_3$) δ (ppm): 8.09 (d, J = 8.2 Hz, 1H), 7.88 (d, J = 8.0 Hz, 1H), 7.50 (t, J = 7.7 Hz, 1H), 7.44 – 7.36 (m, 7H), 7.34 (t, J = 7.9 Hz, 3H), 7.32 – 7.22 (m, 2H), 7.06 (d, J = 8.1 Hz,

2H), 6.88 – 6.81 (m, 2H), 5.01 (s, 2H), 3.91 (dd, J = 17.3, 13.4 Hz, 2H), 3.80 (d, J = 12.7 Hz, 1H), 3.71 (d, J = 1.4 Hz, 3H), 3.65 (d, J = 14.3 Hz, 1H).

^{13}C {^{1}H} NMR (126 MHz, CDCl$_3$) δ (ppm): 173.6, 171.5, 158.1, 153.2, 139.5, 137.1, 136.0, 131.2, 128.7, 128.6, 128.3, 128.1, 127.7, 127.6, 127.4, 126.0, 125.4, 123.6, 121.9, 114.9, 70.8, 70.1, 53.0, 47.7, 40.4.

HRMS (ESI) *m/z:* [M+H]$^+$ calc. for $C_{31}H_{29}N_2O_3S$, 509.1893; found: 509.1895.

HPLC (SA, Hexane : iPrOH = 80:20, 0.5 mL/min, 298 K, 220 nm): t_{R1} = 19.32 min, t_{R2} = 27.33 min.

7.1.1.5 Dimethyl (R)-2-(benzo[d]thiazol-2-yl)-2-(benzylamino)pentanedioate (4f)

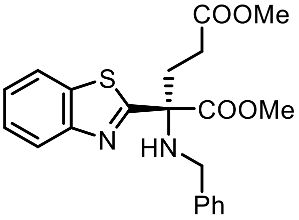

4f

Using sulfonamide ***S9*** according to **GP1**, provided **4f** (88 mg, 0.22 mmol, 56 %, *e.r.* = 94:6) as a white solid, after purification by column chromatography (SiO$_2$, hexane/EtOAc = 15:1 to 10:1).

R$_f$ = 0.39 (hexane/EtOAc = 3:1)

[α]$_D^{20}$ = -12.1° (*c* 1.2, CHCl$_3$)

m. p. = 80-82 °C

^{1}H NMR (500 MHz, CDCl$_3$) δ (ppm): 8.05 (dt, *J* = 8.2, 0.8 Hz, 1H), 7.89 (dt, *J* = 7.9, 0.9 Hz, 1H), 7.49 (ddd, *J* = 8.3, 7.2, 1.3 Hz, 1H), 7.42 – 7.40 (m, 3H), 7.37 – 7.33 (m, 2H), 7.30 – 7.26 (m, 1H), 3.78 (s, 3H), 3.74 (d, *J* = 12.4 Hz, 1H), 3.70 (d, *J* = 12.3 Hz, 1H), 3.63 (s, 3H), 2.89 – 2.82 (m, 1H), 2.77 – 2.71 (m, 1H), 2.46 (t, *J* = 8.1 Hz, 2H).

^{13}C {^{1}H} NMR (126 MHz, CDCl$_3$) δ (ppm): 173.3, 173.0, 171.8, 153.3, 139.3, 135.4, 128.6, 128.3, 127.5, 126.2, 125.4, 123.6, 121.8, 68.7, 53.2, 51.9, 47.5, 30.1, 28.8.

HRMS (ESI) *m/z:* [M+H]$^+$ calc. for $C_{21}H_{23}N_2O_4S$, 399.1373; found, 399.1370.

HPLC (SA, Hexane : iPrOH = 80:20, 0.5 mL/min, 298 K, 220 nm): t_{R1} = 14.78 min, t_{R2} = 16.70 min.

7.1.1.6 Methyl (R)-2-(benzo[d]thiazol-2-yl)pyrrolidine-2-carboxylate (4g)

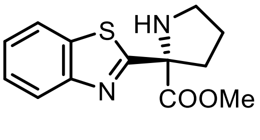

4g

Using sulfonamide ***S10*** according to **GP1**, provided **4g** (91 mg, 0.35 mmol, 87 %, *e.r.* = 95:5) as a colorless oil, after purification by column chromatography (SiO_2, CH_2Cl_2/acetone = 100:1 to 25:1).

R_f = 0.63 (CH_2Cl_2/acetone = 20:1)

$[\alpha]_D^{21}$ = +22.0° (*c* 1.2, $CHCl_3$).

^{1}H NMR (400 MHz, $CDCl_3$) δ (ppm): 8.01 (ddd, *J* = 8.1, 1.2, 0.7 Hz, 1H), 7.86 (ddd, *J* = 8.0, 1.3, 0.6 Hz, 1H), 7.44 (ddd, *J* = 8.3, 7.2, 1.3 Hz, 1H), 7.35 (ddd, *J* = 8.3, 7.3, 1.2 Hz, 1H), 3.80 (s, 3H), 3.36 (bs. 1H), 3.22 – 3.10 (m, 2H), 2.69 – 2.54 (m, 2H), 1.95 – 1.85 (m, 2H).

^{13}C {^{1}H} NMR (101 MHz, $CDCl_3$) δ (ppm): 177.3, 173.1, 154.3, 135.9, 125.9, 125.0, 123.3, 121.8, 72.4, 53.6, 47.1, 38.0, 26.1.

HRMS (ESI) *m/z:* [M+H]$^+$ calc. for $C_{13}H_{15}N_2O_2S$, 263.0849; found, 263.0850.

HPLC (OZ, Hexane : iPrOH = 95:5, 0.5 mL/min, 298 K, 220 nm): t_{R1} = 21.00 min, t_{R2} = 26.02 min.

7.1.1.7 Methyl (R)-2-(benzo[d]thiazol-2-yl)-2-(methylamino)propanoate (4h)

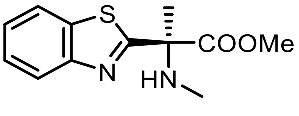

4h

Using sulfonamide ***S11*** according to **GP1**, provided **4h** (118 mg, 0.47 mmol, 95 %, *e.r.* = 98:2) as a colorless oil, after purification by column chromatography (SiO_2, cyclohexane/EtOAc = 15:1 to 5:1).

R_f = 0.29 (cyclohexane/EtOAc = 3:1)

$[\alpha]_D^{20}$ = -6.5° (*c* 1.0, $CHCl_3$).

^{1}H NMR (500 MHz, $CDCl_3$) δ (ppm): 8.02 (dt, *J* = 8.1, 1.0, 0.6 Hz, 2H), 7.88 (dt, *J* = 8.0, 1.3, 0.6 Hz, 1H), 7.47 (ddd, *J* = 8.3, 7.2, 1.3 Hz, 1H), 7.38 (ddd, *J* = 8.2, 7.2, 1.2 Hz, 1H), 3.79 (s, 3H), 2.43 (s, 3H), 1.85 (s, 3H).

^{13}C {^{1}H} NMR (126 MHz, $CDCl_3$) δ (ppm): 175.0, 173.0, 153.4, 135.5, 126.1, 125.3, 123.5, 121.8, 66.8, 53.2, 30.5, 22.8.

HRMS (ESI) *m/z:* [M+H]$^+$ calc. for $C_{12}H_{15}N_2O_2S$, 251.0849; found, 251.0849.

HPLC (SA, Hexane : iPrOH = 90:10, 0.5 mL/min, 298 K, 220 nm): t_{R1} = 10.55 min, t_{R2} = 12.98 min.

7.1.1.8 Methyl (R)-2-(allylamino)-2-(benzo[d]thiazol-2-yl)propanoate (4i)

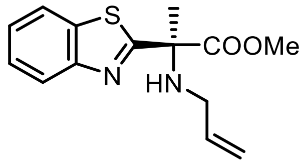

4i

Using sulfonamide ***S12*** according to **GP1**, provided **4i** (118 mg, 0.43 mmol, 85 %, *e.r.* = 6:94) as a colorless oil, after purification by column chromatography (SiO_2, cyclohexane/EtOAc = 15:1 to 5:1).

R_f = 0.43 (cyclohexane/EtOAc = 3:1)

$[\alpha]_D^{24}$ = +11.2° (*c* 1.0, $CHCl_3$).

^{1}H NMR (500 MHz, $CDCl_3$) δ (ppm): 8.02 (dd, *J* = 8.2, 1.0 Hz, 1H), 7.87 (dd, *J* = 7.9, 1.1 Hz, 1H), 7.46 (ddt, *J* = 8.1, 7.1, 1.1 Hz, 1H), 7.37 (ddt, *J* = 7.9, 7.0, 0.9 Hz, 1H), 6.00 – 5.91 (m, 1H), 5.33 – 5.22 (m, 1H), 5.18 – 5.09 (m, 1H), 3.78 (s, 3H), 3.34 – 3.20 (m, 2H), 1.90 (s, 3H).

^{13}C {^{1}H} NMR (126 MHz, $CDCl_3$) δ (ppm): 175.0, 172.8, 153.4, 136.0, 135.6, 126.1, 125.3, 123.5, 121.8, 116.7, 66.2, 53.2, 46.6, 23.4.

HRMS (ESI) *m/z:* $[M+H]^+$ calc. for $C_{14}H_{17}N_2O_2S$, 277.1005; found: 277.1008.

HPLC (SA, Hexane: iPrOH = 90:10, 0.5 mL/min, 298 K, 220 nm): t_{R1} = 9.70 min, t_{R2} = 11.12 min.

7.1.1.9 Methyl (R)-2-(benzo[d]thiazol-2-yl)-2-(butylamino)propanoate (4j)

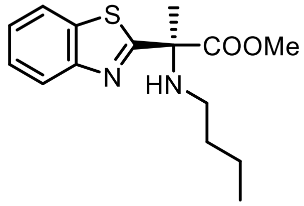

4j

Using sulfonamide ***S13*** according to **GP1**, provided **4j** (132 mg, 0.45 mmol, 90 %, *e.r.* = 97:3) as a colorless oil, after purification by column chromatography (SiO_2, cyclohexane/EtOAc = 15:1 to 5:1).

R_f = 0.47 (cyclohexane/EtOAc = 3:1)

$[\alpha]_D^{20}$ = +20.4° (*c* 1.0, $CHCl_3$).

^{1}H NMR (500 MHz, $CDCl_3$) δ (ppm): 8.01 (dt, *J* = 8.2, 0.9 Hz, 1H), 7.87 (dt, *J* = 8.0, 1.0 Hz, 1H), 7.45 (ddd, *J* = 8.3, 7.2, 1.3 Hz, 1H), 7.36 (ddd, *J* = 8.2, 7.1, 1.2 Hz, 1H), 3.77 (s, 3H), 2.66 – 2.51 (m, 2H), 2.49 (bs, 1H), 1.86 (s, 3H), 1.58 – 1.48 (m, 2H), 1.39 (h, *J* = 7.3 Hz, 2H), 0.91 (t, *J* = 7.3 Hz, 3H).

^{13}C {^{1}H} NMR (126 MHz, $CDCl_3$) δ (ppm): 175.6, 173.1, 153.4, 135.5, 126.0, 125.2, 123.4, 121.8, 66.3, 53.1, 43.5, 32.7, 23.4, 20.5, 14.1.

HRMS (ESI) ***m/z:*** $[M+H]^+$ calc. for $C_{15}H_{21}N_2O_2S$, 293.1318; found, 293.1317.

HPLC (SA, Hexane : iPrOH = 90:10, 0.5 mL/min, 298 K, 220 nm): t_{R1} = 9.07 min, t_{R2} = 10.67 min.

7.1.1.10 Methyl (R)-2-(benzo[d]thiazol-2-yl)-2-(isopropylamino)propanoate (4k)

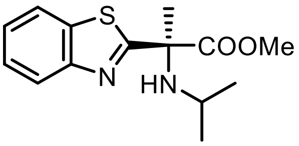

4k

Using sulfonamide ***S14*** according to **GP1**, provided **4k** (108 mg, 0.39 mmol, 78 %, *e.r.* = 1:99) as a colorless oil, after purification by column chromatography (SiO_2, cyclohexane/EtOAc = 15:1 to 5:1).

R_f = 0.53 (cyclohexane/EtOAc = 3:1)

$[\alpha]_D^{20}$ = -24.2° (*c* 1.0, $CHCl_3$).

^{1}H NMR (500 MHz, $CDCl_3$) δ (ppm): 8.00 (dd, *J* = 7.9, 1.5 Hz, 1H), 7.86 (dd, *J* = 7.9, 1.6 Hz, 1H), 7.45 (tt, *J* = 8.7, 1.5 Hz, 1H), 7.36 (td, *J* = 7.6, 6.9, 1.4 Hz, 1H), 3.77 (s, 3H), 3.08 – 3.00 (m, 1H), 2.55 (bs, 1H), 1.86 (s, 3H), 1.10 (ddd, *J* = 5.9, 4.1, 1.7 Hz, 6H).

^{13}C {^{1}H} NMR (126 MHz, $CDCl_3$) δ (ppm): 177.4, 174.2, 153.5, 135.5, 126.0, 125.1, 123.4, 121.8, 65.8, 53.1, 45.0, 25.3, 24.6, 23.8.

HRMS (ESI) ***m/z:*** $[M+H]^+$ calc. for $C_{14}H_{19}N_2O_2S$, 279.1167; found, 279.1169.

HPLC (OJ, Hexane : iPrOH = 90:10, 0.5 mL/min, 298 K, 220 nm): t_{R1} = 7.60 min, t_{R2} = 8.70 min.

7.1.1.11 Methyl (R)-2-(benzo[d]thiazol-2-yl)-2-((2,4-dimethoxybenzyl)amino)propanoate (4l)

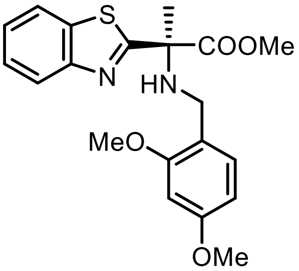

4l

Using sulfonamide ***S15*** according to **GP1**, provided **4l** (837 mg, 2.17 mmol, 98 %, *e.r.* = 99:1) as a colorless oil, after purification by column chromatography (SiO_2, hexane/EtOAc = 15:1 to 5:1).

R_f = 0.58 (hexane/EtOAc = 3:1)

$[\alpha]_D^{31}$ = -6.8° (*c* 1.0, $CHCl_3$).

^{1}H NMR (500 MHz, $CDCl_3$) δ (ppm): 8.07 – 7.97 (m, 1H), 7.91 – 7.83 (m, 1H), 7.51 – 7.41 (m, 1H), 7.42 – 7.32 (m, 1H), 7.25 (d, *J* = 8.1 Hz, 1H), 6.50 – 6.40 (m, 2H), 3.83 (s, 1H), 3.80 (s, 3H), 3.80 (s, 3H), 3.72 (s, 1H), 3.69 (s, 3H), 1.94 (s, 3H).

^{13}C {^{1}H} NMR (126 MHz, $CDCl_3$) δ (ppm): 176.1, 172.9, 160.3, 158.6, 153.5, 135.6, 130.4, 126.0, 125.1, 123.4, 121.8, 120.2, 104.0, 98.6, 66.2, 55.5, 55.4, 53.1, 42.9, 23.4.

HRMS (ESI) *m/z:* $[M+H]^+$ calculated for $C_{20}H_{22}N_2O_4S$, 387.1373; found, 387.1372.

HPLC (SA, Hexane : iPrOH = 90:10, 0.5 mL/min, 298 K, 220 nm): t_{R1} = 15.35 min, t_{R2} = 18.32 min.

7.1.1.12 Methyl (R)-2-(benzo[d]thiazol-2-yl)-2-((4-methylbenzyl)amino)propanoate (4m)

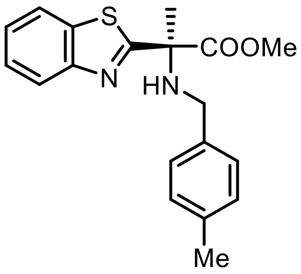

4m

Using sulfonamide ***S16*** according to **GP1**, provided **4m** (157 mg, 0.46 mmol, 92 %, *e.r.* = 99:1) as a colorless oil, after purification by column chromatography (SiO_2, cyclohexane/EtOAc = 15:1 to 5:1).

R_f = 0.46 (cyclohexane/EtOAc = 3:1)

$[\alpha]_D^{30}$ = -2.9° (*c* 1.0, $CHCl_3$).

^{1}H NMR (500 MHz, $CDCl_3$) δ (ppm): 8.04 (d, *J* = 8.0 Hz, 1H), 7.89 (d, *J* = 7.9 Hz, 1H), 7.47 (t, *J* = 7.7 Hz, 1H), 7.38 (t, *J* = 7.6 Hz, 1H), 7.32 (d, *J* = 7.7 Hz, 2H), 7.16 (d, *J* = 7.5 Hz, 2H), 3.78 (s, 3H), 3.74 (d, *J* = 13.6 Hz, 2H), 2.79 (s, 1H), 2.35 (s, 3H), 1.95 (s, 3H).

^{13}C {^{1}H} NMR (126 MHz, $CDCl_3$) δ (ppm): 175.3, 172.9, 153.5, 137.0, 136.6, 135.6, 129.3, 128.3, 126.0, 125.3, 123.5, 121.8, 66.5, 53.2, 47.9, 23.6, 21.3.

HRMS (ESI) *m/z:* $[M+H]^+$ calculated for $C_{19}H_{20}N_2O_2S$, 341.1318; found, 341.1318.

HPLC (SA, Hexane : iPrOH = 90:10, 0.5 mL/min, 298 K, 220 nm): t_{R1} = 12.03 min, t_{R2} = 14.92 min.

7.1.1.13 Methyl (R)-2-(benzo[d]thiazol-2-yl)-2-((4-(trifluoromethyl)benzyl)amino)propanoate (4n)

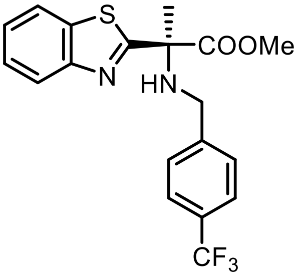

4n

Using sulfonamide ***S17*** according to **GP1**, provided **4n** (160 mg, 0.41 mmol, 81 %, *e.r.* = 99:1) as a colorless oil, after purification by column chromatography (SiO_2, hexane/EtOAc = 15:1 to 5:1).

R_f = 0.44 (hexane/EtOAc = 3:1)

$[\alpha]_D^{31}$ = +7.1° (*c* 1.0, $CHCl_3$).

^{1}H NMR (400 MHz, $CDCl_3$) δ (ppm): 8.04 (ddd, *J* = 8.2, 1.1, 0.7 Hz, 1H), 7.89 (ddd, *J* = 7.9, 1.2, 0.6 Hz, 1H), 7.60 (d, *J* = 8.3 Hz, 2H), 7.55 (d, *J* = 8.3 Hz, 2H), 7.48 (ddd, *J* = 8.3, 7.2, 1.3 Hz, 1H), 7.40 (ddd, *J* = 8.2, 7.3, 1.2 Hz, 1H), 3.89 (d, *J* = 13.1 Hz, 1H), 3.82 (d, *J* = 13.2 Hz, 1H), 3.79 (s, 3H), 2.91 (brs, 1H), 1.96 (s, 3H).

^{13}C {^{1}H, ^{19}F} NMR (101 MHz, $CDCl_3$) δ (ppm): 174.5, 172.8, 153.4, 143.9, 135.6, 129.6, 128.5, 126.2, 125.5, 125.5, 124.3, 123.5, 121.9, 66.4, 53.4, 47.6, 23.6

^{19}F {^{1}H} NMR (376 MHz, Chloroform-*d*) δ (ppm): -62.3 (s)

HRMS (ESI) *m/z:* $[M+H]^+$ calculated for $C_{19}H_{18}F_3N_2O_2S$, 395.1036; found, 395.1037.

HPLC (SA, Hexane : iPrOH = 90:10, 0.5 mL/min, 298 K, 220 nm): t_{R1} = 11.75 min, t_{R2} = 12.67 min.

7.1.1.14 Methyl (R)-2-(benzo[d]thiazol-2-yl)-2-((4-nitrobenzyl)amino)propanoate (4o)

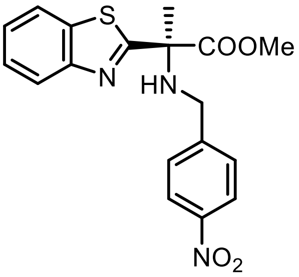

4o

Using sulfonamide ***S18*** according to **GP1**, provided **4o** (92 mg, 0.25 mmol, 41 %, *e.r.* = 65:35) as a colorless oil, after purification by column chromatography (SiO_2, hexane/EtOAc = 5:1 to 1:1).

R_f = 0.25 (hexane/EtOAc = 2:1)

[α]$_D$^{31} = +3.3° (*c* 1.0, $CHCl_3$).

^{1}H NMR (500 MHz, $CDCl_3$) δ (ppm): 8.22 – 8.18 (m, 2H), 8.10 – 8.06 (m, 1H), 8.06 – 8.01 (m, 1H), 7.64 – 7.59 (m, 2H), 7.49 (ddd, *J* = 8.4, 7.2, 1.3 Hz, 1H), 7.40 (ddd, *J* = 8.3, 7.2, 1.2 Hz, 1H), 3.95 (d, *J* = 13.9 Hz, 1H), 3.86 (d, *J* = 13.9 Hz, 1H), 3.80 (s, 3H), 1.96 (s, 3H).

^{13}C {^{1}H} NMR (126 MHz, $CDCl_3$) δ (ppm): 174.0, 172.7, 160.0, 153.3, 147.5, 135.5, 129.7, 128.9, 126.3, 125.6, 124.1, 123.8, 123.6, 121.9, 66.4, 53.4, 47.3, 23.7.

HRMS (ESI) *m/z:* $[M+H]^+$ calculated for $C_{18}H_{18}N_3O_4S$, 372.1013; found: 372.1015

HPLC (SA, Hexane : iPrOH = 90:10, 0.5 mL/min, 298 K, 220 nm): t_{R1} = 34.52 min, t_{R2} = 53.25 min.

7.1.1.15 Methyl (R)-2-(benzo[d]thiazol-2-yl)-2-((tert-butoxycarbonyl)amino)propanoate (4p)

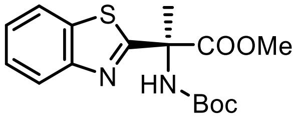

4p

Using sulfonamide ***S19*** according to **GP1**, provided **4p** (69 mg, 0.21 mmol, 82 %, *e.r.* = 98:2) as a colorless oil, after purification by column chromatography (SiO_2, hexane/EtOAc = 15:1 to 5:1).

R_f = 0.41 (hexane/EtOAc = 2:1)

[α]$_D$^{24} = -16.8° (*c* 1.2, $CHCl_3$).

^{1}H NMR (500 MHz, $CDCl_3$) δ (ppm): 8.03 (dd, *J* = 8.0, 0.8 Hz, 1H), 7.86 (dd, *J* = 8.0, 1.4 Hz, 1H), 7.48 (ddd, *J* = 15.4, 8.6, 1.3 Hz, 1H), 7.40 (ddd, *J* = 15.1, 8.7, 1.3 Hz, 1H), 6.56 (bs, 1H), 3.75 (s, 3H), 2.13 (s, 3H), 1.48 – 1.38 (m, 9H).

^{13}C {^{1}H} NMR (126 MHz, $CDCl_3$) δ (ppm): 171.3, 170.7, 152.4, 135.9, 126.4, 125.7, 123.6, 121.8, 80.6, 62.9, 53.7, 28.4, 24.9.

HRMS (ESI) *m/z:* $[M+H]^+$ calc. for $C_{16}H_{20}N_2O_4S$, 337.1217, found: 337.1215.

HPLC (OZ-H, Hexane : iPrOH = 95:5, 0.5 mL/min, 298 K, 220 nm): t_{R1} = 12.85 min, t_{R2} = 14.00 min.

7.1.1.16 Methyl (R)-2-amino-2-(benzo[d]thiazo-2-yl)propanoate (4q)

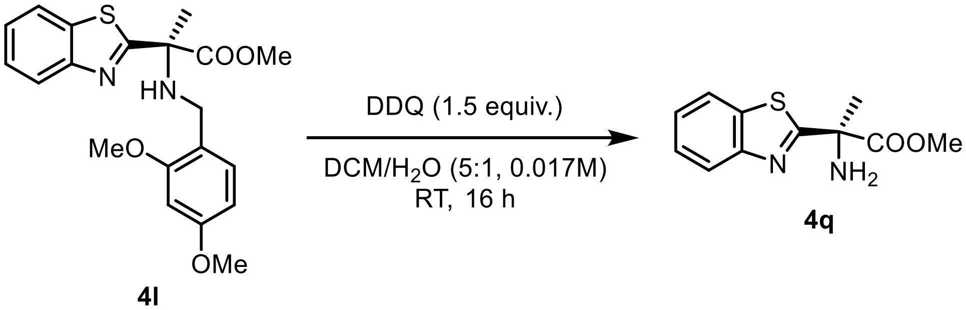

2,3-dichlor-5,6-dicyan-*p*-benzochinon (DDQ) (0.38 g, 1.65 mmol, 1.5 equiv.) was added to a solution of protected amine **4l** (0.43 g, 1.10 mmol, 1.0 equiv.) in mixture CH_2Cl_2/H_2O (5:1, 66 mL, 0.017M) and was stirred at room temperature overnight. The reaction was quenched by sat. aq. solution of $NaHCO_3$ (20 mL) and extracted with EtOAc (3x50 mL). The combined organic phases were washed with brine, dried over $MgSO_4$ and the solvents were removed under reduced pressure. The crude product was purified by gradient column chromatography (SiO_2; hexane/EtOAc = 10:1 – 2:1) to obtain **4q** (0.15 g, 0.62 mmol, 57 % yield) as colorless oil.

R_f = 0.15 (hexane/EtOAc = 2:1)

$[\alpha]_D^{24}$ = +26.8° (*c* 1.0, $CHCl_3$)

^1H NMR (400 MHz, $CDCl_3$) δ (ppm): 8.01 (ddd, *J* = 8.3, 1.0, 0.6 Hz, 1H), 7.91 – 7.84 (m, 1H), 7.47 (ddd, *J* = 8.3, 7.2, 1.3 Hz, 1H), 7.38 (ddd, *J* = 8.3, 7.3, 1.2 Hz, 1H), 3.79 (s, 3H), 2.40 (s, 2H), 1.89 (s, 3H).

$^{13}C\{^1H\}$ NMR (101 MHz, $CDCl_3$) δ (ppm): 175.9, 173.7, 153.5, 135.5, 126.2, 125.3, 123.5, 121.8, 61.9, 53.4, 27.4.

HRMS (ESI) *m/z:* $[M+H]^+$ calculated for $C_{11}H_{13}N_2O_2S$: 237.0692; found, 237.0691.

7.1.1.17 Methyl (S)-2-(benzylamino)-2-(thiazol-2-yl)propanoate (4r)

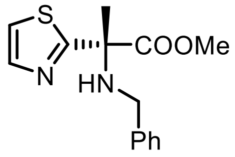

4r

Using sulfonamide ***S20*** according to **GP1**, provided **4r** (60 mg, 0.22 mmol, 31 %, *e.r.* = 7:93) as a colorless oil, after purification by column chromatography (SiO_2, hexane/EtOAc = 10:1 to 2:1).

R_f = 0.63 (hexane/EtOAc = 1:1)

$[\alpha]_D^{23}$ = +1.6° (*c* 1.0, $CHCl_3$).

^1H NMR (500 MHz, $CDCl_3$) δ (ppm): 7.77 (d, *J* = 3.3 Hz, 1H), 7.40 (d, *J* = 7.3 Hz, 2H), 7.34 (t, *J* = 7.5 Hz, 2H), 7.31 (d, *J* = 3.2 Hz, 1H), 7.31 – 7.22 (m, 1H), 3.78 (s, 3H), 3.75 (d, *J* = 12.5 Hz, 1H), 3.73 (d, *J* = 12.6 Hz, 1H), 1.88 (s, 3H).

$^{13}C\{^1H\}$ NMR (126 MHz, $CDCl_3$) δ (ppm): 174.1, 173.2, 142.8, 139.7, 128.6, 128.4, 127.3, 119.8, 65.9, 53.1, 48.2, 23.9.

HRMS (ESI) *m/z:* $[M+H]^+$ calculated for $C_{14}H_{16}N_2O_2S$, 277.1005; found, 277.1004.

HPLC (SA, Hexane : iPrOH = 90:10, 0.5 mL/min, 298 K, 220 nm): t_{R1} = 10.93 min, t_{R2} = 12.85 min.

7.1.1.18 Methyl (S)-2-(benzylamino)-2-(oxazol-2-yl)propanoate (4s)

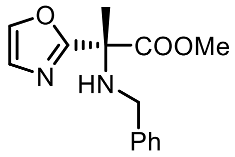

4s

Using sulfonamide ***S21*** according to **GP1**, provided **4s** (114 mg, 0.44 mmol, 63 %, *e.r.* = 9:91) as a colorless oil, after purification by column chromatography (SiO_2, hexane/EtOAc = 10:1 to 2:1).

$\mathbf{R_f}$ = 0.54 (hexane/EtOAc = 1:1)

$[\alpha]_D^{31}$ **=** -30.9° (*c* 1.0, $CHCl_3$).

^{1}H NMR (500 MHz, $CDCl_3$) δ (ppm): 7.64 (d, *J* = 0.7 Hz, 1H), 7.35 – 7.28 (m, 4H), 7.26 – 7.22 (m, 1H), 7.11 (d, *J* = 0.7 Hz, 1H), 3.77 (s, 3H), 3.72 (d, *J* = 12.1 Hz, 1H), 3.61 (d, *J* = 12.1 Hz, 1H), 1.88 (s, 3H).

^{13}C {^{1}H} NMR (126 MHz, $CDCl_3$) δ (ppm): 172.2, 163.7, 139.7, 139.4, 128.6, 128.5, 127.3, 62.8, 53.1, 48.4, 21.5.

HRMS (ESI) ***m/z:*** $[M+H]^+$ calculated for $C_{14}H_{16}N_2O_3$, 261.1234; found, 261.1233.

HPLC (SA, Hexane : iPrOH = 90:10, 0.5 mL/min, 298 K, 220 nm): t_{R1} = 11.07 min, t_{R2} = 11.72 min.

7.1.1.19 Methyl 2-(benzylamino)-2-(4-nitrophenyl)propanoate (4t)

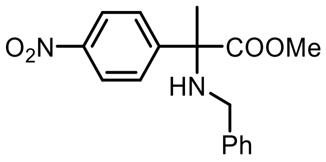

4t

Using sulfonamide ***S22*** according to **GP1**, provided **4t** (353 mg, 1.12 mmol, 75 %, *e.r.* = 57:43) as a colorless oil, after purification by column chromatography (SiO_2, hexane/EtOAc = 15:1 to 5:1).

$\mathbf{R_f}$ = 0.40 (hexane/EtOAc = 4:1)

^{1}H NMR (500 MHz, $CDCl_3$) δ (ppm): 8.22 – 8.19 (m, 2H), 7.73 – 7.70 (m, 2H), 7.38 – 7.33 (m, 4H), 7.30 – 7.27 (m, 1H), 3.78 (s, 3H), 3.67 (d, *J* = 12.2 Hz, 1H), 3.61 (d, *J* = 12.3 Hz, 1H), 2.29 (s, 1H), 1.74 (s, 3H).

^{13}C {^{1}H} NMR (126 MHz, $CDCl_3$) δ (ppm): 174.7, 150.6, 147.4, 139.9, 128.7, 128.3, 127.4, 127.3, 123.8, 65.9, 52.8, 48.4, 25.0.

HRMS (ESI) ***m/z:*** $[M+H]^+$ calculated for $C_{17}H_{19}N_2O_4$, 315.1339; found, 315.1338.

HPLC (SA, Hexane : iPrOH = 90:10, 0.5 mL/min, 298 K, 220 nm): t_{R1} = 13.73 min, t_{R2} = 16.52 min.

7.1.1.20 Methyl (S)-2-(benzylamino)-2-(pyrimidin-2-yl)propanoate (4u)

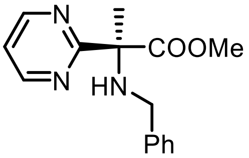

4u

Using sulfonamide ***S23*** according to **GP1**, provided **4u** (90 mg, 0.33 mmol, 66 %, *e.r.* = 92:8) as a colorless oil, after purification by column chromatography (SiO_2, hexane/EtOAc = 15:1 to 1:1).

R_f = 0.35 (hexane/EtOAc = 1:1)

$[\alpha]_D^{23}$ = +3.2° (*c* 1.0, $CHCl_3$).

^1H NMR (400 MHz, $CDCl_3$) δ (ppm): 8.73 (d, *J* = 4.9 Hz, 2H), 7.37 (d, *J* = 7.4 Hz, 2H), 7.30 (t, *J* = 7.5 Hz, 2H), 7.23 (t, *J* = 7.3 Hz, 1H), 7.20 (t, *J* = 4.9 Hz, 1H), 3.75 – 3.69 (m, 5H), 3.05 (brs, 1H), 1.89 (s, 3H).

^{13}C {^1H} NMR (126 MHz, $CDCl_3$) δ (ppm): 173.2, 168.7, 157.4, 138.6, 128.9, 128.7, 127.6, 120.0, 68.7, 52.9, 48.4, 20.8.

HRMS (ESI) *m/z:* $[M+H]^+$ calculated for $C_{15}H_{17}N_3O_2$, 272.1394; found, 272.1396.

HPLC (SA, Hexane : iPrOH = 90:10, 0.5 mL/min, 298 K, 220 nm): t_{R1} = 12.03 min, t_{R2} = 13.20 min.

7.1.1.21 (S)-2-(benzo[d]thiazol-2-yl)-2-(benzylamino)-N-methoxy-N-methylpropanamide (1a)

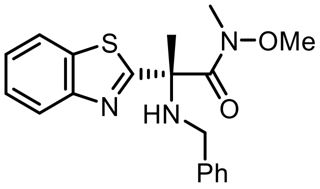

1a

Using sulfonamide ***S24*** according to **GP1**, provided **1a** (1020 mg, 2.87 mmol, 97 %, *e.r.* = 99:1) as a colorless oil, after purification by column chromatography (SiO_2, CH_2Cl_2/acetone = 200:1 to 50:1).

R_f = 0.41 (cyclohexane/EtOAc = 2:1)

$[\alpha]_D^{21}$ = -7.6° (*c* 1.02, $CHCl_3$).

^1H NMR (500 MHz, $CDCl_3$) δ (ppm): 8.01 – 7.99 (m, 1H), 7.90 – 7.88 (m, 1H), 7.45 (ddd, *J* = 8.3, 7.3, 1.3 Hz, 1H), 7.42 – 7.40 (m, 2H), 7.38 – 7.34 (m, 3H), 7.29 – 7.27 (m, 1H), 3.97 (d, *J* = 12.5 Hz, 1H), 3.74 (d, *J* = 12.7 Hz, 1H), 3.21 (s, 3H), 2.88 (s, 3H), 1.81 (s, 3H).

^{13}C {^1H} NMR (126 MHz, $CDCl_3$) δ (ppm): 178.4, 172.2, 153.3, 140.0, 134.8, 128.6, 128.3, 127.3, 126.1, 124.9, 123.0, 121.8, 66.2, 59.6, 47.9, 33.2, 24.8.

HRMS (ESI) ***m/z:*** $[M+H]^+$ calculated for $C_{19}H_{22}N_3O_2S$, 356.1427; found, 356.1425.

HPLC (OD-H, Hexane : iPrOH = 90:10, 0.5 mL/min, 298 K, 220 nm): t_{R1} = 12.33 min, t_{R2} = 12.90 min.

7.1.1.22 tert-butyl (R)-2-(benzo[d]thiazol-2-yl)-2-(benzylamino)propanoate (S1)

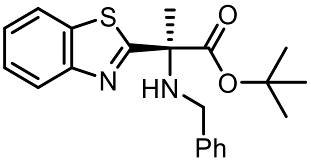

S1

Using sulfonamide ***S25*** according to **GP1**, provided **S1** (710 mg, 1.93 mmol, 96 %, *e.r.* = 1:99) as a colorless oil, after purification by column chromatography (SiO_2, cyclohexane/EtOAc = 20:1 to 15:1).

R_f = 0.58 (cyclohexane/EtOAc = 4:1)

$[\alpha]_D^{23}$ = +0.5° (*c* 1.0, $CHCl_3$).

^{1}H NMR (400 MHz, $CDCl_3$) δ (ppm): 8.03 (ddd, *J* = 8.2, 1.2, 0.7 Hz, 1H), 7.89 (ddd, *J* = 7.9, 1.3, 0.7 Hz, 1H), 7.49 – 7.32 (m, 6H), 7.29 – 7.25 (m, 1H), 3.79 (d, *J* = 12.1 Hz, 1H), 3.75 (d, *J* = 12.4 Hz, 1H), 2.78 (s, 1H), 1.92 (s, 3H), 1.45 (s, 9H).

^{13}C {^{1}H} NMR (101 MHz, $CDCl_3$) δ (ppm): 175.5, 171.4, 153.4, 140.0, 135.7, 128.6, 128.4, 127.3, 125.9, 125.1, 123.4, 121.8, 82.8, 66.9, 48.1, 28.0, 23.3.

HRMS (ESI) ***m/z:*** $[M+H]^+$ calc. for $C_{21}H_{25}N_2O_2S$, 369.1631; found, 369.1631.

HPLC (OD-H, Hexane : iPrOH = 95:5, 0.5 mL/min, 298 K, 220 nm): t_{R1} = 8.55 min, t_{R2} = 9.23 min.

7.1.2 Synthesis of Hydroxylamines 5

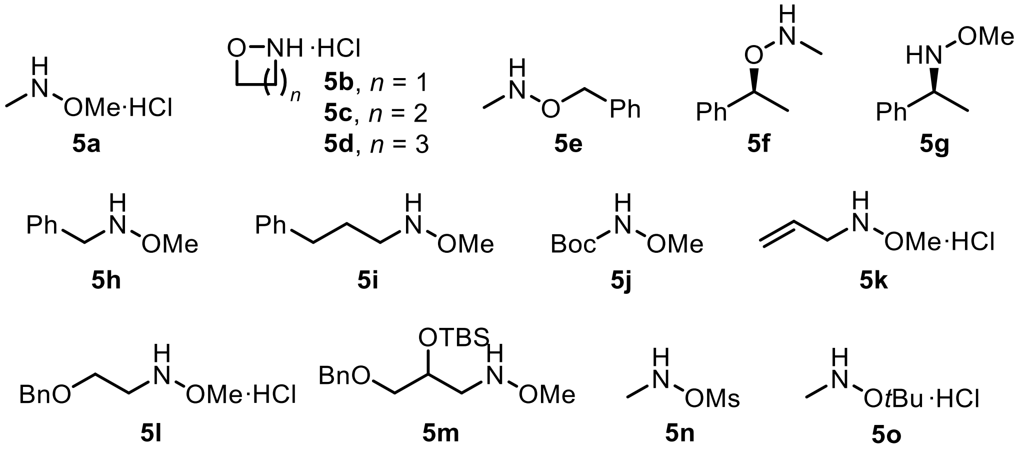

Hydroxylamine 5a is commercially available and was used as received without further purification.

7.1.2.1 Synthesis of 1,2-oxazetidine hydrochloride (5b)

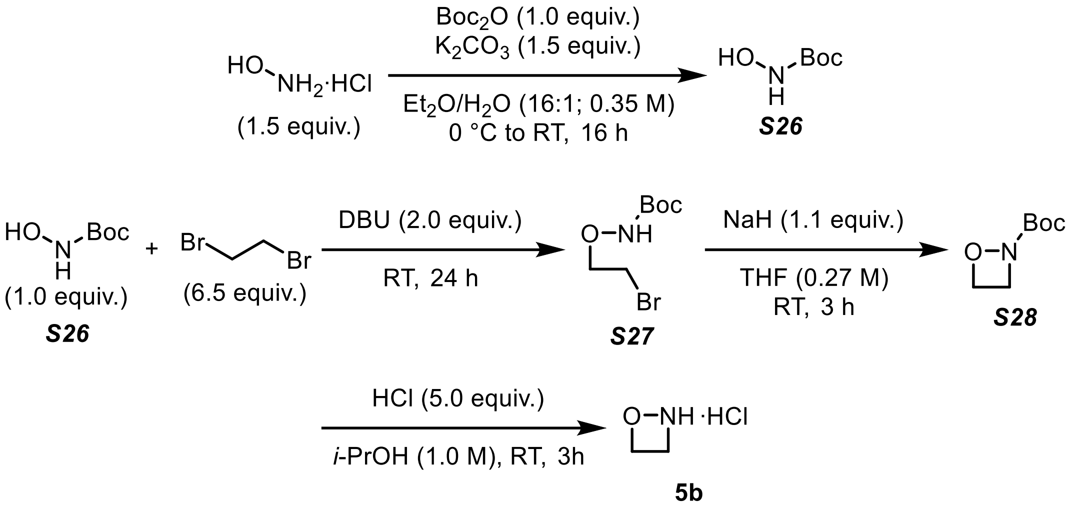

A suspension of hydroxylamine hydrochloride (2.08 g, 30.0 mmol, 1.5 equiv) and K_2CO_3 (4.15 g, 30.0 mmol, 1.5 equiv) in Et_2O (40 mL, 0.5 M) and water (5.0 mL, 4.0 M) was stirred for 1 h at rt with evolution of CO_2. A solution of Boc_2O (4.41 g, 20.0 mmol, 1.0 equiv) in Et_2O (40 mL, 0.5 M; overall concentration 0.35 M) was then added dropwise at 0 °C, and the resulting suspension was stirred for 16 h at rt. The reaction was quenched with water (20 mL), and the aqueous phase was extracted with Et_2O (3 × 60 mL). The combined organic layers were washed with brine (50 mL), dried over Na_2SO_4, and concentrated under reduced pressure. The crude product was used as received without further purification to afford N-Boc-protected hydroxylamine **S26** (quant.) as a white solid. Spectroscopic data were consistent with those reported in the literature.[17]

R_f = 0.21 (cyclohexane/EtOAc = 2:1)

^{1}H NMR (400 MHz, $CDCl_3$) δ (ppm): 7.14 (brs, 1H), 1.46 (s, 9H).

^{13}C {^{1}H} NMR (101 MHz, $CDCl_3$) δ (ppm): 159.0, 82.2, 28.3.

Hydroxylamine **S26** (2.66 g, 20.0 mmol, 1.0 equiv) was stirred in 1,2-dibromoethane (4.48 mL, 52.0 mmol, 2.6 equiv) with DBU (2.44 mL, 16.0 mmol, 2.0 equiv) at rt for 24 h. The reaction mixture was diluted with CH_2Cl_2 (10 mL), poured into 1.0 M HCl (10 mL), and extracted with CH_2Cl_2 (3 × 20 mL). The combined organic layers were washed with brine (50 mL), dried over Na_2SO_4, and concentrated under reduced pressure to afford a crude mixture of hydroxylamines **S27** and **S28**, which was used directly in the next step.

The crude reaction mixture containing **S27** was dissolved in dry THF (30.0 mL, 0.27 M), and NaH (60% dispersion in mineral oil, 0.350 g, 8.8 mmol, 1.1 equiv) was added slowly under an argon atmosphere. After 3 h, the reaction was quenched with 1.0 M HCl (10 mL), and the aqueous phase was extracted with EtOAc (3 × 20 mL). The combined organic layers were washed with brine (50 mL), dried over Na_2SO_4, and concentrated under reduced pressure. Purification of the crude residue by gradient column chromatography (SiO_2, cyclohexane/EtOAc = 10:1 → 5:1) afforded *N*-Boc-protected oxazetidine **S28** (0.480 g,

3.02 mmol, 38% over 2 steps) as a clear oil. Spectroscopic data were consistent with those reported in the literature.[18]

^{1}H NMR (400 MHz, $CDCl_3$) δ (ppm): 4.86 (t, *J* = 7.9 Hz, 2H), 4.55 (t, *J* = 8.1 Hz, 2H), 1.52 (s, 9H).

^{13}C {^{1}H} NMR (101 MHz, $CDCl_3$) δ (ppm): 161.4, 83.1, 67.6, 53.9, 28.2.

HRMS (APCI) ***m/z:*** $[M+H]^+$ calculated for $C_7H_{14}NO_3$, 160.0968; found, 160.0966.

Hydroxylamine **S28** (0.318 g, 2.0 mmol, 1.0 equiv) was dissolved in *i*-PrOH (2 mL, 1.0 M), and 35% aq HCl (0.883 mL, 5.0 equiv) was added slowly. The reaction mixture was stirred until complete consumption of the starting material (TLC, 2–3 h). The mixture was concentrated to dryness and co-evaporated twice with 1:1 MeOH/toluene (10 mL each). The resulting residue was dried overnight by lyophilization to afford oxazetidine hydrochloride **5b** (0.19 g, quant.) as a purple solid.

^{1}H NMR (400 MHz, CD_3OD) δ (ppm): 4.86 (broad s, 2H), 3.96 – 3.93 (m, 2H), 3.64 – 3.61 (m, 2H).

^{13}C {^{1}H} NMR (101 MHz, CD_3OD) δ (ppm): 53.6, 37.5.

HRMS (APCI) ***m/z:*** $[M+H]^+$ calculated for C_2H_7ClNO, 96.0211; found, 96.0214.

7.1.2.2 Synthesis of isoxazolidine hydrochloride (5c)

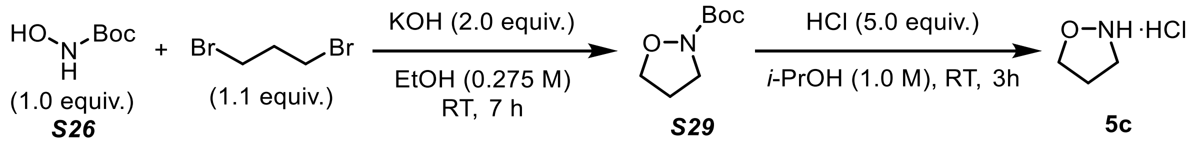

Protected hydroxylamine **S26** (2.66 g, 20.0 mmol, 1.0 equiv) and 1,3-dibromopropane (2.23 mL, 22.0 mmol, 1.1 equiv) were dissolved in EtOH (72.7 mL, 0.275 M), and KOH (2.24 g, 40.0 mmol, 2.0 equiv) was added. The solution was stirred at rt for 7 h, the solids were filtered off, and the volatiles were removed under reduced pressure. Water (30 mL) was added to the resulting residue, and the aqueous phase was extracted with CH_2Cl_2 (3 × 50 mL). The combined organic layers were washed with brine (50 mL), dried over Na_2SO_4, and concentrated under reduced pressure. Purification of the crude product by gradient column chromatography (SiO_2, cyclohexane/EtOAc = 15:1 → 5:1) afforded N-Boc-protected isoxazolidine **S29** (1.95 g, 11.3 mmol, 56%) as a clear oil. The analytical data are consistent with those reported in the literature.[19]

R_f = 0.50 (cyclohexane/EtOAc = 2:1)

^{1}H NMR (400 MHz, $CDCl_3$) δ (ppm): 3.89 (t, *J* = 7.1 Hz, 2H), 3.61 – 3.57 (m, 2H), 2.20 (pd, *J* = 7.2, 1.0 Hz, 2H), 1.48 (s, 9H).

^{13}C {^{1}H} NMR (101 MHz, $CDCl_3$) δ (ppm): 157.8, 81.9, 68.5, 46.9, 28.3, 28.0.

Hydroxylamine **S29** (1.91 g, 11.0 mmol, 1.0 equiv) was dissolved in *i*-PrOH (11 mL, 1.0 M), and 35% aq HCl (4.9 mL, 5.0 equiv) was added slowly. The reaction mixture was stirred until complete consumption of the starting material (TLC, 2–3 h). The mixture was concentrated to dryness and co-evaporated twice with 1:1 MeOH/toluene (20 mL each). The resulting residue was dried overnight by lyophilization to afford isoxazolidine hydrochloride **5c** (0.53 g, 4.84 mmol, 44%) as a white solid.

^{1}H NMR (400 MHz, CD_3OD) δ (ppm): 4.27 (t, *J* = 6.8 Hz, 2H), 3.62 (t, *J* = 7.3 Hz, 2H), 2.52 (p, *J* = 7.0 Hz, 2H).

^{13}C {^{1}H} NMR (101 MHz, CD_3OD) δ (ppm): 72.5, 47.7, 28.8.

HRMS (APCI) *m/z:* $[M\text{-}Cl]^+$ calculated for C_3H_8NO, 74.0600; found, 74.0604.

7.1.2.3 Synthesis of 1,2-oxazinane hydrochloride (5d)

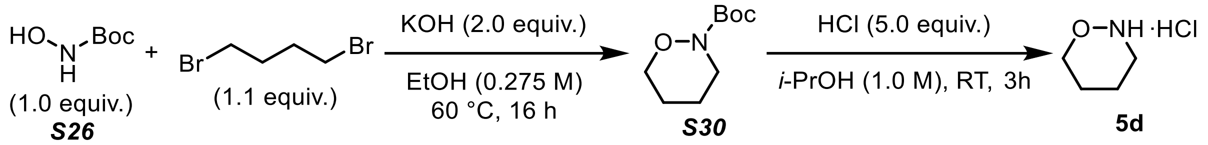

Protected hydroxylamine **S23** (1.07 g, 8.0 mmol, 1.0 equiv) and 1,3-dibromobutane (1.05 mL, 8.8 mmol, 1.1 equiv) were dissolved in EtOH (29.1 mL, 0.275 M), and KOH (0.898 g, 16.0 mmol, 2.0 equiv) was added. The solution was stirred at 60 °C for 16 h, the solids were filtered off, and the volatiles were removed under reduced pressure. Water (30 mL) was added to the resulting residue, and the aqueous phase was extracted with CH_2Cl_2 (3 × 30 mL). The combined organic layers were washed with brine (30 mL), dried over Na_2SO_4, and concentrated under reduced pressure. Purification of the crude product by gradient column chromatography (SiO_2, cyclohexane/EtOAc = 10:1 → 5:1) afforded *N*-Boc-protected oxazinane **S30** (0.998 g, 5.33 mmol, 67%) as a clear oil. The analytical data are consistent with those reported in the literature.[19]

R_f = 0.66 (cyclohexane/EtOAc = 2:1)

^{1}H NMR (400 MHz, $CDCl_3$) δ (ppm): 3.93 – 3.91 (m, 2H), 3.59 (d, *J* = 5.4 Hz, 2H), 1.79 – 1.71 (m, 2H), 1.68 – 1.62 (m, 2H), 1.47 (s, 9H).

^{13}C {^{1}H} NMR (101 MHz, $CDCl_3$) δ (ppm): 155.2, 81.3, 71.5, 46.9, 28.4, 24.5, 22.7.

Hydroxylamine **S30** (0.936 g, 5.0 mmol, 1.0 equiv) was dissolved in *i*-PrOH (5.0 mL, 1.0 M), and 35% aq HCl (2.21 mL, 5.0 equiv) was added slowly. The reaction mixture was stirred until complete consumption of the starting material (TLC, 2–3 h). The mixture was concentrated to dryness and co-evaporated twice with 1:1 MeOH/toluene (20 mL each). The resulting residue

was dried overnight by lyophilization to afford oxazinane hydrochloride **5d** (0.606 g, 4.90 mmol, 98%) as a white solid.

^{1}H NMR (400 MHz, CD_3OD) δ (ppm): 4.28 – 4.26 (m, 2H), 3.46 – 3.43 (m, 2H), 2.03 – 1.97 (m, 2H), 1.92 – 1.86 (m, 2H).

^{13}C {^{1}H} NMR (101 MHz, CD_3OD) δ (ppm): 73.1, 47.6, 23.3, 20.1.

HRMS (APCI) *m/z:* $[M\text{-}Cl]^+$ calculated for $C_4H_{10}NO$, 88.0757; found, 88.0760.

7.1.2.4 Synthesis of O-benzyl-N-methylhydroxylamine (5e)

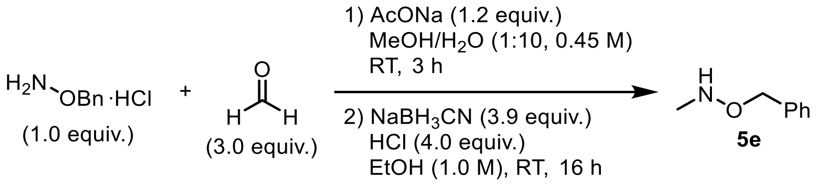

In a round-bottom flask, AcONa (0.295 g, 3.6 mmol, 1.2 equiv) and *O*-benzylhydroxylamine hydrochloride (0.479 g, 3.0 mmol, 1.0 equiv) were dissolved in MeOH/H_2O (1:10; 6.6 mL, 0.45 M). To the resulting suspension, 36% aq formaldehyde (0.70 mL, 9.0 mmol, 3.0 equiv) was added slowly. The reaction mixture was stirred at rt for 3 h and then extracted with CH_2Cl_2 (6 × 20 mL). The combined organic layers were washed with sat. $NaHCO_3$ (20 mL), dried over $MgSO_4$, and concentrated under reduced pressure to afford the crude oxime, which was used directly in the next step.

The crude oxime was dissolved in EtOH (3 mL, 1.0 M), and $NaBH_3CN$ (0.735 g, 11.7 mmol, 3.9 equiv) was added portionwise at rt. Subsequently, 35% aq HCl (1.0 mL, 4.0 equiv) was added slowly. The reaction mixture was stirred for 16 h. A saturated solution of Na_2CO_3 was added, and the aqueous phase was extracted with CH_2Cl_2 (3 × 30 mL). The combined organic layers were washed with water (30 mL) and brine (30 mL), dried over Na_2SO_4, and concentrated under reduced pressure. Purification of the crude residue by gradient column chromatography (SiO_2, cyclohexane/EtOAc = 15:1 → 5:1) afforded **5e** (159 mg, 1.16 mmol, 39%) as a clear oil. The analytical data are consistent with those reported in the literature.[20]

R_f = 0.40 (cyclohexane/EtOAc = 3:1)

^{1}H NMR (400 MHz, $CDCl_3$) δ (ppm): 7.40 – 7.28 (m, 5H), 5.51 (brs, 1H), 4.73 (s, 2H), 2.74 (s, 3H).

^{13}C {^{1}H} NMR (101 MHz, $CDCl_3$) δ (ppm): 138.1, 128.5, 128.4, 127.9, 75.8, 39.4.

HRMS (ESI) *m/z:* $[M+H]^+$ calculated for $C_8H_{12}NO$, 138.0913; found, 138.0911.

7.1.2.5 Synthesis of (S)-N-methyl-O-(1-phenylethyl)hydroxylamine (5f)

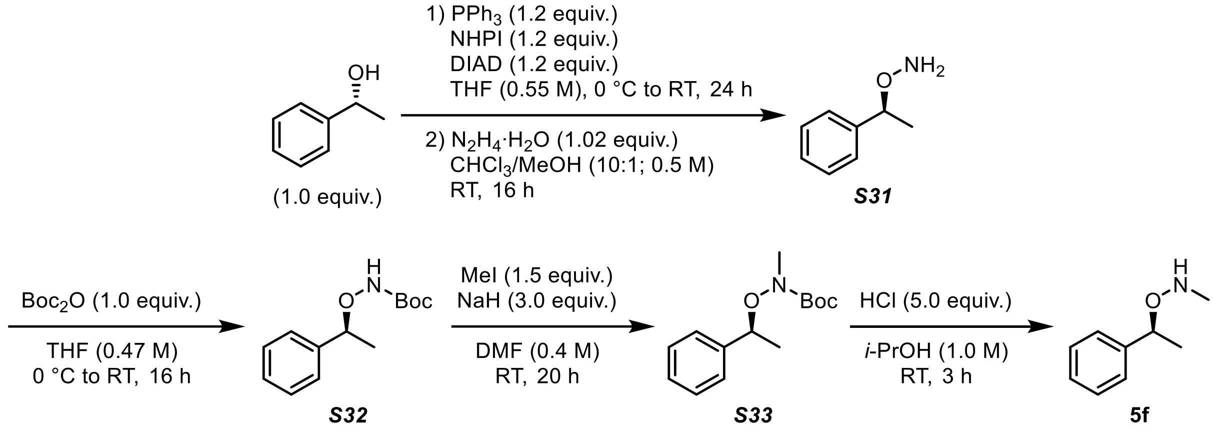

To a solution of (R)-1-phenylethanol (1.26 mL, 10.0 mmol, 1.0 equiv) in THF (18.2 mL, 0.55 M), PPh_3 (3.18 g, 12.0 mmol, 1.2 equiv) was added at rt, followed by *N*-hydroxyphthalimide (NHPI, 2.02 g, 12.0 mmol, 1.2 equiv). DIAD (2.38 mL, 12.0 mmol, 1.2 equiv) was then added at 0 °C, and the mixture was stirred at rt for 24 h. The reaction mixture was concentrated *in vacuo*, and the residue was dissolved in $CHCl_3$/MeOH (10:1; 20.0 mL, 0.5 M). Hydrazine hydrate (0.773 mL, 10.2 mmol, 1.02 equiv) was added, and the solution was stirred at rt for 16 h. The resulting suspension was filtered, and the filtrate was evaporated *in vacuo*. The residue was dissolved in 2 M aq HCl (20 mL), washed with CH_2Cl_2 (2 × 10 mL), and neutralized to pH 9 with K_2CO_3. The aqueous phase was extracted with CH_2Cl_2 (2 × 20 mL). The combined organic layers were dried over Na_2SO_4 and concentrated under reduced pressure. The crude product was used without further purification to afford hydroxylamine **S31** (1.32 g, 9.65 mmol, 97%) as a colorless oil. The analytical data were consistent with those reported in the literature.[21]

R_f = 0.29 (cyclohexane/EtOAc = 4:1)

$[\alpha]_D^{21}$ = -96.1° (*c* 1.0, MeOH); lit.[21] $[\alpha]_D^{21}$ = -98.5° (*c* 1.0, MeOH)

^1H NMR (500 MHz, $CDCl_3$) δ (ppm): 7.39 – 7.28 (m, 5H), 5.22 (brs, 2H), 4.66 (q, *J* = 6.6 Hz, 1H), 1.44 (d, *J* = 6.7 Hz, 3H).

^{13}C {^1H} NMR (126 MHz, $CDCl_3$) δ (ppm): 143.1, 128.6, 127.8, 126.4, 82.9, 21.9.

A solution of Boc_2O (1.98 g, 9.0 mmol, 1.0 equiv) in THF (1.3 mL, 7.0 M) was added to amine **S31** (1.24 g, 9.0 mmol, 1.0 equiv) in THF (18 mL, 0.5 M; overall concentration 0.47 M) at 0 °C. After 16 h, water (40 mL) was added until a clear two-phase system was obtained, and the layers were separated. The aqueous phase was extracted with Et_2O (3 × 50 mL). The combined organic layers were washed with brine (30 mL), dried over Na_2SO_4, and concentrated under reduced pressure. Purification of the crude residue by gradient column chromatography (SiO_2, cyclohexane/EtOAc = 15:1 → 6:1) afforded **S32** (1.941 g, 8.18 mmol, 91%) as a clear oil.

R_f = 0.47 (cyclohexane/EtOAc = 4:1)

$[\alpha]_D^{22}$ = -114° (*c* 1.0, MeOH).

^{1}H NMR (500 MHz, CDCl$_3$) δ (ppm): 7.39 – 7.30 (m, 5H), 6.94 (s, 1H), 4.91 (q, *J* = 6.5 Hz, 1H), 1.53 (d, *J* = 6.5 Hz, 3H), 1.45 (s, 9H).

^{13}C {^{1}H} NMR (126 MHz, CDCl$_3$) δ (ppm): 156.7, 141.6, 128.7, 128.3, 126.9, 83.6, 81.7, 28.3, 21.0.

HRMS (ESI) *m/z:* [M-H]$^-$ calculated for $C_{13}H_{18}NO_3$, 236.1281; found, 236.1285.

NaH (60% dispersion in mineral oil, 0.720 g, 18.0 mmol, 3.0 equiv) was added under an argon atmosphere to a solution of amine **S32** (1.42 g, 6.0 mmol, 1.0 equiv) in DMF (15.0 mL, 0.4 M), followed 15 min later by MeI (0.566 mL, 9.0 mmol, 1.5 equiv). After 20 h, the reaction was quenched with water (50 mL), and the aqueous phase was extracted with Et_2O (3 × 50 mL). The combined organic layers were washed with brine (30 mL), dried over Na_2SO_4, and concentrated under reduced pressure. Purification of the crude residue by gradient column chromatography (SiO_2, cyclohexane/EtOAc = 15:1 → 10:1) afforded **S33** (0.910 g, 3.62 mmol, 60%) as a clear oil.

R$_f$ = 0.63 (cyclohexane/EtOAc = 4:1)

[α]$_D^{22}$ = -143° (*c* 1.0, MeOH).

^{1}H NMR (500 MHz, CDCl$_3$) δ (ppm): 7.40 – 7.29 (m, 5H), 4.93 (q, *J* = 6.6 Hz, 1H), 2.89 (s, 3H), 1.53 (d, *J* = 6.6 Hz, 3H), 1.48 (s, 9H).

^{13}C {^{1}H} NMR (126 MHz, CDCl$_3$) δ (ppm): 157.7, 141.5, 128.4, 128.3, 127.5, 82.2, 81.3, 38.1, 28.4, 20.5.

HRMS (ESI) *m/z:* [M+H]$^+$ calculated for $C_{14}H_{22}NO_3$, 252.1594; found, 252.1591.

Hydroxylamine **S33** (0.779 g, 3.1 mmol, 1.0 equiv) was dissolved in *i*-PrOH (3.1 mL, 1.0 M), and 35% aq HCl (1.37 mL, 5.0 equiv) was added slowly. The reaction mixture was stirred until complete consumption of the starting material (TLC, 2–3 h). The mixture was concentrated to dryness, and the resulting residue was dissolved in water (15 mL) and washed with CH_2Cl_2 (2 × 10 mL). The aqueous phase was neutralized with sat. $NaHCO_3$ and extracted with CH_2Cl_2 (3 × 40 mL). The combined organic layers were dried over Na_2SO_4 and concentrated under reduced pressure to afford hydroxylamine **5f** (0.40 g, 2.65 mmol, 85%) as a colorless oil.

R$_f$ = 0.53 (cyclohexane/EtOAc = 2:1)

[α]$_D^{23}$ = -103° (*c* 1.0, MeOH).

^{1}H NMR (500 MHz, CDCl$_3$) δ (ppm): 7.38 – 7.27 (m, 5H), 5.33 (brs, 1H), 4.76 (q, *J* = 6.6 Hz, 1H), 2.69 (s, 3H), 1.43 (d, *J* = 6.6 Hz, 3H).

^{13}C {^{1}H} NMR (126 MHz, CDCl$_3$) δ (ppm): 143.9, 128.5, 127.6, 126.3, 80.6, 39.6, 22.2.

HRMS (ESI) *m/z:* [M+H]$^+$ calculated for $C_9H_{14}NO$, 152.1070; found, 152.1068.

7.1.2.6 *Synthesis of (S)-O-methyl-N-(1-phenylethyl)hydroxylamine (5g)*

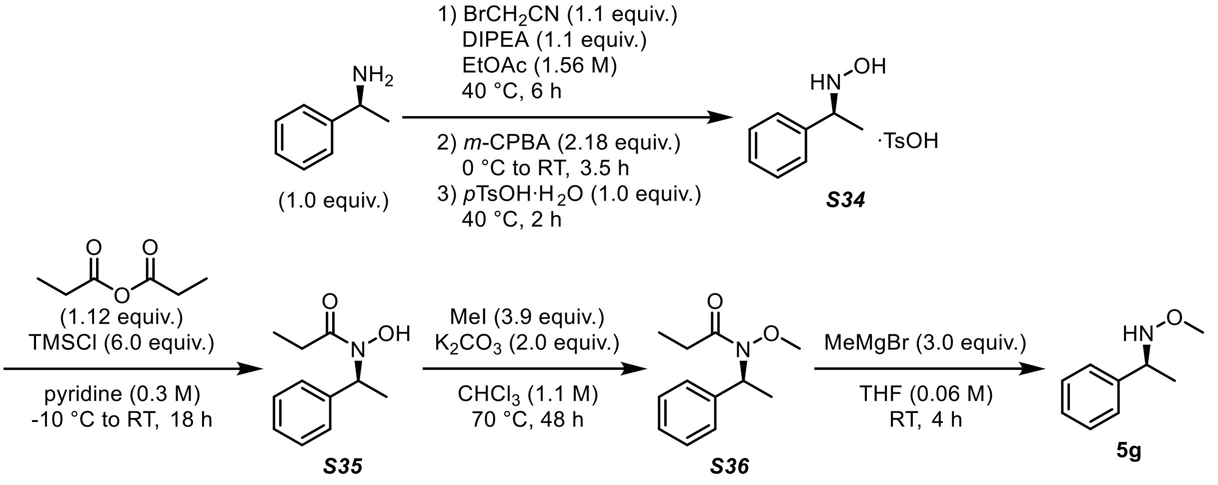

Following a reported procedure,[22] (S)-1-phenylethylamine (5.16 mL, 39.0 mmol, 1.0 equiv) and DIPEA (7.55 mL, 42.9 mmol, 1.1 equiv) were dissolved in EtOAc (25.0 mL, 1.56 M), and the solution was heated to 40 °C (heating block). Bromoacetonitrile (3.08 mL, 42.9 mmol, 1.1 equiv) was added dropwise over 2 h. At the end of the addition, an EtOAc line wash (2.5 mL) was added, and the reaction mixture was stirred for an additional 3 h. The suspension was cooled to 20 °C and washed with water (7.5 mL) to afford an ethyl acetate solution of the secondary amine.

The ethyl acetate solution of the amine (as a crude material from the previous reaction) was cooled to 0 °C, and a freshly prepared solution of *m*-CPBA (19.1 g, 85.0 mmol, 2.18 equiv) in EtOAc (25.0 mL, 1.56 M) was added slowly, keeping the internal temperature below 5 °C (typically over 2 h). After the addition, an EtOAc line wash (5.0 mL) was added, and the reaction mixture was warmed to rt. The mixture was washed with sat. aq $NaHCO_3$ (3 × 25 mL) and brine (25 mL) to afford an ethyl acetate solution of the nitrone.

The ethyl acetate solution of the nitrone (previous reaction) was heated to 40 °C (heating block), and *p*-TsOH·H_2O (7.57 g, 39.0 mmol, 1.0 equiv) and additional EtOAc (5 mL, 7.8 M) were added. The reaction mixture was stirred for 3 h **[CAUTION: potential release of HCN in the headspace; ensure adequate ventilation]**. The mixture was then cooled to 0 °C, and the hydroxylamine salt was isolated by filtration and washed with EtOAc and dry Et_2O to afford hydroxylamine **S34** (8.98 g, 29.0 mmol, 74% over 3 steps) as a colorless solid.

The analytical data are consistent with those reported in the literature.[22]

R_f = 0.29 (cyclohexane/EtOAc = 4:1)

^1H NMR (400 MHz, CD_3OD) δ (ppm): 7.73 – 7.70 (m, 2H), 7.50 – 7.43 (m, 5H), 7.25 – 7.22 (m, 2H), 4.53 (q, *J* = 6.9 Hz, 1H), 2.37 (s, 3H), 1.68 (d, *J* = 6.9 Hz, 3H).

^{13}C {^1H} NMR (101 MHz, CD_3OD) δ (ppm): 143.4, 141.8, 135.5, 130.9, 130.3, 129.8, 129.4, 126.9, 62.9, 21.3, 16.0.

Following a reported procedure,[23] TMSCl (6.26 mL, 48.0 mmol, 6.0 equiv) was added dropwise to a stirred solution of hydroxylamine salt **S34** (2.48 g, 8.0 mmol, 1.0 equiv) in dry pyridine (26.7 mL, 0.30 M) at –10 °C. After 2 h at –10 °C, propionic anhydride (1.15 mL, 8.96 mmol, 1.12 equiv) was added. The reaction mixture was allowed to warm to rt and stirred for 18 h. The residue was acidified with 2 M HCl to pH 2 and extracted with EtOAc (2 × 25 mL). The combined organic extracts were washed successively with 0.5 M HCl (25 mL), water (45 mL), and sat. aq $NaHCO_3$ (25 mL), dried over $MgSO_4$, filtered, and concentrated *in vacuo*. Recrystallization from pentane/EtOAc afforded **S35** (1.25 g, 6.47 mmol, 81%) as an off-white to pale-yellow solid.

$\mathbf{R_f}$ = 0.51 (hexane/EtOAc = 1:1)

^{1}H NMR (400 MHz, DMSO-*d*$_6$) δ (ppm): 9.40 (s, 1H), 7.34 – 7.22 (m, 5H), 5.60 (q, *J* = 7.1 Hz, 1H), 2.38 (q, *J* = 7.5 Hz, 2H), 1.45 (d, *J* = 7.1 Hz, 3H), 0.98 (t, *J* = 7.5 Hz, 3H).

^{13}C {^{1}H} NMR (101 MHz, DMSO-*d*$_6$) δ (ppm): 173.6, 141.5, 128.1, 127.0, 126.9, 52.9, 25.3, 17.2, 8.8.

HRMS (APCI) *m/z:* $[M\text{-}H]^-$ calculated for $C_{11}H_{14}NO_2$, 192.1019; found, 192.1016.

In a pressure vial, a solution of **S35** (1.06 g, 5.5 mmol, 1.0 equiv), MeI (1.36 mL, 21.6 mmol, 3.9 equiv), and K_2CO_3 (1.52 g, 11.0 mmol, 2.0 equiv) in $CHCl_3$ (5.0 mL, 1.1 M) was refluxed for 48 h. The solvent was removed under reduced pressure, and the residue was redissolved in Et_2O, passed through a small pad of silica, washed with Et_2O (3 × 30 mL), and concentrated *in vacuo* to afford **S36** (1.05 g, 5.06 mmol, 92%) as a yellow oil.

The analytical data are consistent with those reported in the literature.[23]

$\mathbf{R_f}$ = 0.71 (hexane/EtOAc = 1:1)

$[\alpha]_D^{26}$ = -90.8° (*c* 1.07, $CHCl_3$).

^{1}H NMR (400 MHz, $CDCl_3$) δ (ppm): 7.42 – 7.40 (m, 2H), 7.35 – 7.25 (m, 3H), 5.68 (q, *J* = 7.1 Hz, 1H), 3.43 (s, 3H), 2.53 – 2.35 (m, 2H), 1.60 (d, *J* = 7.0 Hz, 3H), 1.13 (t, *J* = 7.5 Hz, 3H).

^{13}C {^{1}H} NMR (101 MHz, $CDCl_3$) δ (ppm): 176.7, 140.7, 128.5, 127.9, 127.7, 64.9, 55.5, 26.2, 16.4, 8.8.

HRMS (ESI) *m/z:* $[M+H]^+$ calculated for $C_{12}H_{18}NO_2$, 208.1332; found, 208.1329.

MeMgBr (3 M in Et_2O; 2.0 mL, 6.0 mmol, 3.0 equiv) was added dropwise to a solution of amide **S36** (0.415 g, 2.0 mmol, 1.0 equiv) in THF (34.0 mL, 0.06 M) at rt. After stirring for 4 h, 10% HCl (5 mL) was added, and the mixture was concentrated *in vacuo*. The residue was basified with sat. aq $NaHCO_3$, extracted with Et_2O (3 × 40 mL), dried over Na_2SO_4, filtered, and concentrated

in vacuo. Purification of the crude product by gradient column chromatography (SiO_2, hexane/EtOAc = 20:1 → 10:1) afforded **5g** (0.21 g, 1.4 mmol, 70%) as a clear oil.

The analytical data are consistent with those reported in the literature.[24]

R$_f$ = 0.46 (hexane/EtOAc = 5:1)

$[\alpha]_D^{23}$ = -35.3° (*c* 1.15, $CHCl_3$).

^{1}H NMR (500 MHz, $CDCl_3$) δ (ppm): 7.37 – 7.25 (m, 5H), 5.62 (brs, 1H), 4.15 (q, *J* = 6.7 Hz, 1H), 3.49 (s, 3H), 1.37 (d, *J* = 6.6 Hz, 3H).

^{13}C {^{1}H} NMR (126 MHz, $CDCl_3$) δ (ppm): 143.0, 128.6, 127.6, 127.2, 62.6, 60.6, 20.0.

7.1.2.7 Synthesis of N-benzyl-O-methylhydroxylamine (5h)

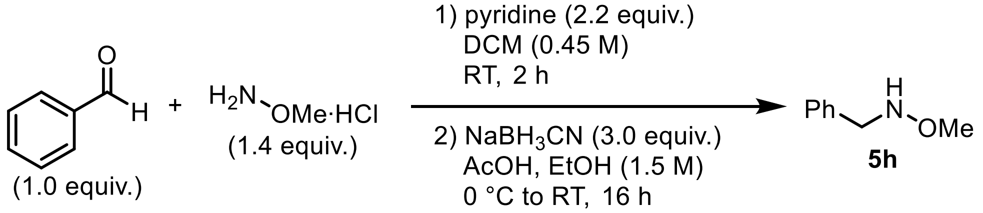

Benzaldehyde (0.826 mL, 8.0 mmol, 1.0 equiv) was dissolved in CH_2Cl_2 (17.8 mL, 0.45 M). *O*-Methylhydroxylamine hydrochloride (0.955 g, 11.2 mmol, 1.4 equiv) and pyridine (1.43 mL, 17.6 mmol, 2.2 equiv) were added, and the mixture was stirred at rt until complete consumption of the aldehyde (TLC, ~2 h). The reaction mixture was washed with 5% aq HCl (3 × 40 mL) and brine (2 × 40 mL). The organic layer was dried over Na_2SO_4 and concentrated under reduced pressure to afford the crude oxime, which was used directly in the next step.

The crude oxime was dissolved in EtOH (5.33 mL, 1.5 M) and cooled to 0 °C. $NaBH_3CN$ (1.51 g, 24.0 mmol, 3.0 equiv) was added, and the mixture was stirred for 15 min at 0 °C. A chilled solution of 20% AcOH in EtOH (5.33 mL, 0 °C) was then added dropwise over 5 min. The reaction was allowed to warm to rt and stirred overnight. The mixture was neutralized with Na_2CO_3 until gas evolution ceased, concentrated under reduced pressure, and CH_2Cl_2 (20 mL) was added. The layers were separated, and the aqueous layer was extracted with CH_2Cl_2 (3 × 20 mL). The combined organic layers were washed with sat. $NaHCO_3$ (20 mL) and brine (20 mL), dried over Na_2SO_4, filtered, and concentrated under reduced pressure. Purification of the crude residue by gradient column chromatography (SiO_2, cyclohexane/EtOAc = 30:1 → 10:1) afforded **5h** (643 mg, 4.69 mmol, 59%) as a clear oil.

The analytical data are consistent with those reported in the literature.[25]

R$_f$ = 0.35 (cyclohexane/EtOAc = 4:1)

^{1}H NMR (400 MHz, $CDCl_3$) δ (ppm): 7.37 – 7.27 (m, 5H), 5.72 (s, 1H), 4.06 (s, 2H), 3.52 (d, *J* = 0.8 Hz, 3H).

^{13}C {^{1}H} NMR (101 MHz, $CDCl_3$) δ (ppm): 137.7, 129.0, 128.6, 127.6, 62.0, 56.4.

HRMS (ESI) ***m/z:*** $[M+H]^+$ calculated for $C_8H_{12}NO$, 138.0913; found, 138.0911.

7.1.2.8 Synthesis of O-methyl-N-(3-phenylpropyl)hydroxylamine (5i)

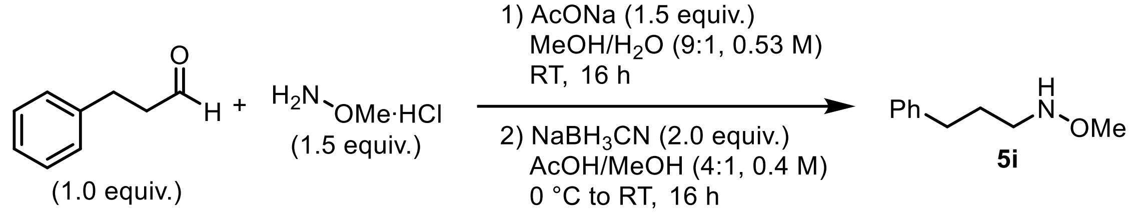

AcONa (0.984 g, 12.0 mmol, 1.5 equiv) and *O*-methylhydroxylamine hydrochloride (1.023 g, 12.0 mmol, 1.5 equiv) were dissolved in MeOH/H_2O (9:1; 15 mL, 0.53 M) in a round-bottom flask. To the resulting suspension, 3-phenylpropionaldehyde (1.06 mL, 8.0 mmol, 1.0 equiv) was added slowly. The reaction mixture was stirred at rt for 16 h, after which the solvents were removed under reduced pressure. The residue was diluted with water (15 mL) and extracted with EtOAc (3 × 40 mL). The combined organic layers were washed with water (2 × 30 mL) and brine (30 mL), dried over Na_2SO_4, and concentrated under reduced pressure to afford the crude oxime, which was used directly in the next step.

The crude oxime was dissolved in AcOH/MeOH (4:1; 20 mL, 0.4 M) and cooled to 0 °C. $NaBH_3CN$ (1.005 g, 16.0 mmol, 2.0 equiv) was added portionwise at 0 °C. The reaction mixture was allowed to warm to rt and stirred for 16 h. A saturated solution of Na_2CO_3 was added, and the aqueous phase was extracted with EtOAc (3 × 20 mL). The combined organic layers were washed with water (30 mL) and brine (30 mL), dried over Na_2SO_4, and concentrated under reduced pressure. Purification of the crude residue by gradient column chromatography (SiO_2, cyclohexane/EtOAc = 15:1 → 5:1) afforded **5i** (1051 mg, 6.36 mmol, 80%) as a clear oil.

$\mathbf{R_f}$ = 0.42 (cyclohexane/EtOAc = 2:1)

^{1}H NMR (400 MHz, $CDCl_3$) δ (ppm): 7.31 – 7.27 (m, 2H), 7.21 – 7.17 (m, 3H), 5.56 (s, 1H), 3.55 (d, *J* = 0.9 Hz, 3H), 2.97 – 2.93 (m, 2H), 2.71 – 2.67 (m, 2H), 1.90 – 1.82 (m, 2H).

^{13}C {^{1}H} NMR (101 MHz, $CDCl_3$) δ (ppm): 142.1, 128.5, 128.5, 126.0, 62.0, 51.5, 33.6, 29.1.

HRMS (ESI) ***m/z:*** $[M+H]^+$ calculated for $C_{10}H_{16}NO$, 166.1226; found, 166.1228.

Synthesis of *tert*-butyl methoxycarbamate (5j)

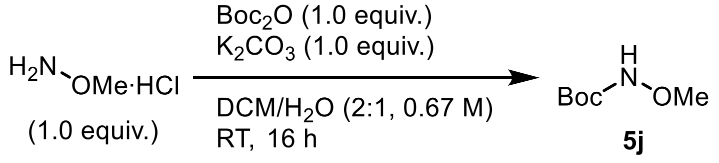

A suspension of *O*-methylhydroxylamine hydrochloride (1.70 g, 20.0 mmol, 1.0 equiv) and K_2CO_3 (2.76 g, 20.0 mmol, 1.0 equiv) in CH_2Cl_2/H_2O (2:1; 30 mL, 0.67 M) was stirred at rt for 1 h. To the resulting suspension, Boc_2O (4.41 g, 20.0 mmol, 1.0 equiv) dissolved in CH_2Cl_2

(4.0 mL, 5.0 M; overall concentration 0.59 M) was added dropwise at 0 °C. The reaction mixture was allowed to warm to rt and stirred for 16 h. Water (50 mL) was added, and the aqueous phase was extracted with CH_2Cl_2 (3 × 50 mL). The combined organic layers were washed with water (50 mL) and brine (50 mL), dried over Na_2SO_4, and concentrated under reduced pressure. The crude product was used without further purification to afford hydroxylamine **5j** (2.79 g, 18.9 mmol, 95%) as a white solid. The analytical data are consistent with those reported in the literature.[26]

R_f = 0.49 (cyclohexane/EtOAc = 3:1)

^{1}H NMR (400 MHz, $CDCl_3$) δ (ppm): 7.27 (s, 1H), 3.69 (s, 3H), 1.47 (s, 9H).

^{13}C {^{1}H} NMR (101 MHz, $CDCl_3$) δ (ppm): 157.0, 81.8, 64.5, 28.3.

7.1.2.9 Synthesis of N-allyl-O-methylhydroxylamine hydrochloride (5k)

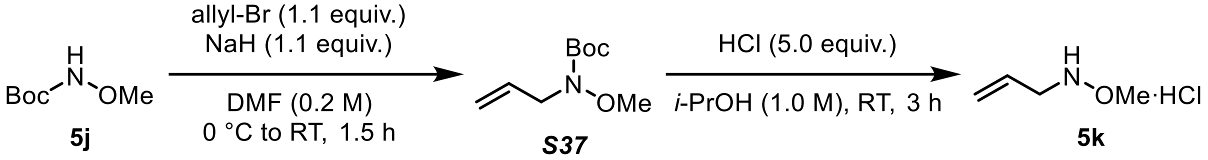

NaH (60% dispersion in mineral oil, 0.308 g, 7.7 mmol, 1.1 equiv) was washed three times with dry hexane under an argon atmosphere. DMF (28.0 mL, 0.25 M) was added, and the suspension was cooled to 0 °C in an ice bath. A solution of protected hydroxylamine **5j** (1.03 g, 7.0 mmol, 1.0 equiv) in DMF (7.0 mL, 1.0 M; overall concentration 0.20 M) was added slowly at 0 °C and stirred for 20 min. Allyl bromide (0.67 mL, 7.7 mmol, 1.1 equiv) was then added. The reaction mixture was allowed to warm to rt and stirred for an additional 1 h. The reaction was quenched with water (30 mL), and the aqueous phase was extracted with EtOAc (3 × 50 mL). The combined organic layers were washed with brine (50 mL), dried over Na_2SO_4, and concentrated under reduced pressure. Product **S37** (0.888 g, 4.74 mmol, 68%) was obtained in sufficient purity and used in the next step without further purification. The analytical data are consistent with those reported in the literature.[27]

R_f = 0.69 (cyclohexane/EtOAc = 2:1)

^{1}H NMR (400 MHz, $CDCl_3$) δ (ppm): 5.88 (ddt, *J* = 17.2, 10.2, 6.0 Hz, 1H), 5.24 (dq, *J* = 17.2, 1.5 Hz, 1H), 5.18 (dq, *J* = 10.1, 1.3 Hz, 1H), 4.03 (dt, *J* = 6.0, 1.4 Hz, 2H), 3.68 (s, 3H), 1.49 (s, 9H).

^{13}C {^{1}H} NMR (101 MHz, $CDCl_3$) δ (ppm): 156.5, 132.9, 118.0, 81.5, 62.7, 52.3, 28.4.

Hydroxylamine **S37** (0.861 g, 4.6 mmol, 1.0 equiv) was dissolved in *i*-PrOH (4.6 mL, 1.0 M), and 35% aq HCl (2.03 mL, 5.0 equiv) was added slowly. The reaction mixture was stirred until complete consumption of the starting material (TLC, 2–3 h). The mixture was concentrated to dryness and co-evaporated twice with 1:1 MeOH/toluene (20 mL each). The resulting residue was dried overnight by lyophilization to afford **5k** (237 mg, 1.92 mmol, 42%) as a brownish solid.

^{1}H NMR (400 MHz, D$_2$O) δ (ppm): 5.87 (ddt, *J* = 17.2, 10.3, 6.9 Hz, 1H), 5.56 – 5.49 (m, 2H), 3.90 (dt, *J* = 6.9, 1.1 Hz, 2H), 3.86 (s, 3H).

^{13}C {^{1}H} NMR (101 MHz, D$_2$O) δ (ppm): 125.2, 124.8, 61.5, 51.2.

HRMS (ESI) *m/z:* [M-Cl]$^+$ calculated for $C_4H_{10}NO$, 88.0757; found, 88.0760.

7.1.2.10 Synthesis of N-(2-(benzyloxy)ethyl)-O-methylhydroxylamine hydrochloride (5l)

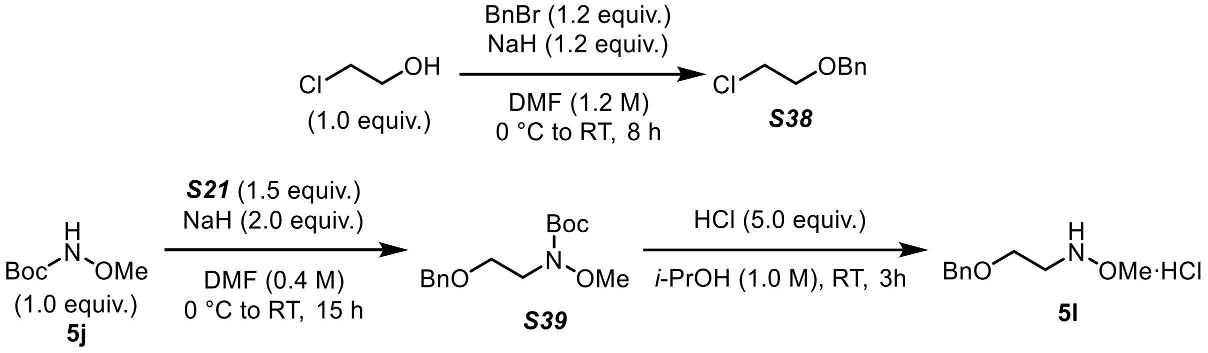

To an ice-cooled suspension of NaH (60% dispersion in mineral oil, 0.720 g, 18.0 mmol, 1.2 equiv) in DMF (12.5 mL, 1.2 M) under an argon atmosphere was added benzyl bromide (2.18 mL, 18.0 mmol, 1.2 equiv). 2-Chloroethanol (1.01 mL, 15.0 mmol, 1.0 equiv) was then added slowly at 0 °C. The reaction mixture was allowed to warm to rt and stirred for 8 h. The reaction was quenched with water (40 mL), and the aqueous phase was extracted with EtOAc (3 × 50 mL). The combined organic layers were washed with brine (50 mL), dried over Na_2SO_4, and concentrated under reduced pressure to afford crude alkyl chloride **S38**, which was used directly in the next step.

NaH (60% dispersion in mineral oil, 0.160 g, 4.0 mmol, 2.0 equiv) was added under an argon atmosphere to DMF (5.0 mL, 0.4 M) at 0 °C. Hydroxylamine **5j** (0.294 g, 2.0 mmol, 1.0 equiv) was added slowly, and after addition was complete, the reaction mixture was allowed to warm to rt and stirred for 1 h. The mixture was cooled to 0 °C, and alkyl chloride **S38** (0.512 mL, 3.0 mmol, 1.5 equiv) was added. The reaction mixture was allowed to warm to rt and stirred for 14 h. The reaction was quenched with water (30 mL), and the aqueous phase was extracted with Et_2O (3 × 20 mL). The combined organic layers were washed with brine (50 mL), dried over Na_2SO_4, and concentrated under reduced pressure. Purification of the crude product by gradient column chromatography (SiO_2, hexane/EtOAc = 15:1 → 5:1) afforded **S39** (170 mg, 0.60 mmol, 30%) as a clear oil.

R$_f$ = 0.61 (cyclohexane/EtOAc = 2:1)

^{1}H NMR (400 MHz, CDCl$_3$) δ (ppm): 7.34 – 7.27 (m, 5H), 4.55 (s, 2H), 3.69 (s, 3H), 3.69 – 3.63 (m, 4H), 1.48 (s, 9H).

^{13}C {^{1}H} NMR (101 MHz, CDCl$_3$) δ (ppm): 156.6, 138.3, 128.5, 127.8, 127.8, 81.5, 73.1, 66.7, 62.5, 49.2, 28.4.

Anal. Calcd for $C_{15}H_{23}NO_4$: C, 64.04; H, 8.24; N, 4.98. Found: C, 64.16; H, 8.11; N, 5.16.

Hydroxylamine **S39** (155 mg, 0.55 mmol, 1.0 equiv) was dissolved in *i*-PrOH (0.55 mL, 1.0 M), and 35% aq HCl (0.243 mL, 5.0 equiv) was added slowly. The reaction mixture was stirred until complete consumption of the starting material (TLC, 2–3 h). The mixture was concentrated to dryness and co-evaporated twice with 1:1 MeOH/toluene (20 mL each). The resulting residue was dried overnight by lyophilization to afford **5l** (120 mg, 0.55 mmol, quant.) as a white solid.

^{1}H NMR (400 MHz, D_2O) δ (ppm): 7.45 – 7.36 (m, 5H), 4.59 (s, 2H), 3.85 (s, 3H), 3.81 – 3.79 (m, 2H), 3.51 – 3.49 (m, 2H).

^{13}C {^{1}H} NMR (101 MHz, D_2O) δ (ppm): 136.9, 128.8, 128.5, 128.5, 72.9, 62.2, 61.5, 48.6.

HRMS (ESI) *m/z:* $[M\text{-}Cl+H]^+$ calculated for $C_{10}H_{16}NO_2$, 182.1176; found, 182.1175.

7.1.2.11 Synthesis of N-(3-(benzyloxy)-2-((tert-butyldimethylsilyl)oxy)propyl)-O-methylhydroxylamine (5m)

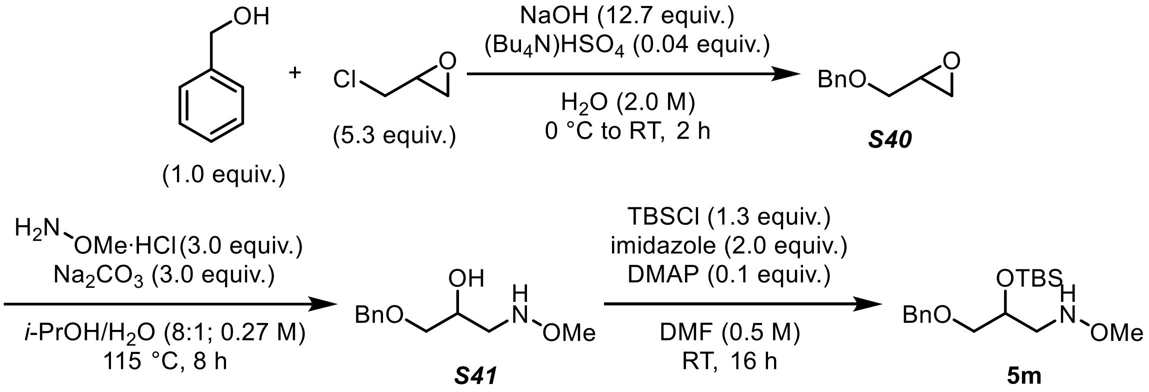

To an ice-cooled solution of NaOH (10.1 g, 12.7 mmol, 12.7 equiv) in water (10.0 mL, 2.0 M) were added 2-(chloromethyl)oxirane (8.4 mL, 107 mmol, 5.3 equiv) and tetrabutylammonium hydrogen sulfate (0.272 g, 0.80 mmol, 0.04 equiv). Phenylmethanol (2.1 mL, 20.0 mmol, 1.0 equiv) was added slowly over 30 min. The reaction mixture was allowed to warm to rt and stirred for 1.5 h until complete consumption of the starting material (TLC). The reaction was poured into ice water (40 mL), and the aqueous phase was extracted with Et_2O (3 × 50 mL). The combined organic layers were washed with brine (50 mL), dried over $MgSO_4$, and concentrated under reduced pressure. Purification of the crude residue by gradient column chromatography (SiO_2, cyclohexane/EtOAc = 15:1 → 5:1) afforded epoxide **S40** (3.13 g, 19.1 mmol, 95%) as a clear oil. Spectroscopic data were consistent with values reported in the literature.[28]

R_f = 0.51 (cyclohexane/EtOAc = 3:1)

^{1}H NMR (400 MHz, $CDCl_3$) δ (ppm): 7.36 – 7.26 (m, 5H), 4.59 (dd, *J* = 23.2, 11.9 Hz, 2H), 3.77 (dd, *J* = 11.5, 3.1 Hz, 1H), 3.45 (dd, *J* = 11.4, 5.8 Hz, 1H), 3.21 – 3.17 (m, 1H), 2.82 – 2.79 (m, 1H), 2.62 (dd, *J* = 5.1, 2.7 Hz, 1H).

^{13}C {^{1}H} NMR (101 MHz, $CDCl_3$) δ (ppm): 138.0, 128.5, 127.9, 73.4, 70.9, 51.0, 44.4.

Epoxide **S40** (0.821 g, 5.0 mmol, 1.0 equiv), *O*-methylhydroxylamine hydrochloride (1.28 g, 15.0 mmol, 3.0 equiv), and Na_2CO_3 (1.61 g, 15.0 mmol, 3.0 equiv) were dissolved in *i*-PrOH/H_2O (8:1; 18.8 mL, 0.27 M) and heated (heating block) in a pressure vial at 115 °C until complete consumption of the starting epoxide (8 h, TLC). The volatiles were removed under reduced pressure, and water (20 mL) was added to the resulting slurry. The aqueous phase was extracted with EtOAc (3 × 30 mL). The combined organic layers were washed with brine (50 mL), dried over $MgSO_4$, and concentrated under reduced pressure to afford **S41** (0.96 g, 4.56 mmol, 91%) as a colorless oil.

R_f = 0.14 (cyclohexane/EtOAc = 3:1)

^{1}H NMR (400 MHz, $CDCl_3$) δ (ppm): 7.37 – 7.27 (m, 5H), 4.56 (s, 2H), 4.05 (ddt, *J* = 7.8, 6.3, 3.7 Hz, 1H), 3.55 (dd, *J* = 9.7, 4.0 Hz, 1H), 3.53 (s, 3H), 3.46 (dd, *J* = 9.9, 6.3 Hz, 1H), 3.08 (dd, *J* = 13.5, 3.4 Hz, 1H), 2.90 (dd, *J* = 13.5, 8.2 Hz, 1H).

^{13}C {^{1}H} NMR (101 MHz, $CDCl_3$) δ (ppm): 138.1, 128.6, 127.9, 127.8, 73.6, 72.7, 68.1, 61.9, 54.3.

HRMS (ESI) *m/z:* $[M+H]^+$ calculated for $C_{11}H_{18}NO_3$, 212.1281; found, 212.1282.

To a solution of unprotected alcohol **S41** (0.634 g, 3.0 mmol, 1.0 equiv), DMAP (0.037 g, 0.30 mmol, 0.10 equiv), and imidazole (0.408 g, 6.0 mmol, 2.0 equiv) in DMF (6.0 mL, 0.50 M) was added TBSCl (0.600 g, 3.9 mmol, 1.3 equiv) slowly, and the reaction mixture was stirred at rt for 16 h. The reaction was quenched with water (20 mL), diluted with EtOAc (20 mL), and the aqueous phase was extracted with EtOAc (3 × 15 mL). The combined organic layers were washed with brine (3 × 30 mL), dried over Na_2SO_4, and concentrated under reduced pressure. Purification of the crude residue by gradient column chromatography (SiO_2, hexane/Et_2O = 15:1 → 5:1) afforded **5m** (0.47 g, 1.45 mmol, 48%) as a clear oil.

R_f = 0.54 (hexane/Et_2O = 4:1)

^{1}H NMR (400 MHz, $CDCl_3$) δ (ppm): 7.37 – 7.26 (m, 5H), 5.87 (s, 1H), 4.55 (d, *J* = 12.2 Hz, 1H), 4.51 (d, *J* = 12.2 Hz, 1H), 4.12 – 4.07 (m, 1H), 3.50 (s, 3H), 3.47 (dd, *J* = 9.8, 5.5 Hz, 1H), 3.43 (dd, *J* = 9.8, 5.5 Hz, 1H), 3.12 (dd, *J* = 13.2, 4.0 Hz, 1H), 2.87 (dd, *J* = 13.2, 7.2 Hz, 1H), 0.90 (d, *J* = 0.7 Hz, 9H), 0.10 (s, 3H), 0.07 (s, 3H).

^{13}C {^{1}H} NMR (101 MHz, $CDCl_3$) δ (ppm): 138.4, 128.5, 127.7, 127.7, 73.6, 73.5, 68.4, 61.3, 55.2, 26.0, 18.3, -4.4, -4.9.

HRMS (ESI) *m/z:* $[M+H]^+$ calculated for $C_{17}H_{32}NO_3Si$, 326.2146; found, 326.2146.

7.1.2.12 Synthesis of N-methyl-O-(methylsulfonyl)hydroxylamine (5n)

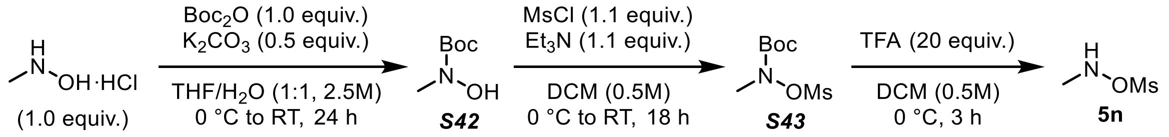

Potassium carbonate (0.81 g, 5.87 mmol, 0.50 equiv) was added to an ice-cold solution of *N*-methylhydroxylamine hydrochloride (1.00 g, 11.7 mmol, 1.0 equiv) in THF/H_2O (1:1; 4.7 mL, 2.5 M). A solution of Boc_2O (4.8 g, 22.0 mmol, 2.0 equiv) in THF (3.5 mL, 3.3 M) was then added dropwise, and the mixture was stirred for 4 h at 0 °C and then overnight at rt. The mixture was concentrated *in vacuo*, and the residue was dissolved in CH_2Cl_2 (25 mL) and washed with water (3 × 10 mL) and brine (25 mL). The organic phase was dried over Na_2SO_4 and concentrated under reduced pressure to afford **S42** (1.07 g, 66%) as an orange oil, which was used directly in the next step.

To a solution of crude **S42** (0.734 g, 5.0 mmol, 1.0 equiv) in CH_2Cl_2 (10 mL, 0.50 M) were added Et_3N (0.76 mL, 5.5 mmol, 1.1 equiv) and methanesulfonyl chloride (0.43 mL, 5.5 mmol, 1.1 equiv) at 0 °C. The reaction mixture was allowed to warm to rt and stirred for 18 h. The mixture was washed with 1 M HCl (10 mL) and brine (20 mL), dried over Na_2SO_4, filtered, and concentrated under reduced pressure. Purification of the crude residue by gradient column chromatography (SiO_2, cyclohexane/EtOAc = 20:1 → 7:1) afforded **S43** (1.05 g, 4.65 mmol, 93%) as a clear oil. The analytical data are consistent with those reported in the literature.[29]

$\mathbf{R_f}$ = 0.42 (cyclohexane/EtOAc = 3:1)

^{1}H NMR (400 MHz, $CDCl_3$) δ (ppm): 3.32 (s, 3H), 3.14 (s, 3H), 1.52 (s, 9H).

^{13}C {^{1}H} NMR (101 MHz, $CDCl_3$) δ (ppm): 156.2, 84.5, 40.7, 36.8, 28.2.

To a solution of carbamate **S43** (0.68 g, 3.0 mmol, 1.0 equiv.) in CH_2Cl_2 (6.0 mL, 0.5M) was added trifluoroacetic acid (4.5 mL, 60 mmol, 20 equiv.) at 0 °C. The reaction was mixed for 3 h at 0 °C and then it was poured into 15 mL ice water and extracted with CH_2Cl_2 (3 x 20 mL). The combined organic layers were dried over Na_2SO_4, filtered and concentrated under reduced pressure. The crude product was purified by gradient column chromatography (SiO_2, cyclohexane/EtOAc = 10:1 to 1:1) to give **5n** (0.33 g, 2.60 mmol, 87 % yield) as a clear oil. Spectroscopic data were consistent with the values reported in the literature.[29]

$\mathbf{R_f}$ = 0.43 (cyclohexane/EtOAc = 1:1)

^{1}H NMR (400 MHz, $CDCl_3$) δ (ppm): 6.75 (s, 1H), 3.09 (s, 3H), 2.98 (s, 3H).

^{13}C {^{1}H} NMR (101 MHz, $CDCl_3$) δ (ppm): 40.6, 35.1

7.1.2.13 Synthesis of O-(tert-butyl)-N-methylhydroxylamine hydrochloride (5o)

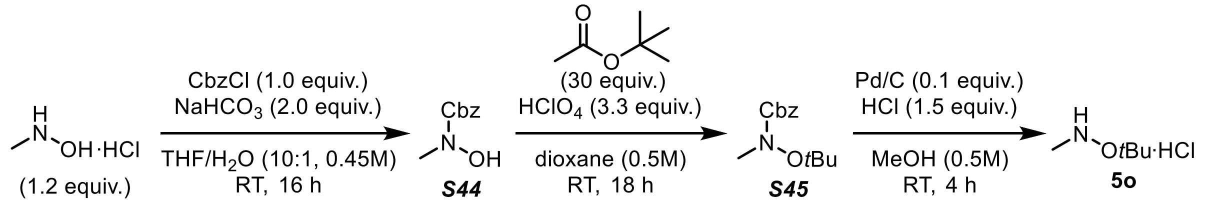

To a stirred suspension of *N*-methyl hydroxylamine hydrochloride (2.56 g, 15 mmol, 1.2 equiv.) in a 10:1 mixture of tetrahydrofuran and water (33 mL, 0.45M) was added $NaHCO_3$ (2.52 g, 30 mmol, 2.0 equiv.) and benzyl chloroformate (2.1 mL, 15 mmol, 1.0 equiv.). The resulting

suspension was stirred overnight at room temperature. After this period, the solution was diluted with water (20 mL) and extracted with dichloromethane (3x30 mL). The combined organic extracts were washed with brine (25 ml) and dried over $MgSO_4$, filtered and reduced in vacuo to yield ***S44*** in quant. yield as an oil, which was used directly in the next step.

To a solution of crude *N*-Cbz-*N*-methyl hydroxylamine ***S44*** (0.453 g, 2.5 mmol, 1.0 equiv.) in dioxane (4 mL, 0.5M) was added AcO*t*Bu (10 mL, 75 mmol, 30 equiv.) and 70% $HClO_4$ (0.71 mL, 8.25 mmol, 3.3 equiv.) at 0 °C. The reaction mixture was allowed to warm to room temperature and stirring for 18 h. The reaction mixture was then poured onto a saturated $NaHCO_3$ (30 mL) and stirred for 30 min. Then, the aqueous phase was extracted with dichloromethane (3x20 mL). The combined organic extracts were washed with brine (20 mL) and organic phase was dried over $MgSO_4$, filtered and concentrated under reduced pressure. The crude product was purified by gradient column chromatography (SiO_2, cyclohexane/EtOAc = 20:1 → 10:1) to give **S45** (0.43 g, 1.81 mmol, 73 % yield) as a clear oil. Spectroscopic data were consistent with the values reported in the literature.[16]

R_f = 0.42 (cyclohexane/EtOAc = 5:1)

^{1}H NMR (400 MHz, $CDCl_3$) δ (ppm): 7.39 – 7.29 (m, 5H), 5.16 (s, 2H), 3.19 (s, 3H), 1.24 (s, 9H).

^{13}C {^{1}H} NMR (101 MHz, $CDCl_3$) δ (ppm): 160.1, 136.3, 128.6, 128.3, 128.3, 82.1, 68.0, 41.3, 27.3.

Hydroxylamine ***S45*** (356 mg, 1.55 mmol, 1.0 equiv.) was dissolved in MeOH (3 mL, 0.5 M), treated with 35 % aq. HCl (0.199 mL, 1.5 equiv.) and put under argon atmosphere. To the solution was then added 10 % Pd/C (16 mg, 0.15 mmol, 0.1 equiv.) and whole flask was evacuated and backfilled with nitrogen three times, and then filled with hydrogen and evacuated three times and finally a balloon of hydrogen was attached to the flask. The reaction was stirred until complete consumption of starting material (monitored by TLC, 4-5 h). The reaction was then filtered through a Celite, washed with methanol and concentrated to dryness, then co-evaporated twice from 10 mL dry MeOH. The resulting residue was dried overnight by lyophilization to afford **5o** (210 mg, 1.5 mmol, quant. yield) as white solid. Spectroscopic data were consistent with the values reported in the literature.[16]

^{1}H NMR (400 MHz, CD_3OD) δ (ppm): 2.97 (s, 3H), 1.42 (s, 9H).

^{13}C {^{1}H} NMR (101 MHz, CD_3OD) δ (ppm): 85.0, 37.4, 26.6.

7.2 Substrate Scope of Synthesis of 1,3-diazetidin-2-one (aza-β-lactam)

General Procedure (Starting from Ammonium Salts – METHOD A)

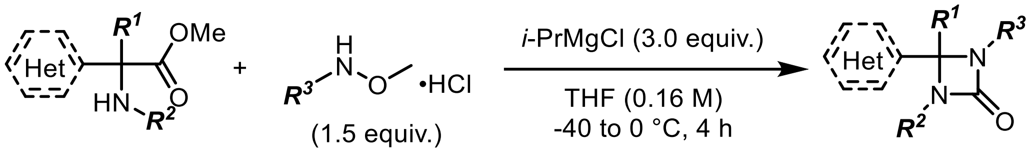

Amino ester **4** (0.20 mmol, 1.0 equiv) was dissolved in dry THF (1.25 mL, 0.16 M) containing a suspension of hydroxylamine hydrochloride **5** (0.30 mmol, 1.5 equiv) in a flame-dried round-bottom flask under an argon atmosphere. The reaction mixture was cooled to –40 °C, and *i*PrMgCl (2.0 M in THF, 0.60 mmol, 3.0 equiv) was added dropwise. The mixture was stirred while slowly warming from –40 °C to 0 °C over 4 h. After this time (or upon complete consumption of the starting material), the reaction was quenched with sat. aq NH_4Cl and extracted with EtOAc (3 × 20 mL). The combined organic layers were washed with brine, dried over Na_2SO_4, and concentrated under reduced pressure. Purification of the crude residue by gradient column chromatography afforded the desired products **3**.

General Procedure (Starting from Free Hydroxylamines – METHOD B)

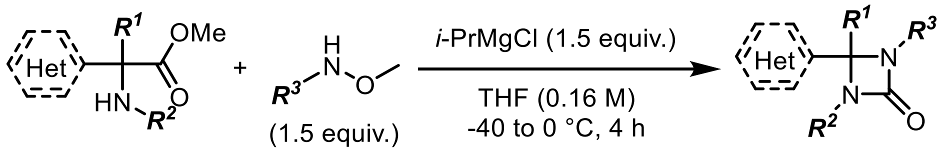

Amino ester **4** (1.0 equiv) was dissolved in dry THF (0.16 M) containing a suspension of hydroxylamine **5** (1.5 equiv) in a flame-dried round-bottom flask under an argon atmosphere. The reaction mixture was cooled to –40 °C, and *i*PrMgCl (2.0 M in THF, 1.5 equiv) was added dropwise. The mixture was stirred while gradually warming from –40 °C to 0 °C over 4 h. Upon completion (TLC) or after 4 h, the reaction was quenched with sat. aq NH_4Cl and extracted with EtOAc (3 × 20 mL). The combined organic layers were washed with brine, dried over Na_2SO_4, and concentrated under reduced pressure. Purification of the crude residue by gradient column chromatography afforded the desired products **3**.

7.2.1 Characterization data

7.2.1.1 4-(benzo[d]thiazol-2-yl)-1-benzyl-3,4-dimethyl-1,3-diazetidin-2-one (3a)

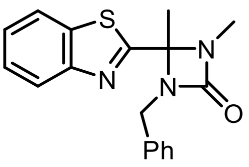

3a

Synthesized following **Method A**, starting from amino ester **4a** (65.3 mg, 0.20 mmol, 1.0 equiv) and hydroxylamine hydrochloride **5a** (29.3 mg, 0.30 mmol, 1.5 equiv). Purification by column chromatography (SiO_2, hexane/EtOAc = 10:1 → 3:1) afforded 1,3-diazetidin-2-one **3a** (49 mg, 0.15 mmol, 76%) as a colorless oil.

$\mathbf{R_f}$ = 0.32 (hexane/EtOAc = 3:1)

^{1}H NMR (400 MHz, $CDCl_3$) δ (ppm): 8.02 – 8.00 (m, 1H), 7.88 – 7.86 (m, 1H), 7.49 (ddt, *J* = 8.1, 7.3, 1.0 Hz, 1H), 7.41 (ddt, *J* = 8.0, 7.2, 1.0 Hz, 1H), 7.25 – 7.16 (m, 5H), 4.36 (d, *J* = 15.4 Hz, 1H), 4.15 (d, *J* = 15.5 Hz, 1H), 2.77 (s, 3H), 1.93 (s, 3H).

^{13}C {^{1}H} NMR (101 MHz, $CDCl_3$) δ (ppm): 170.4, 158.9, 153.8, 137.0, 135.8, 128.5, 128.5, 127.5, 126.4, 126.0, 123.6, 122.1, 78.7, 45.6, 26.6, 19.6.

HRMS (ESI) *m/z:* $[M+H-C_8H_7NO]^+$ calculated for $C_{10}H_{11}N_2S$, 191.0637; found, 191.0637.

CHIRAL HPLC (SA, Hexane : iPrOH = 95:5, 0.5 mL/min, 298 K, 220 nm): t_{R1} = 21.52 min, t_{R2} = 22.68 min.

7.2.1.2 4-(benzo[d]thiazol-2-yl)-1,4-dibenzyl-3-methyl-1,3-diazetidin-2-one (3b)

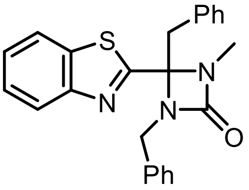

3b

Synthesized following **METHOD A**, starting from amino ester **4b** (80.5 mg, 0.20 mmol, 1.0 equiv.) and hydroxylamine hydrochloride **5a** (29.3 mg, 0.30 mmol, 1.5 equiv.). Purification by column chromatography (SiO_2, hexane/EtOAc = 15:1 to 3:1) afforded 1,3-diazetidin-2-one **3b** (52 mg, 0.13 mmol, 65 % yield) as a colorless oil.

$\mathbf{R_f}$ = 0.34 (hexane/EtOAc = 3:1)

^{1}H NMR (400 MHz, $CDCl_3$) δ (ppm): 8.10 (ddd, *J* = 8.2, 1.1, 0.6 Hz, 1H), 7.89 (ddd, *J* = 8.0, 1.2, 0.6 Hz, 1H), 7.55 (ddd, *J* = 8.3, 7.3, 1.3 Hz, 1H), 7.45 (ddd, *J* = 8.3, 7.3, 1.2 Hz, 1H), 7.22 – 7.13 (m, 10H), 4.31 (d, *J* = 15.4 Hz, 1H), 4.03 (d, *J* = 15.4 Hz, 1H), 3.80 (d, *J* = 15.1 Hz, 1H), 3.71 (d, *J* = 15.1 Hz, 1H), 2.74 (s, 3H).

$^{13}C\{^{1}H\}$ **NMR (101 MHz, CDCl₃) δ (ppm):** 169.9, 158.2, 153.7, 137.0, 135.7, 134.0, 130.8, 128.7, 128.5, 128.4, 127.5, 127.3, 126.5, 126.1, 123.8, 122.2, 81.2, 45.9, 39.1, 27.3.

HRMS (ESI) *m/z:* $[M+H]^+$ calculated for $C_{24}H_{22}N_3OS$, 400.1478; found, 400.1477.

7.2.1.3 4-(benzo[d]thiazol-2-yl)-1-benzyl-4-isopropyl-3-methyl-1,3-diazetidin-2-one (3c)

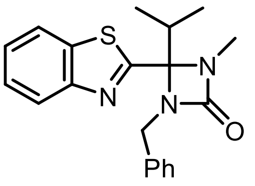

3c

Synthesized following **METHOD A**, starting from amino ester **4c** (70.9 mg, 0.20 mmol, 1.0 equiv.) and hydroxylamine hydrochloride **5a** (29.3 mg, 0.30 mmol, 1.5 equiv.). Purification by column chromatography (SiO_2, hexane/EtOAc = 15:1 → 5:1) afforded 1,3-diazetidin-2-one **3c** (40 mg, 0.12 mmol, 58 % yield) as a colorless oil.

$\mathbf{R_f}$ = 0.49 (hexane/EtOAc = 3:1)

^{1}**H NMR (500 MHz, CDCl₃) δ (ppm):** 8.06 – 8.04 (m, 1H), 7.90 – 7.89 (m, 1H), 7.52 (ddd, *J* = 8.3, 7.2, 1.3 Hz, 1H), 7.44 (ddd, *J* = 8.2, 7.2, 1.2 Hz, 1H), 7.25 – 7.17 (m, 5H), 4.36 (d, *J* = 15.4 Hz, 1H), 3.93 (d, *J* = 15.5 Hz, 1H), 2.97 (s, 3H), 2.82 (hept, *J* = 7.0 Hz, 1H), 1.09 (d, *J* = 6.9 Hz, 3H), 1.08 (d, *J* = 7.0 Hz, 3H).

$^{13}C\{^{1}H\}$ **NMR (126 MHz, CDCl₃) δ (ppm):** 169.7, 158.5, 154.0, 137.4, 135.1, 128.6, 128.4, 127.4, 126.4, 125.9, 123.7, 122.0, 84.7, 45.8, 31.9, 28.3, 17.6, 16.7.

HRMS (ESI) *m/z:* $[M+H]^+$ calculated for $C_{20}H_{22}N_3OS$, 352.1478; found, 352.1474.

7.2.1.4 4-(benzo[d]thiazol-2-yl)-1-benzyl-4-isobutyl-3-methyl-1,3-diazetidin-2-one (3d)

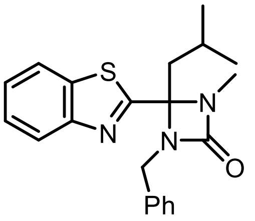

3d

Synthesized following **METHOD A**, starting from amino ester **4d** (73.7 mg, 0.20 mmol, 1.0 equiv.) and hydroxylamine hydrochloride **5a** (29.3 mg, 0.30 mmol, 1.5 equiv.). Purification by column chromatography (SiO_2, hexane/EtOAc = 15:1 → 5:1) afforded 1,3-diazetidin-2-one **3d** (38 mg, 0.11 mmol, 53 % yield) as a colorless oil.

$\mathbf{R_f}$ = 0.53 (hexane/EtOAc = 3:1)

^{1}H NMR (500 MHz, CDCl$_3$) δ (ppm): 8.04 – 8.02 (m, 1H), 7.85 – 7.83 (m, 1H), 7.50 (ddd, *J* = 8.3, 7.2, 1.3 Hz, 1H), 7.41 (ddd, *J* = 8.3, 7.2, 1.2 Hz, 1H), 7.24 – 7.15 (m, 5H), 4.22 (s, 2H), 2.77 (s, 3H), 2.32 – 2.31 (m, 2H), 1.89 – 1.81 (m, 1H), 1.01 (d, *J* = 6.7 Hz, 3H), 0.91 (d, *J* = 6.7 Hz, 3H)

^{13}C {^{1}H} NMR (126 MHz, CDCl$_3$) δ (ppm): 170.5, 158.8, 153.7, 137.0, 135.5, 128.8, 128.4, 127.5, 126.4, 125.9, 123.6, 122.1, 81.6, 45.9, 40.1, 27.1, 24.3, 24.2, 23.4.

HRMS (ESI) *m/z:* [M+H]$^+$ calculated for $C_{21}H_{24}N_3OS$, 366.1635; found, 366.1635.

7.2.1.5 4-(benzo[d]thiazol-2-yl)-1-benzyl-4-(4-(benzyloxy)benzyl)-3-methyl-1,3-diazetidin-2-one (3e)

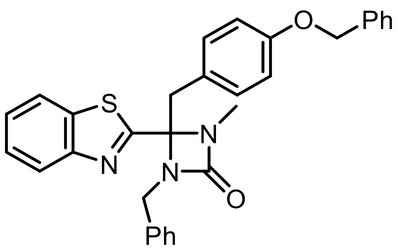

3e

Synthesized following **METHOD A**, starting from amino ester **4e** (102 mg, 0.20 mmol, 1.0 equiv.) and hydroxylamine hydrochloride **5a** (29.3 mg, 0.30 mmol, 1.5 equiv.). Purification by column chromatography (SiO$_2$, hexane/EtOAc = 15:1 → 5:1) afforded 1,3-diazetidin-2-one **3e** (51 mg, 0.10 mmol, 50 % yield) as a colorless oil.

R$_f$ = 0.38 (hexane/EtOAc = 2:1)

^{1}H NMR (500 MHz, CDCl$_3$) δ (ppm): 8.10 (ddd, *J* = 8.4, 1.1, 0.5 Hz, 1H), 7.90 – 7.88 (m, 1H), 7.55 (ddd, *J* = 8.3, 7.2, 1.3 Hz, 1H), 7.46 (ddd, *J* = 8.4, 7.4, 1.2 Hz, 1H), 7.44 – 7.37 (m, 4H), 7.35 – 7.31 (m, 1H), 7.23 – 7.17 (m, 5H), 7.07 – 7.05 (m, 2H), 6.84 – 6.81 (m, 2H), 5.02 (s, 2H), 4.31 (d, *J* = 15.4 Hz, 1H), 4.03 (d, *J* = 15.4 Hz, 1H), 3.73 (d, *J* = 15.1 Hz, 1H), 3.66 (d, *J* = 15.2 Hz, 1H), 2.75 (s, 3H).

^{13}C {^{1}H} NMR (126 MHz, CDCl$_3$) δ (ppm): 169.9, 158.3, 158.1, 153.7, 137.1, 137.1, 135.7, 131.8, 128.72 and 128.71 (partial overlap), 128.4, 128.1, 127.6, 127.4, 126.5, 126.2, 126.1, 123.8, 122.1, 114.7, 81.2, 70.1, 45.9, 38.2, 27.3.

HRMS (ESI) *m/z:* [M+H]$^+$ calculated for $C_{31}H_{28}N_3O_2S$, 506.1897; found, 506.1901.

7.2.1.6 2-(benzo[d]thiazol-2-yl)-N-methoxy-N-methylpyrrolidine-2-carboxamide (1g)

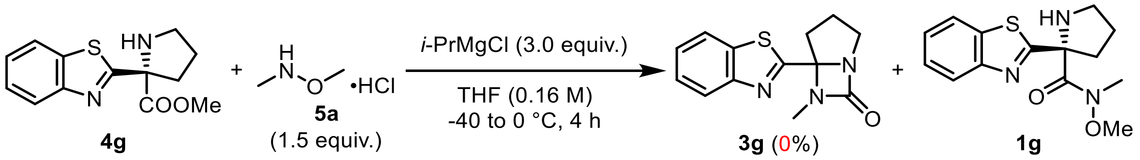

Synthesized following **METHOD A**, starting from amino ester **4g** (35 mg, 0.13 mmol, 1.0 equiv.) and hydroxylamine hydrochloride **5a** (19.5 mg, 0.20 mmol, 1.5 equiv.). Purification by column chromatography (SiO_2, hexane/EtOAc = 10:1 → 3:1) afforded no 1,3-diazetidin-2-one **3g**, but amide **1g** (6 mg, 0.02 mmol, 15 % yield) as a colorless oil.

R_f = 0.44 (hexane/EtOAc = 2:1)

^1H NMR (400 MHz, $CDCl_3$) δ (ppm): 7.97 (ddd, *J* = 8.2, 1.3, 0.7 Hz, 1H), 7.86 (ddd, *J* = 7.9, 1.3, 0.6 Hz, 1H), 7.43 (ddd, *J* = 8.3, 7.2, 1.3 Hz, 1H), 7.34 (ddd, *J* = 8.3, 7.2, 1.2 Hz, 1H), 3.24 (s, 3H), 3.19 (ddd, *J* = 9.4, 6.8, 5.5 Hz, 1H), 3.09 (dt, *J* = 9.4, 7.3 Hz, 1H), 3.02 (s, 3H), 2.88 (dt, *J* = 13.2, 7.0 Hz, 1H), 2.24 (dt, *J* = 13.1, 7.4 Hz, 1H), 1.95 (dddd, *J* = 12.4, 7.4, 6.2, 2.5 Hz, 2H).

^{13}C {^1H} NMR (101 MHz, $CDCl_3$) δ (ppm): 178.9, 172.1, 153.8, 135.2, 125.9, 124.9, 123.1, 121.8, 72.0, 60.0, 46.6, 36.8, 33.5, 25.6.

HRMS (ESI) *m/z:* $[M+H]^+$ calculated for $C_{14}H_{18}N_3O_2S$, 292.1114; found, 292.1115.

7.2.1.7 4-(benzo[d]thiazol-2-yl)-1,3,4-trimethyl-1,3-diazetidin-2-one (3h)

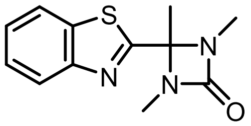

3h

Synthesized following **METHOD A**, starting from amino ester **4h** (50.1 mg, 0.20 mmol, 1.0 equiv.) and hydroxylamine hydrochloride **5a** (29.3 mg, 0.30 mmol, 1.5 equiv.). Purification by column chromatography (SiO_2, hexane/EtOAc = 15:1 → 3:1) afforded 1,3-diazetidin-2-one **3h** (23 mg, 0.09 mmol, 46 % yield) as a colorless oil.

R_f = 0.31 (hexane/EtOAc = 2:1)

^1H NMR (500 MHz, $CDCl_3$) δ (ppm): 8.05 – 8.03 (m, 1H), 7.92 – 7.90 (m, 1H), 7.51 (ddd, *J* = 8.3, 7.2, 1.2 Hz, 1H), 7.43 (ddd, *J* = 8.3, 7.3, 1.2 Hz, 1H), 2.73 (s, 6H), 2.00 (s, 3H).

^{13}C {^1H} NMR (126 MHz, $CDCl_3$) δ (ppm): 170.4, 159.4, 154.0, 135.7, 126.4, 126.0, 123.6, 122.2, 78.0, 26.8, 18.8.

HRMS (ESI) *m/z:* $[M+H-C_2H_3NO]^+$ calculated for $C_{10}H_{11}N_2S$, 191.0637; found, 191.0634.

7.2.1.8 1-allyl-4-(benzo[d]thiazol-2-yl)-3,4-dimethyl-1,3-diazetidin-2-one (3i)

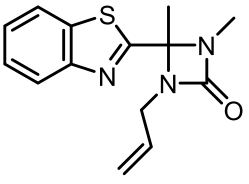

3i

Synthesized following **METHOD A**, starting from amino ester **4i** (55.3 mg, 0.20 mmol, 1.0 equiv.) and hydroxylamine hydrochloride **5a** (29.3 mg, 0.30 mmol, 1.5 equiv.). Purification by column chromatography (SiO_2, hexane/EtOAc = 15:1 → 3:1) afforded 1,3-diazetidin-2-one **3i** (34 mg, 0.13 mmol, 63 % yield) as a colorless oil.

R_f = 0.49 (hexane/EtOAc = 2:1)

^{1}H NMR (500 MHz, $CDCl_3$) δ (ppm): 8.04 – 8.02 (m, 1H), 7.92 – 7.90 (m, 1H), 7.51 (ddd, *J* = 8.3, 7.2, 1.3 Hz, 1H), 7.43 (ddd, *J* = 8.3, 7.2, 1.2 Hz, 1H), 5.79 (ddt, *J* = 16.7, 10.2, 6.2 Hz, 1H), 5.14 (dq, *J* = 17.1, 1.5 Hz, 1H), 5.05 (dq, *J* = 10.1, 1.3 Hz, 1H), 3.79 – 3.70 (m, 2H), 2.72 (s, 3H), 2.03 (s, 3H).

^{13}C {^{1}H} NMR (126 MHz, $CDCl_3$) δ (ppm): 170.6, 158.8, 153.8, 135.7, 133.4, 126.4, 126.0, 123.6, 122.2, 118.1, 78.3, 44.5, 26.6, 19.4.

HRMS (ESI) *m/z:* $[M+H-C_4H_5NO]^+$ calculated for $C_{10}H_{11}N_2S$, 191.0637; found, 191.0635.

7.2.1.9 4-(benzo[d]thiazol-2-yl)-1-butyl-3,4-dimethyl-1,3-diazetidin-2-one (3j)

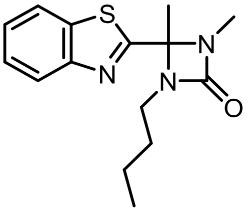

3j

Synthesized following **METHOD A**, starting from amino ester **4j** (58.5 mg, 0.20 mmol, 1.0 equiv.) and hydroxylamine hydrochloride **5a** (29.3 mg, 0.30 mmol, 1.5 equiv.). Purification by column chromatography (SiO_2, hexane/EtOAc = 15:1 → 3:1) afforded 1,3-diazetidin-2-one **3j** (32 mg, 0.11 mmol, 55 % yield) as a colorless oil.

R_f = 0.60 (hexane/EtOAc = 2:1)

^{1}H NMR (400 MHz, $CDCl_3$) δ (ppm): 8.03 (ddd, *J* = 8.2, 1.2, 0.6 Hz, 1H), 7.91 (ddd, *J* = 7.9, 1.3, 0.6 Hz, 1H), 7.50 (ddt, *J* = 8.2, 7.3, 1.1 Hz, 1H), 7.43 (ddt, *J* = 8.3, 7.2, 1.0 Hz, 1H), 3.13 – 2.98 (m, 2H), 2.71 (s, 3H), 2.02 (s, 3H), 1.57 – 1.45 (m, 2H), 1.32 (h, *J* = 7.4 Hz, 2H), 0.86 (t, *J* = 7.3 Hz, 3H).

^{13}C {^{1}H} NMR (126 MHz, CDCl$_3$) δ (ppm): 170.9, 159.4, 153.8, 135.7, 126.4, 126.0, 123.6, 122.2, 78.1, 41.4, 31.4, 26.6, 20.2, 19.3, 13.7.

HRMS (ESI) *m/z:* $[M+H-C_5H_9NO]^+$ calculated for $C_{10}H_{11}N_2S$, 191.0637; found, 191.0634.

7.2.1.10 4-(benzo[d]thiazol-2-yl)-1-isopropyl-3,4-dimethyl-1,3-diazetidin-2-one (3k)

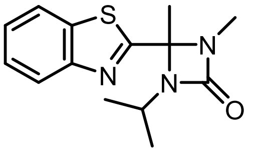

3k

Synthesized following **METHOD A**, starting from amino ester **4k** (55.7 mg, 0.20 mmol, 1.0 equiv.) and hydroxylamine hydrochloride **5a** (29.3 mg, 0.30 mmol, 1.5 equiv.). Purification by column chromatography (SiO_2, hexane/EtOAc = 15:1 → 3:1) afforded 1,3-diazetidin-2-one **3k** (36 mg, 0.13 mmol, 66 % yield) as a colorless oil.

R$_f$ = 0.53 (hexane/EtOAc = 2:1)

^{1}H NMR (500 MHz, CDCl$_3$) δ (ppm): 8.04 – 8.02 (m, 1H), 7.91 – 7.90 (m, 1H), 7.50 (ddd, *J* = 8.3, 7.1, 1.3 Hz, 1H), 7.43 (ddd, *J* = 8.3, 7.2, 1.2 Hz, 1H), 3.48 (hept, *J* = 6.6 Hz, 1H), 2.69 (s, 3H), 2.05 (s, 3H), 1.23 (d, *J* = 2.3 Hz, 3H), 1.22 (d, *J* = 2.3 Hz, 3H).

^{13}C {^{1}H} NMR (126 MHz, CDCl$_3$) δ (ppm): 171.6, 158.3, 153.8, 135.8, 126.3, 126.0, 123.6, 122.2, 77.8, 46.1, 26.4, 22.1, 22.0, 19.9.

HRMS (ESI) *m/z:* $[M+H-C_4H_7NO]^+$ calculated for $C_{10}H_{11}N_2S$, 191.0637; found, 191.0634.

7.2.1.11 4-(benzo[d]thiazol-2-yl)-1-(2,4-dimethoxybenzyl)-3,4-dimethyl-1,3-diazetidin-2-one (3l)

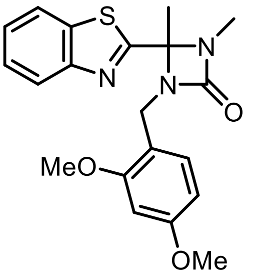

3l

Synthesized following **METHOD A**, starting from amino ester **4l** (77.3 mg, 0.20 mmol, 1.0 equiv.) and hydroxylamine hydrochloride **5a** (29.3 mg, 0.30 mmol, 1.5 equiv.). Purification by column chromatography (SiO_2, hexane/EtOAc = 15:1 → 5:1) afforded 1,3-diazetidin-2-one **3l** (42 mg, 0.11 mmol, 55 % yield) as a colorless oil.

R_f = 0.27 (hexane/EtOAc = 2:1)

^{1}H NMR (400 MHz, $CDCl_3$) δ (ppm): 8.01 – 7.98 (m, 1H), 7.85 – 7.83 (m, 1H), 7.48 (ddd, J = 8.3, 7.2, 1.3 Hz, 1H), 7.39 (ddd, J = 8.3, 7.2, 1.2 Hz, 1H), 7.19 (d, J = 8.3 Hz, 1H), 6.37 (dd, J = 8.3, 2.4 Hz, 1H), 6.26 (d, J = 2.5 Hz, 1H), 4.32 (d, J = 15.1 Hz, 1H), 4.21 (d, J = 15.2 Hz, 1H), 3.74 (s, 3H), 3.53 (s, 3H), 2.67 (s, 3H), 1.98 (s, 3H).

^{13}C {^{1}H} NMR (101 MHz, $CDCl_3$) δ (ppm): 171.2, 160.6, 159.1, 158.2, 153.6, 135.9, 131.0, 126.2, 125.8, 123.4, 122.1, 117.8, 103.9, 98.2, 78.8, 55.4, 55.0, 39.6, 26.6, 19.2.

HRMS (ESI) *m/z:* $[M+Na]^+$ calculated for $C_{20}H_{21}N_3NaO_3S$, 406.1196; found, 406.1199.

7.2.1.12 4-(benzo[d]thiazol-2-yl)-1,4-dimethyl-3-(4-methylbenzyl)-1,3-diazetidin-2-one (3m)

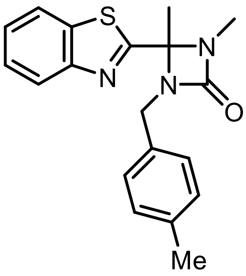

3m

Synthesized following **METHOD A**, starting from amino ester **4m** (68.1 mg, 0.20 mmol, 1.0 equiv.) and hydroxylamine hydrochloride **5a** (29.3 mg, 0.30 mmol, 1.5 equiv.). Purification by column chromatography (SiO_2, hexane/EtOAc = 15:1 → 3:1) afforded 1,3-diazetidin-2-one **3m** (46 mg, 0.14 mmol, 68 % yield) as a colorless oil.

R_f = 0.26 (hexane/EtOAc = 3:1)

^{1}H NMR (500 MHz, $CDCl_3$) δ (ppm): 8.01 (ddd, J = 8.1, 1.2, 0.6 Hz, 1H), 7.88 (ddd, J = 8.0, 1.3, 0.7 Hz, 1H), 7.50 (ddd, J = 8.3, 7.2, 1.3 Hz, 1H), 7.42 (ddd, J = 8.2, 7.2, 1.2 Hz, 1H), 7.13 – 7.11 (m, 2H), 7.03 – 7.01 (m, 2H), 4.32 (d, J = 15.3 Hz, 1H), 4.11 (d, J = 15.4 Hz, 1H), 2.75 (s, 3H), 2.27 (s, 3H), 1.92 (s, 3H).

^{13}C {^{1}H} NMR (126 MHz, $CDCl_3$) δ (ppm): 170.5, 158.9, 153.7, 137.2, 135.8, 133.8, 129.2, 128.4, 126.3, 125.9, 123.6, 122.1, 78.7, 45.3, 26.6, 21.2, 19.5.

HRMS (ESI) *m/z:* $[M+Na]^+$ calculated for $C_{19}H_{19}N_3NaOS$, 360.1141; found, 360.1145.

7.2.1.13 4-(benzo[d]thiazol-2-yl)-1,4-dimethyl-3-(4-(trifluoromethyl)benzyl)-1,3-diazetidin-2-one (3n) and 5-(benzo[d]thiazol-2-yl)-1,5-dimethyl-4-(4-(trifluoromethyl)phenyl)imidazolidin-2-one (7n)

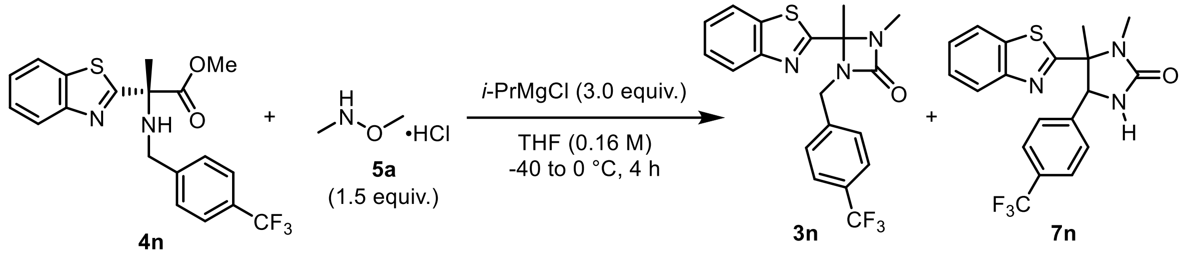

Synthesized following **METHOD A**, starting from amino ester **4n** (78.9 mg, 0.20 mmol, 1.0 equiv.) and hydroxylamine hydrochloride **5a** (29.3 mg, 0.30 mmol, 1.5 equiv.). Purification by column chromatography (SiO_2, hexane/EtOAc = 15:1 → 5:1) afforded 1,3-diazetidin-2-one **3n** (32 mg, 0.08 mmol, 41 % yield) as a colorless oil and imidazolidin-2-one **7n** (21 mg, 0.05 mmol, 27 %) as colorless oil.

Analytical data for **3n**:

R_f = 0.38 (hexane/EtOAc = 3:1)

^{1}H NMR (400 MHz, $CDCl_3$) δ (ppm): 8.01 – 7.99 (m, 1H), 7.87 (ddd, *J* = 8.1, 1.2, 0.7 Hz, 1H), 7.51 (ddd, *J* = 8.4, 7.3, 1.3 Hz, 1H), 7.45 (d, *J* = 8.1 Hz, 2H), 7.43 (ddd, *J* = 8.4, 7.2, 1.3 Hz, 1H), 7.34 (d, *J* = 8.0 Hz, 2H), 4.35 (d, *J* = 15.8 Hz, 1H), 4.18 (d, *J* = 15.9 Hz, 1H), 2.82 (s, 3H), 1.96 (s, 3H).

^{13}C {^{1}H, ^{19}F} NMR (101 MHz, $CDCl_3$) δ (ppm): 169.9, 158.7, 153.7, 141.1, 135.6, 129.8, 128.6, 126.6, 126.2, 125.5, 124.2, 123.6, 122.2, 78.8, 45.2, 26.6, 19.6.

^{19}F NMR (376 MHz, $CDCl_3$) δ (ppm): -62.41.

HRMS (ESI) *m/z:* $[M+H]^+$ calculated for $C_{19}H_{17}F_3N_3OS$, 392.1039; found, 392.1042.

Analytical data for **7n**:

R_f = 0.16 (hexane/EtOAc = 3:1)

^{1}H NMR (400 MHz, $CDCl_3$) δ (ppm): 8.06 (dd, *J* = 8.1, 1.2 Hz, 1H), 7.96 (dd, *J* = 8.0, 1.3 Hz, 1H), 7.63 (d, *J* = 7.8 Hz, 2H), 7.54 (ddd, *J* = 8.4, 7.3, 1.3 Hz, 1H), 7.46 (td, *J* = 7.6, 7.2, 1.2 Hz, 1H), 7.38 (d, *J* = 8.0 Hz, 2H), 5.35 (s, 1H), 5.13 (s, 1H), 2.82 (s, 3H), 1.33 (s, 3H).

^{13}C {^{1}H, ^{19}F} NMR (101 MHz, $CDCl_3$) δ (ppm): 174.7, 161.0, 153.3, 140.4, 135.7, 131.1, 127.8, 126.6, 126.0, 125.8, 124.0, 123.8, 122.1, 68.6, 65.3, 27.0, 18.3.

^{19}F NMR (376 MHz, $CDCl_3$) δ (ppm): -62.51.

HRMS (ESI) *m/z:* $[M-H]^-$ calculated for $C_{19}H_{15}F_3N_3OS$, 390.0882; found, 390.0886.

7.2.1.14 tert-butyl 2-(benzo[d]thiazol-2-yl)-1-(methoxy(methyl)amino)-1-oxopropan-2-yl)carbamate (1p)

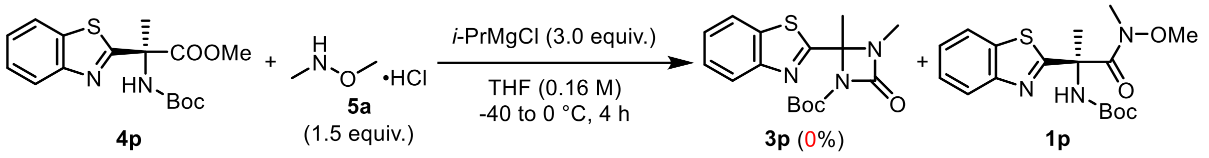

Synthesized following **METHOD A**, starting from amino ester **4p** (27.0 mg, 0.08 mmol, 1.0 equiv.) and hydroxylamine hydrochloride **5a** (11.7 mg, 0.12 mmol, 1.5 equiv.). Purification by column chromatography (SiO_2, hexane/EtOAc = 15:1 → 3:1) afforded no 1,3-diazetidin-2-one **3p**, but Weinreb amide **1p** (10 mg, 0.03 mmol, 35 % yield) as a colorless oil.

R_f = 0.41 (hexane/EtOAc = 2:1)

^{1}H NMR (400 MHz, $CDCl_3$) δ (ppm): 8.03 – 8.01 (m, 1H), 7.87 – 7.85 (m, 1H), 7.47 (ddd, *J* = 8.3, 7.2, 1.3 Hz, 1H), 7.38 (ddd, *J* = 8.2, 7.2, 1.2 Hz, 1H), 6.90 (s, 1H), 3.20 (s, 3H), 3.05 (s, 3H), 2.11 (s, 3H), 1.37 (s, 9H).

^{13}C {^{1}H} NMR (126 MHz, $CDCl_3$) δ (ppm): 172.1, 170.2, 153.7, 152.2, 135.4, 126.2, 125.5, 123.4, 121.9, 80.1, 62.4, 60.4, 34.2, 28.4, 24.0.

HRMS (ESI) *m/z:* $[M+H]^+$ calculated for $C_{17}H_{24}N_3O_4S$, 366.1482; found, 366.1479.

7.2.1.15 2-amino-2-(benzo[d]thiazol-2-yl)-N-methoxy-N-methylpropanamide (1q)

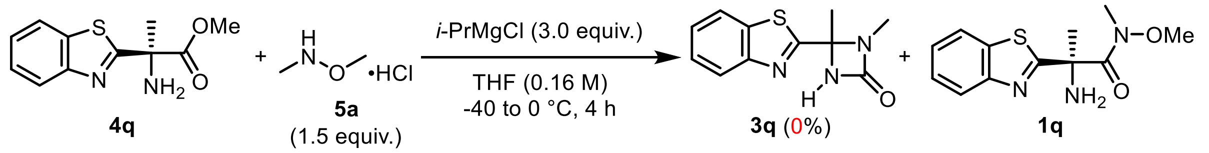

Synthesized following **METHOD A**, starting from amino ester **4q** (47 mg, 0.20 mmol, 1.0 equiv.) and hydroxylamine hydrochloride **5a** (29.3 mg, 0.30 mmol, 1.5 equiv.). Purification by column chromatography (SiO_2, CH_2Cl_2/acetone = 100:1 → 10:1) afforded no 1,3-diazetidin-2-one **3q**, but amide **1q** (9 mg, 0.03 mmol, 17 % yield) as a colorless oil.

R_f = 0.36 (CH_2Cl_2/acetone = 10:1)

^{1}H NMR (500 MHz, $CDCl_3$) δ (ppm): 7.99 (dd, J = 8.3, 1.1 Hz, 1H), 7.90 (dd, J = 8.0, 1.2 Hz, 1H), 7.45 (ddd, J = 8.4, 7.2, 1.3 Hz, 1H), 7.36 (td, J = 7.6, 1.2 Hz, 1H), 3.23 (s, 3H), 2.89 (s, 3H), 2.49 (s, 2H), 1.75 (s, 3H).

^{13}C {^{1}H} NMR (126 MHz, $CDCl_3$) δ (ppm): 178.8, 173.4, 153.6, 134.8, 126.1, 125.0, 123.0, 121.8, 61.6, 59.7, 33.4, 27.7.

HRMS (ESI) *m/z:* $[M+H]^+$ calculated for $C_{12}H_{16}N_3O_2S$, 266.0958; found, 266.0955.

7.2.1.16 1-benzyl-3-methoxy-3-methyl-1-(1-(thiazol-2-yl)ethyl)urea (8r)

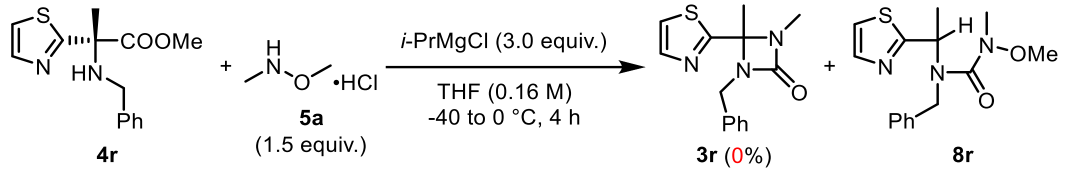

Synthesized following **METHOD A**, starting from amino ester **4r** (55 mg, 0.20 mmol, 1.0 equiv.) and hydroxylamine hydrochloride **5a** (29.3 mg, 0.30 mmol, 1.5 equiv.). Purification by column chromatography (SiO_2, CH_2Cl_2/acetone = 300:1 → 100:1) afforded no 1,3-diazetidin-2-one **3r**, but amide **8r** (13 mg, 0.04 mmol, 21 % yield) as a colorless oil.

$\mathbf{R_f}$ = 0.17 (CH_2Cl_2/acetone = 100:1)

^{1}H NMR (400 MHz, $CDCl_3$) δ (ppm): 7.71 (d, *J* = 3.2 Hz, 1H), 7.30 (d, *J* = 3.2 Hz, 1H), 7.28 – 7.17 (m, 5H), 5.49 (q, *J* = 7.0 Hz, 1H), 4.56 (d, *J* = 16.1 Hz, 1H), 4.32 (d, *J* = 16.2 Hz, 1H), 3.47 (s, 3H), 3.01 (s, 3H), 1.71 (d, *J* = 7.1 Hz, 3H).

^{13}C {^{1}H} NMR (126 MHz, $CDCl_3$) δ (ppm): 171.4, 163.0, 142.1, 138.9, 128.3, 127.2, 126.8, 119.9, 59.3, 55.6, 49.2, 36.0, 18.2.

HRMS (ESI) *m/z:* $[M+H]^+$ calculated for $C_{15}H_{20}N_3O_2S$, 306.1271; found, 306.1269.

7.2.1.17 1-benzyl-3-methoxy-3-methyl-1-(1-(oxazol-2-yl)ethyl)urea (8s)

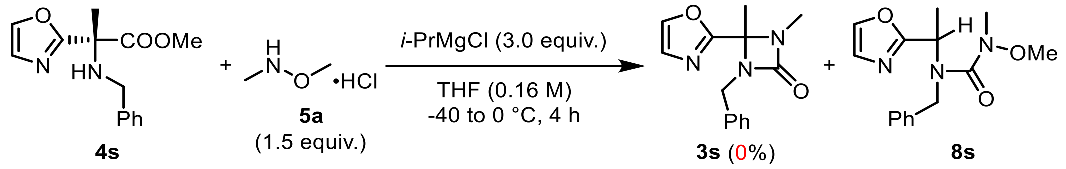

Synthesized following **METHOD A**, starting from amino ester **4s** (52 mg, 0.20 mmol, 1.0 equiv.) and hydroxylamine hydrochloride **5a** (29.3 mg, 0.30 mmol, 1.5 equiv.). Purification by column chromatography (SiO_2, hexane/EtOAc = 10:1 → 1:1) afforded no 1,3-diazetidin-2-one **3s**, but urea **8s** (23 mg, 0.08 mmol, 40 % yield) as a colorless oil.

$\mathbf{R_f}$ = 0.42 (hexane/EtOAc = 1:1)

^{1}H NMR (500 MHz, $CDCl_3$) δ (ppm): 7.54 (s, 1H), 7.25 – 7.16 (m, 5H), 7.00 (s, 1H), 5.31 (q, *J* = 7.1 Hz, 1H), 4.55 (d, *J* = 16.2 Hz, 1H), 4.33 (d, *J* = 16.2 Hz, 1H), 3.48 (s, 3H), 3.01 (s, 3H), 1.65 (d, *J* = 7.0 Hz, 3H).

^{13}C {^{1}H} NMR (126 MHz, $CDCl_3$) δ (ppm): 163.9, 162.9, 138.9, 138.5, 128.3, 127.2, 127.1, 126.8, 59.1, 51.6, 49.2, 36.0, 16.6.

HRMS (ESI) *m/z:* $[M+H]^+$ calculated for $C_{15}H_{20}N_3O_3$, 290.1499; found, 290.1496.

7.2.1.18 4-(benzo[d]thiazol-2-yl)-1-benzyl-3-(3-hydroxypropyl)-4-methyl-1,3-diazetidin-2-one (3w)

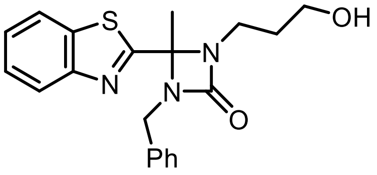

3w

Synthesized following **METHOD A**, starting from amino ester **4a** (65.3 mg, 0.20 mmol, 1.0 equiv.) and hydroxylamine hydrochloride **5c** (32.9 mg, 0.30 mmol, 1.5 equiv.). Purification by column chromatography (SiO_2, hexane/EtOAc = 10:1 → 2:1) afforded 1,3-diazetidin-2-one **3w** (50 mg, 0.14 mmol, 68 % yield) as a colorless oil.

R_f = 0.25 (cyclohexane/EtOAc = 1:1)

^{1}H NMR (500 MHz, $CDCl_3$) δ (ppm): 8.04 – 7.99 (m, 1H), 7.88 (ddd, *J* = 8.0, 1.2, 0.6 Hz, 1H), 7.51 (ddd, *J* = 8.3, 7.2, 1.3 Hz, 1H), 7.44 (ddd, *J* = 8.3, 7.3, 1.2 Hz, 1H), 7.28 – 7.19 (m, 5H), 4.37 (d, *J* = 15.4 Hz, 1H), 4.18 (d, *J* = 15.4 Hz, 1H), 3.82 – 3.71 (m, 2H), 3.25 (t, *J* = 6.6 Hz, 2H), 2.69 (brs, 1H), 1.98 (s, 3H), 1.78 (dtdd, *J* = 20.5, 14.0, 6.8, 5.2 Hz, 2H).

^{13}C {^{1}H} NMR (126 MHz, $CDCl_3$) δ (ppm): 170.4, 159.5, 153.6, 136.7, 135.6, 128.6, 128.5, 127.6, 126.5, 126.1, 123.6, 122.1, 79.1, 59.7, 45.6, 37.9, 32.1, 20.2.

HRMS (ESI) *m/z:* $[M+H]^+$ calculated for $C_{20}H_{22}N_3O_2S$, 368.1427; found, 368.1424.

7.2.1.19 4-(benzo[d]thiazol-2-yl)-1-benzyl-3-(4-hydroxybutyl)-4-methyl-1,3-diazetidin-2-one (3x)

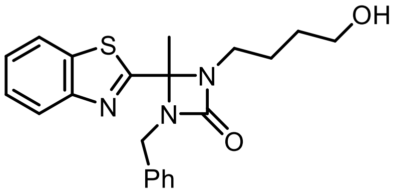

3x

Synthesized following **METHOD A**, starting from amino ester **4a** (65.3 mg, 0.20 mmol, 1.0 equiv.) and hydroxylamine hydrochloride **5d** (37.1 mg, 0.30 mmol, 1.5 equiv.). Purification by column chromatography (SiO_2, hexane/EtOAc = 5:1 → 1:2) afforded 1,3-diazetidin-2-one **3x** (53 mg, 0.14 mmol, 70 % yield) as a colorless oil.

R_f = 0.32 (cyclohexane/EtOAc = 1:2)

^{1}H NMR (500 MHz, $CDCl_3$) δ (ppm): 8.01 – 7.99 (m, 1H), 7.87 – 7.85 (m, 1H), 7.49 (ddd, *J* = 8.3, 7.1, 1.3 Hz, 1H), 7.42 (ddd, *J* = 8.4, 7.1, 1.2 Hz, 1H), 7.25 – 7.17 (m, 5H), 4.34 (d, *J* = 15.4 Hz, 1H), 4.14 (d, *J* = 15.5 Hz, 1H), 3.64 – 3.59 (m, 2H), 3.14 (t, *J* = 6.7 Hz, 2H), 1.96 (s, 3H), 1.67 – 1.59 (m, 4H).

$^{13}C\{^{1}H\}$ NMR (126 MHz, $CDCl_3$) δ (ppm): 170.7, 159.0, 153.6, 136.9, 135.7, 128.6, 128.5, 127.6, 126.4, 126.0, 123.6, 122.1, 78.9, 62.4, 45.5, 41.3, 30.1, 25.8, 20.1.

HRMS (ESI) *m/z:* $[M+H]^+$ calculated for $C_{21}H_{24}N_3O_2S$, 382.1584; found, 382.1588.

7.2.1.20 4-(benzo[d]thiazol-2-yl)-1,3-dibenzyl-4-methyl-1,3-diazetidin-2-one (3y)

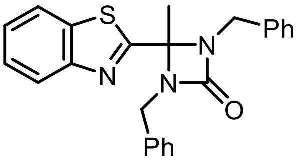

3y

Synthesized following **METHOD B**, starting from amino ester **4a** (65.3 mg, 0.20 mmol, 1.0 equiv.) and hydroxylamine **5h** (41.2 mg, 0.30 mmol, 1.5 equiv.). Purification by column chromatography (SiO_2, cyclohexane/EtOAc = 15:1 → 5:1) afforded 1,3-diazetidin-2-one **3y** (46 mg, 0.12 mmol, 58 % yield) as a colorless oil.

R_f = 0.46 (cyclohexane/EtOAc = 3:1)

^{1}H NMR (500 MHz, $CDCl_3$) δ (ppm): 7.99 – 7.97 (m, 1H), 7.84 – 7.82 (m, 1H), 7.49 (ddd, *J* = 8.3, 7.2, 1.3 Hz, 1H), 7.41 (ddd, *J* = 8.3, 7.2, 1.2 Hz, 1H), 7.28 – 7.18 (m, 10H), 4.37 (d, *J* = 15.4 Hz, 2H), 4.22 (d, *J* = 15.4 Hz, 2H), 1.88 (s, 3H).

$^{13}C\{^{1}H\}$ NMR (126 MHz, $CDCl_3$) δ (ppm): 170.4, 158.6, 153.5, 136.8, 135.9, 128.6, 128.5, 127.6, 126.3, 125.9, 123.6, 122.1, 79.4, 45.5, 20.3.

HRMS (ESI) *m/z:* $[M+Na]^+$ calculated for $C_{24}H_{21}N_3NaOS$, 422.1298; found, 422.1294.

7.2.1.21 4-(benzo[d]thiazol-2-yl)-1-benzyl-4-methyl-3-(3-phenylpropyl)-1,3-diazetidin-2-one (3z)

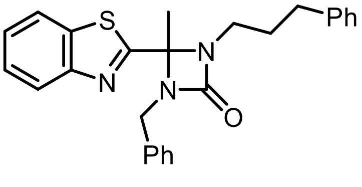

3z

Synthesized following **METHOD B**, starting from amino ester **4a** (65.3 mg, 0.20 mmol, 1.0 equiv.) and hydroxylamine **5i** (49.6 mg, 0.30 mmol, 1.5 equiv.). Purification by column chromatography (SiO_2, cyclohexane/EtOAc = 15:1 → 5:1) afforded 1,3-diazetidin-2-one **3z** (73 mg, 0.17 mmol, 85 % yield) as a colorless oil.

R_f = 0.47 (cyclohexane/EtOAc = 3:1)

^{1}H NMR (500 MHz, $CDCl_3$) δ (ppm): 8.01 – 8.00 (m, 1H), 7.88 – 7.86 (m, 1H), 7.50 (ddd, *J* = 8.2, 7.2, 1.3 Hz, 1H), 7.44 – 7.41 (m, 1H), 7.26 – 7.17 (m, 7H), 7.16 – 7.13 (m, 1H), 7.11 – 7.09 (m, 2H), 4.36 (d, *J* = 15.5 Hz, 1H), 4.15 (d, *J* = 15.4 Hz, 1H), 3.14 (t, *J* = 7.2 Hz, 2H), 2.65 (t, *J* = 7.7 Hz, 2H), 1.95 (s, 3H), 1.93 – 1.79 (m, 2H).

^{13}C {^{1}H} NMR (126 MHz, $CDCl_3$) δ (ppm): 170.8, 158.9, 153.6, 141.4, 136.9, 135.8, 128.6, 128.52 and 128.51 and 128.50 (peak overlaps), 127.6, 126.4, 126.1, 126.0, 123.6, 122.1, 78.8, 45.5, 41.1, 33.4, 31.0, 20.0.

HRMS (ESI) *m/z:* $[M+Na]^+$ calculated for $C_{26}H_{25}N_3NaOS$, 450.1611; found, 450.1613.

7.2.1.22 1-allyl-4-(benzo[d]thiazol-2-yl)-3-benzyl-4-methyl-1,3-diazetidin-2-one (3aa)

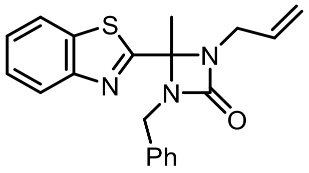

3aa

Synthesized following **METHOD A**, starting from amino ester **4a** (65.3 mg, 0.20 mmol, 1.0 equiv.) and hydroxylamine hydrochloride **5k** (37.1 mg, 0.30 mmol, 1.5 equiv.). Purification by column chromatography (SiO_2, hexane/Et_2O = 15:1 → 2:1) afforded 1,3-diazetidin-2-one **3aa** (32 mg, 0.09 mmol, 46 % yield) as a colorless oil.

R_f = 0.60 (cyclohexane/Et_2O = 1:3)

^{1}H NMR (400 MHz, $CDCl_3$) δ (ppm): 8.02 – 7.99 (m, 1H), 7.88 – 7.86 (m, 1H), 7.49 (ddd, *J* = 8.3, 7.2, 1.3 Hz, 1H), 7.42 (ddd, *J* = 8.3, 7.2, 1.3 Hz, 1H), 7.25 – 7.16 (m, 5H), 5.82 (dddd, *J* = 16.9, 10.1, 6.6, 5.9 Hz, 1H), 5.15 (dq, *J* = 17.1, 1.5 Hz, 1H), 5.06 (dq, *J* = 10.2, 1.3 Hz, 1H), 4.36 (d, *J* = 15.4 Hz, 1H), 4.12 (d, *J* = 15.5 Hz, 1H), 3.85 – 3.74 (m, 2H), 1.95 (s, 3H).

^{13}C {^{1}H} NMR (101 MHz, $CDCl_3$) δ (ppm): 170.6, 158.4, 153.6, 136.8, 135.8, 133.4, 128.5, 128.5, 127.6, 126.3, 126.0, 123.6, 122.1, 118.3, 79.1, 45.4, 44.4, 20.1.

HRMS (ESI) *m/z:* $[M+H-C_8H_7NO]^+$ calculated for $C_{12}H_{13}N_2S$, 217.0794; found, 217.0792.

7.2.1.23 4-(benzo[d]thiazol-2-yl)-1-benzyl-3-(2-(benzyloxy)ethyl)-4-methyl-1,3-diazetidin-2-one (3ab)

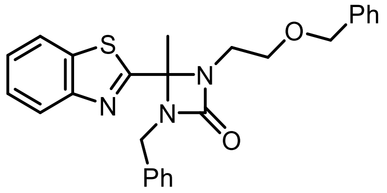

3ab

Synthesized following **METHOD A**, starting from amino ester **4a** (65.3 mg, 0.20 mmol, 1.0 equiv.) and hydroxylamine hydrochloride **5l** (65.3 mg, 0.30 mmol, 1.5 equiv.). Purification by column chromatography (SiO_2, cyclohexane/EtOAc = 15:1 → 5:1) afforded 1,3-diazetidin-2-one **3ab** (50 mg, 0.11 mmol, 56 % yield) as a colorless oil.

R_f = 0.51 (cyclohexane/EtOAc = 2:1)

^{1}H NMR (400 MHz, $CDCl_3$) δ (ppm): 7.98 (dt, *J* = 8.2, 0.9 Hz, 1H), 7.85 (dt, *J* = 7.9, 1.0 Hz, 1H), 7.49 (ddd, *J* = 8.3, 7.2, 1.3 Hz, 1H), 7.41 (ddd, *J* = 8.4, 7.2, 1.2 Hz, 1H), 7.20 – 7.16 (m, 5H), 7.16 – 7.08 (m, 3H), 6.96 – 6.94 (m, 2H), 4.35 (d, *J* = 15.5 Hz, 1H), 4.31 (d, *J* = 11.5 Hz, 1H), 4.24 (d, *J* = 11.5 Hz, 1H), 3.98 (d, *J* = 15.5 Hz, 1H), 3.62 – 3.58 (m, 2H), 3.53 – 3.47 (m, 1H), 3.36 (ddd, *J* = 14.7, 7.3, 5.0 Hz, 1H), 1.94 (s, 3H).

^{13}C {^{1}H} NMR (126 MHz, $CDCl_3$) δ (ppm): 171.2, 158.5, 153.5, 137.6, 136.9, 135.8, 128.5, 128.4, 128.2, 127.7, 127.6, 127.5, 126.2, 125.9, 123.6, 122.0, 79.6, 73.3, 69.4, 45.4, 41.1, 19.8.

HRMS (ESI) *m/z:* $[M+H]^+$ calculated for $C_{26}H_{26}N_3O_2S$, 444.1740; found, 444.1738.

7.2.1.24 4-(benzo[d]thiazol-2-yl)-1-benzyl-3-(3-(benzyloxy)-2-((tert-butyldimethylsilyl)oxy)propyl)-4-methyl-1,3-diazetidin-2-one (3ad)

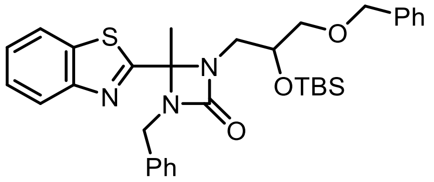

3ad

Synthesized following **METHOD B**, starting from amino ester **4a** (65.3 mg, 0.20 mmol, 1.0 equiv.) and hydroxylamine **5m** (97.7 mg, 0.30 mmol, 1.5 equiv.). Purification by column chromatography (SiO_2, CH_2Cl_2/acetone = 500:1 → 300:1) afforded inseparable mixture of two diastereomers (in ratio 2:1) of 1,3-diazetidin-2-one **3ad** (64 mg, 0.11 mmol, 54 % combined yield) as a colorless oil.

R_f = 0.46 (CH_2Cl_2/acetone = 300:1)

Major isomer (overlapping signals are marked with an asterisk):*

^{1}H NMR (500 MHz, $CDCl_3$) δ (ppm): 8.00 – 7.97 (m, 1H)*, 7.87 – 7.85 (m, 1H)*, 7.50 – 7.47 (m, 1H)*, 7.43 – 7.40 (m, 1H)*, 7.33 – 7.17 (m, 10H)*, 4.51 (d, *J* = 11.9 Hz, 1H), 4.48 (d, *J* = 11.8 Hz, 1H), 4.35 (d, *J* = 15.4 Hz, 1H), 4.25 (d, *J* = 15.5 Hz, 1H), 3.88 – 3.83 (m, 1H), 3.54 (dd, *J* = 9.7, 5.1 Hz, 1H), 3.44 (dd, *J* = 9.7, 5.7 Hz, 1H), 3.29 (dd, *J* = 14.6, 4.2 Hz, 1H), 3.08 (dd, *J* = 14.6, 5.9 Hz, 1H), 1.96 (s, 3H), 0.83 (s, 9H), -0.01 (s, 3H), -0.02 (s, 3H).

^{13}C {^{1}H} NMR (126 MHz, $CDCl_3$) δ (ppm): 170.5, 158.9, 153.5, 138.4*, 136.8, 135.9, 128.6*, 128.5*, 128.4*, 127.8, 127.6*, 127.6*, 126.3, 125.9, 123.7, 122.1, 79.4, 73.5, 72.4, 70.5*, 45.6, 44.8, 26.0, 20.2, 18.3, -4.6, -4.7.

HRMS (ESI) ***m/z:*** $[M+H]^+$ calculated for $C_{33}H_{42}N_3O_3SSi$, 588.2711; found, 588.2711.

Minor isomer (overlapping signals are marked with an asterisk):*

^{1}H NMR (500 MHz, $CDCl_3$) δ (ppm): 8.00 – 7.98 (m, 1H)*, 7.87 – 7.85 (m, 1H)*, 7.50 – 7.47 (m, 1H)*, 7.43 – 7.40 (m, 1H)*, 7.33 – 7.17 (m, 10H)*, 4.54 (d, *J* = 11.9 Hz, 1H), 4.49 (d, *J* = 11.9 Hz, 1H), 4.39 (d, *J* = 15.5 Hz, 1H), 4.00 (d, *J* = 15.5 Hz, 1H), 3.98 – 3.95 (m, 1H), 3.64 (dd, *J* = 9.6, 5.5 Hz, 1H), 3.47 – 3.44 (m, 1H)*, 3.35 (dd, *J* = 14.4, 3.7 Hz, 1H), 3.19 (dd, *J* = 14.4, 5.7 Hz, 1H), 1.92 (s, 3H), 0.65 (d, *J* = 1.0 Hz, 9H), -0.05 (s, 3H), -0.09 (s, 3H).

^{13}C {^{1}H} NMR (126 MHz, $CDCl_3$) δ (ppm): 170.7, 159.1, 153.3, 138.4*, 136.8, 136.0, 128.5*, 128.5, 128.4*, 127.9, 127.6*, 127.6*, 126.2, 125.9, 123.6, 122.0, 79.4, 73.5, 72.0, 70.5*, 45.5, 44.6, 25.7, 19.9, 18.0, -4.7, -4.8.

7.3 Additional Experiments and Post-modifications

7.3.1 Stereocenter adjacent to oxygen

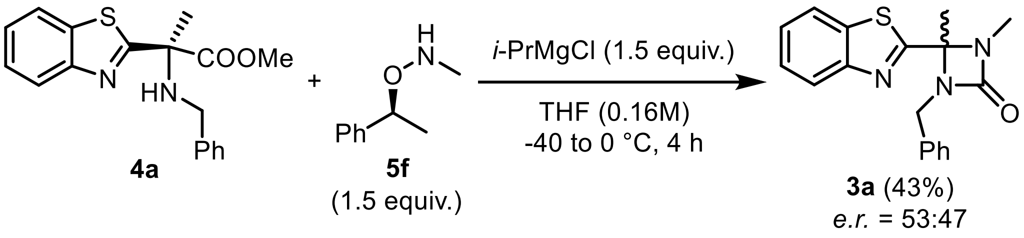

Following **METHOD B**, starting from amino ester **4a** (65.3 mg, 0.20 mmol, 1.0 equiv.) and hydroxylamine **5f** (45.4 mg, 0.30 mmol, 1.5 equiv.). Purification by column chromatography (SiO_2, hexane/EtOAc = 15:1 to 5:1) afforded 1,3-diazetidin-2-one **3a** (28 mg, 0.09 mmol, 43 % yield) as a racemic mixture (*e.r.* 53:47).

CHIRAL HPLC (SA, Hexane : iPrOH = 95:5, 0.5 mL/min, 298 K, 220 nm): t_{R1} = 21.52 min, t_{R2} = 22.68 min.

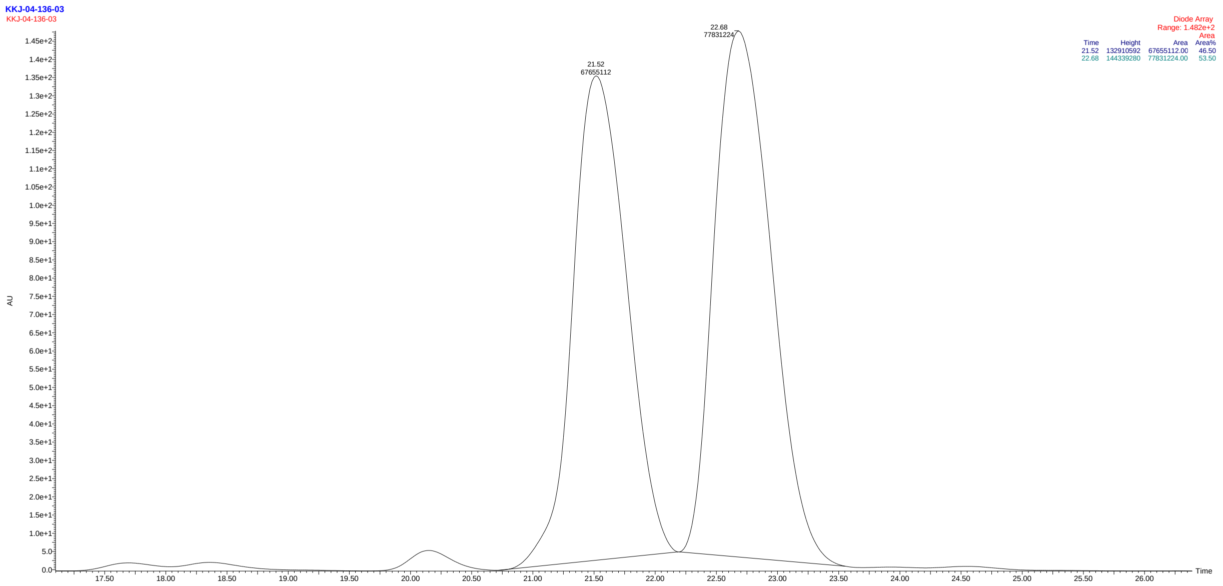

7.3.2 Stereocenter adjacent to nitrogen

7.3.2.1 4-(benzo[d]thiazol-2-yl)-3-benzyl-4,5-dimethyl-1-phenylimidazolidin-2-one (9)

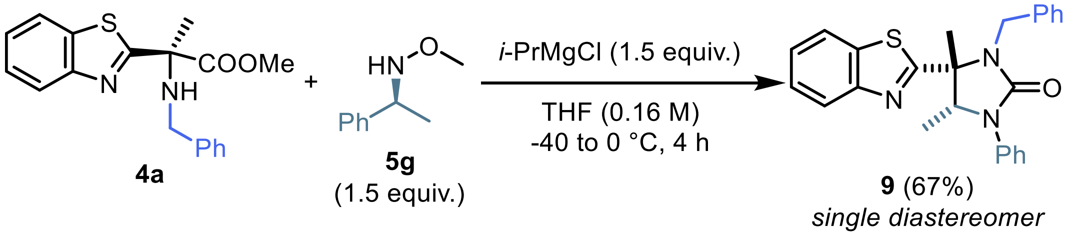

Synthesized following **METHOD B**, starting from amino ester **4a** (65.3 mg, 0.20 mmol, 1.0 equiv.) and hydroxylamine **5g** (45.4 mg, 0.30 mmol, 1.5 equiv.). Purification by column chromatography (SiO_2, hexane/EtOAc = 15:1 → 7:1) afforded a single diastereomer of imidazolidin-2-one **9** (55 mg, 0.13 mmol, 67 % yield) as a colorless oil.

$\mathbf{R_f}$ = 0.47 (hexane/EtOAc = 3:1)

^{1}H NMR (400 MHz, CDCl$_3$) δ (ppm): 8.06 (ddd, *J* = 8.2, 1.2, 0.6 Hz, 1H), 7.91 (ddd, *J* = 8.0, 1.4, 0.6 Hz, 1H), 7.51 (ddd, *J* = 8.3, 7.2, 1.3 Hz, 1H), 7.45 – 7.41 (m, 1H), 7.40 – 7.34 (m, 5H), 7.29 – 7.21 (m, 4H), 7.18 (tt, *J* = 6.5, 1.7 Hz, 1H), 5.05 (d, *J* = 15.7 Hz, 1H), 4.30 (q, *J* = 6.4 Hz, 1H), 3.81 (d, *J* = 15.8 Hz, 1H), 1.82 (s, 3H), 0.95 (d, *J* = 6.5 Hz, 3H).

^{13}C {^{1}H} NMR (101 MHz, CDCl$_3$) δ (ppm): 172.4, 158.7, 153.1, 139.2, 137.8, 135.6, 129.0, 128.6, 128.1, 127.3, 126.3, 125.8, 125.3, 124.1, 123.6, 122.0, 66.9, 61.4, 46.1, 24.3, 13.6.

HRMS (ESI) *m/z:* [M+H]$^+$ calculated for $C_{25}H_{24}N_3OS$, 414.1635; found, 414.1631.

7.3.2.2 2-(2-(benzo[d]thiazol-2-yl)-3-benzyl-2-methyl-4-oxo-1,3-diazetidin-1-yl)acetaldehyde (10) and 2-(benzo[d]thiazol-2-yl)-3-benzyl-2-methyl-4-oxo-1,3-diazetidine-1-carbaldehyde (11)

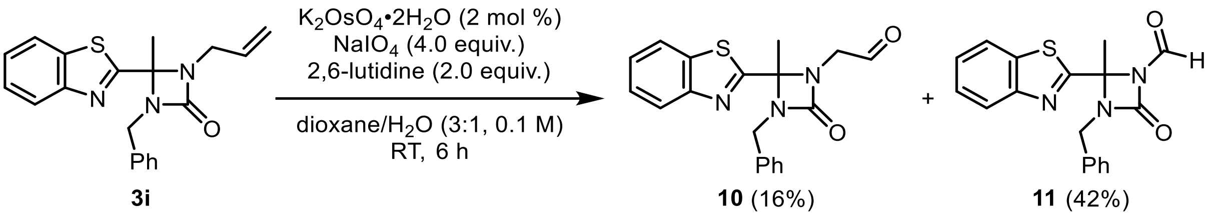

Alkene **3i** (97.8 mg, 0.28 mmol, 1.0 equiv.) was dissolved in mixture of dioxane/water (3:1, 2.8 mL, 0.1 M) and lutidine (66 µL, 0.56 mmol, 2.0 equiv.), $K_2OsO_4{\cdot}2H_2O$ (2.1 mg, 0.056 mmol, 2 mol %) and $NaIO_4$ (244 mg, 1.12 mmol, 4.0 equiv.) were added. Reaction was stirred at room temperature for 6 h, the it was quenched by addition of water and CH_2Cl_2. The aqueous phase was extracted with CH_2Cl_2 (3x10 mL) and the combined organic phases were washed with brine, dried over Na_2SO_4 and the solvents were removed under reduced pressure. The residue was purified by gradient column chromatography (SiO_2, cyclohexane/EtOAc = 10:1 → 1:1) to afford the aldehyde **10** (16 mg, 0.046 mmol, 16 % yield) as a colorless oil and carbaldehyde **11** (40 mg, 0.119 mmol, 42 % yield) as colorless oil.

Analytical data for **10**:

$\mathbf{R_f}$ = 0.22 (cyclohexane/EtOAc = 1:1)

^{1}H NMR (400 MHz, CDCl$_3$) δ (ppm): 9.61 (t, *J* = 1.0 Hz, 1H), 8.00 (ddd, *J* = 8.2, 1.2, 0.7 Hz, 1H), 7.86 (ddd, *J* = 8.0, 1.3, 0.7 Hz, 1H), 7.50 (ddd, *J* = 8.3, 7.2, 1.3 Hz, 1H), 7.45 – 7.40 (m, 1H), 7.29 – 7.20 (m, 5H), 4.42 (d, *J* = 15.4 Hz, 1H), 4.26 (d, *J* = 15.5 Hz, 1H), 4.01 (dd, *J* = 19.2, 1.2 Hz, 1H), 3.87 (dd, *J* = 19.2, 0.8 Hz, 1H), 1.98 (s, 3H).

^{13}C {^{1}H} NMR (101 MHz, CDCl$_3$) δ (ppm): 196.8, 169.4, 157.9, 153.5, 136.3, 135.6, 128.7, 128.6, 127.8, 126.5, 126.2, 123.8, 122.1, 79.4, 51.1, 45.7, 20.3

HRMS (ESI) *m/z:* [M+H]$^+$ calculated for $C_{19}H_{18}N_3O_2S$, 352.1114; found, 352.1110.

Analytical data for **11**:

R_f = 0.58 (cyclohexane/EtOAc = 1:1)

^{1}H NMR (400 MHz, $CDCl_3$) δ (ppm): 8.70 (s, 1H), 8.06 (ddd, *J* = 8.2, 1.2, 0.6 Hz, 1H), 7.87 (ddd, *J* = 8.0, 1.2, 0.6 Hz, 1H), 7.53 (ddd, *J* = 8.3, 7.2, 1.3 Hz, 1H), 7.45 (ddd, *J* = 8.4, 7.2, 1.2 Hz, 1H), 7.25 – 7.20 (m, 5H), 4.58 (d, *J* = 15.4 Hz, 1H), 4.27 (d, *J* = 15.5 Hz, 1H), 2.10 (s, 3H).

^{13}C {^{1}H} NMR (101 MHz, $CDCl_3$) δ (ppm): 165.8, 154.4, 153.1, 149.4, 135.6, 135.1, 128.8, 128.5, 128.2, 126.7, 126.5, 124.1, 122.1, 79.1, 44.6, 21.4.

HRMS (ESI) *m/z:* $[M+H]^+$ calculated for $C_{18}H_{16}N_3O_2S$, 338.0958; found, 338.0955.

7.3.2.3 1-(benzo[d]thiazol-2-yl)-N-methylethan-1-amine (12) and N-benzylformamide (13)

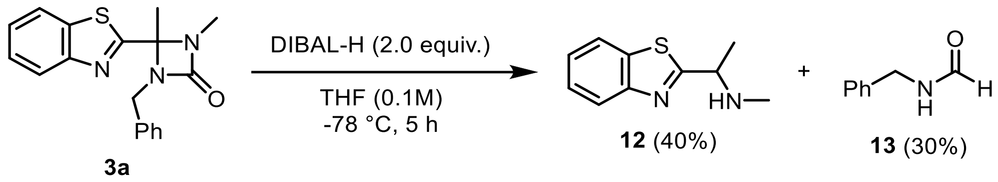

To a solution of diazetidinone **3a** (23 mg, 0.07 mmol, 1.0 equiv.) in CH_2Cl_2 (1.4 mL, 0.05M) was added diisobutylaluminium hydride (1 mol/L in CH_2Cl_2, 0.14 mL, 0.14 mmol, 2.0 equiv.) at -78 °C under argon atmosphere. The resulting mixture was stirred at that temperature for 5 h. The reaction mixture was quenched by saturated aq. solution NH_4Cl (3 mL), filtered through short pad of celite and rinsed with EtOAc. The filtrate was extracted with EtOAc (3x10 mL). The combined organic layers were washed with brine, dried over Na_2SO_4, filtered and concentrated under reduced pressure. The residue was purified by gradient column chromatography (SiO_2, cyclohexane/EtOAc = 10:1 → 1:1) to afford 9 mg of the mixture of the amine **12** (40 % yield) and formamide **13** (30 % yield) as a colorless oil in ratio 1.7 : 1 (amine : formamide).

Analytical data for **12**:

^{1}H NMR (500 MHz, $CDCl_3$) δ (ppm): 7.97 (dd, *J* = 8.4, 1.0 Hz, 1H), 7.88 (dd, *J* = 8.1, 1.2 Hz, 1H), 7.47 – 7.44 (m, 1H), 7.38 – 7.34 (m, 1H), 4.15 (q, *J* = 6.7 Hz, 1H), 2.49 (s, 3H), 1.56 (d, *J* = 6.7 Hz, 3H).

^{13}C {^{1}H} NMR (126 MHz, $CDCl_3$) δ (ppm): 178.8, 153.4, 135.0, 125.9, 124.9, 122.8, 122.0, 59.0, 34.9, 23.1.

HRMS (ESI) *m/z:* $[M+H]^+$ calculated for $C_{10}H_{13}N_2S$, 193.0794; found, 193.0791.

Analytical data for **13**:

Mixture of rotamers was observed, ratio 6.3:1.

^{1}H NMR (500 MHz, CDCl$_3$) δ (ppm): 8.28 (s, 1H), 7.35 – 7.33 (m, 2H), 7.30 (dq, J = 7.0, 3.0 Hz, 3H), 4.50 (d, J = 5.9 Hz, 3H).

^{13}C {^{1}H} NMR (101 MHz, CDCl$_3$) δ (ppm): 161.0, 137.6, 129.0, 128.0, 127.9, 42.4.

The analytical data are consistent with those reported in the literature.[30]

7.3.2.4 5-(benzo[d]thiazol-2-yl)-5-benzyl-1-methyl-4-phenylimidazolidin-2-one (7a)

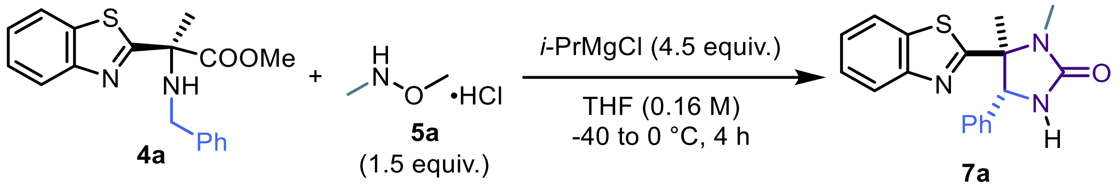

Amino ester **4a** (22.8 mg, 0.07 mmol, 1.0 equiv) was dissolved in dry THF (0.44 mL, 0.16 M) containing a suspension of hydroxylamine hydrochloride **5a** (10.2 mg, 0.11 mmol, 1.5 equiv) in a flame-dried round-bottom flask under an argon atmosphere. The reaction mixture was cooled to –40 °C, and *i*PrMgCl (2.0 M in THF, 0.158 mL, 0.32 mmol, 4.5 equiv) was added dropwise. The mixture was stirred while gradually warming from –40 °C to 0 °C over 4 h. The reaction was then quenched with sat. aq NH_4Cl and extracted with EtOAc (3 × 20 mL). The combined organic layers were washed with brine, dried over Na_2SO_4, and concentrated under reduced pressure. The crude residue (*d.r.* = 3.6:1, determined from the crude mixture) was purified by gradient column chromatography (SiO_2, CH_2Cl_2/acetone = 20:1 → 10:1) to afford imidazolidin-2-one **7a** as the major isomer (12.5 mg, 0.04 mmol, 55%) and a mixture of major and minor isomers (1:1.3 ratio; 6.5 mg, 0.02 mmol, 29%), combined yield is 19 mg (0.06 mmol, 84% yield).

Major isomer:

R_f = 0.49 (CH_2Cl_2/acetone = 9:1)

^{1}H NMR (500 MHz, CDCl$_3$) δ (ppm): 8.06 – 8.04 (m, 1H), 7.95 – 7.93 (m, 1H), 7.52 (ddd, J = 8.4, 7.2, 1.4 Hz, 1H), 7.45 (ddd, J = 8.3, 7.2, 1.2 Hz, 1H), 7.38 – 7.34 (m, 3H), 7.25 – 7.22 (m, 2H), 5.10 (s, 1H), 5.05 (s, 1H), 2.80 (s, 3H), 1.34 (s, 3H).

^{13}C {^{1}H} NMR (126 MHz, CDCl$_3$) δ (ppm): 175.3, 161.0, 153.3, 136.4, 135.7, 128.8, 128.8, 127.3, 126.4, 125.8, 123.7, 122.0, 68.7, 65.7, 26.8, 18.3.

HRMS (ESI) *m/z:* $[M+H]^+$ calculated for $C_{18}H_{18}N_3OS$, 324.1165; found, 324.1161.

Minor isomer (overlapping signals are marked with an asterisk):*

R_f = 0.32 (CH_2Cl_2/acetone = 9:1)

^{1}H NMR (500 MHz, CDCl$_3$) δ (ppm): 7.81 – 7.79 (m, 1H), 7.72 – 7.70 (m, 1H), 7.37 – 7.34 (m, 1H)*, 7.28 (td, *J* = 7.6, 1.2 Hz, 1H), 7.12 – 7.10 (m, 2H), 7.07 – 7.01 (m, 3H), 5.13 (s, 1H), 4.86 (s, 1H), 2.85 (s, 3H), 2.01 (s, 3H).

^{13}C {^{1}H} NMR (126 MHz, CDCl$_3$) δ (ppm): 171.2, 161.4, 153.3, 135.9, 135.5, 128.5, 128.2, 126.9, 125.8, 125.2, 123.2, 121.5, 69.2, 66.7, 26.9, 23.4.

7.3.2.5 5-(benzo[d]thiazol-2-yl)-5-benzyl-1-methyl-4-phenylimidazolidin-2-one (7b)

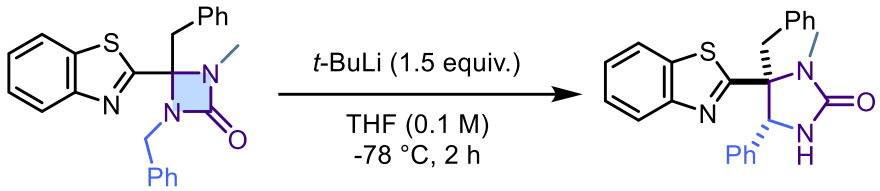

3b

7b (83%)
d.r. = 5.7:1

Diazetidinone **3b** (60 mg, 0.15 mmol, 1.0 equiv.) was dissolved in a flame-dried round-bottom flask in dry THF (1.5 mL, 0.1 M) under argon atmosphere. The reaction mixture was cooled to -78 °C and *t*-BuLi (1.7 mol/l in pentane, 0.12 mL, 0.23 mmol, 1.5 equiv.) was added dropwise. The mixture was stirred at -78 °C for 2 hours. After this time (or full consumption of starting material), the reaction was quenched by saturated aqueous solution of NH_4Cl and extracted with ethyl acetate (3x10 mL). The combined organic phases were washed with brine, dried over Na_2SO_4 and the solvents were removed under reduced pressure. The residue was purified by gradient column chromatography (SiO_2, cyclohexane/EtOAc = 10:1 → 1:1) to afford inseparable mixture of diastereomers of the imidazolidin-2-one **7b** (50 mg, 0.13 mmol, 83 % yield, *d.r.* = 5.7:1) as a colorless oil.

Major isomer (overlapping signals are marked with an asterisk):*

R_f = 0.31 (cyclohexane/EtOAc = 1:1)

^{1}H NMR (500 MHz, CDCl$_3$) δ (ppm): 8.17 (dt, *J* = 8.1, 0.9 Hz, 1H), 7.97 (dt, *J* = 7.9, 0.9 Hz, 1H), 7.59 (ddd, *J* = 8.3, 7.3, 1.3 Hz, 1H), 7.49 (ddd, *J* = 8.2, 7.2, 1.2 Hz, 1H), 7.35 – 7.30 (m, 3H)*, 7.21 – 7.19 (m, 2H)*, 7.13 – 7.03 (m, 5H)* , 5.12 (d, *J* = 1.7 Hz, 1H), 5.05 (d, *J* = 1.7 Hz, 1H), 3.36 (d, *J* = 14.3 Hz, 1H), 3.14 (d, *J* = 14.4 Hz, 1H), 2.49 (s, 3H).

^{13}C {^{1}H} NMR (126 MHz, CDCl$_3$) δ (ppm): 174.3, 161.3*, 153.0, 136.2, 136.0, 135.4, 130.9*, 128.8, 128.6, 128.3, 127.7, 126.9, 126.5*, 125.9, 123.9, 122.1, 72.2, 67.5, 38.9, 29.9.

HRMS (ESI) *m/z:* $[M+H]^+$ calculated for $C_{24}H_{22}N_3OS$, 400.1478; found, 400.1474.

Minor isomer (overlapping signals are marked with an asterisk):*

^{1}H NMR (500 MHz, CDCl$_3$) δ (ppm): 7.85 (dt, *J* = 8.2, 0.9 Hz, 1H), 7.74 (dt, *J* = 7.9, 1.1 Hz, 1H), 7.40 – 7.29 (m, 3H)*, 7.13 – 7.00 (m, 7H)*, 4.97 (d, *J* = 1.8 Hz, 1H), 4.76 (s, 1H), 3.90 (d, *J* = 15.3 Hz, 1H), 3.67 (d, *J* = 15.4 Hz, 1H), 3.01 (s, 3H).

^{13}C {^{1}H} NMR (126 MHz, $CDCl_3$) δ (ppm): 171.5, 161.3*, 152.8, 136.5, 135.5, 135.5, 130.9*, 129.1, 128.2, 128.1, 127.6, 127.1, 126.5*, 125.3, 123.3, 121.6, 72.9, 60.8, 39.4, 28.3.

7.4 Control and Mechanistic Experiments

7.4.1 Reaction with **S1** under standard reaction conditions

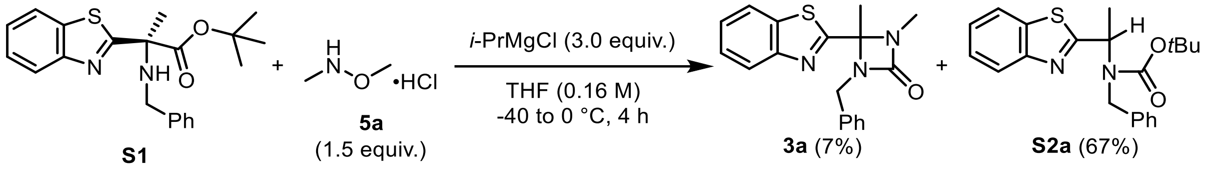

Following **METHOD A**, starting from amino ester **S1** (73.7 mg, 0.20 mmol, 1.0 equiv.) and hydroxylamine hydrochloride **5a** (29.3 mg, 0.30 mmol, 1.5 equiv.). Standard reaction work-up afforded 1,3-diazetidin-2-one **3a** (7 % NMR yield) and carbamate **S2a** (67 % NMR yield).

*Note: Carbamate S2a could not be isolated after column chromatography and was detected only in the crude reaction mixture with help of ^{1}H NMR spectroscopy. The structure determined by analogy with carbamates **6**.*

7.4.2 Preparing and isolating the carbamate **6a**

Methyl (1-(benzo[*d*]thiazol-2-yl)ethyl)(benzyl)carbamate (6a)

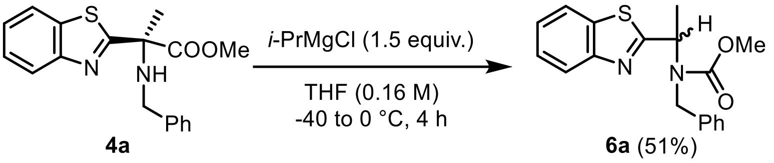

Amino ester **4a** (65.3 mg, 0.20 mmol, 1.0 equiv) was dissolved in dry THF (1.25 mL, 0.16 M) in a flame-dried round-bottom flask under an argon atmosphere. The reaction mixture was cooled to –40 °C, and *i*PrMgCl (2.0 M in THF, 0.30 mmol, 1.5 equiv) was added dropwise. The mixture was stirred while gradually warming from –40 °C to 0 °C over 4 h. Upon completion (TLC), the reaction was quenched with sat. aq NH_4Cl and extracted with EtOAc (3 × 20 mL). The combined organic layers were washed with brine, dried over Na_2SO_4, and concentrated under reduced pressure. Purification of the crude residue by gradient column chromatography (SiO_2, cyclohexane/EtOAc = 15:1 → 3:1) afforded carbamate **6a** (33 mg, 0.10 mmol, 51%) as a colorless oil.

R_f = 0.44 (cyclohexane/EtOAc = 3:1)

^{1}H NMR (400 MHz, DMSO-*d*$_6$, 80 °C) δ (ppm): 8.02 (ddd, *J* = 7.9, 1.3, 0.6 Hz, 1H), 7.96 (ddd, *J* = 8.1, 1.3, 0.6 Hz, 1H), 7.49 (ddd, *J* = 8.1, 7.2, 1.3 Hz, 1H), 7.41 (ddd, *J* = 8.0, 7.2, 1.3 Hz, 1H), 7.30 – 7.28 (m, 4H), 7.25 – 7.19 (m, 1H), 5.49 (q, *J* = 7.0 Hz, 1H), 4.65 (d, *J* = 16.1 Hz, 1H), 4.46 (d, *J* = 16.1 Hz, 1H), 3.66 (s, 3H), 1.64 (d, *J* = 7.0 Hz, 3H).

^{13}C {^{1}H} NMR (101 MHz, DMSO-*d*$_6$, 80 °C) δ (ppm): 172.5, 155.8, 152.2, 138.3, 134.5, 127.8, 126.8, 126.5, 125.6, 124.7, 122.3, 121.6, 54.7, 52.2, 48.5, 17.3.

HRMS (ESI) *m/z*: $[M+H]^+$ calculated for $C_{18}H_{19}N_2O_2S$, 327.1162; found, 327.1158.

7.4.3 Reaction of carbamate **6a** under standard conditions

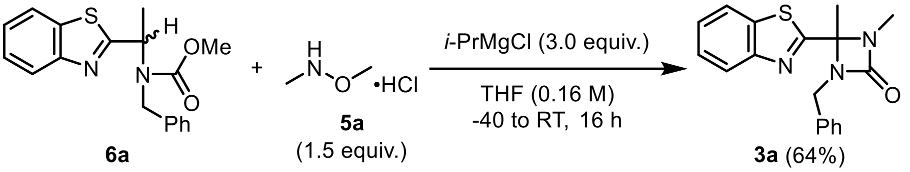

Synthesized following **METHOD A**, starting from carbamate **6a** (32.6 mg, 0.10 mmol, 1.0 equiv.) and hydroxylamine hydrochloride **5a** (14.6 mg, 0.15 mmol, 1.5 equiv.). Purification by column chromatography (SiO_2, hexane/EtOAc = 15:1 to 5:1) afforded 1,3-diazetidin-2-one **3a** (20.6 mg, 0.06 mmol, 64 % yield) as a colorless oil.

7.4.4 Transformation of carbamate **6a** to amine **S3**

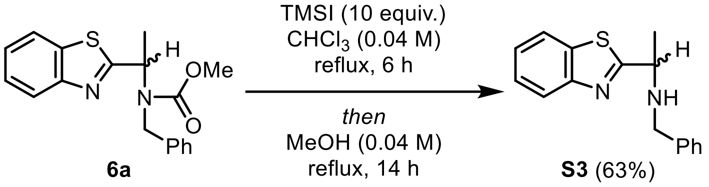

To a solution of carbamate **6a** (32.6 mg, 0.10 mmol, 1.0 equiv.) in $CHCl_3$ (2.5 mL, 0.04 M) was added TMSI (0.150 mL, 1.0 mmol, 10 equiv.) and the whole mixture was refluxed for 6 h. Then, it was cooled down to room temperature, treated with methanol (2.5 mL, 0.04 M) and refluxed again for 14 h. The reaction mixture was concentrated under reduced pressure to dryness and the residue was purified by gradient column chromatography (SiO_2, cyclohexane/EtOAc = 10:1 to 5:1) to afford the desired amine **S3** (17 mg, 0.06 mmol, 63 % yield) as a colorless oil.

7.4.4.1 1-(benzo[d]thiazol-2-yl)-N-benzylethan-1-amine (S3)

$\mathbf{R_f}$ = 0.46 (cyclohexane/EtOAc = 2:1)

^{1}H NMR (400 MHz, $CDCl_3$) δ (ppm): 8.00 – 7.97 (m, 1H), 7.91 – 7.89 (m, 1H), 7.47 (ddd, *J* = 8.3, 7.3, 1.2 Hz, 1H), 7.39 – 7.32 (m, 5H), 7.29 – 7.24 (m, 1H), 4.33 (q, *J* = 6.7 Hz, 1H), 3.90 (d, *J* = 13.1 Hz, 1H), 3.82 (d, *J* = 13.1 Hz, 1H), 1.59 (d, *J* = 6.7 Hz, 3H).

^{13}C {^{1}H} NMR (101 MHz, $CDCl_3$) δ (ppm): 179.3, 153.7, 139.9, 135.2, 128.6, 128.3, 127.3, 125.9, 124.9, 122.9, 122.0, 56.5, 52.2, 23.3.

HRMS (ESI) *m/z:* $[M+H]^+$ calculated for $C_{16}H_{17}N_2S$, 269.1107; found, 269.1104.

7.4.5 Basic hydrolysis of **4a** to amine **S3**

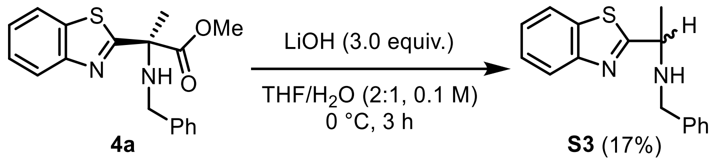

To a solution of methyl ester **4a** (0.10 g, 0.31 mmol, 1.0 equiv) in THF/H_2O (2:1; 3.0 mL, 0.10 M) at 0 °C was added LiOH·H_2O (0.039 g, 0.93 mmol, 3.0 equiv). The reaction mixture was stirred for 3 h. After completion (TLC), the mixture was concentrated on a rotary evaporator, diluted with water (10 mL), and acidified to pH 3 with 5 M HCl. The aqueous layer was extracted with EtOAc (3 × 15 mL), and the combined organic layers were washed with brine, dried over $MgSO_4$, and concentrated under reduced pressure. Purification of the crude residue by gradient column chromatography (SiO_2; hexane/EtOAc = 15:1 → 1:1) afforded **S3** (0.014 g, 0.05 mmol, 17%) as a colorless oil.

7.4.6 Control reaction with Weinreb amide **1a**

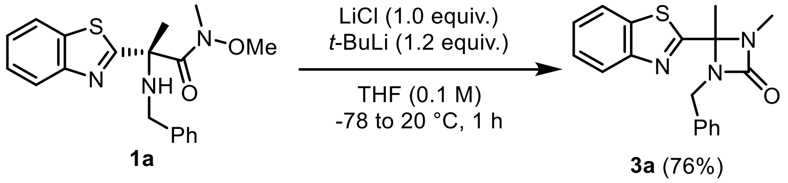

Amide **1a** (150 mg, 0.42 mmol, 1.0 equiv) and LiCl (18 mg, 0.42 mmol, 1.0 equiv) were dissolved in dry THF (4.2 mL, 0.10 M) in a flame-dried round-bottom flask under an argon atmosphere. The reaction mixture was cooled to –78 °C, and *t*-BuLi (1.7 M in pentane, 0.30 mL, 0.51 mmol, 1.2 equiv) was added dropwise. The mixture was stirred at –78 °C for 5 min and then warmed to 20 °C and stirred for 1 h. The reaction was quenched with sat. aq NH_4Cl and extracted with EtOAc (3 × 10 mL). The combined organic layers were washed with brine, dried over Na_2SO_4, and concentrated under reduced pressure. Purification of the crude residue by gradient column chromatography (SiO_2, hexane/EtOAc = 15:1 → 2:1) afforded 1,3-diazetidin-2-one **3a** (104 mg, 0.32 mmol, 76%) as a colorless solid.

*7.4.6.1 Reaction of **4a** with O-benzylhydroxylamine **5e***

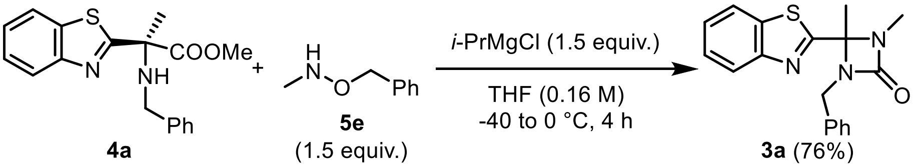

Following **METHOD B**, starting from amino ester **4a** (65.3 mg, 0.20 mmol, 1.0 equiv.) and hydroxylamine **5e** (41.2 mg, 0.30 mmol, 1.5 equiv.). Purification by column chromatography (SiO_2, hexane/EtOAc = 15:1 → 5:1) afforded 1,3-diazetidin-2-one **3a** (49 mg, 0.15 mmol, 76 % yield) as a colorless oil.

7.4.7 Reaction of **4a** with *N*-methyl-*O*-(methylsulfonyl)hydroxylamine **5n**

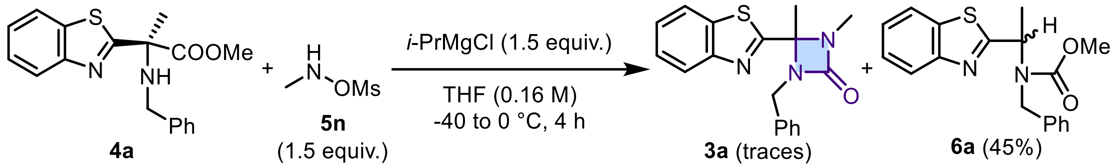

Following **METHOD B**, starting from amino ester **4a** (65.3 mg, 0.20 mmol, 1.0 equiv.) and hydroxylamine **5n** (37.5 mg, 0.30 mmol, 1.5 equiv.). Standard reaction work-up afforded carbamate **6a** (45 % NMR yield), starting amino ester **4a** (20 % NMR yield) and traces of 1,3-diazetidin-2-one **3a**.

7.4.8 Reaction of **4a** with *O*-(*tert*-butyl)-*N*-methylhydroxylamine hydrochloride **5o**

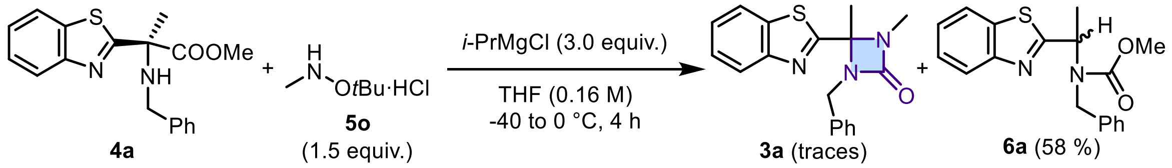

Following **METHOD A**, starting from amino ester **4a** (65.3 mg, 0.20 mmol, 1.0 equiv.) and hydroxylamine **5o** (41.9 mg, 0.30 mmol, 1.5 equiv.). Standard reaction work-up afforded carbamate **6a** (58 % NMR yield) and traces of 1,3-diazetidin-2-one **3a**.

8 References

(1) Murray, C. J. L.; Ikuta, K. S.; Sharara, F.; Swetschinski, L.; Robles Aguilar, G.; Gray, A.; Han, C.; Bisignano, C.; Rao, P.; Wool, E.; Johnson, S. C.; Browne, A. J.; Chipeta, M. G.; Fell, F.; Hackett, S.; Haines-Woodhouse, G.; Kashef Hamadani, B. H.; Kumaran, E. A. P.; McManigal, B.; Achalapong, S.; Agarwal, R.; Akech, S.; Albertson, S.; Amuasi, J.; Andrews, J.; Aravkin, A.; Ashley, E.; Babin, F.-X.; Bailey, F.; Baker, S.; Basnyat, B.; Bekker, A.; Bender, R.; Berkley, J. A.; Bethou, A.; Bielicki, J.; Boonkasidecha, S.; Bukosia, J.; Carvalheiro, C.; Castañeda-Orjuela, C.; Chansamouth, V.; Chaurasia, S.; Chiurchiù, S.; Chowdhury, F.; Clotaire Donatien, R.; Cook, A. J.; Cooper, B.; Cressey, T. R.; Criollo-Mora, E.; Cunningham, M.; Darboe, S.; Day, N. P. J.; De Luca, M.; Dokova, K.; Dramowski, A.; Dunachie, S. J.; Duong Bich, T.; Eckmanns, T.; Eibach, D.; Emami, A.; Feasey, N.; Fisher-Pearson, N.; Forrest, K.; Garcia, C.; Garrett, D.; Gastmeier, P.; Giref, A. Z.; Greer, R. C.; Gupta, V.; Haller, S.; Haselbeck, A.; Hay, S. I.; Holm, M.; Hopkins, S.; Hsia, Y.; Iregbu, K. C.; Jacobs, J.; Jarovsky, D.; Javanmardi, F.; Jenney, A. W. J.; Khorana, M.; Khusuwan, S.; Kissoon, N.; Kobeissi, E.; Kostyanev, T.; Krapp, F.; Krumkamp, R.; Kumar, A.; Kyu, H. H.; Lim, C.; Lim, K.; Limmathurotsakul, D.; Loftus, M. J.; Lunn, M.; Ma, J.; Manoharan, A.; Marks, F.; May, J.; Mayxay, M.; Mturi, N.; Munera-Huertas, T.; Musicha, P.; Musila, L. A.; Mussi-Pinhata, M. M.; Naidu, R. N.; Nakamura, T.; Nanavati, R.; Nangia, S.; Newton, P.; Ngoun, C.; Novotney, A.; Nwakanma, D.; Obiero, C. W.; Ochoa, T. J.; Olivas-Martinez, A.; Olliaro, P.; Ooko, E.; Ortiz-Brizuela, E.; Ounchanum, P.; Pak, G. D.; Paredes, J. L.; Peleg, A. Y.; Perrone, C.; Phe, T.; Phommasone, K.; Plakkal, N.; Ponce-de-Leon, A.; Raad, M.; Ramdin, T.; Rattanavong, S.; Riddell, A.; Roberts, T.; Robotham, J. V.; Roca, A.; Rosenthal, V. D.; Rudd, K. E.; Russell, N.; Sader, H. S.; Saengchan, W.; Schnall, J.; Scott, J. A. G.; Seekaew, S.; Sharland, M.; Shivamallappa, M.; Sifuentes-Osornio, J.; Simpson, A. J.; Steenkeste, N.; Stewardson, A. J.; Stoeva, T.; Tasak, N.; Thaiprakong, A.; Thwaites, G.; Tigoi, C.; Turner, C.; Turner, P.; Van Doorn, H. R.; Velaphi, S.; Vongpradith, A.; Vongsouvath, M.; Vu, H.; Walsh, T.; Walson, J. L.; Waner, S.; Wangrangsimakul, T.; Wannapinij, P.; Wozniak, T.; Young Sharma, T. E. M. W.; Yu, K. C.; Zheng, P.; Sartorius, B.; Lopez, A. D.; Stergachis, A.; Moore, C.; Dolecek, C.; Naghavi, M. Global Burden of Bacterial Antimicrobial Resistance in 2019: A Systematic Analysis. *The Lancet* **2022**, *399* (10325), 629–655. https://doi.org/10.1016/S0140-6736(21)02724-0.

(2) Fisher, J. F.; Meroueh, S. O.; Mobashery, S. Bacterial Resistance to β-Lactam Antibiotics: Compelling Opportunism, Compelling Opportunity. *Chem. Rev.* **2005**, *105* (2), 395–424. https://doi.org/10.1021/cr030102i.

(3) Pemberton, O. A.; Noor, R. E.; Kumar M. V., V.; Sanishvili, R.; Kemp, M. T.; Kearns, F. L.; Woodcock, H. L.; Gelis, I.; Chen, Y. Mechanism of Proton Transfer in Class A β-Lactamase Catalysis and Inhibition by Avibactam. *Proc. Natl. Acad. Sci.* **2020**, *117* (11), 5818–5825. https://doi.org/10.1073/pnas.1922203117.

(4) Arer, V.; Kar, D. Biochemical Exploration of β-Lactamase Inhibitors. *Front. Genet.* **2023**, *13* (13), 1060736. https://doi.org/10.3389/fgene.2022.1060736.

(5) Chandrakala, P. S.; Katz, A. K.; Carrell, H. L.; Sailaja, P. R.; Podile, A. R.; Nangia, A.; Desiraju, G. R. Synthesis, X-Ray Crystal Structures and Biological Evaluation of Some Mono- and Bi-Cyclic 1,3-Diazetidin-2-Ones: Non-Natural β-Lactam Analogues. *J. Chem. Soc. Perkin 1* **1998**, *0* (16), 2597–2608. https://doi.org/10.1039/A802438C.

(6) Coll, M.; Frau, J.; Donoso, J.; Muñoz, F. PM3 Study of Reactivity of Non-Classical β-Lactam Structures. *J. Mol. Struct. THEOCHEM* **1999**, *493* (1–3), 287–299. https://doi.org/10.1016/S0166-1280(99)00250-X.

(7) Kristek, J.; Pospíšil, J. 1,3-Diazetidin-2-Ones: Synthetic Approaches and Therapeutic Promise within the Aza-β-Lactam Family. *Eur. J. Org. Chem.* **2025**, *28* (43), e202500747. https://doi.org/10.1002/ejoc.202500747.
(8) Coll, M.; Frau, J.; Vilanova, B.; Donoso, J.; Muñoz, F. Electrostatic and Structural Similarity of Classical and Non-Classical Lactam Compounds. *J. Comput. Aided Mol. Des.* **2001**, *15* (9), 819–833. https://doi.org/10.1023/A:1013123702720.
(9) Cognetta, A. B.; Niphakis, M. J.; Lee, H. C.; Martini, M. L.; Hulce, J. J.; Cravatt, B. F. Selective N-Hydroxyhydantoin Carbamate Inhibitors of Mammalian Serine Hydrolases. *Chem. Biol.* **2015**, *22* (7), 928–937. https://doi.org/10.1016/j.chembiol.2015.05.018.
(10) Taylor, J. S.; Cohrs, M. P. DNA, Light, and Dewar Pyrimidinones: The Structure and Biological Significance of TpT3. *J. Am. Chem. Soc.* **1987**, *109* (9), 2834–2835. https://doi.org/10.1021/JA00243A052/ASSET/JA00243A052.FP.PNG_V03.
(11) Nishio, T.; Kato, A.; Kashima, C.; Omote, Y. Photochemical Electrocyclization of 1,4,6-Trisubstituted Pyrimidin-2-Ones to 2-Oxo-1,3-Diazabicyclo[2.2.0]Hex-5-Enes. *J. Chem. Soc. Perkin 1* **1980**, 607. https://doi.org/10.1039/p19800000607.
(12) Cotter, E.; Pultar, F.; Riniker, S.; Altmann, K. Experimental and Theoretical Studies on the Reactions of Aliphatic Imines with Isocyanates. *Chem. – Eur. J.* **2024**, *30* (14), e202304272. https://doi.org/10.1002/chem.202304272.
(13) Bla, B. No Title. *ChemRivX*.
(14) Schöllkopf, U.; Lau, H.; Scheunemann, K.; Blume, E.; Madawinata, K. Umsetzungen A-metallierter Isocyanide Mit Einigen 1,3-Dipolen. *Liebigs Ann. Chem.* **1980**, *1980* (4), 600–610. https://doi.org/10.1002/jlac.198019800412.
(15) Fritz, J. A.; Wolfe, J. P. Stereoselective Synthesis of Imidazolidin-2-Ones via Pd-Catalyzed Alkene Carboamination. Scope and Limitations. *Tetrahedron* **2008**, *64* (29), 6838–6852. https://doi.org/10.1016/j.tet.2008.04.015.
(16) Labeeuw, O.; Phansavath, P.; Genêt, J.-P. Synthesis of Modified Weinreb Amides: N-Tert-Butoxy-N-Methylamides as Effective Acylating Agents. *Tetrahedron Lett.* **2004**, *45* (38), 7107–7110. https://doi.org/10.1016/j.tetlet.2004.07.106.
(17) Behr, J.-B.; Chevrier, C.; Defoin, A.; Tarnus, C.; Streith, J. Asymmetric Synthesis of Potent Glycosidase and Very Potent α-Mannosidase Inhibitors: 4-Amino-4-Deoxy-l-Erythrose and 4-Amino-4,5-Dideoxy-l-Ribose. *Tetrahedron* **2003**, *59* (4), 543–553. https://doi.org/10.1016/S0040-4020(02)01512-0.
(18) Javorskis, T.; Sriubaitė, S.; Bagdžiūnas, G.; Orentas, E. N-Protected 1,2-Oxazetidines as a Source of Electrophilic Oxygen: Straightforward Access to Benzomorpholines and Related Heterocycles by Using a Reactive Tether. *Chem. – Eur. J.* **2015**, *21* (25), 9157–9164. https://doi.org/10.1002/chem.201500731.
(19) Rohrbacher, F.; Baldauf, S.; Wucherpfennig, T.; Bode, J. Product Selectivity in KAHA Ligations: Ester vs. Amide Formation with Cyclic Hydroxylamines. *Synlett* **2017**, *28* (15), 1929–1933. https://doi.org/10.1055/s-0036-1588480.
(20) Teze, D.; Dion, M.; Daligault, F.; Tran, V.; André-Miral, C.; Tellier, C. Alkoxyamino Glycoside Acceptors for the Regioselective Synthesis of Oligosaccharides Using Glycosynthases and Transglycosidases. *Bioorg. Med. Chem. Lett.* **2013**, *23* (2), 448–451. https://doi.org/10.1016/j.bmcl.2012.11.065.
(21) Maillard, L. T.; Benohoud, M.; Durand, P.; Badet, B. A New Supported Reagent for the Parallel Synthesis of Primary and Secondary *O* -Alkyl Hydroxylamines through a Base-Catalyzed Mitsunobu Reaction. *J. Org. Chem.* **2005**, *70* (16), 6303–6312. https://doi.org/10.1021/jo050722e.

(22) Patel, I.; Smith, N. A.; Tyler, S. N. G. An Improved Process for the Synthesis and Isolation of (*S*)- *N* -(1-Phenylethyl)Hydroxylamine. *Org. Process Res. Dev.* **2009**, *13* (1), 49–53. https://doi.org/10.1021/op800230f.

(23) Da Costa, M. R. G.; Curto, M. J. M.; Davies, S. G.; Sanders, J.; Teixeira, F. C. Synthesis of (R)-{Î·6-[O-Methyl-N-(Î±-Methylbenzyl)Hydroxyamino]Benzene} Chromium Tricarbonyl via Nucleophilic Aromatic Substitution of (Î·6-Fluorobenzene) Chromium Tricarbonyl. *J. Chem. Soc. Perkin 1* **2001**, No. 21, 2850–2855. https://doi.org/10.1039/b107237b.

(24) Li, B.; Chen, J.; Liu, D.; Gridnev, I. D.; Zhang, W. Nickel-Catalysed Asymmetric Hydrogenation of Oximes. *Nat. Chem.* **2022**, *14* (8), 920–927. https://doi.org/10.1038/s41557-022-00971-8.

(25) Gantt, R. W.; Goff, R. D.; Williams, G. J.; Thorson, J. S. Probing the Aglycon Promiscuity of an Engineered Glycosyltransferase. *Angew. Chem. Int. Ed.* **2008**, *47* (46), 8889–8892. https://doi.org/10.1002/anie.200803508.

(26) Lee, J.; Ban, J. W.; Kim, J.; Yang, S.; Lee, G.; Dhorma, L. P.; Kim, M.; Ha, M. W.; Hong, S.; Park, H. Asymmetric Phase-Transfer Catalytic Aza-Michael Addition to Cyclic Enone: Highly Enantioselective and Diastereoselective Synthesis of Cyclic 1,3-Aminoalcohols. *Org. Lett.* **2022**, *24* (8), 1647–1651. https://doi.org/10.1021/acs.orglett.2c00192.

(27) Miyabe, H.; Asada, R.; Takemoto, Y. Lewis Acid-Mediated Radical Cyclization: Stereocontrol in Cascade Radical Addition–Cyclization–Trapping Reactions. *Org. Biomol. Chem.* **2012**, *10* (17), 3519. https://doi.org/10.1039/c2ob25073j.

(28) Kon, Y.; Nakashima, T.; Makino, Y.; Nagashima, H.; Onozawa, S.; Kobayashi, S.; Sato, K. Continuous Synthesis of Epoxides from Alkenes by Hydrogen Peroxide with Titanium Silicalite-1 Catalyst Using Flow Reactors. *Adv. Synth. Catal.* **2023**, *365* (19), 3227–3233. https://doi.org/10.1002/adsc.202300181.

(29) Wang, T.; Hoffmann, M.; Dreuw, A.; Hasagić, E.; Hu, C.; Stein, P. M.; Witzel, S.; Shi, H.; Yang, Y.; Rudolph, M.; Stuck, F.; Rominger, F.; Kerscher, M.; Comba, P.; Hashmi, A. S. K. A Metal-Free Direct Arene C−H Amination. *Adv. Synth. Catal.* **2021**, *363* (11), 2783–2795. https://doi.org/10.1002/adsc.202100236.

(30) Ortega, N.; Richter, C.; Glorius, F. N-Formylation of Amines by Methanol Activation. *Org. Lett.* **2013**, *15* (7), 1776–1779. https://doi.org/10.1021/ol400639m.

9 Copy of ^{1}H, ^{13}C{^{1}H}NMR, and ^{19}F NMR Spectra

Copy of ^{1}H NMR Spectrum (400 MHz, $CDCl_3$) of **4a**

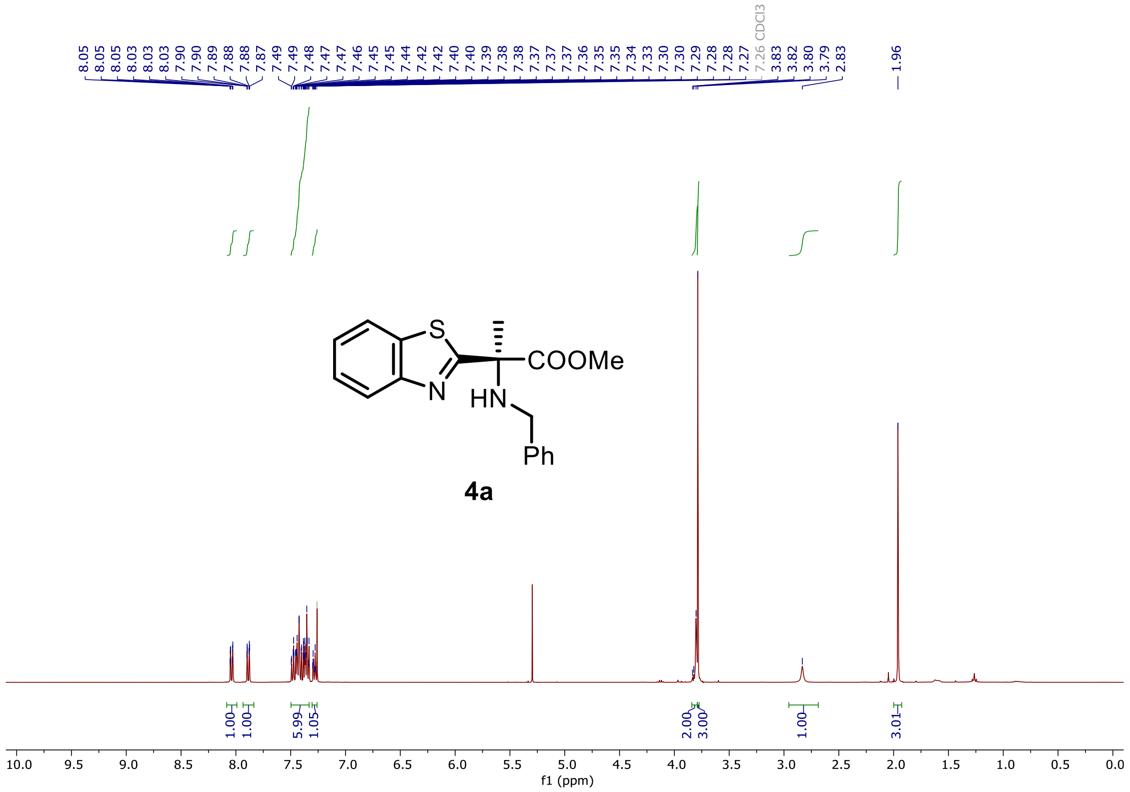

Copy of ^{13}C {^{1}H} NMR Spectrum (101 MHz, $CDCl_3$) of **4a**

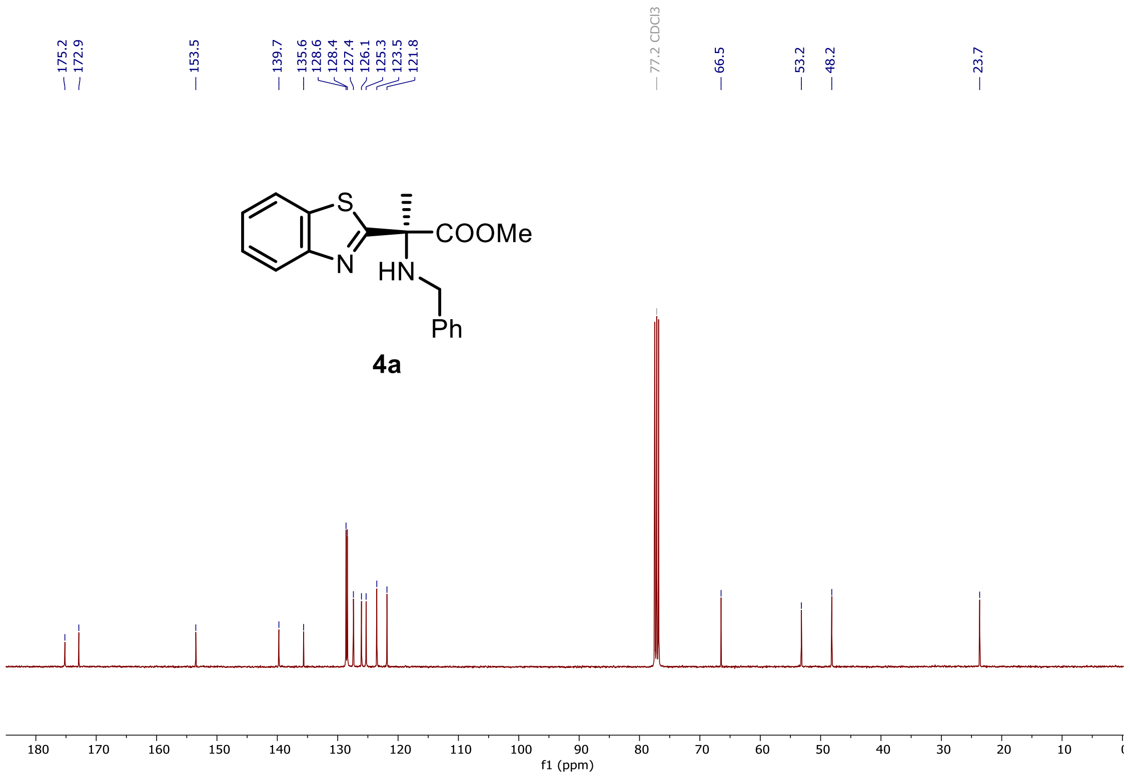

Copy of ^{1}H NMR Spectrum (400 MHz, $CDCl_3$) of **4b**

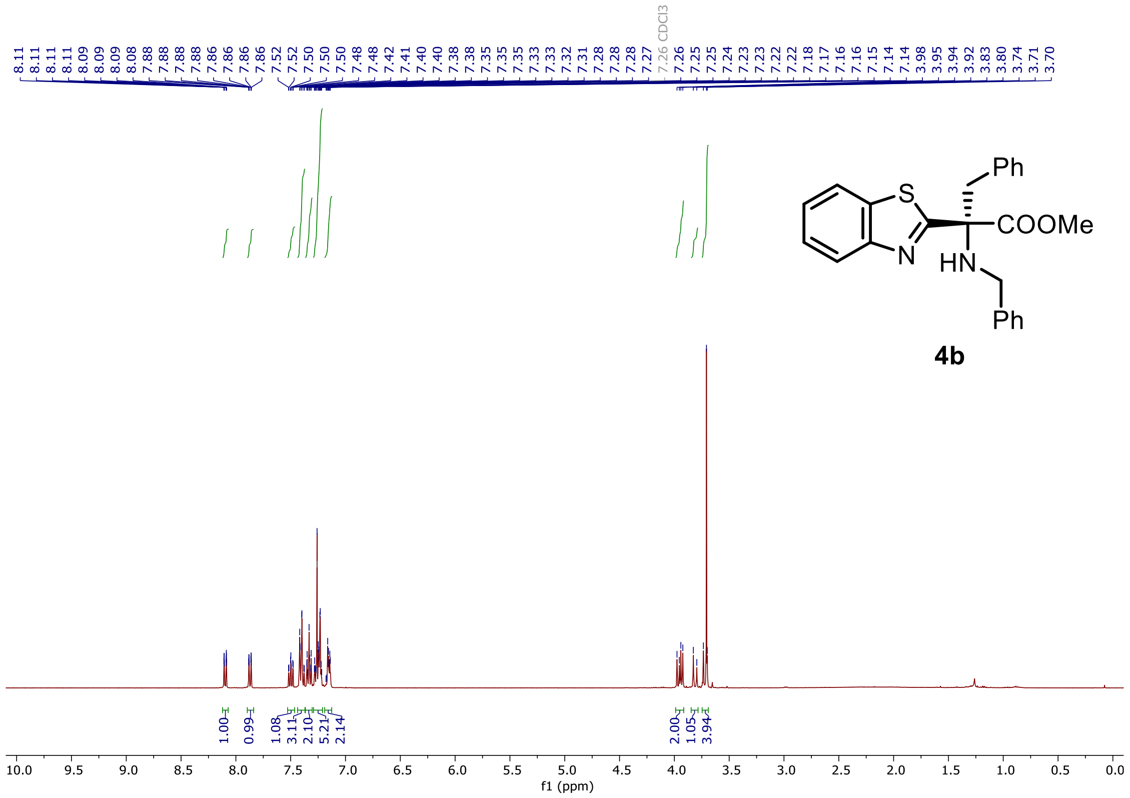

Copy of ^{13}C {^{1}H} NMR Spectrum (101 MHz, $CDCl_3$) of **4b**

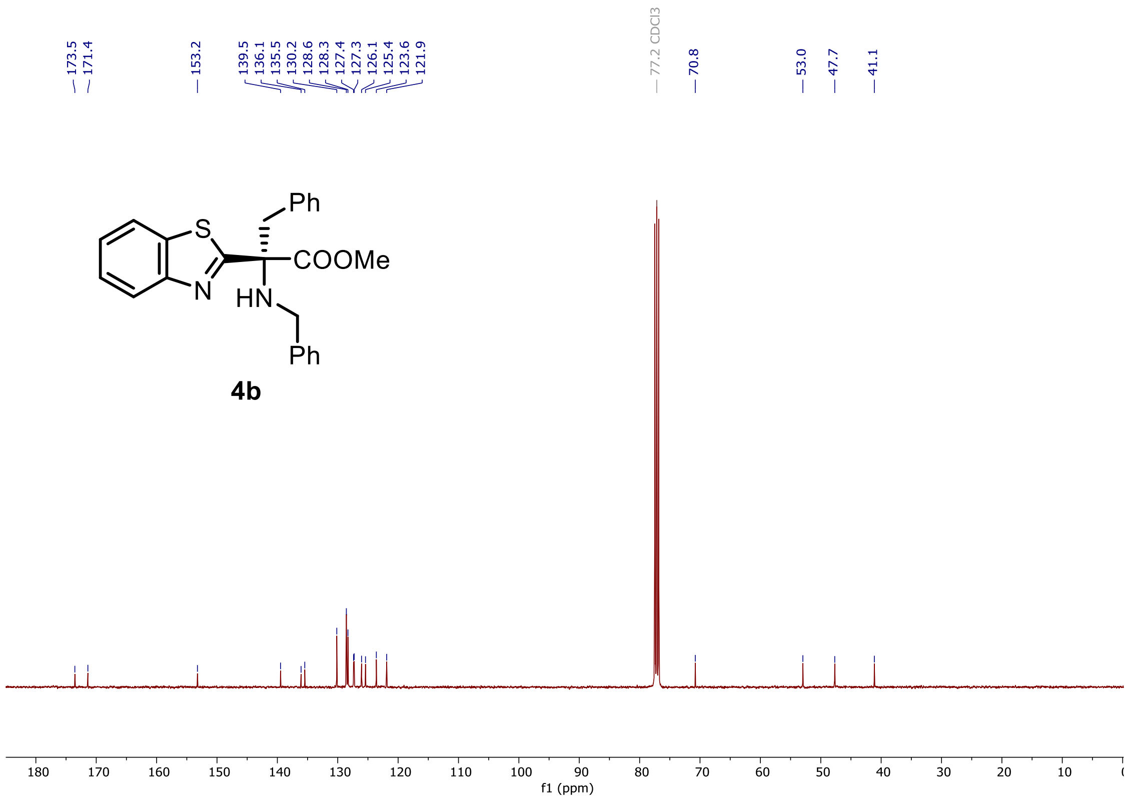

Copy of ^{1}H NMR Spectrum (500 MHz, $CDCl_3$) of **4c**

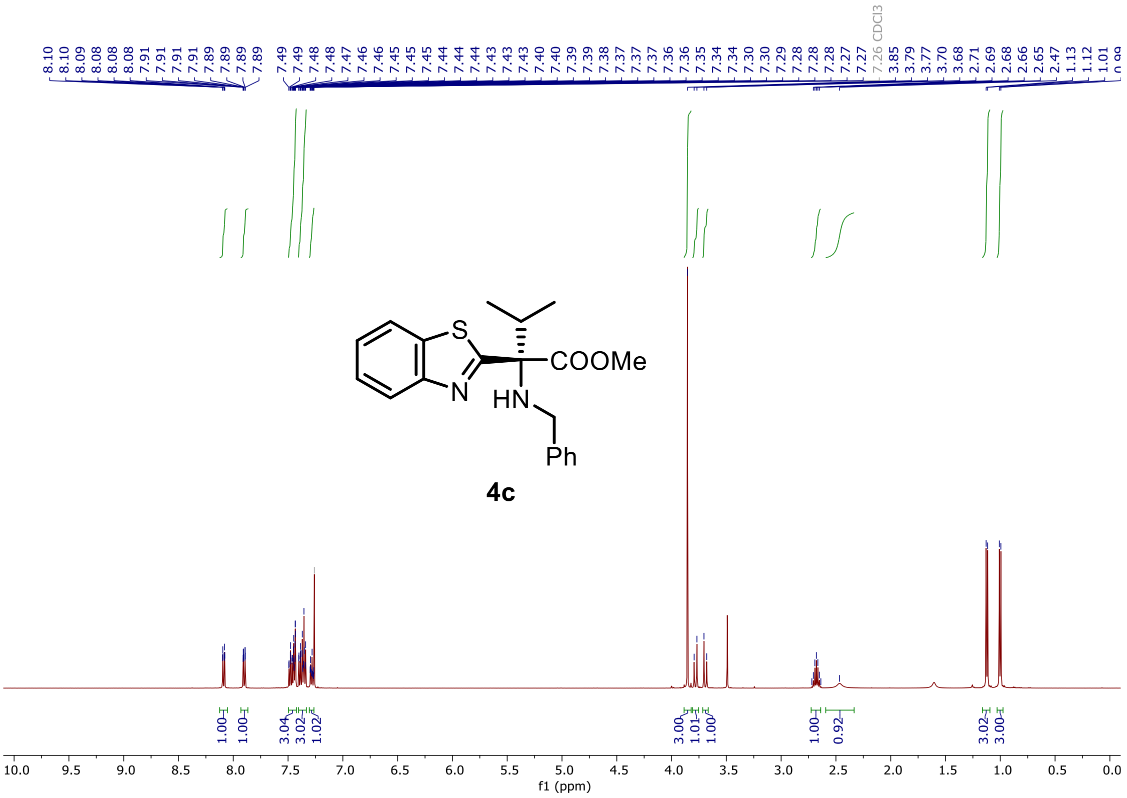

Copy of ^{13}C {^{1}H} NMR Spectrum (126 MHz, $CDCl_3$) of **4c**

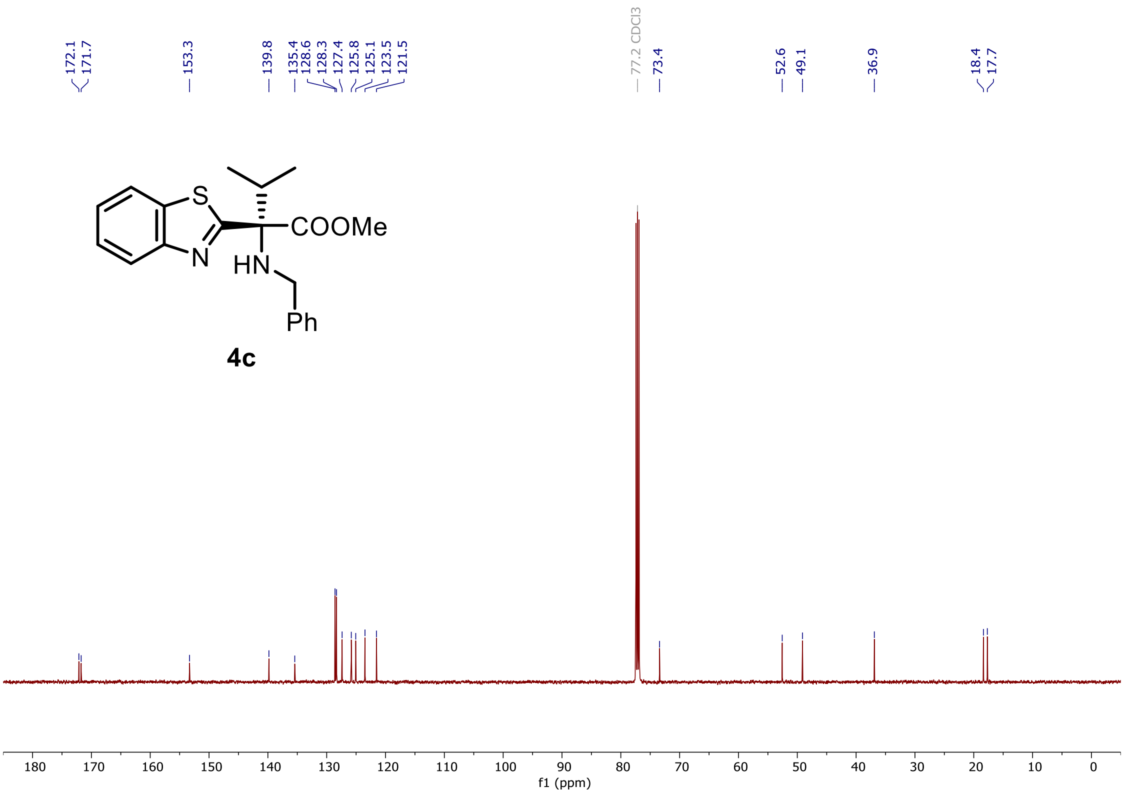

Copy of ^{1}H NMR Spectrum (500 MHz, $CDCl_3$) of **4d**

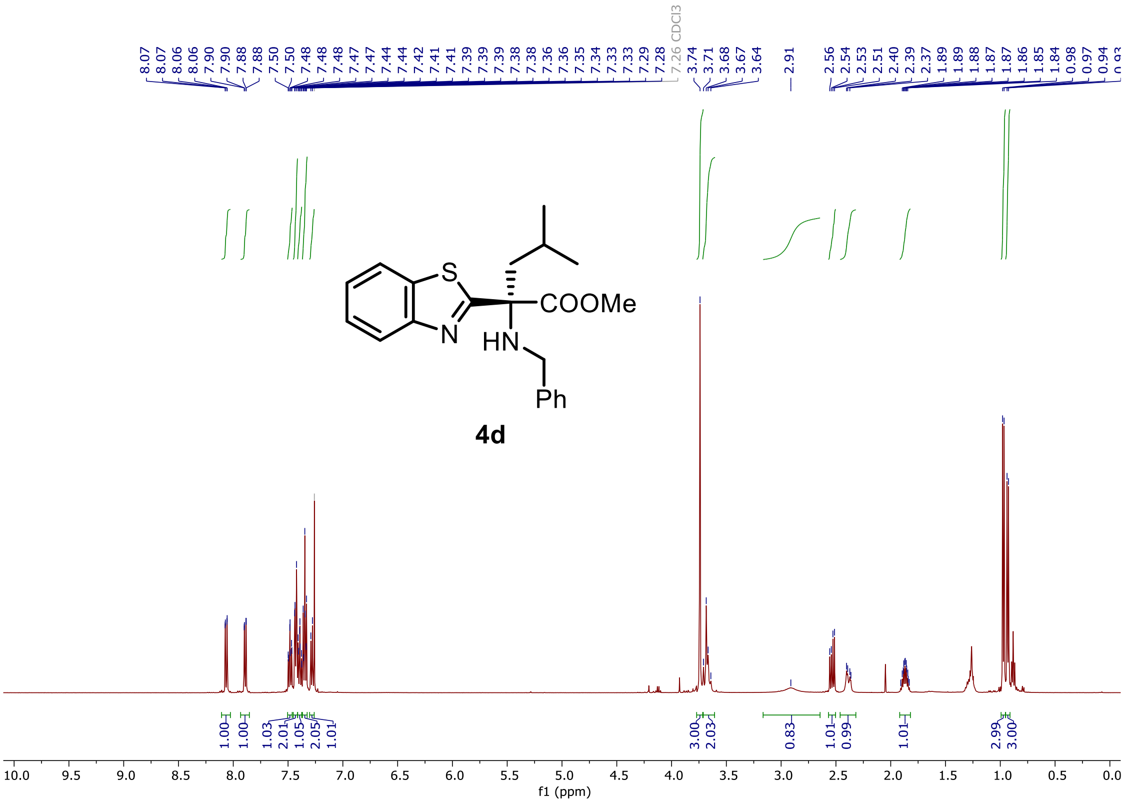

Copy of ^{13}C {^{1}H} NMR Spectrum (126 MHz, $CDCl_3$) of **4d**

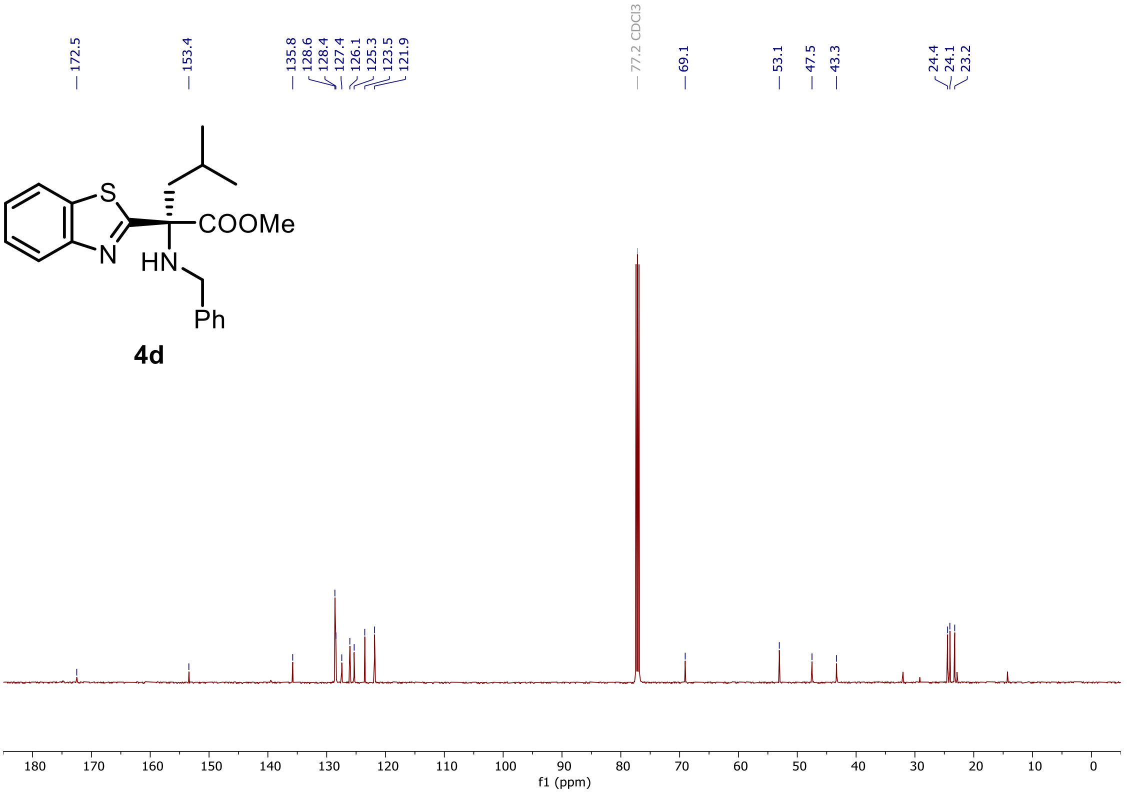

Copy of ^{1}H NMR Spectrum (500 MHz, $CDCl_3$) of **4e**

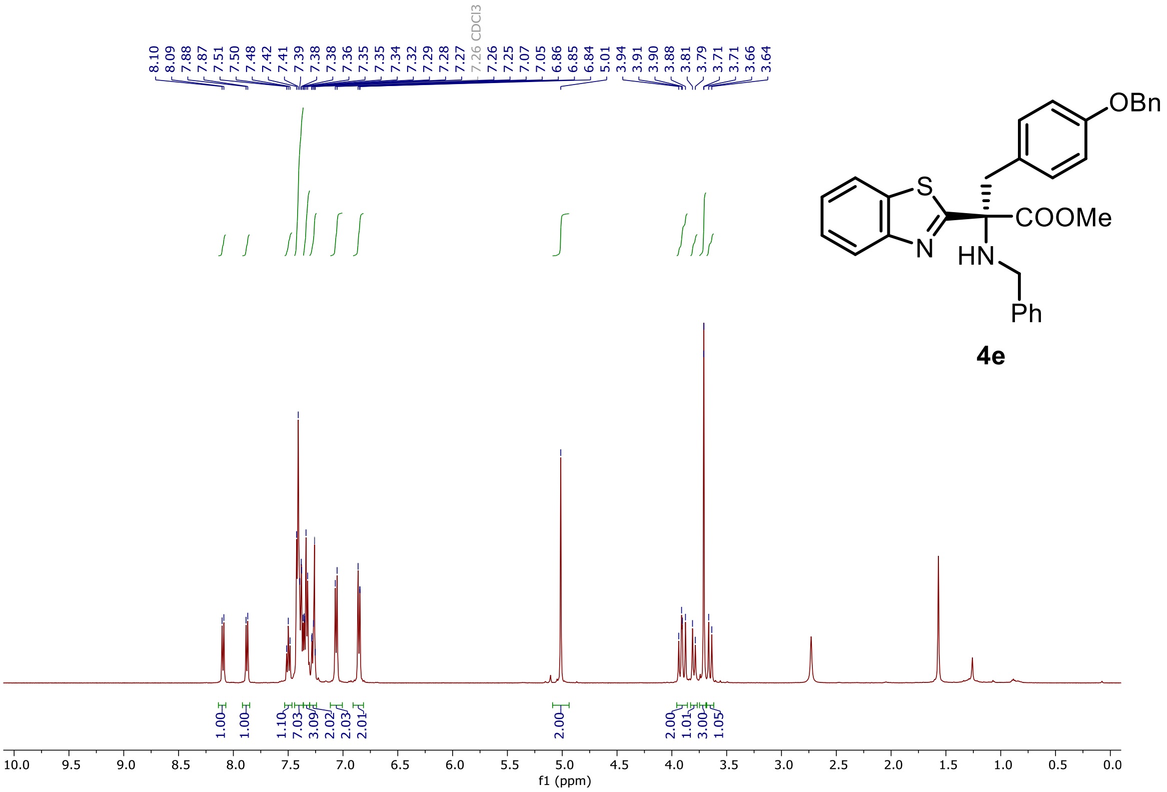

Copy of ^{13}C {^{1}H} NMR Spectrum (126 MHz, $CDCl_3$) of **4e**

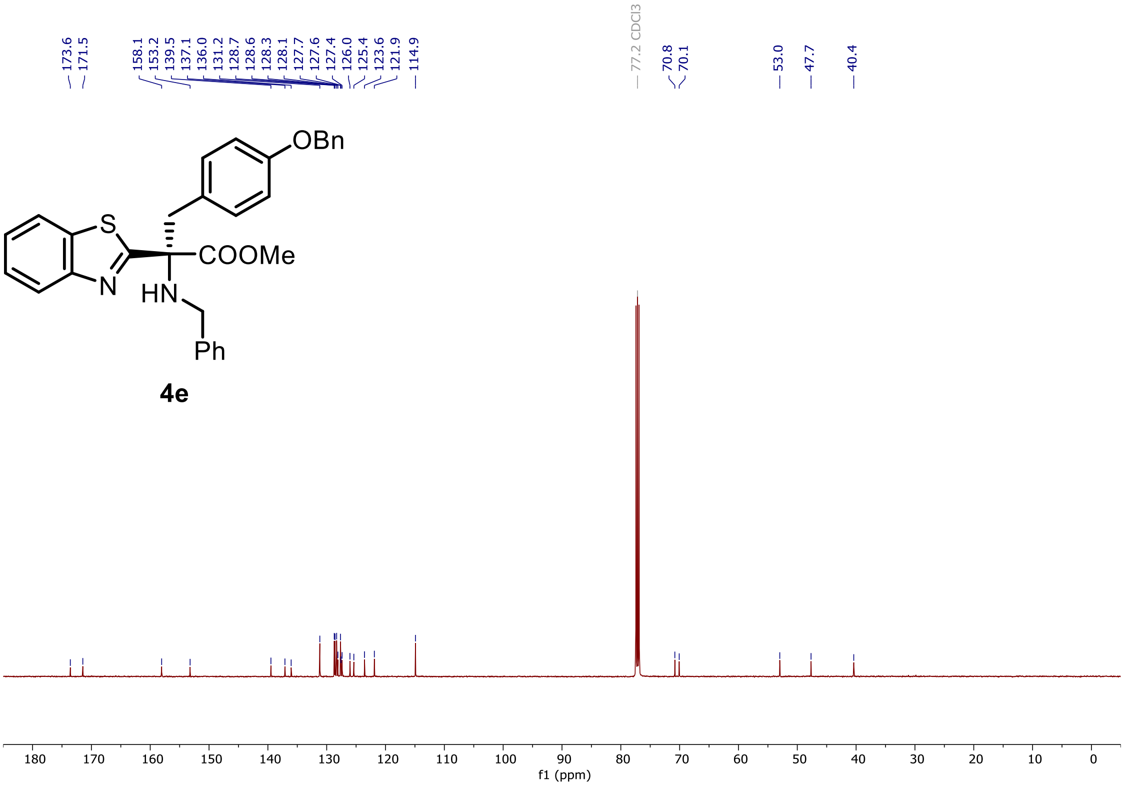

Copy of ^{1}H NMR Spectrum (500 MHz, $CDCl_3$) of **4f**

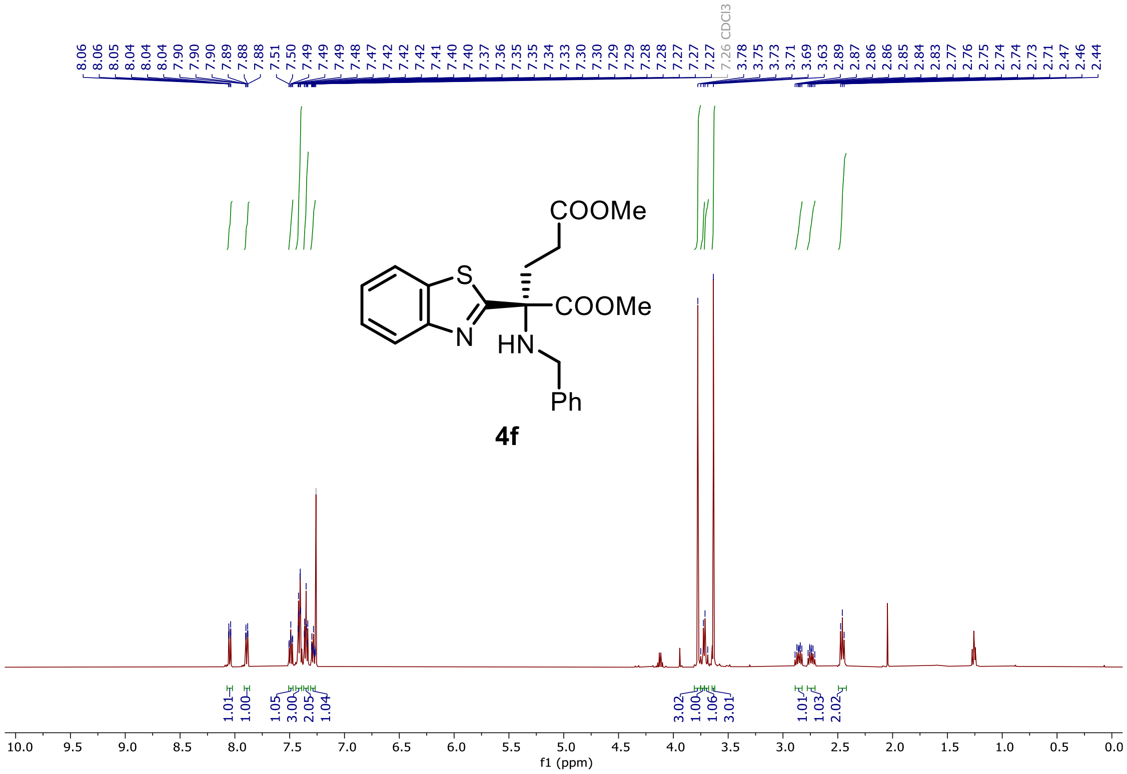

Copy of ^{13}C {^{1}H} NMR Spectrum (126 MHz, $CDCl_3$) of **4f**

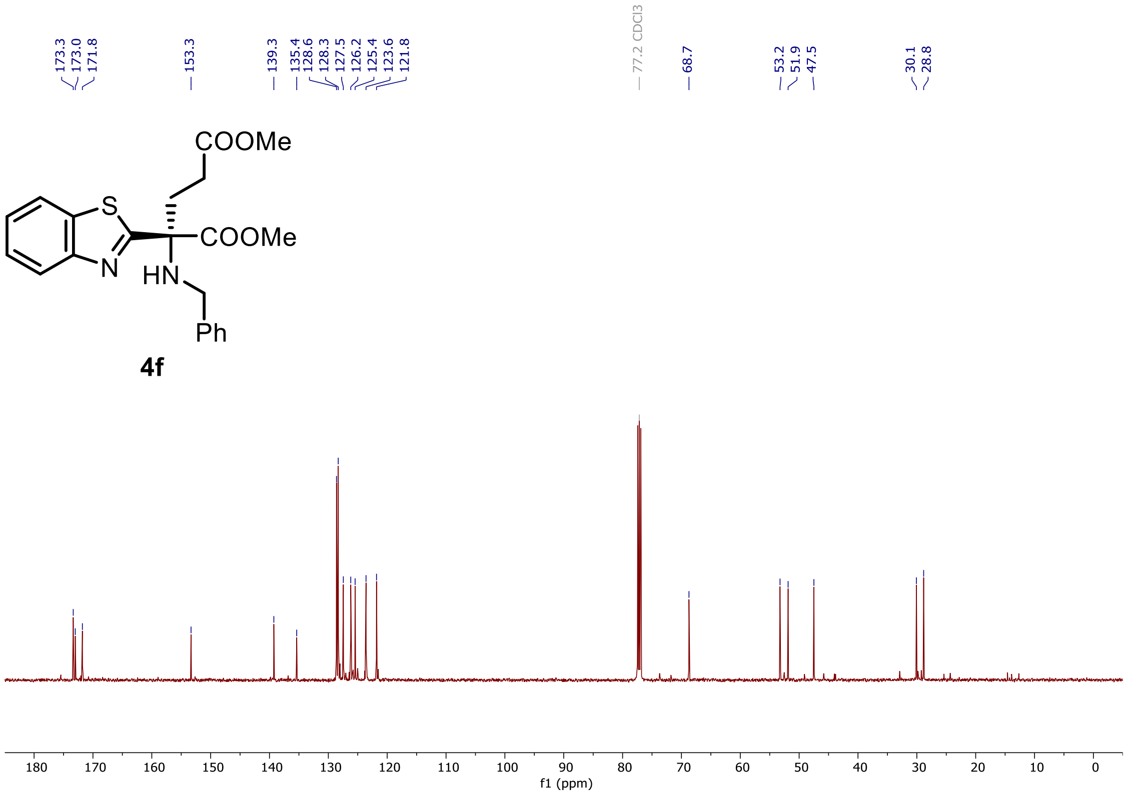

Copy of ^{1}H NMR Spectrum (400 MHz, $CDCl_3$) of **4g**

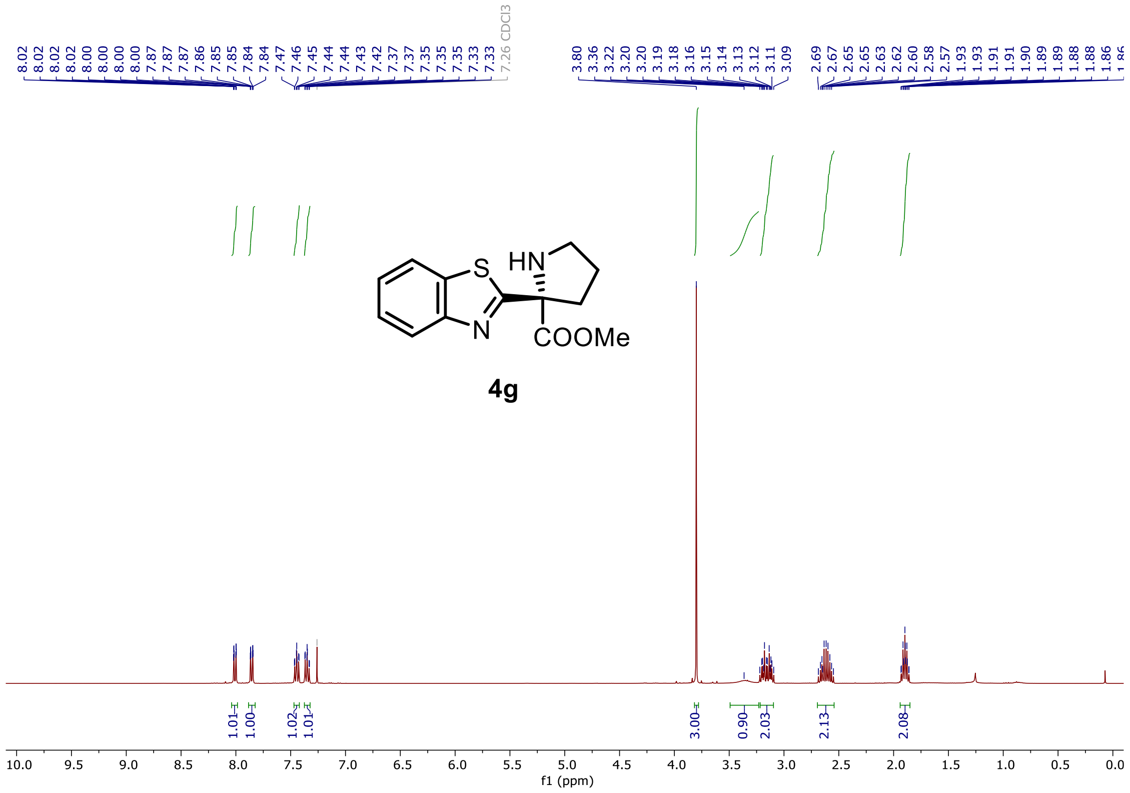

Copy of ^{13}C {^{1}H} NMR Spectrum (101 MHz, $CDCl_3$) of **4g**

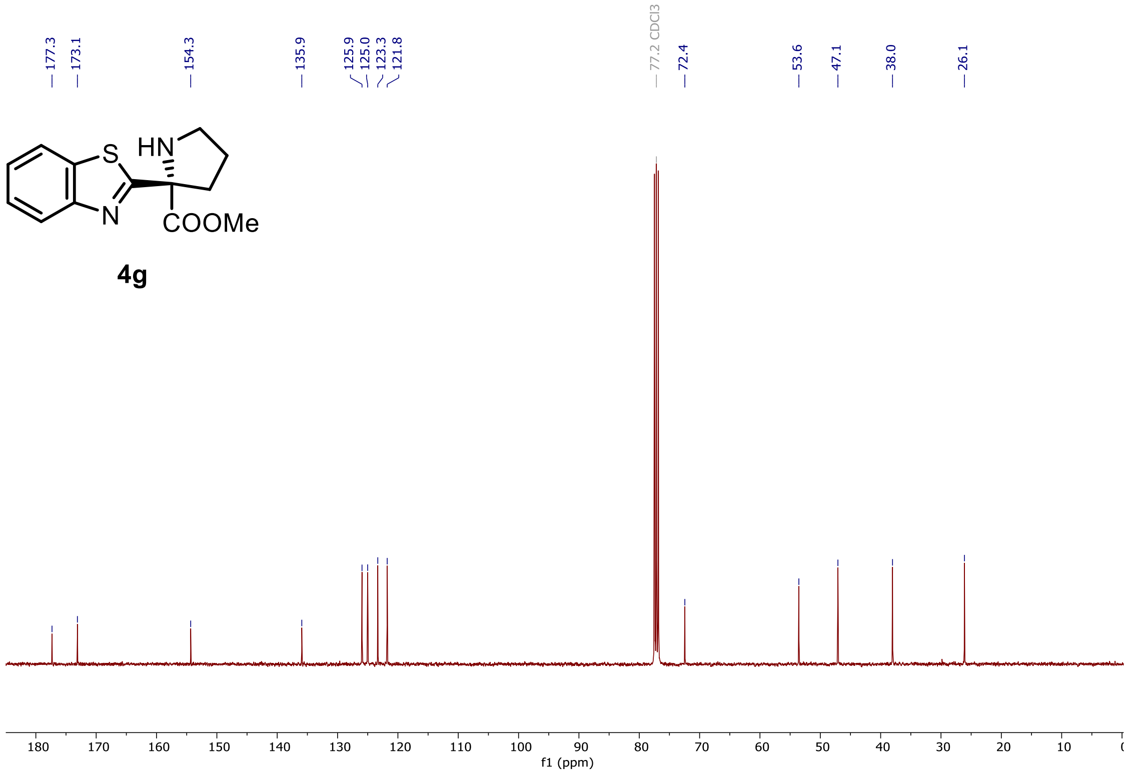

Copy of ^{1}H NMR Spectrum (500 MHz, $CDCl_3$) of **4h**

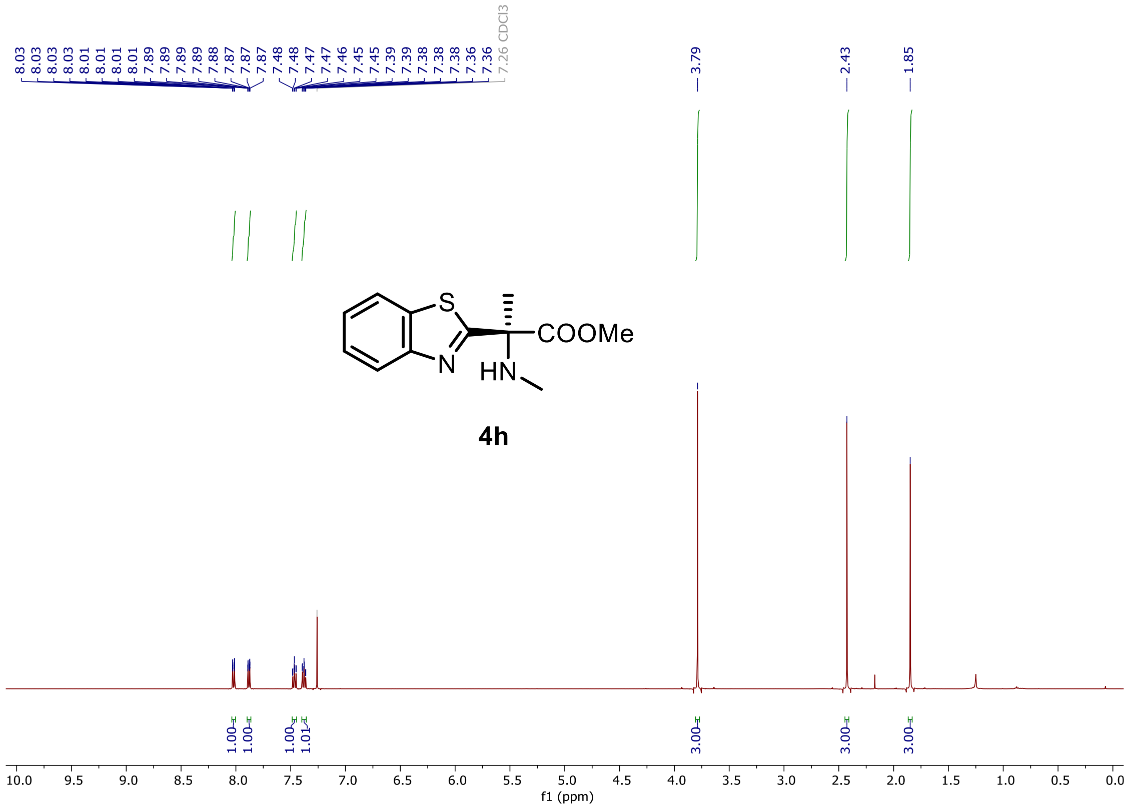

Copy of ^{13}C {^{1}H} NMR Spectrum (126 MHz, $CDCl_3$) of **4h**

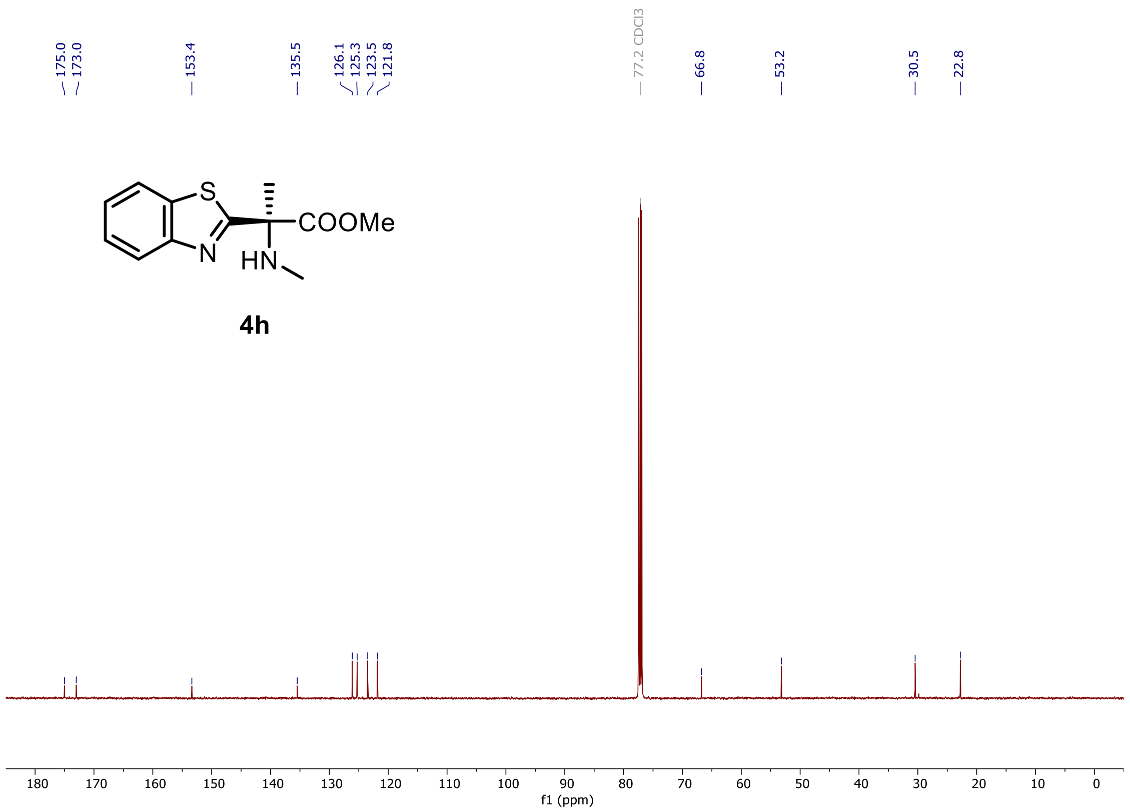

Copy of ^{1}H NMR Spectrum (500 MHz, $CDCl_3$) of **4i**

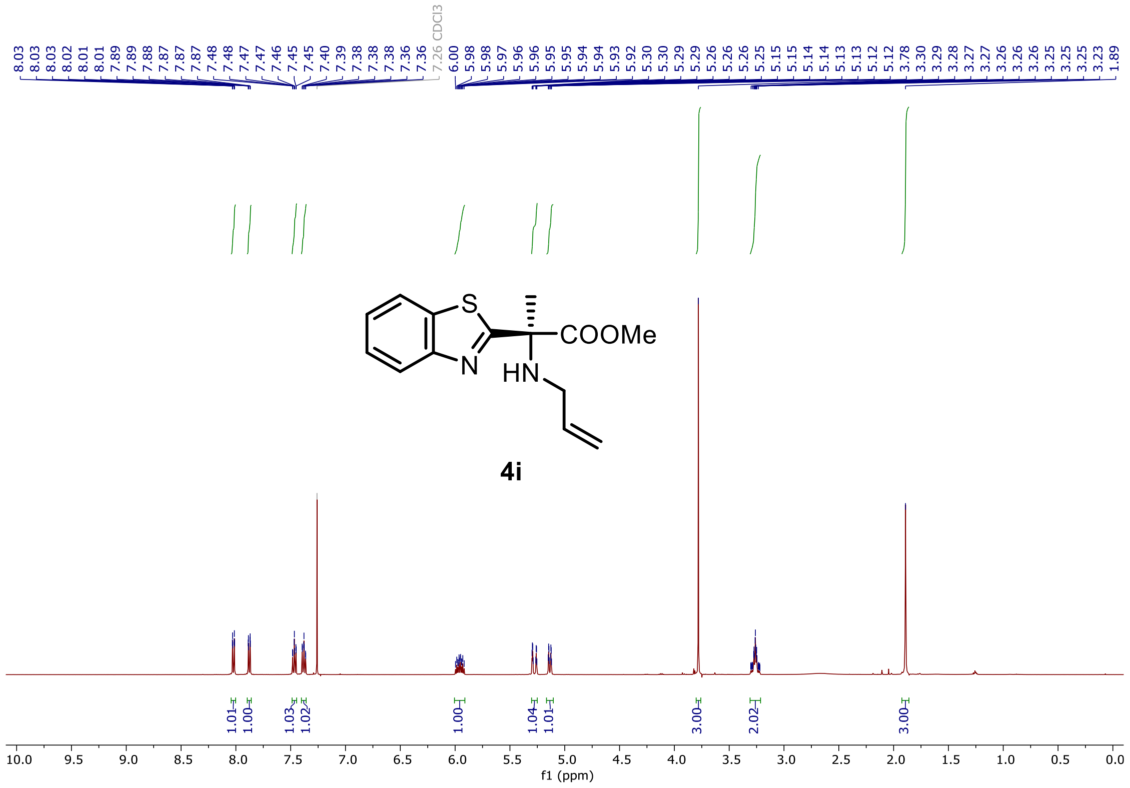

Copy of ^{13}C {^{1}H} NMR Spectrum (126 MHz, $CDCl_3$) of **4i**

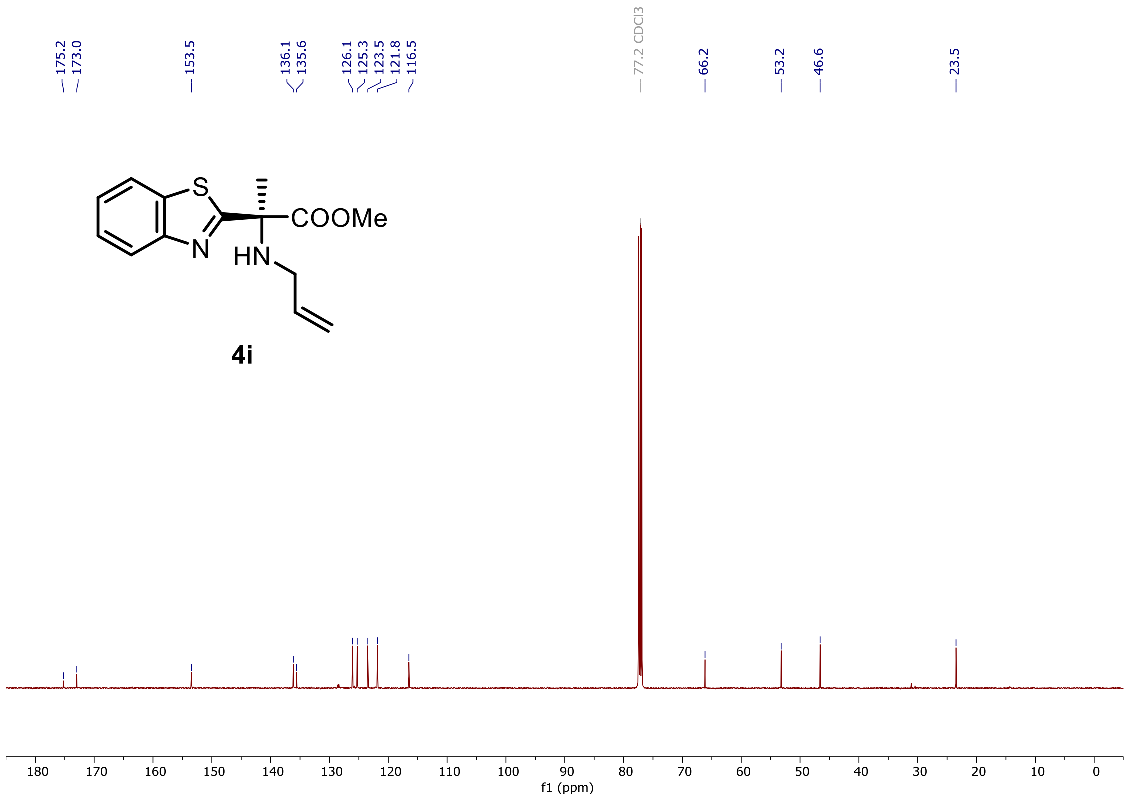

Copy of ^{1}H NMR Spectrum (500 MHz, $CDCl_3$) of **4j**

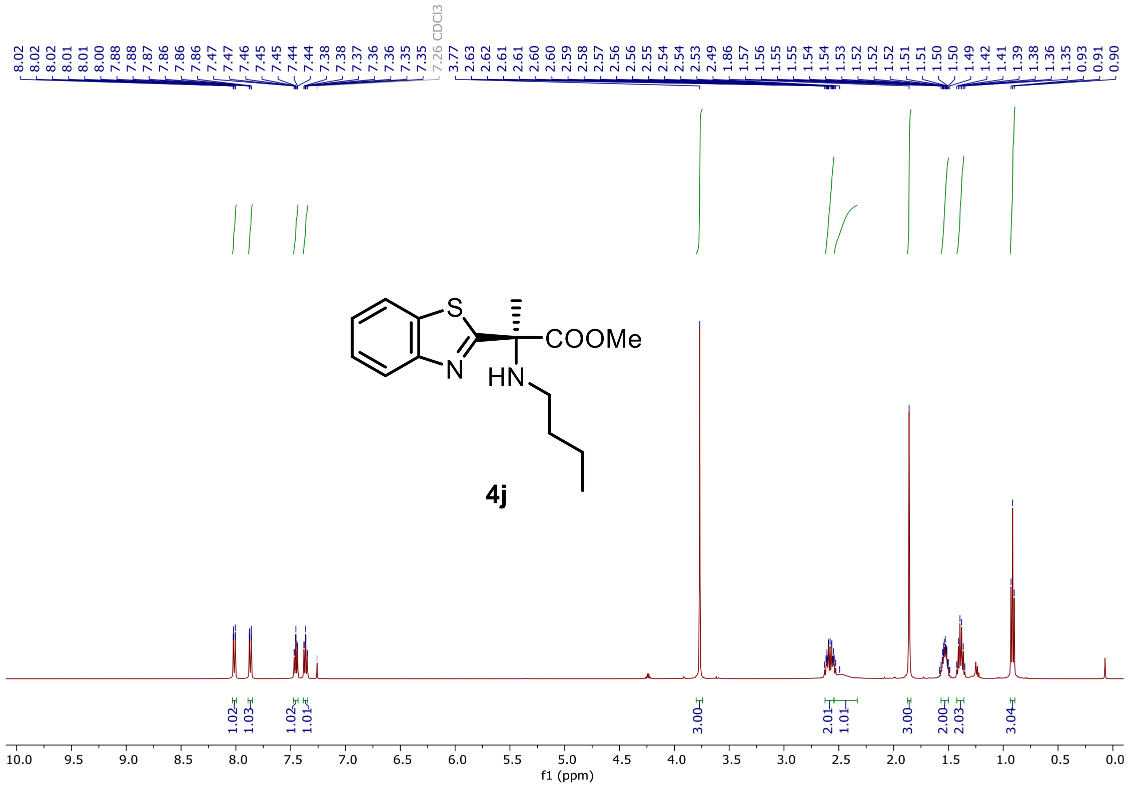

Copy of ^{13}C {^{1}H} NMR Spectrum (126 MHz, $CDCl_3$) of **4j**

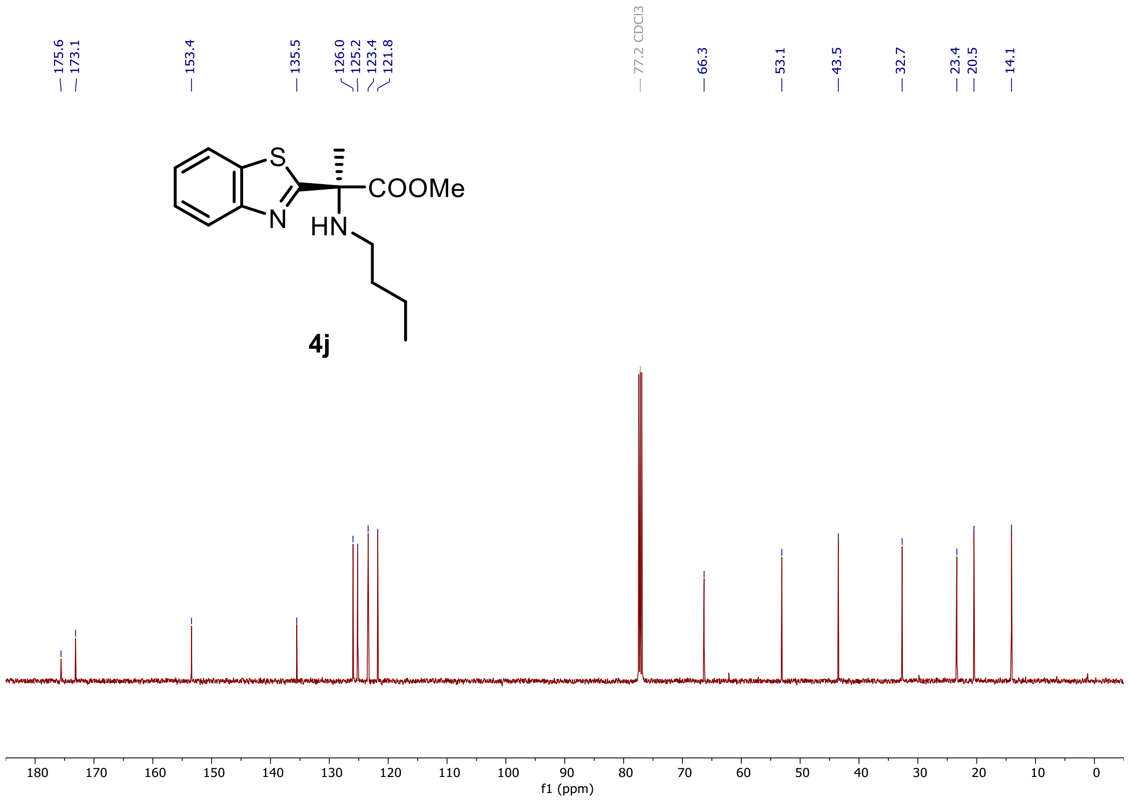

Copy of ^{1}H NMR Spectrum (500 MHz, $CDCl_3$) of **4k**

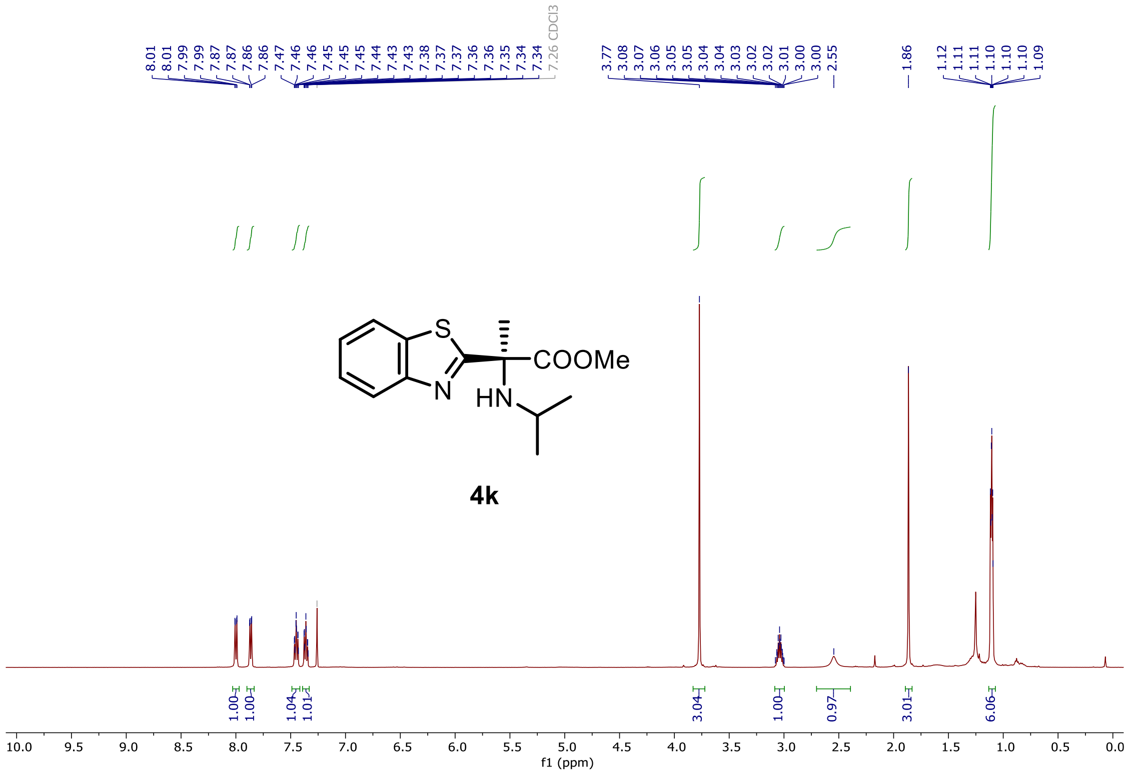

Copy of ^{13}C {^{1}H} NMR Spectrum (126 MHz, $CDCl_3$) of **4k**

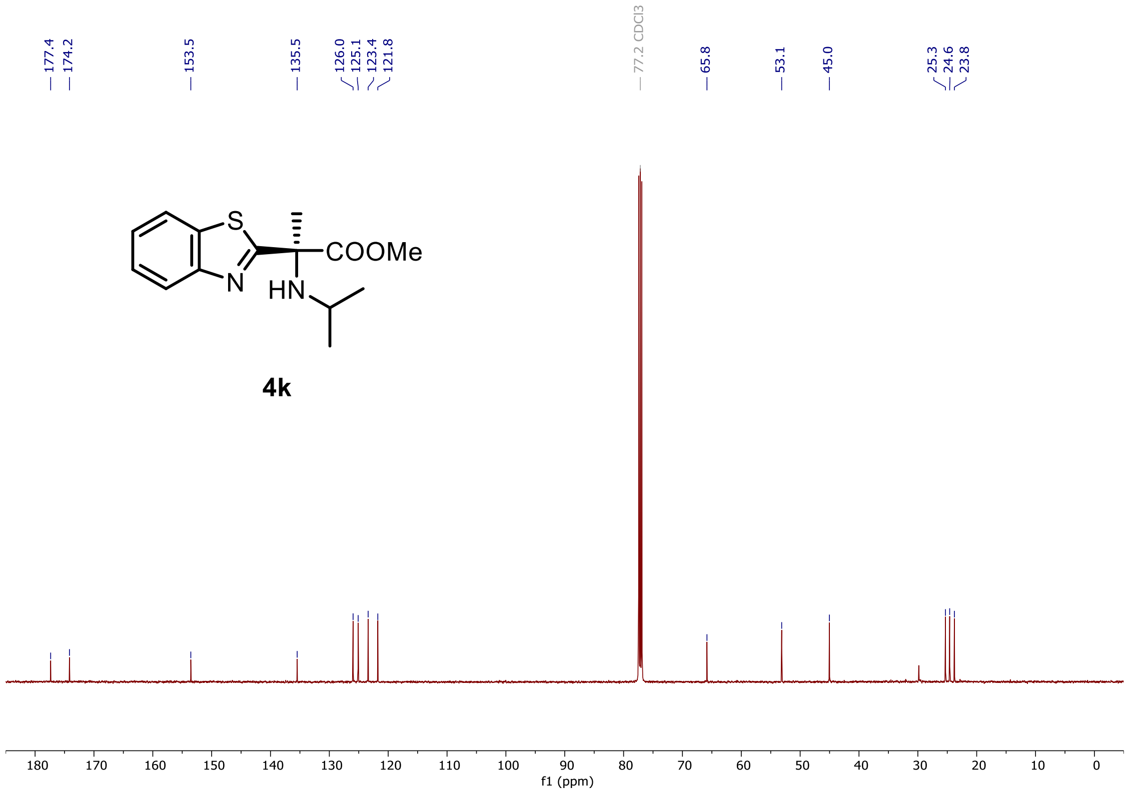

Copy of ^{1}H NMR Spectrum (500 MHz, $CDCl_3$) of **4l**

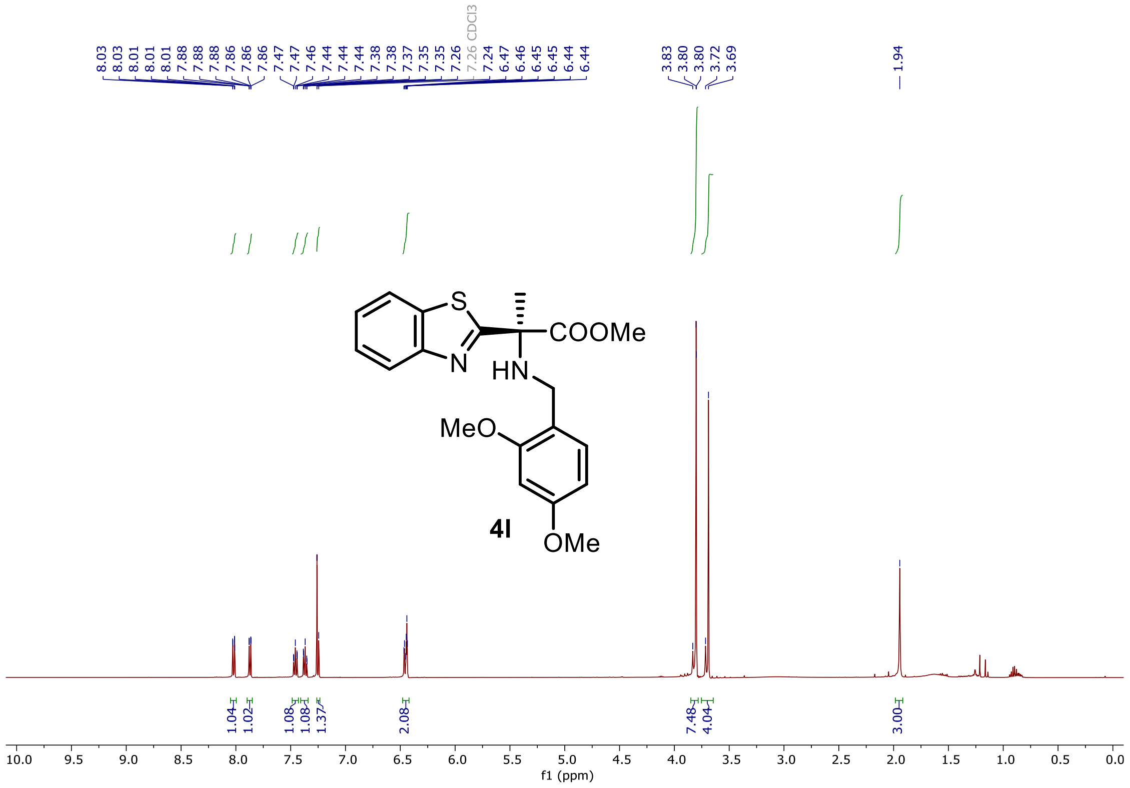

Copy of ^{13}C {^{1}H} NMR Spectrum (126 MHz, $CDCl_3$) of **4l**

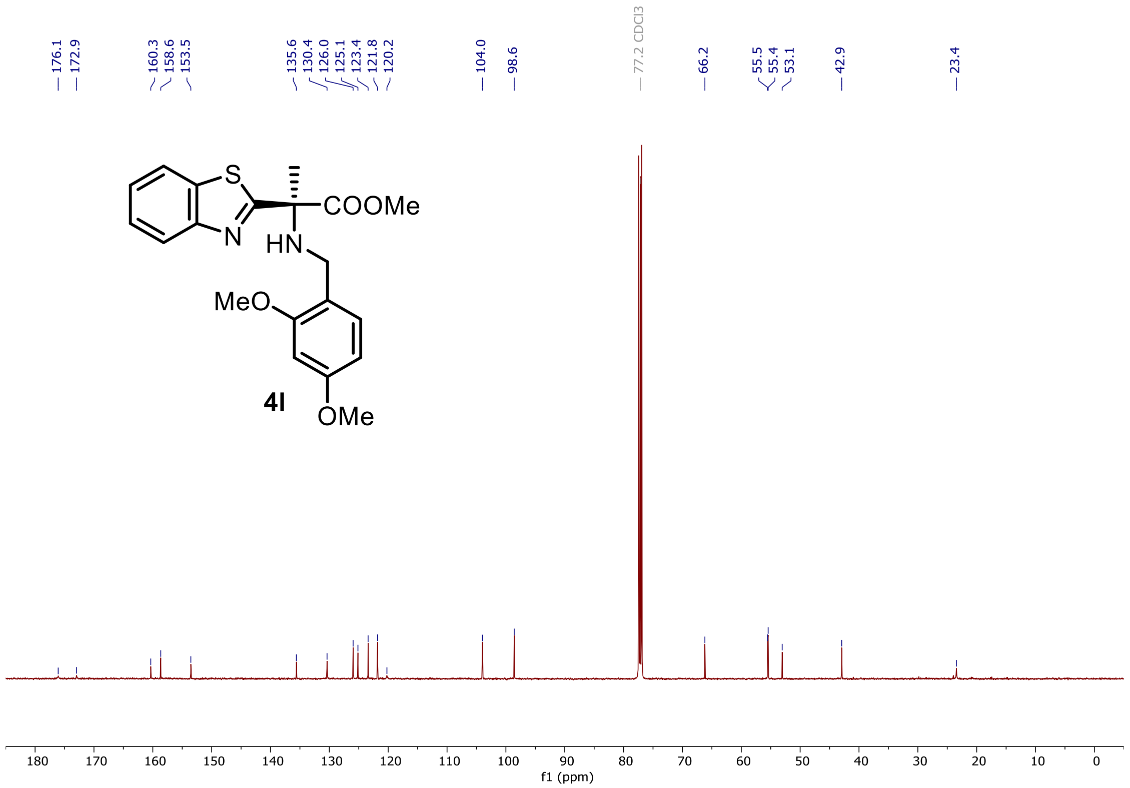

Copy of ^{1}H NMR Spectrum (500 MHz, $CDCl_3$) of **4m**

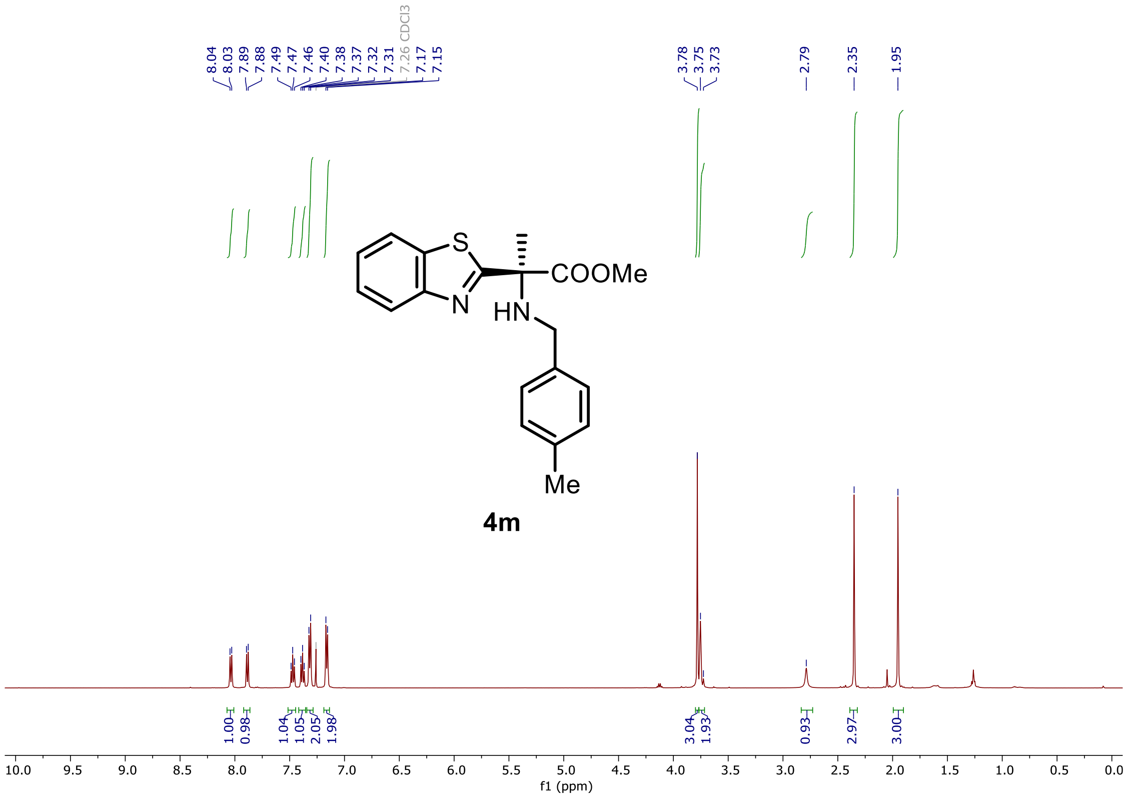

Copy of ^{13}C {^{1}H} NMR Spectrum (126 MHz, $CDCl_3$) of **4m**

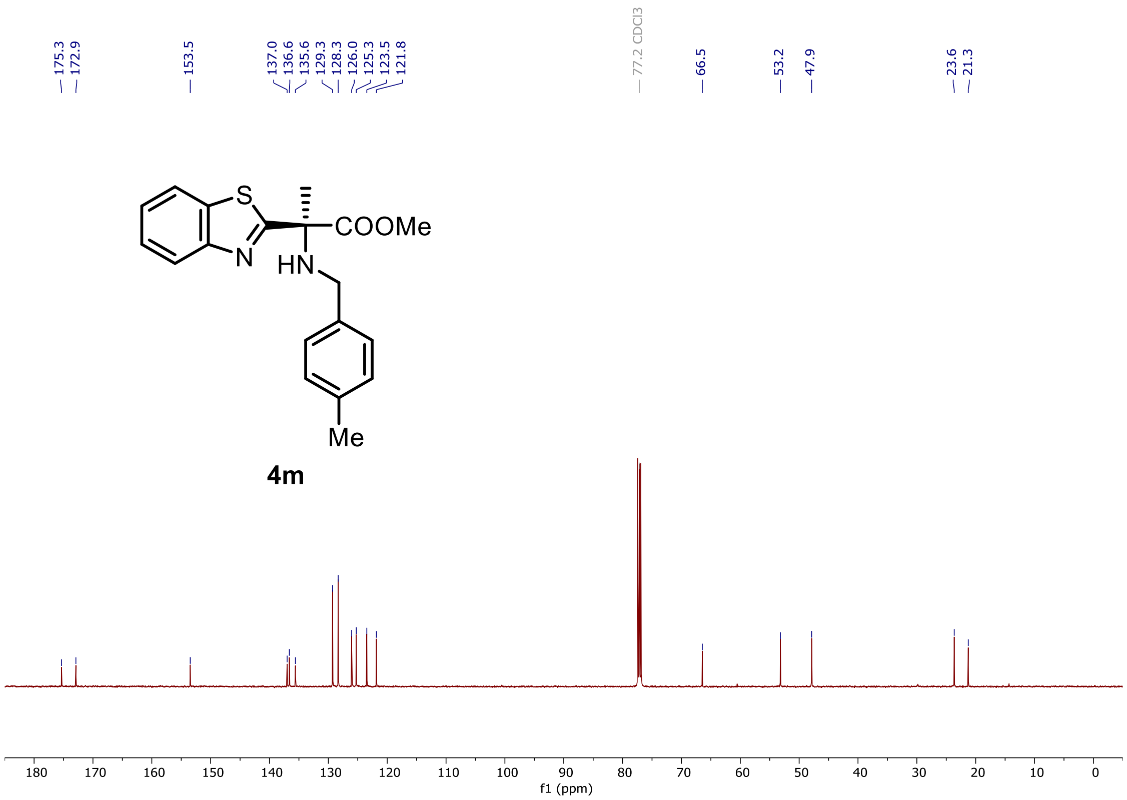

Copy of ^{1}H NMR Spectrum (400 MHz, $CDCl_3$) of **4n**

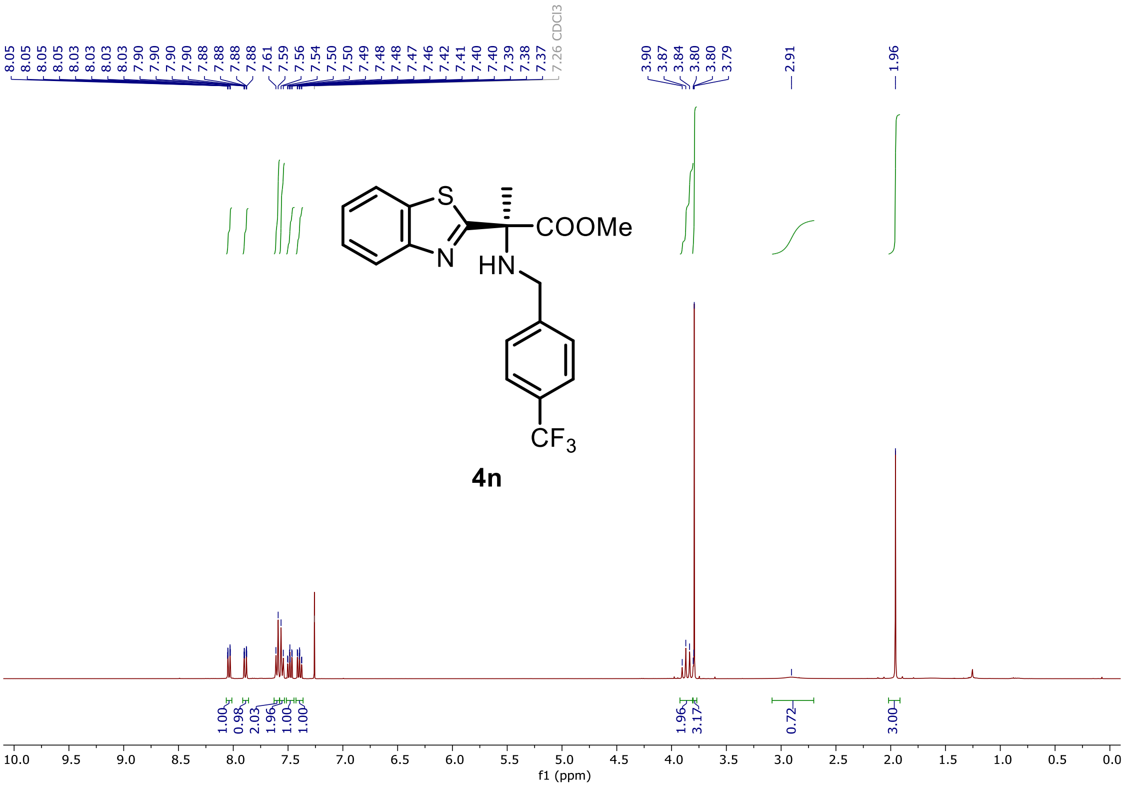

Copy of ^{13}C {^{1}H, ^{19}F} NMR Spectrum (101 MHz, $CDCl_3$) of **4n**

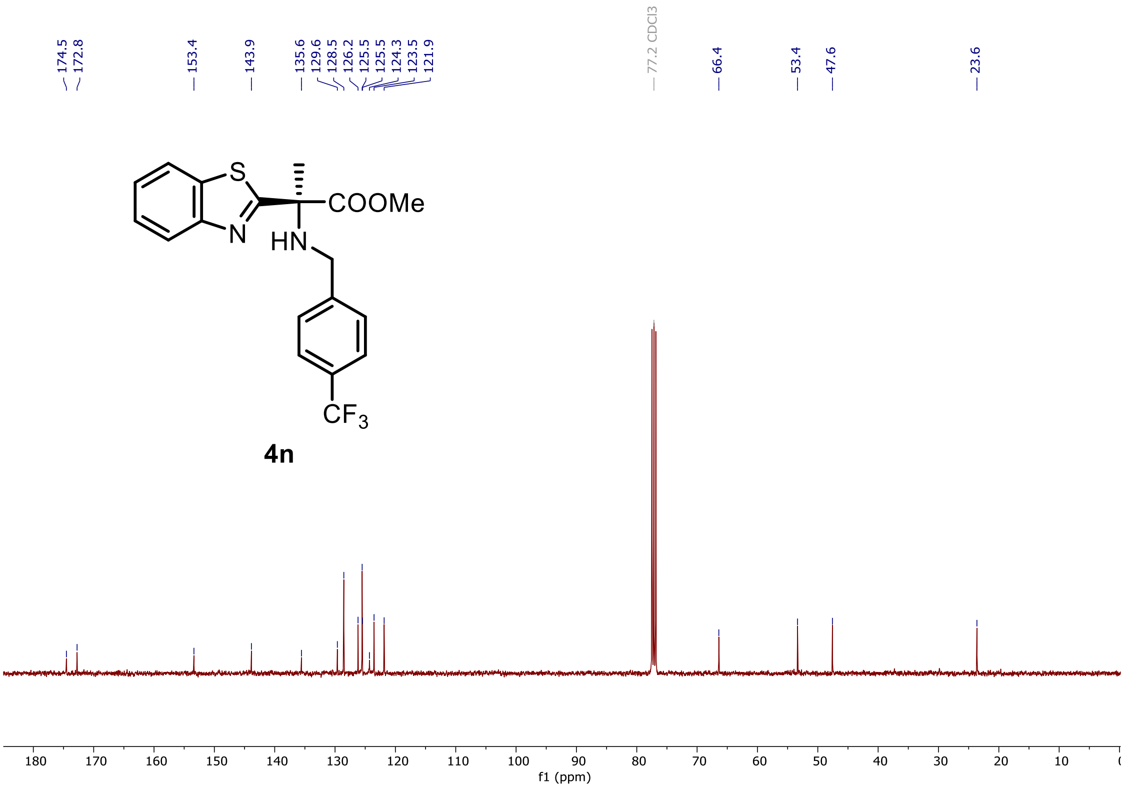

Copy of ^{19}FNMR Spectrum (376 MHz, $CDCl_3$) of **4n**

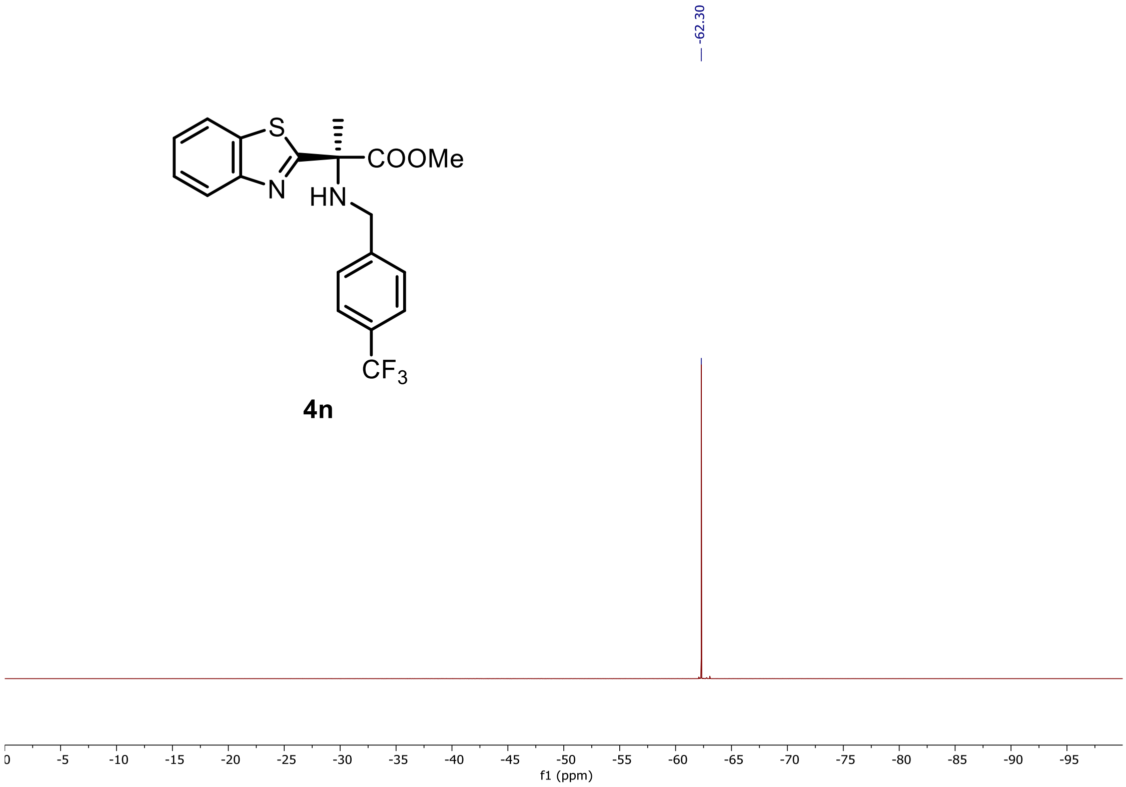

Copy of ^{1}H NMR Spectrum (500 MHz, $CDCl_3$) of **4o**

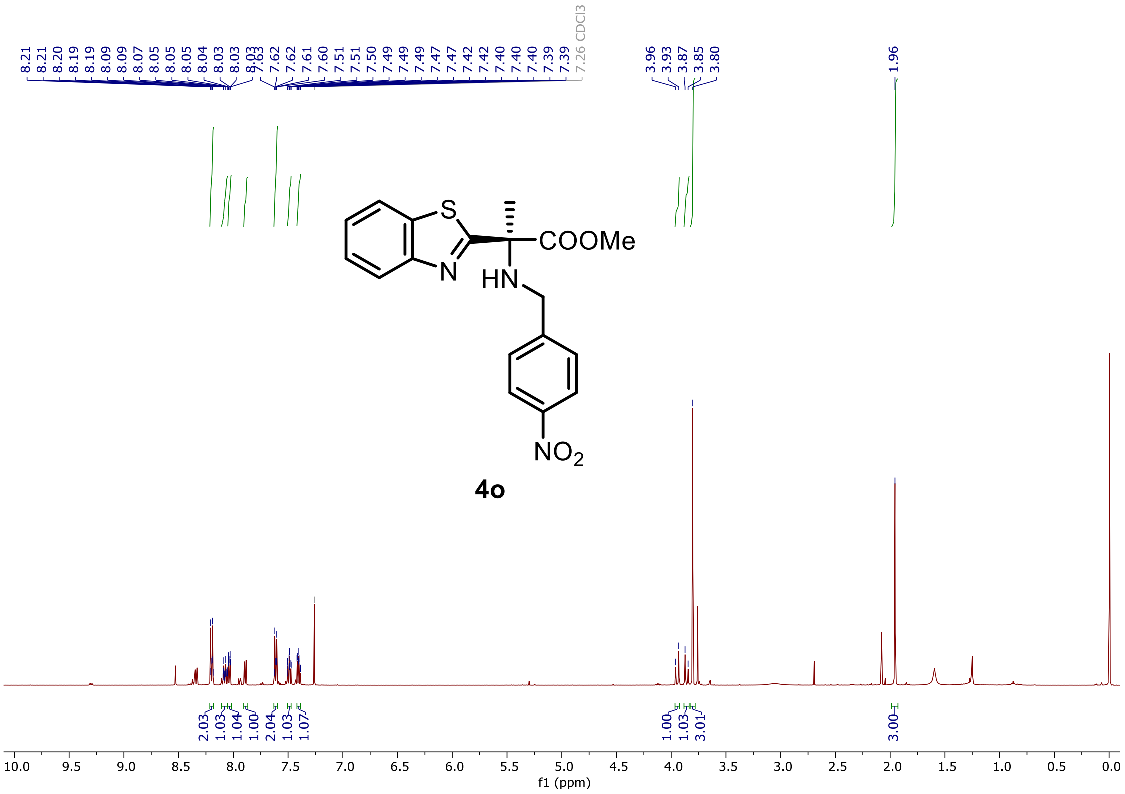

Copy of ^{13}C {^{1}H} NMR Spectrum (126 MHz, $CDCl_3$) of **4o**

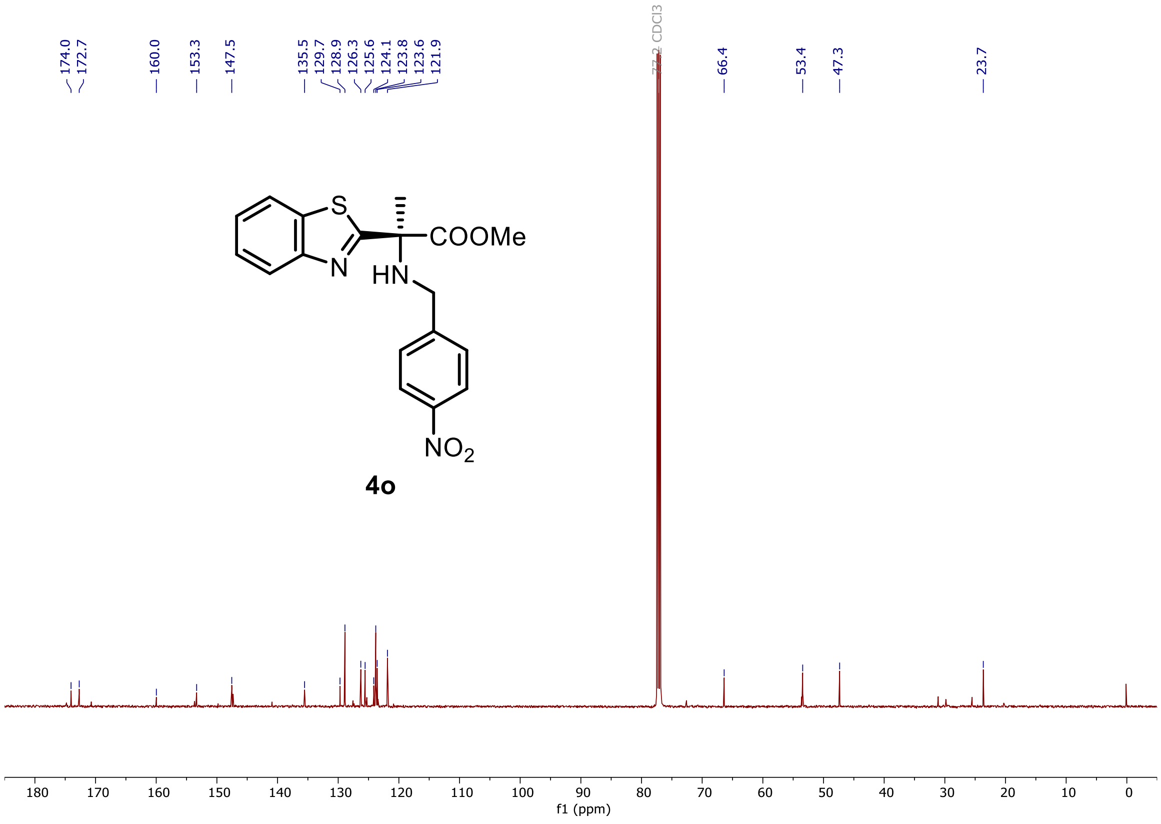

Copy of ^{1}H NMR Spectrum (500 MHz, $CDCl_3$) of **4p**

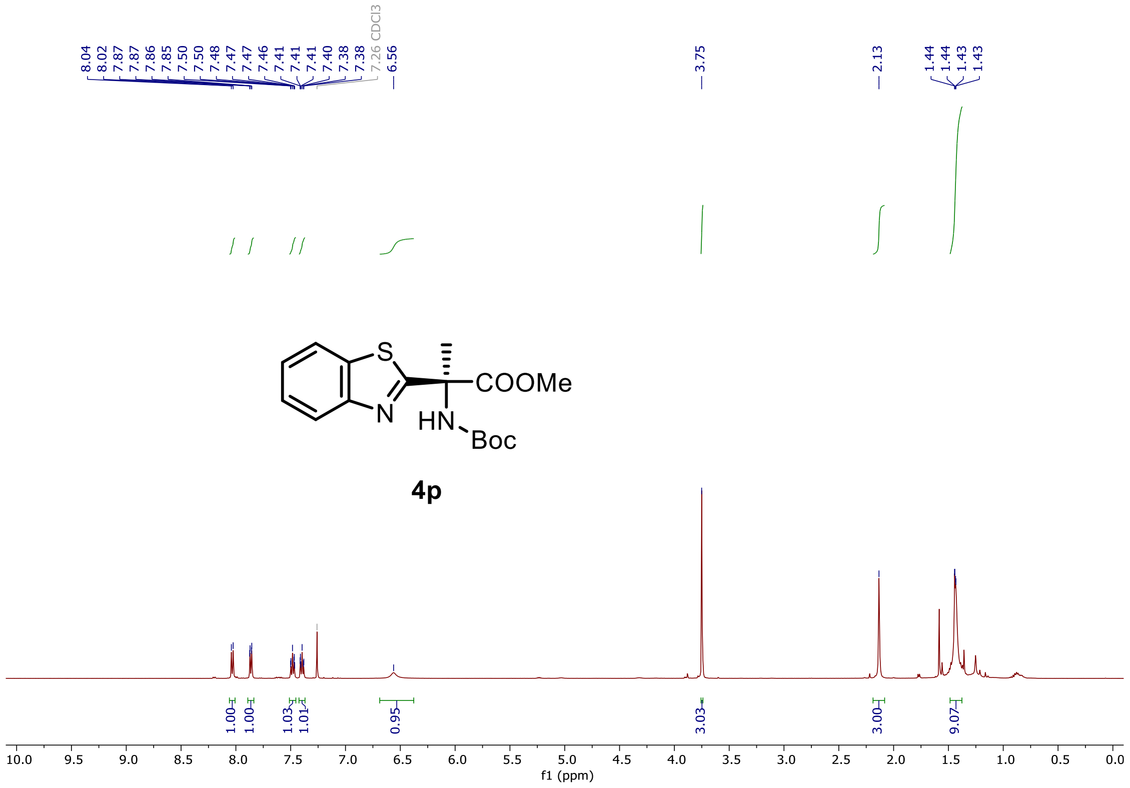

Copy of ^{13}C {^{1}H} NMR Spectrum (126 MHz, $CDCl_3$) of **4p**

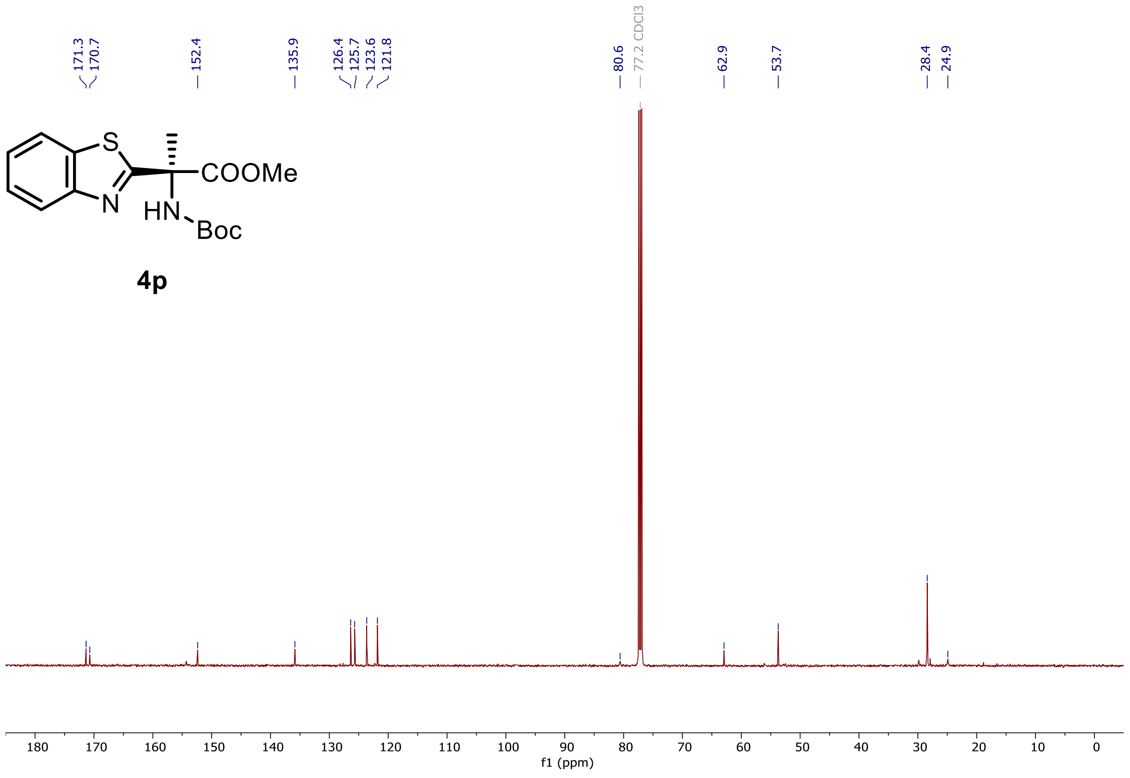

Copy of ^{1}H NMR Spectrum (400 MHz, $CDCl_3$) of **4q**

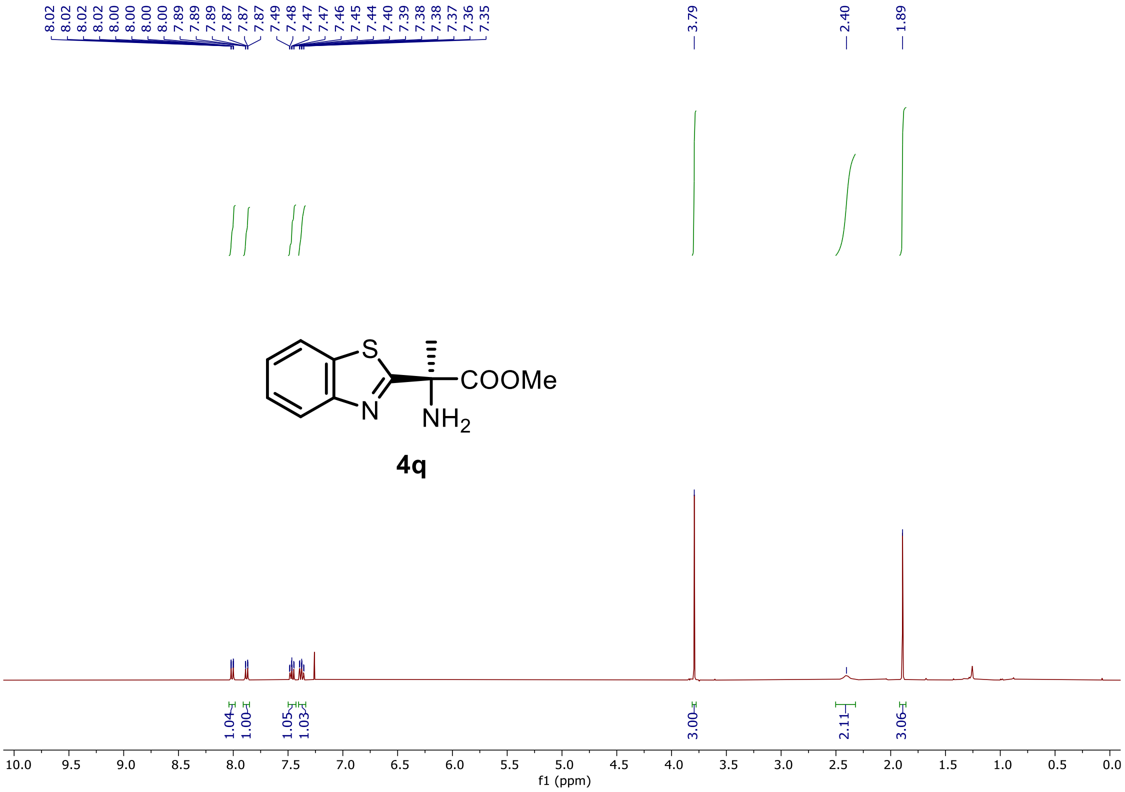

Copy of ^{13}C {^{1}H} NMR Spectrum (101 MHz, $CDCl_3$) of **4q**

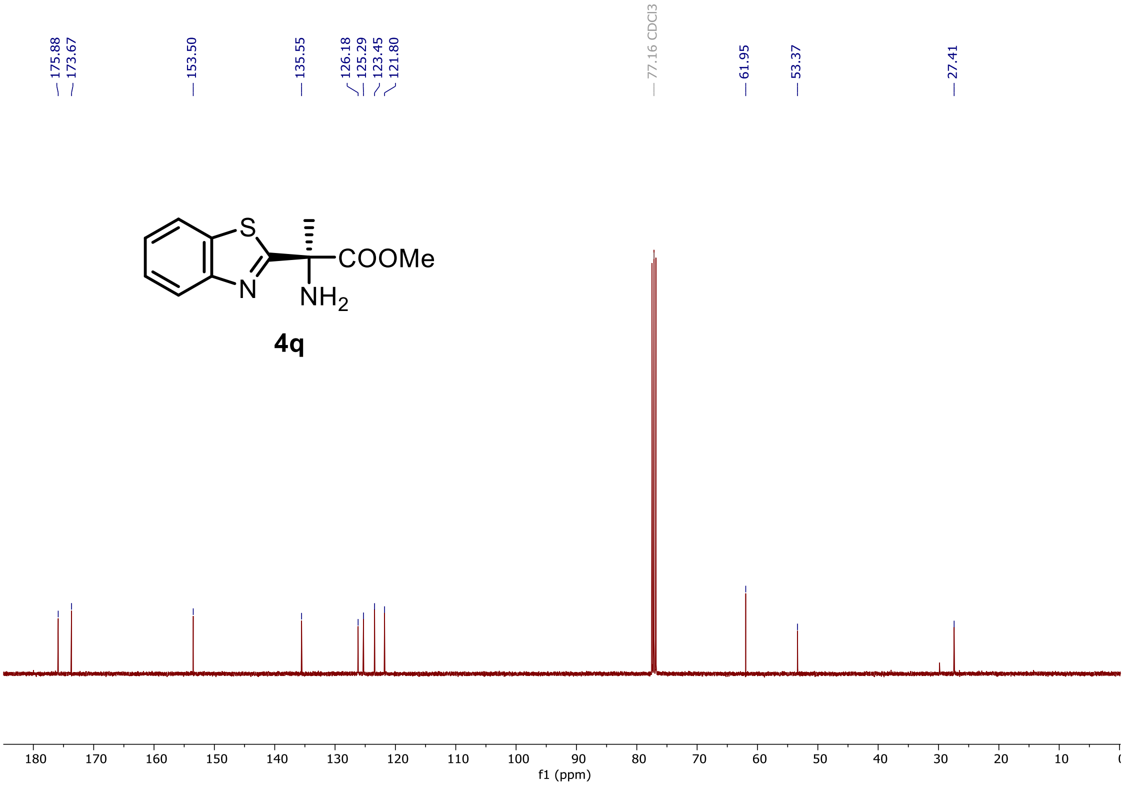

Copy of ^{1}H NMR Spectrum (500 MHz, $CDCl_3$) of **4r**

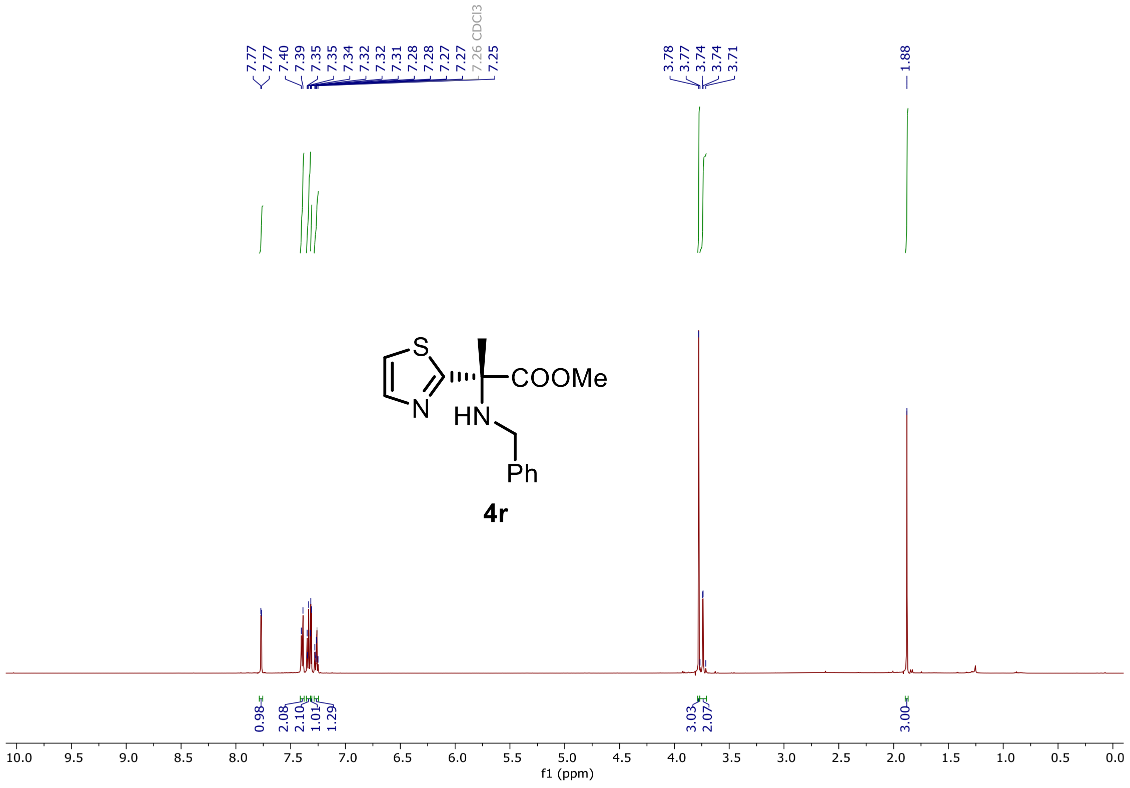

Copy of ^{13}C {^{1}H} NMR Spectrum (126 MHz, $CDCl_3$) of **4r**

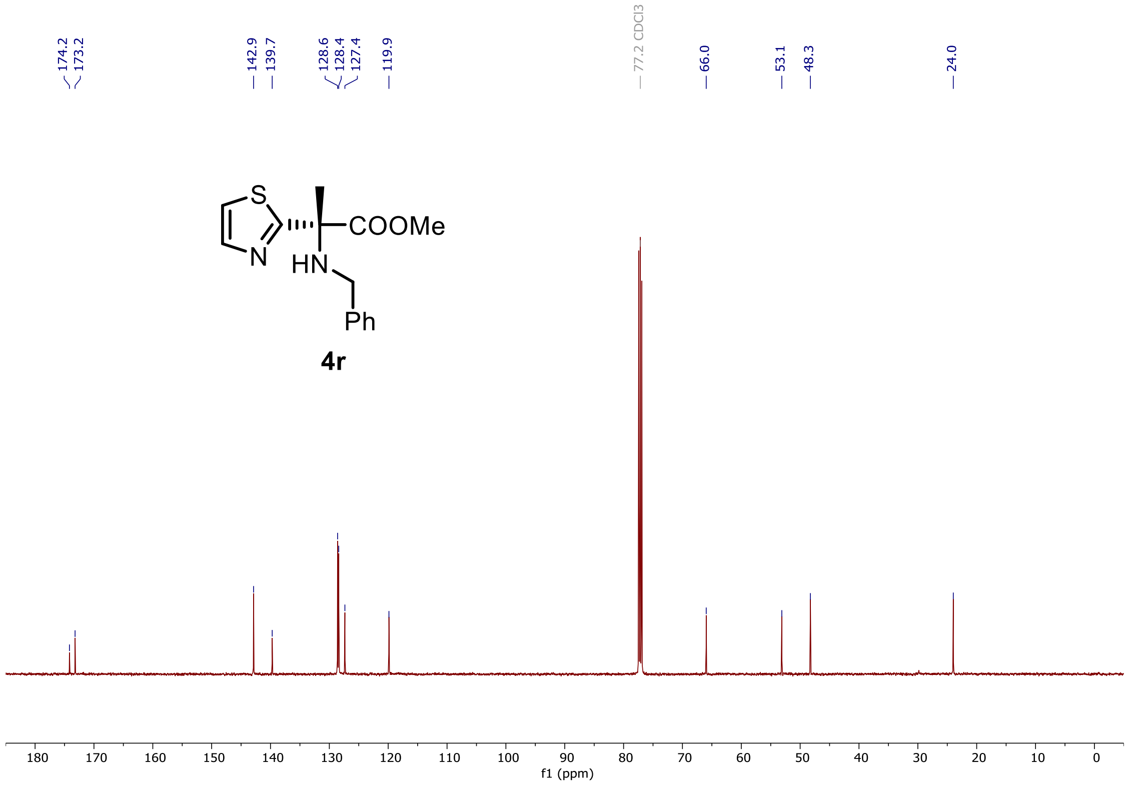

Copy of ^{1}H NMR Spectrum (500 MHz, $CDCl_3$) of **4s**

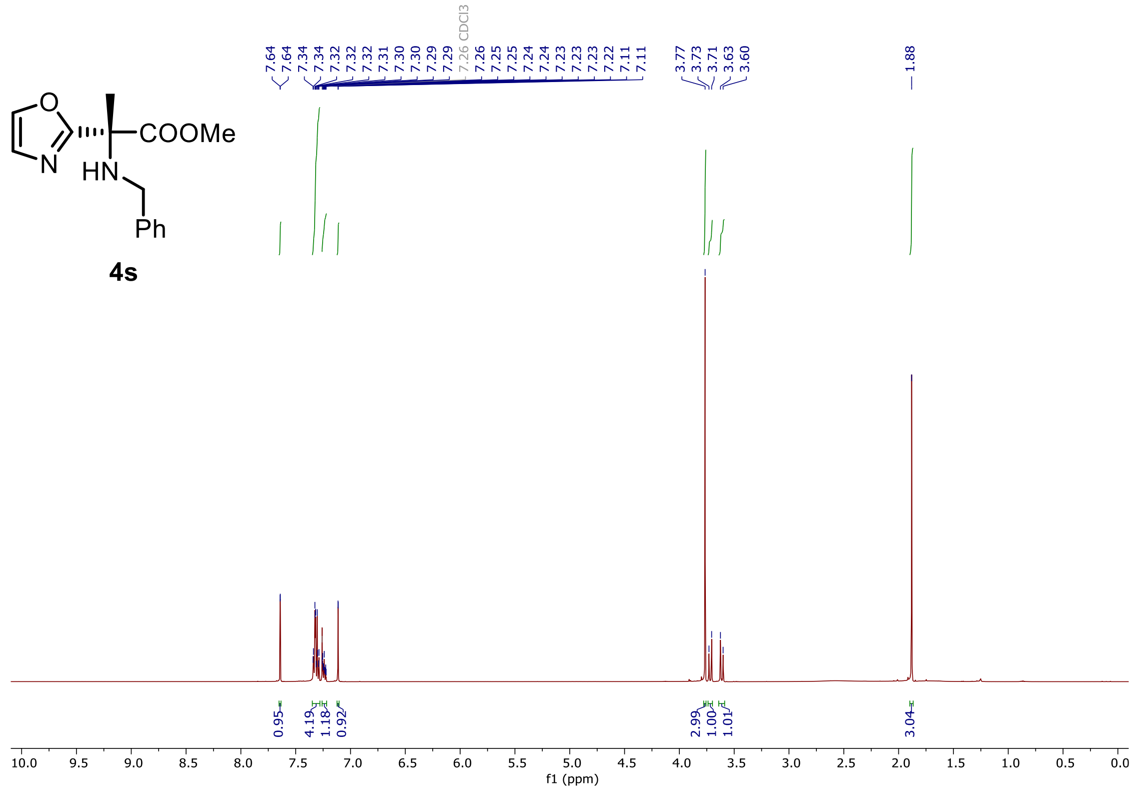

Copy of ^{13}C {^{1}H} NMR Spectrum (126 MHz, $CDCl_3$) of **4s**

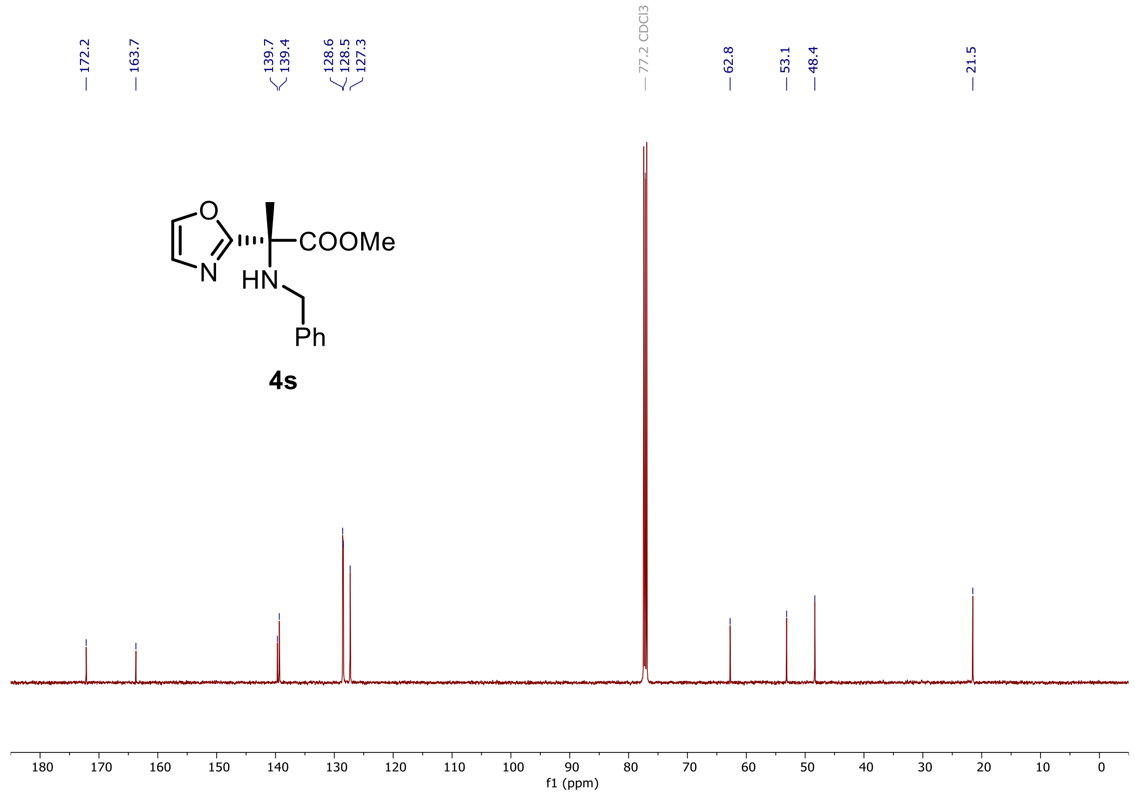

Copy of ^{1}H NMR Spectrum (500 MHz, $CDCl_3$) of **4t**

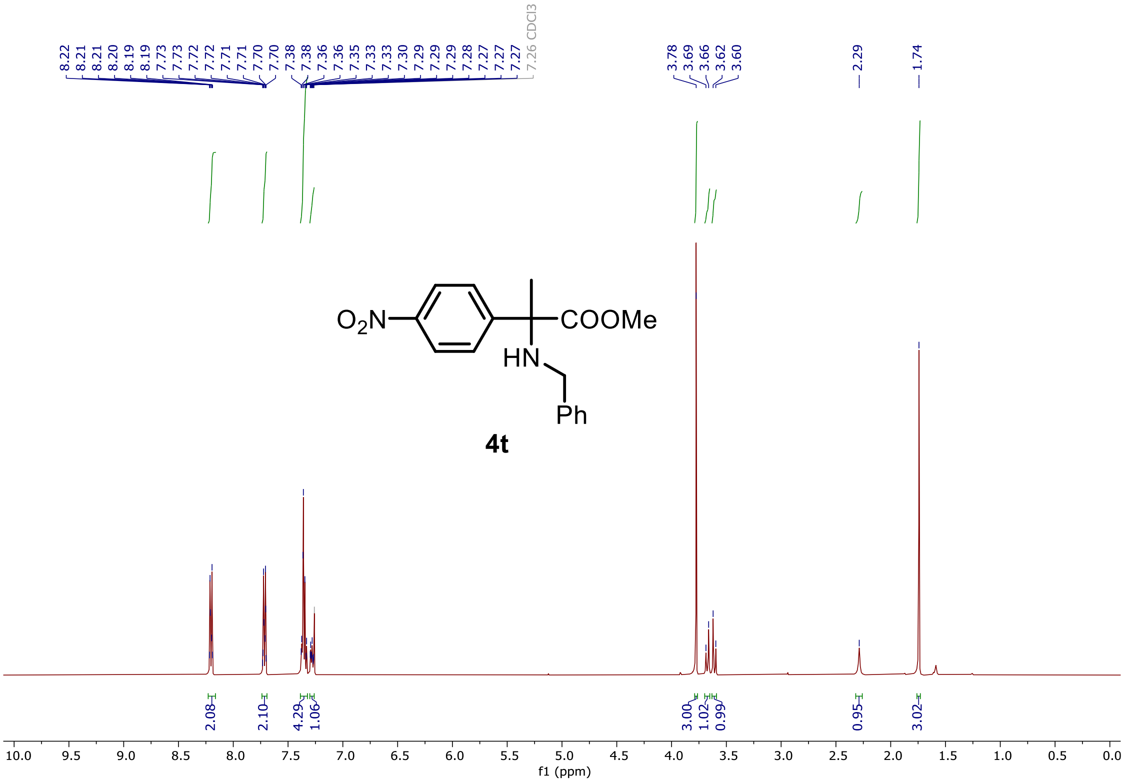

Copy of ^{13}C {^{1}H} NMR Spectrum (126 MHz, $CDCl_3$) of **4t**

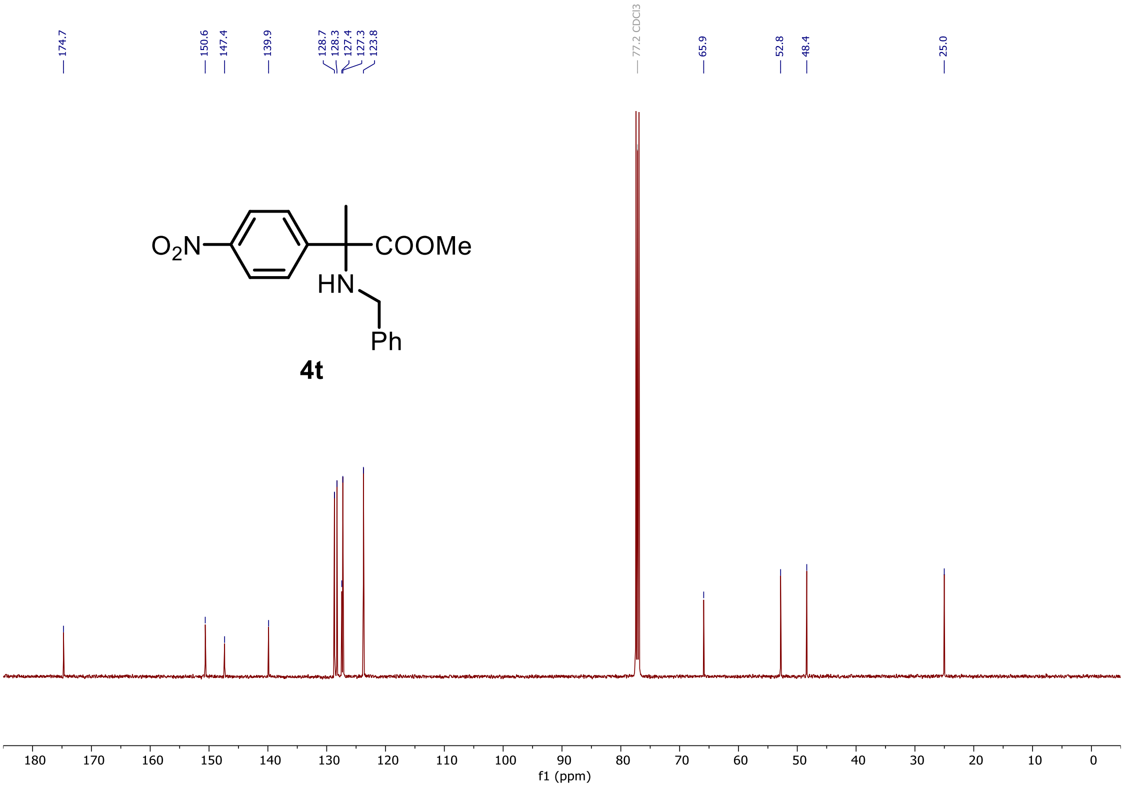

Copy of ^{1}H NMR Spectrum (500 MHz, $CDCl_3$) of **4u**

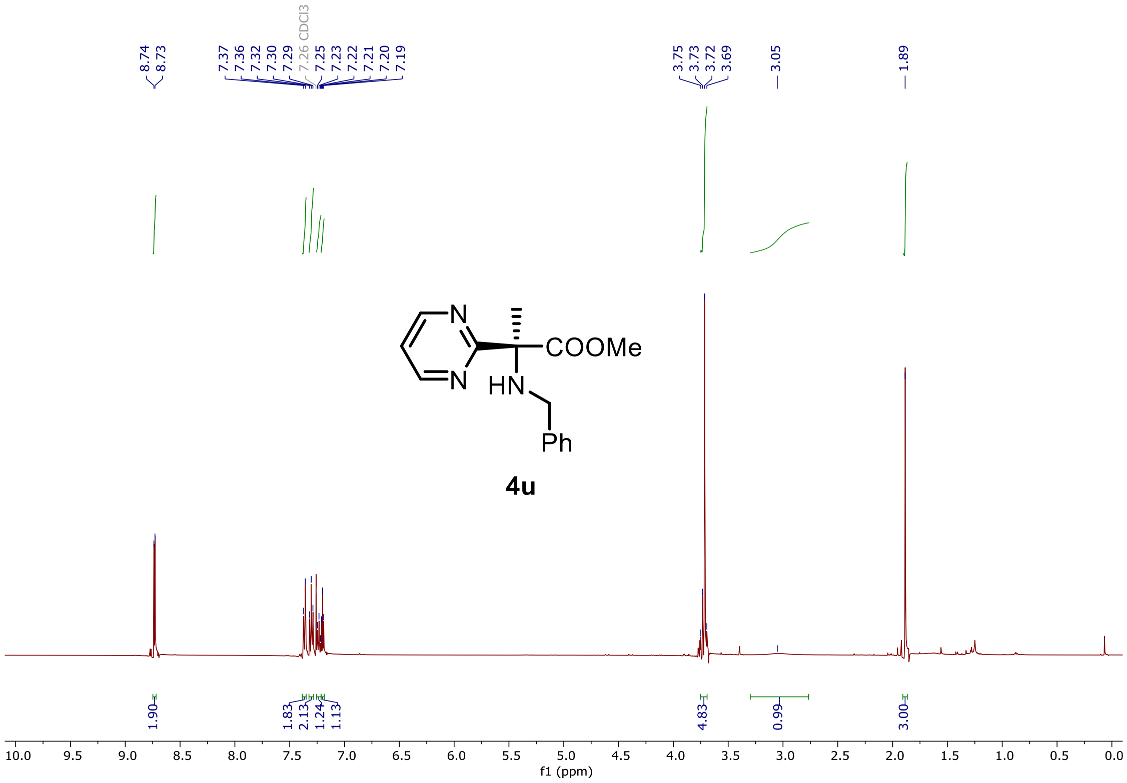

Copy of ^{13}C {^{1}H} NMR Spectrum (126 MHz, $CDCl_3$) of **4u**

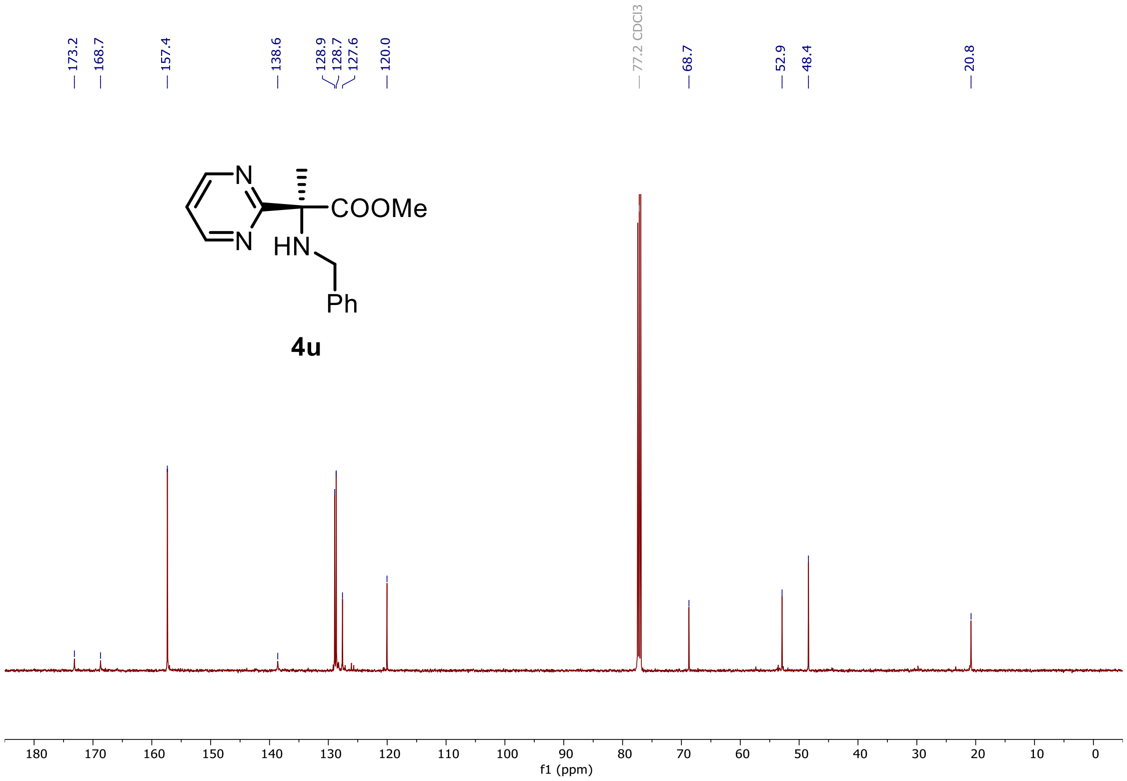

Copy of ^{1}H NMR Spectrum (500 MHz, $CDCl_3$) of **1a**

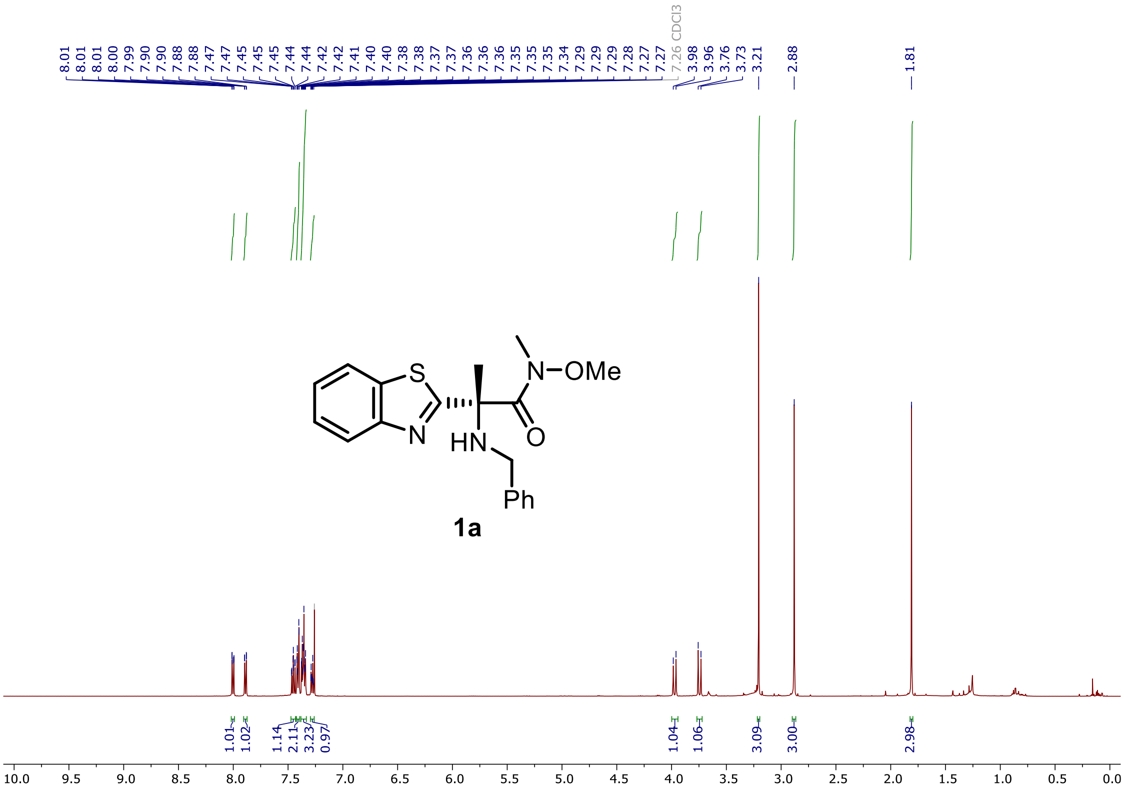

Copy of ^{13}C {^{1}H} NMR Spectrum (126 MHz, $CDCl_3$) of **1a**

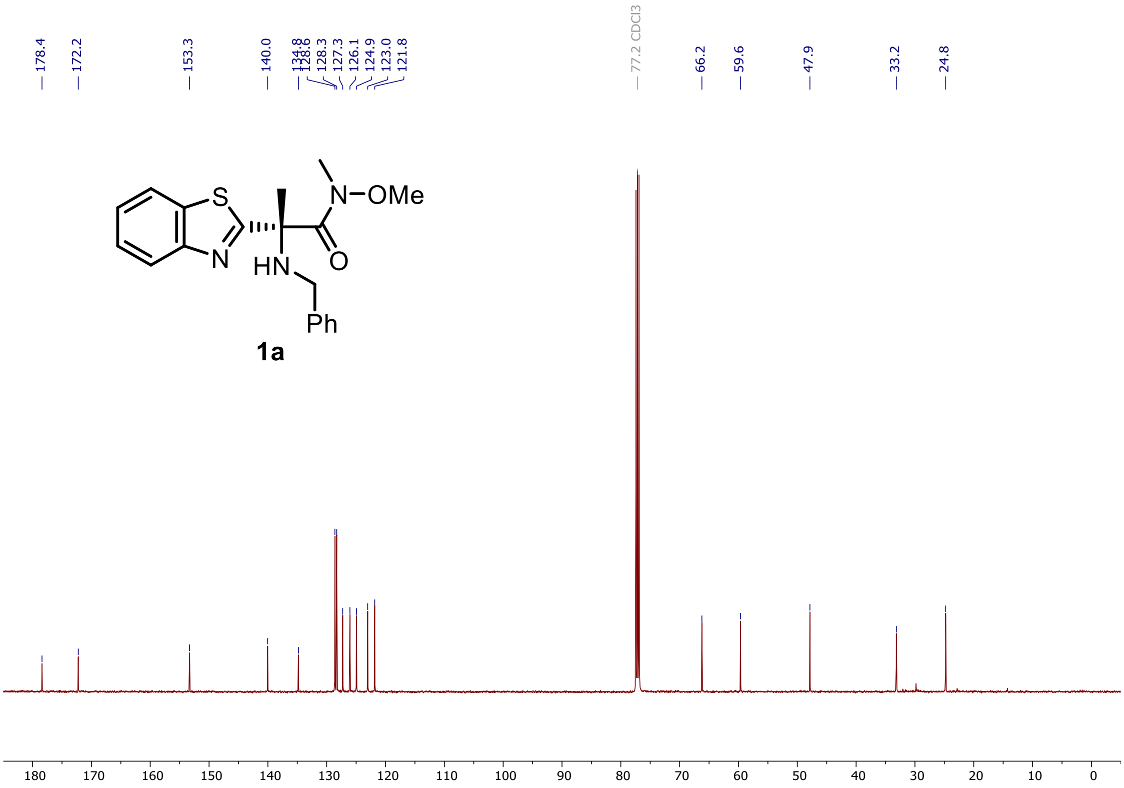

Copy of ^{1}H NMR Spectrum (400 MHz, $CDCl_3$) of **S1**

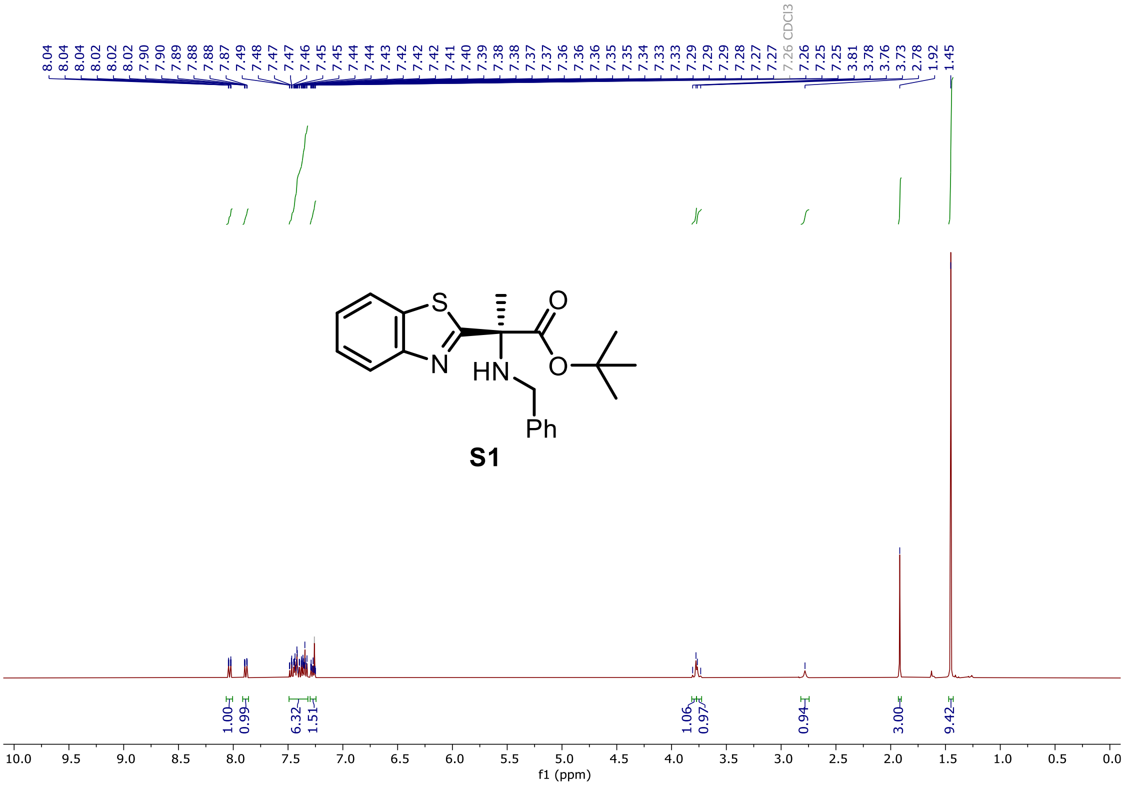

Copy of ^{13}C {^{1}H} NMR Spectrum (101 MHz, $CDCl_3$) of **S1**

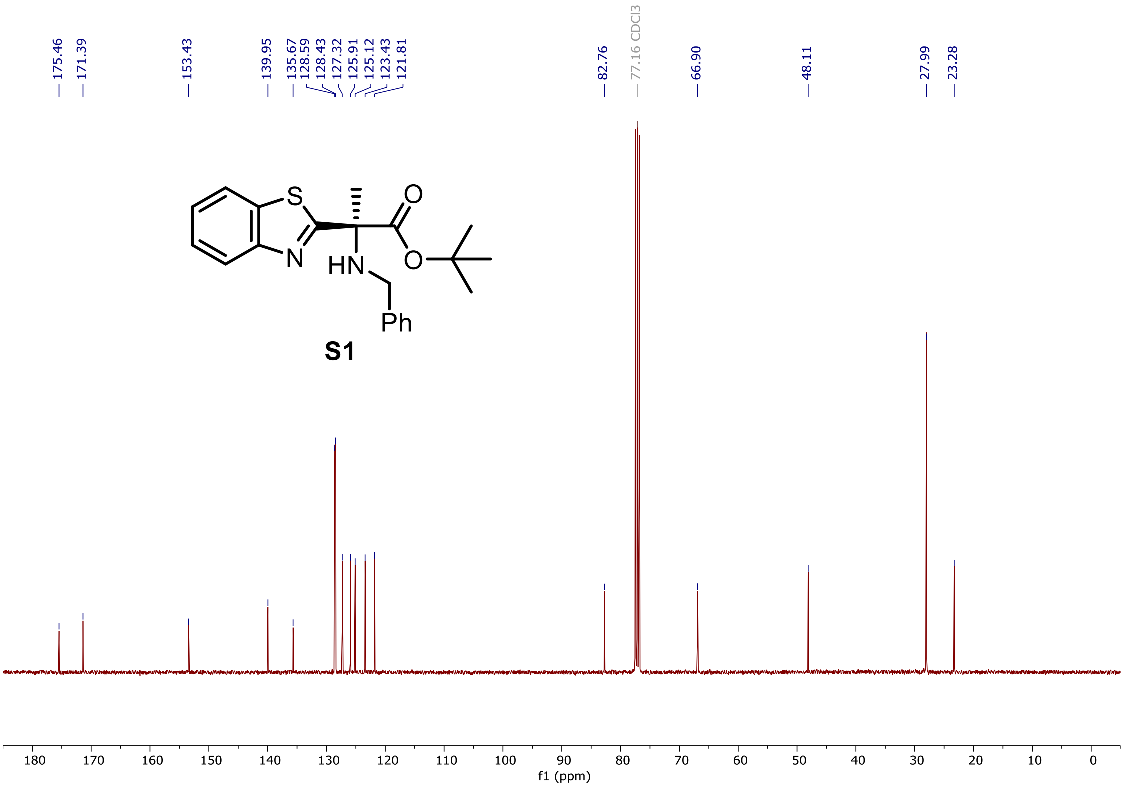

Copy of ^{1}H NMR Spectrum (400 MHz, $CDCl_3$) of **S26**

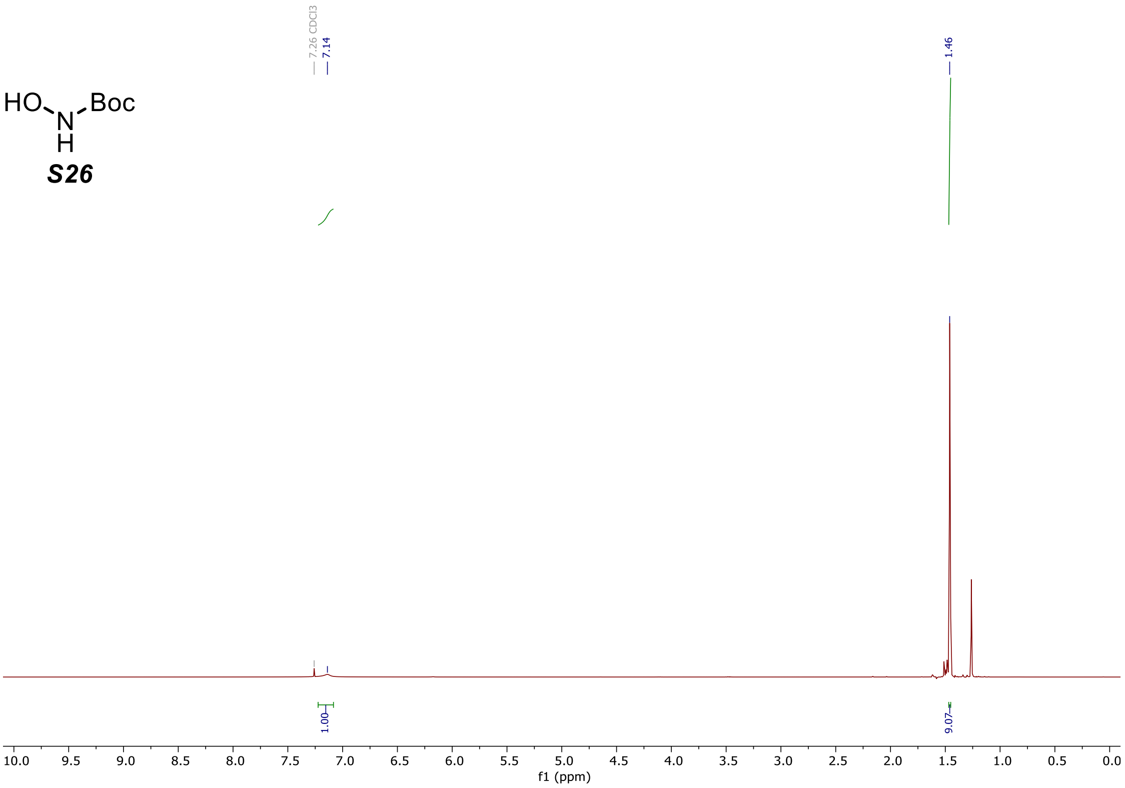

Copy of ^{13}C {^{1}H} NMR Spectrum (101 MHz, $CDCl_3$) of **S26**

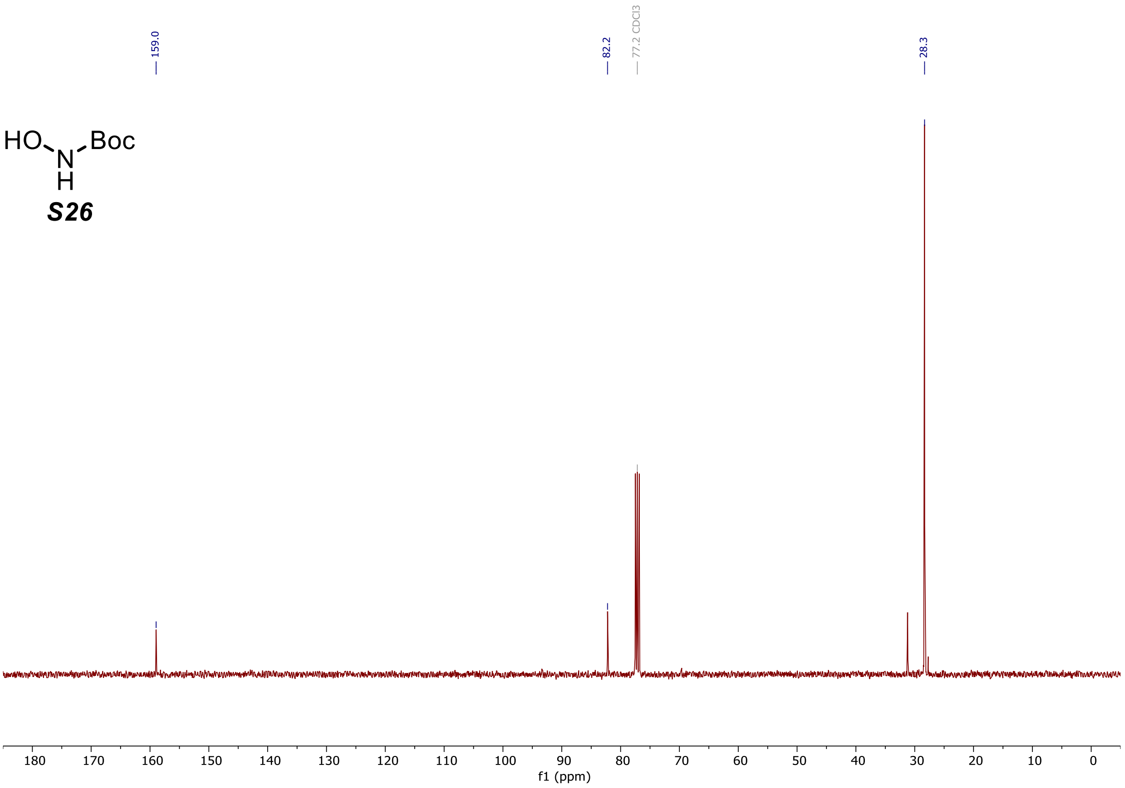

Copy of ^{1}H NMR Spectrum (400 MHz, $CDCl_3$) of **S28**

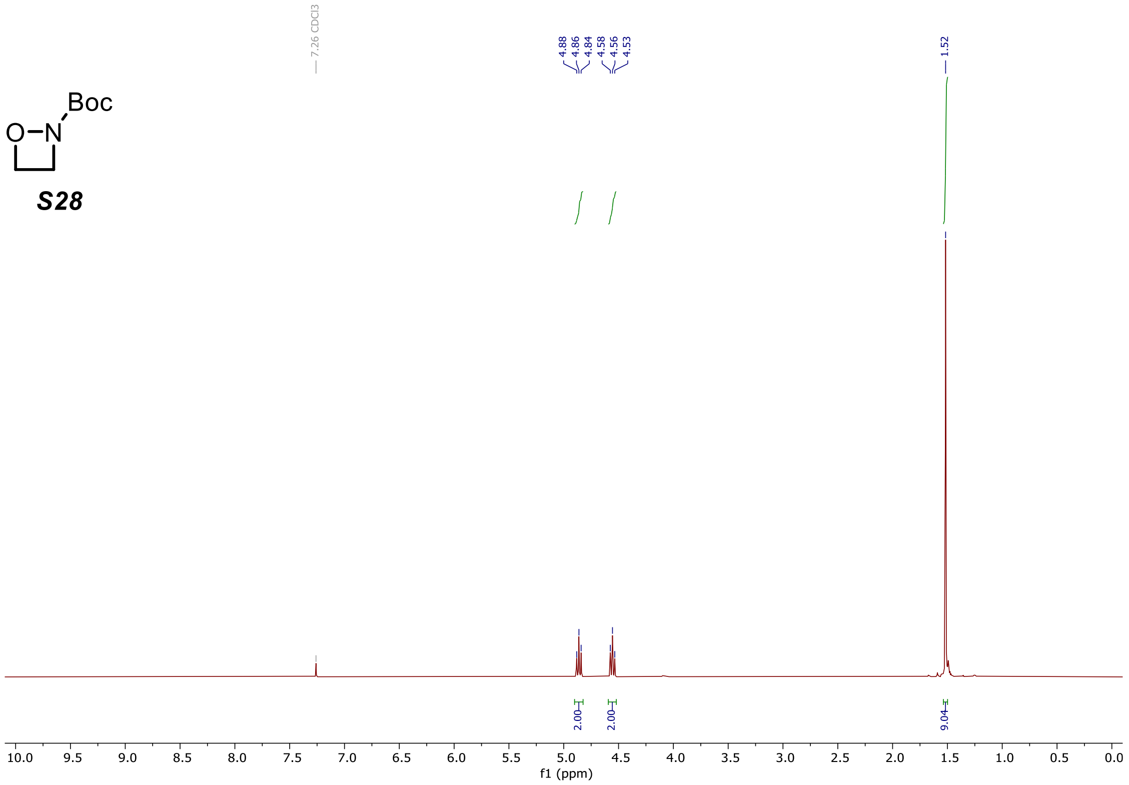

Copy of ^{13}C {^{1}H} NMR Spectrum (101 MHz, $CDCl_3$) of **S28**

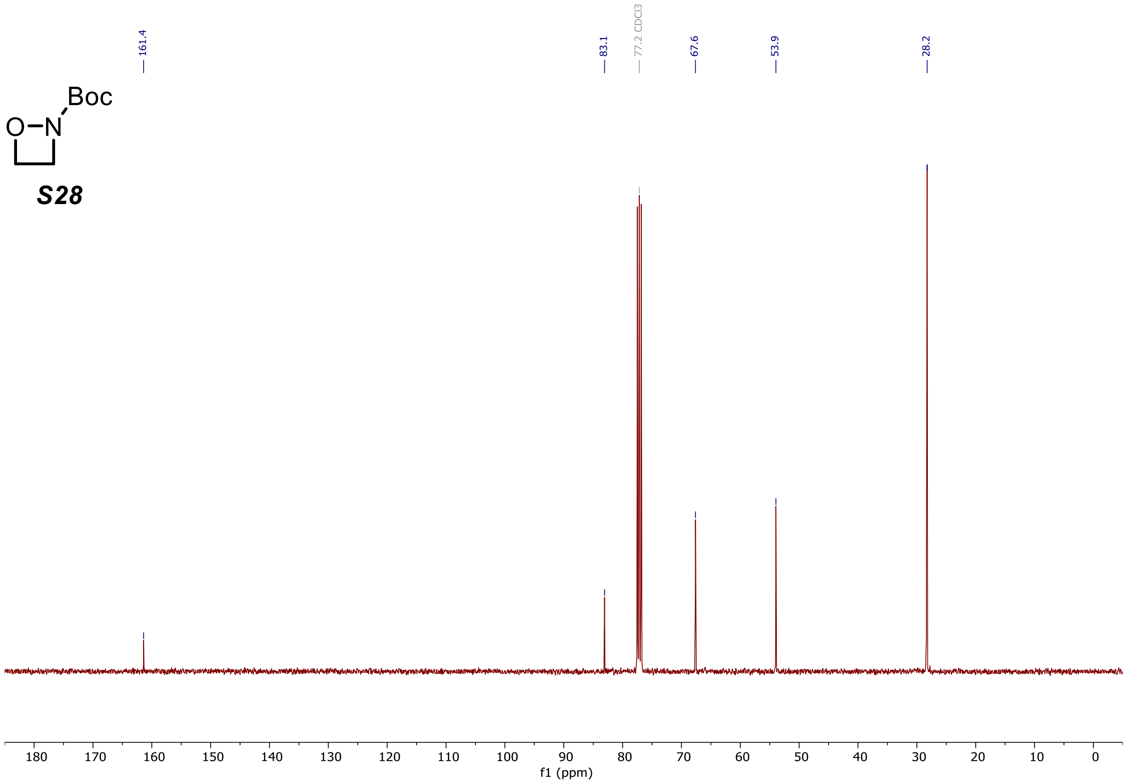

Copy of ^{1}H NMR Spectrum (400 MHz, CD_3OD) of **5b**

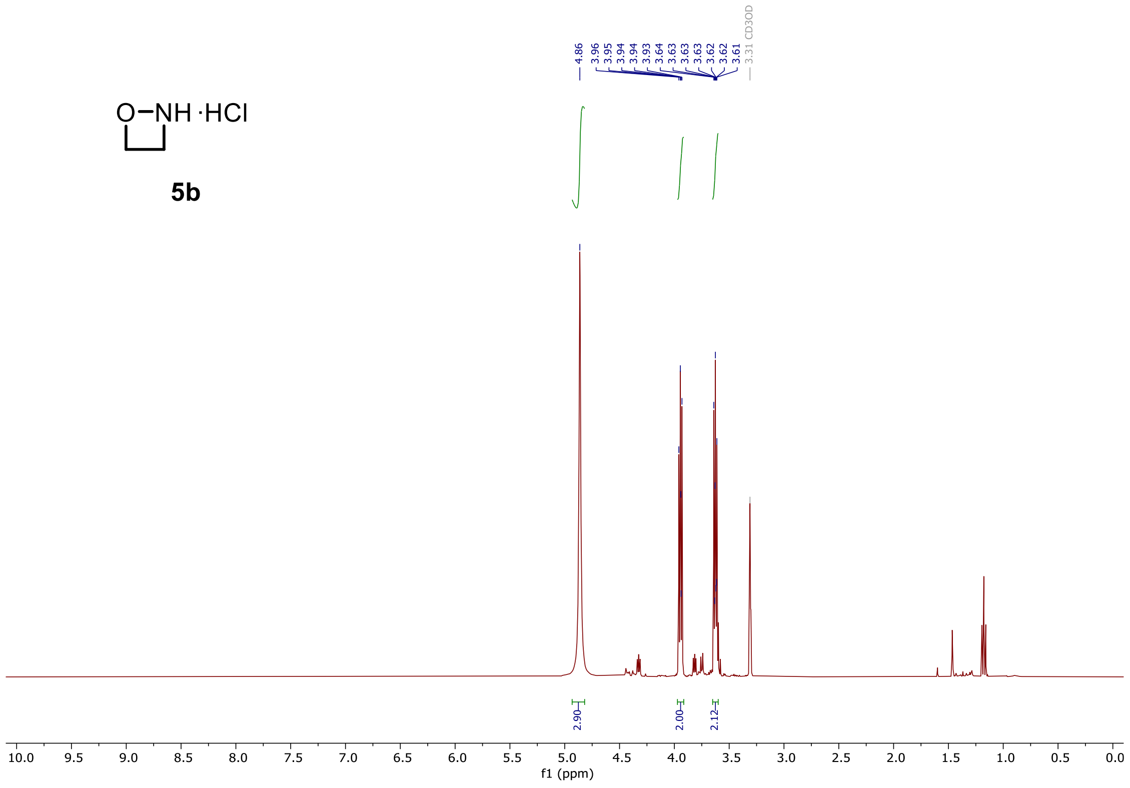

Copy of ^{13}C {^{1}H} NMR Spectrum (101 MHz, CD_3OD) of **5b**

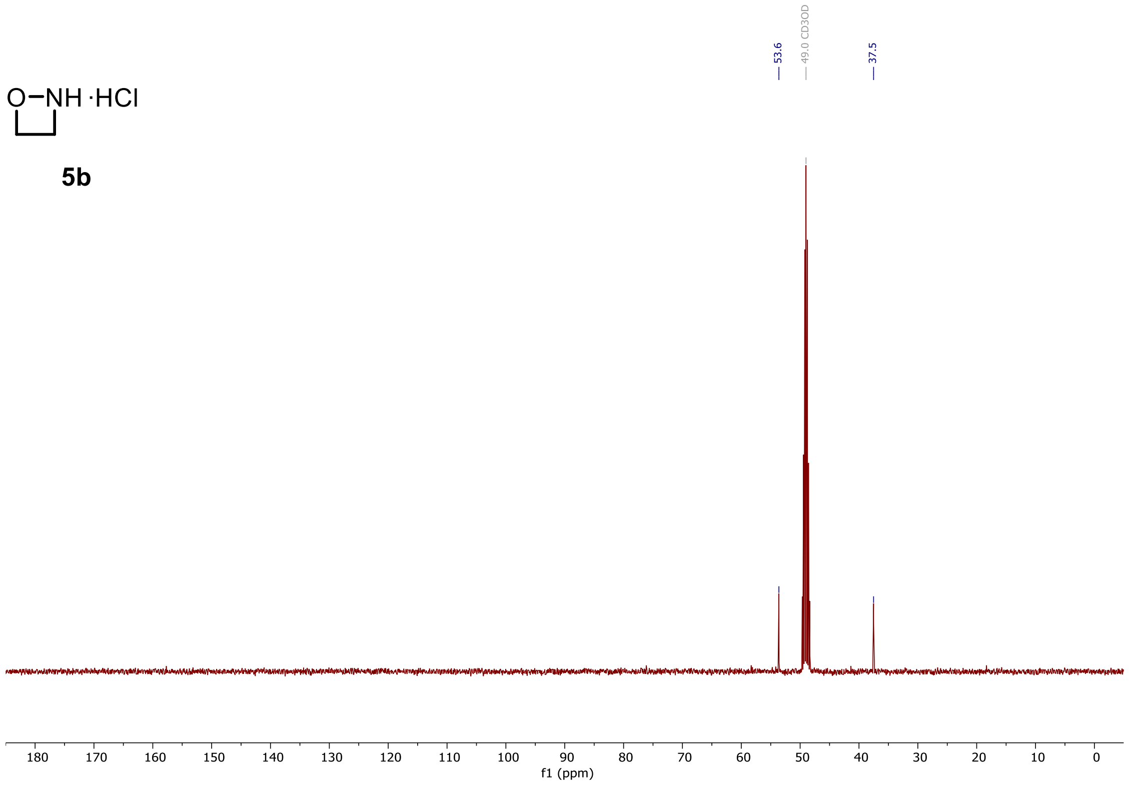

Copy of ^{1}H NMR Spectrum (400 MHz, $CDCl_3$) of **S29**

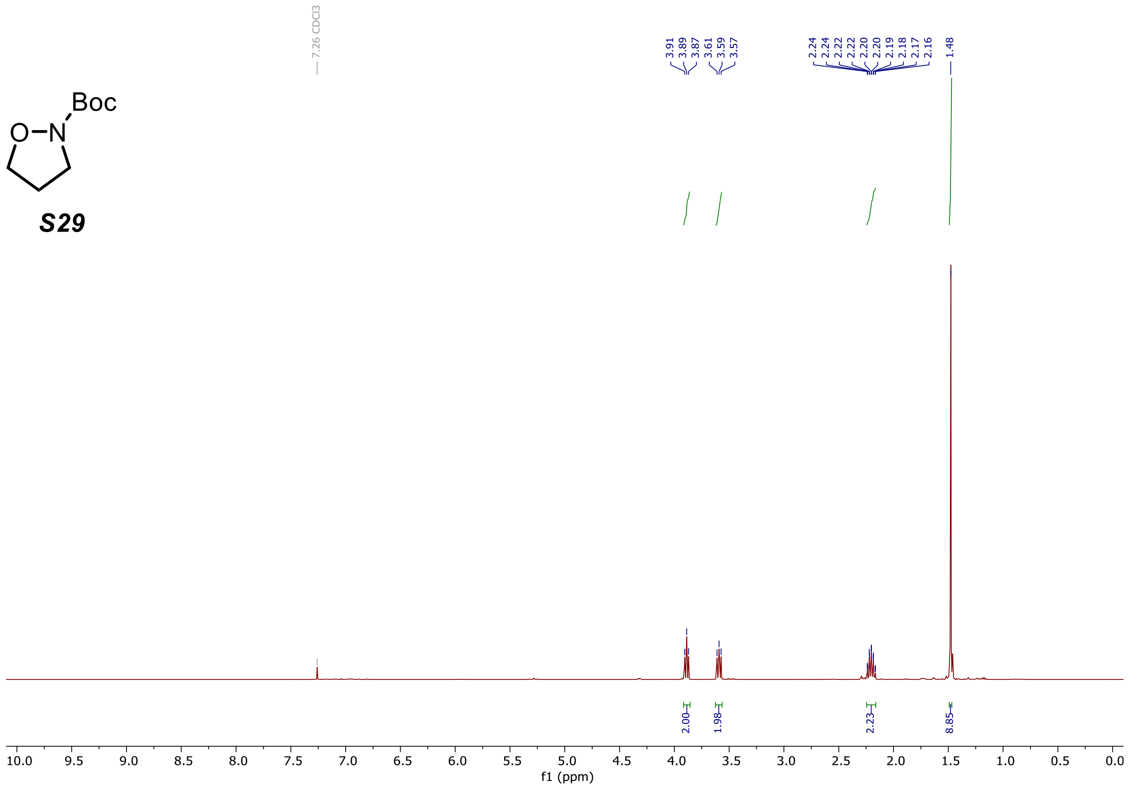

Copy of ^{13}C {^{1}H} NMR Spectrum (101 MHz, $CDCl_3$) of **S29**

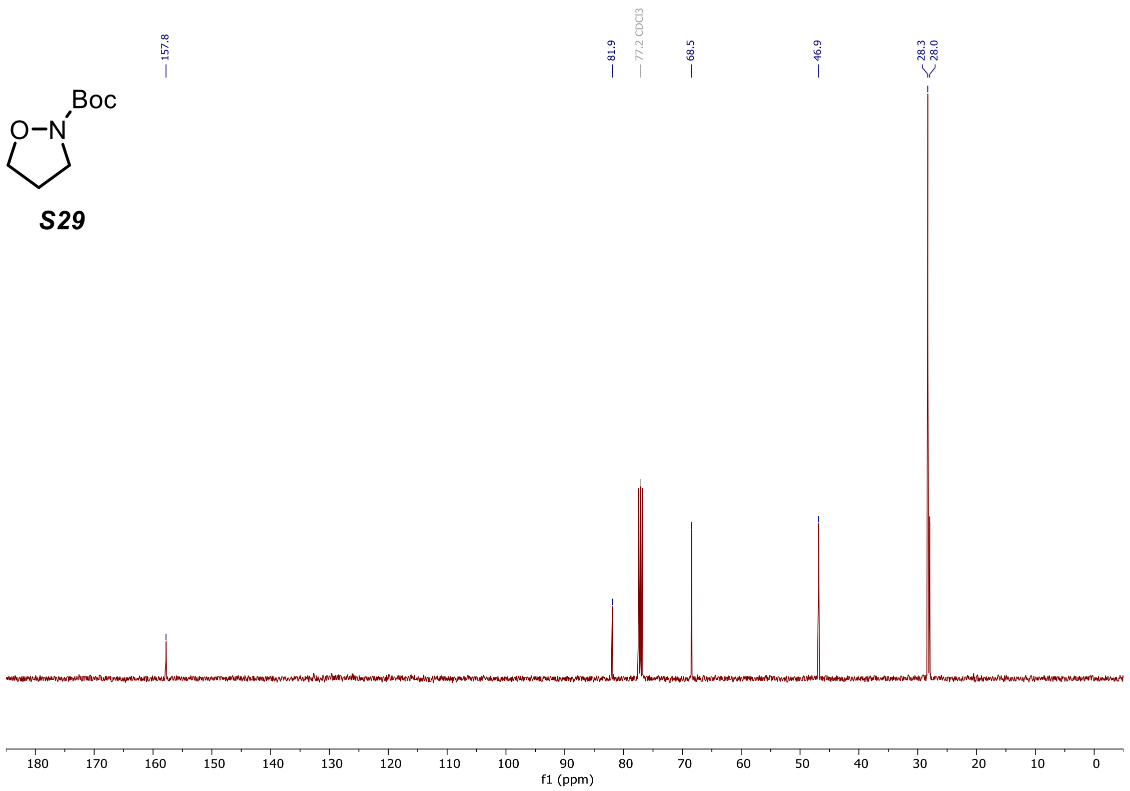

Copy of ^{1}H NMR Spectrum (400 MHz, CD_3OD) of **5c**

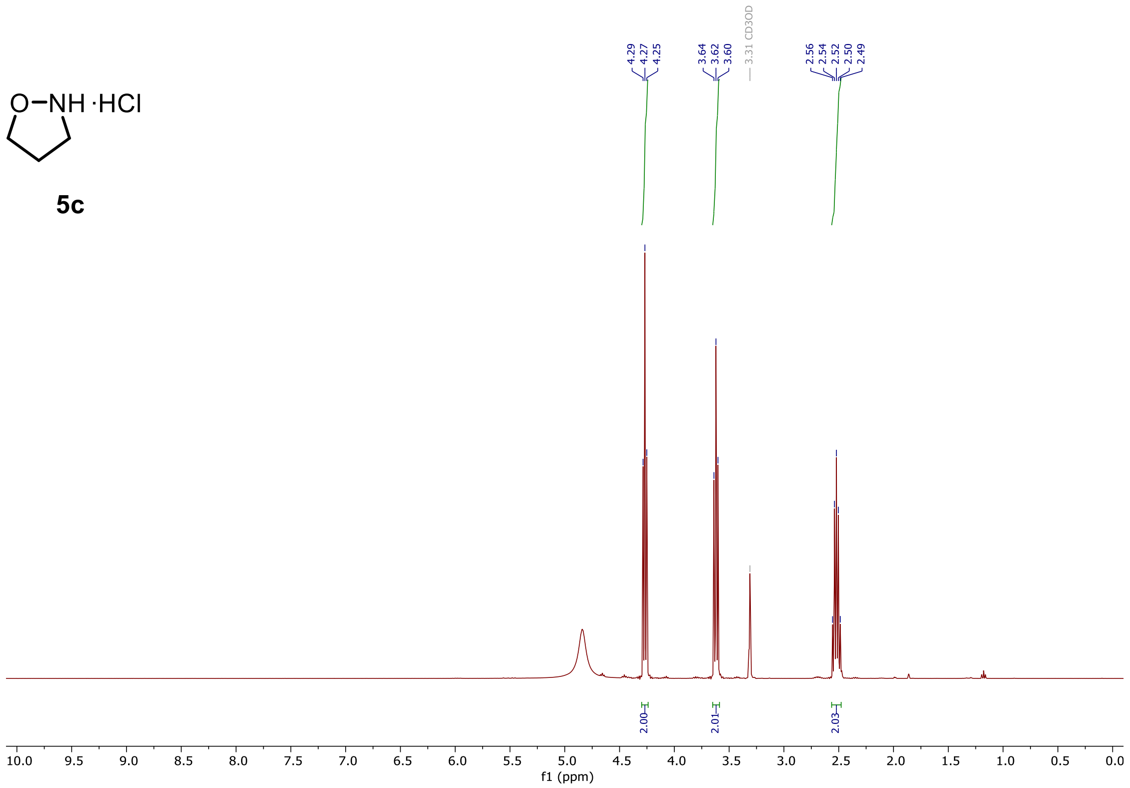

Copy of ^{13}C {^{1}H} NMR Spectrum (101 MHz, CD_3OD) of **5c**

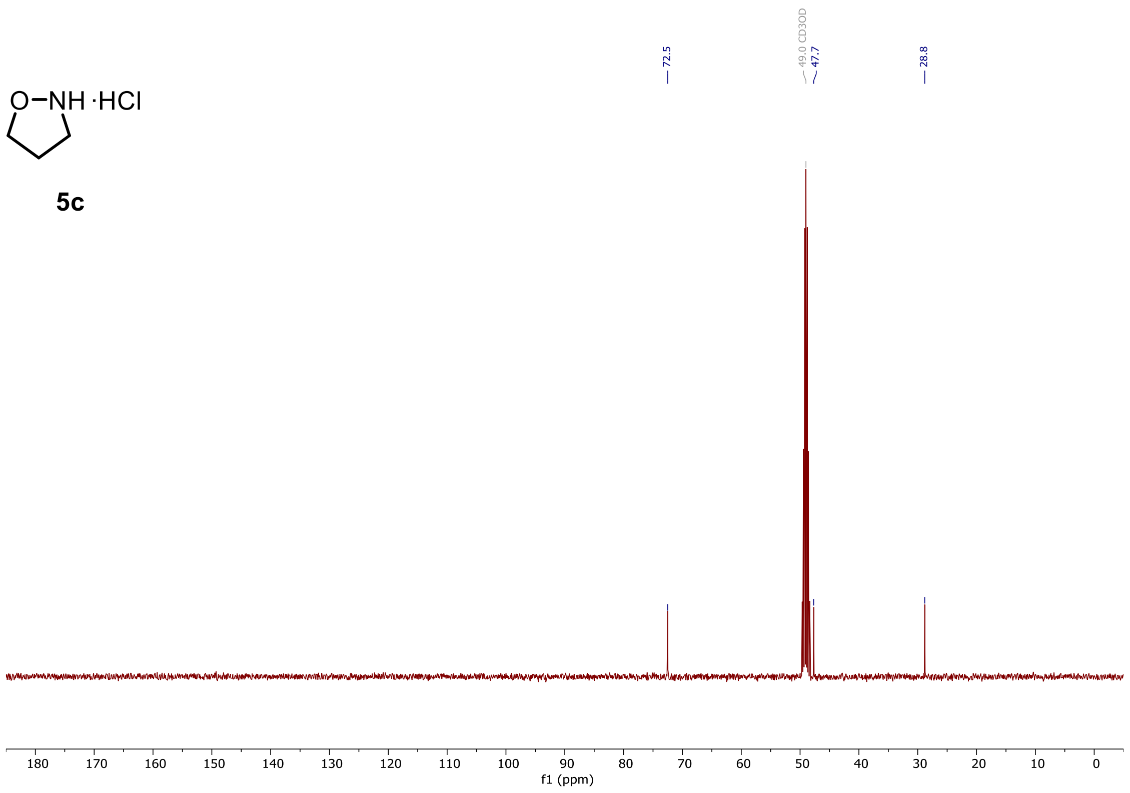

Copy of ^{1}H NMR Spectrum (400 MHz, $CDCl_3$) of **S30**

S30

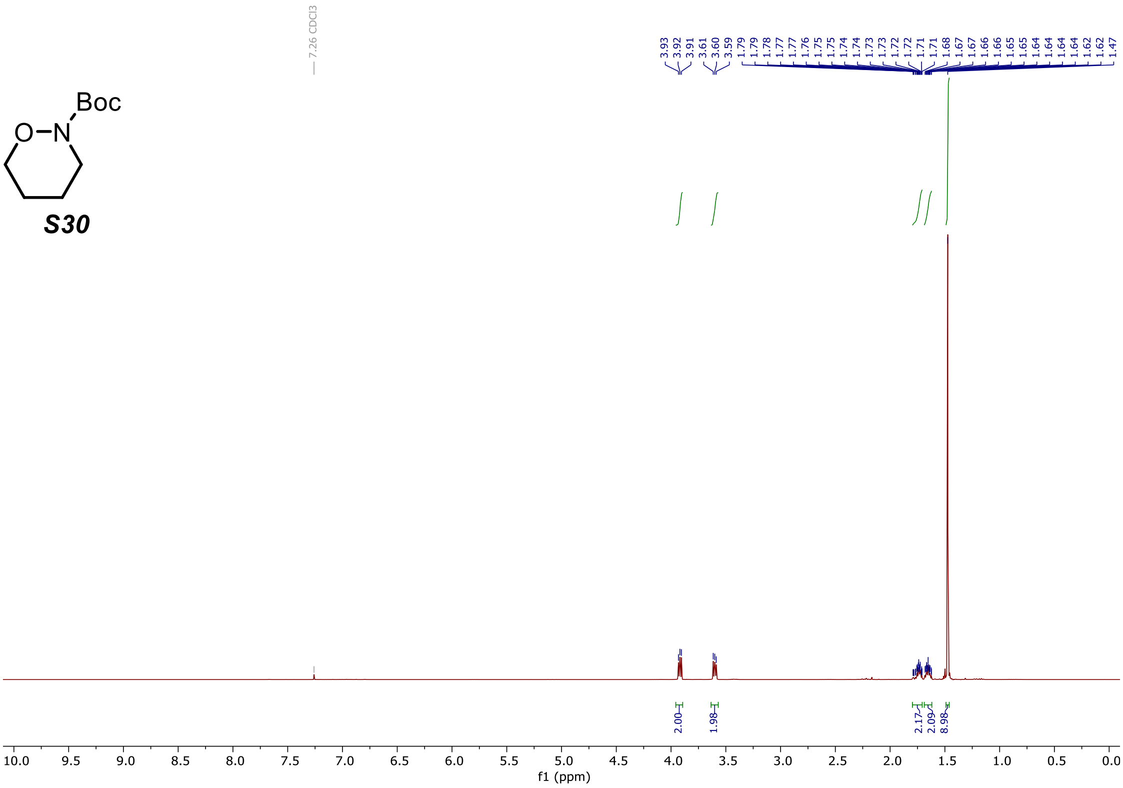

Copy of ^{13}C {^{1}H} NMR Spectrum (101 MHz, $CDCl_3$) of **S30**

S30

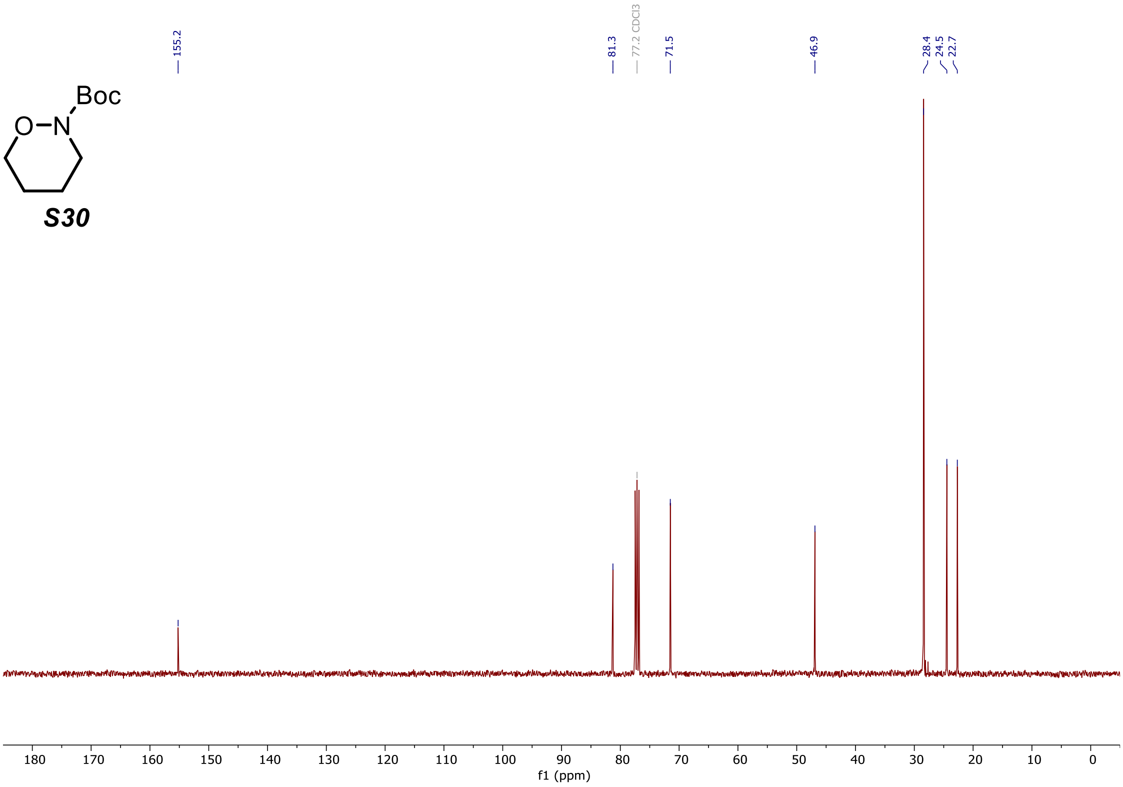

Copy of ^{1}H NMR Spectrum (400 MHz, CD_3OD) of **5d**

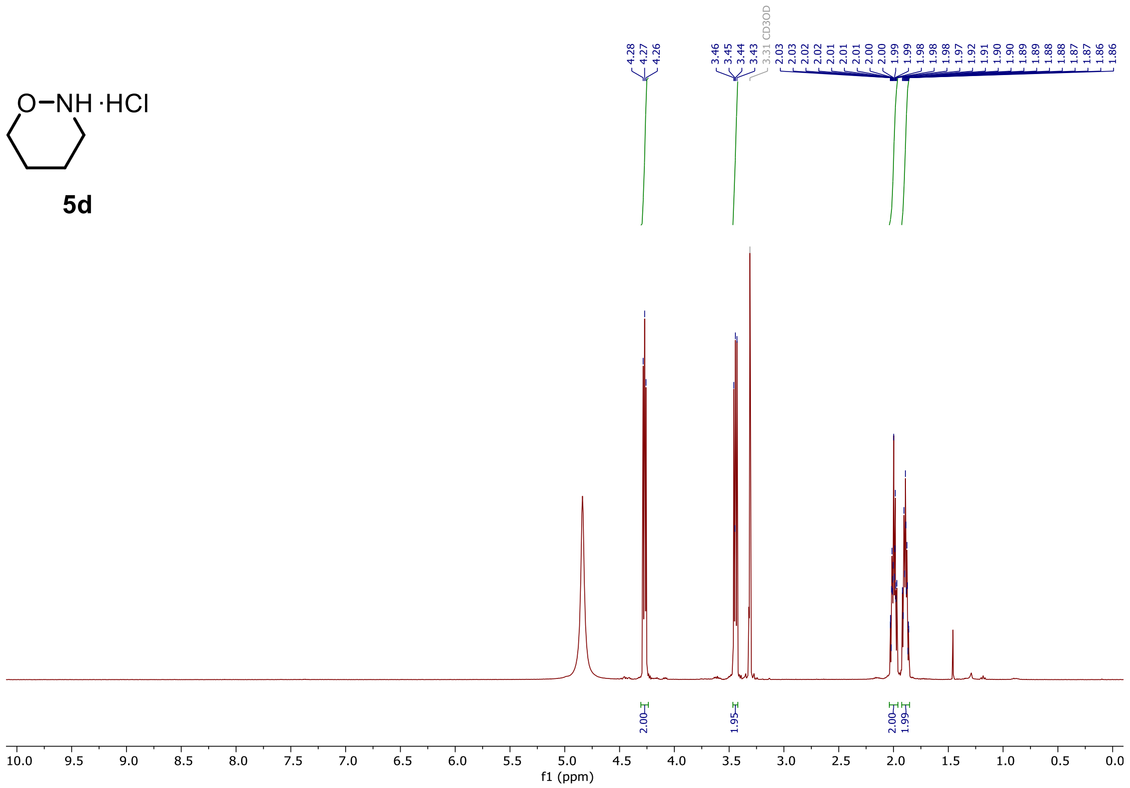

Copy of ^{13}C {^{1}H} NMR Spectrum (101 MHz, CD_3OD) of **5d**

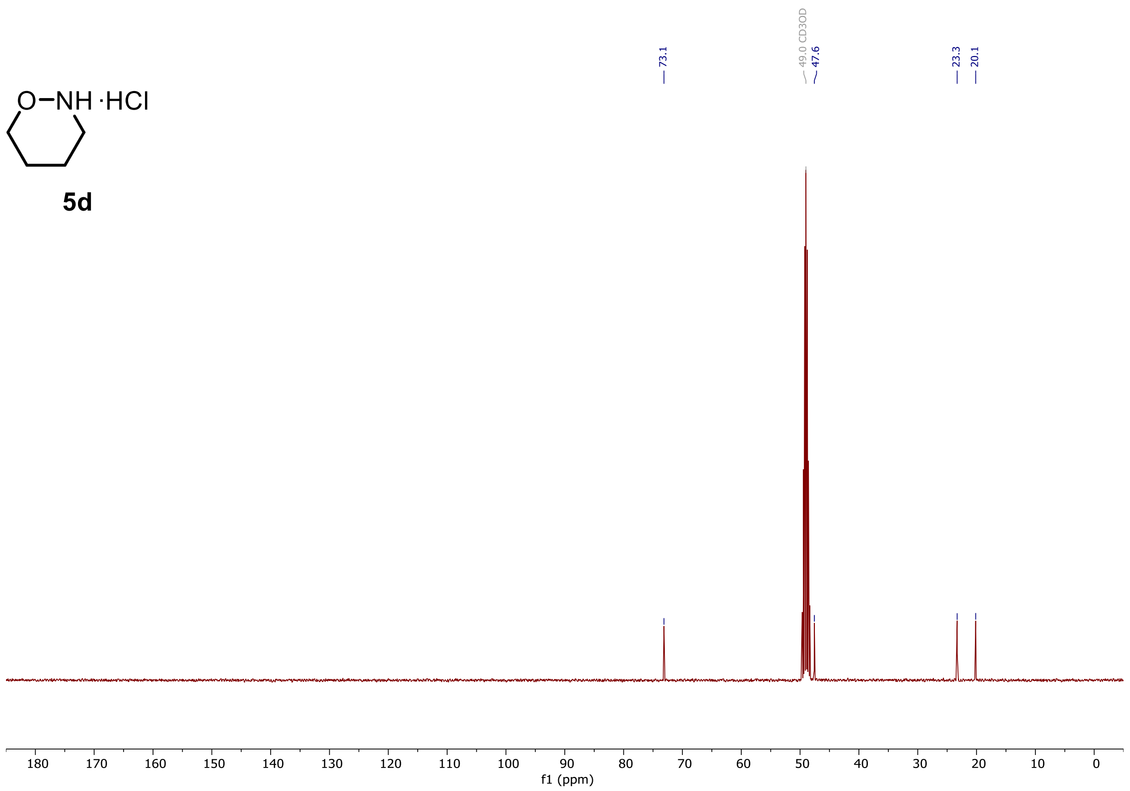

Copy of ^{1}H NMR Spectrum (400 MHz, $CDCl_3$) of **5e**

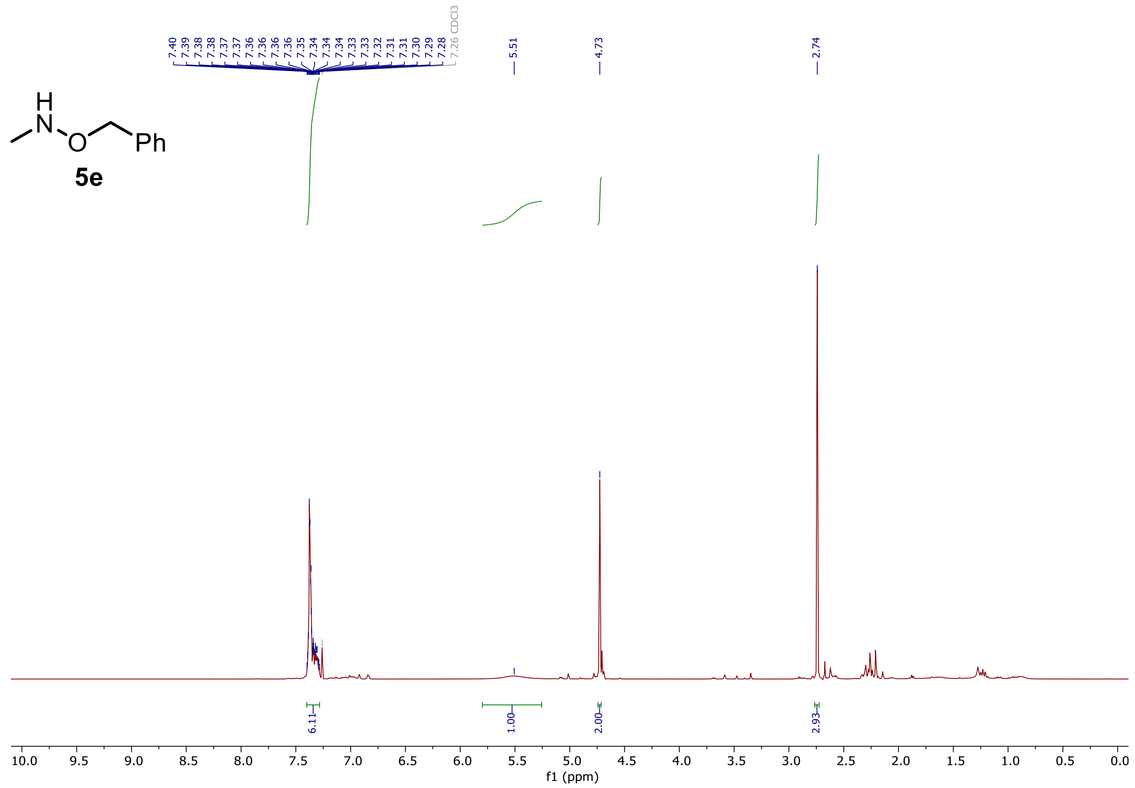

Copy of ^{13}C {^{1}H} NMR Spectrum (101 MHz, $CDCl_3$) of **5e**

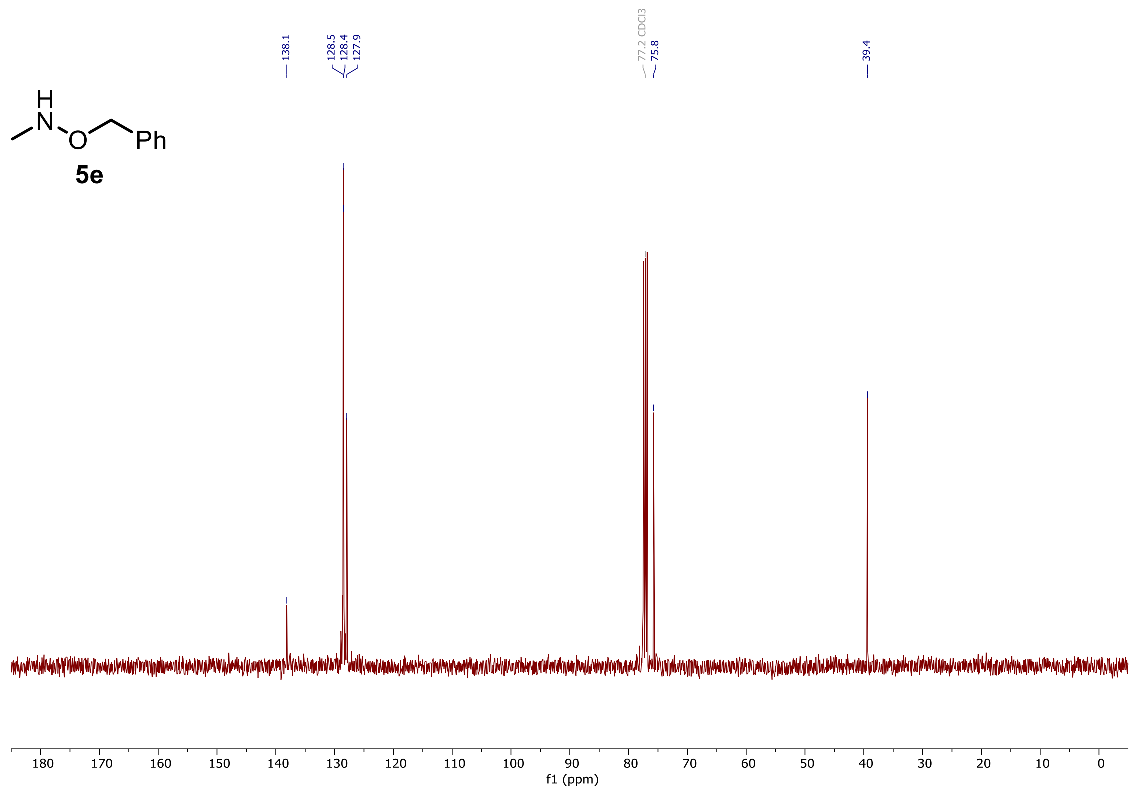

Copy of ^{1}H NMR Spectrum (500 MHz, $CDCl_3$) of **S31**

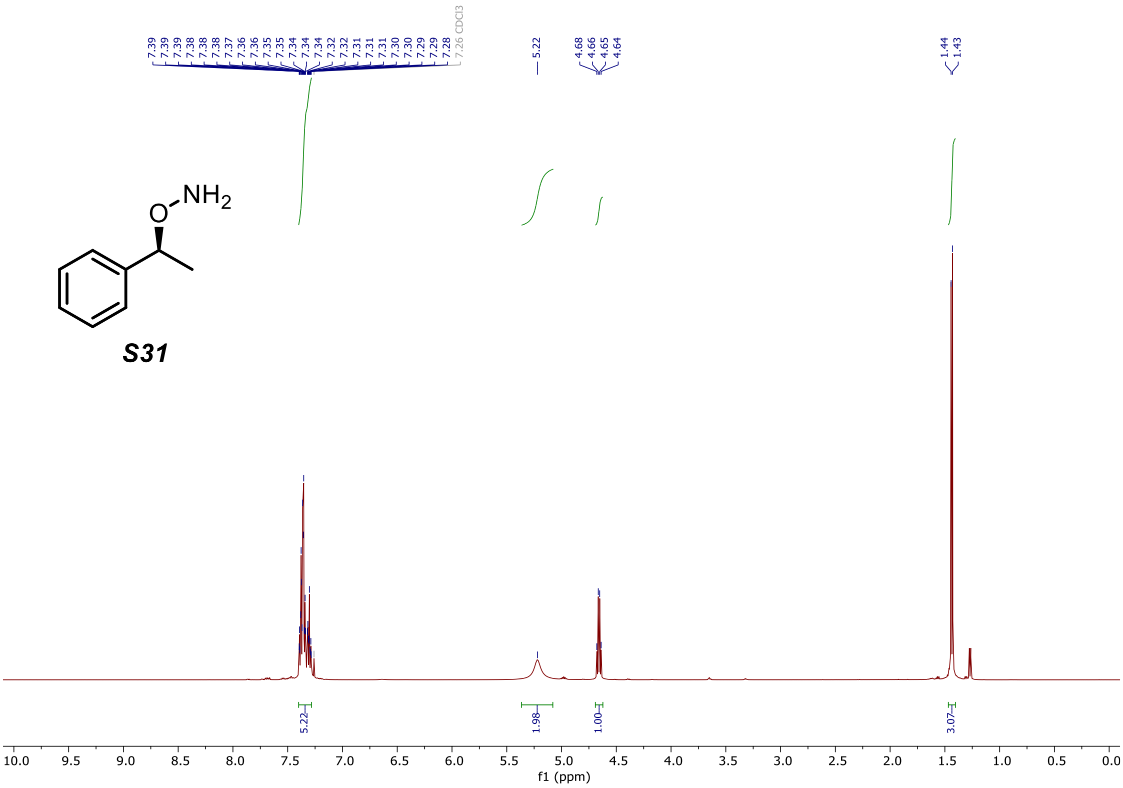

Copy of ^{13}C {^{1}H} NMR Spectrum (126 MHz, $CDCl_3$) of **S31**

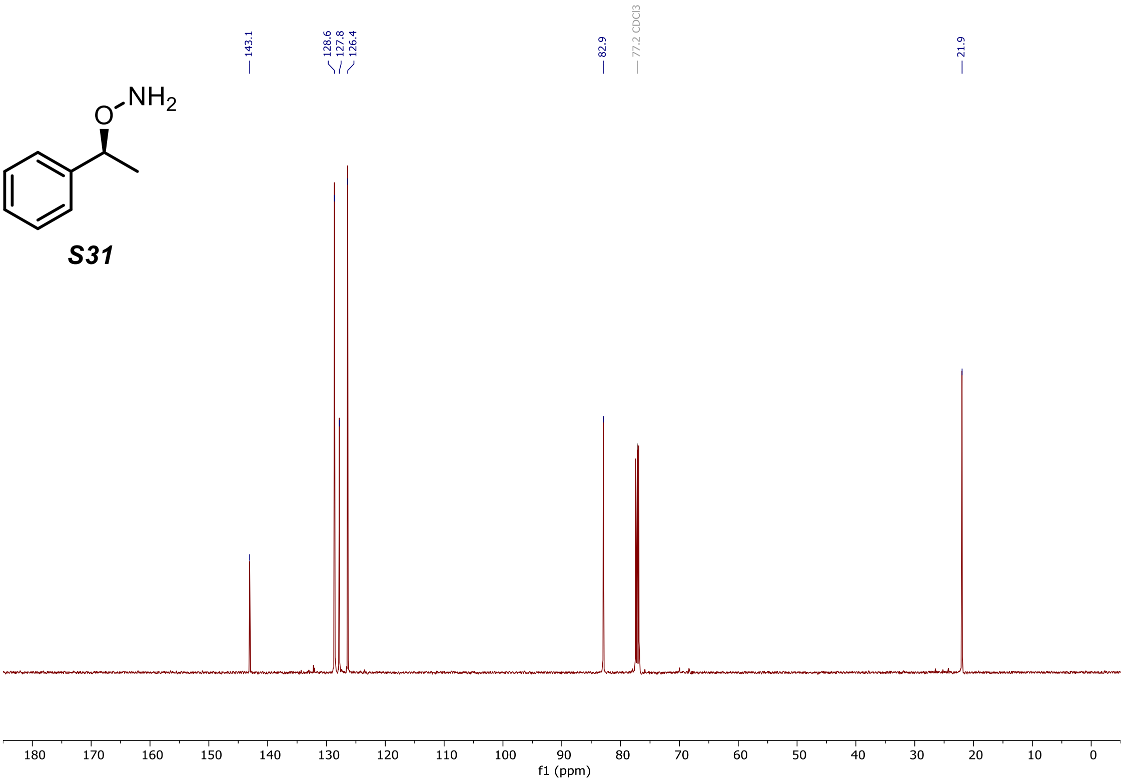

Copy of ¹H NMR Spectrum (500 MHz, $CDCl_3$) of **S32**

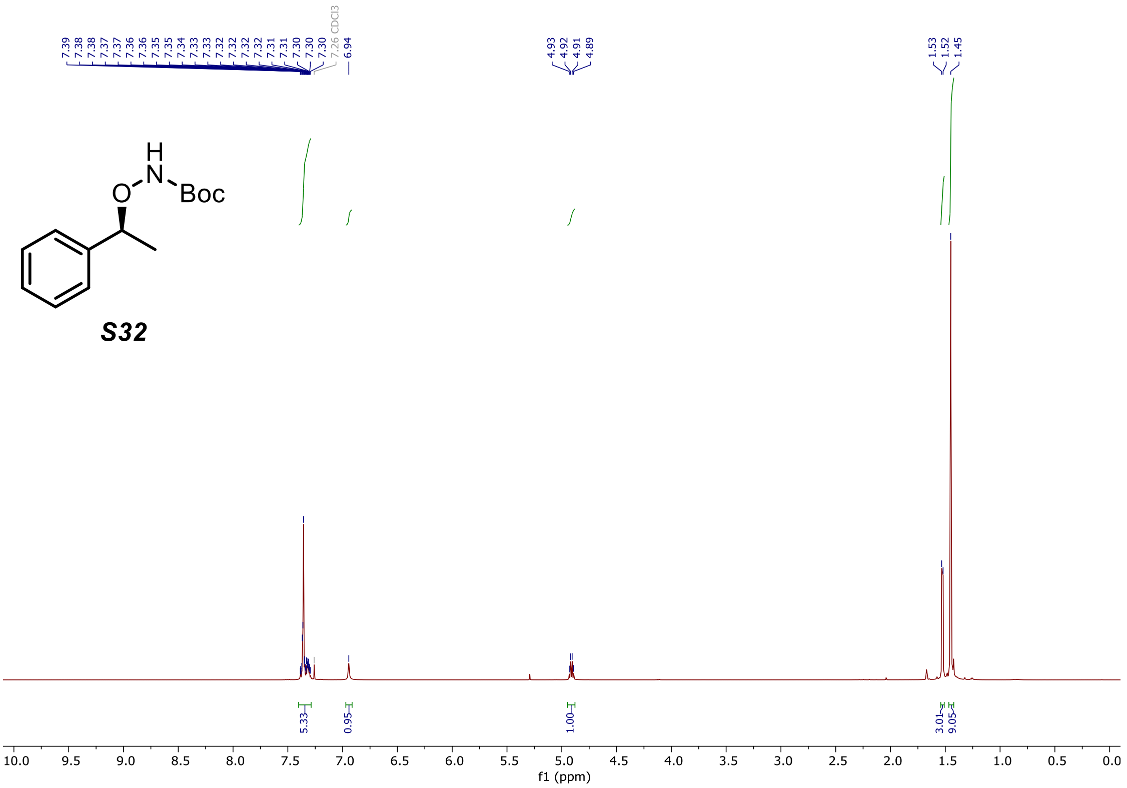

Copy of ¹³C {¹H} NMR Spectrum (126 MHz, $CDCl_3$) of **S32**

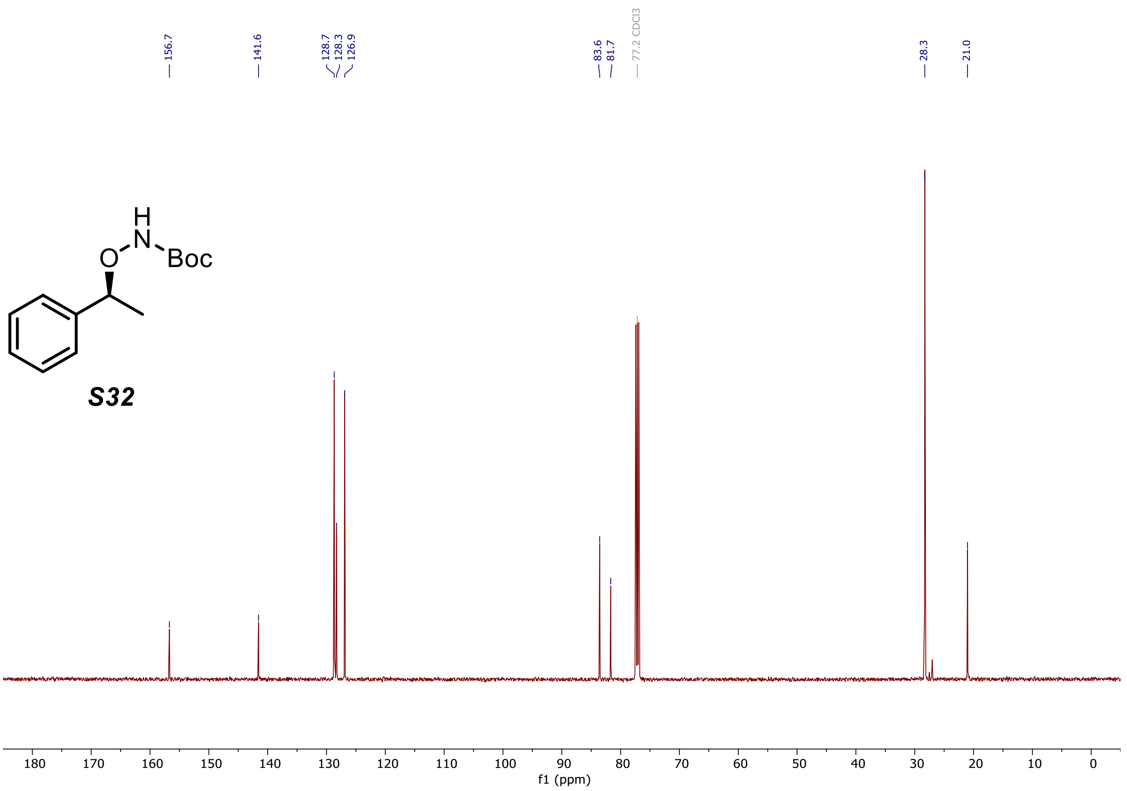

Copy of ^{1}H NMR Spectrum (500 MHz, $CDCl_3$) of **S33**

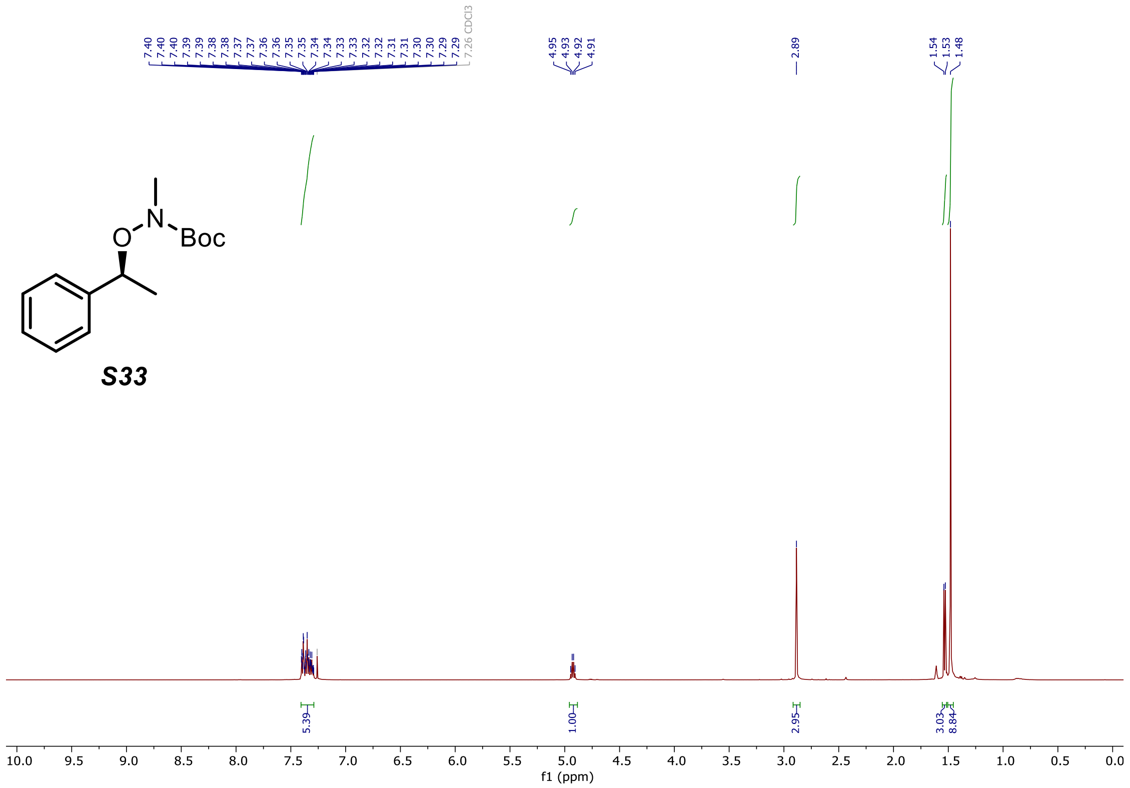

Copy of ^{13}C {^{1}H} NMR Spectrum (126 MHz, $CDCl_3$) of **S33**

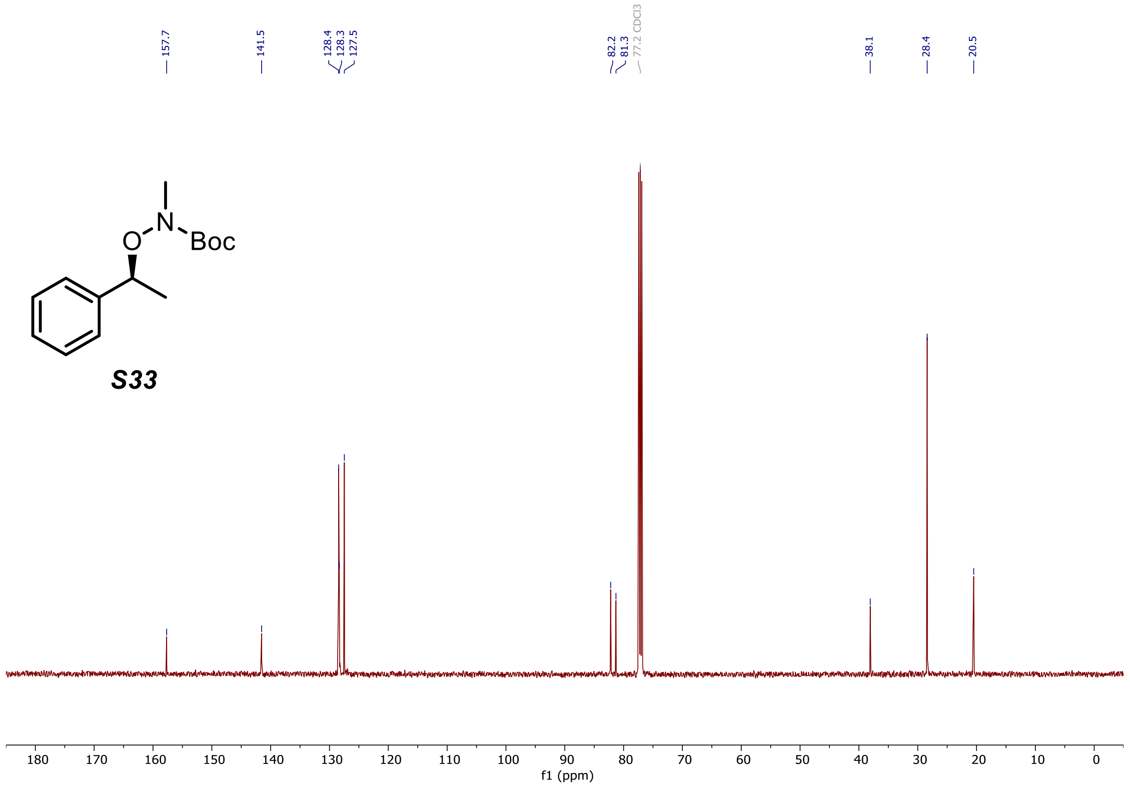

Copy of ^{1}H NMR Spectrum (500 MHz, $CDCl_3$) of **5f**

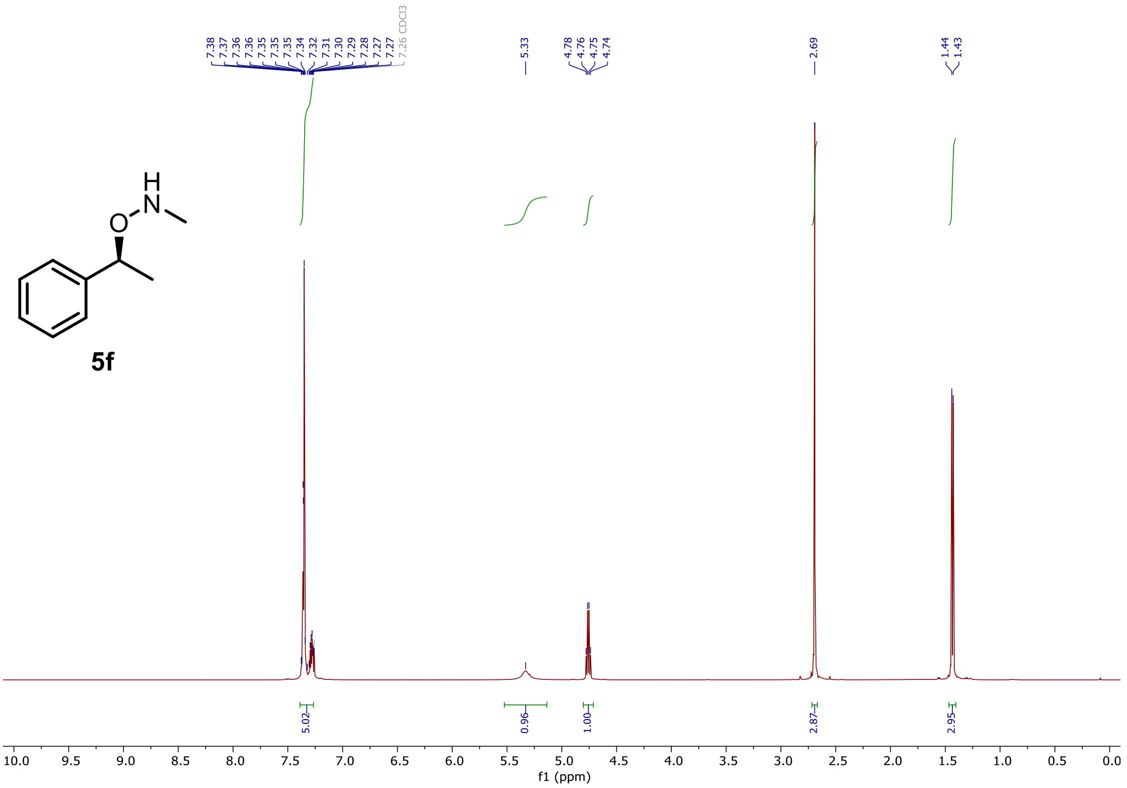

5f

Copy of ^{13}C {^{1}H} NMR Spectrum (126 MHz, $CDCl_3$) of **5f**

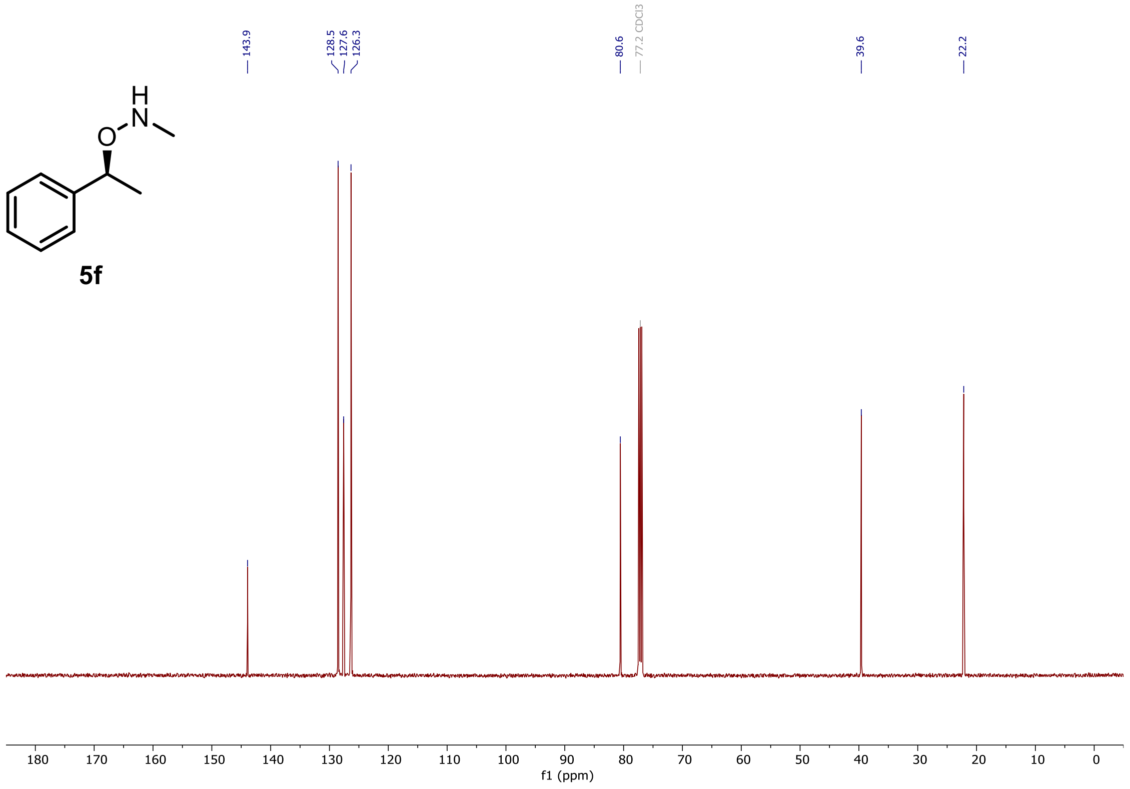

5f

Copy of ^{1}H NMR Spectrum (400 MHz, CD_3OD) of **S34**

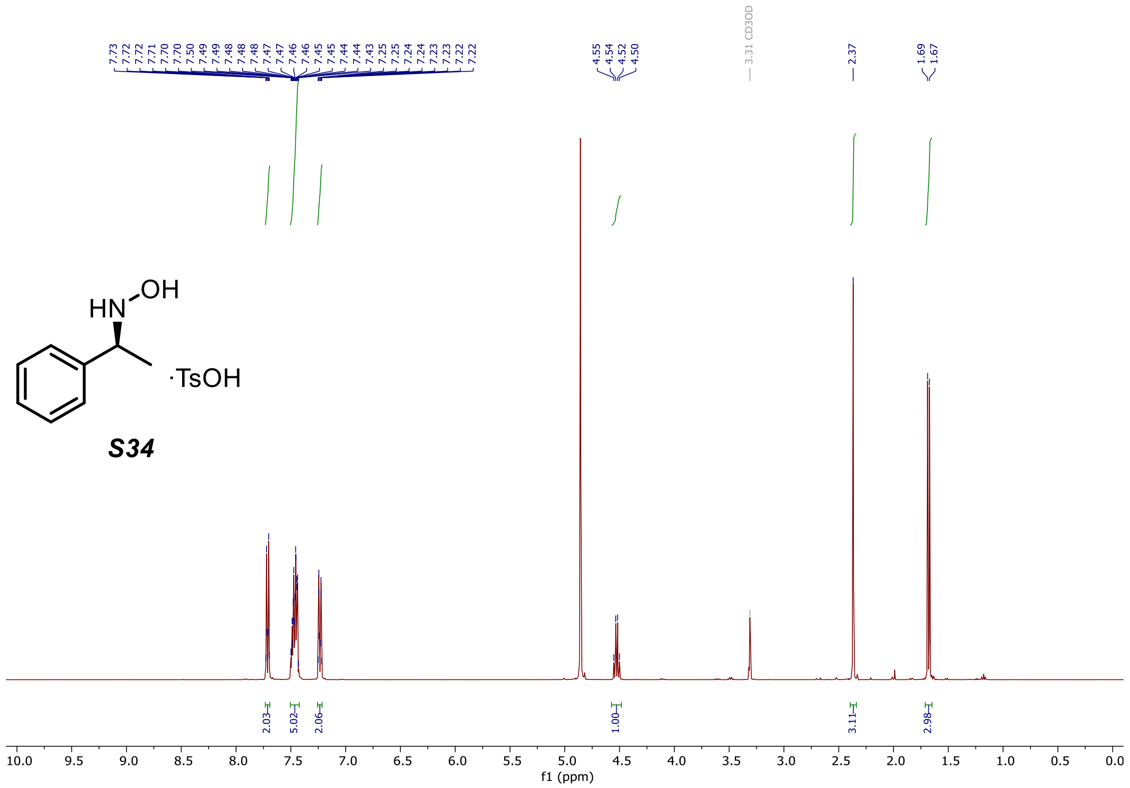

Copy of ^{13}C {^{1}H} NMR Spectrum (101 MHz, CD_3OD) of **S34**

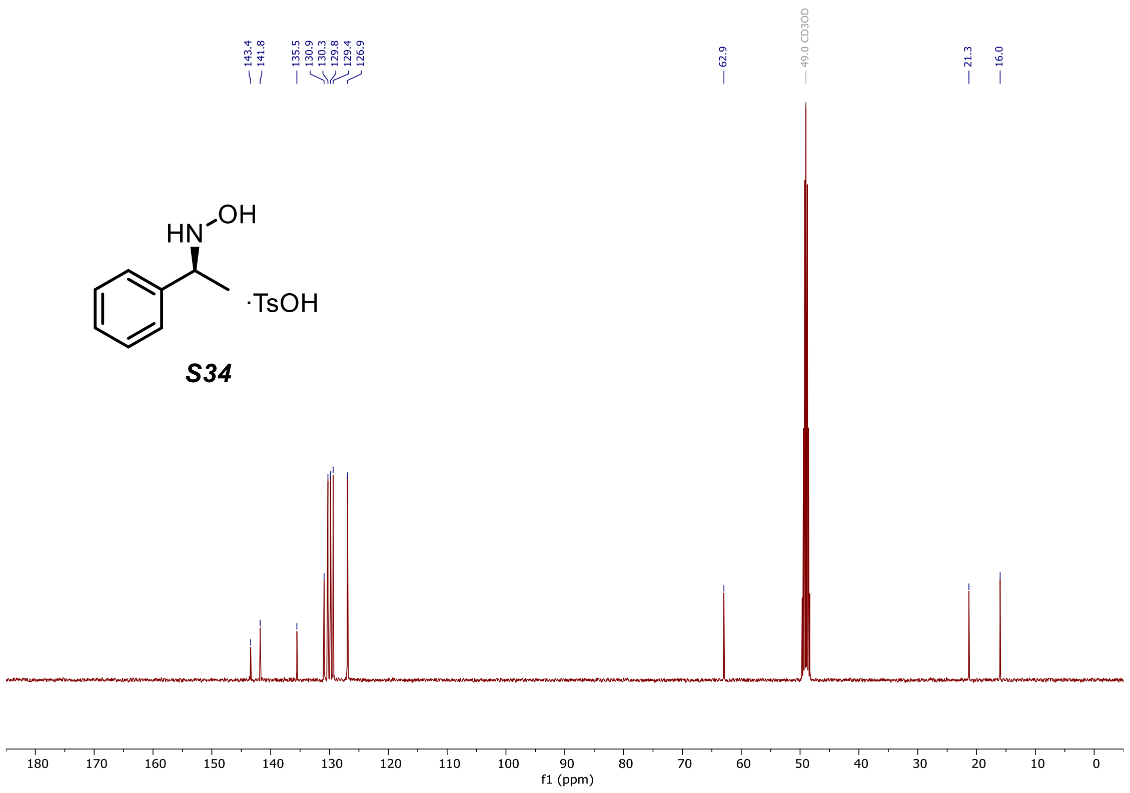

Copy of ^{1}H NMR Spectrum (400 MHz, DMSO-d_6) of **S35**

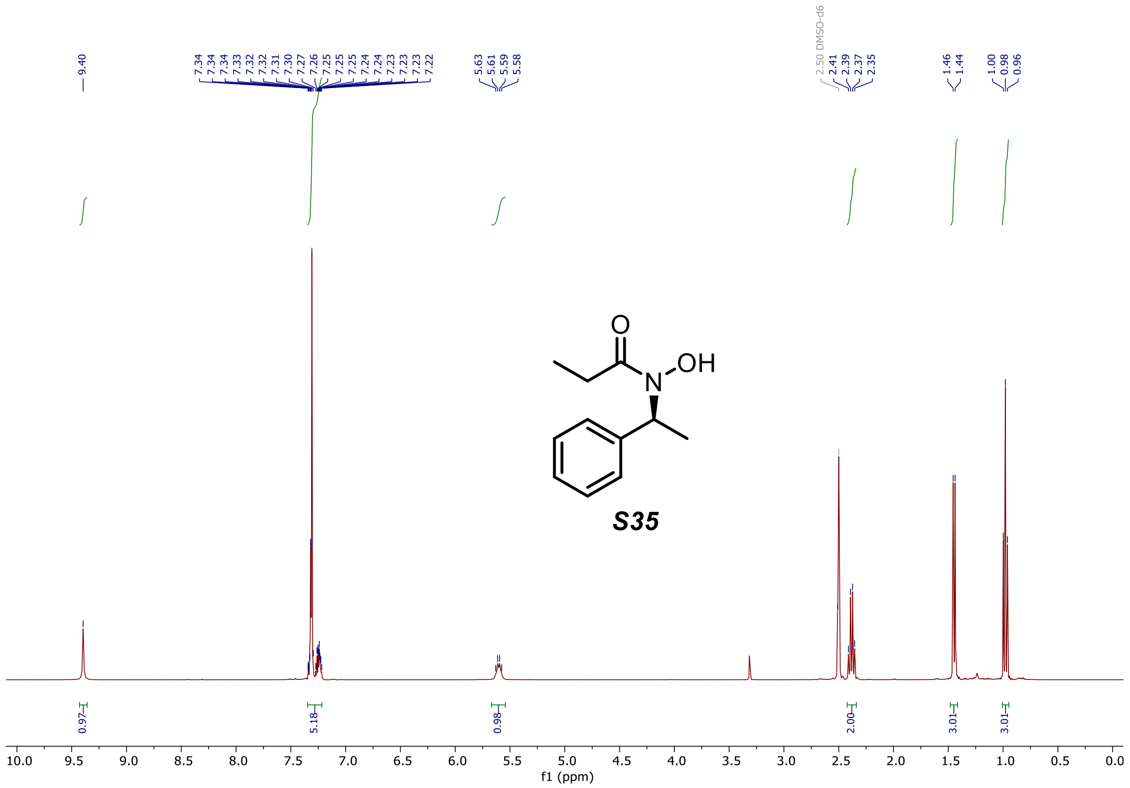

Copy of ^{13}C {^{1}H} NMR Spectrum (101 MHz, DMSO-d_6) of **S35**

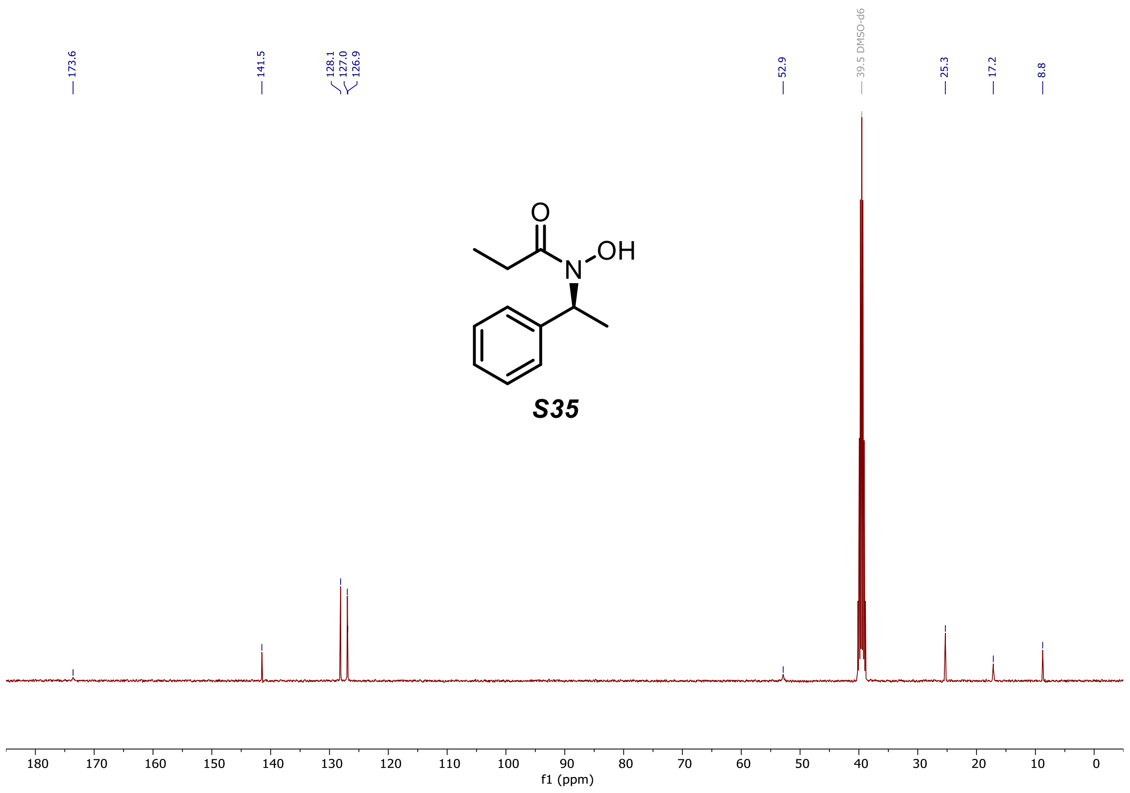

Copy of ^{1}H NMR Spectrum (400 MHz, $CDCl_3$) of **S36**

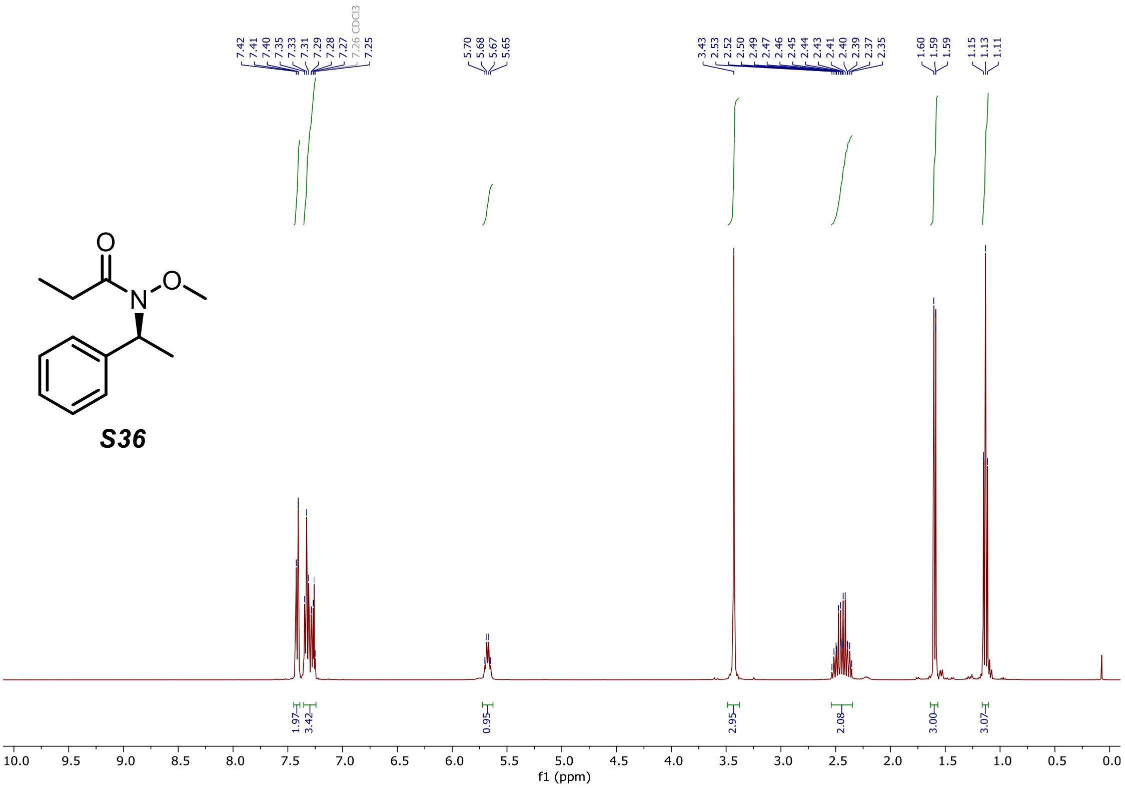

Copy of ^{13}C {^{1}H} NMR Spectrum (101 MHz, $CDCl_3$) of **S36**

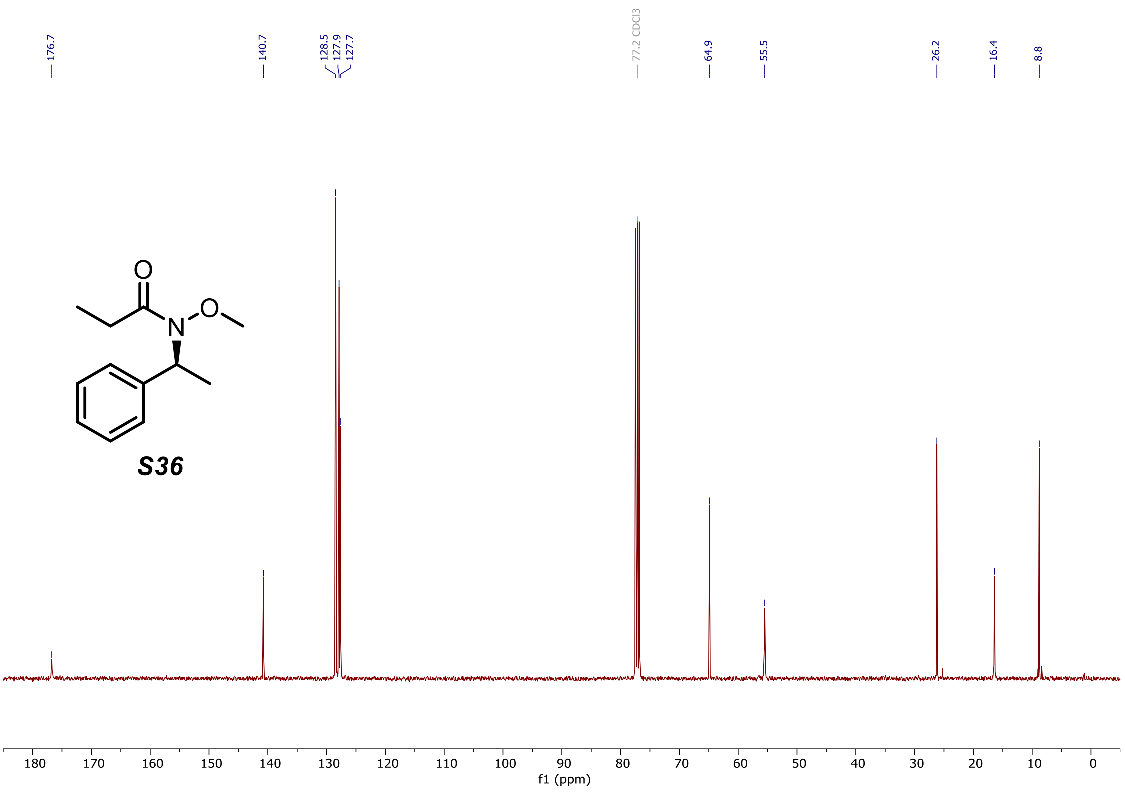

Copy of ^{1}H NMR Spectrum (500 MHz, $CDCl_3$) of **5g**

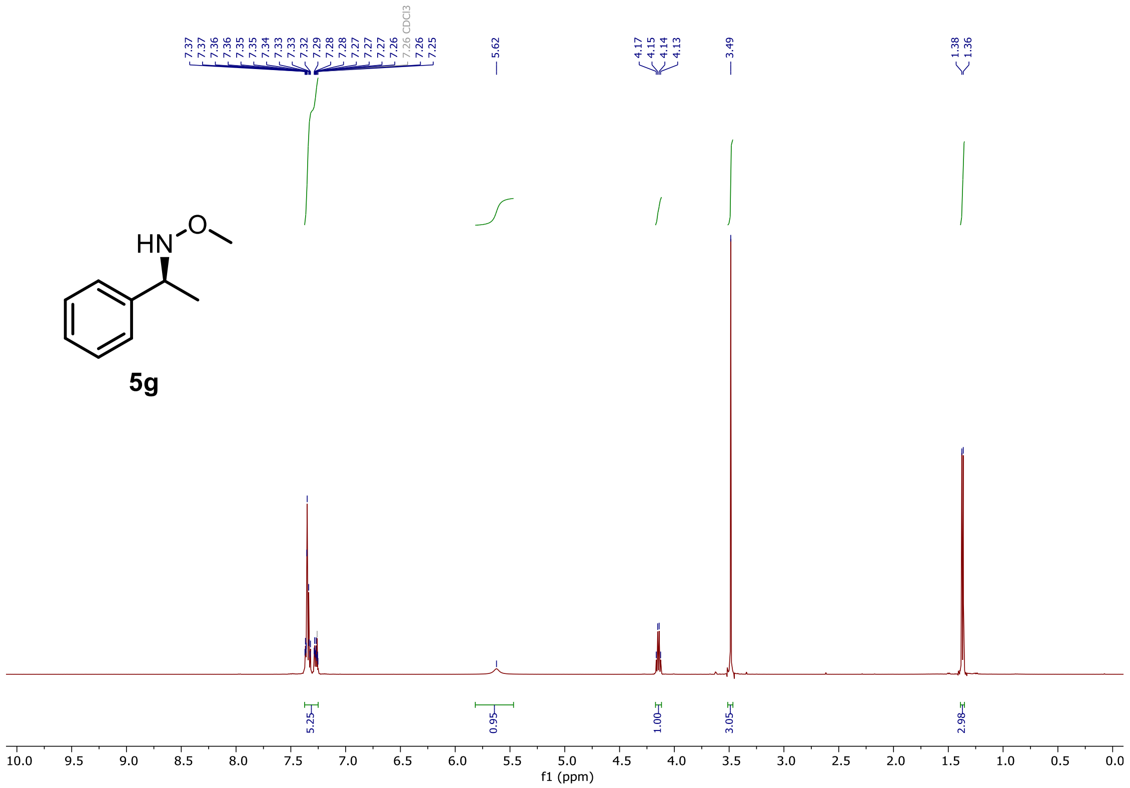

Copy of ^{13}C {^{1}H} NMR Spectrum (126 MHz, $CDCl_3$) of **5g**

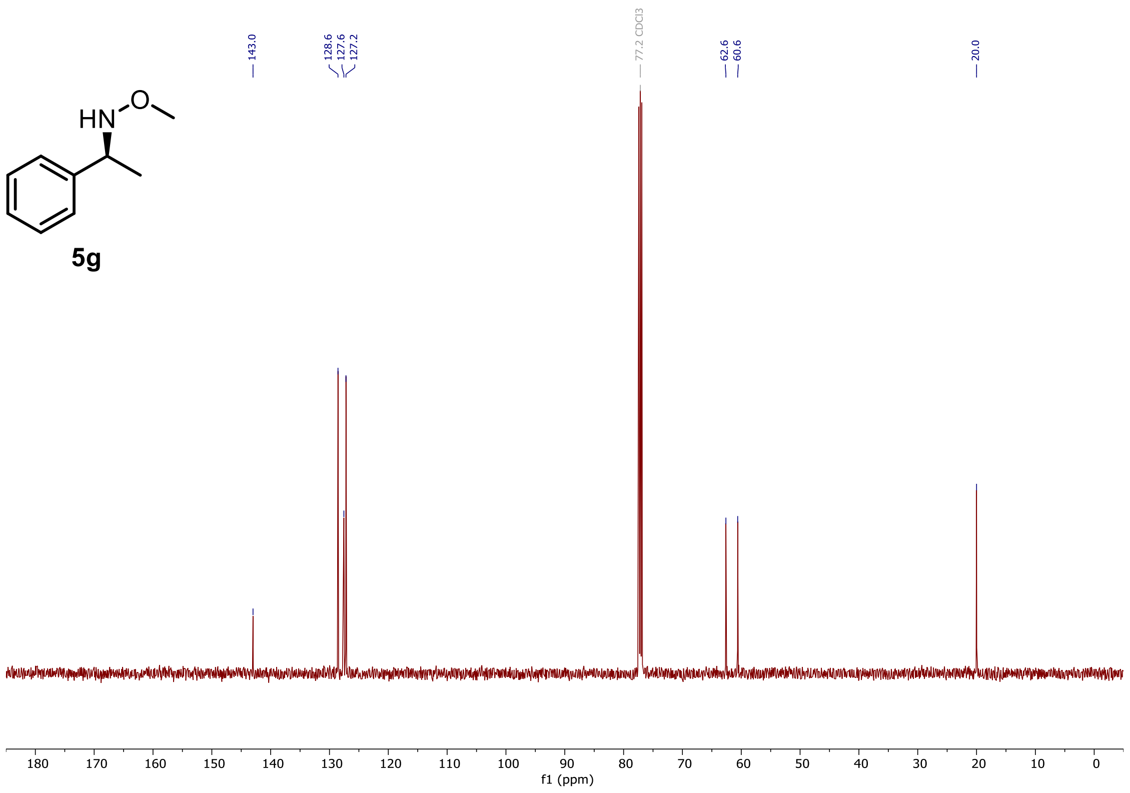

Copy of ^{1}H NMR Spectrum (400 MHz, $CDCl_3$) of **5h**

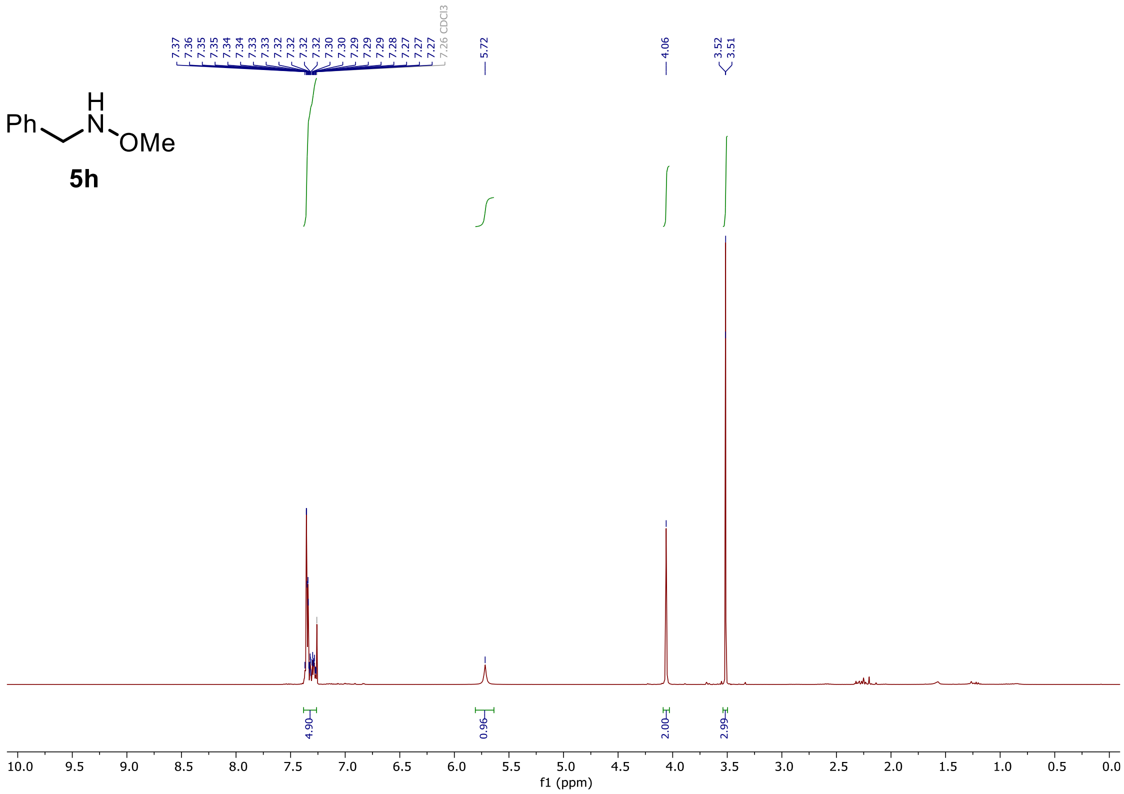

Copy of ^{13}C {^{1}H} NMR Spectrum (101 MHz, $CDCl_3$) of **5h**

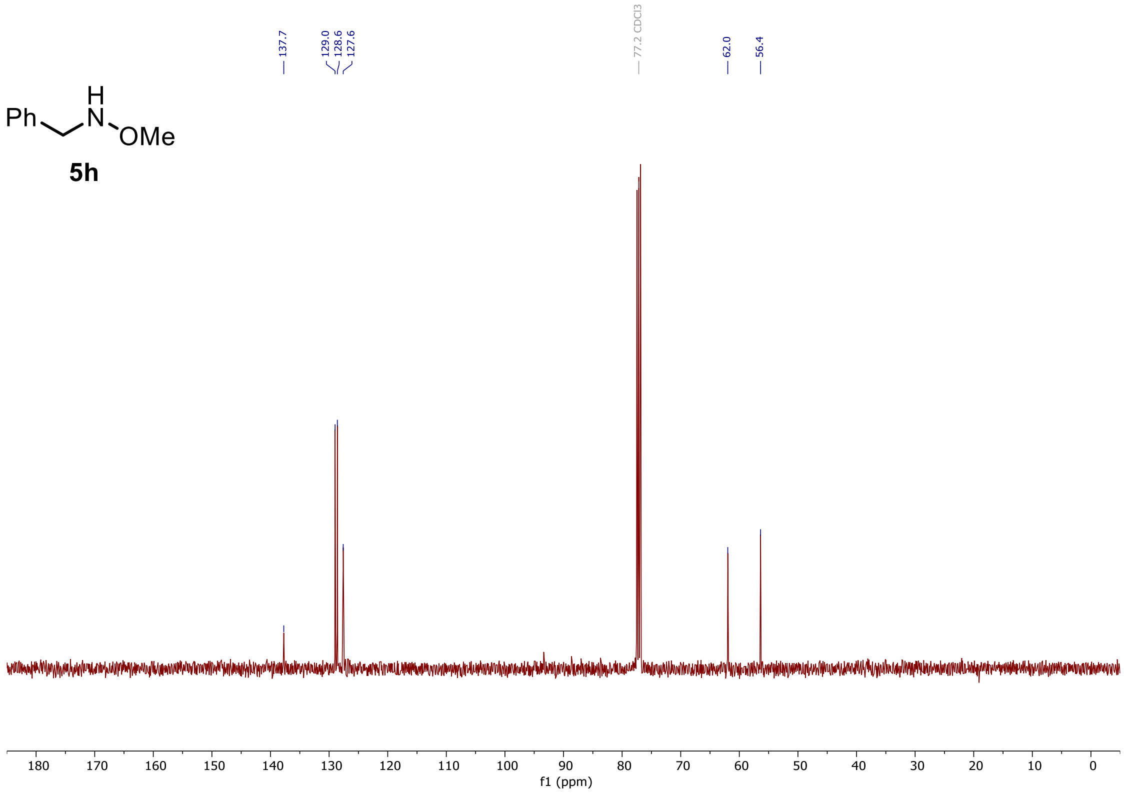

Copy of ^{1}H NMR Spectrum (400 MHz, $CDCl_3$) of **5i**

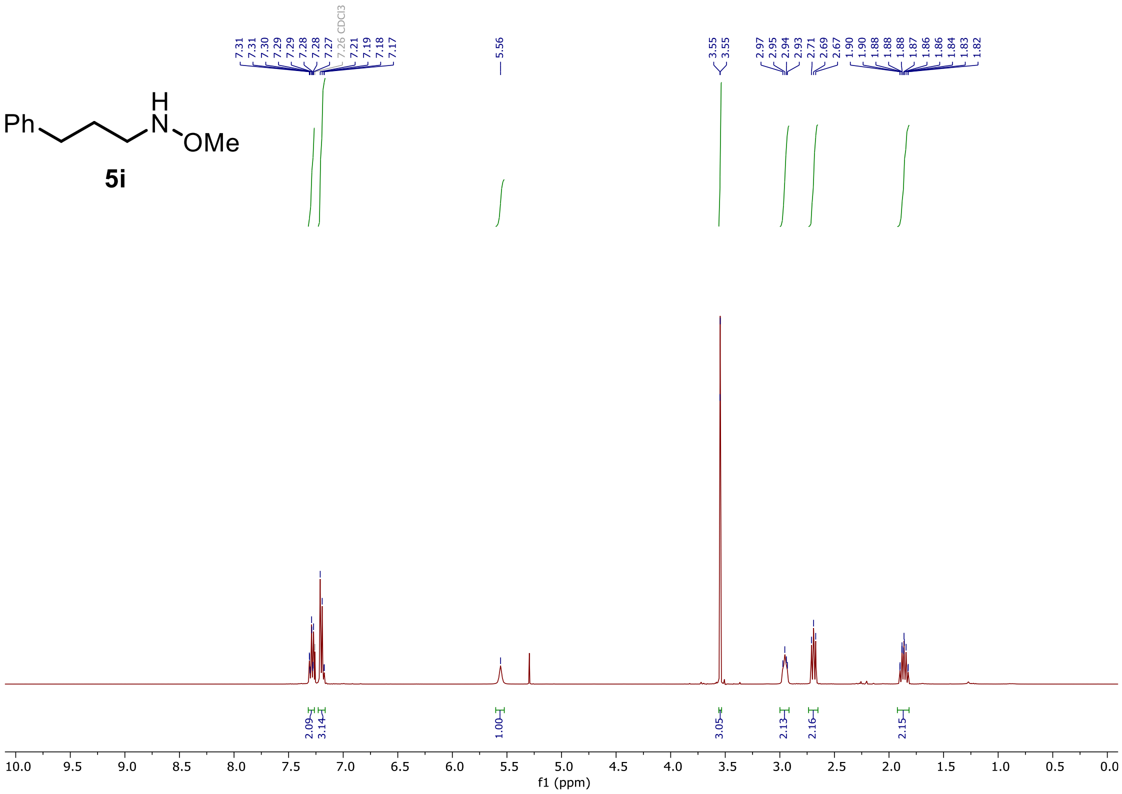

Copy of ^{13}C {^{1}H} NMR Spectrum (101 MHz, $CDCl_3$) of **5i**

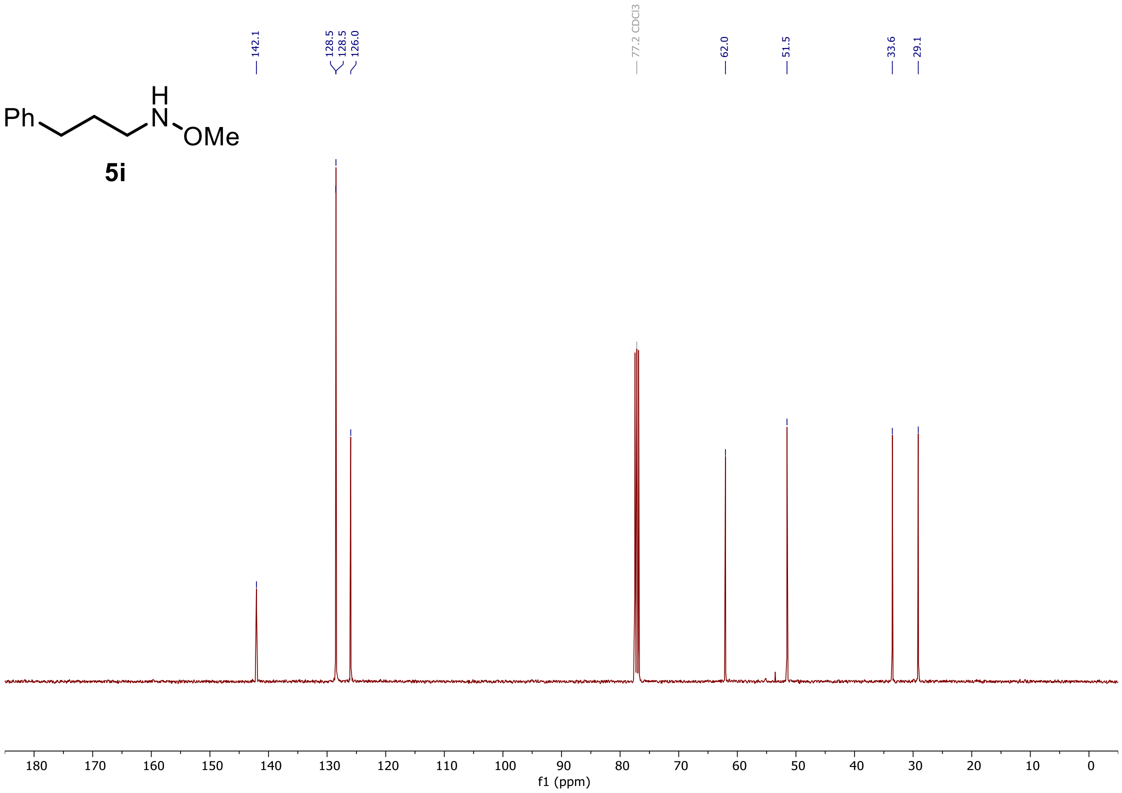

Copy of ^{1}H NMR Spectrum (400 MHz, $CDCl_3$) of **5j**

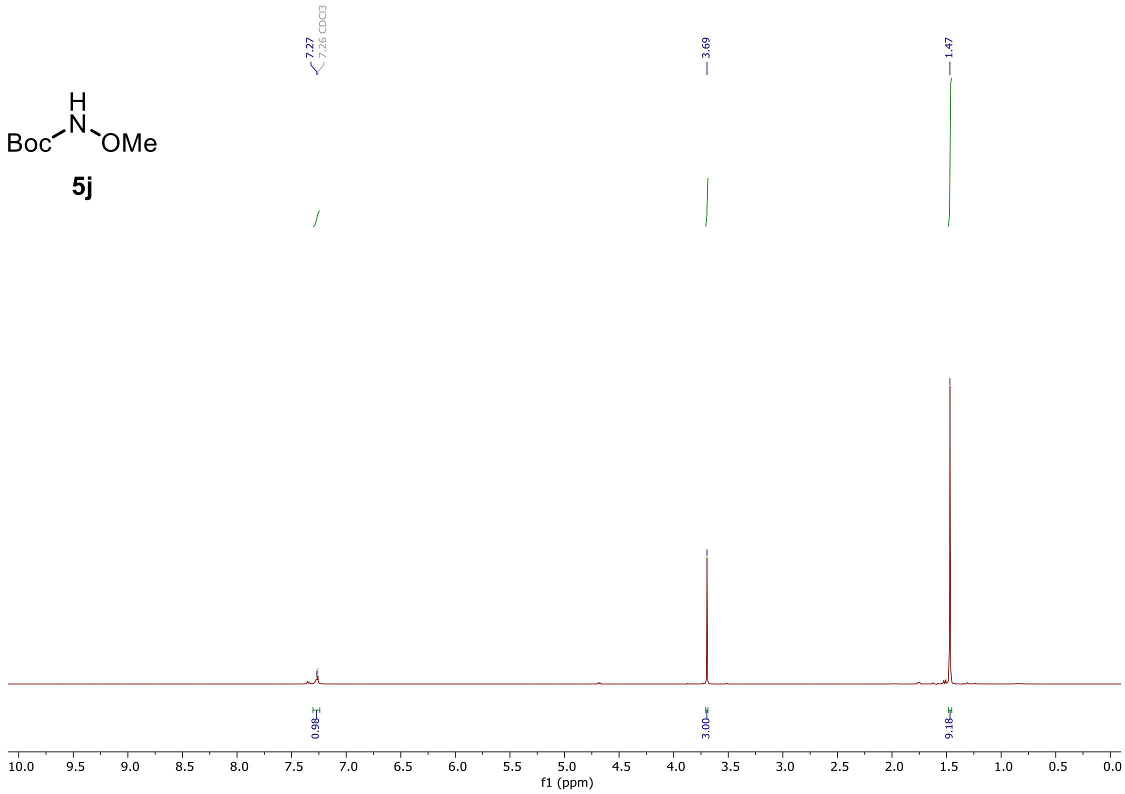

Copy of ^{13}C {^{1}H} NMR Spectrum (101 MHz, $CDCl_3$) of **5j**

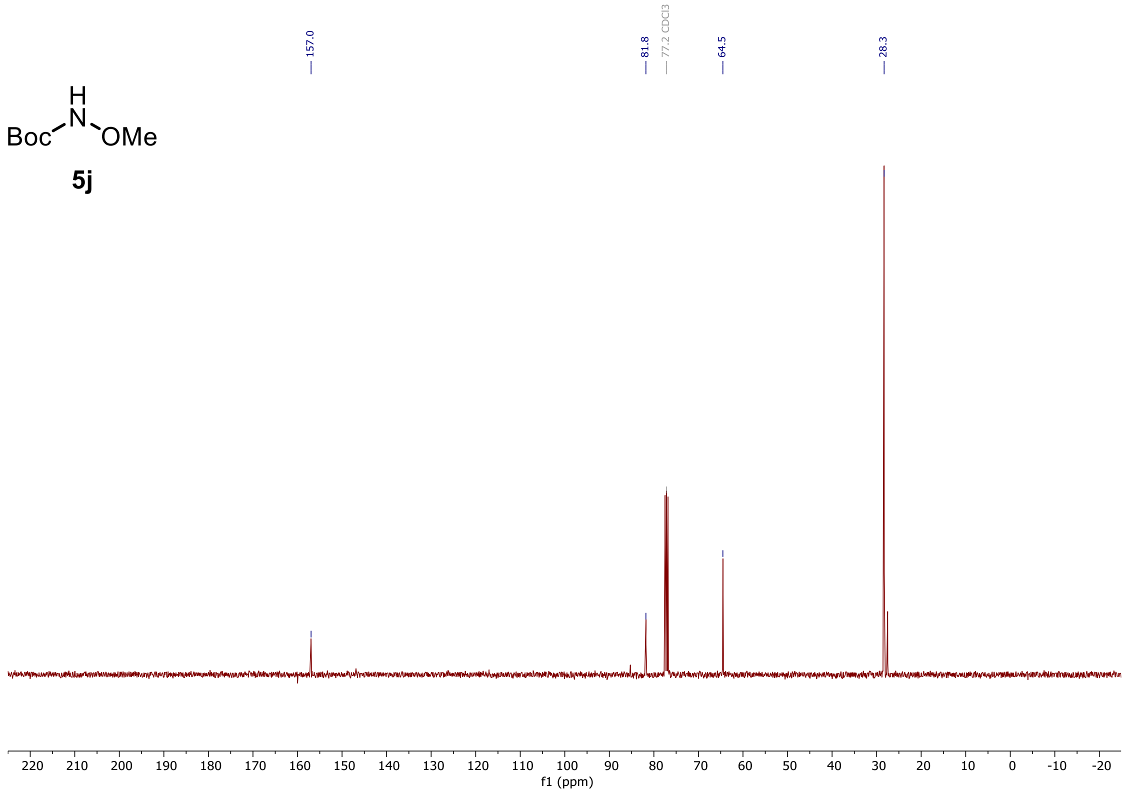

Copy of ^{1}H NMR Spectrum (400 MHz, $CDCl_3$) of **S37**

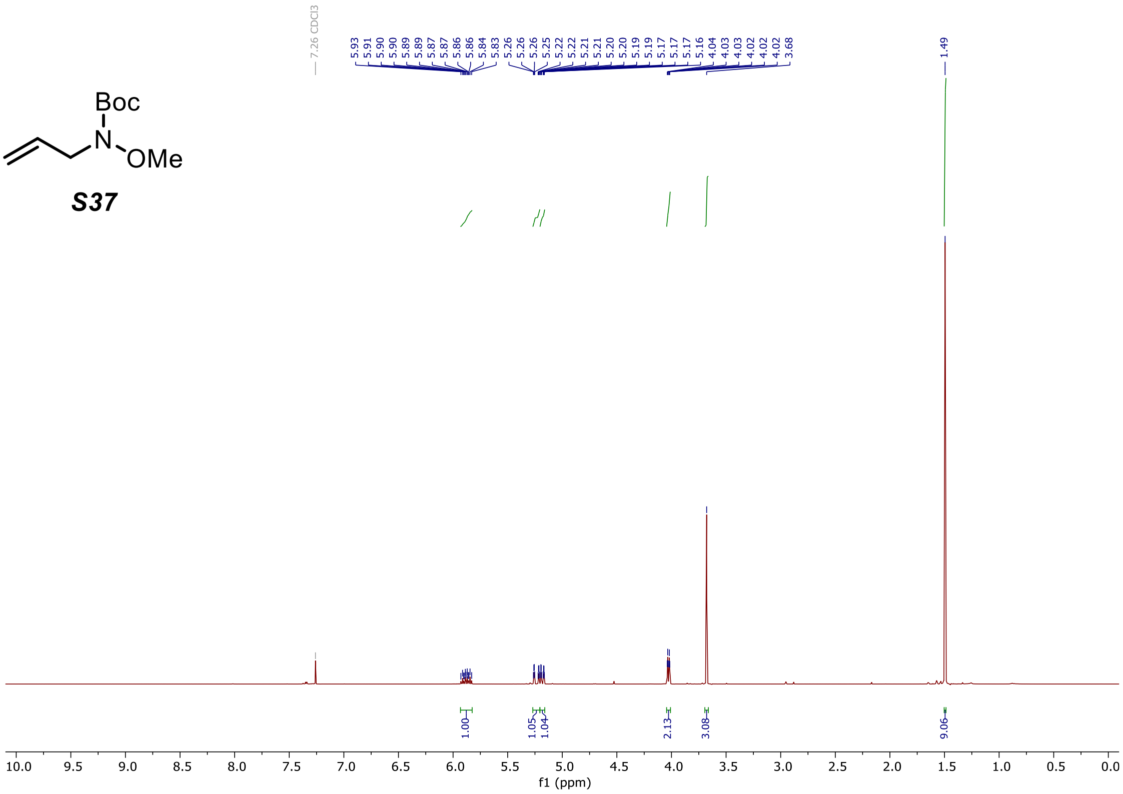

Copy of ^{13}C {^{1}H} NMR Spectrum (101 MHz, $CDCl_3$) of **S37**

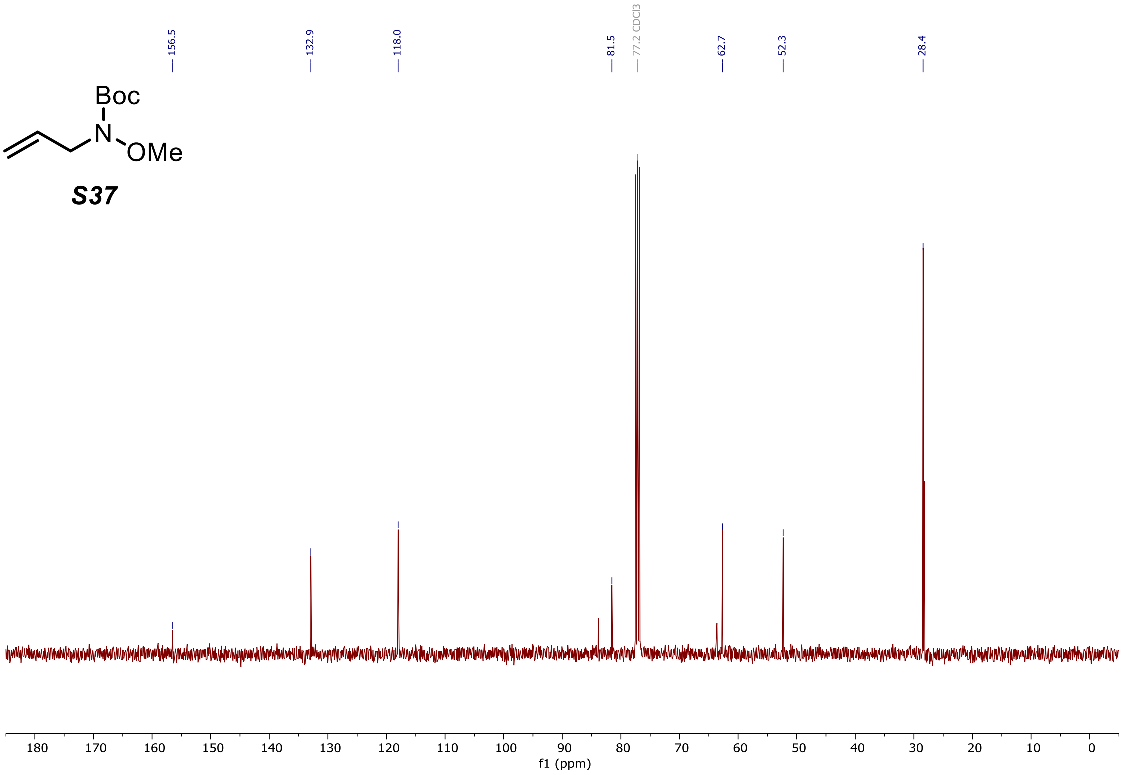

Copy of ^{1}H NMR Spectrum (400 MHz, D_2O) of **5k**

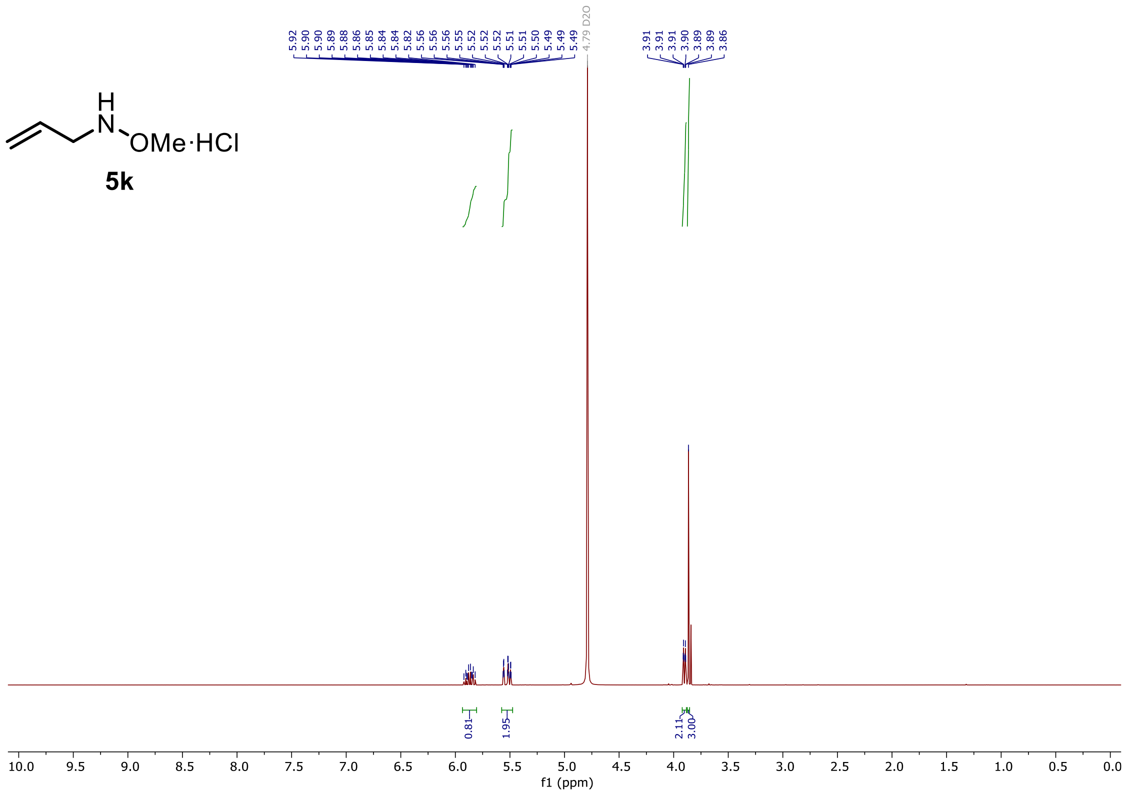

Copy of ^{13}C {^{1}H} NMR Spectrum (101 MHz, D_2O) of **5k**

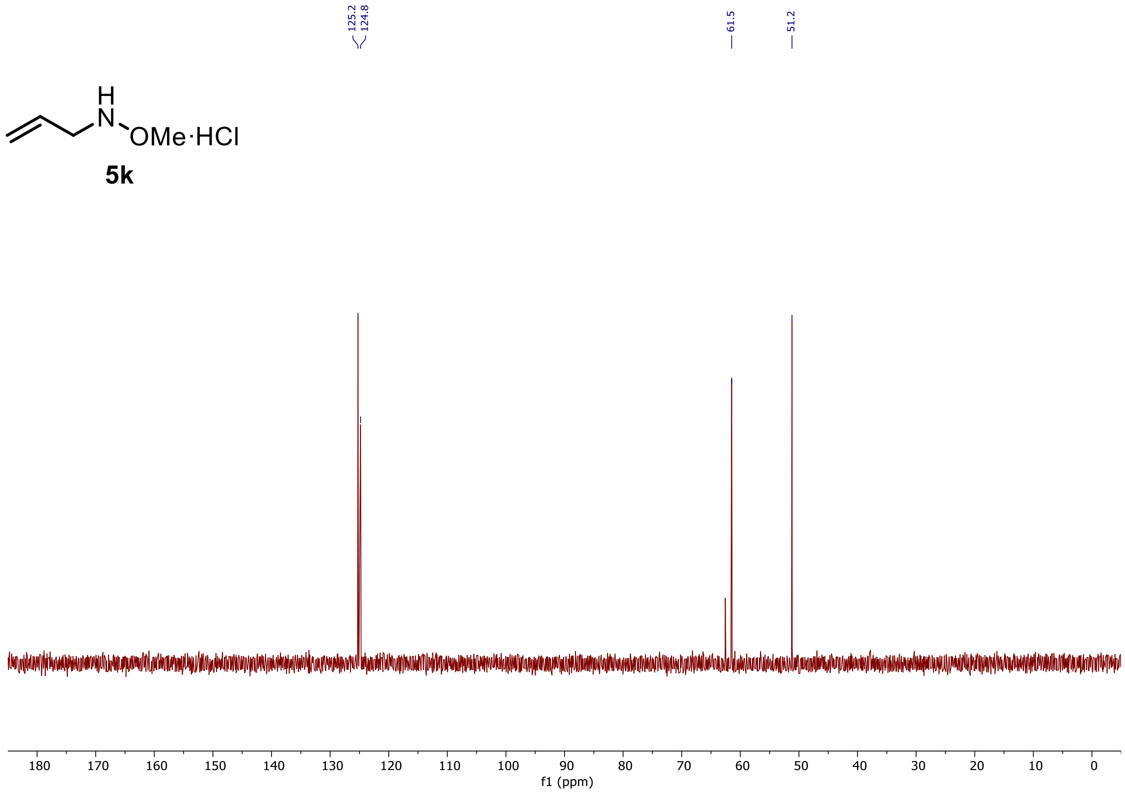

Copy of ^{1}H NMR Spectrum (400 MHz, $CDCl_3$) of **S39**

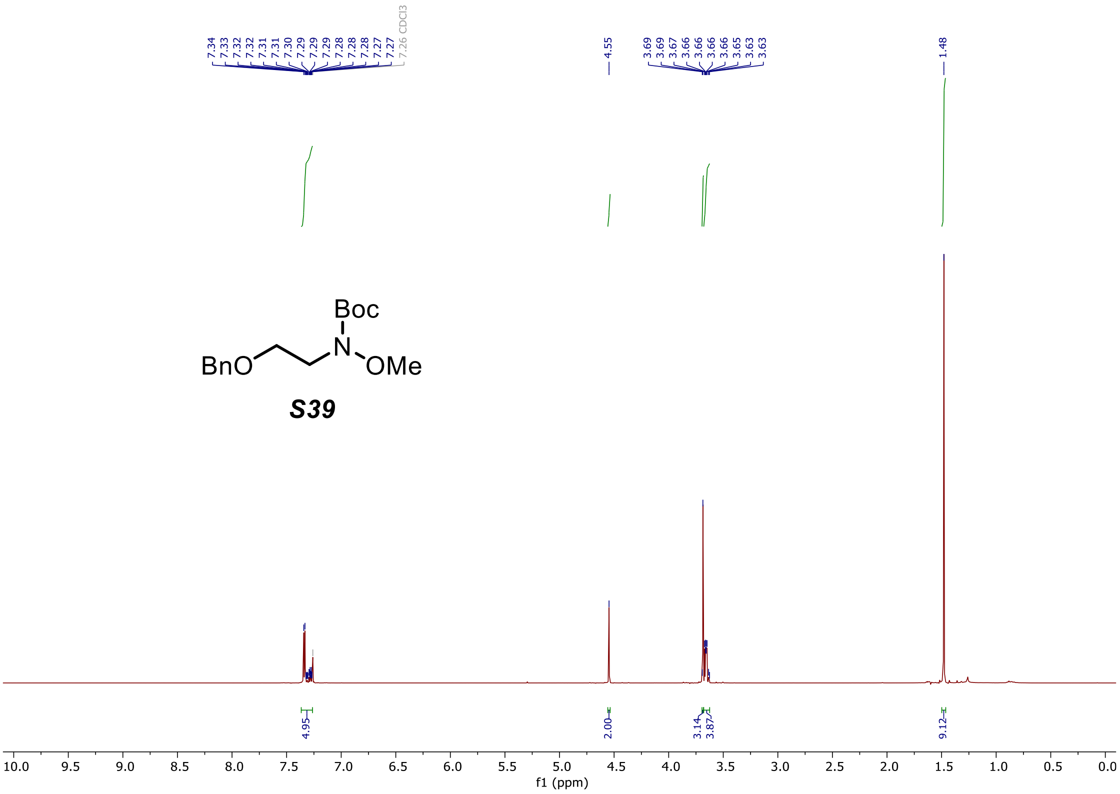

Copy of ^{13}C {^{1}H} NMR Spectrum (101 MHz, $CDCl_3$) of **S39**

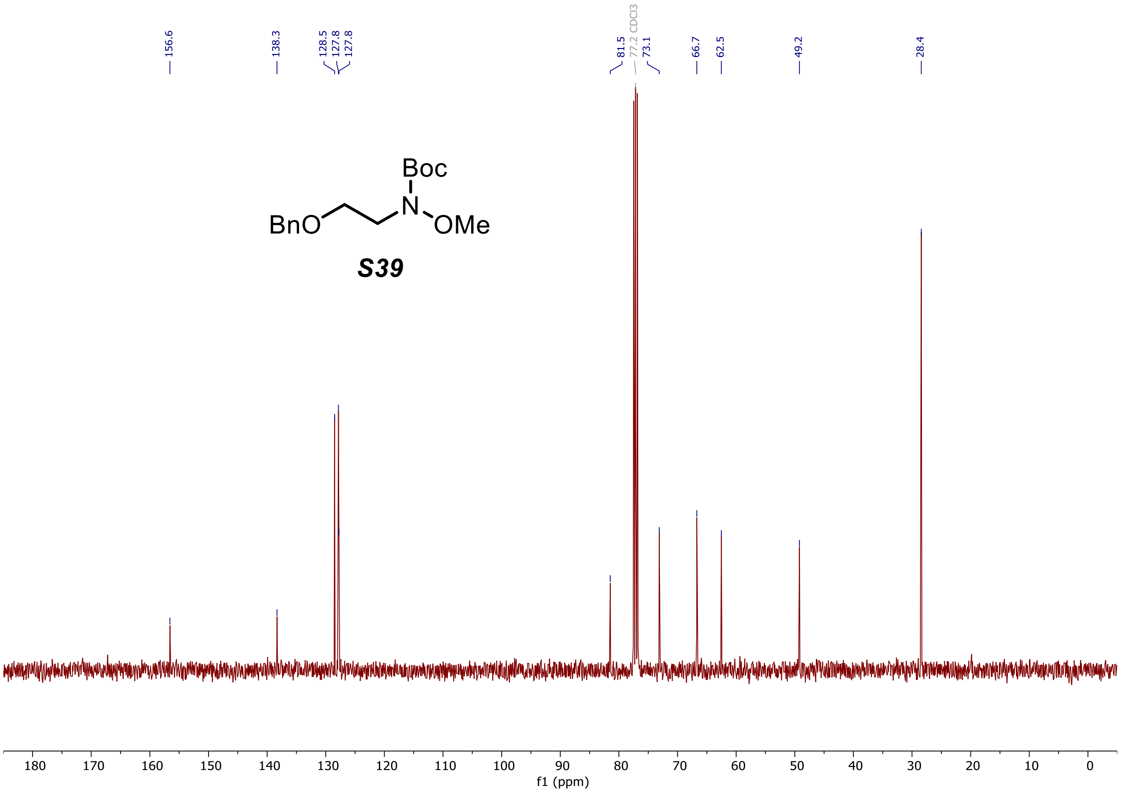

Copy of ^{1}H NMR Spectrum (400 MHz, D_2O) of **5l**

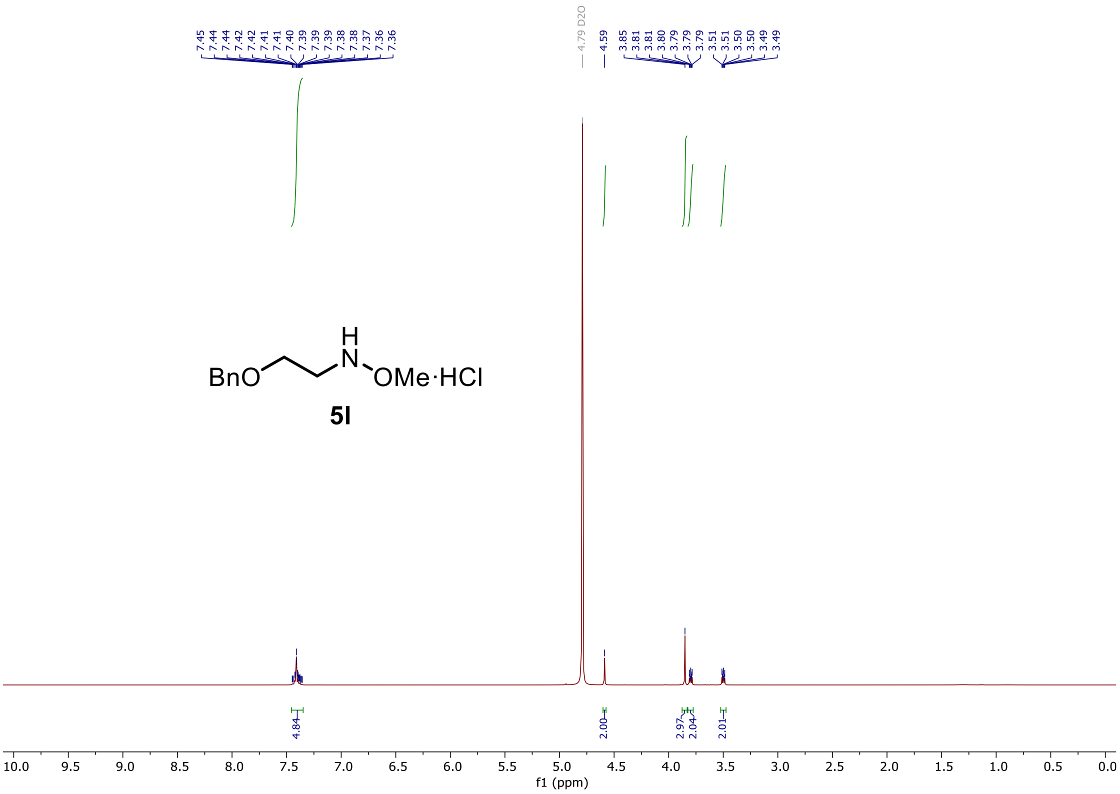

Copy of ^{13}C {^{1}H} NMR Spectrum (101 MHz, D_2O) of **5l**

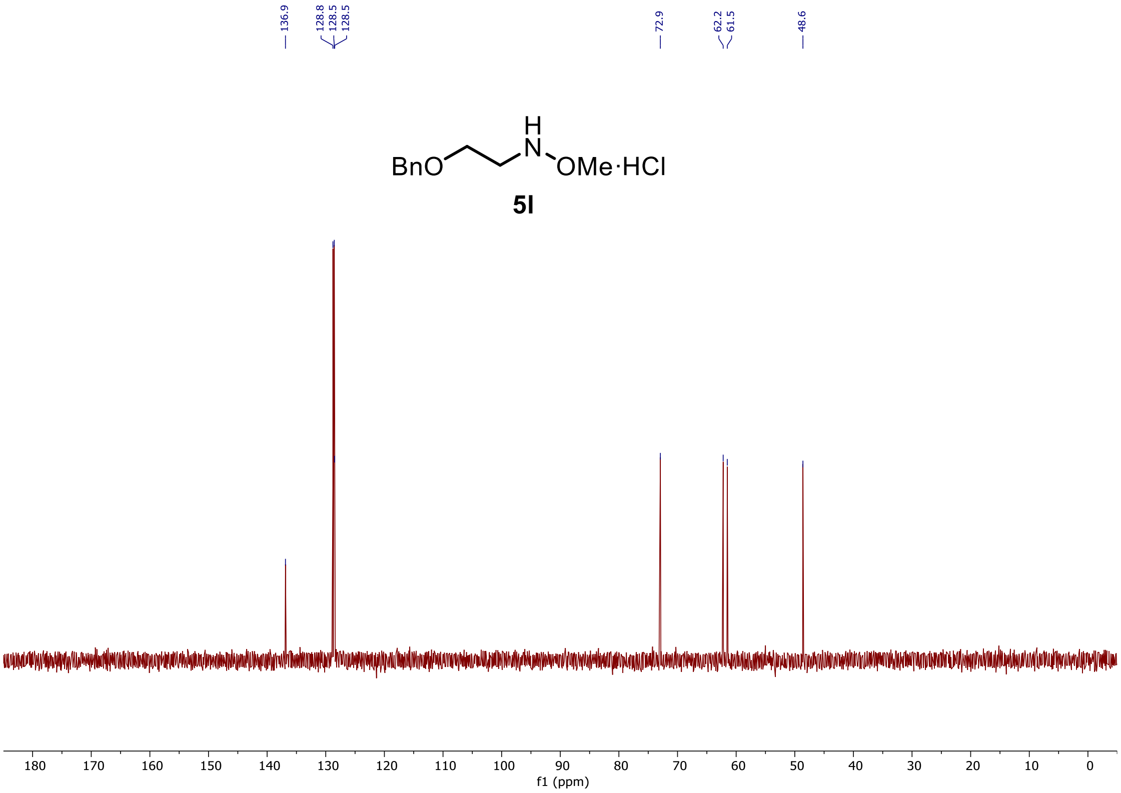

Copy of ^{1}H NMR Spectrum (400 MHz, $CDCl_3$) of **S40**

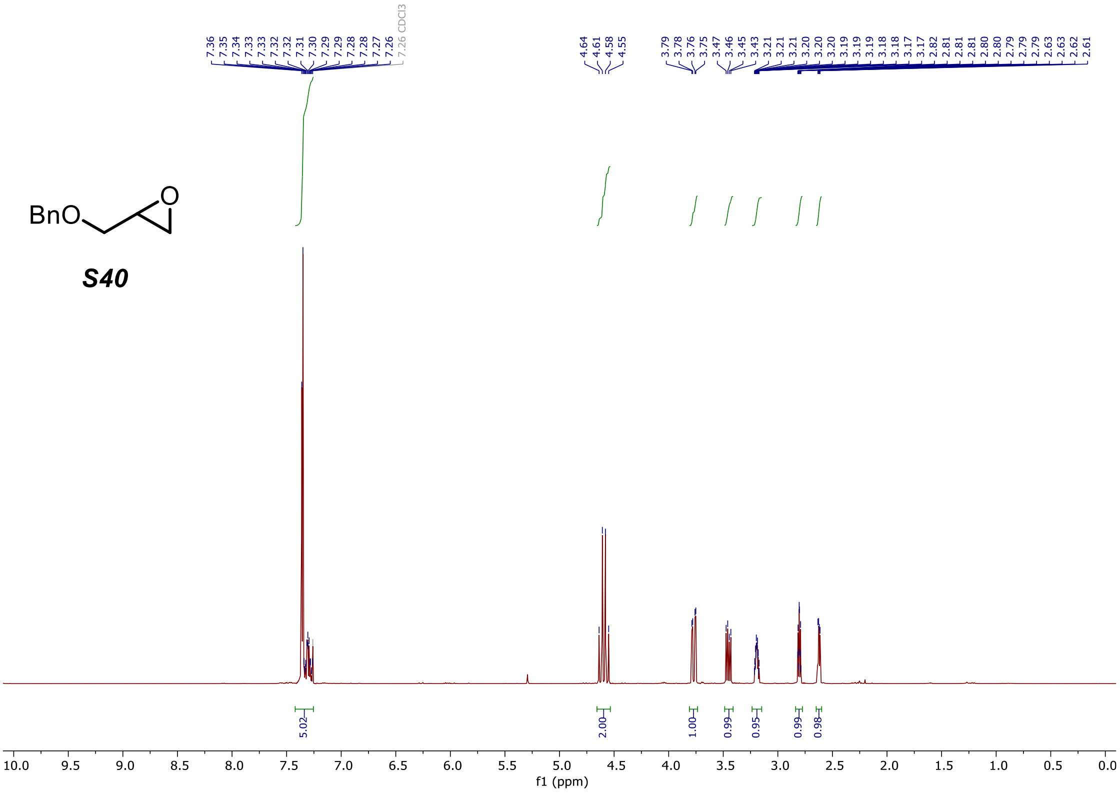

Copy of ^{13}C {^{1}H} NMR Spectrum (101 MHz, $CDCl_3$) of **S40**

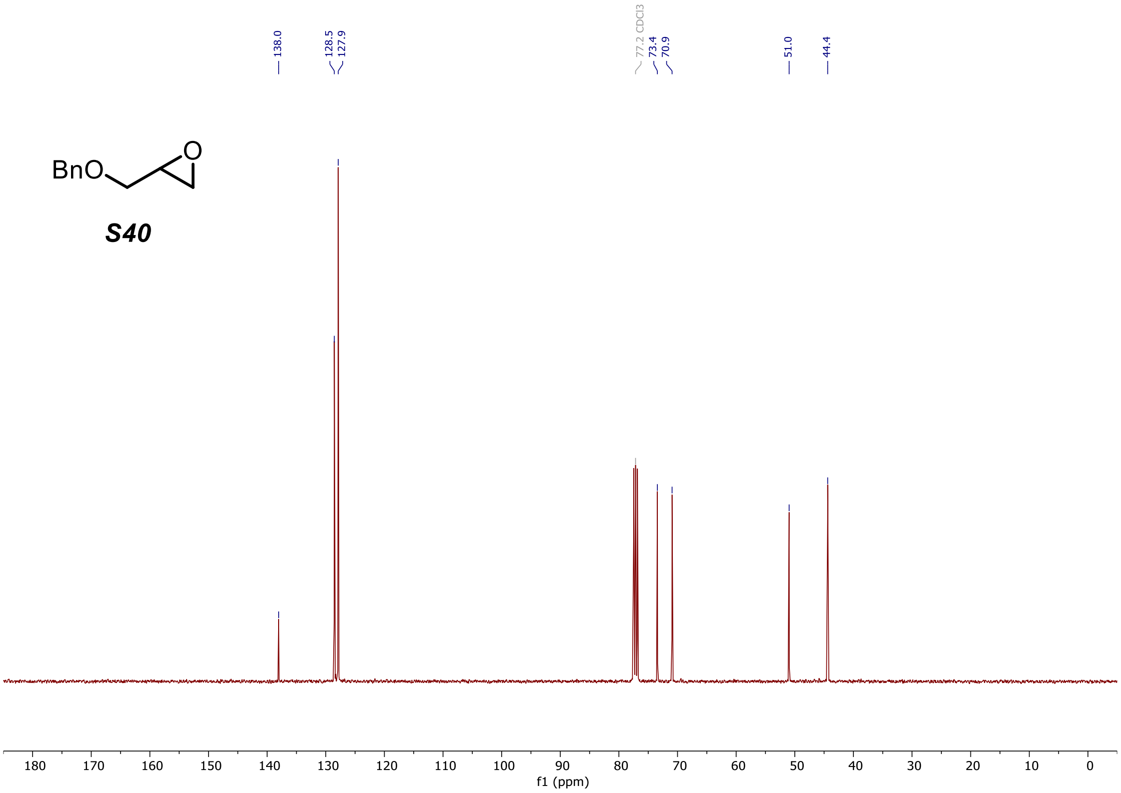

Copy of ^{1}H NMR Spectrum (400 MHz, $CDCl_3$) of **S41**

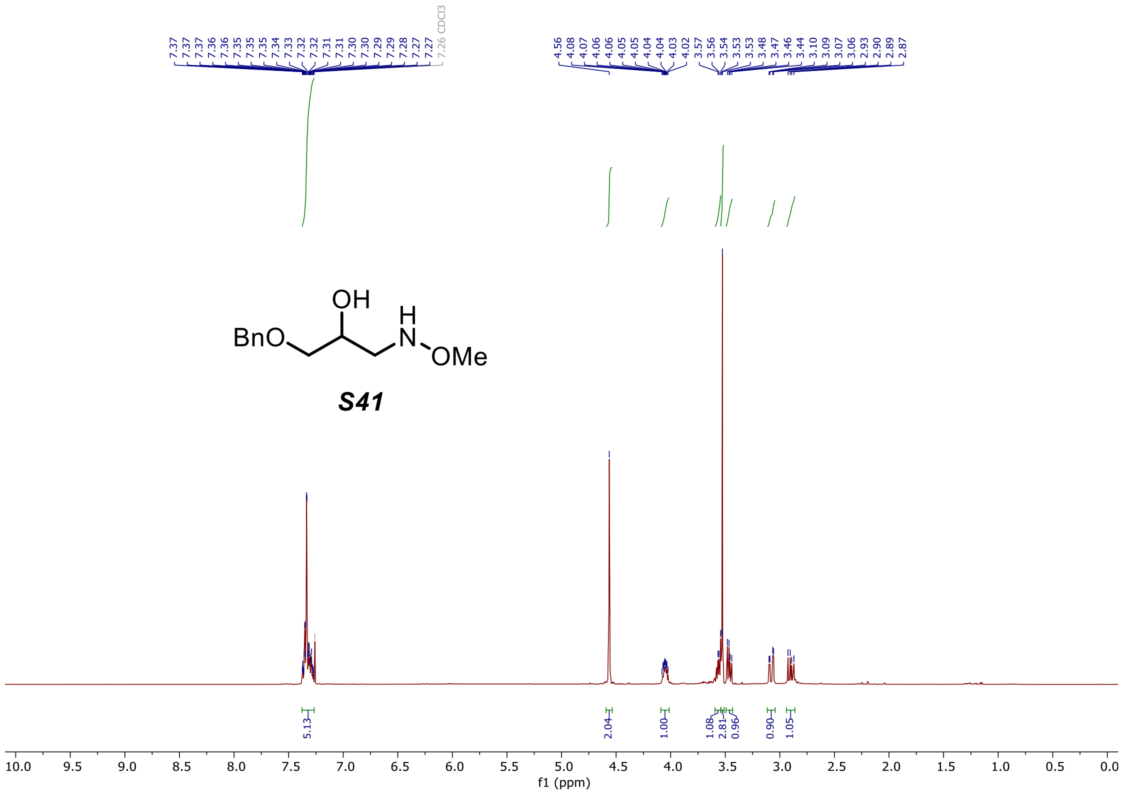

Copy of ^{13}C {^{1}H} NMR Spectrum (101 MHz, $CDCl_3$) of **S41**

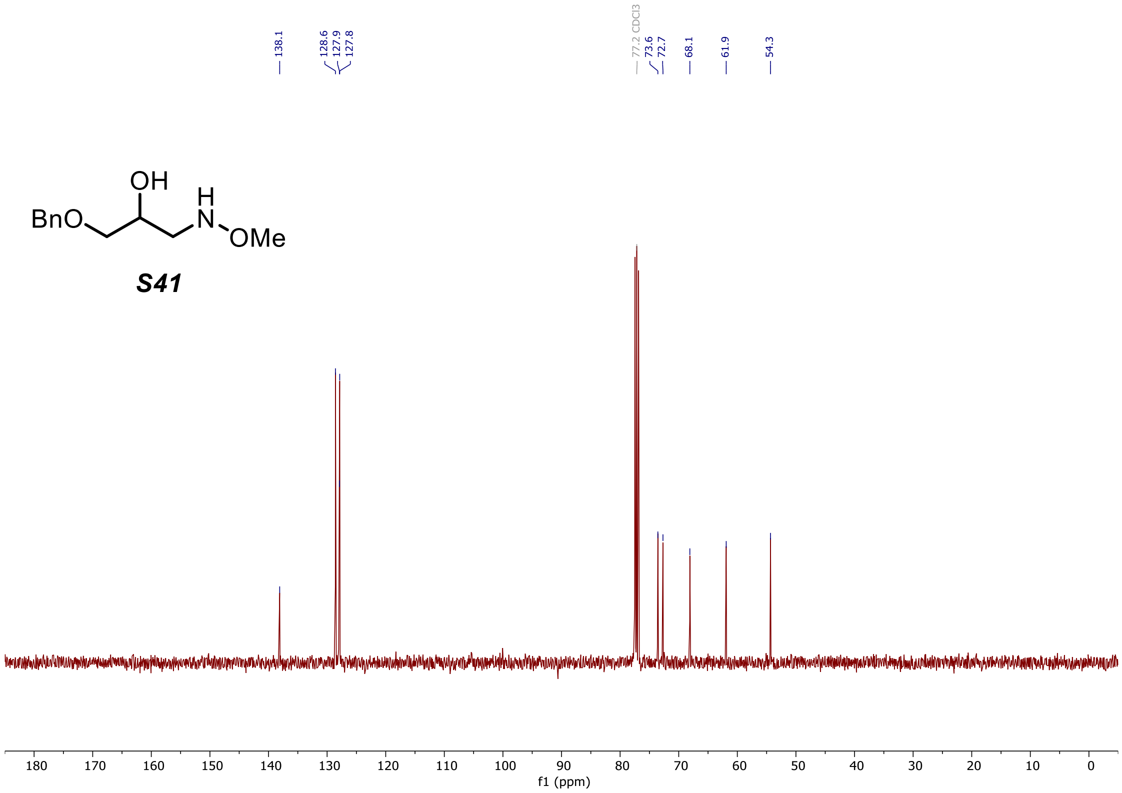

Copy of ^{1}H NMR Spectrum (400 MHz, $CDCl_3$) of **5m**

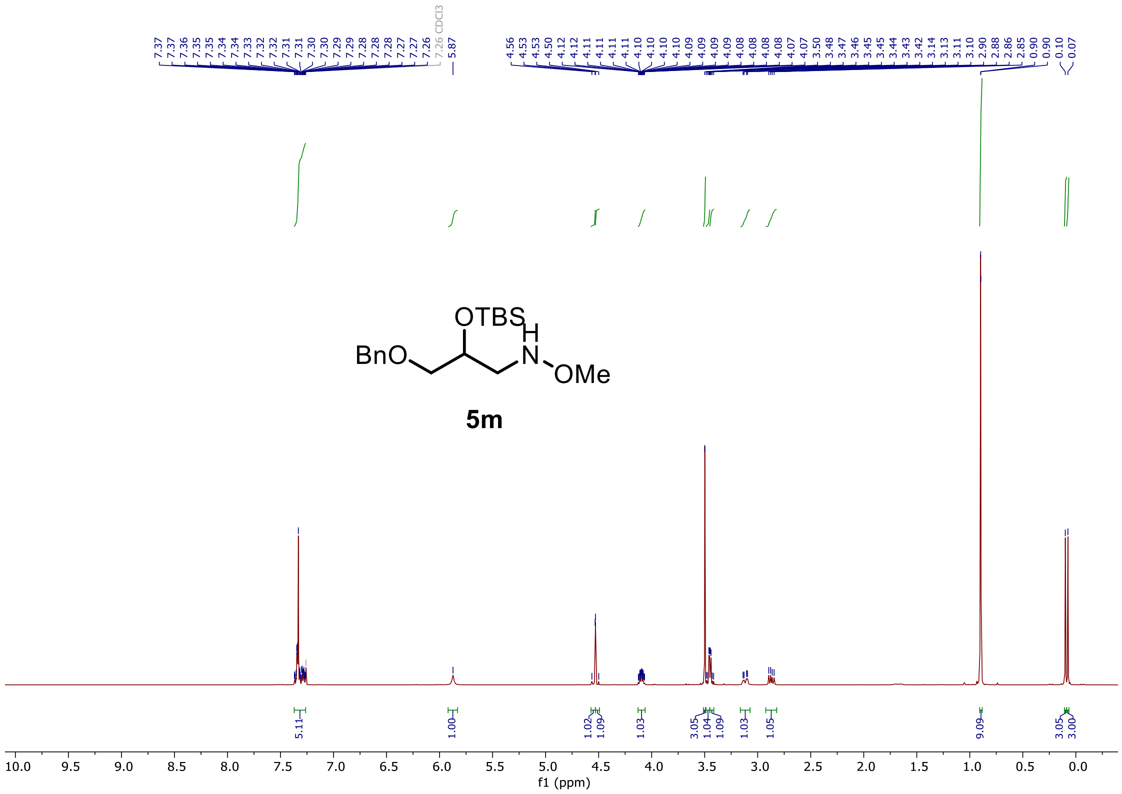

Copy of ^{13}C {^{1}H} NMR Spectrum (101 MHz, $CDCl_3$) of **5m**

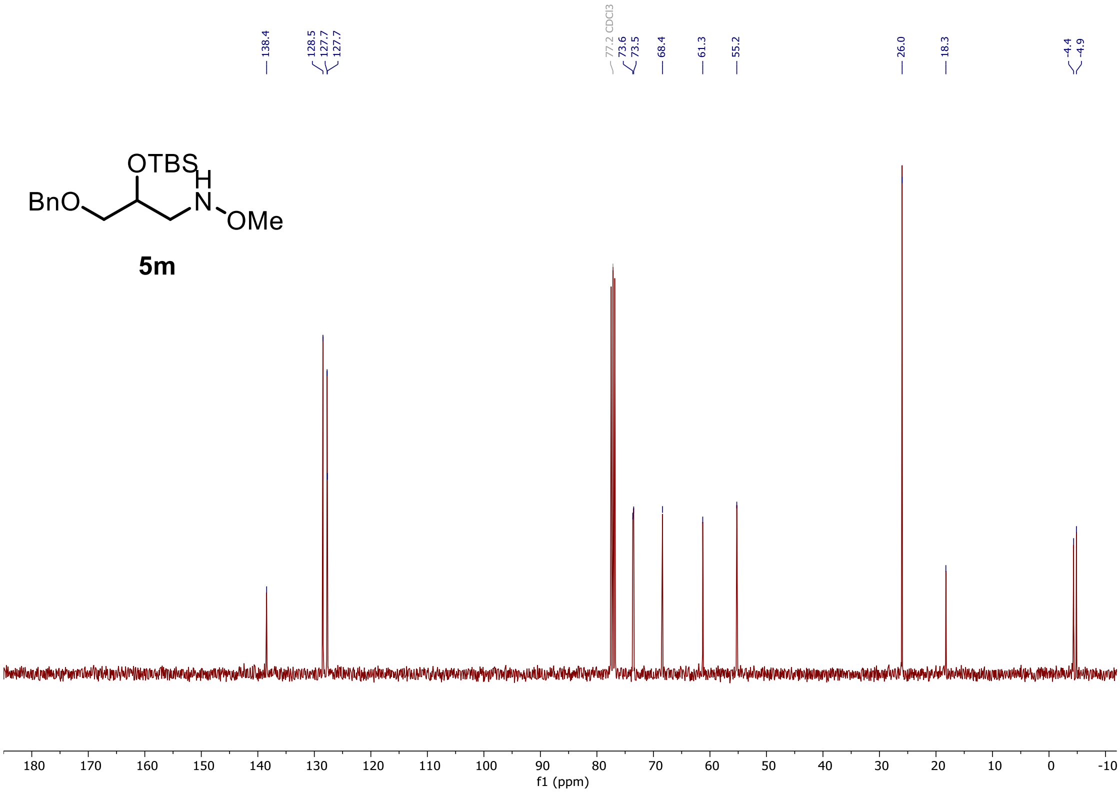

Copy of ^{1}H NMR Spectrum (400 MHz, $CDCl_3$) of **S43**

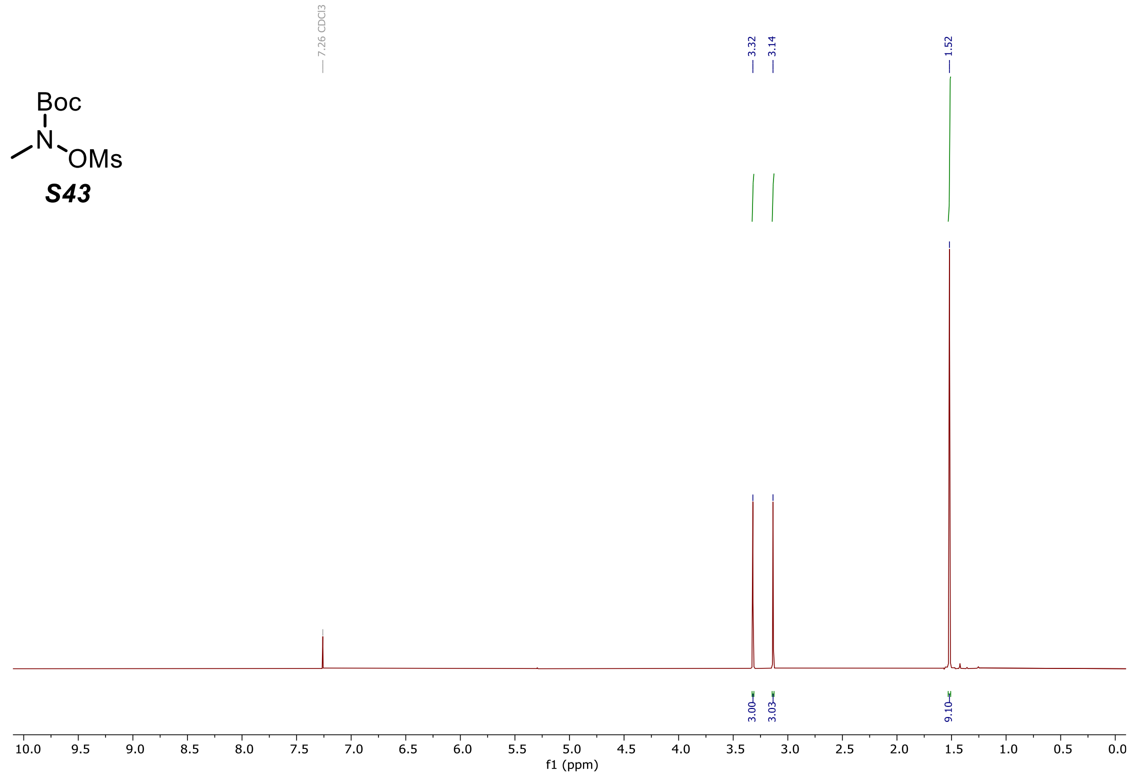

Copy of ^{13}C {^{1}H} NMR Spectrum (101 MHz, $CDCl_3$) of **S43**

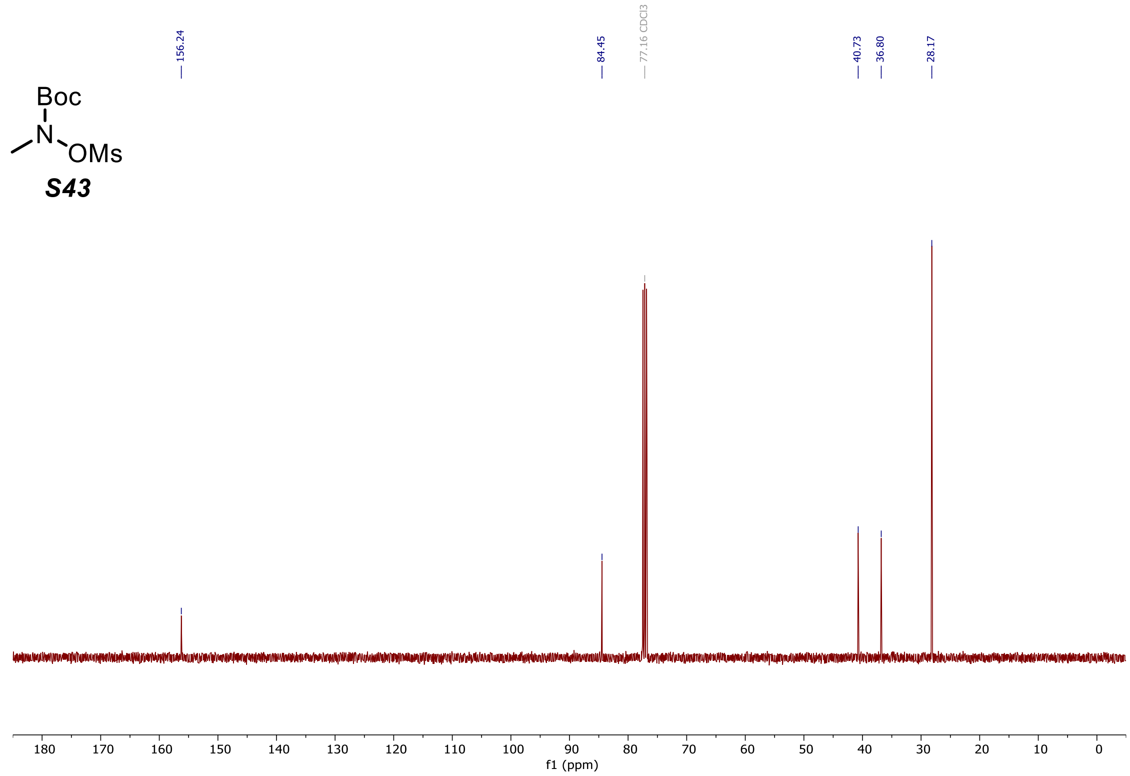

Copy of ^{1}H NMR Spectrum (400 MHz, $CDCl_3$) of **5n**

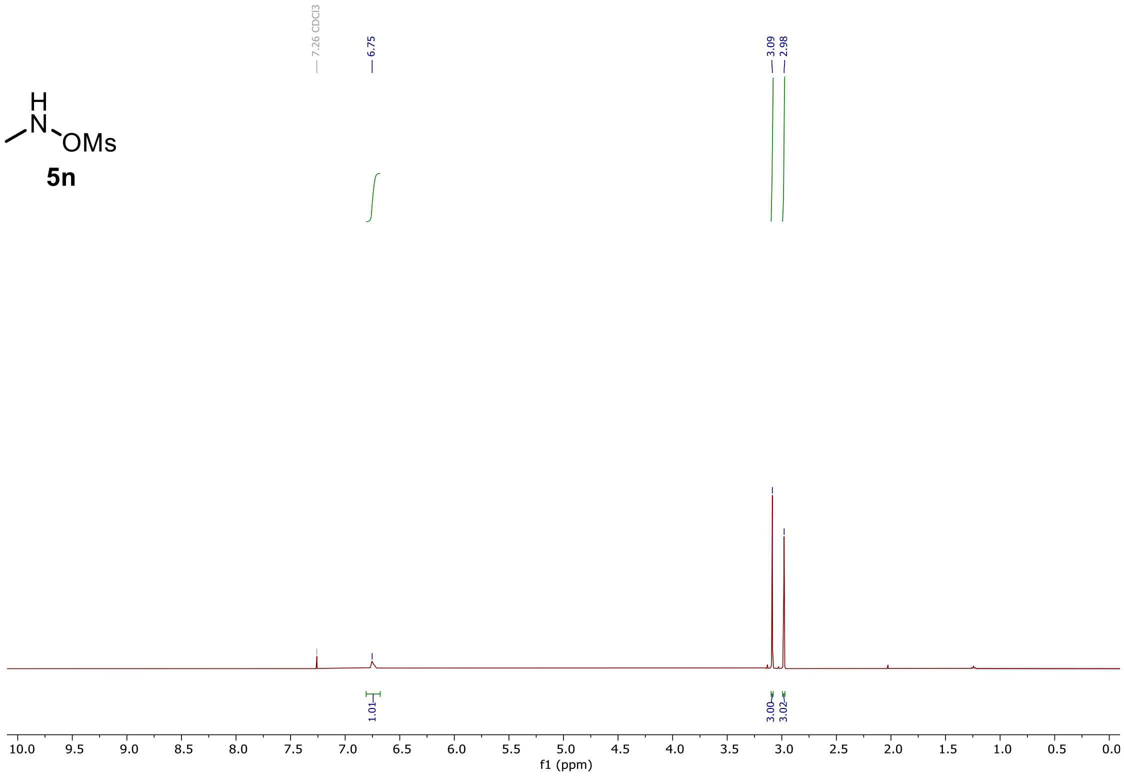

Copy of ^{13}C {^{1}H} NMR Spectrum (101 MHz, $CDCl_3$) of **5n**

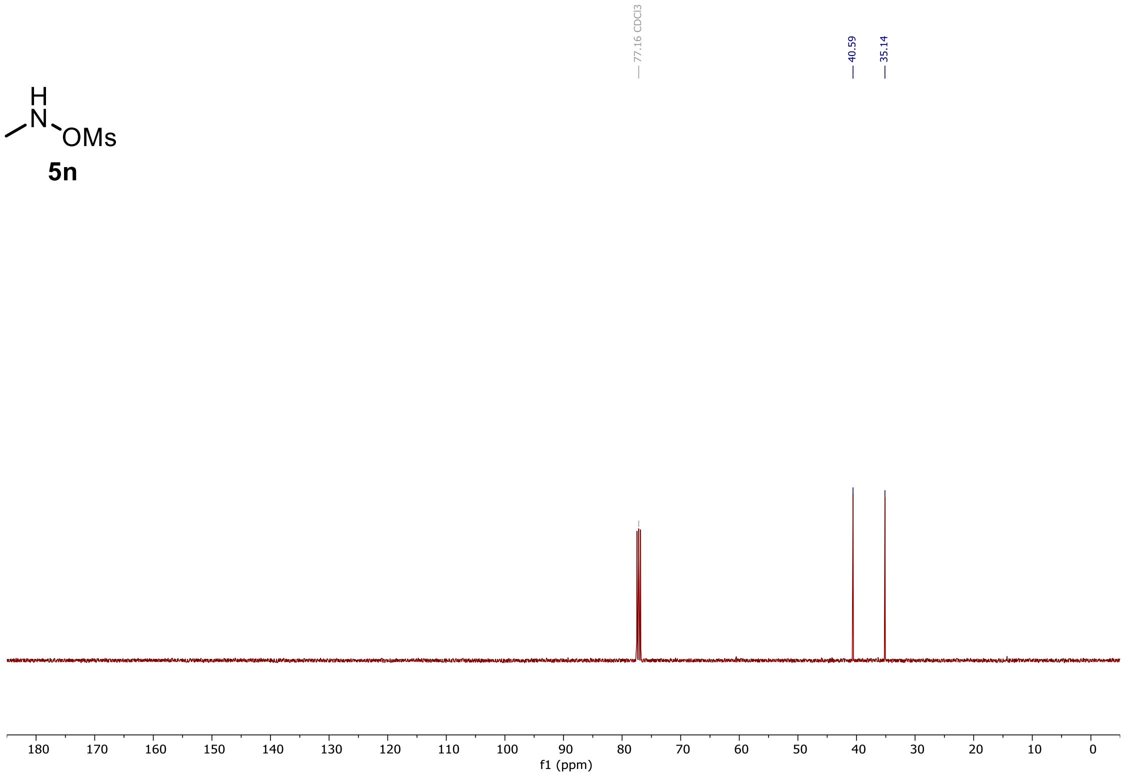

Copy of ^{1}H NMR Spectrum (400 MHz, $CDCl_3$) of **S45**

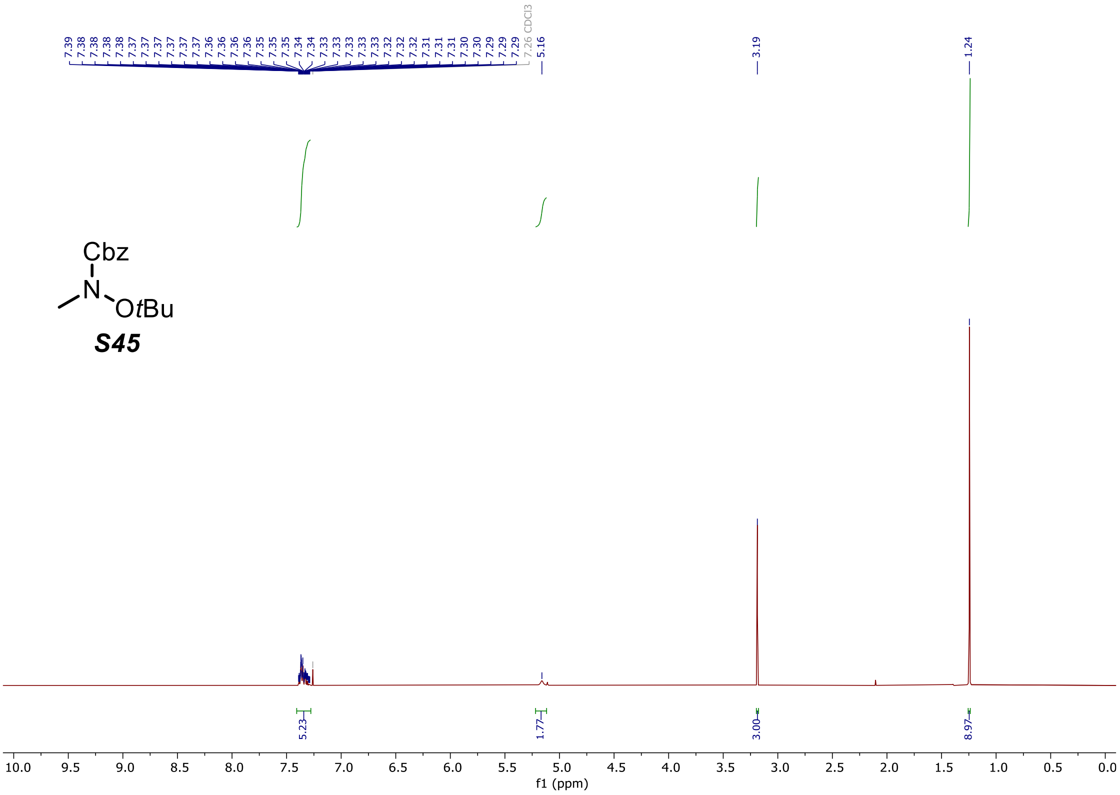

Copy of ^{13}C {^{1}H} NMR Spectrum (101 MHz, $CDCl_3$) of **S45**

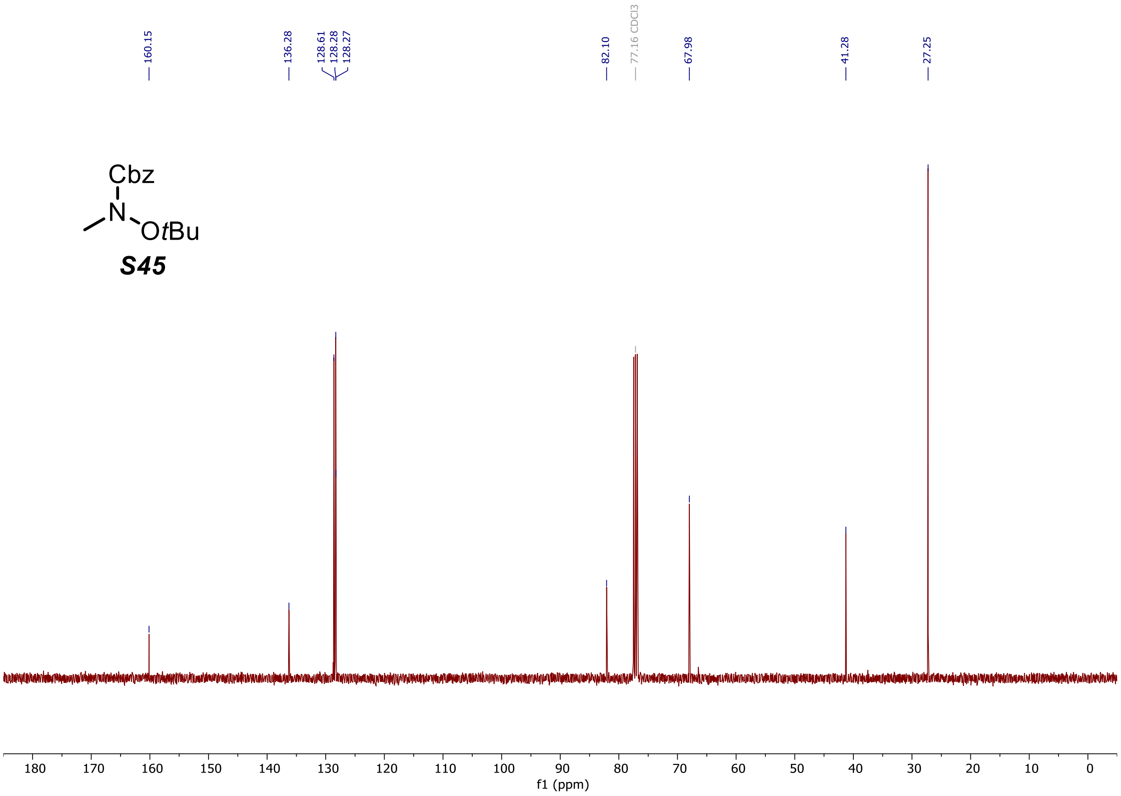

Copy of ^{1}H NMR Spectrum (400 MHz, $CDCl_3$) of **5o**

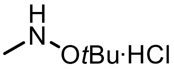

5o

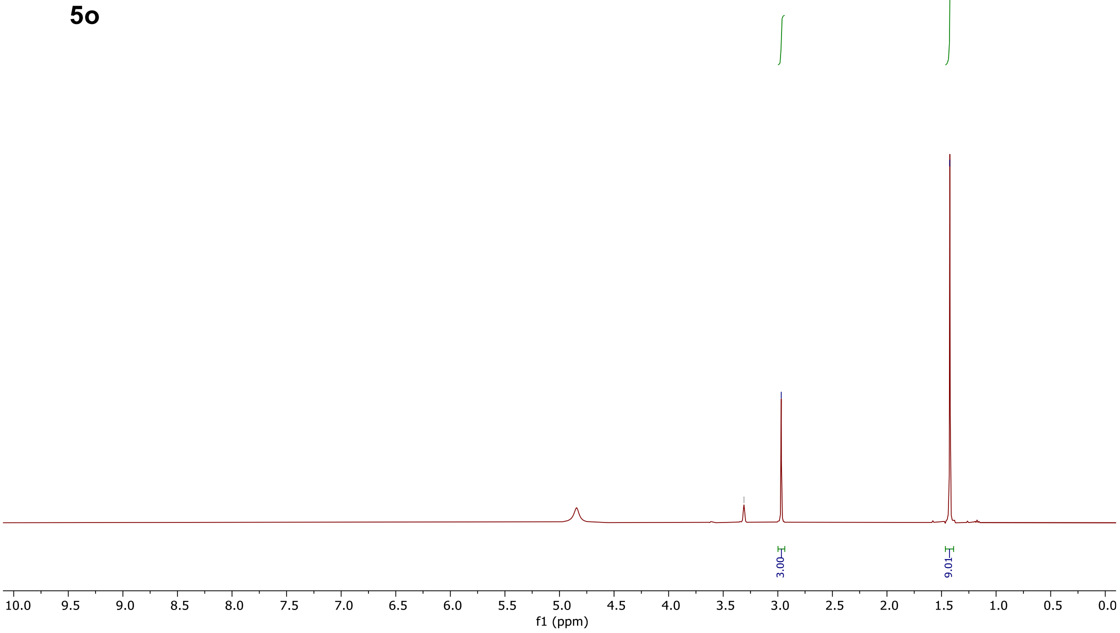

Copy of ^{13}C {^{1}H} NMR Spectrum (101 MHz, $CDCl_3$) of **5o**

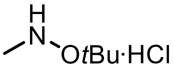

5o

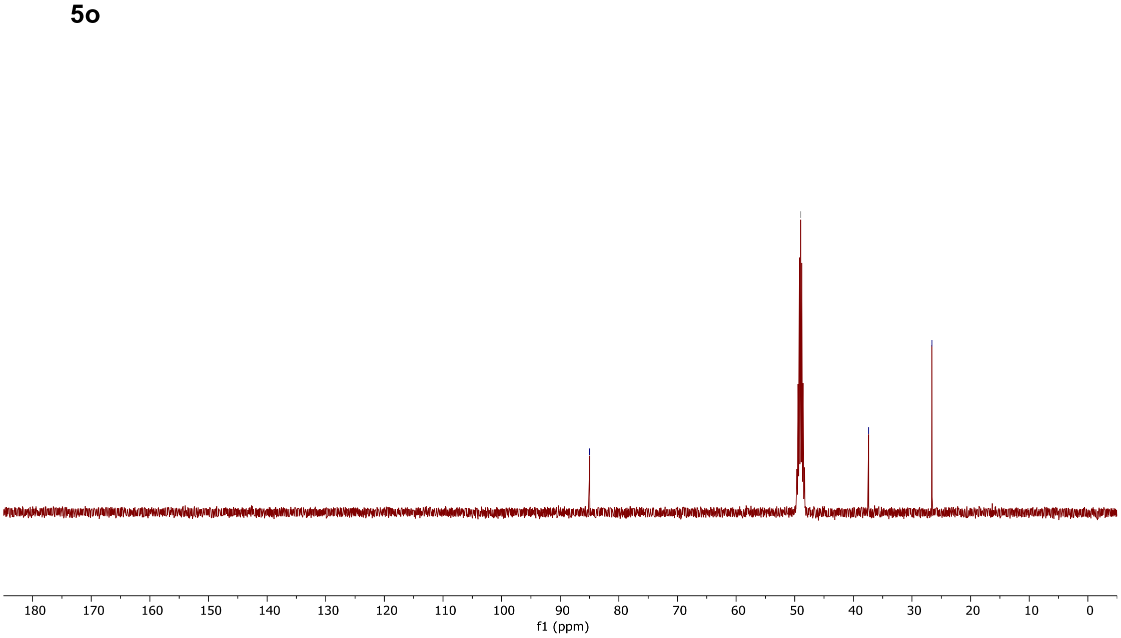

Copy of ^{1}H NMR Spectrum (400 MHz, $CDCl_3$) of **3a**

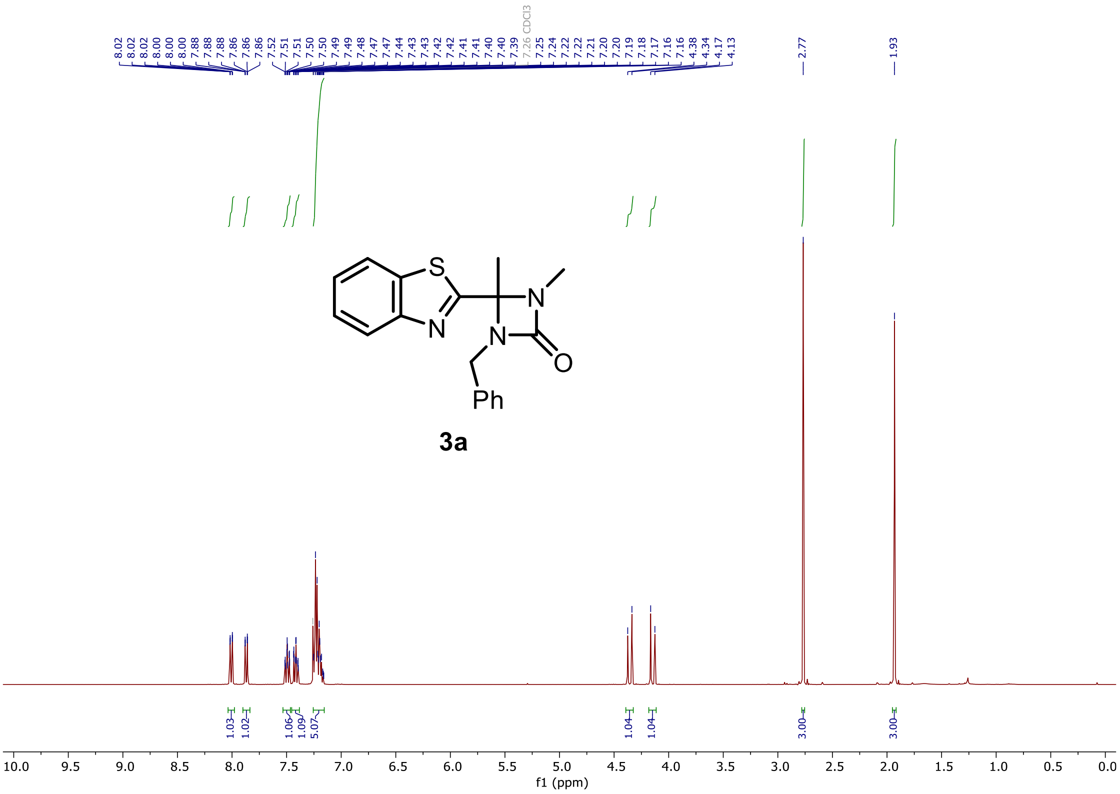

Copy of ^{13}C {^{1}H} NMR Spectrum (101 MHz, $CDCl_3$) of **3a**

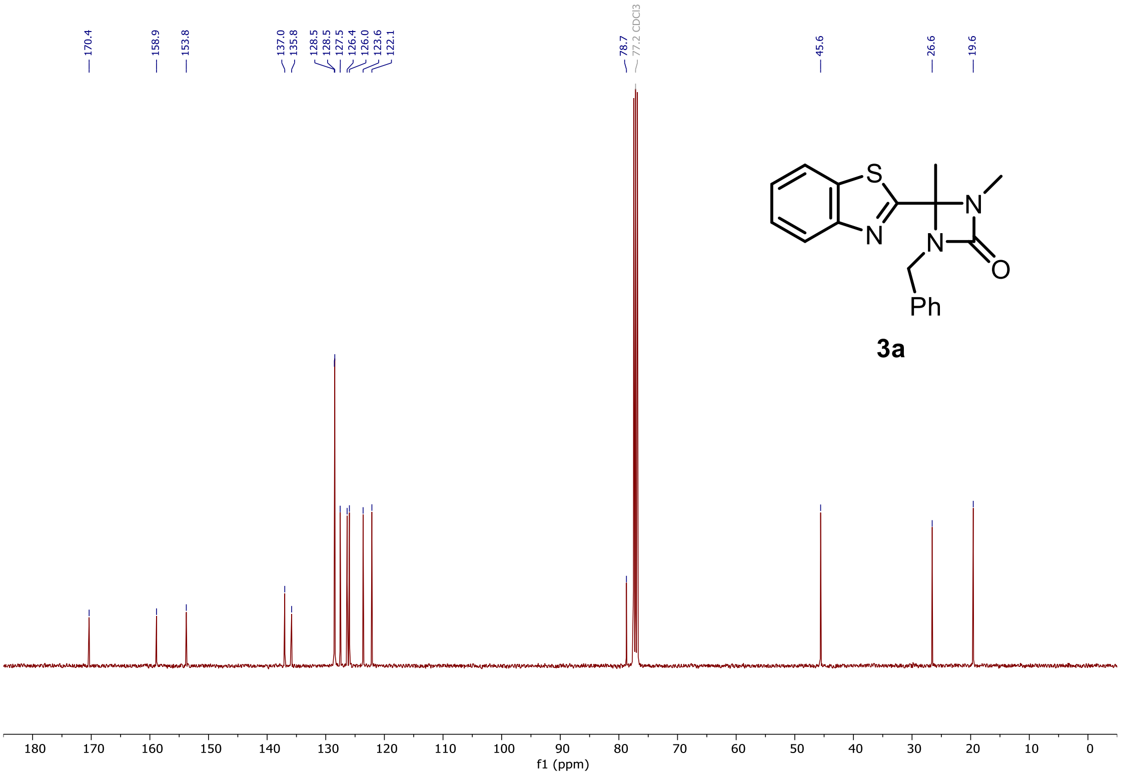

Copy of ^{1}H NMR Spectrum (400 MHz, $CDCl_3$) of **3b**

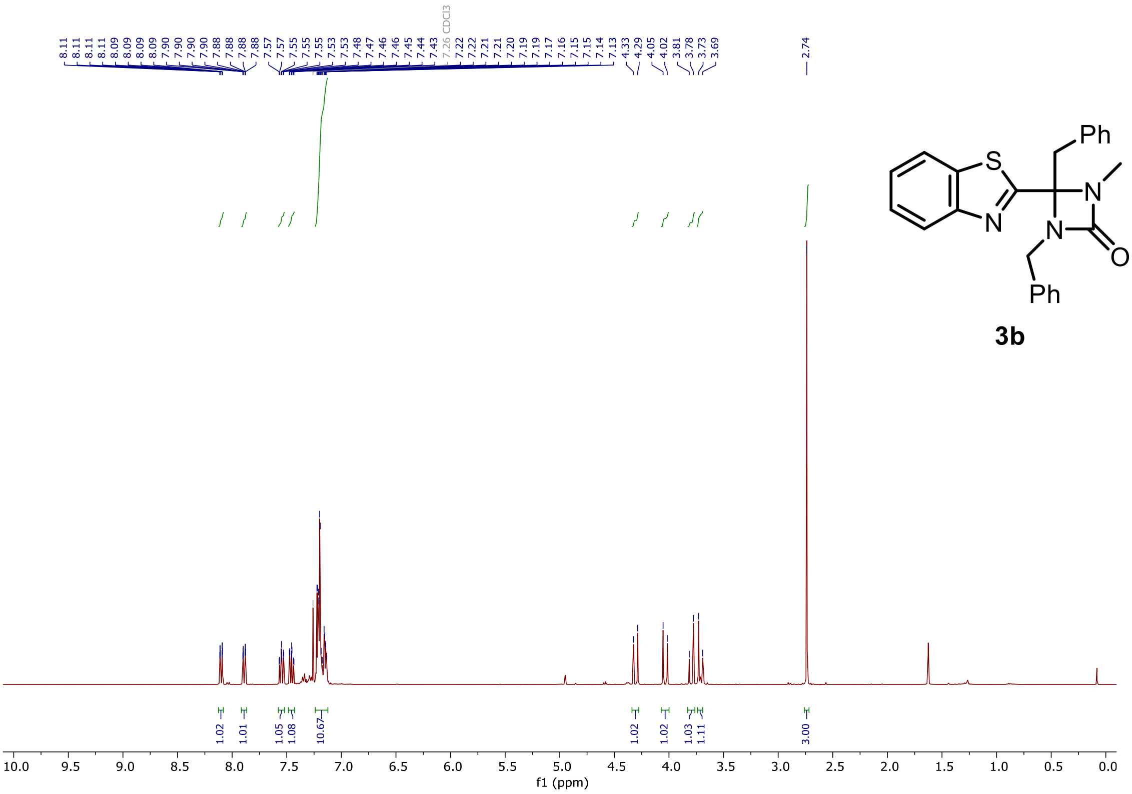

3b

Copy of ^{13}C {^{1}H} NMR Spectrum (101 MHz, $CDCl_3$) of **3b**

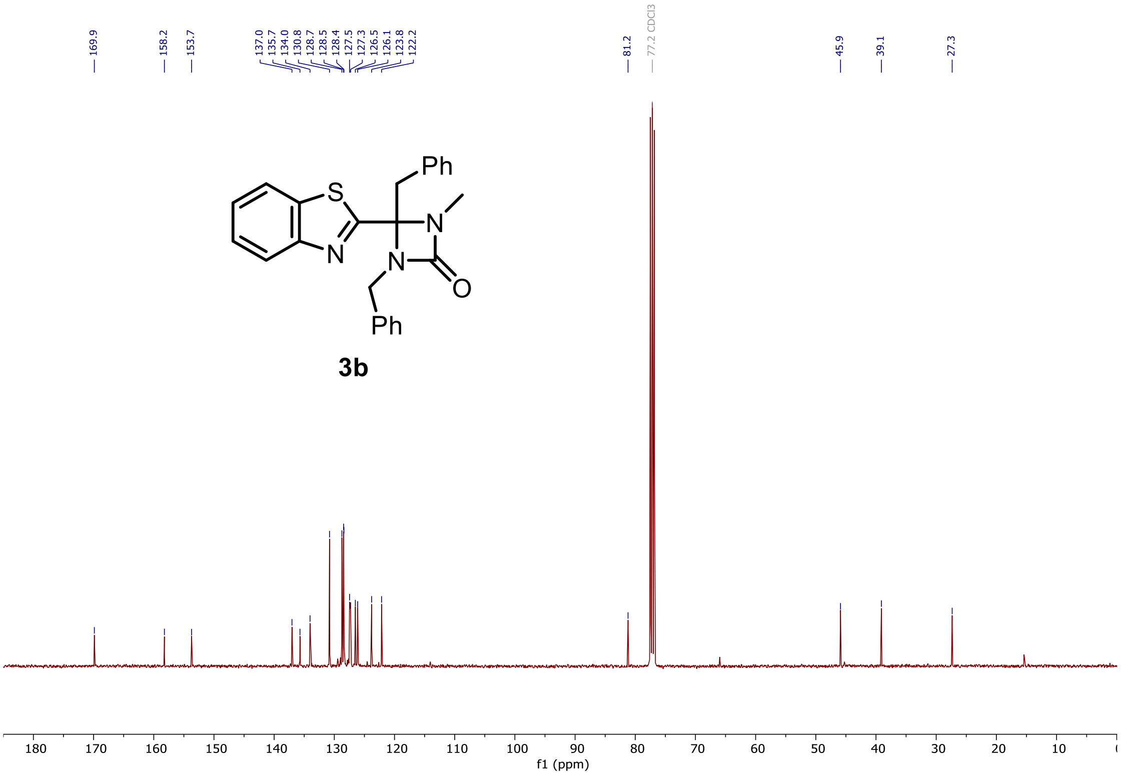

3b

Copy of COSY NMR Spectrum (400 MHz, $CDCl_3$) of **3b**

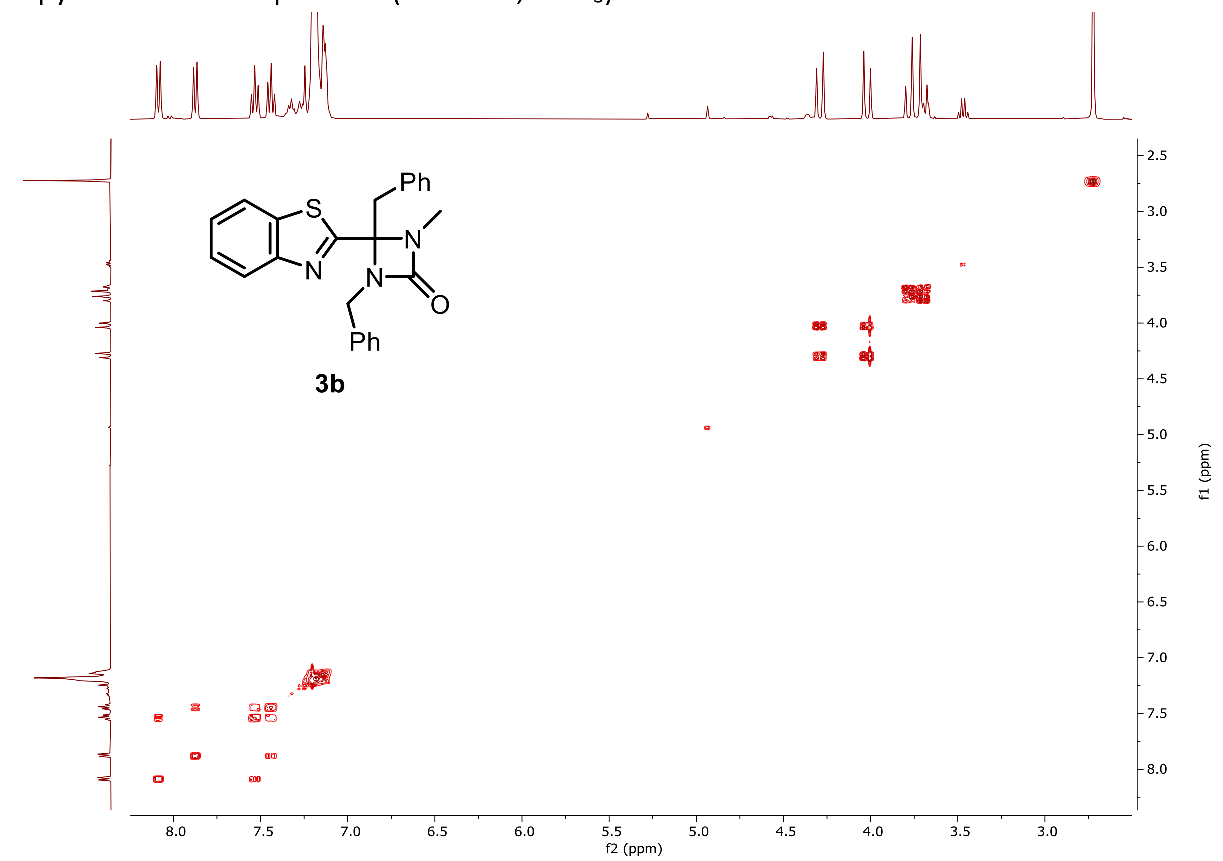

Copy of HMBC NMR Spectrum (400 MHz, $CDCl_3$) of **3b**

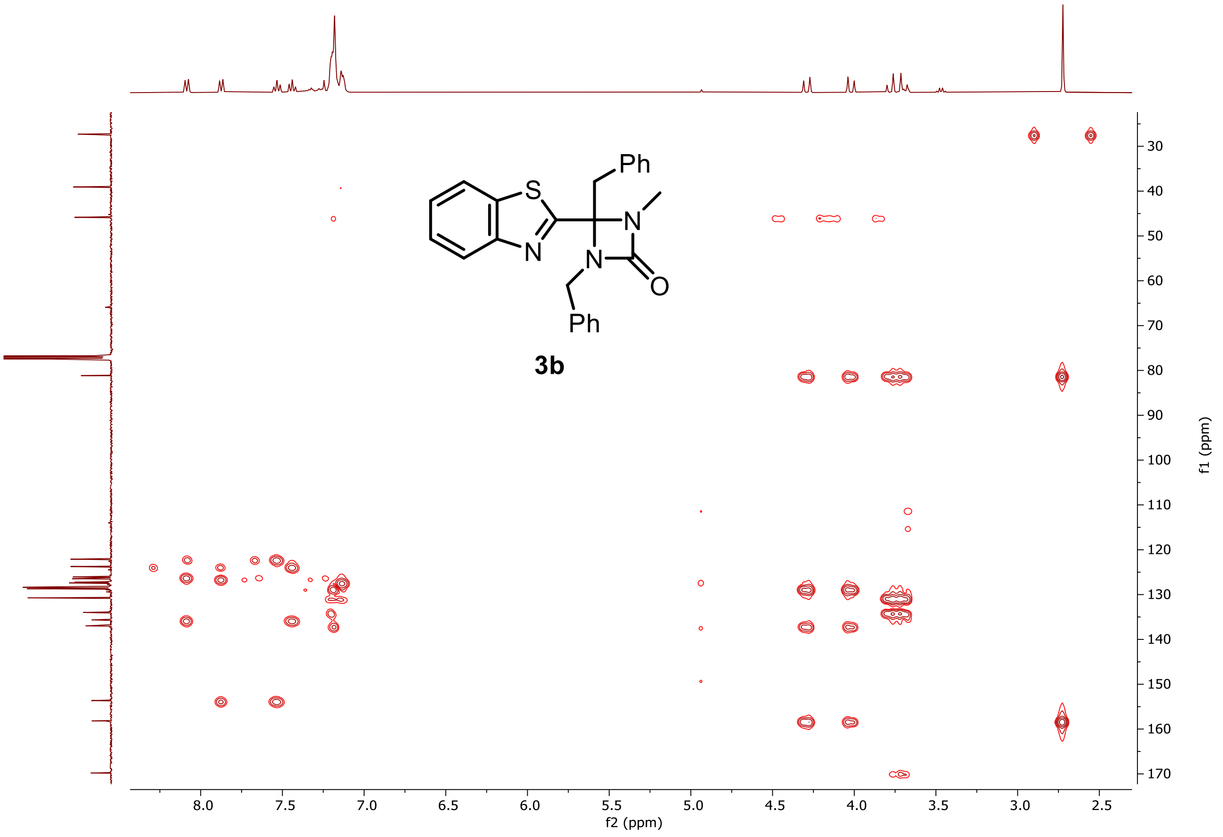

Copy of HMQC NMR Spectrum (400 MHz, $CDCl_3$) of **3b**

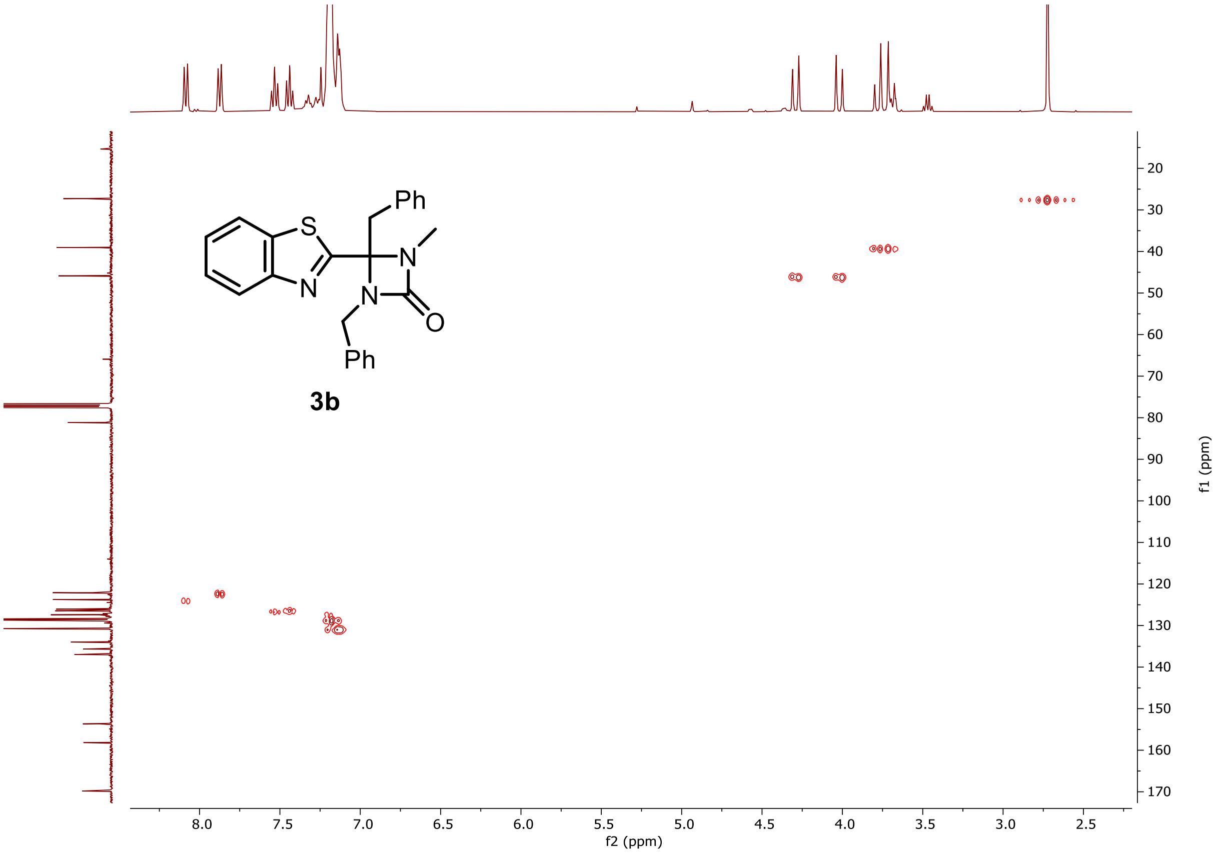

Copy of ^{1}H NMR Spectrum (500 MHz, $CDCl_3$) of **3c**

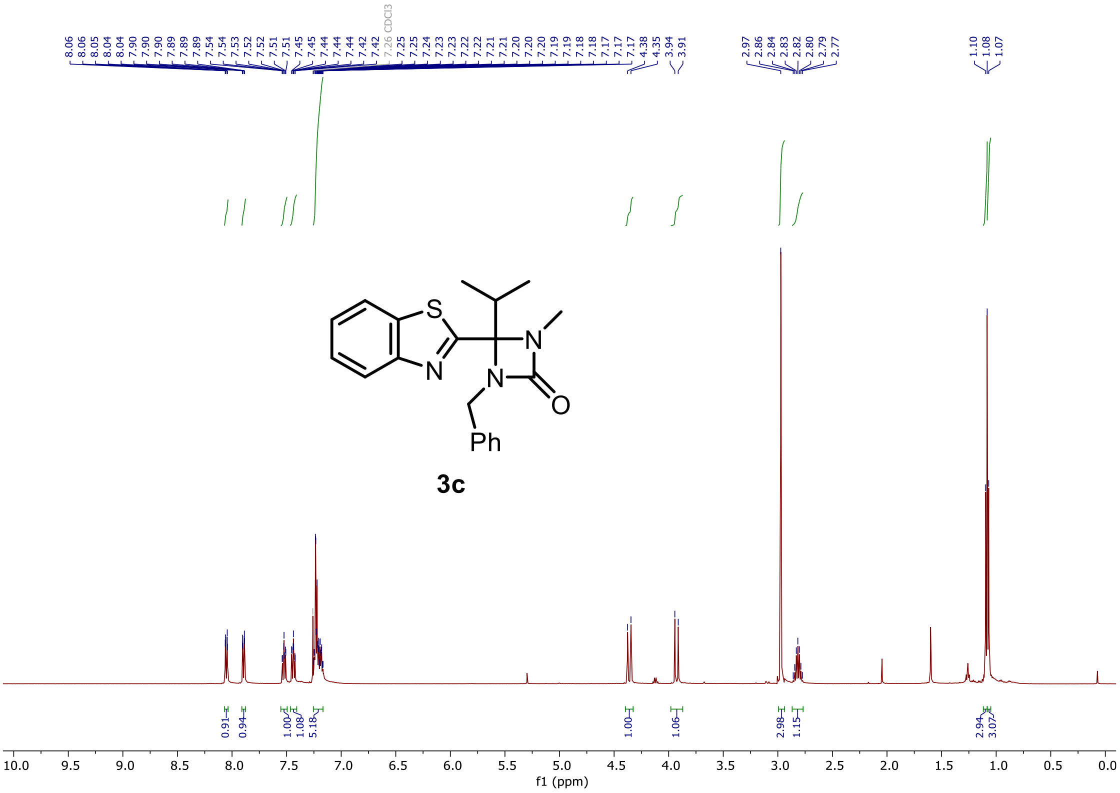

Copy of ^{13}C {^{1}H} NMR Spectrum (126 MHz, $CDCl_3$) of **3c**

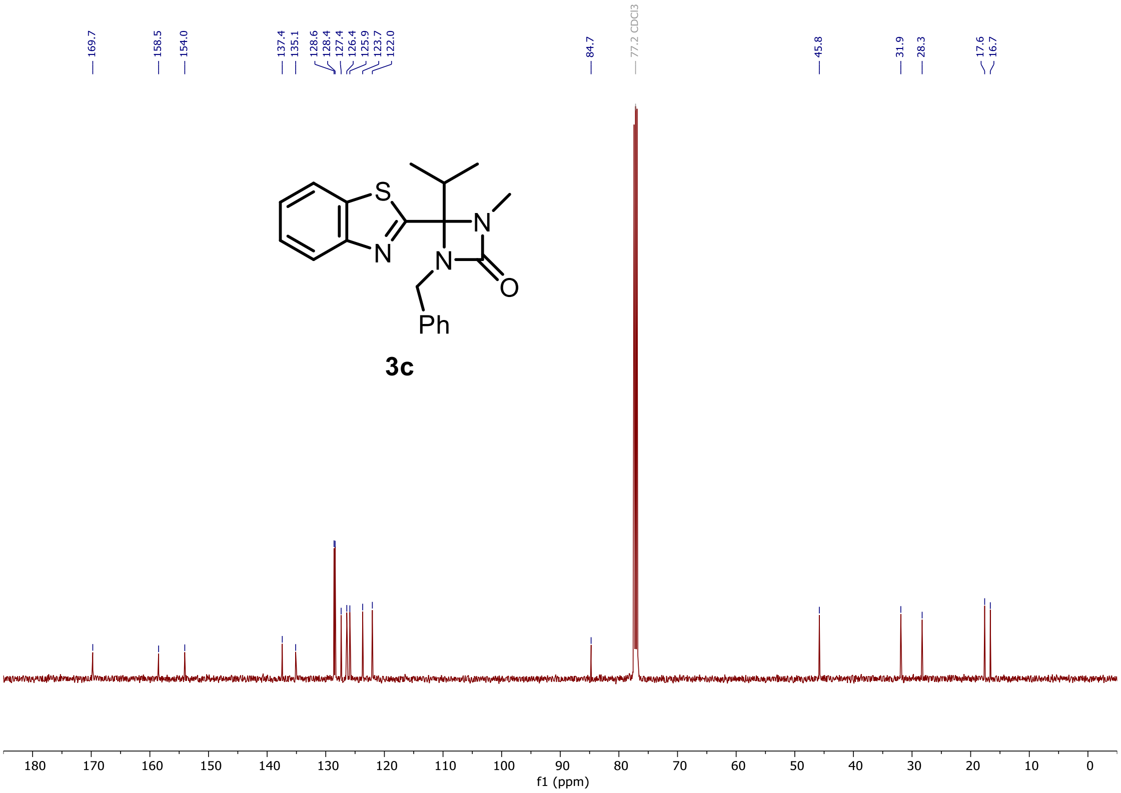

Copy of ^{1}H NMR Spectrum (500 MHz, $CDCl_3$) of **3d**

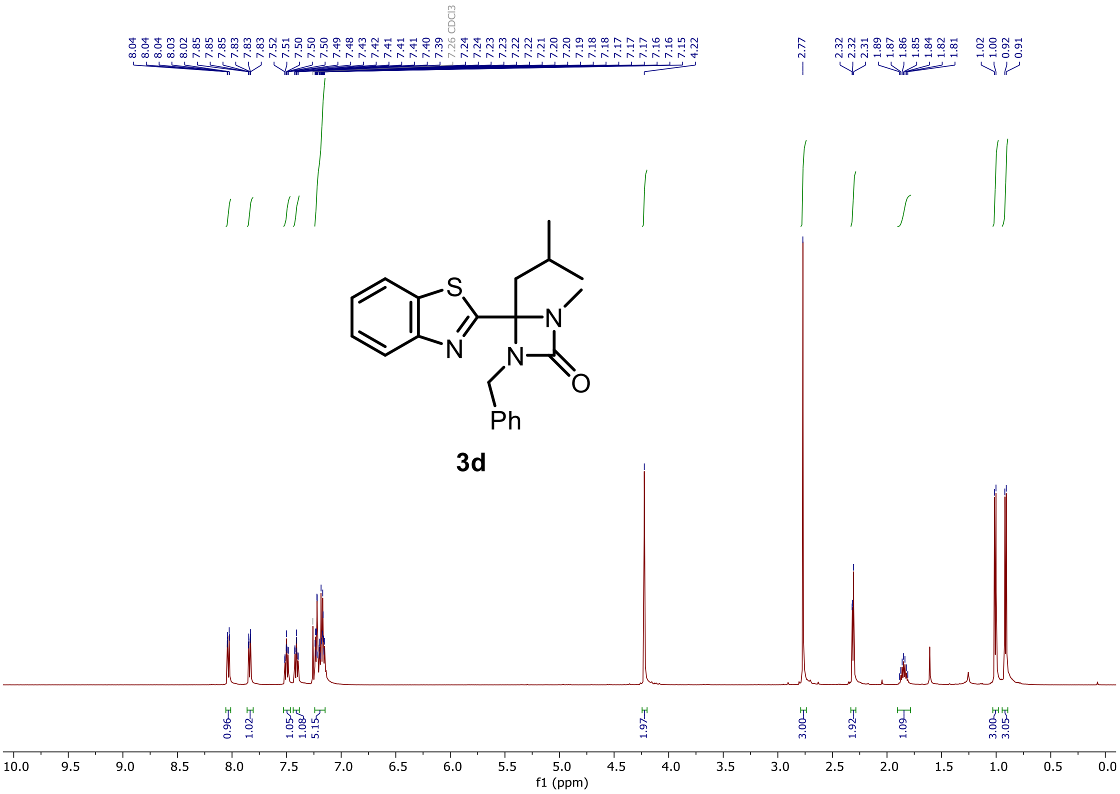

Copy of ^{13}C {^{1}H} NMR Spectrum (126 MHz, $CDCl_3$) of **3d**

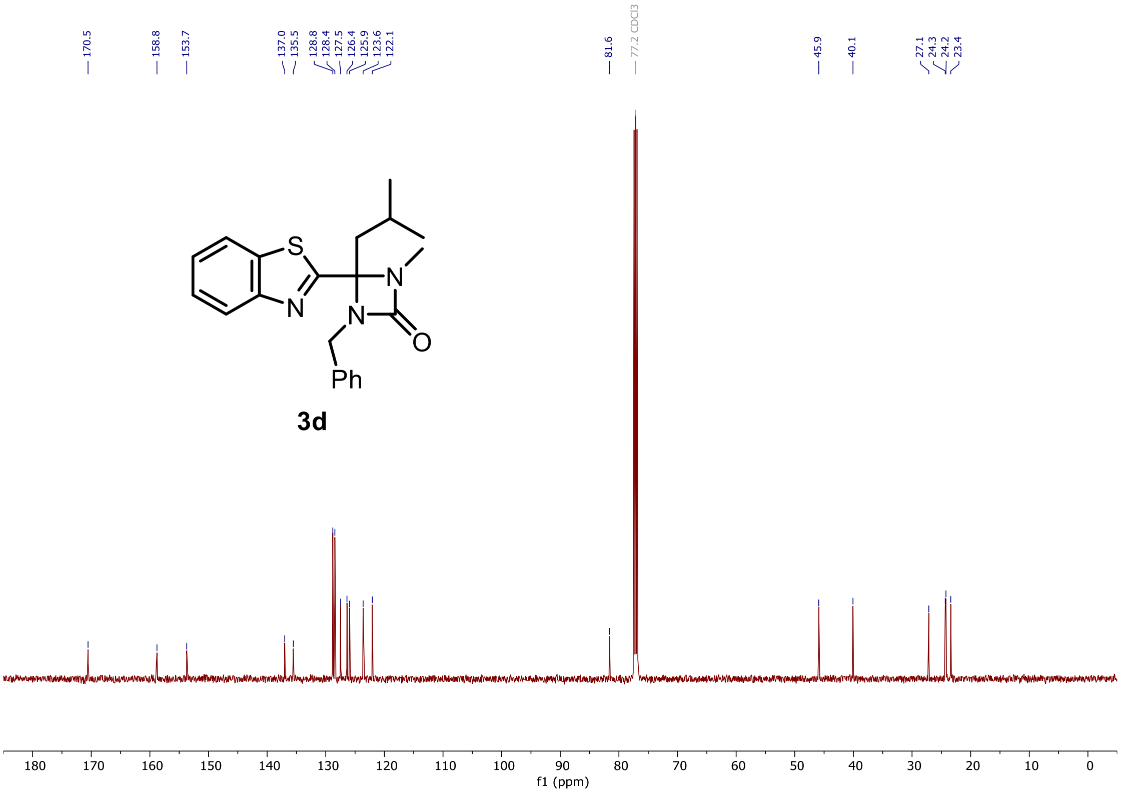

Copy of ^{1}H NMR Spectrum (500 MHz, $CDCl_3$) of **3e**

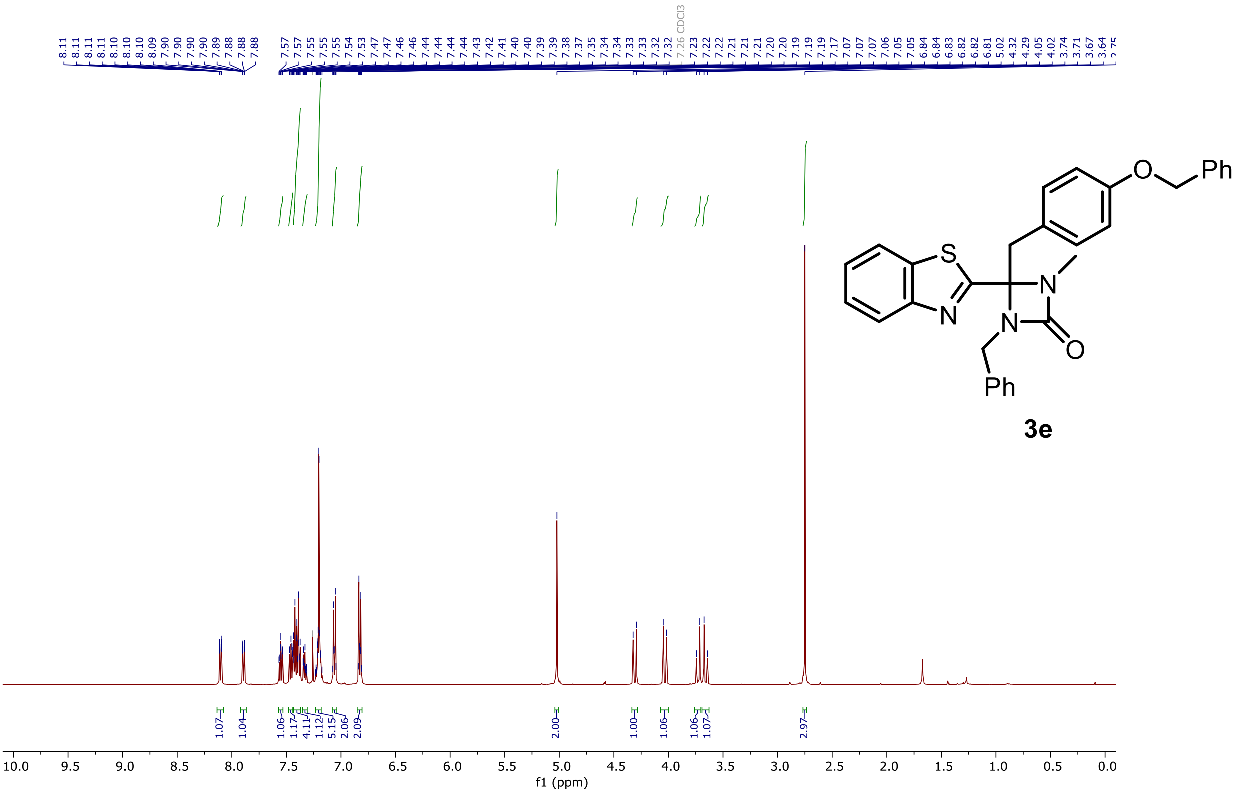

Copy of ^{13}C {^{1}H} NMR Spectrum (126 MHz, $CDCl_3$) of **3e**

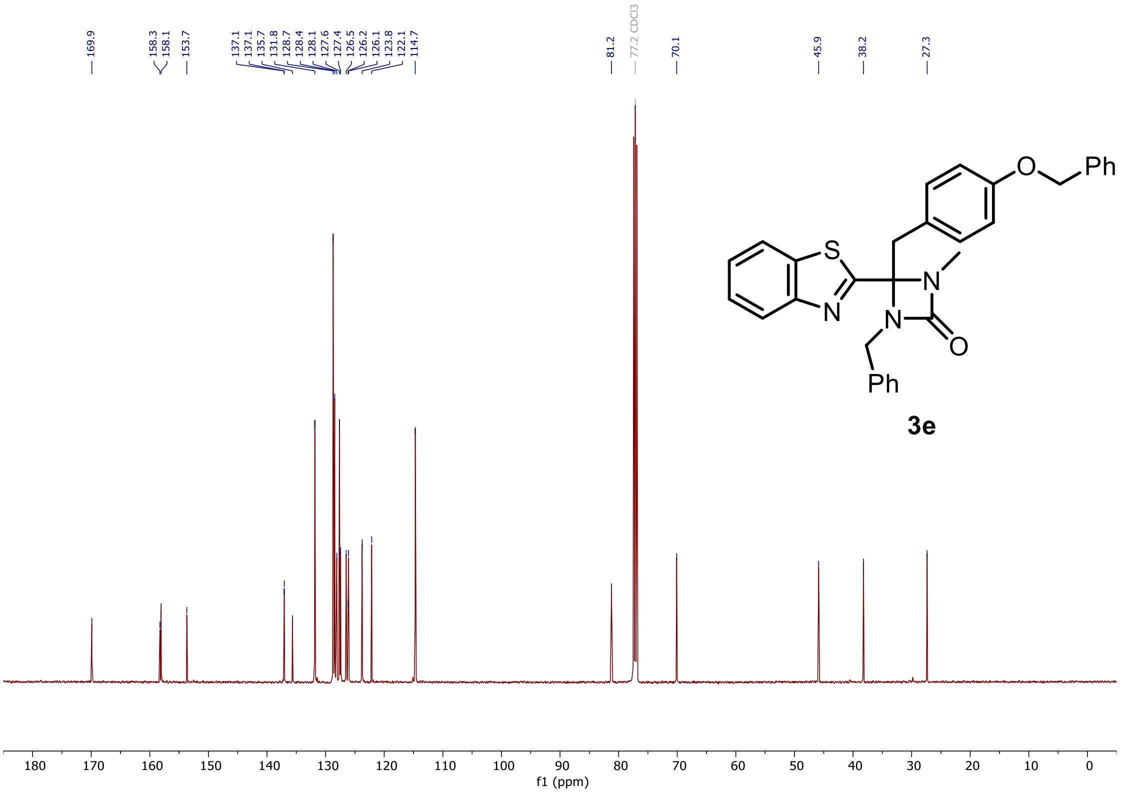

Copy of ^{1}H NMR Spectrum (400 MHz, $CDCl_3$) of **1g**

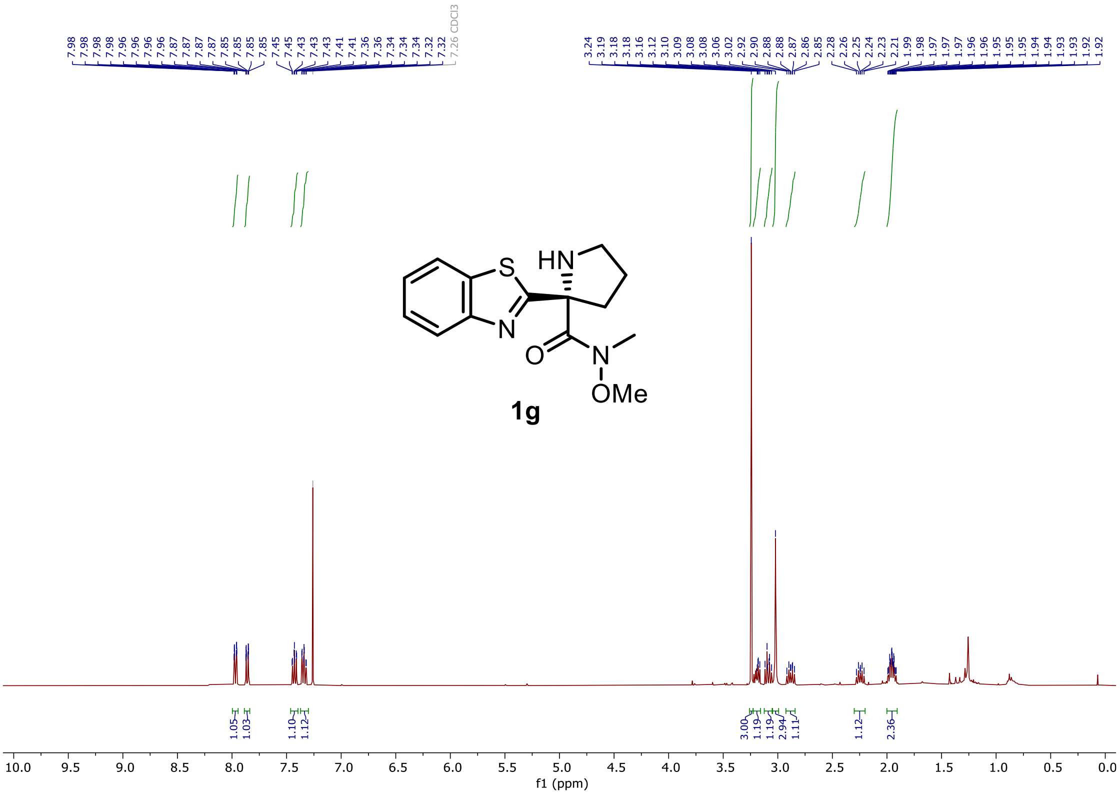

Copy of ^{13}C {^{1}H} NMR Spectrum (101 MHz, $CDCl_3$) of **1g**

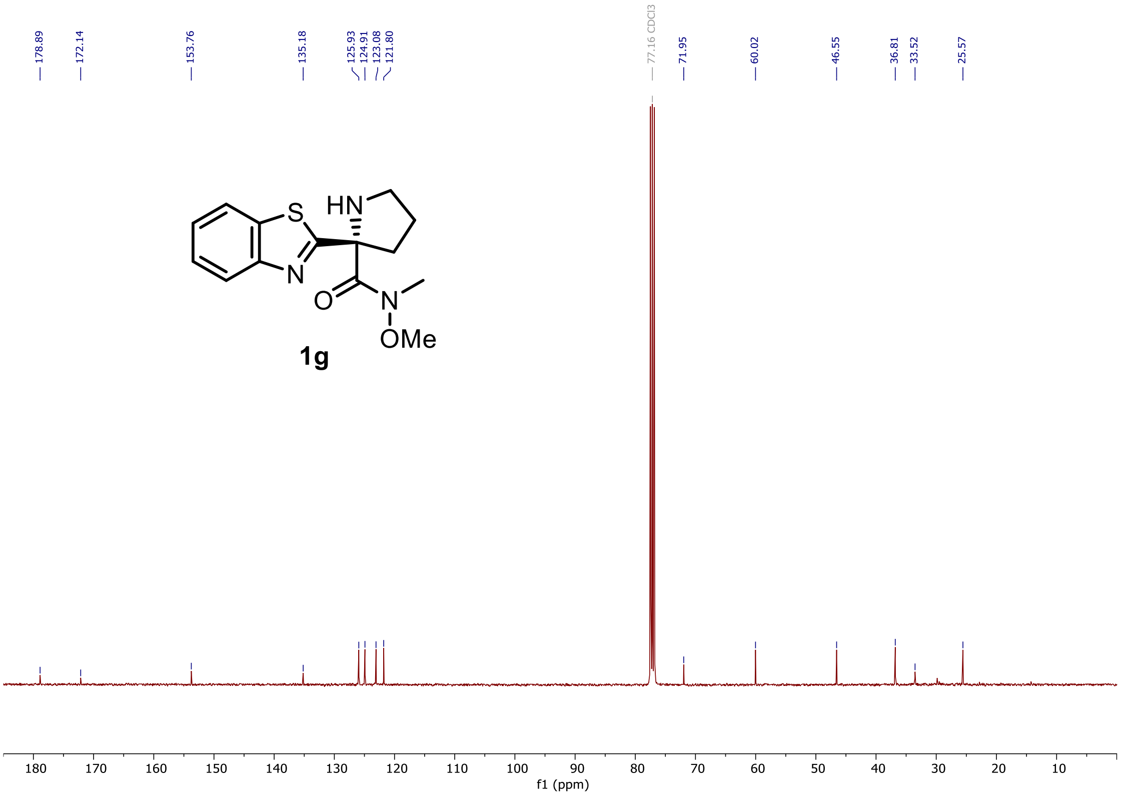

Copy of ^{1}H NMR Spectrum (500 MHz, $CDCl_3$) of **3h**

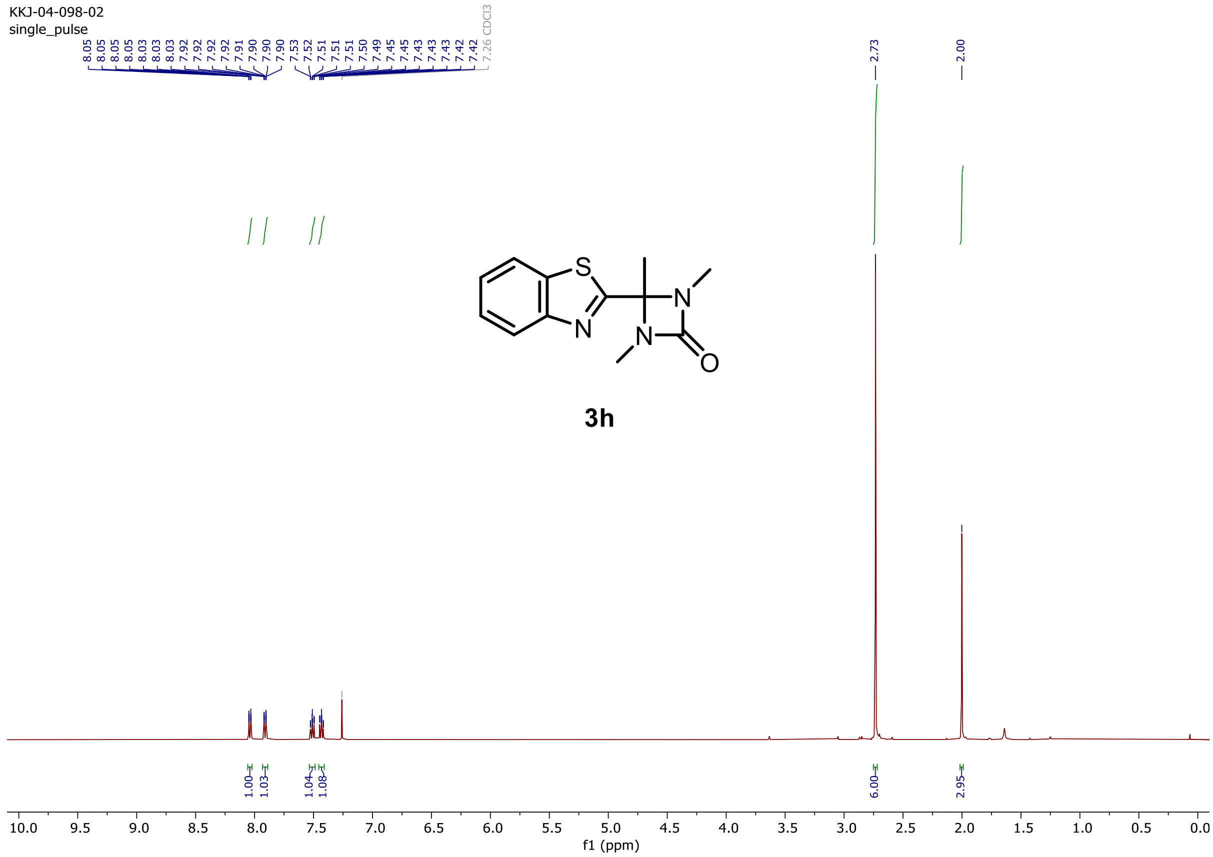

Copy of ^{13}C {^{1}H} NMR Spectrum (126 MHz, $CDCl_3$) of **3h**

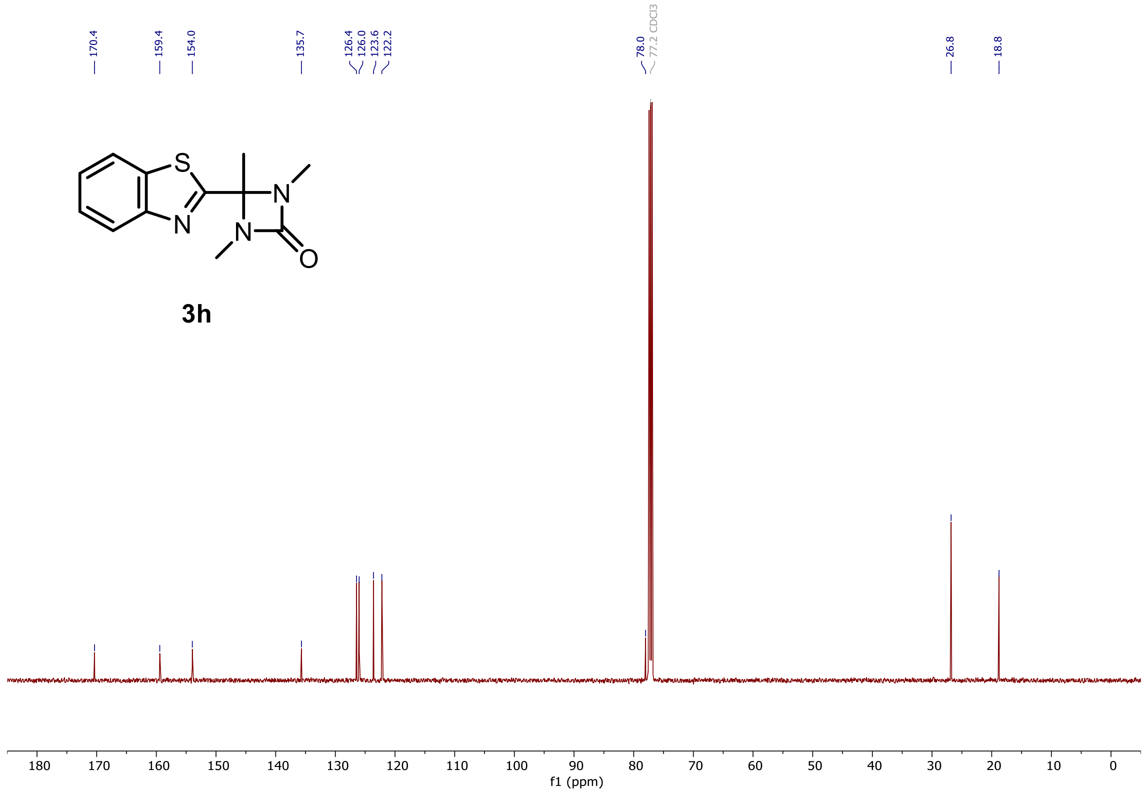

Copy of ^{1}H NMR Spectrum (500 MHz, $CDCl_3$) of **3i**

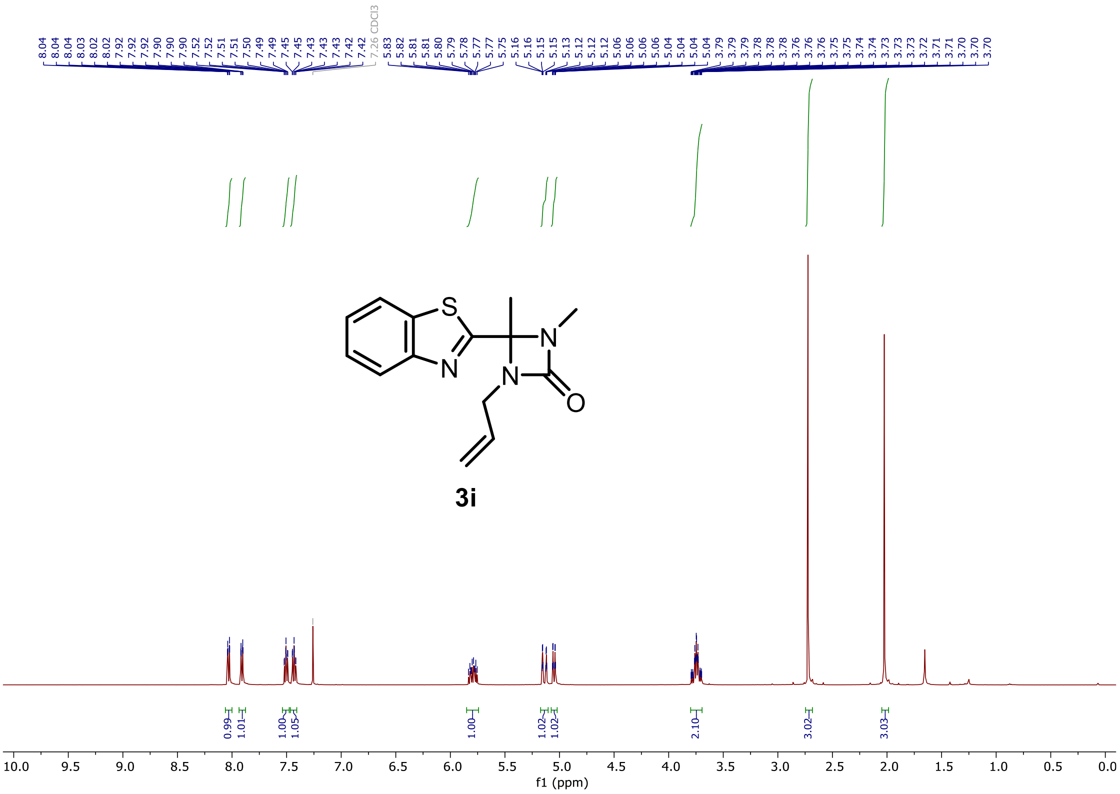

Copy of $^{13}C\{^{1}H\}$ NMR Spectrum (126 MHz, $CDCl_3$) of **3i**

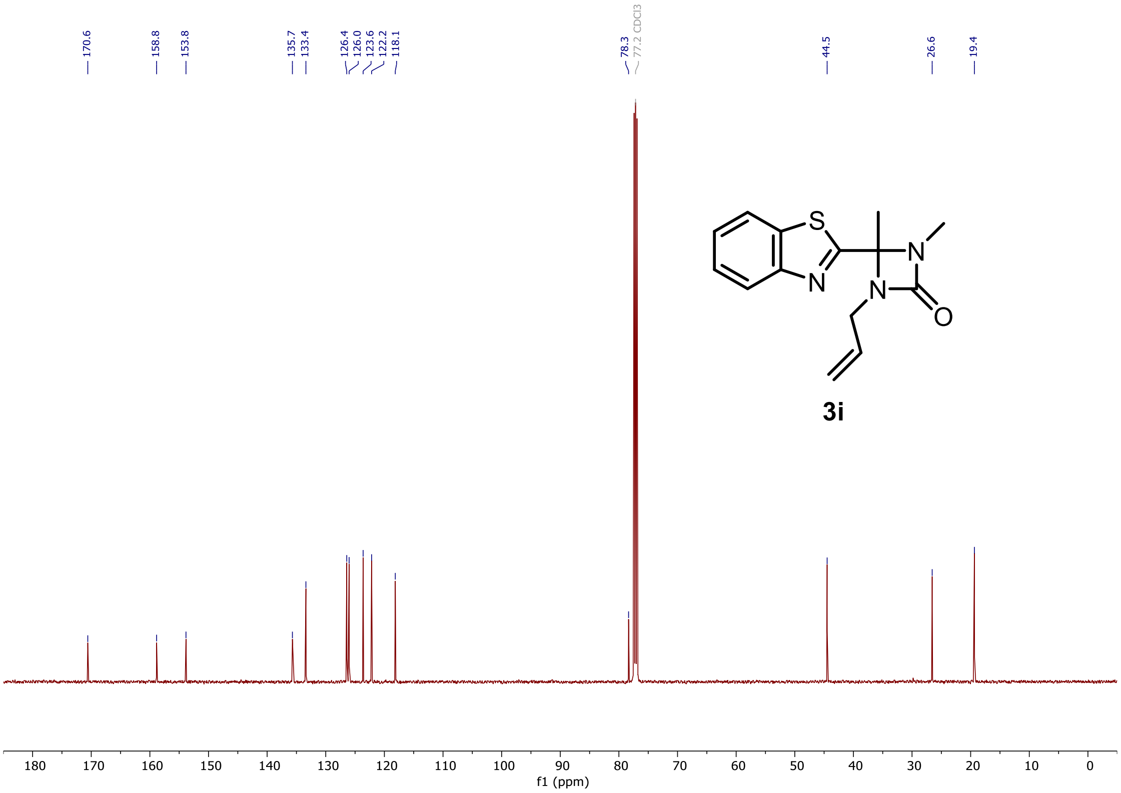

Copy of ^{1}H NMR Spectrum (400 MHz, $CDCl_3$) of **3j**

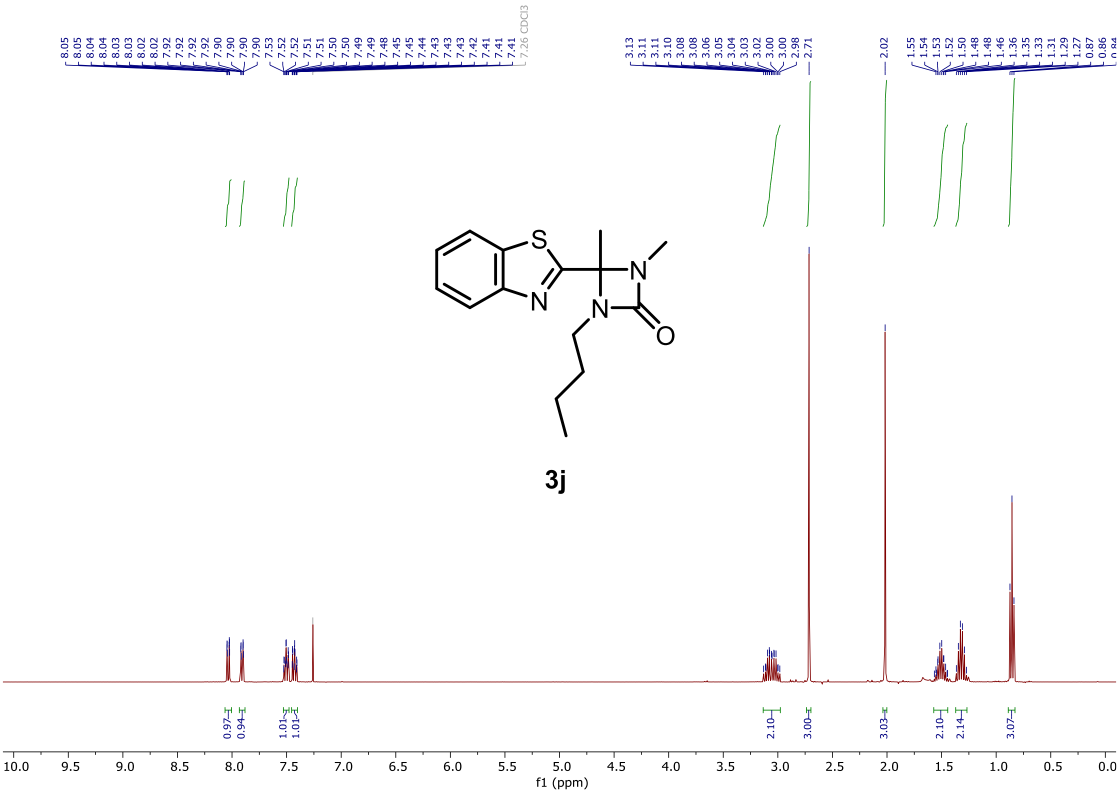

Copy of ^{13}C {^{1}H} NMR Spectrum (126 MHz, $CDCl_3$) of **3j**

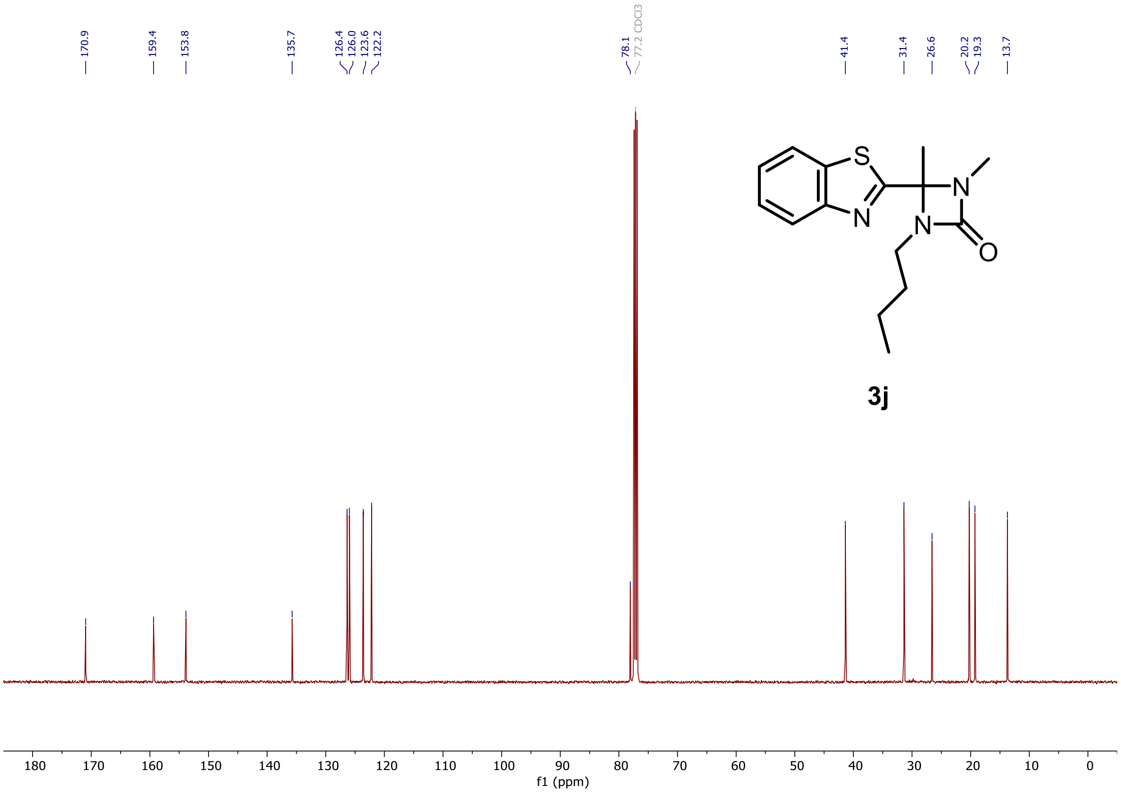

Copy of ^{1}H NMR Spectrum (500 MHz, $CDCl_3$) of **3k**

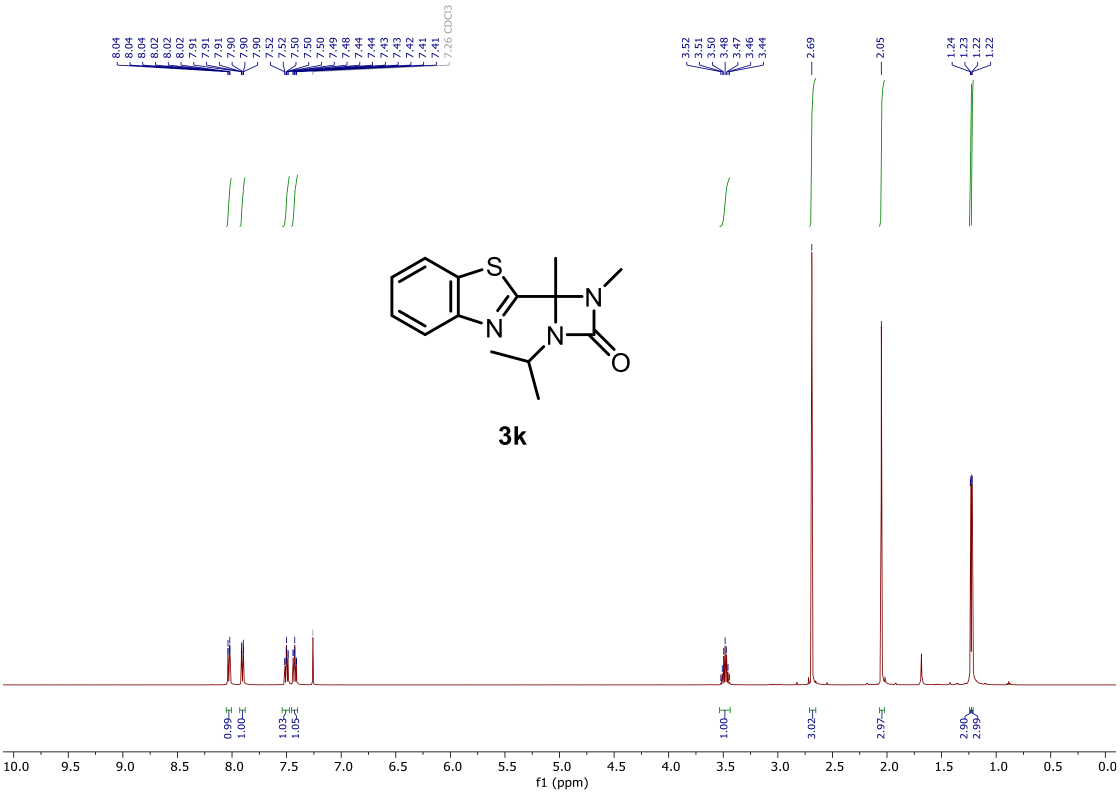

Copy of ^{13}C {^{1}H} NMR Spectrum (126 MHz, $CDCl_3$) of **3k**

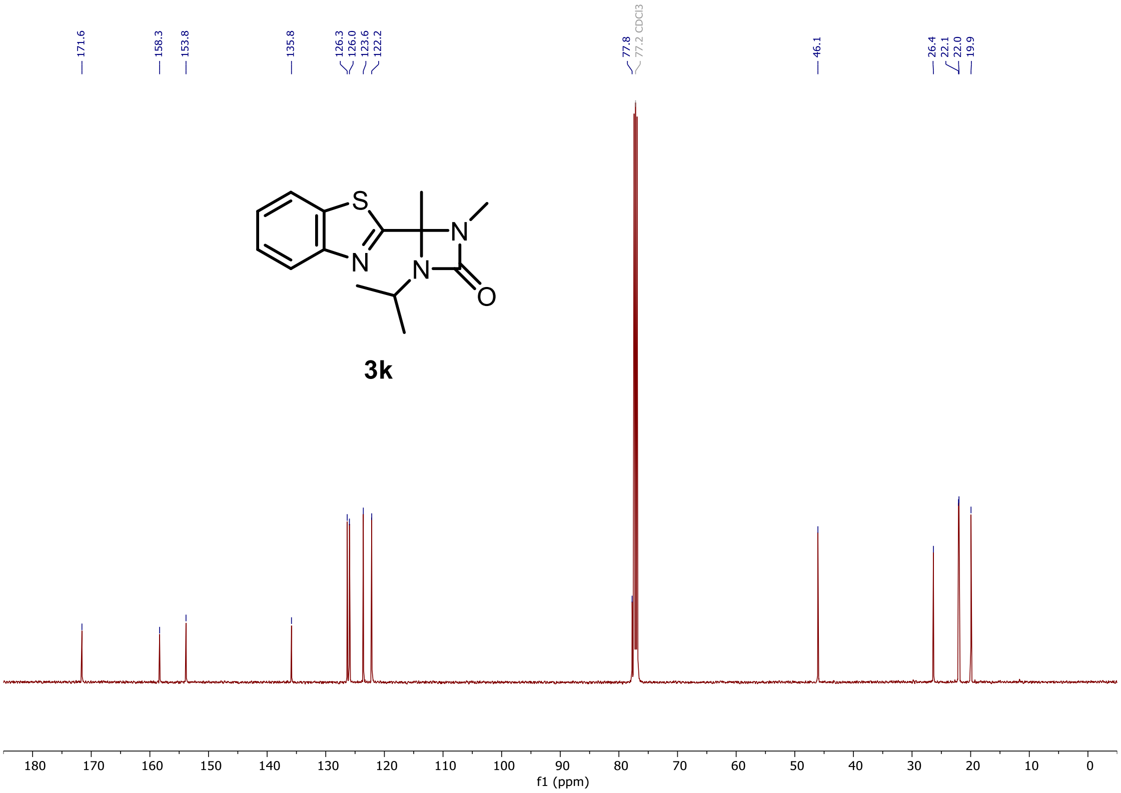

Copy of ^{1}H NMR Spectrum (400 MHz, $CDCl_3$) of **3l**

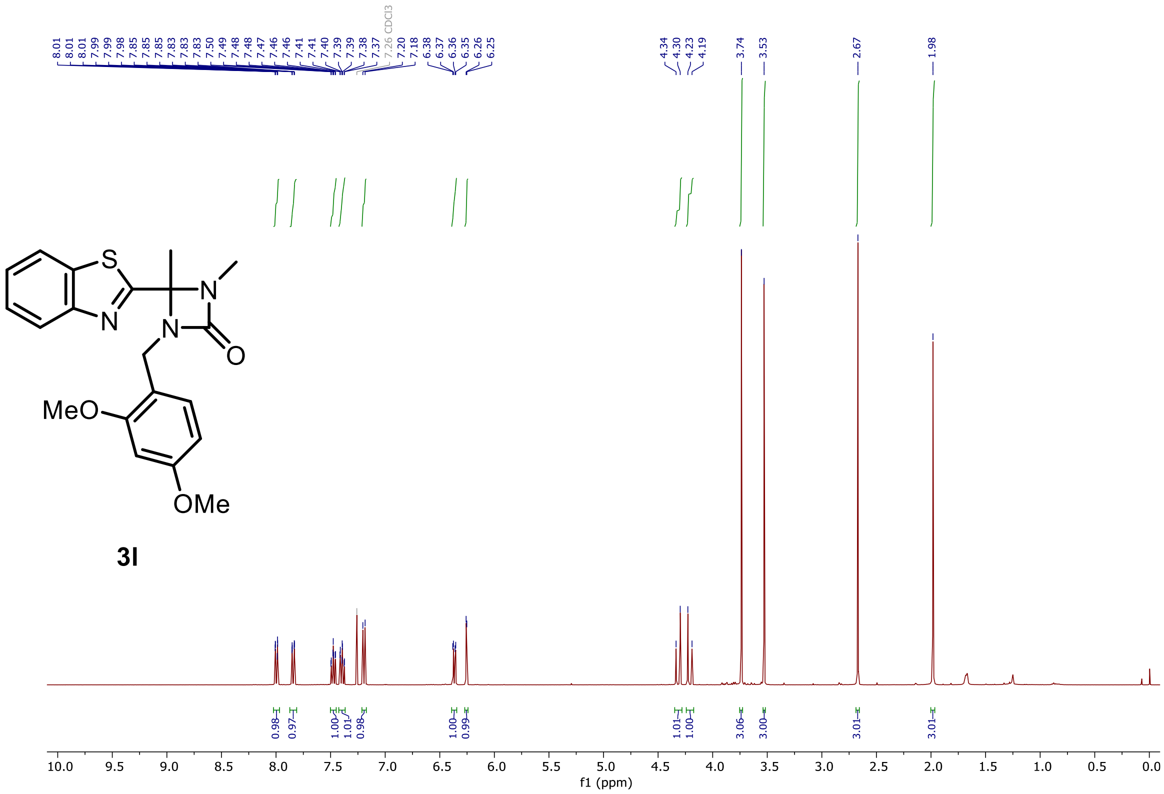

Copy of ^{13}C {^{1}H} NMR Spectrum (101 MHz, $CDCl_3$) of **3l**

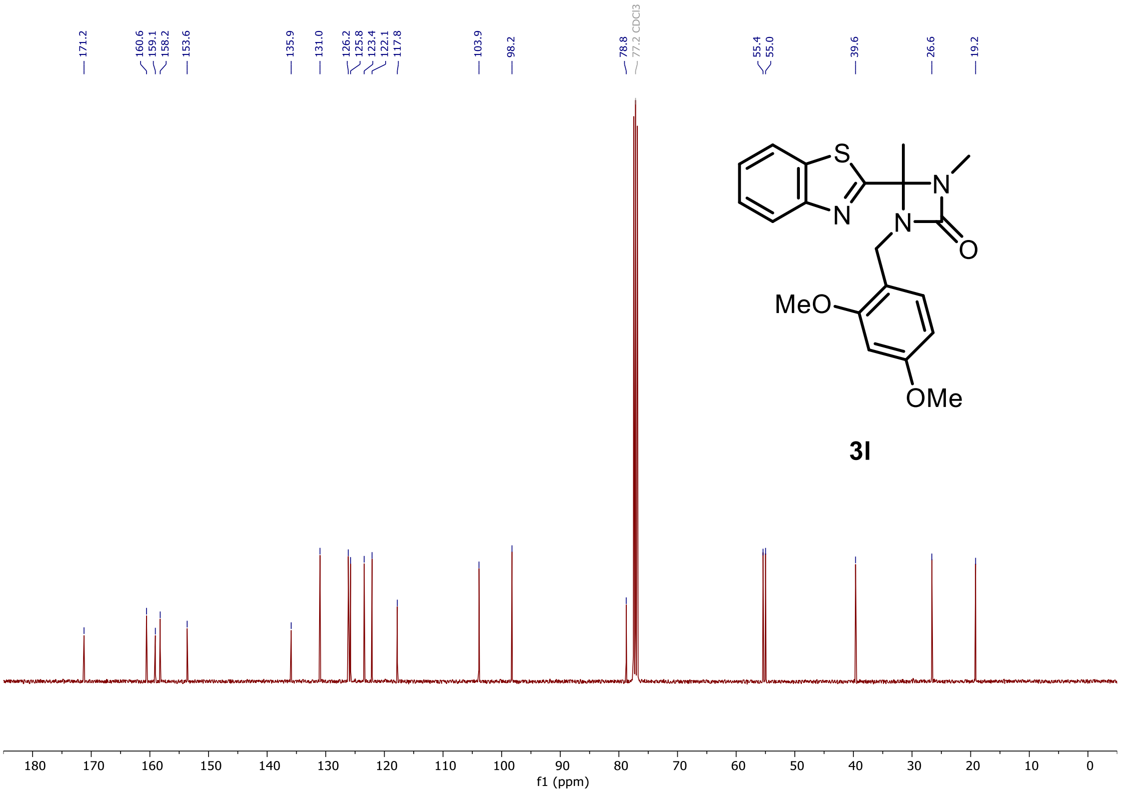

Copy of ^{1}H NMR Spectrum (500 MHz, $CDCl_3$) of **3m**

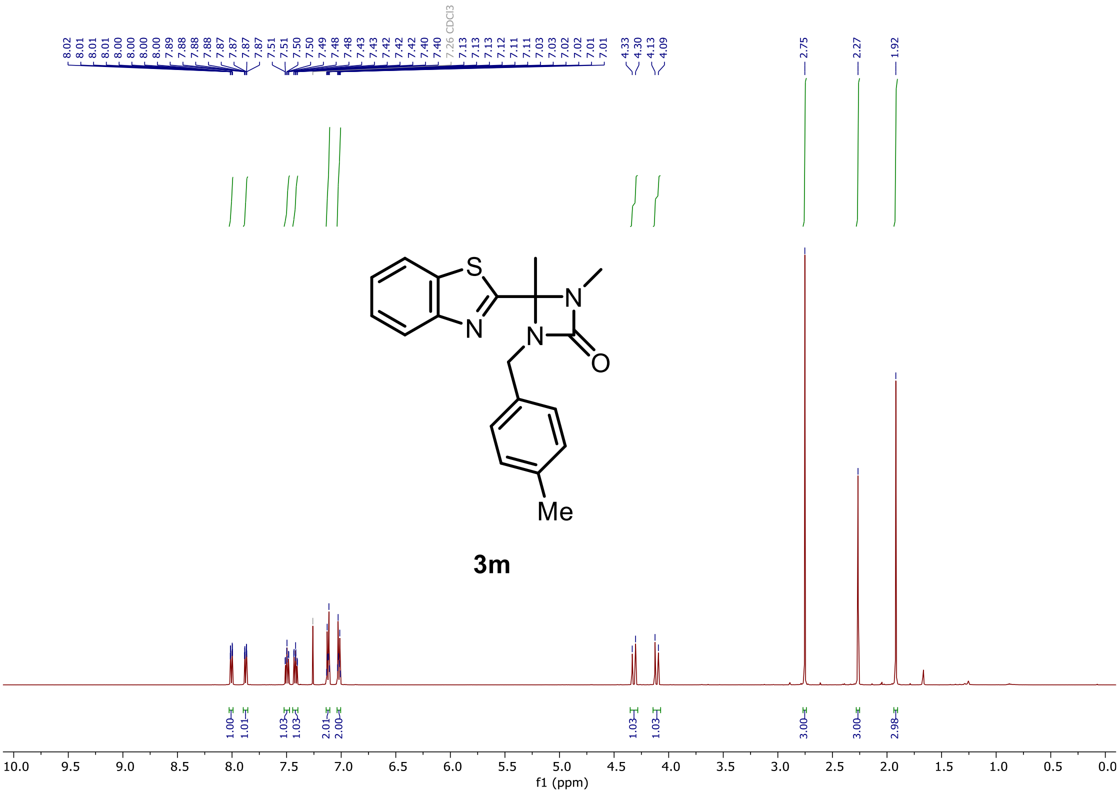

Copy of ^{13}C {^{1}H} NMR Spectrum (126 MHz, $CDCl_3$) of **3m**

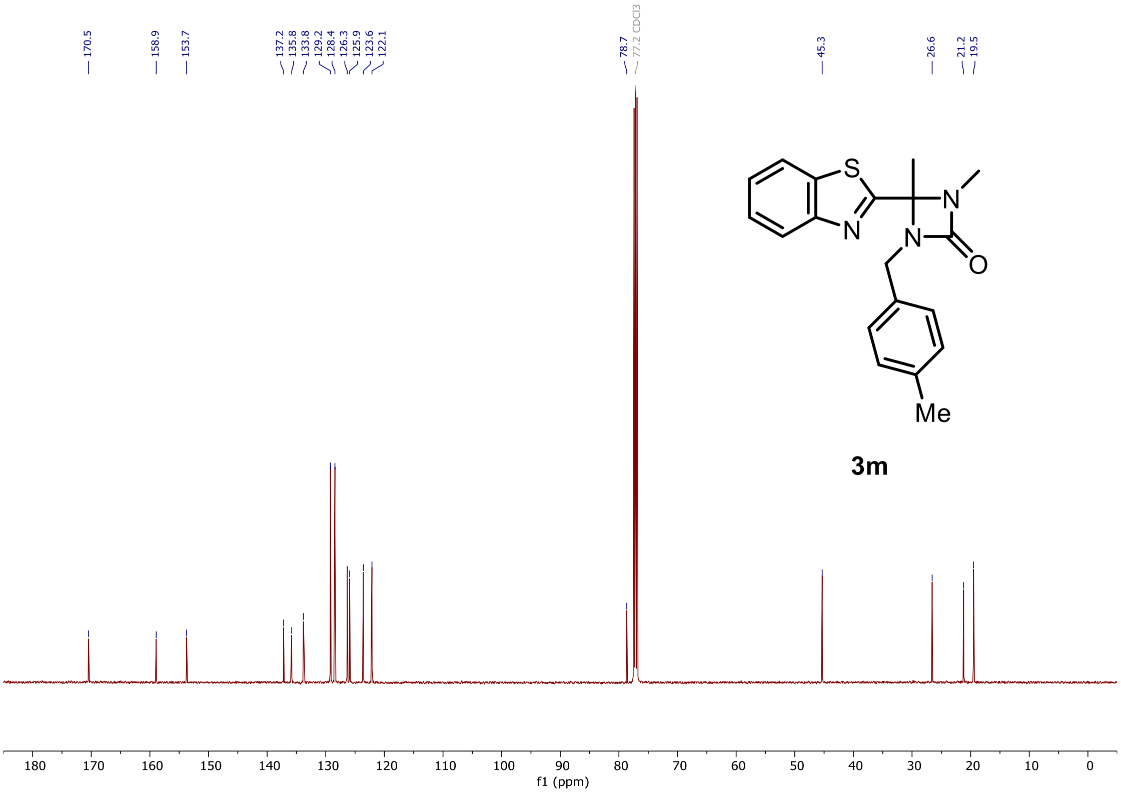

Copy of ^{1}H NMR Spectrum (400 MHz, $CDCl_3$) of **3n**

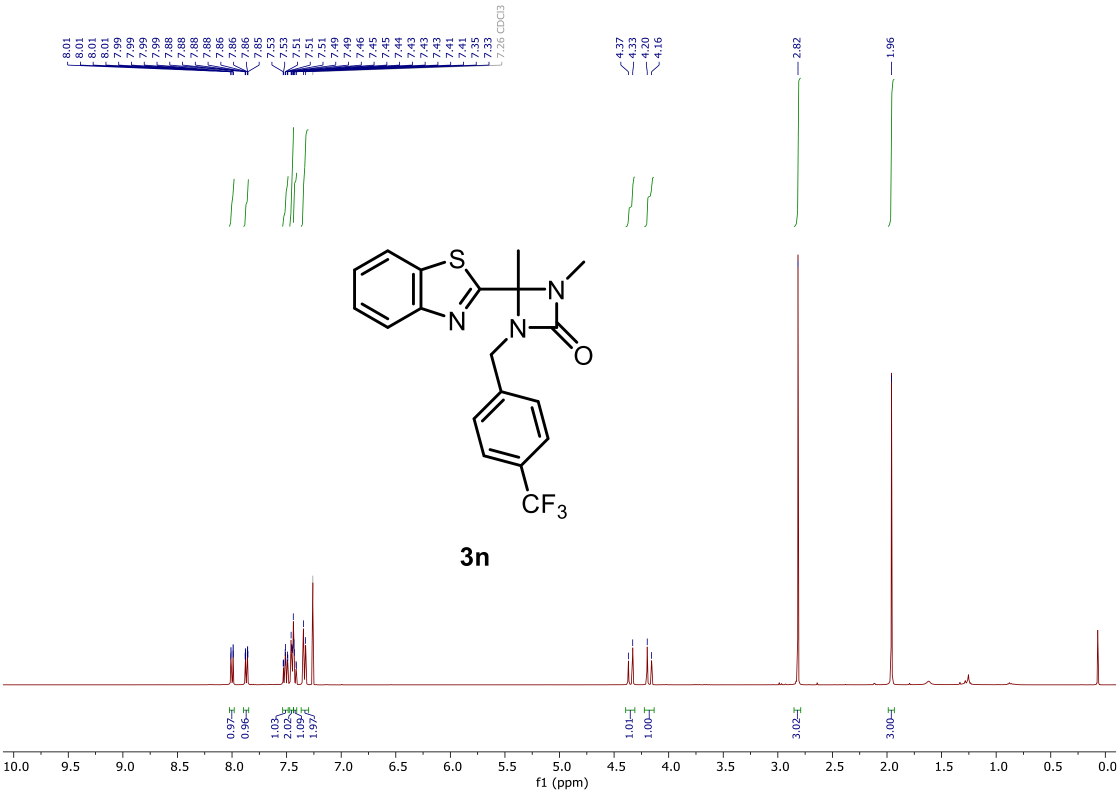

Copy of ^{13}C {^{1}H, ^{19}F} NMR Spectrum (101 MHz, $CDCl_3$) of **3n**

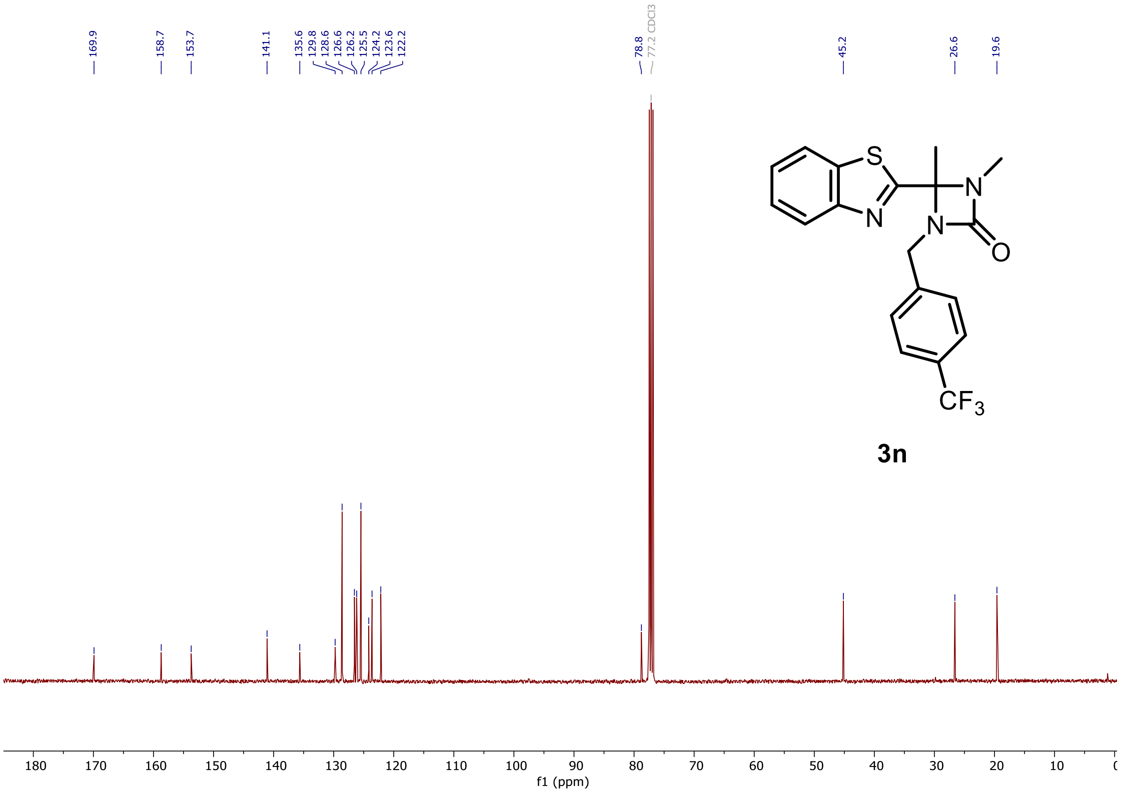

Copy of ^{19}F NMR Spectrum (376 MHz, $CDCl_3$) of **3n**

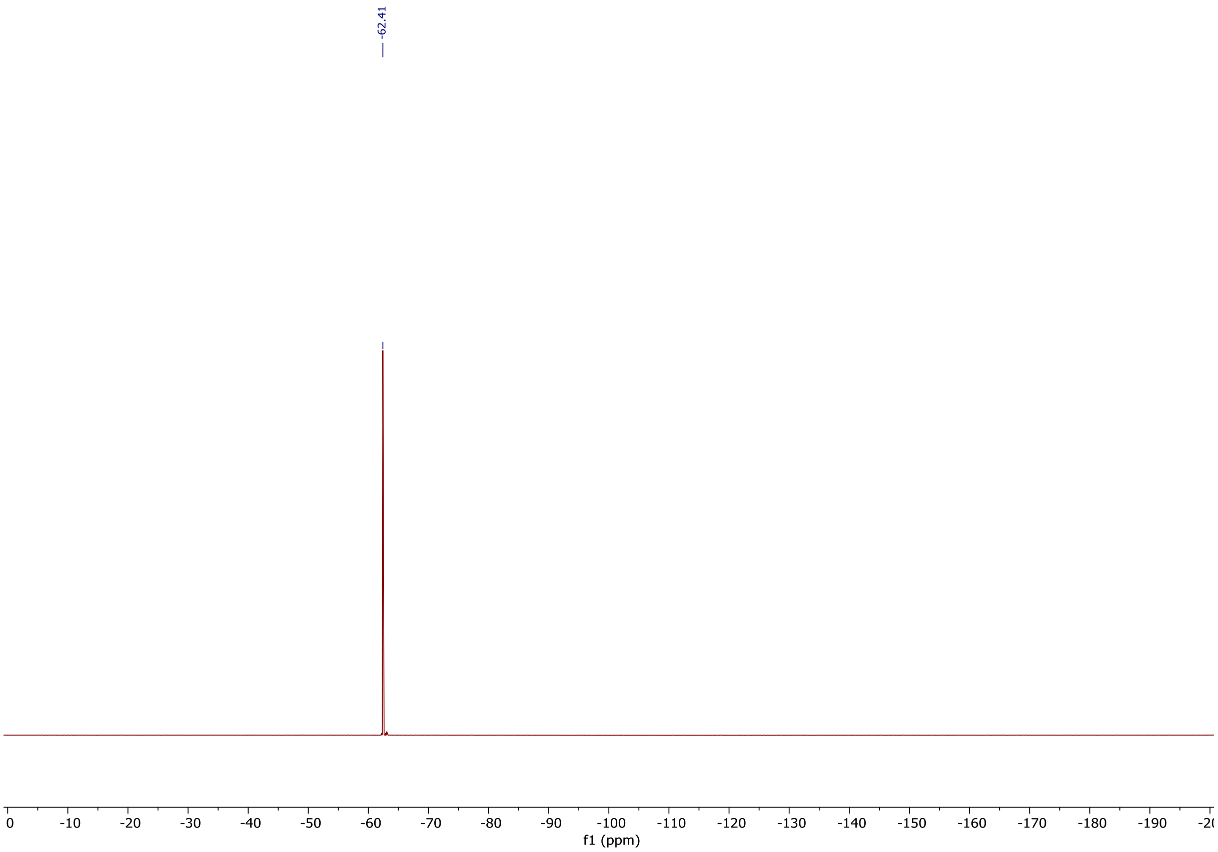

Copy of ^{1}H NMR Spectrum (500 MHz, $CDCl_3$) of **1p**

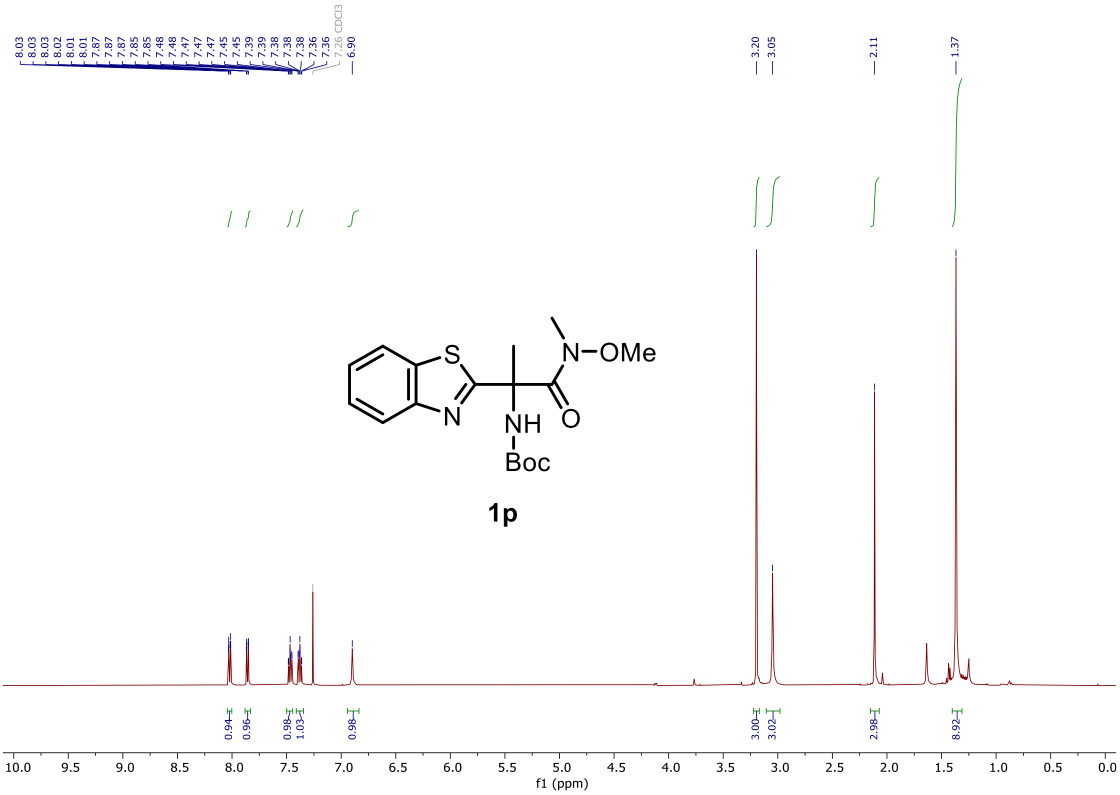

Copy of^{13}C {^{1}H} NMR Spectrum (126 MHz, $CDCl_3$) of **1p**

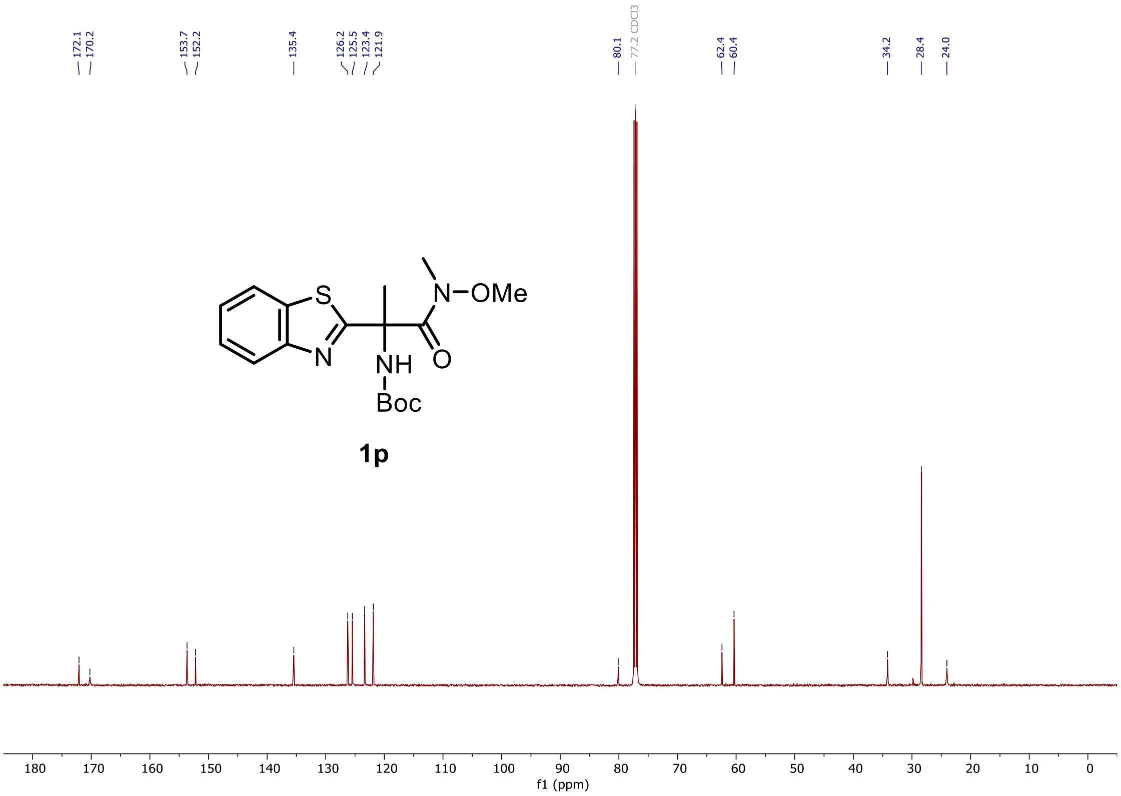

Copy of ^{1}H NMR Spectrum (500 MHz, $CDCl_3$) of **1q**

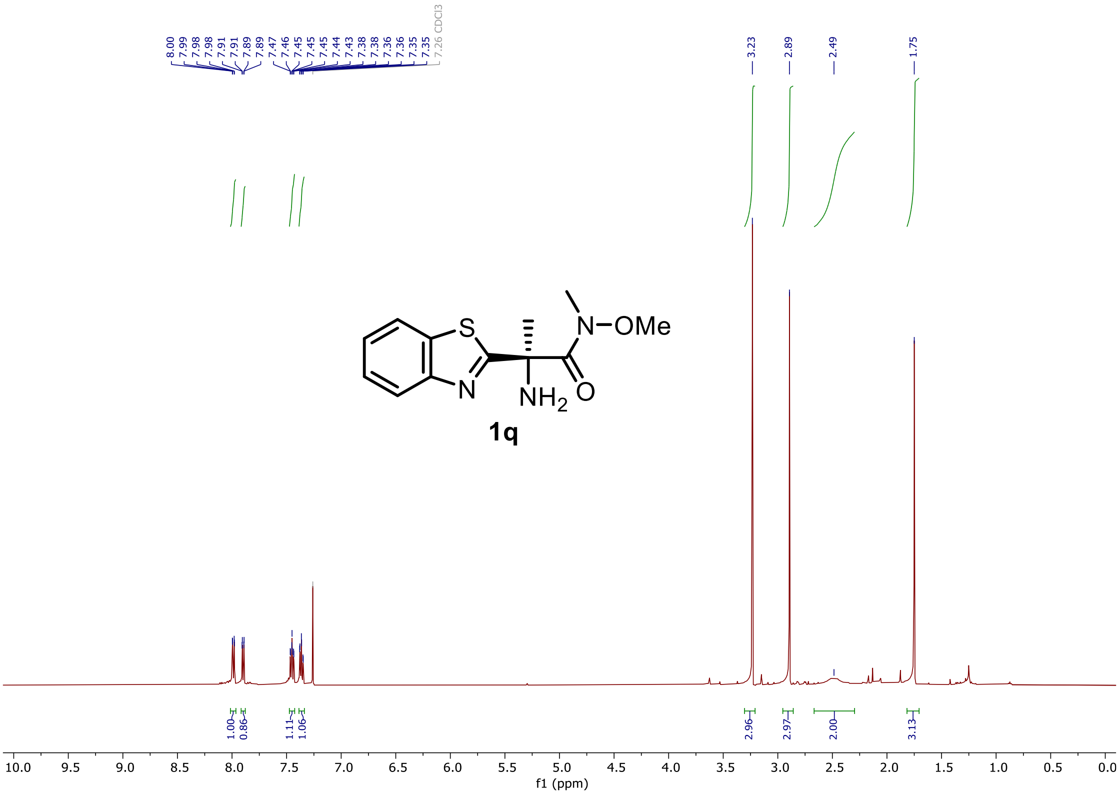

Copy of ^{13}C {^{1}H} NMR Spectrum (126 MHz, $CDCl_3$) of **1q**

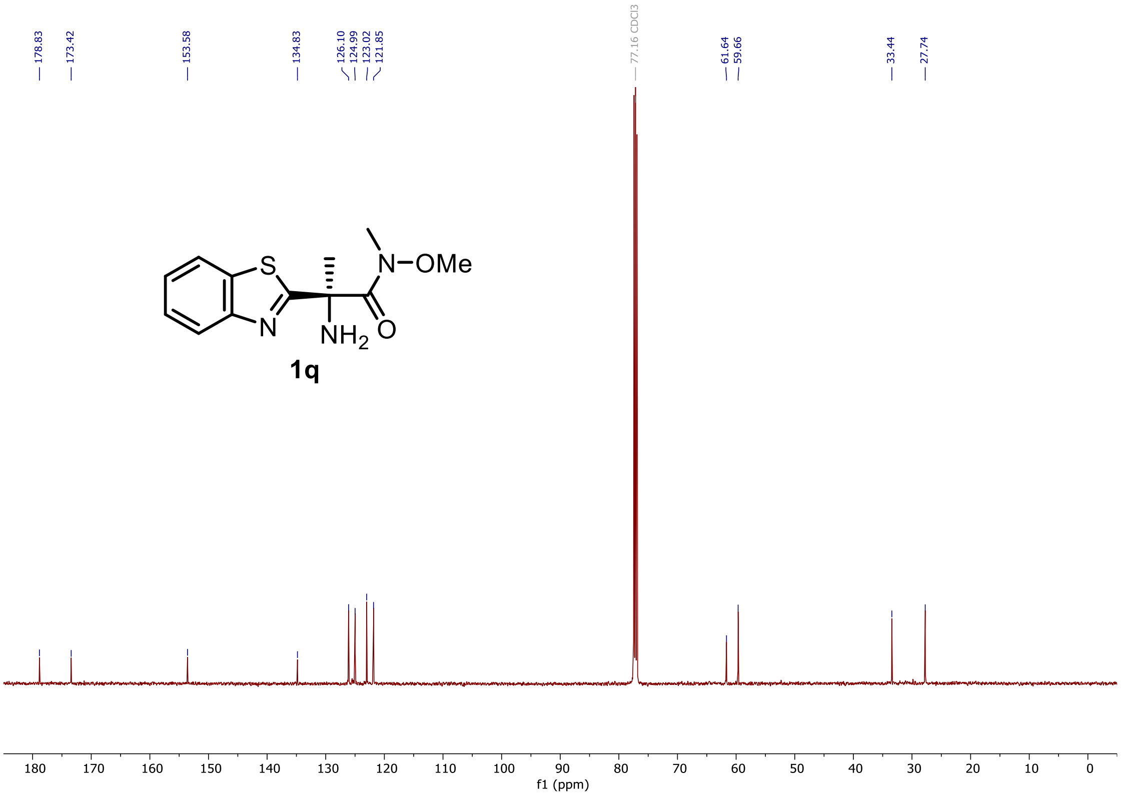

Copy of ^{1}H NMR Spectrum (400 MHz, $CDCl_3$) of **8r**

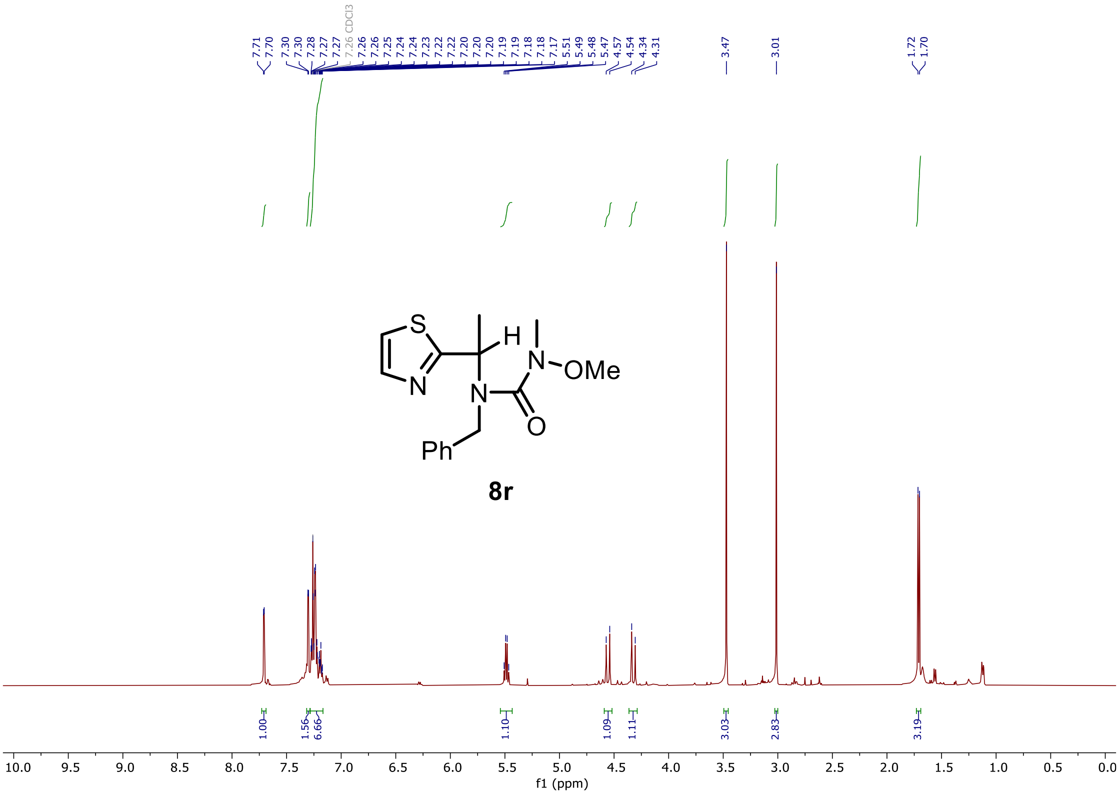

Copy of ^{13}C {^{1}H} NMR Spectrum (126 MHz, $CDCl_3$) of **8r**

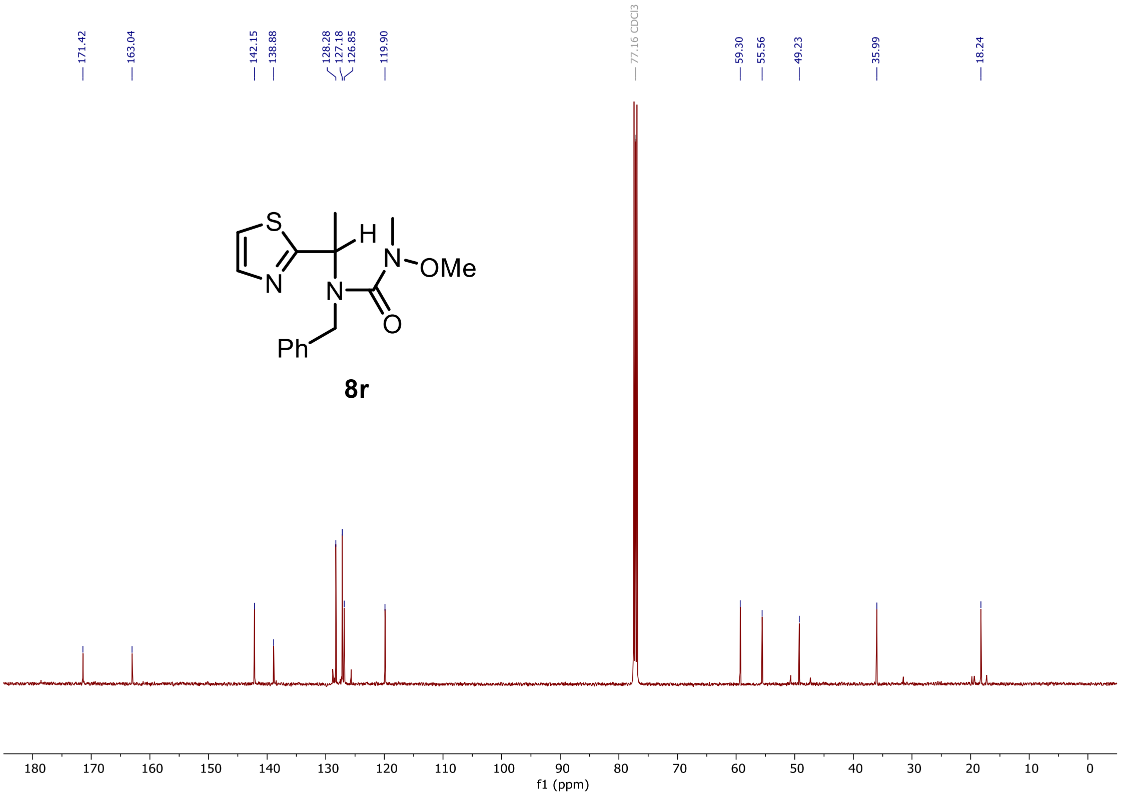

Copy of ^{1}H NMR Spectrum (500 MHz, $CDCl_3$) of **8s**

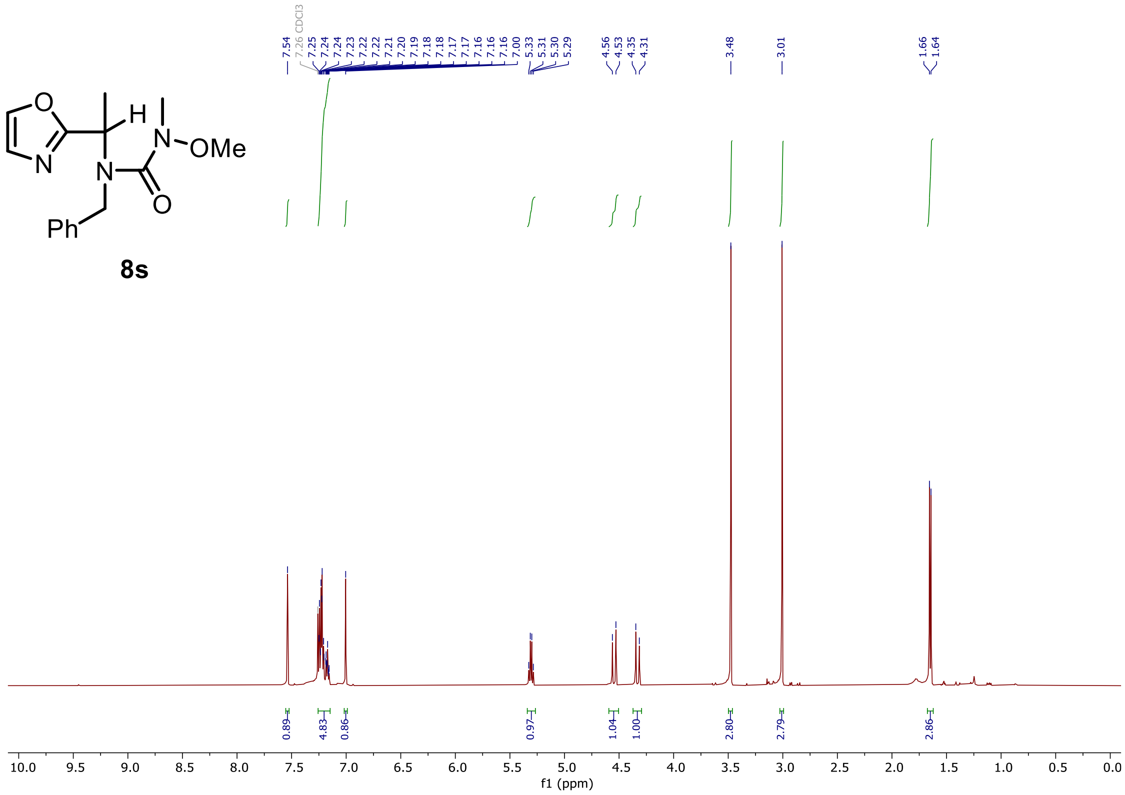

8s

Copy of ^{13}C {^{1}H} NMR Spectrum (126 MHz, $CDCl_3$) of **8s**

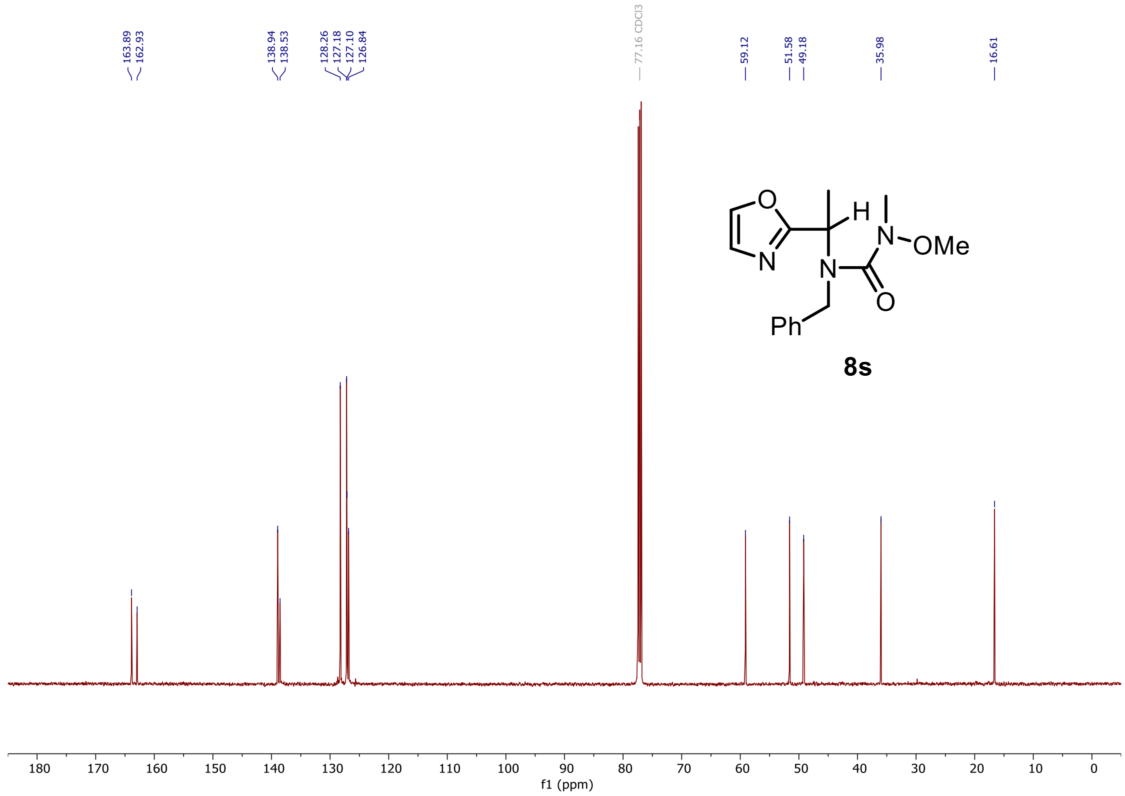

8s

Copy of ^{1}H NMR Spectrum (500 MHz, $CDCl_3$) of **3w**

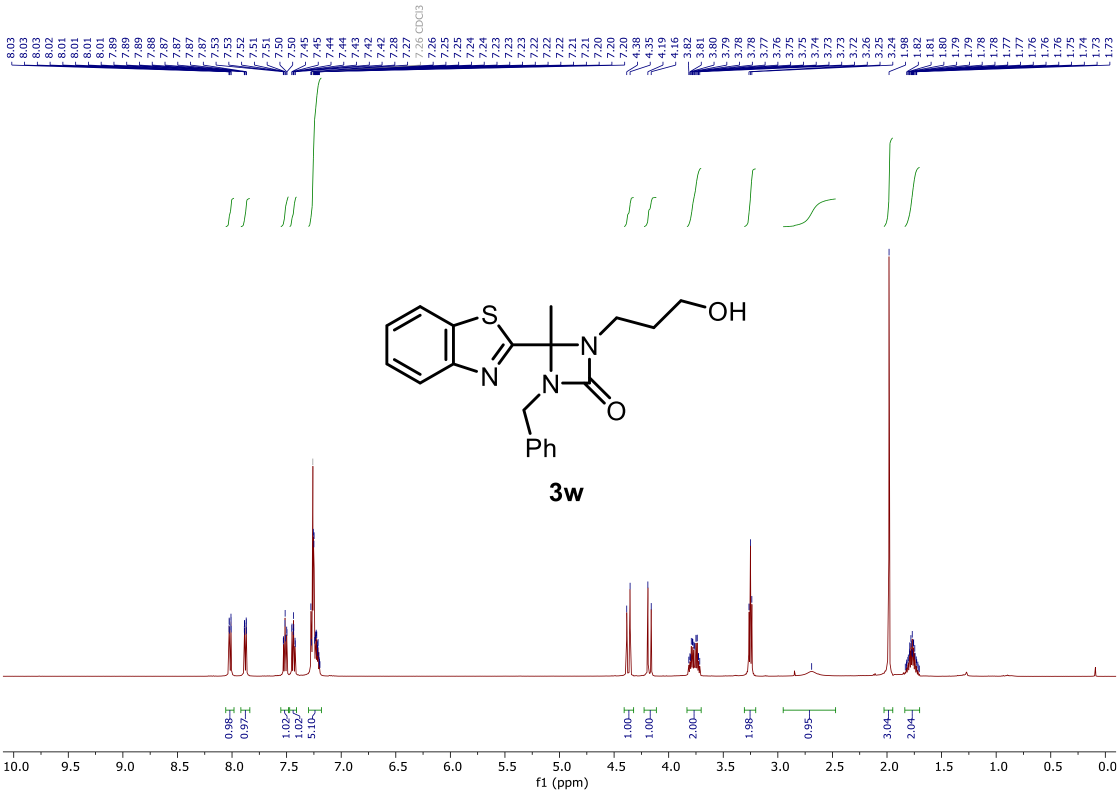

Copy of ^{13}C {^{1}H} NMR Spectrum (126 MHz, $CDCl_3$) of **3w**

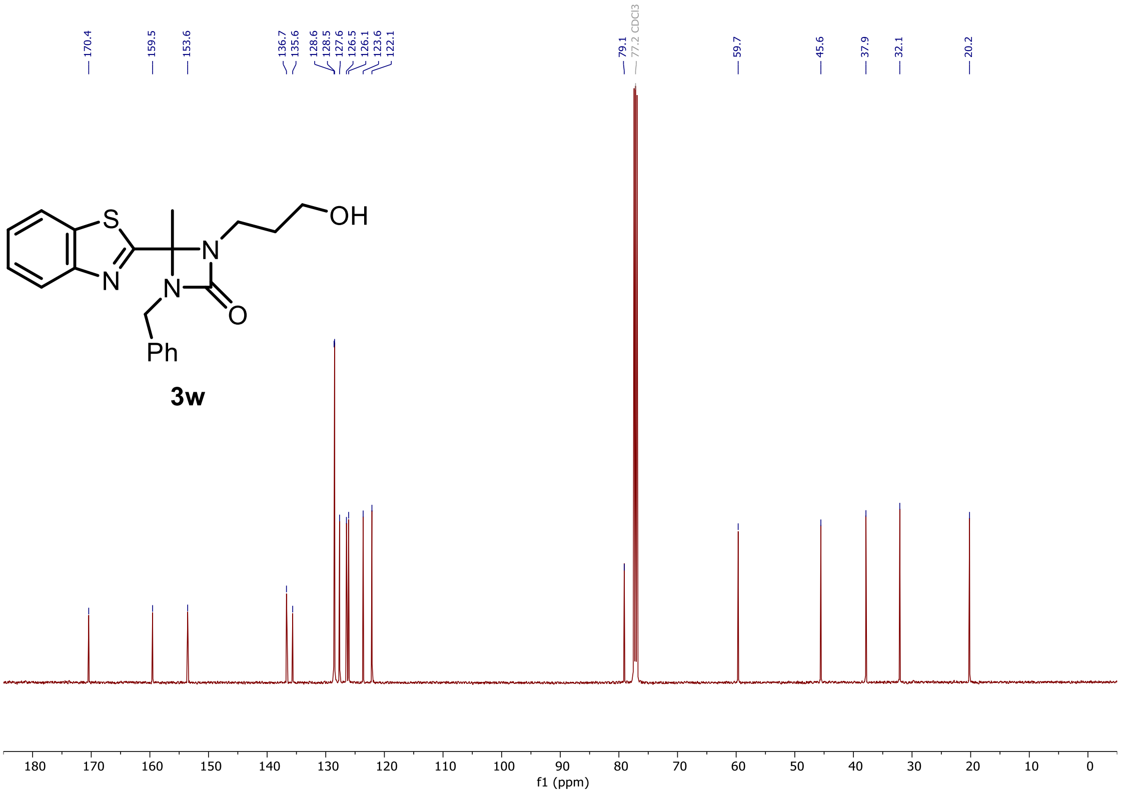

Copy of ^{1}H NMR Spectrum (500 MHz, $CDCl_3$) of **3x**

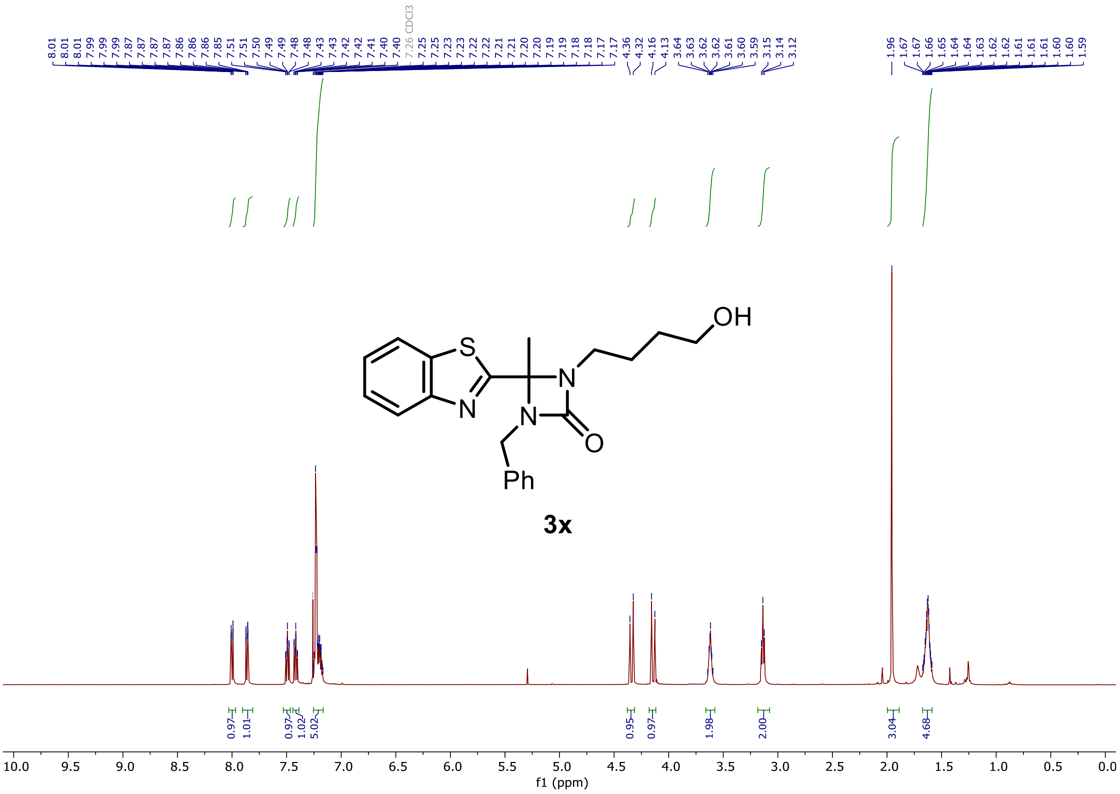

Copy of ^{13}C {^{1}H} NMR Spectrum (126 MHz, $CDCl_3$) of **3x**

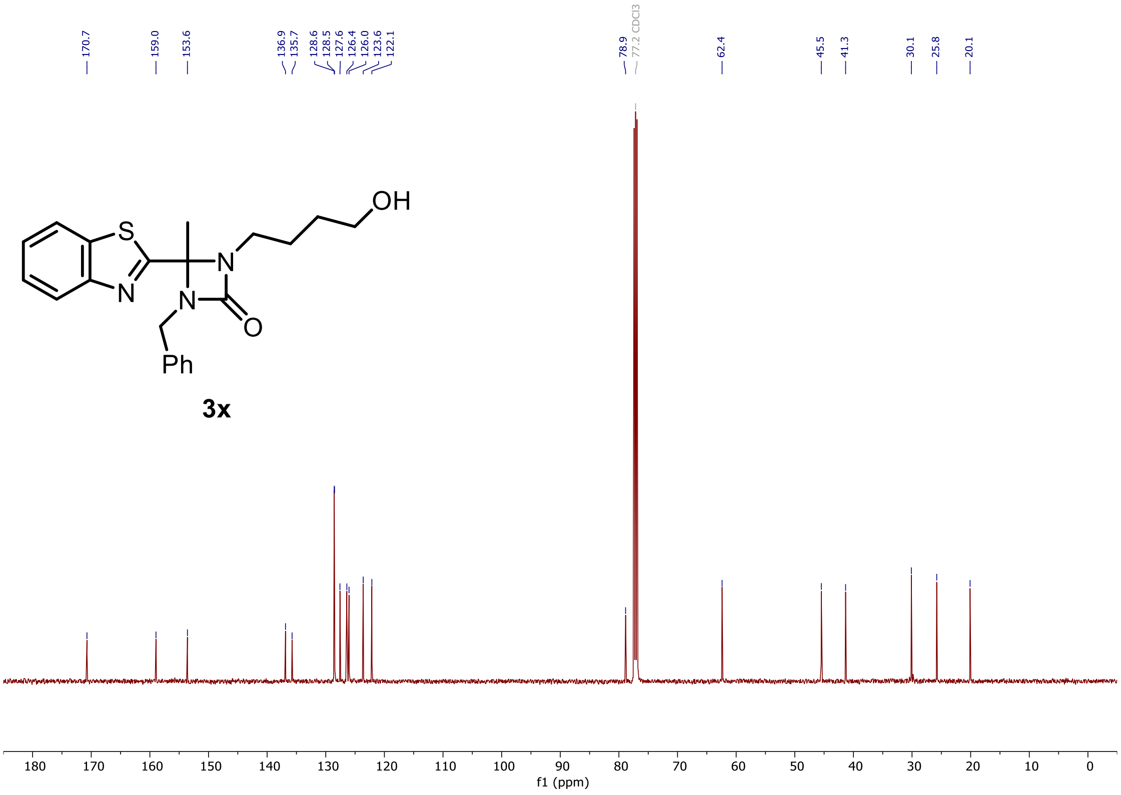

Copy of ^{1}H NMR Spectrum (500 MHz, $CDCl_3$) of **3y**

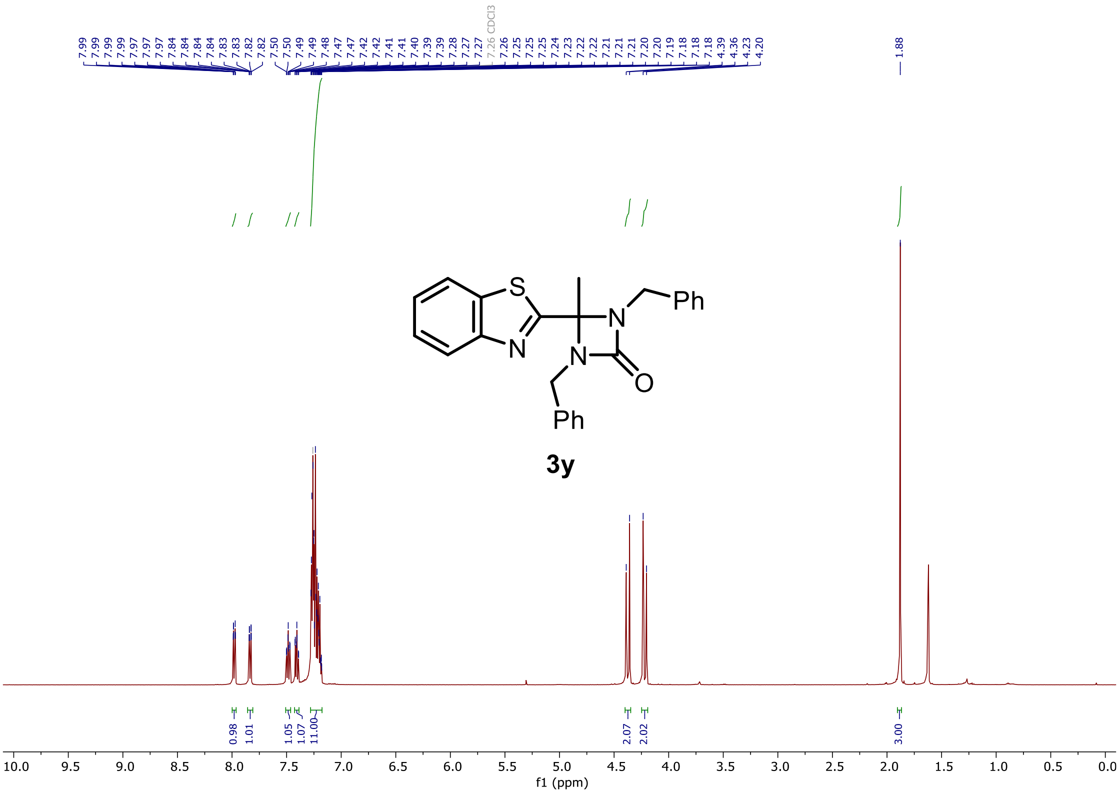

Copy of ^{13}C {^{1}H} NMR Spectrum (126 MHz, $CDCl_3$) of **3y**

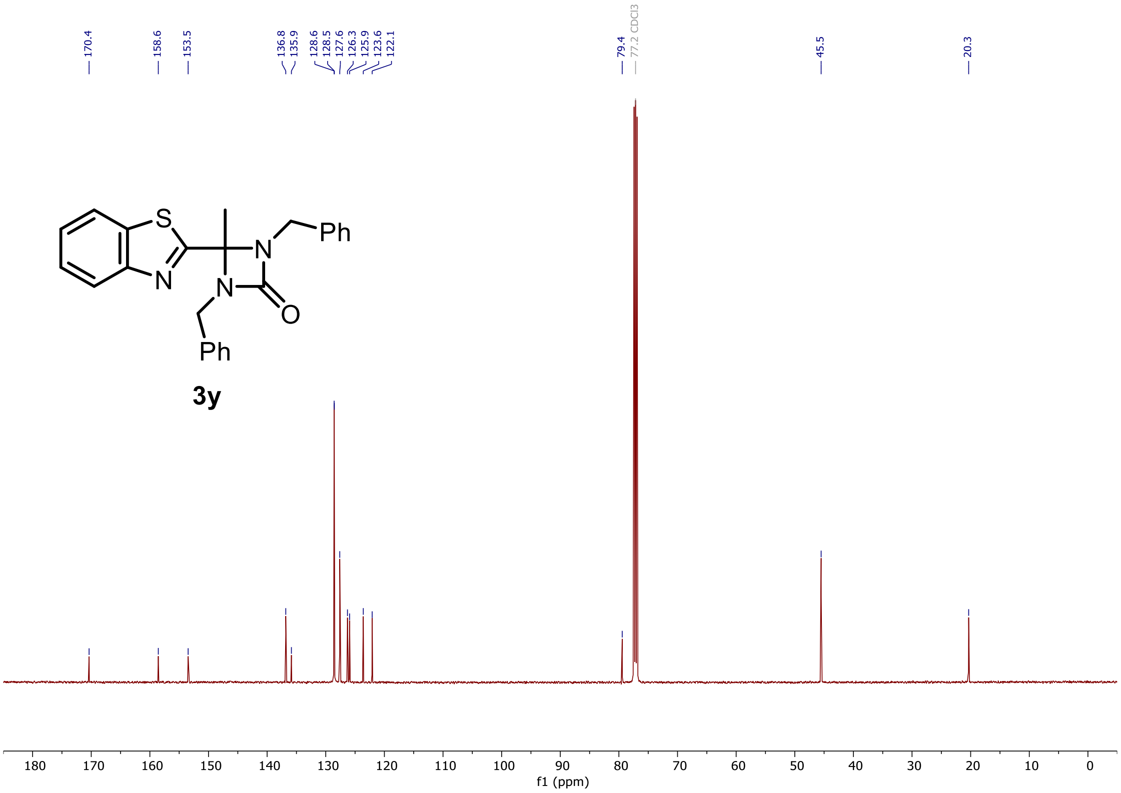

Copy of ^{1}H NMR Spectrum (500 MHz, $CDCl_3$) of **3z**

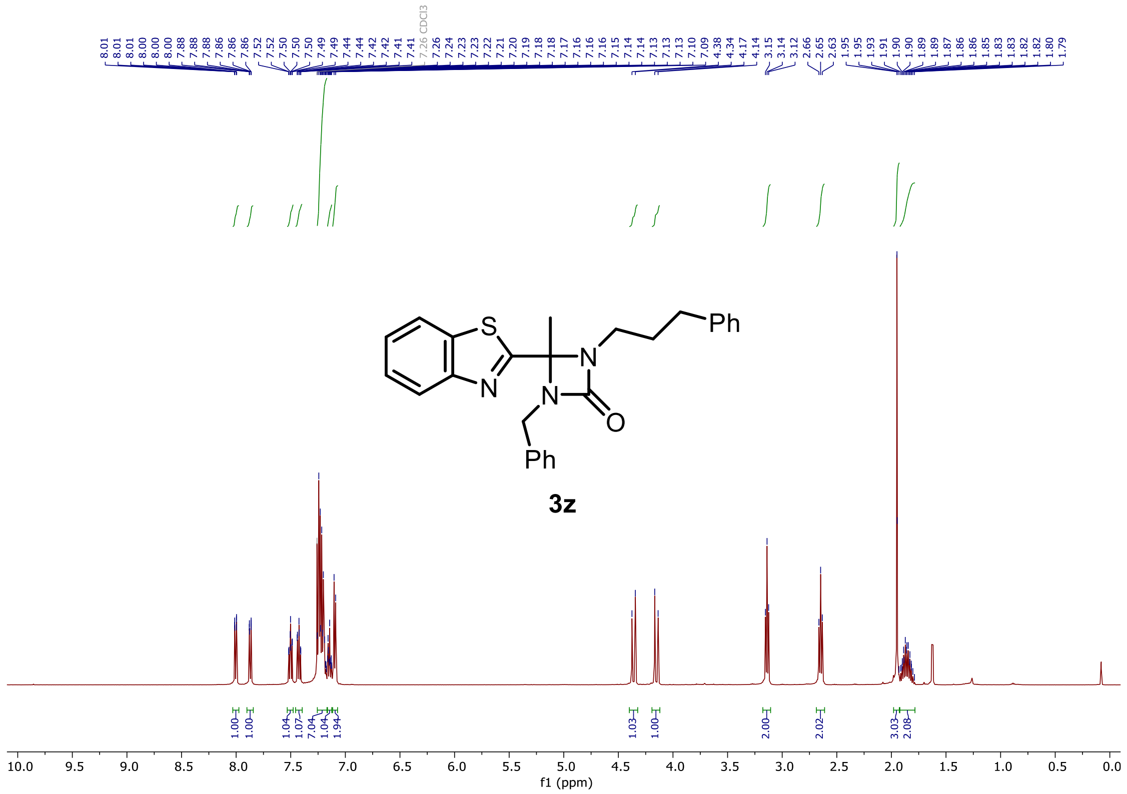

Copy of ^{13}C {^{1}H} NMR Spectrum (126 MHz, $CDCl_3$) of **3z**

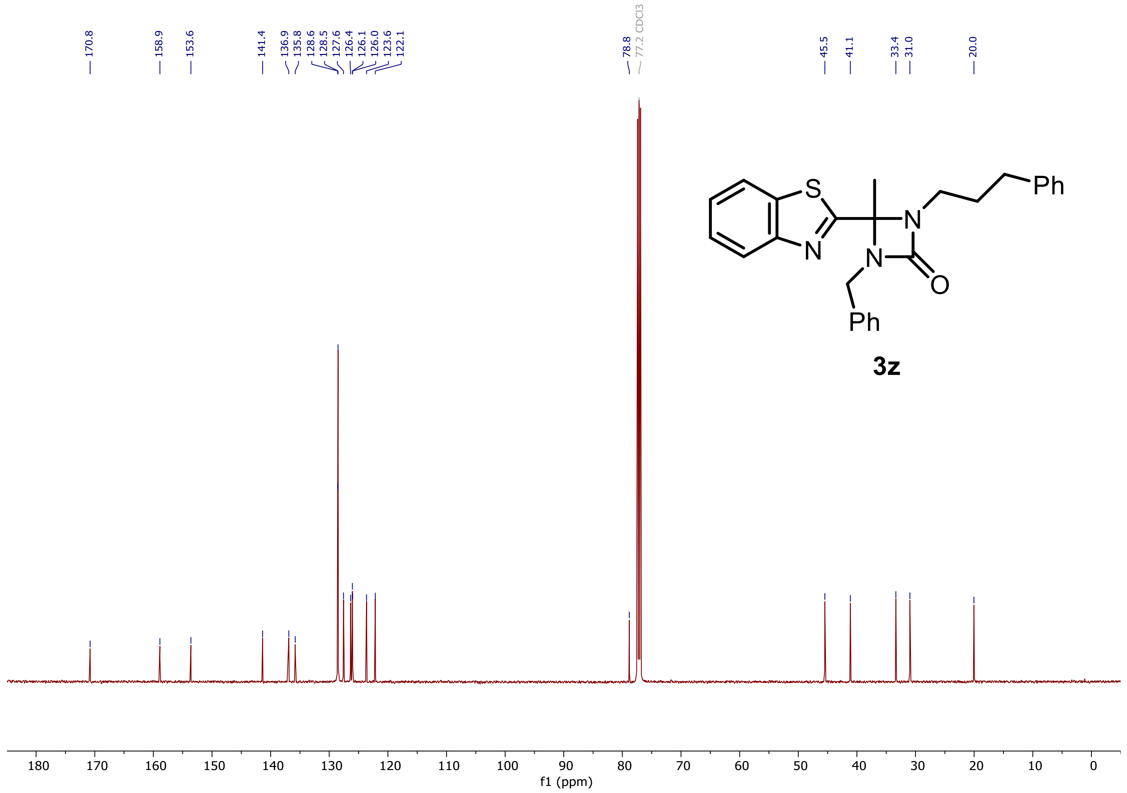

Copy of ^{1}H NMR Spectrum (400 MHz, $CDCl_3$) of **3aa**

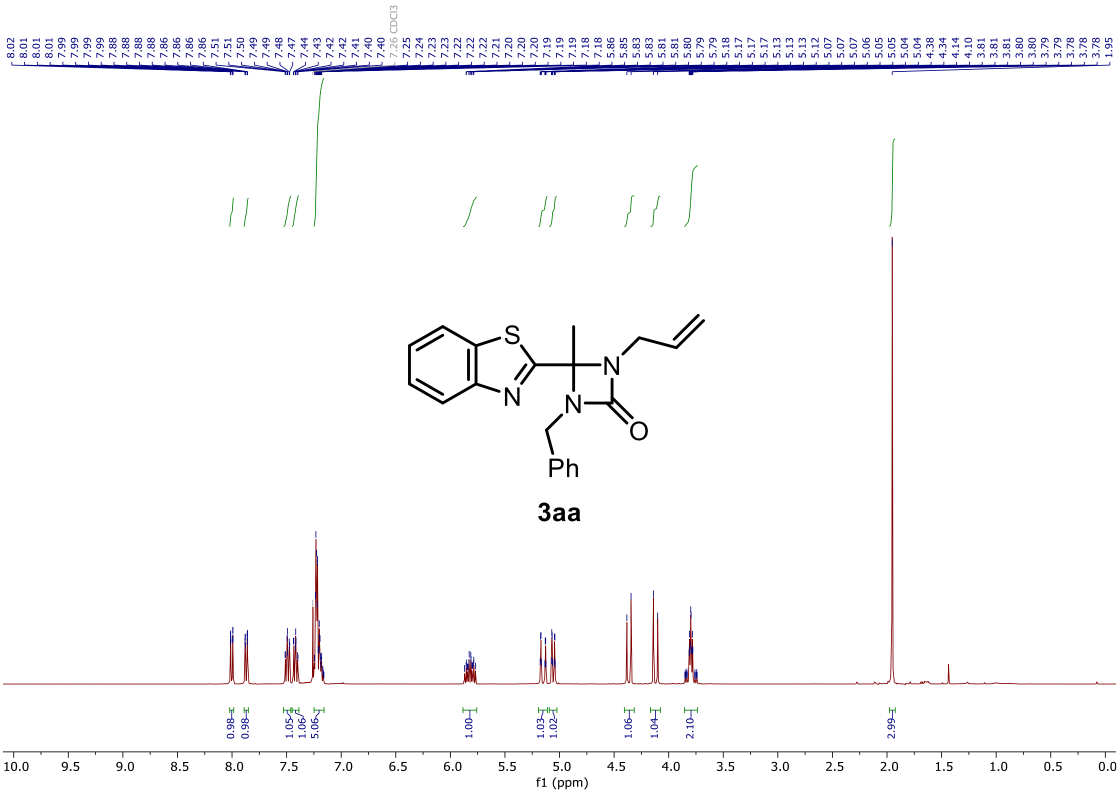

Copy of ^{13}C {^{1}H} NMR Spectrum (101 MHz, $CDCl_3$) of **3aa**

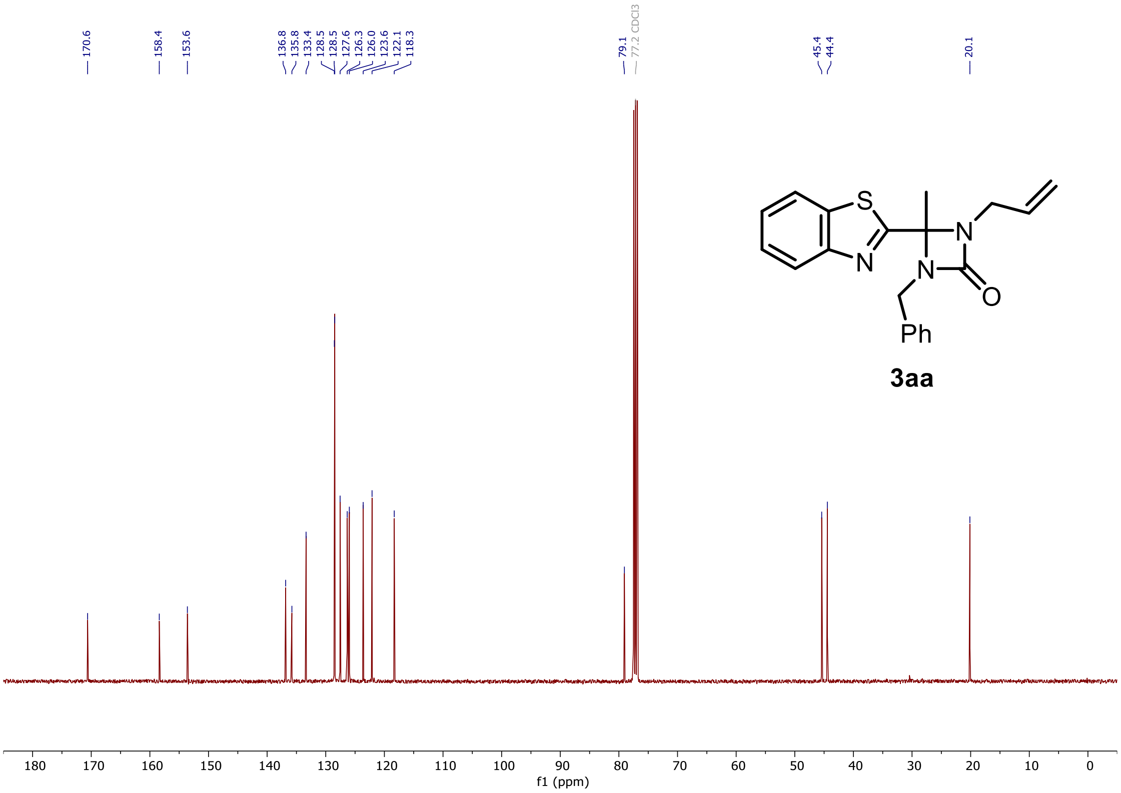

Copy of ^{1}H NMR Spectrum (400 MHz, $CDCl_3$) of **3ab**

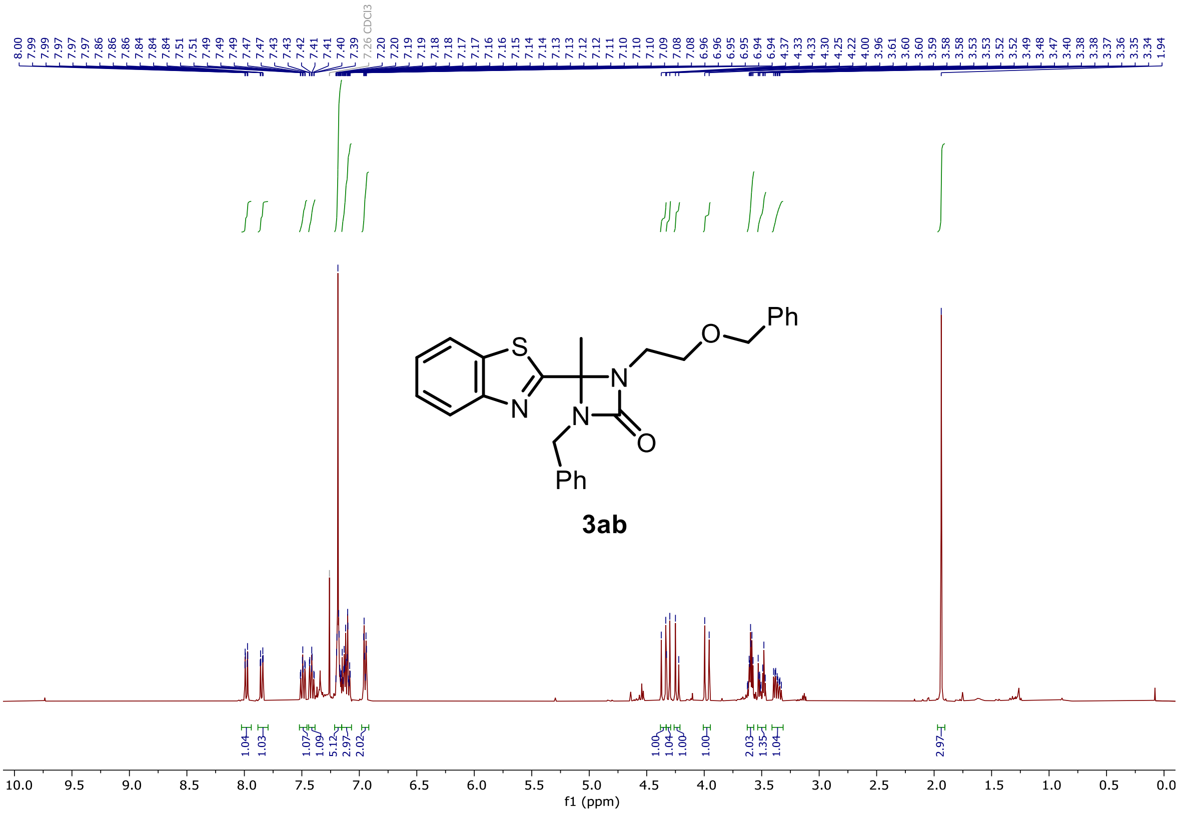

Copy of ^{13}C {^{1}H} NMR Spectrum (126 MHz, $CDCl_3$) of **3ab**

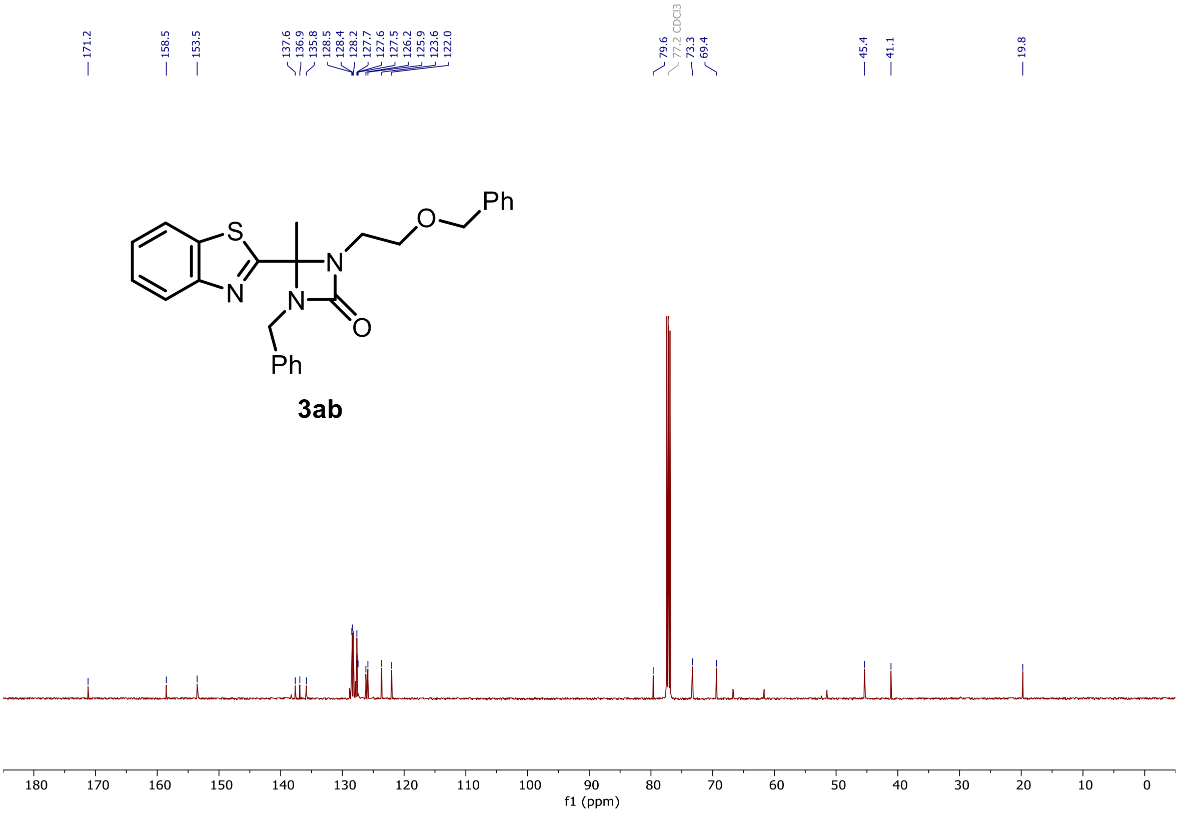

Copy of ^{1}H NMR Spectrum (500 MHz, $CDCl_3$) of **3ad** – major isomer

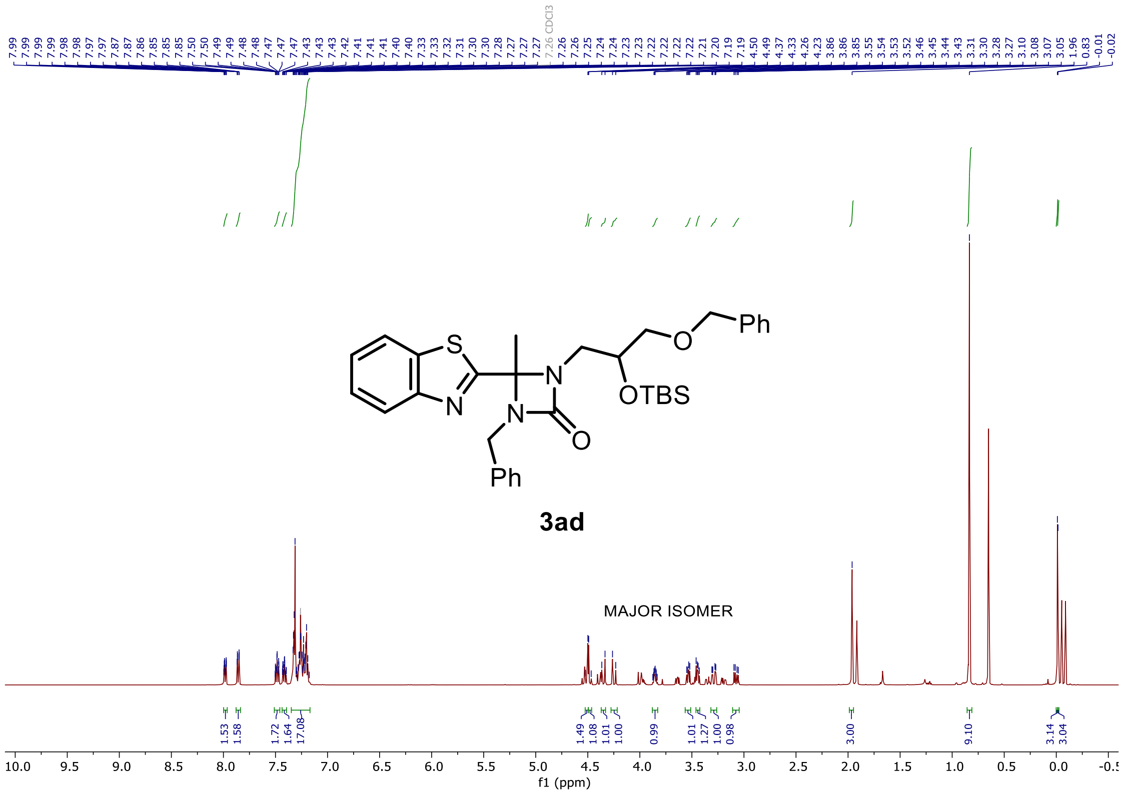

Copy of ^{13}C {^{1}H} NMR Spectrum (126 MHz, $CDCl_3$) of **3ad** – major isomer

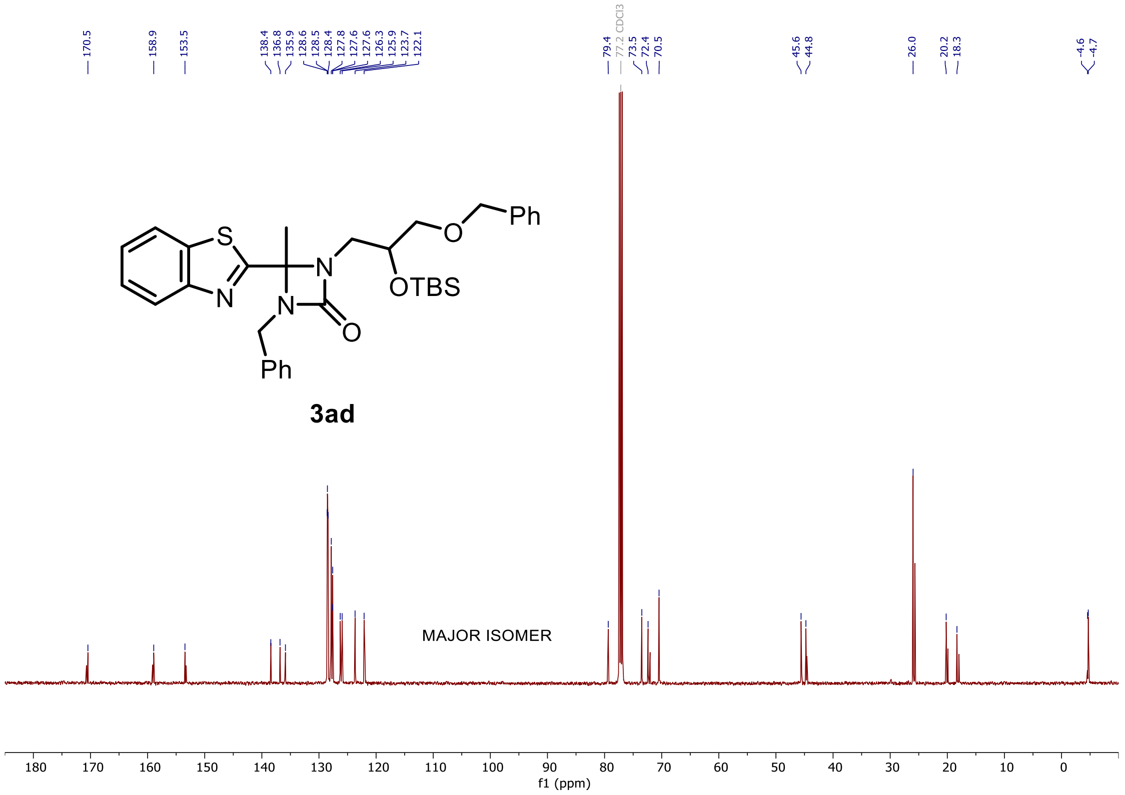

Copy of ^{1}H NMR Spectrum (500 MHz, $CDCl_3$) of **3ad** – minor isomer

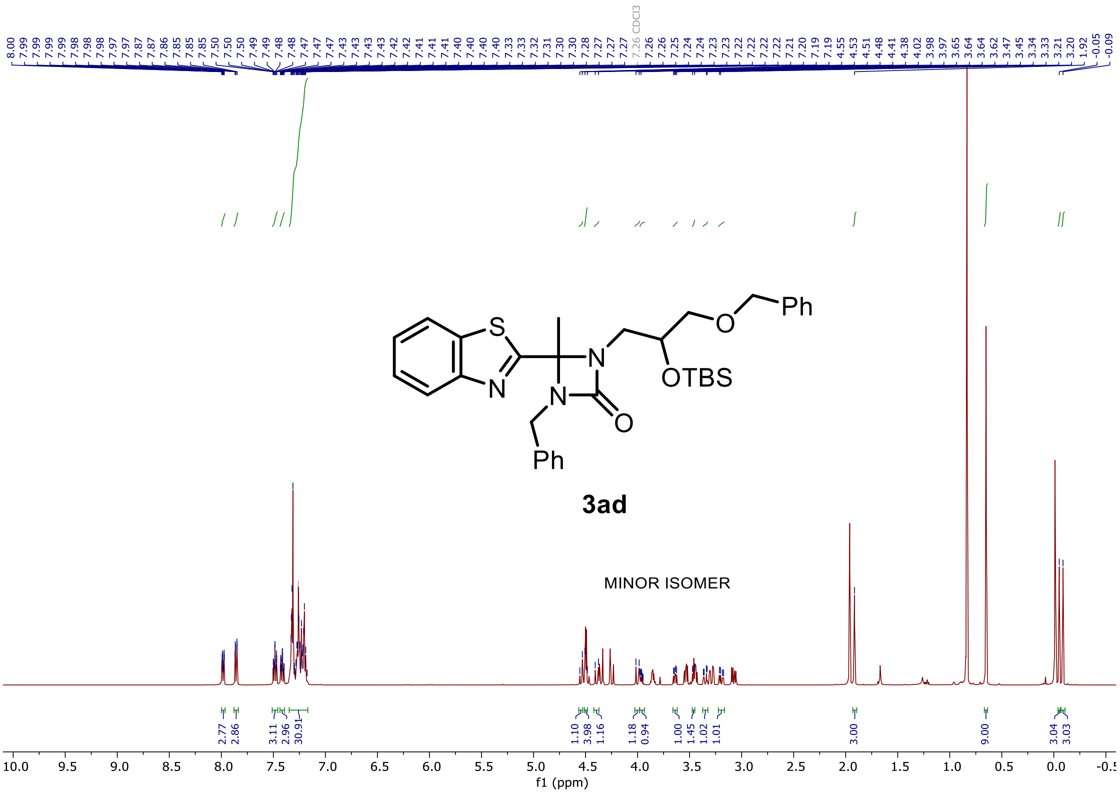

Copy of ^{13}C {^{1}H} NMR Spectrum (126 MHz, $CDCl_3$) of **3ad** – minor isomer

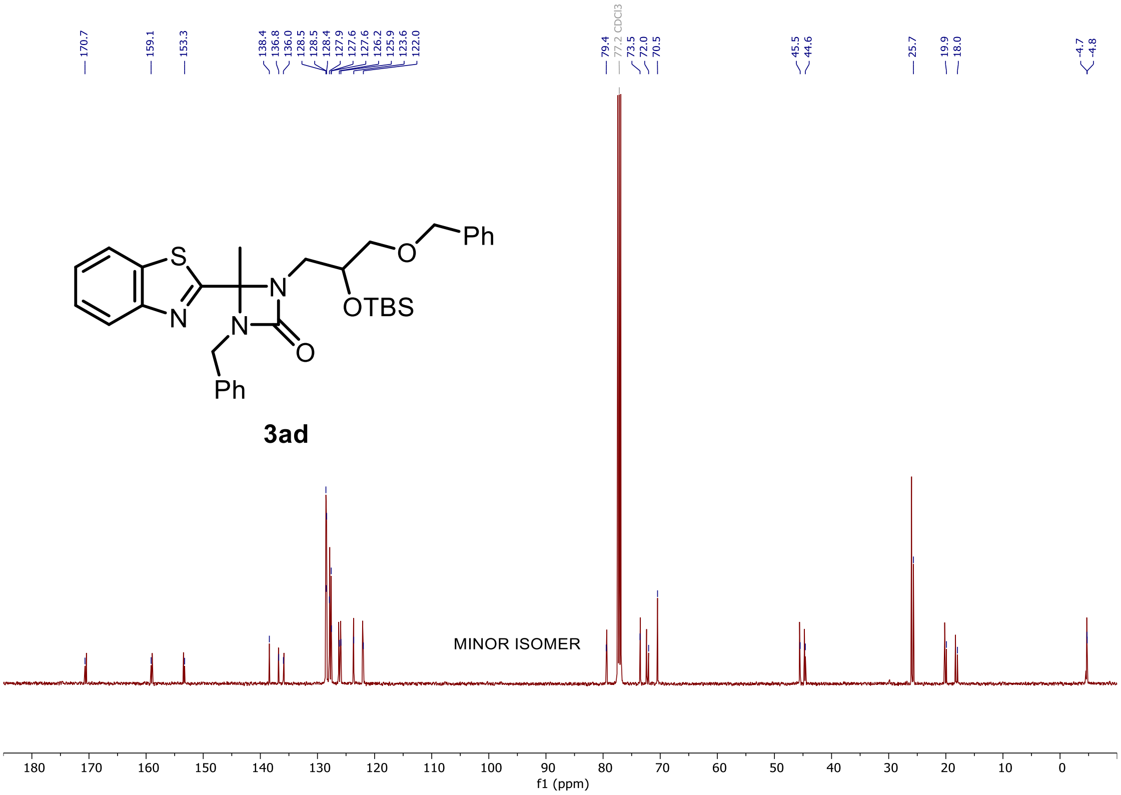

Copy of ^{1}H NMR Spectrum (400 MHz, DMSO-d_6, 80 °C) of **6a**

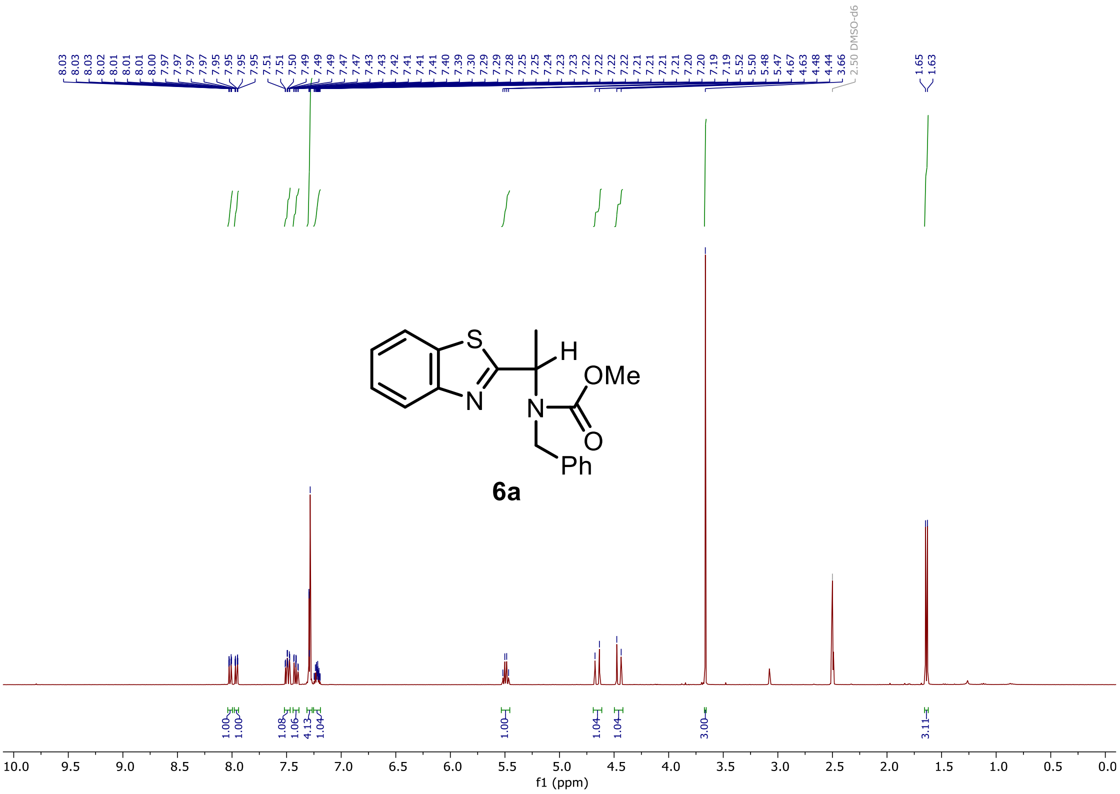

Copy of ^{13}C {^{1}H} NMR Spectrum (101 MHz, DMSO-d_6, 80 °C) of **6a**

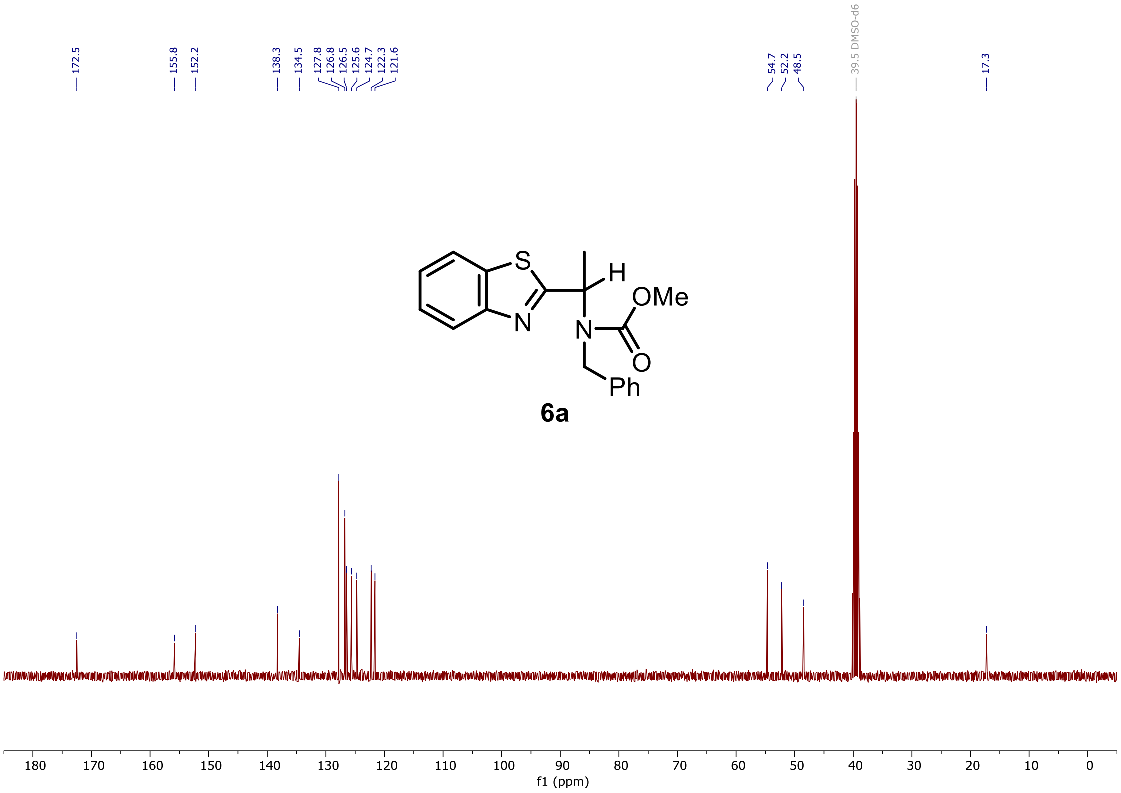

Copy of DEPT NMR Spectrum (101 MHz, DMSO-d_6, 80 °C) of **6a**

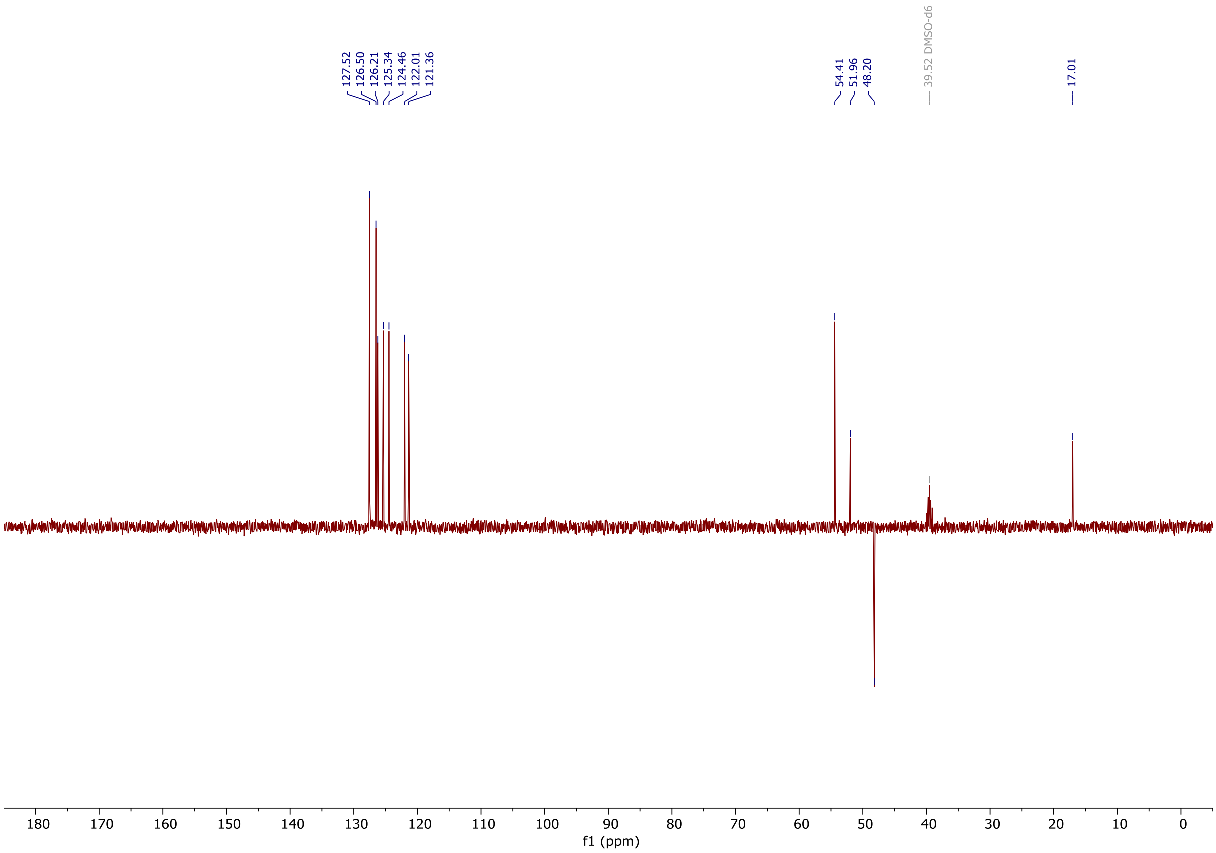

Copy of COSY NMR Spectrum (400 MHz, DMSO-d_6, 80 °C) of **6a**

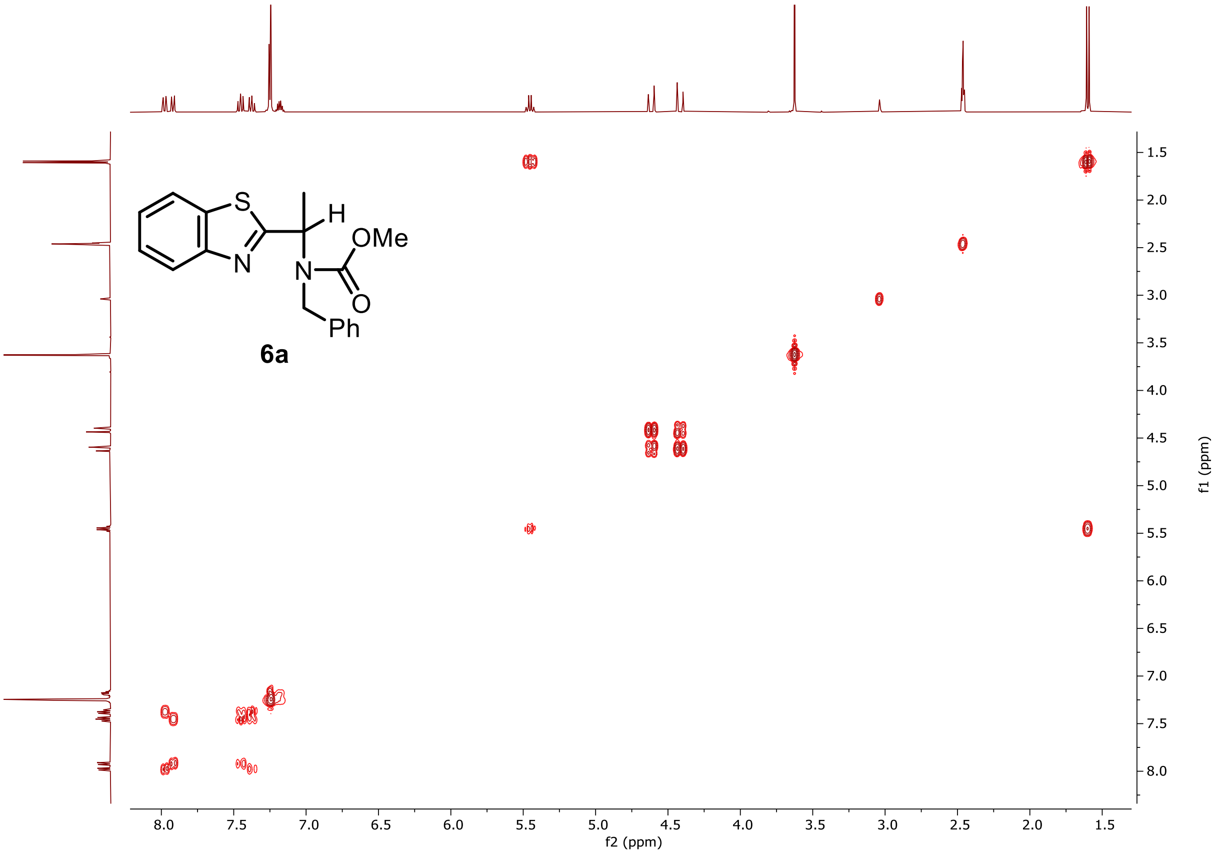

Copy of HMBC NMR Spectrum (400 MHz, DMSO-d_6, 80 °C) of **6a**

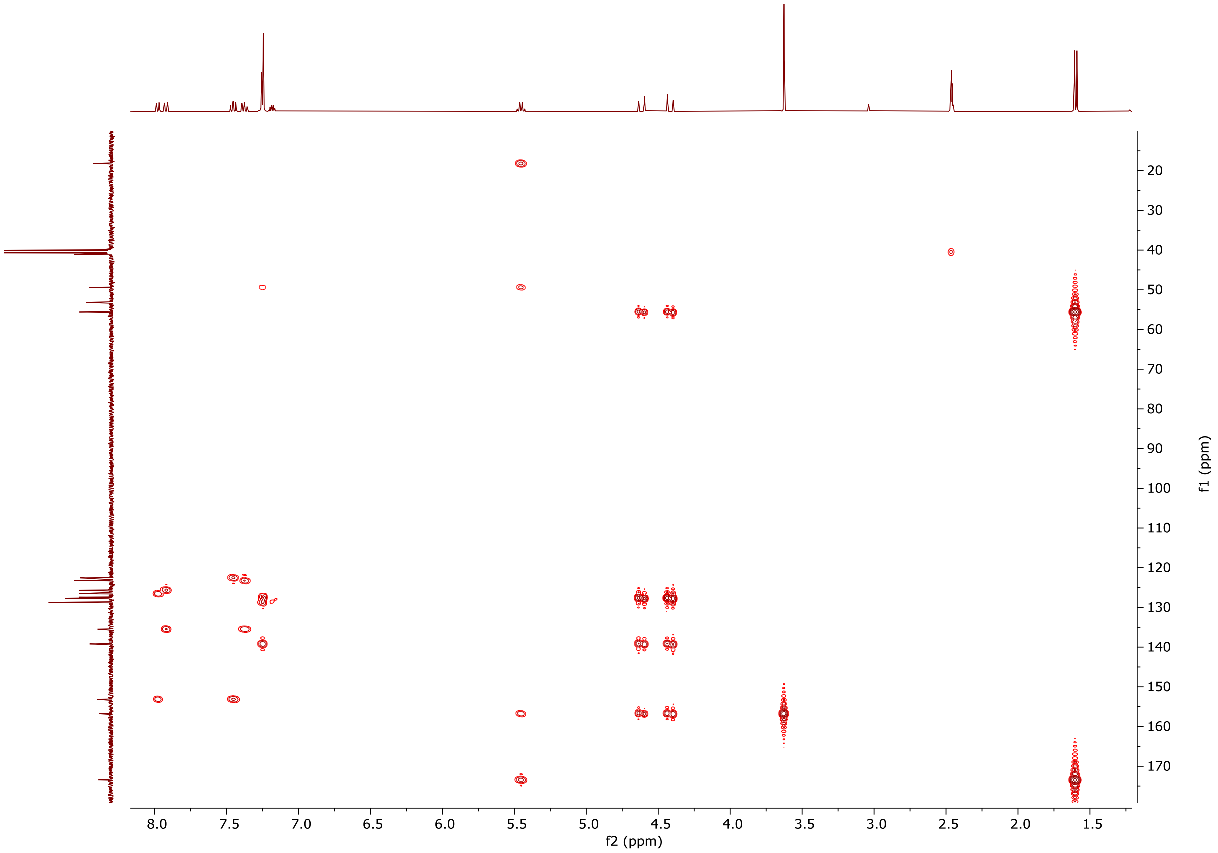

Copy of HMQC NMR Spectrum (400 MHz, DMSO-d_6, 80 °C) of **6a**

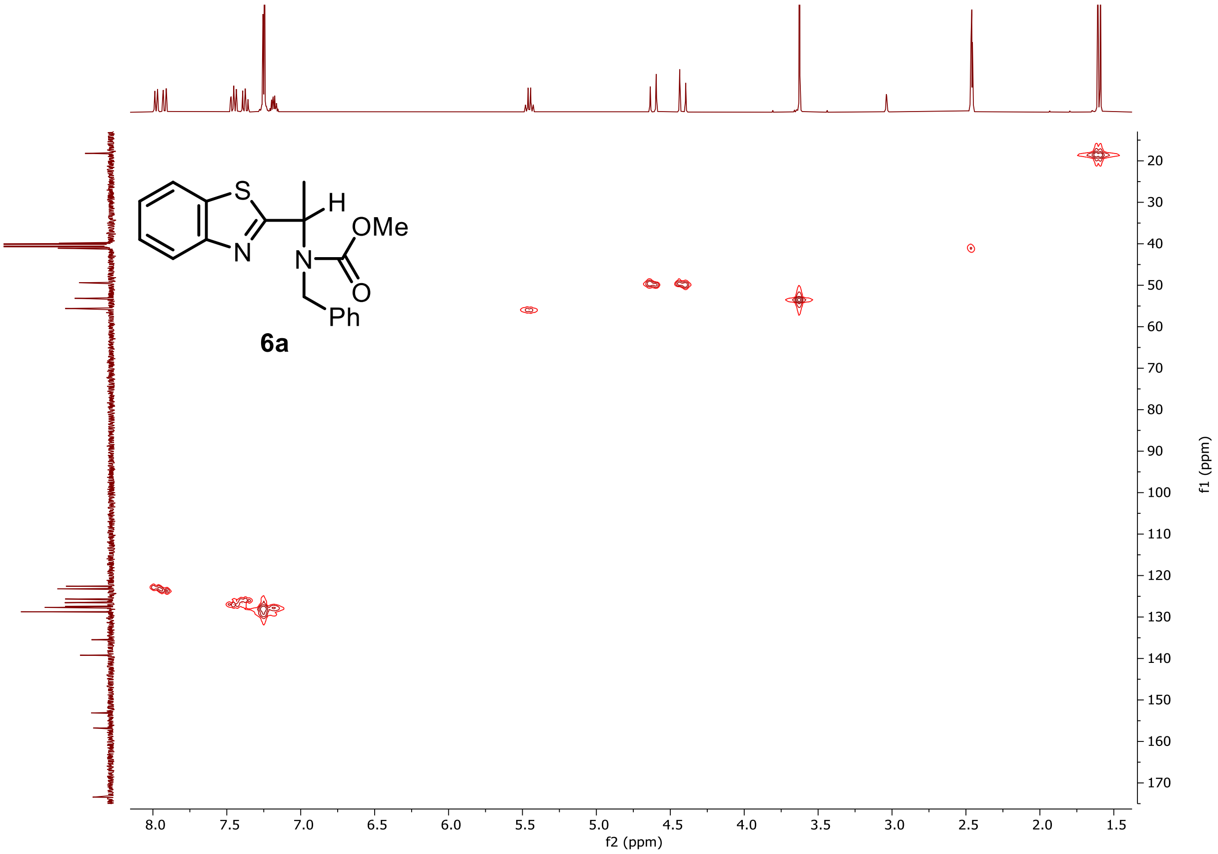

Copy of ^{1}H NMR Spectrum (500 MHz, $CDCl_3$) of **7a** – *major isomer*

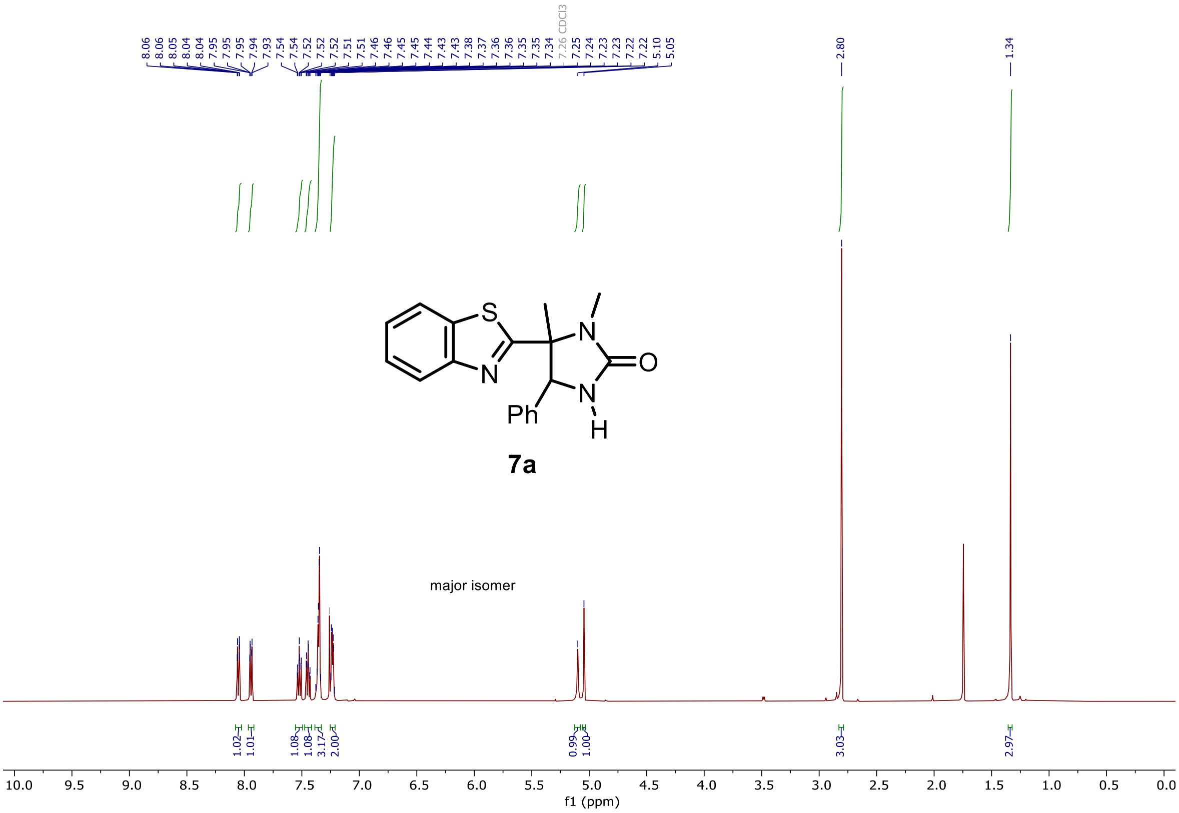

Copy of ^{13}C {^{1}H} NMR Spectrum (126 MHz, $CDCl_3$) of **7a** – *major isomer*

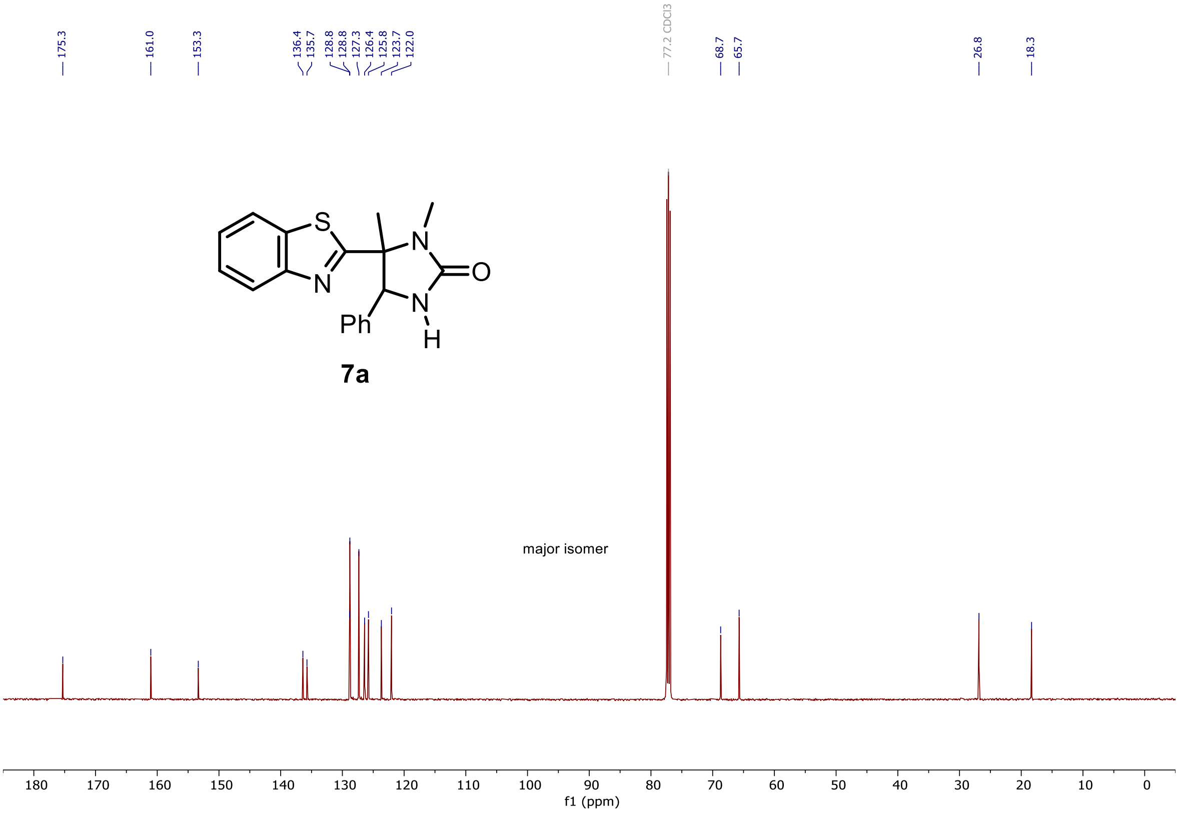

Copy of ^{1}H NMR Spectrum (500 MHz, $CDCl_3$) of **7a** – *minor isomer*

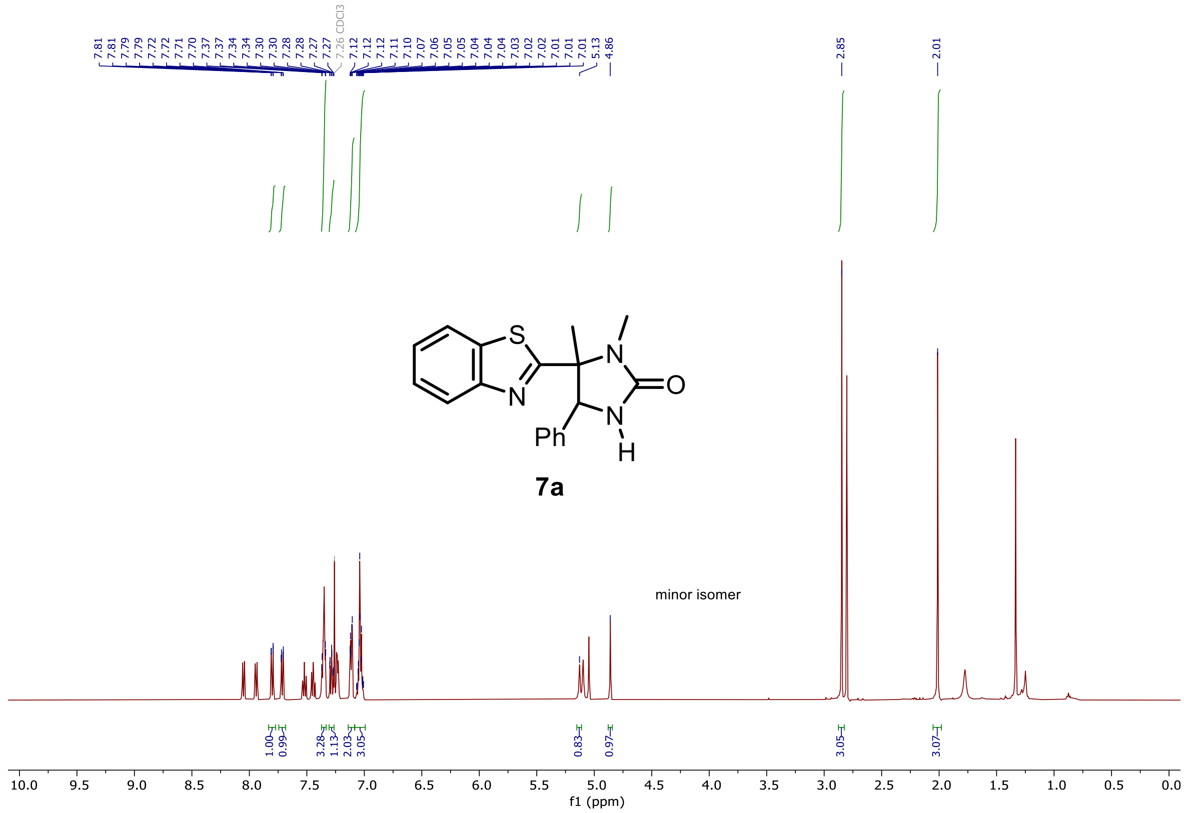

Copy of ^{13}C {^{1}H} NMR Spectrum (126 MHz, $CDCl_3$) of **7a** – *minor isomer*

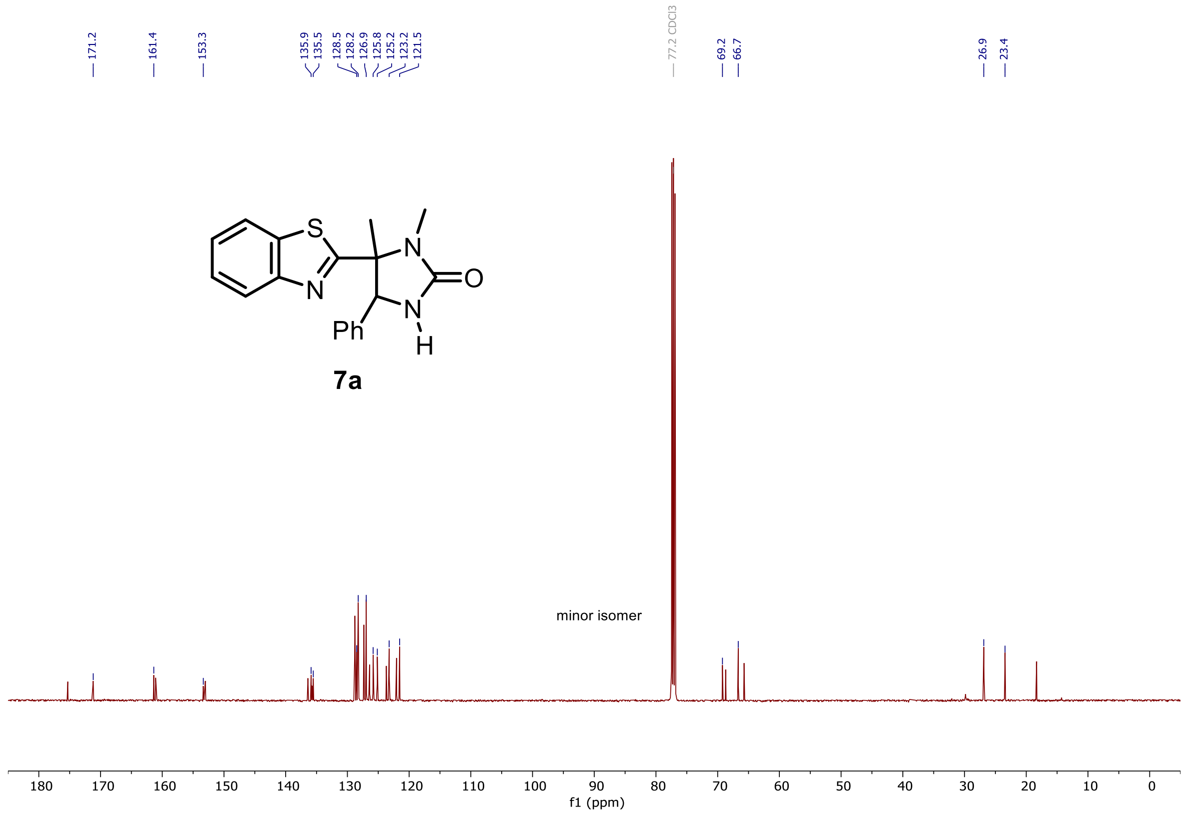

Copy of ^{1}H NMR Spectrum (500 MHz, $CDCl_3$) of **7b** – *major isomer*

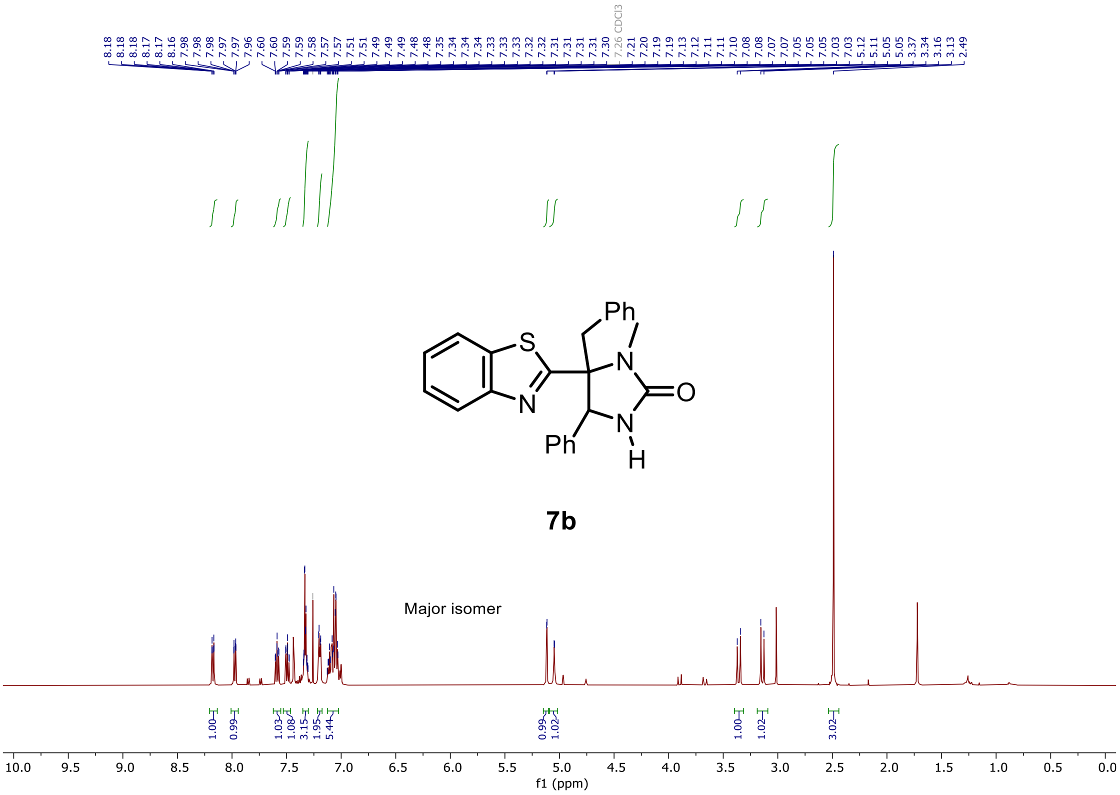

Copy of ^{13}C {^{1}H} NMR Spectrum (126 MHz, $CDCl_3$) of **7b** – *major isomer*

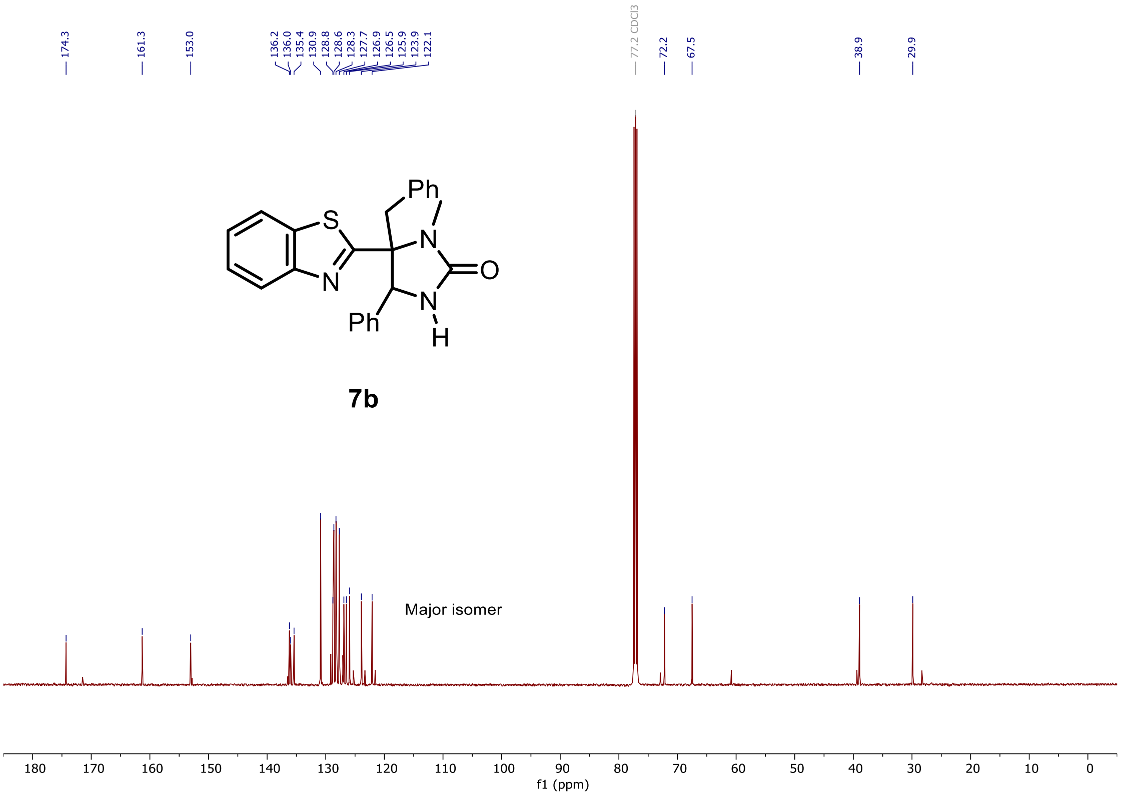

Copy of ^{1}H NMR Spectrum (500 MHz, $CDCl_3$) of **7b** – *minor isomer*

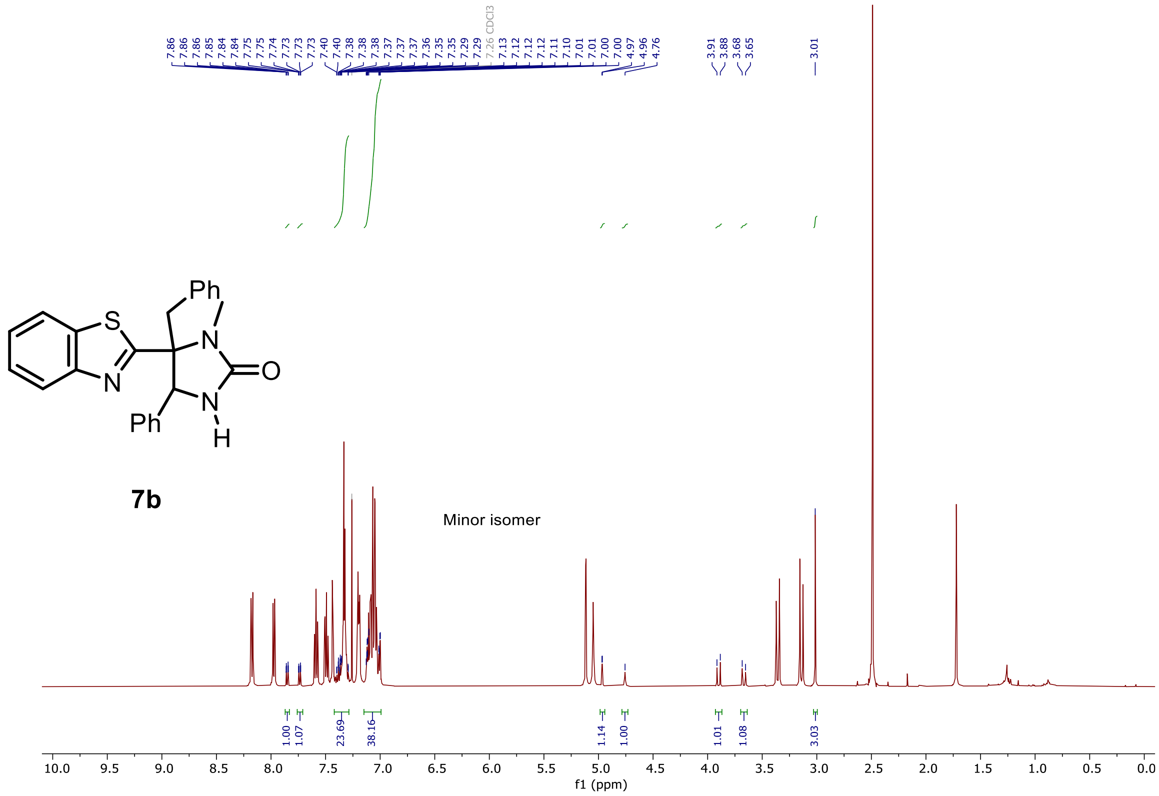

Copy of ^{13}C {^{1}H} NMR Spectrum (126 MHz, $CDCl_3$) of **7b** – *minor isomer*

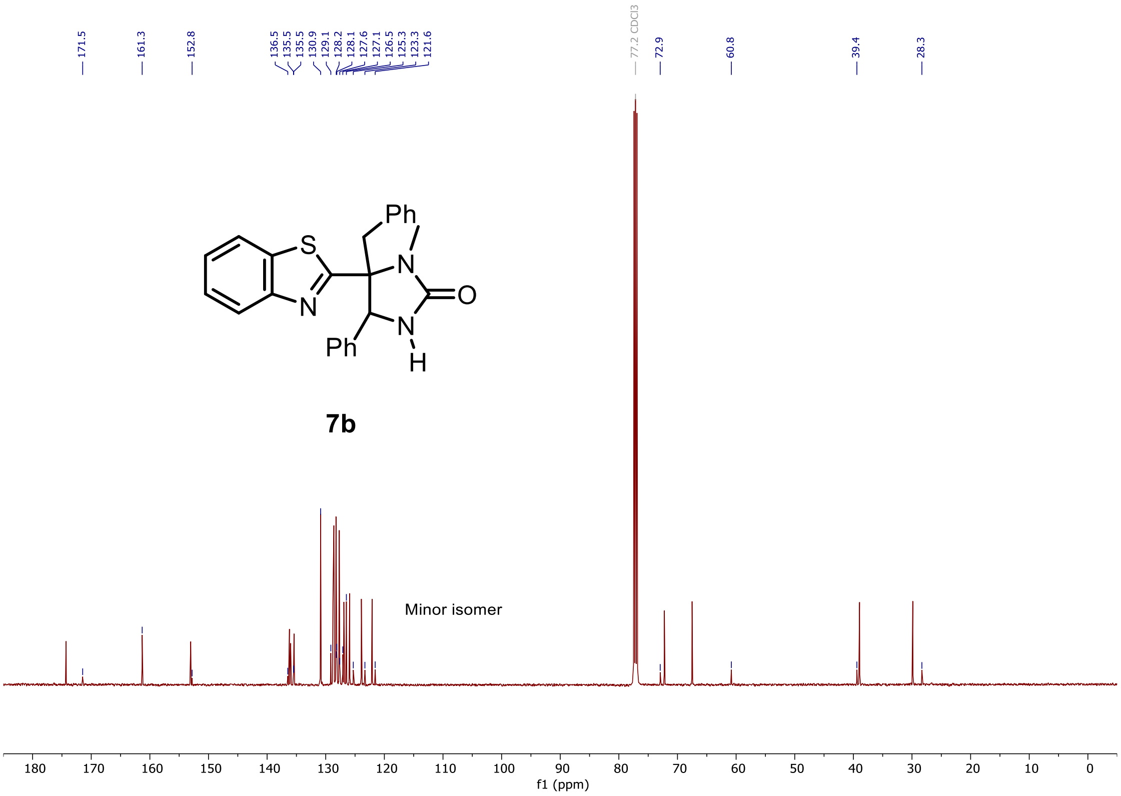

Copy of COSY NMR Spectrum (500 MHz, $CDCl_3$) of **7b**

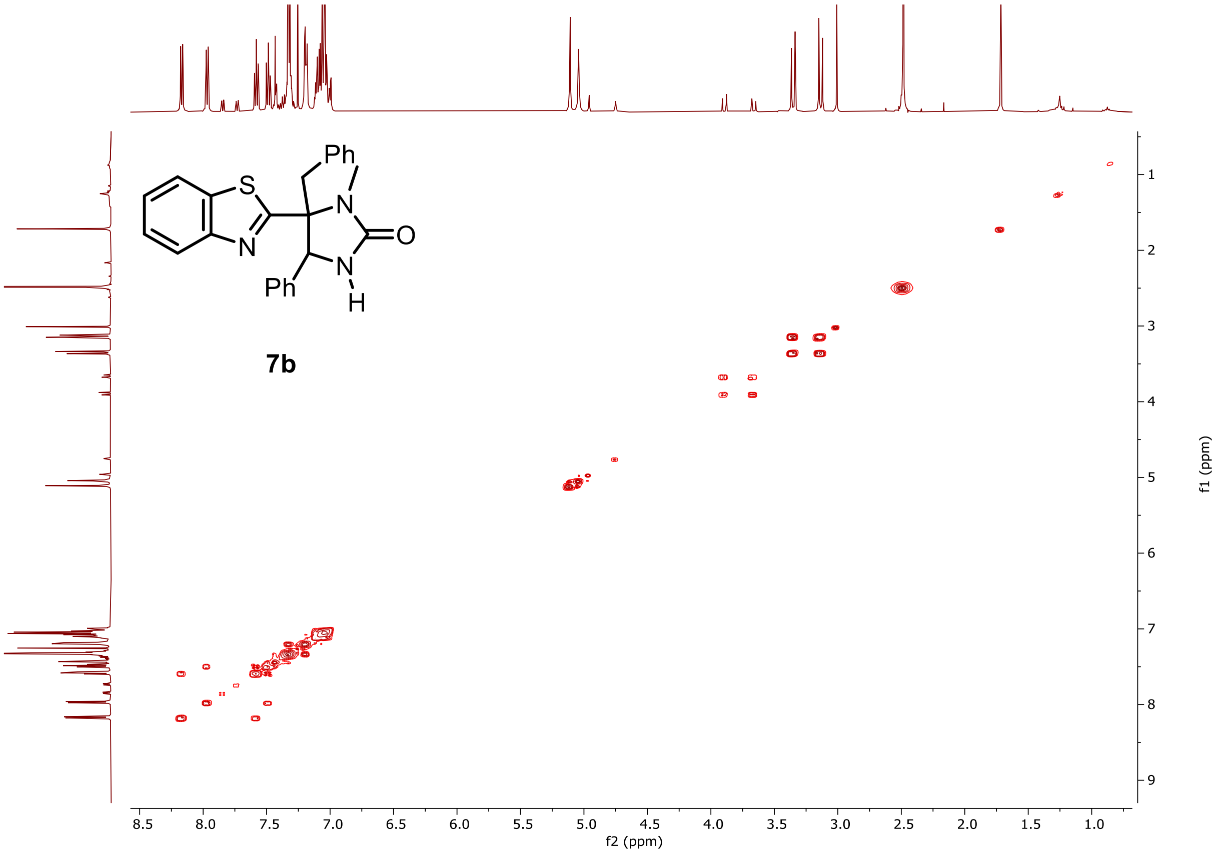

Copy of HMBC NMR Spectrum (500 MHz, $CDCl_3$) of **7b**

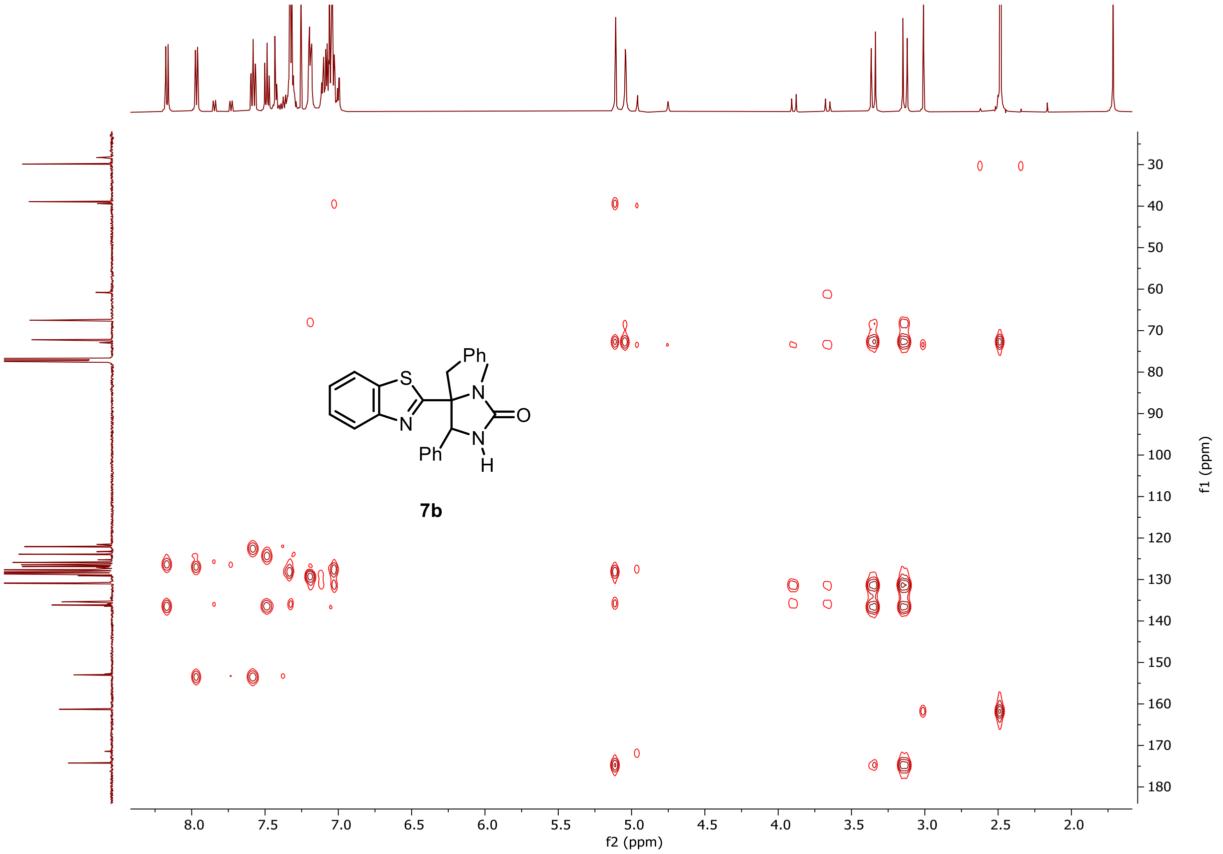

Copy of HMQC NMR Spectrum (500 MHz, $CDCl_3$) of **7b**

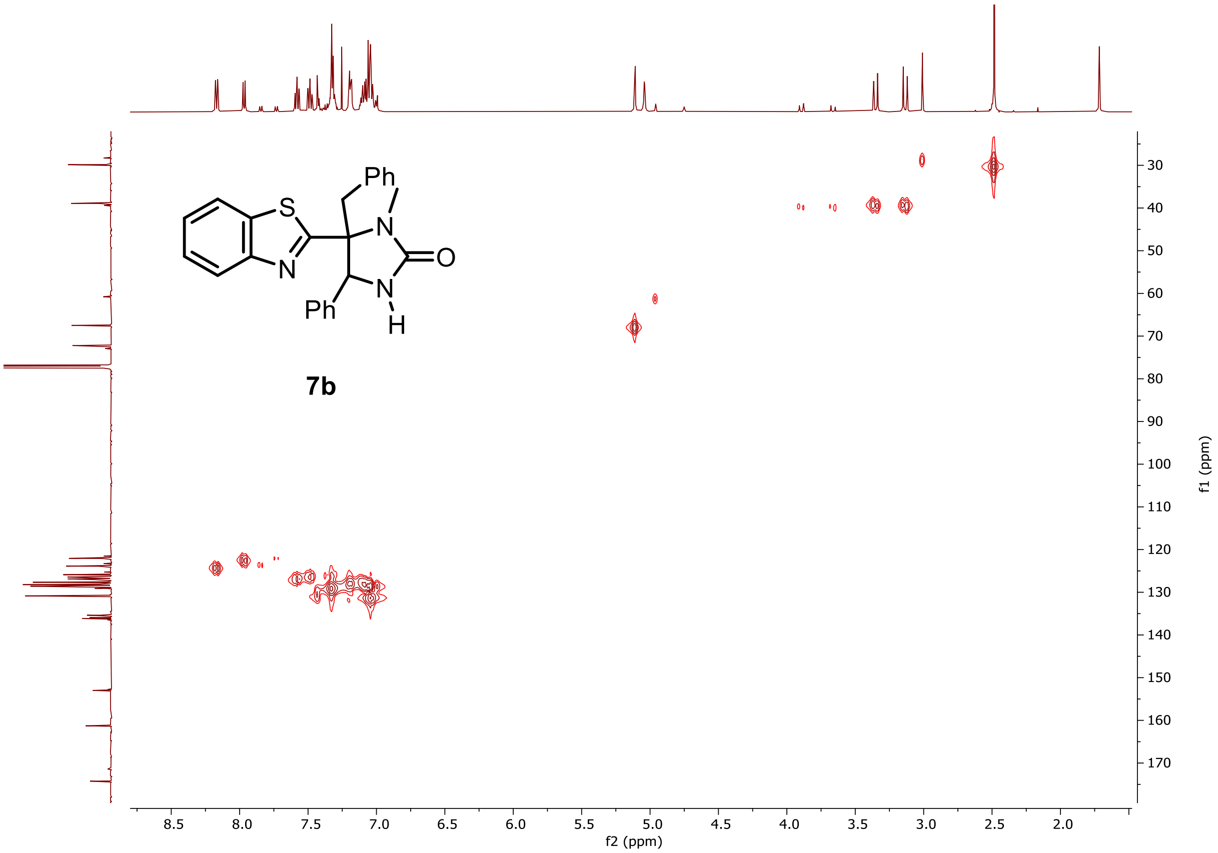

Copy of ^{1}H NMR Spectrum (400 MHz, $CDCl_3$) of **7n**

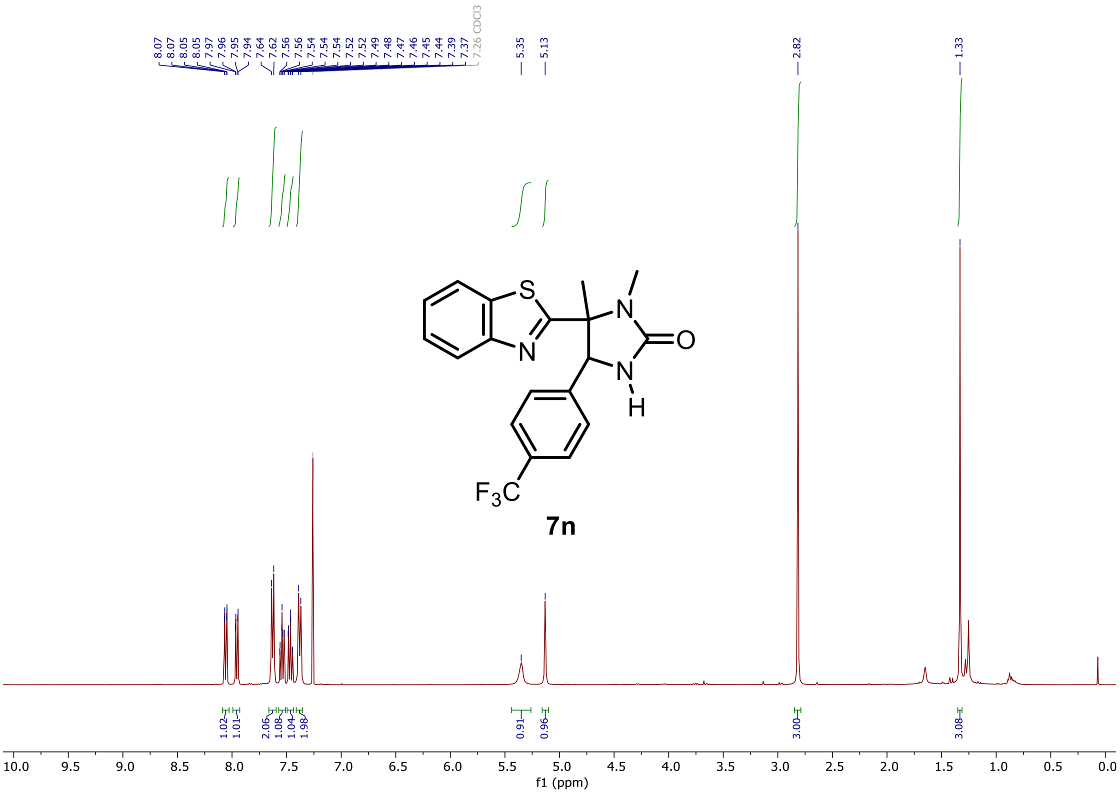

Copy of ^{13}C {^{1}H, ^{19}F} NMR Spectrum (101 MHz, $CDCl_3$) of **7n**

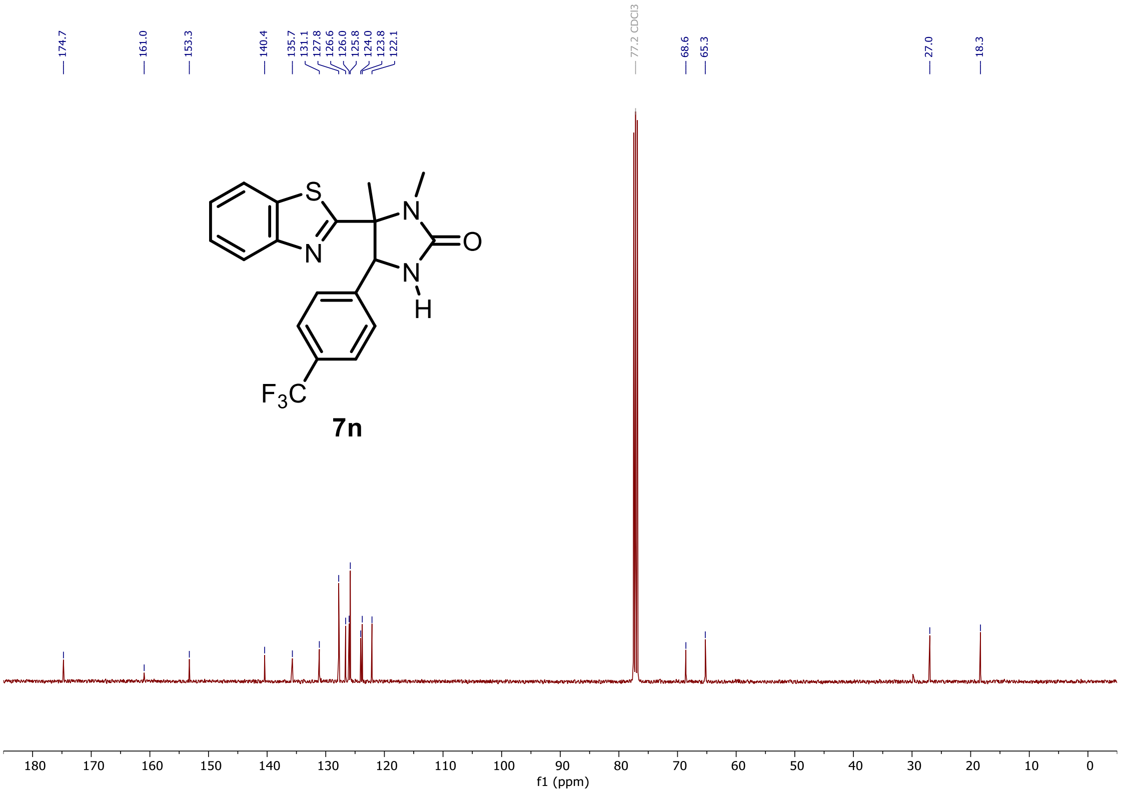

Copy of ^{19}F NMR Spectrum (376 MHz, $CDCl_3$) of **7n**

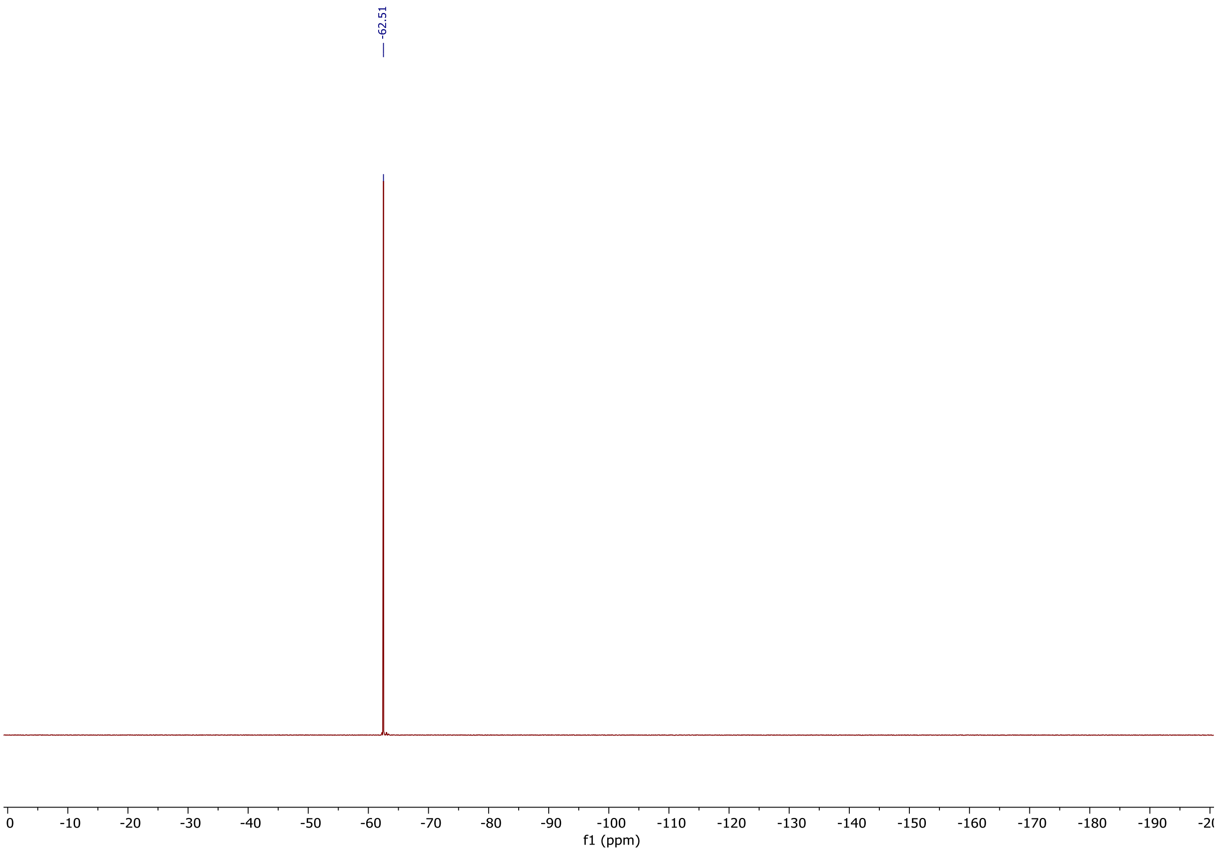

Copy of ^{1}H NMR Spectrum (400 MHz, $CDCl_3$) of **9**

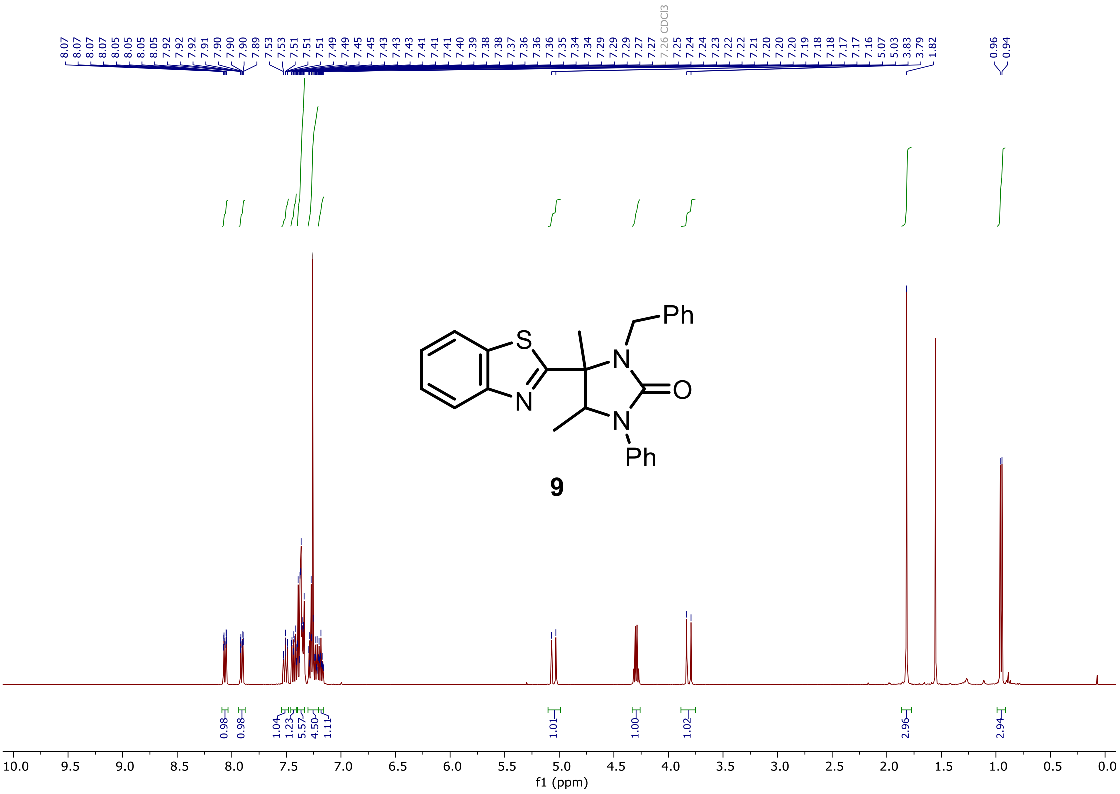

Copy of ^{13}C {^{1}H} NMR Spectrum (126 MHz, $CDCl_3$) of **9**

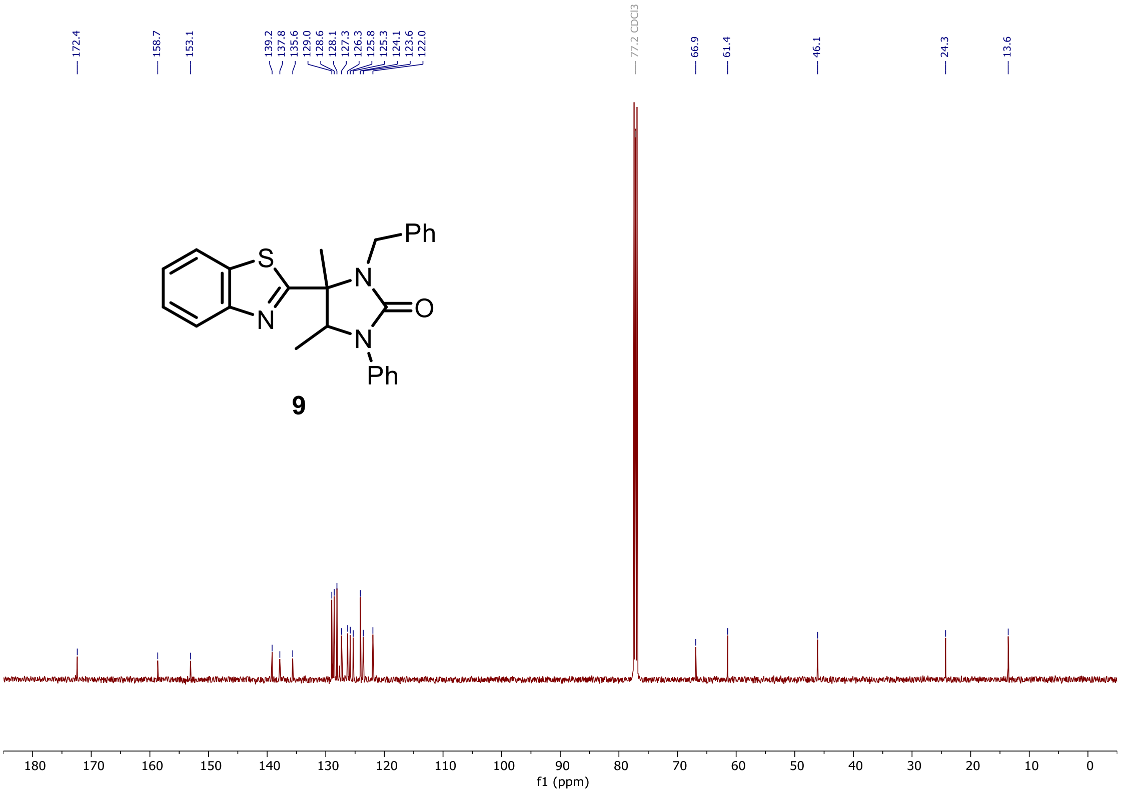

Copy of ^{1}H NMR Spectrum (400 MHz, $CDCl_3$) of **10**

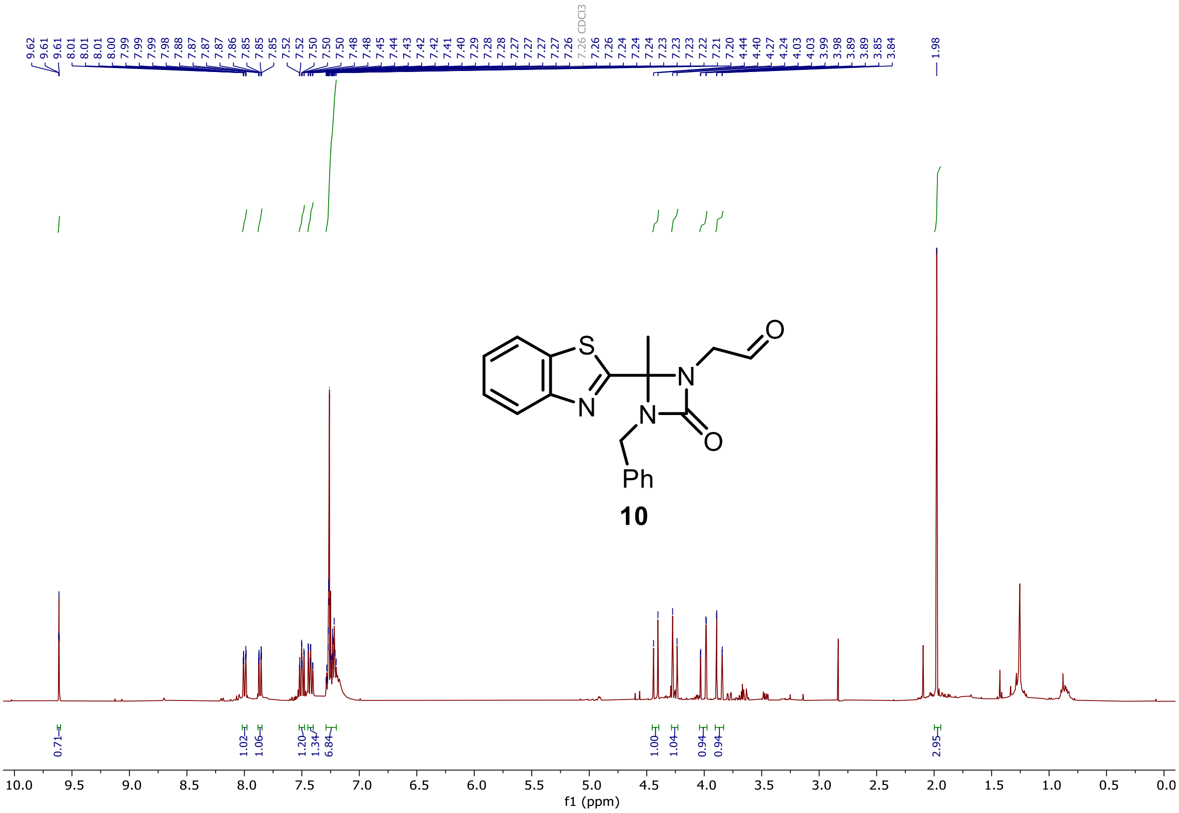

Copy of ^{13}C {^{1}H} NMR Spectrum (101 MHz, $CDCl_3$) of **10**

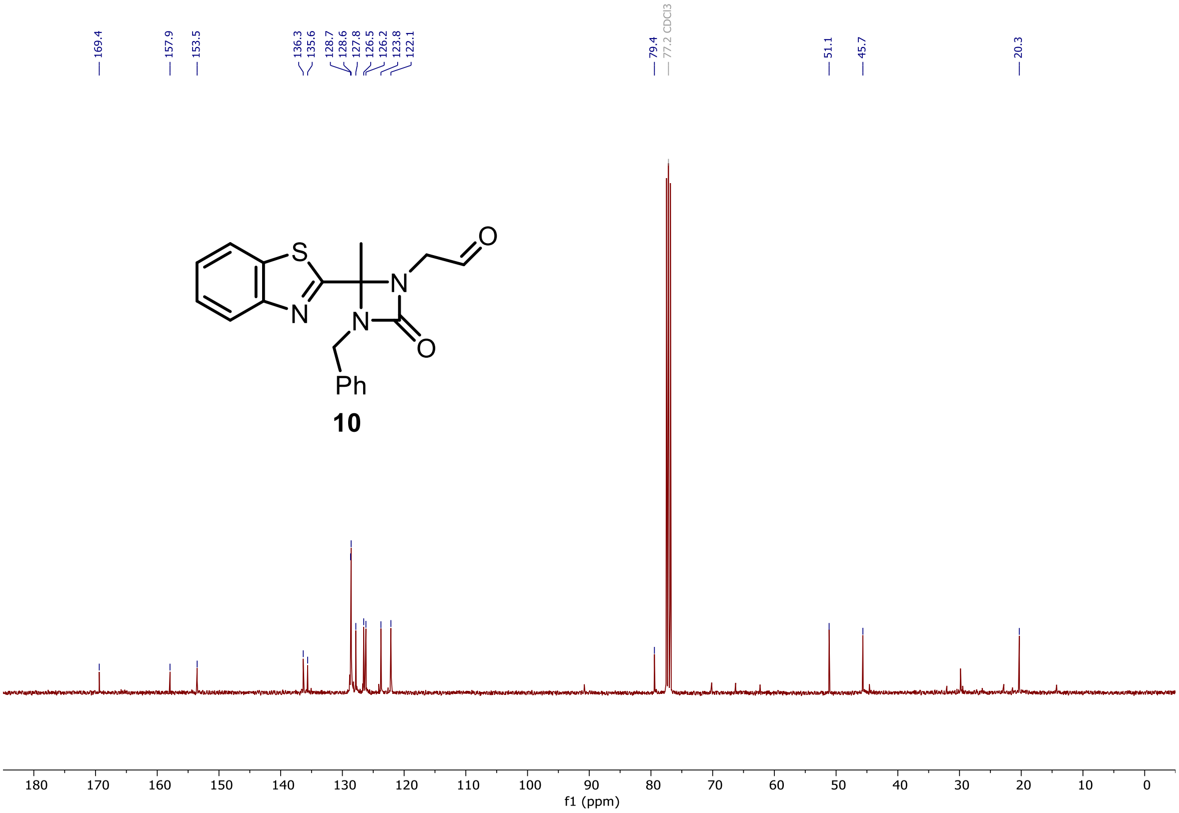

Copy of ^{1}H NMR Spectrum (400 MHz, $CDCl_3$) of **11**

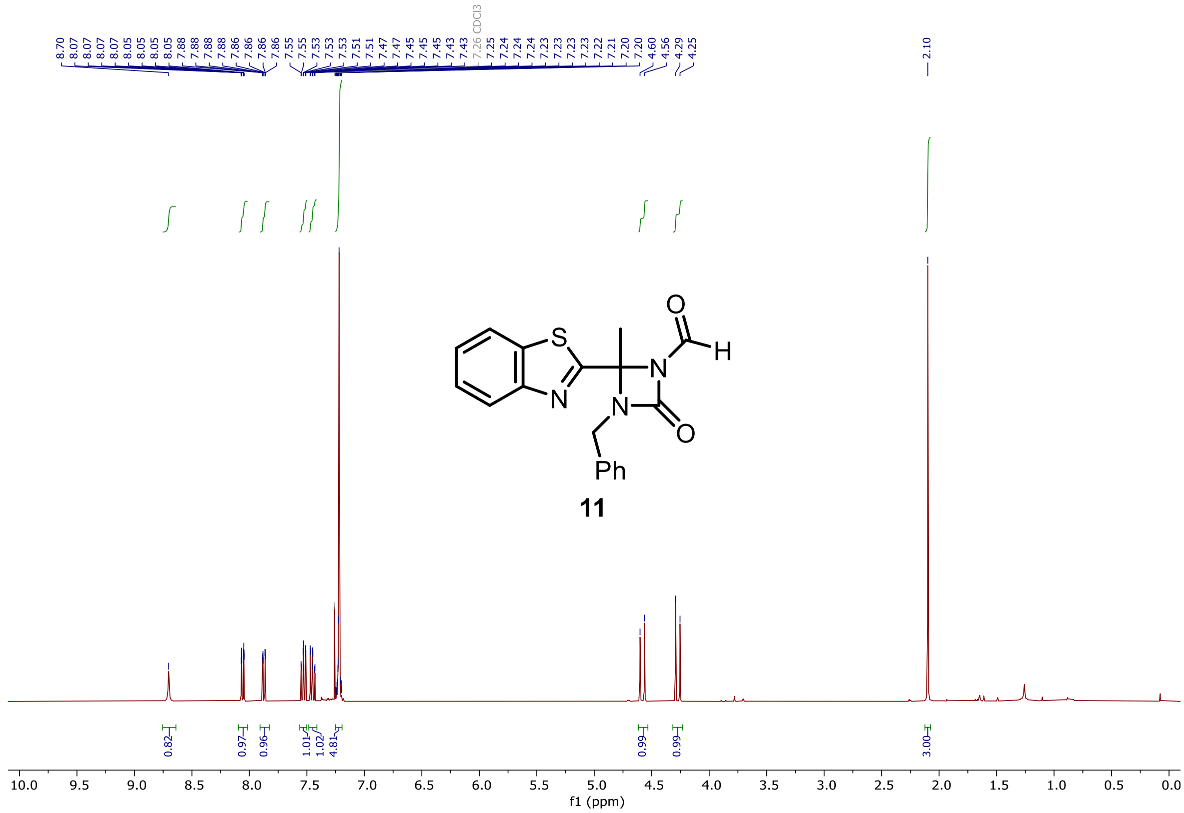

Copy of ^{13}C {^{1}H} NMR Spectrum (101 MHz, $CDCl_3$) of **11**

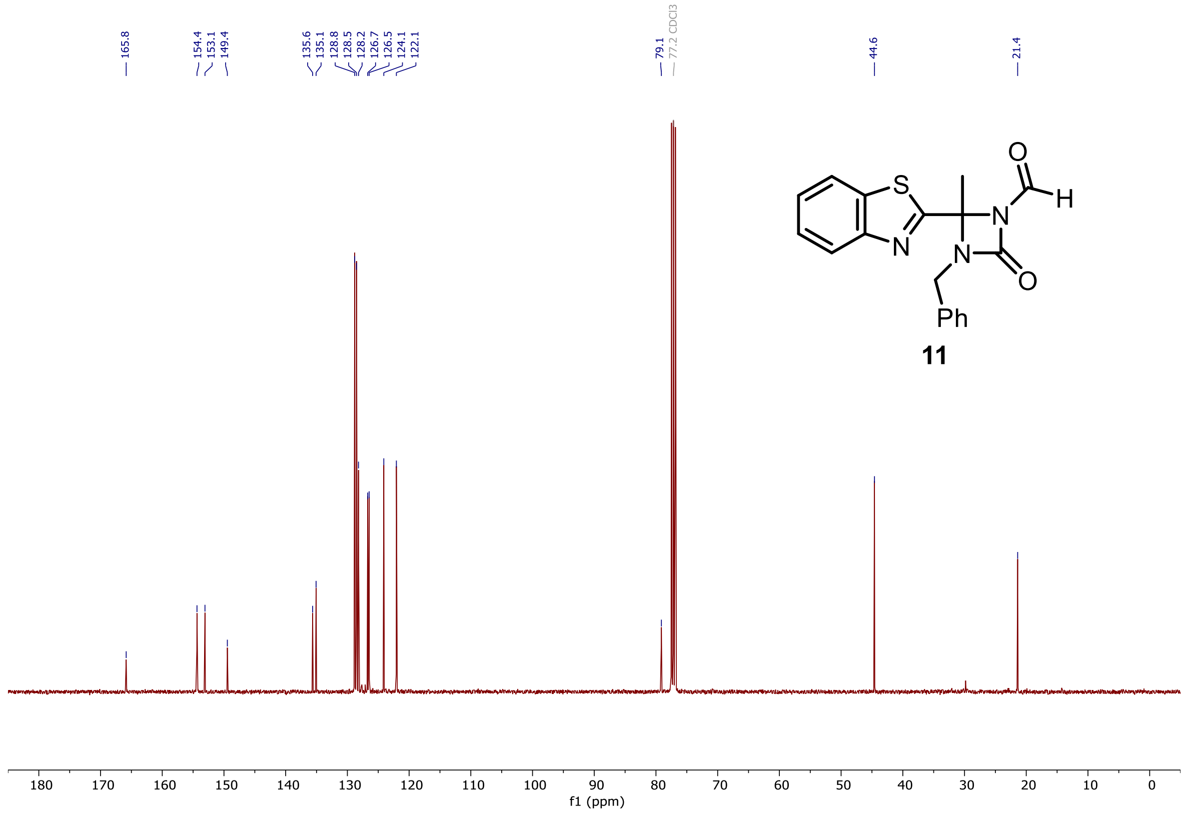

Copy of ^{1}H NMR Spectrum (400 MHz, $CDCl_3$) of **12**

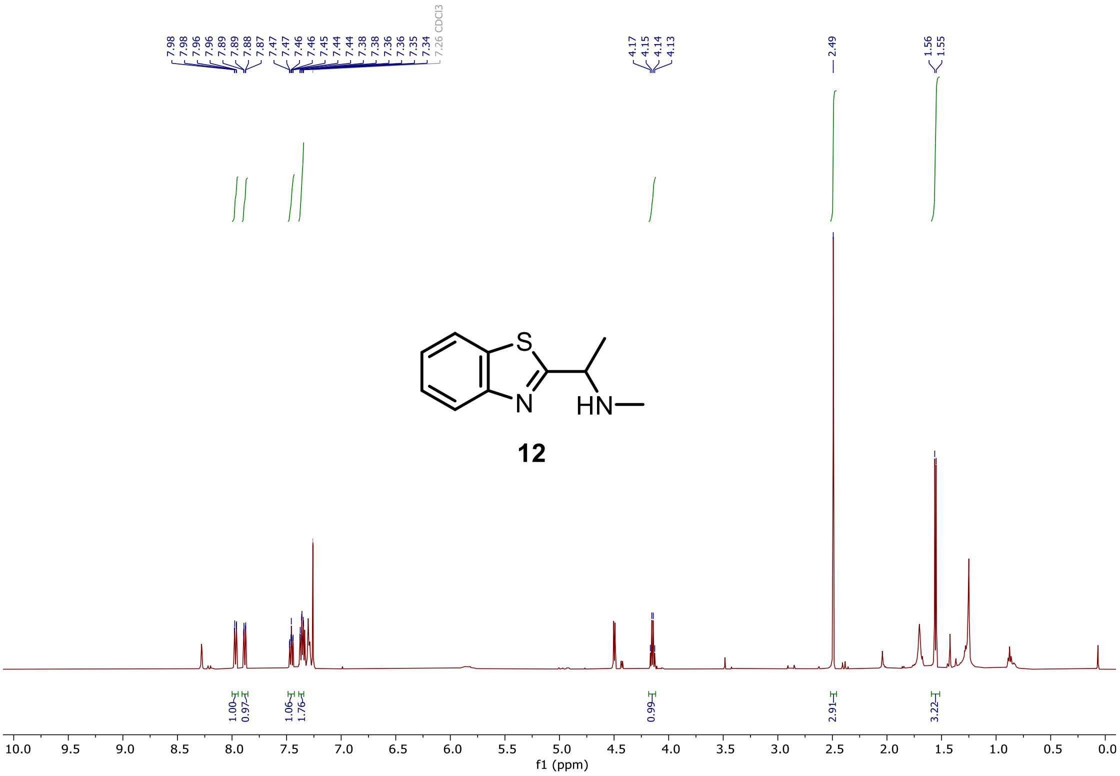

Copy of ^{13}C {^{1}H} NMR Spectrum (101 MHz, $CDCl_3$) of **12**

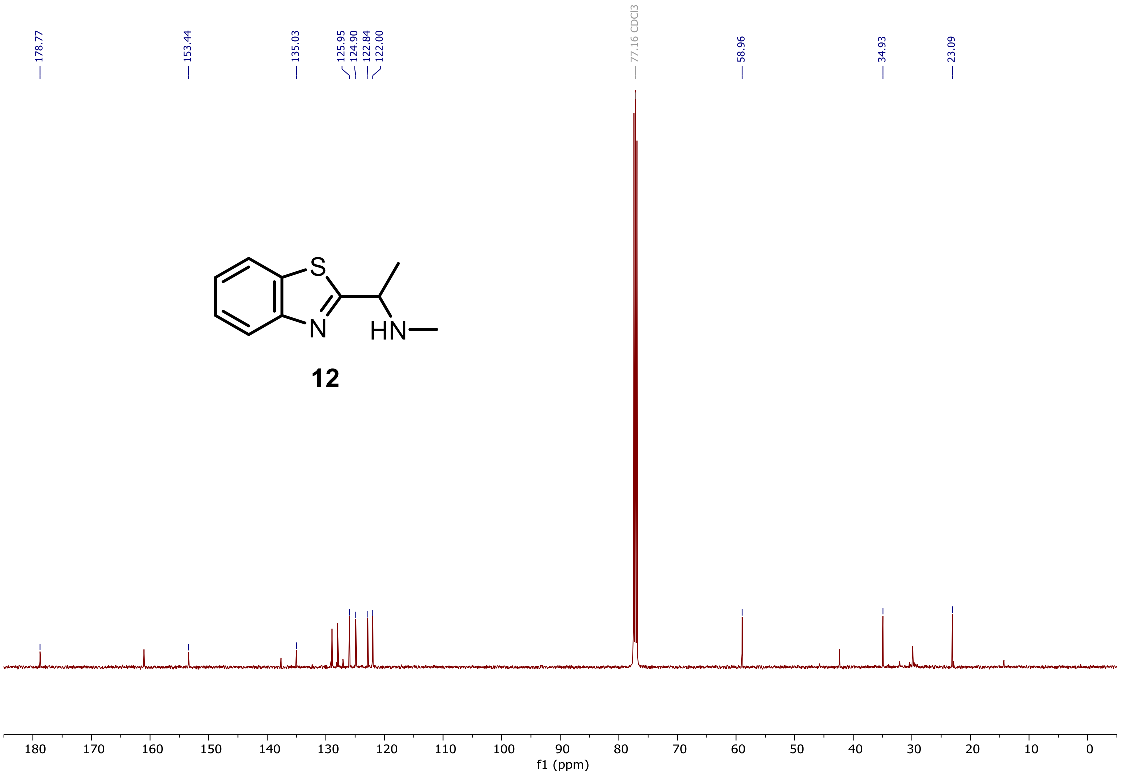

Copy of ^{1}H NMR Spectrum (400 MHz, $CDCl_3$) of **13**

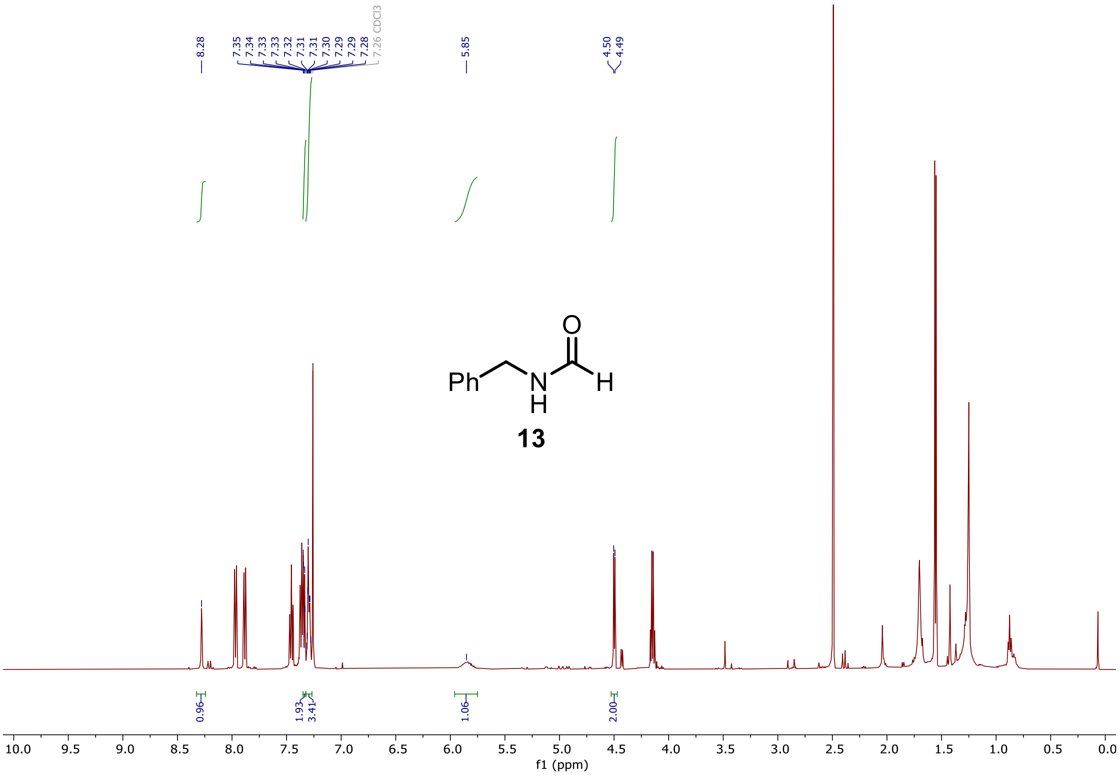

Copy of $^{13}C\{^{1}H\}$ NMR Spectrum (101 MHz, $CDCl_3$) of **13**

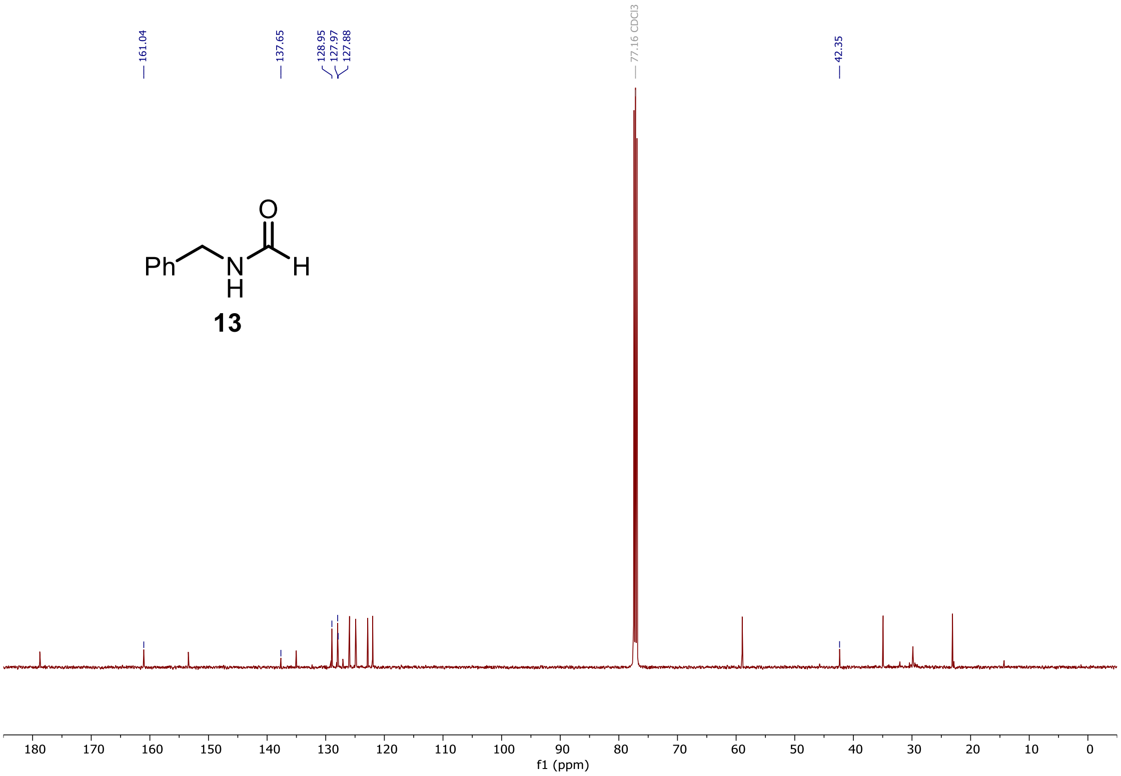

Copy of ^{1}H NMR Spectrum (400 MHz, $CDCl_3$) of **S3**

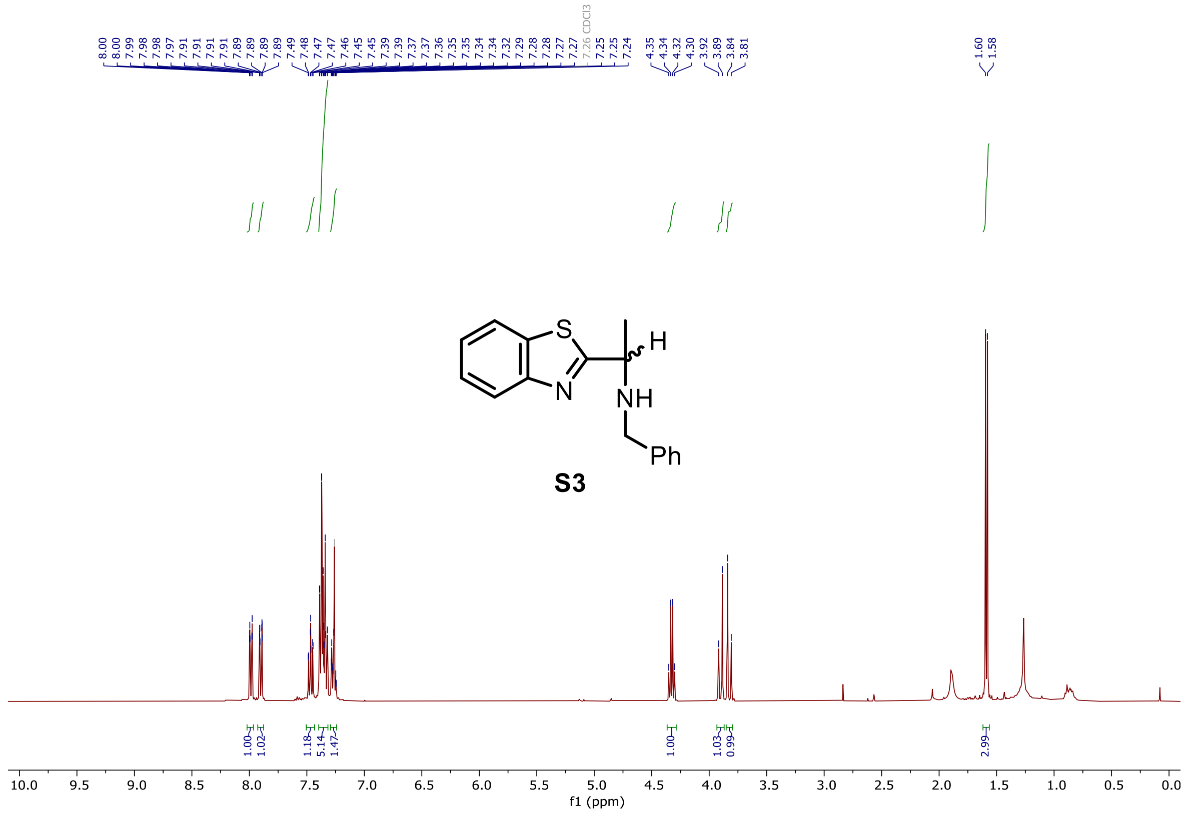

Copy of ^{13}C {^{1}H} NMR Spectrum (101 MHz, $CDCl_3$) of **S3**

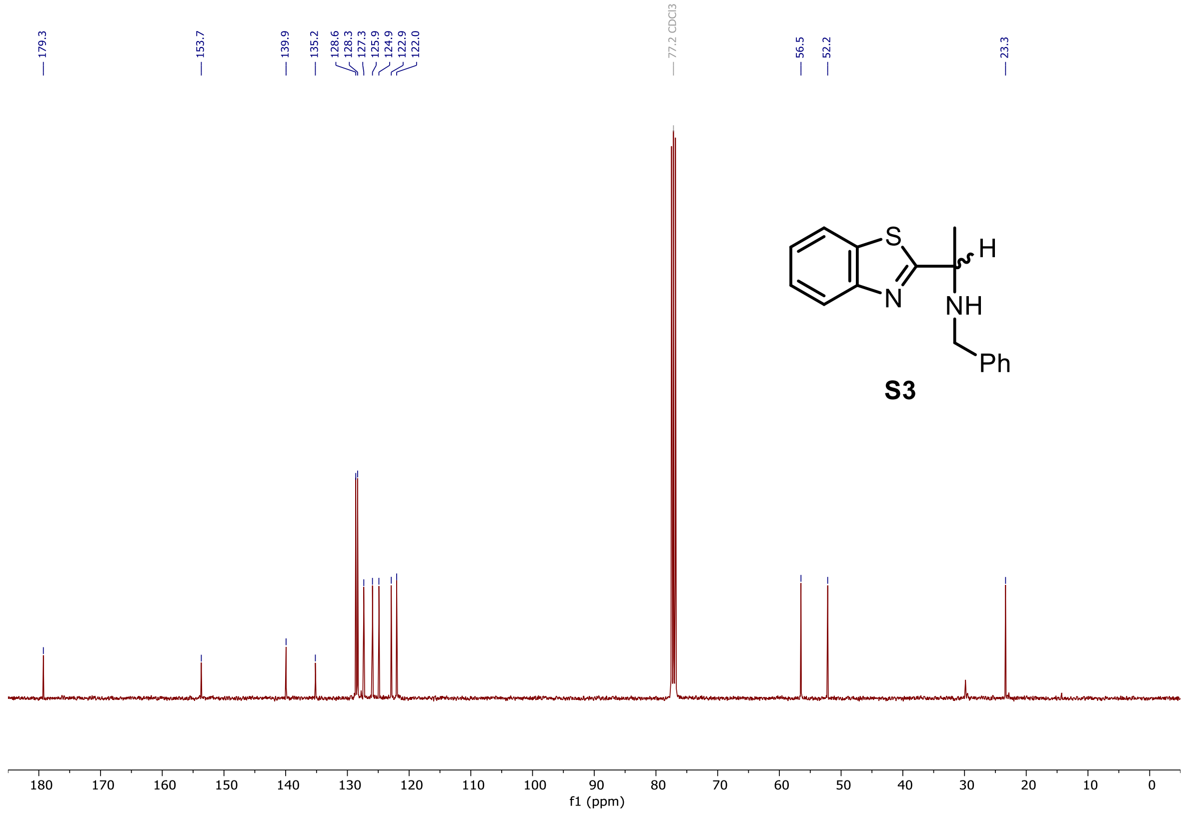

Supplement: Supplementary file 1 [file ol6c01451_si_001.pdf]
